# Causal association between circulating blood cell traits and pulmonary embolism: a mendelian randomization study

**DOI:** 10.1186/s12959-024-00618-3

**Published:** 2024-06-11

**Authors:** Chen Jiang, Jianing Lin, Bin Xie, Meijuan Peng, Ziyu Dai, Suyin Mai, Qiong Chen

**Affiliations:** 1 Department of Geriatrics, Respiratory Medicine, Xiangya Hospital, Central South University, Changsha, 410008 China; 2 National Clinical Research Center for Geriatric Disorders, Xiangya Hospital, Central South University, Changsha, 410008 China

**Keywords:** Pulmonary embolism, Lymphocyte subsets, Peripheral blood cell, Mendelian randomization, HLA-DR^+^ NK cells

## Abstract

**Background:**

Pulmonary embolism (PE) is a life-threatening thromboembolic disease for which there is limited evidence for effective prevention and treatment. Our goal was to determine whether genetically predicted circulating blood cell traits could influence the incidence of PE.

**Methods:**

Using single variable Mendelian randomization (SVMR) and multivariate Mendelian randomization (MVMR) analyses, we identified genetic associations between circulating blood cell counts and lymphocyte subsets and PE. GWAS blood cell characterization summary statistics were compiled from the Blood Cell Consortium. The lymphocyte subpopulation counts were extracted from summary GWAS statistics for samples from 3757 individuals that had been analyzed by flow cytometry. GWAS data related to PE were obtained from the FinnGen study.

**Results:**

According to the SVMR and reverse MR, increased levels of circulating white blood cells (odds ratio [OR]: 0.88, 95% confidence interval [CI]: 0.81-0.95, *p* = 0.0079), lymphocytes (OR: 0.90, 95% CI: 0.84-0.97, *p* = 0.0115), and neutrophils (OR: 0.88, 95% CI: 0.81–0.96, *p* = 0.0108) were causally associated with PE susceptibility. MVMR analysis revealed that lower circulating lymphocyte counts (OR: 0.84, 95% CI: 0.75-0.94, *p* = 0.0139) were an independent predictor of PE. According to further MR results, this association may be primarily related to HLA-DR^+^ natural killer (NK) cells.

**Conclusions:**

Among European populations, there is a causal association between genetically predicted low circulating lymphocyte counts, particularly low HLA-DR^+^ NK cells, and an increased risk of PE. This finding supports observational studies that link peripheral blood cells to PE and provides recommendations for predicting and preventing this condition.

**Graphical Abstract:**

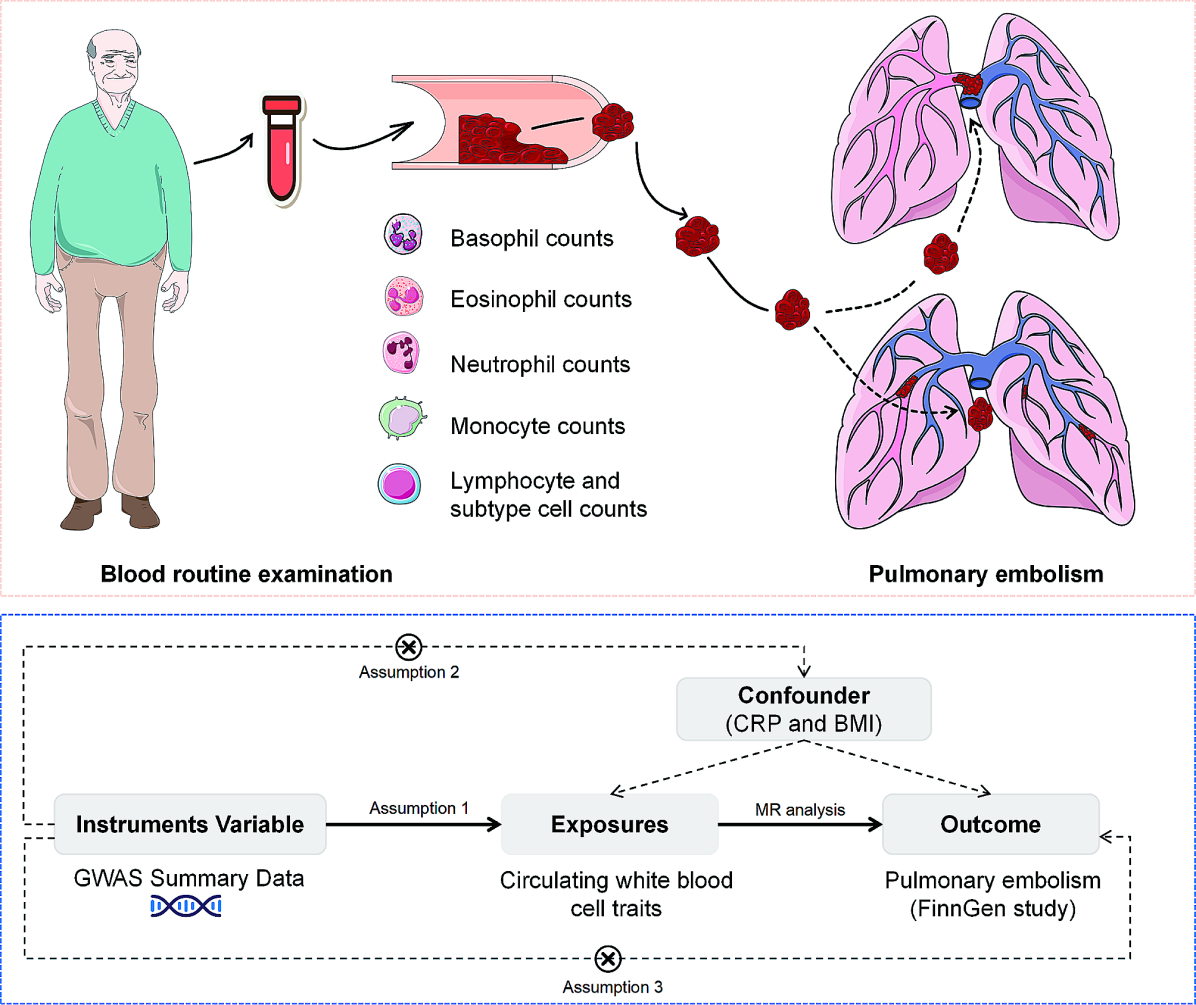

**Supplementary Information:**

The online version contains supplementary material available at 10.1186/s12959-024-00618-3.

## Introduction

Pulmonary embolism (PE) is a life-threatening lung condition caused by embolisms that enter and occlude a distal pulmonary artery [1]. Studies have reported that the annual incidence of PE ranges from 10 to 417 cases per 100,000 individuals, with the rate increasing as age advances [2, 3]. A number of risk factors for PE have been identified, including recent surgery, infection, inflammation, obesity, and genetic factors [4]. Given the high mortality rate of up to 20% and the frequent occurrence of complications among survivors, preventing PE is critically important [5]. To date, there is limited evidence on the effective prevention and treatment of PE. Genetic factors may play an essential role in the pathogenesis of PE. Multiple genomic variants associated with an increased genetic risk of PE have been identified [6]. PE is often categorized together with deep vein thrombosis (DVT) as venous thromboembolism (VTE), and they both share common risk factors, including venous stasis, hypercoagulability, and endothelial damage. Two large-scale genome-wide association studies (GWASs) have identified 34 and 22 independent genetic signals associated with VTE risk [7, 8], primarily involving loci related to coagulation and fibrinolytic, respectively [9, 10]. In addition, circulating cytokines, blood pressure and blood metabolites have been linked to an increased risk of VTE [11–13]. However, given the pathophysiological differences between PE and DVT and the current limited understanding of the genetic contributions to PE, further exploration into the genetics of PE is valuable for identifying at-risk patients and improving their prognosis.

Circulating blood cell traits are approximate indicators of systemic inflammation and immune status and are commonly measured via routine blood tests and categorized into six traits: white blood cells (WBCs), basophils, eosinophils, lymphocytes, monocytes, and neutrophils. In cross-sectional clinical studies, circulating blood counts have been associated with the incidence, severity, and progression of PE. Increased neutrophil and lymphocyte counts can serve as predictors for the early development of PE in patients with COVID-2019 [14]. Total WBC counts, as well as the neutrophil/lymphocyte ratio, can aid the risk assessment of mortality in patients with acute PE [15, 16]. Elevated WBC counts are independently associated with poor clinical outcomes in patients with central PE [17]. Notably, 30-day mortality rates in patients with PE who have a WBC count < 5.0 × 10^9^/L are significant higher than in those with a WBC count of 7.9-8.2 × 10^9^/L [18]. An increase in the platelet-to-lymphocyte ratio is associated with worse in-hospital and long-term outcomes in patients with acute PE [19]. The δ-neutrophil index reflects the ratio of immature granulocytes to the total number of neutrophils in the peripheral circulation, which can be determined through certain automated blood cell analysers [20, 21]. A higher δ-neutrophil index on admission has been associated with increased short-term mortality in patients with acute PE [22]. A lower eosinophil-to-monocyte ratio can independently predict the long-term mortality in patients with intermediate-high and high risk of PE [23].

Mendelian randomization (MR) is an epidemiological method that utilizes single nucleotide polymorphisms (SNPs) of exposure factors to evaluate whether genetic variation is associated with an outcome (disease) [24]. Because alleles are randomly distributed at conception and should be robust to confounding factors and reverse causality, MR can address the limitations of confounding and reverse causation in observational studies from a genetic viewpoint. MR has already been applied to infer causative relationships between various diseases and risk factors [25–27]. Using summary statistics from GWAS, this study employed bidirectional MR analysis to evaluate whether there is a causal relationship between circulating blood cell counts and PE and further explored the role of lymphocyte subgroups in PE.

## Materials and methods

### Study design

**Fig. 1:**
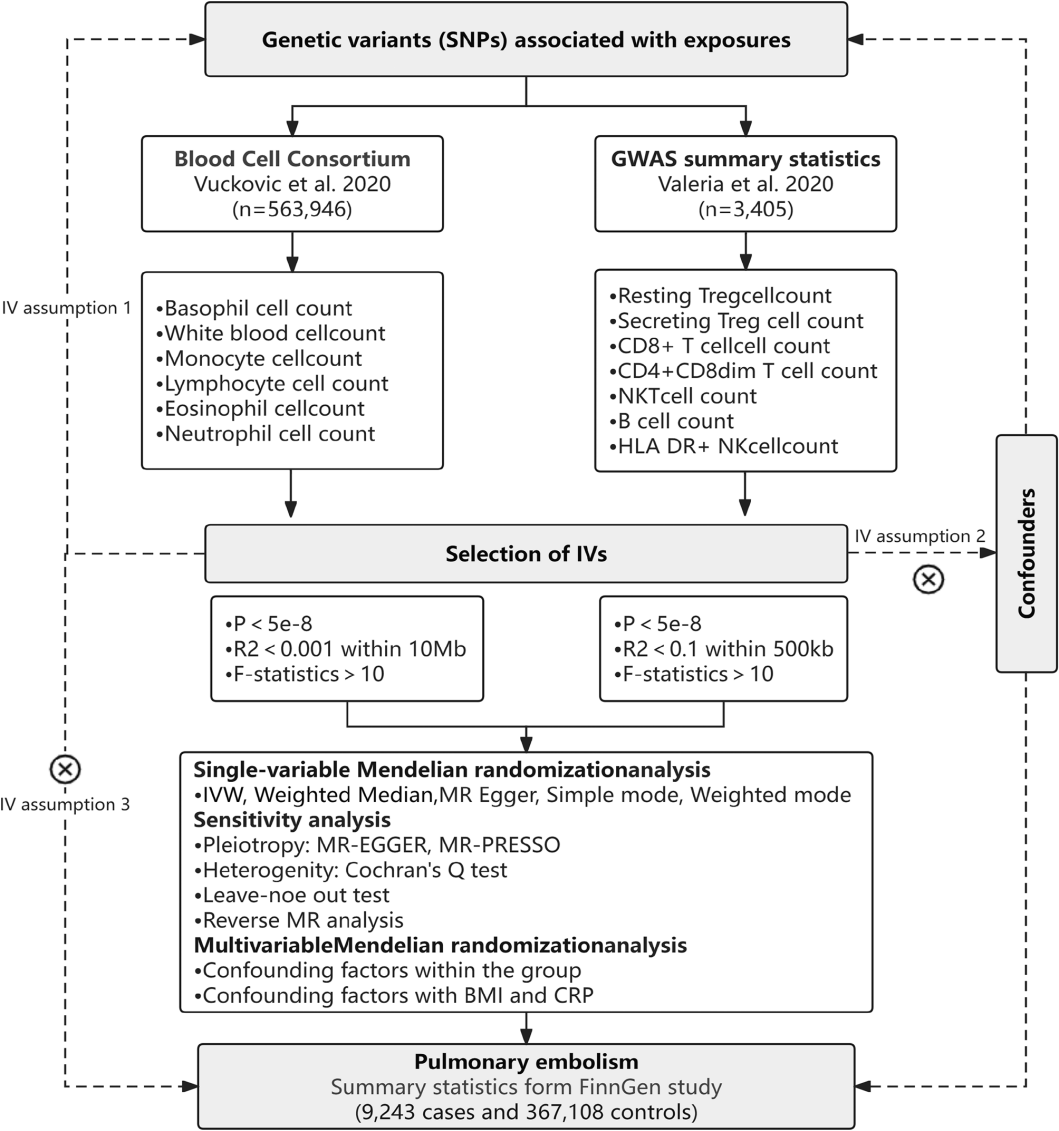
Flowchart of the overall MR design. In assumption 1, exposure is robustly correlated with instrument variables (IVs); In assumption 2, IVs are not affected by confounders; In assumption 3, IVs only affect outcome through exposure. *Abbreviation* SNPs, single-nucleotide polymorphisms; GWAS, genome-wide association study; NK cell, nature kill cell; IVs, instrumental variables; IVW, inverse variance weighted; BMI, body mass index; CRP, C-reactive protein

We conducted bidirectional two-sample MR analysis to explore the causal relationship between circulating blood cell counts (total WBC counts, eosinophil counts, basophil counts, lymphocyte counts, monocyte counts, and neutrophil counts) and PE. We then performed MVMR to determine the independent effect of these cell types on PE risk after considering the interactions between these cell types. Additionally, to adjust for potential influences of inflammation (C-reactive protein, CRP) and obesity on positive blood cell outcomes, we employed three models for MVMR analysis. We adjusted for the inflammatory effect (CRP) in Model 1, the obesity effect (body mass index, BMI) in Model 2, and both effects in Model 3. Finally, we conducted two-sample MR as well as MVMR on lymphocyte subpopulations to examine the critical role of these subpopulations in the process. The entire research process is illustrated in Fig. 1.

Three hypotheses underlie our MR study: (1) a close relationship exists between IV and blood cell characteristics; (2) IVs are independent of any confounding factors relating to blood cell characteristics and PE; and (3) IVs only affect PE through their effect on blood cell characteristics and not through any other direct route.

### Data sources

In this study, we performed MR analyses using multiple genetic variants as instrumental variables (IVs) based on summary statistics derived from the IEU Open GWAS database (https://gwas.mrcieu.ac.uk/) and the FinnGen study (https://r9.risteys.finngen.fi/). To prevent polytomy bias in cross-spectrum cases, we excluded individuals of non-European ancestry from the study. Our exposure tool relies on summary statistics from the Blood Cell Consortium large-scale GWAS on blood cell characterization, which included 563,946 participants of European descent [28]. Based on this GWAS, genetic variants associated with WBC, lymphocyte, monocyte, neutrophil, eosinophil, and basophil counts were identified. Cell subpopulation analyses, including absolute T-cell and B-cell counts, were conducted using summary GWAS statistics from 3757 individual samples analysed by flow cytometry [29]. These cells included human leukocyte antigen (HLA) DR^+^ natural killer (NK) cells, NKT cells, CD4^+^ CD8^dim^ T cells, CD8^+^ T cells, resting regulatory T (Treg) AC cells, secreting Treg AC cells and B cells. For outcome variables, we analysed the R9 release data for PE from the FinnGen study, which included 9243 PE patients and 367,108 healthy individuals. The datasets are publicly accessible, and each phenotype is described in Table 1.

**Table 1:** An overview of the GWAS datasets used in the MR analysis

Contribution	Traits	Sample size	Number of SNPs	Author	GWAS ID
Exposure	Basophil cell count	563,946	–	Vuckovic et al.	ieu-b-29
	White blood cell count	563,946	–	Vuckovic et al.	ieu-b-30
	Monocyte cell count	563,946	–	Vuckovic et al.	ieu-b-31
	Lymphocyte cell count	563,946	–	Vuckovic et al.	ieu-b-32
	Eosinophil cell count	563,946	–	Vuckovic et al.	ieu-b-33
	Neutrophil cell count	563,946	-	Vuckovic et al.	ieu-b-34
	Resting CD4 regulatory T cell count	3,405	15,131,843	Orrù et al.	ebi-a-GCST90001480
	Secreting CD4 Regulatory T cell count	3,405	15,131,843	Orrù et al.	ebi-a-GCST90001492
	CD8^+^ T cell count	3,405	15,131,843	Orrù et al.	ebi-a-GCST90001592
	CD4^+^CD8^dim^ T cell count	3,405	15,131,843	Orrù et al.	ebi-a-GCST90001609
	Nature Kill T cell count	3,405	15,131,843	Orrù et al.	ebi-a-GCST90001621
	B cell count	3,405	15,131,843	Orrù et al.	ebi-a-GCST90001642
	HLA DR^+^ Nature Kill cell count	3,405	15,131,843	Orrù et al.	ebi-a-GCST90001648
Outcome	Pulmonary embolism	376,351	16,380,466	NA	finn-b-I9_PULMEMB
Confounders	Body mass index	681,275	2,336,260	Yengo et al.	ieu-b-40
	C reactive protein	353,466	19,057,467	Sakaue et al.	ebi-a-GCST90018950

*Abbreviation* GWAS, genome-wide association study; SNPs, single nucleotide polymorphism

### IV selection

SNPs associated with circulating blood cell traits that exhibited genome-wide significant associations (*p* < 5 × 10^− 8^) with modifiable risk factors for blood cell traits were identified as IVs. The inclusion criteria of IVs in the model of confounders are consistent with circulating blood cell traits. To obtain independent IVs, we clustered them according to the linkage disequilibrium reference panel of the 1000 Genomes Project (R^2^ < 0.001 at a distance of 10,000 kb). SNPs with palindromic and ambiguous alleles were deleted. IVs with F-statistics > 10 were regarded as strong tools and reserved for the following analyses to avoid bias caused by weak tools. With respect to the relatively medium-sized GWAS on lymphocyte subpopulations, we applied a *p*-value cut-off of 5 × 10^− 8^ and a looser aggregation threshold (R^2^ < 0.1 at a distance of 500 kb) [30].

### Statistical analyses

A two-sample MR method utilizes publicly available summary-level data from multiple sources to infer causal relationships between circulating blood cell counts and PE by utilizing SNPs as IVs. For single variable MR (SVMR), we first used MR-PRESSO to identify outliers and discard them, and then used an inverse variance weighted approach (IVW) to assess the causal relationship between blood cells and PE. To assess the robustness of the IVW method, we also performed MR analysis using other four alternative methods (weighted median, MR-Egger, simple mode and weighted mode). Next, we assessed heterogeneity among IVs using the Cochran heterogeneity test; if heterogeneity was high (Q_pval < 0.05), a random effects model was used to estimate MR effect sizes, and a fixed effects model was used if heterogeneity among IVs was low (Q_pval > 0.05). The MR-Egger test was used to detect multiple effects in the calibration of horizontal genes. MR‒Egger intercepts were used to determine horizontal pleiotropy in the horizontal direction. The false discovery rate (FDR) method was used to interpret multiple comparisons and adjust for statistical significance in the study. Causal effects are considered significant if the IVW *p*-value, after FDR correction, is below the 0.05 threshold, and both the weighted median and MR‒Egger results are directionally consistent.

In multivariate MR (MVMR), we performed MV-weighted linear regressions (variable-uncorrelated and random-effects models) using an extension of the IVW MR method, with the intercept set to zero. Multivariate effects were corrected using an extension of the MR-Egger method. First, considering the interplay between different types of peripheral blood cells, MVMR analysis was applied to individually determine their effects on the risk of PE, considering adjust *p-*values less than 0.05 as indicating statistical significance. Next, exposures with a significant causal effect were subjected to MVMR analyses using the MVMR-IVW method with three models adjusted for potential confounders, with *p*-values less than 0.05 indicating statistical significance. As we hypothesized that inflammation and obesity might be confounders of the exposure-outcome relationship, we selected the CRP level and BMI as measures. Finally, we conducted MVMR analysis to independently assess the influence of lymphocyte subgroups on PE risk, with *p*-values less than 0.05 indicating statistically significant. Table 1 provides details on these publicly available data.

Causal estimation was conducted using the “TwoSampleMR” package, and outliers were detected using the “MR-PRESSO” package. MVMR analysis was performed using the “MVMR” and “Mendelian Randomization” packages. MR estimates are expressed as odds ratios (ORs) and 95% confidence intervals (CIs). We used R software version 4.3.0 for all statistical analyses (See Fig. 1).

## Results

### SVMR predicts the effect of the circulating blood cell counts on PE

As a result of the selection and harmonization of IVs, we included 186 SNPs for basophil counts, 450 SNPs for WBCs, 465 SNPs for monocyte counts, 466 SNPs for lymphocyte counts, 424 SNPs for eosinophil counts, and 390 SNPs for neutrophil counts for MR analysis. The F-statistic for all SNPs was greater than 10, indicating their potential as powerful diagnostic tools (see Supplementary Tables 1–7).

**Fig. 2:**
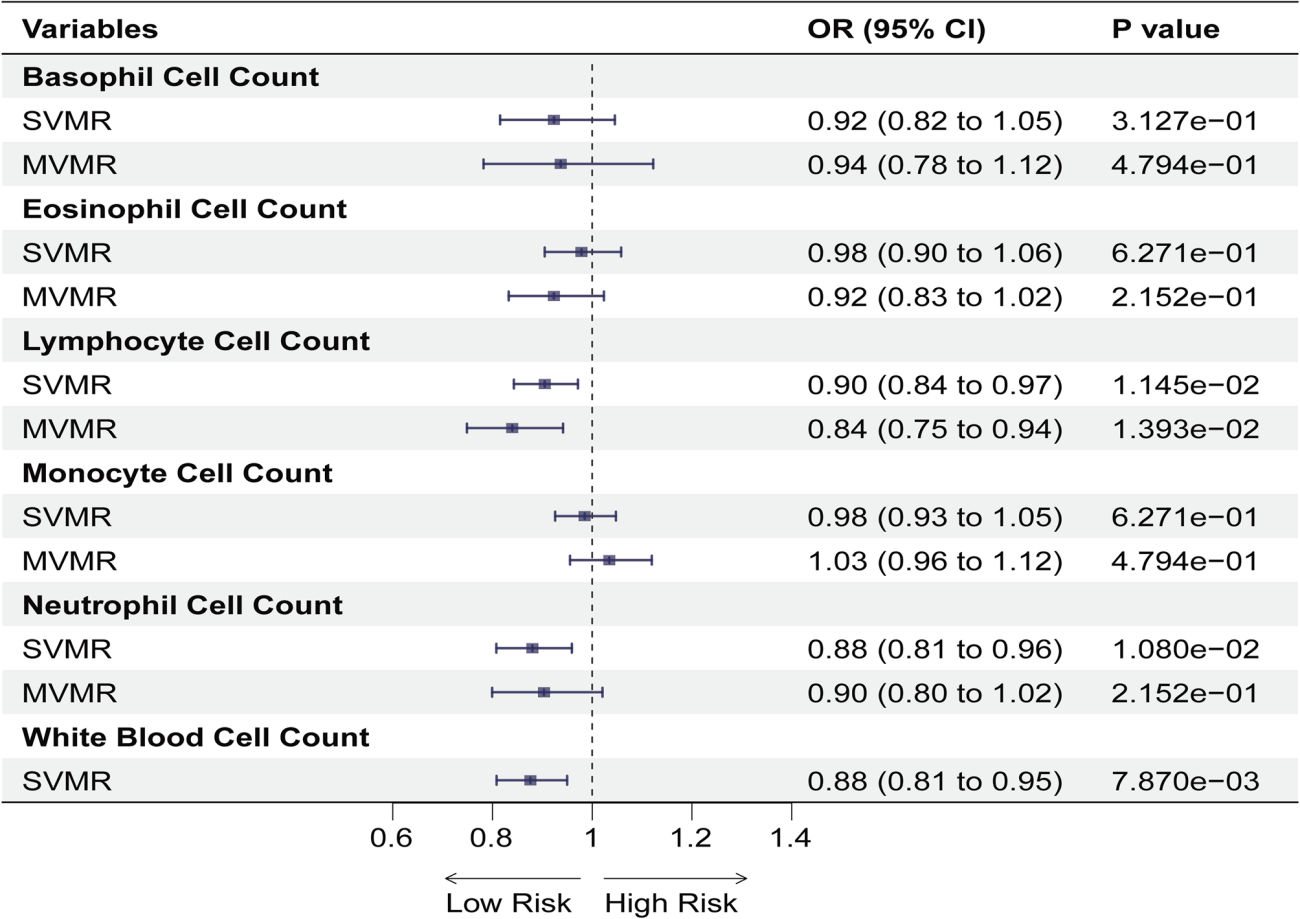
Forest plot for the causal effect of circulating blood cell counts on the risk of pulmonary embolism derived from IVW method. *Abbreviation* OR, odds ratio; CI, confidence interval; IVW, inverse variance weighted; SVMR, single-variable mendelian randomization; MVMR, multivariate mendelian randomization

The causal effect of the circulating blood cell counts on PE susceptibility is summarized in Fig. 2. In particular, our findings indicate that increased susceptibility to PE was strongly associated with reduced total WBC counts (OR: 0.88, 95% CI: 0.81–0.95, *p* = 0.0079), lymphocyte counts (OR: 0.90, 95% CI: 0.84–0.97, *p* = 0.0115) and neutrophil counts (OR: 0.88, 95% CI: 0.81–0.96, *p* = 0.0108). The results remained significant even after FDR correction. However, no significant associations were observed between the eosinophil, basophil, or monocyte counts and PE susceptibility.

### Sensitivity analysis

To determine the stability and reliability of the results, we performed sensitivity analyses. In the SVMR analysis, we used MR‒Egger’s method, weighted median (weighted median), Cochran’s Q test, and the MR-PRESSO model to verify the robustness of the IVW results (see Supplementary Table 8). The potential heterogeneity of IV effects was assessed using heterogeneity tests. We applied a random effects model when heterogeneity was detected and a fixed effects model when no heterogeneity was detected. The Cochran’s Q test indicated heterogeneity in all peripheral blood cells, thus, we used random effects IVW for the MR analysis. The MR-PRESSO model removed outliers, and an MR‒Egger intercept test revealed no horizontal pleiotropy. A leave-one-out analysis confirmed the robustness of our MR results by testing the effect of each SNP individually (see Supplementary Table 9).

### Reverse MR

**Fig. 3:**
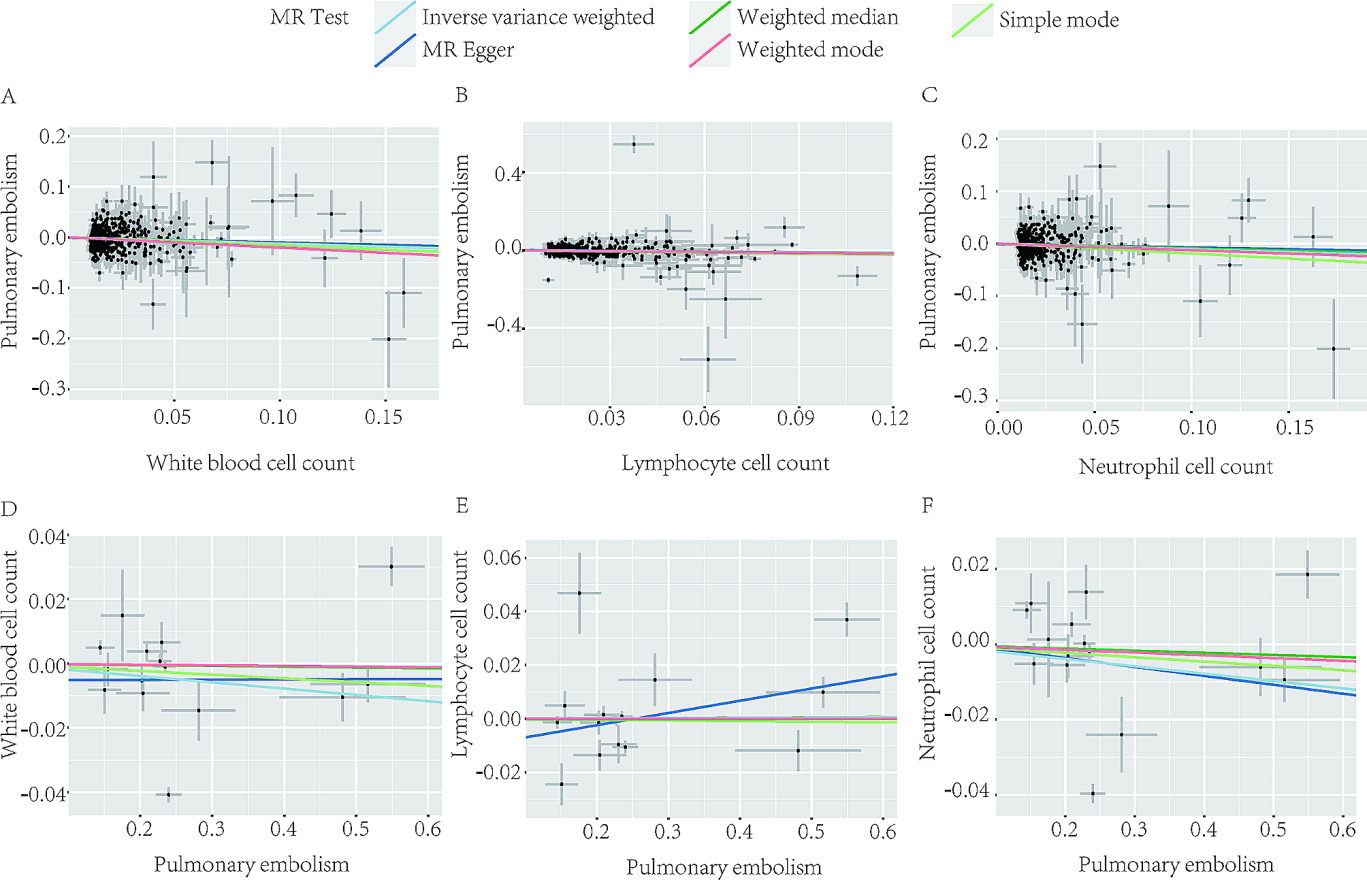
The scatter plots of bidirectional MR analysis results between circulating white blood cell traits and PE. The scatter plots of genetic associations between white blood cell counts **(A)**, lymphocyte cell counts **(B)** and neutrophil cell counts **(C)** associated SNPs against the genetic associations of PE. The scatter plots of genetic associations between PE associated SNPs against the genetic associations of white blood cell count **(D)**, lymphocyte cell count **(E)** and neutrophil cell count **(F)**. The sky-blue, dark-blue, bottle-green, red and light-green line represents the IVW, MR-Egger, weighted median, weighted mode and simple mode effect, respectively. The slope of the line represents the mendelian randomization effect size. *Abbreviation* PE, pulmonary embolism; MR, mendelian randomization; IVW, inverse variance weighted

We performed a reverse MR analysis based on the same screening criteria to detect potential reverse causality. The results indicated that there was no reverse causality between the total WBC counts (P_ivw_= 0.143; Fig. 3D), neutrophil counts (P_ivw_= 0.992; Fig. 3E), or lymphocyte counts (P_ivw_= 0.138; Fig. 3F) and the risk of PE. The MR‒Egger intercept test revealed no evidence of horizontal pleiotropy (0.289, 0.554, and 0.233, respectively) (see Supplementary Table 10). Reverse Mendelian randomization further confirmed the stability of the results obtained through SVMR.

### MVMR predicts the effect of the circulating blood cell counts on PE

Considering the potential interaction between the various cell types, we performed MVMR to independently assess their impact on PE risk (Fig. 2). MVMR revealed a strong association between higher susceptibility to PE and decreased circulating lymphocyte counts (OR: 0.84, 95% CI: 0.75–0.94, *p* = 0.0139) (Supplementary Table 11). There was no significant causal association between neutrophil counts and PE (OR: 0.90, 95% CI: 0.80-1.00, *p* = 0.2152), suggesting that neutrophils and other cells may be confounding elements (see Supplementary Table 11). To validate the IVW results, the MVMR Egger method was applied, and no horizontal pleiotropy was observed, indicating the validity of the results. The Weighted median exhibited a similar trend. The results are presented in Fig. 3 and Supplementary Table 11.

**Fig. 4:**
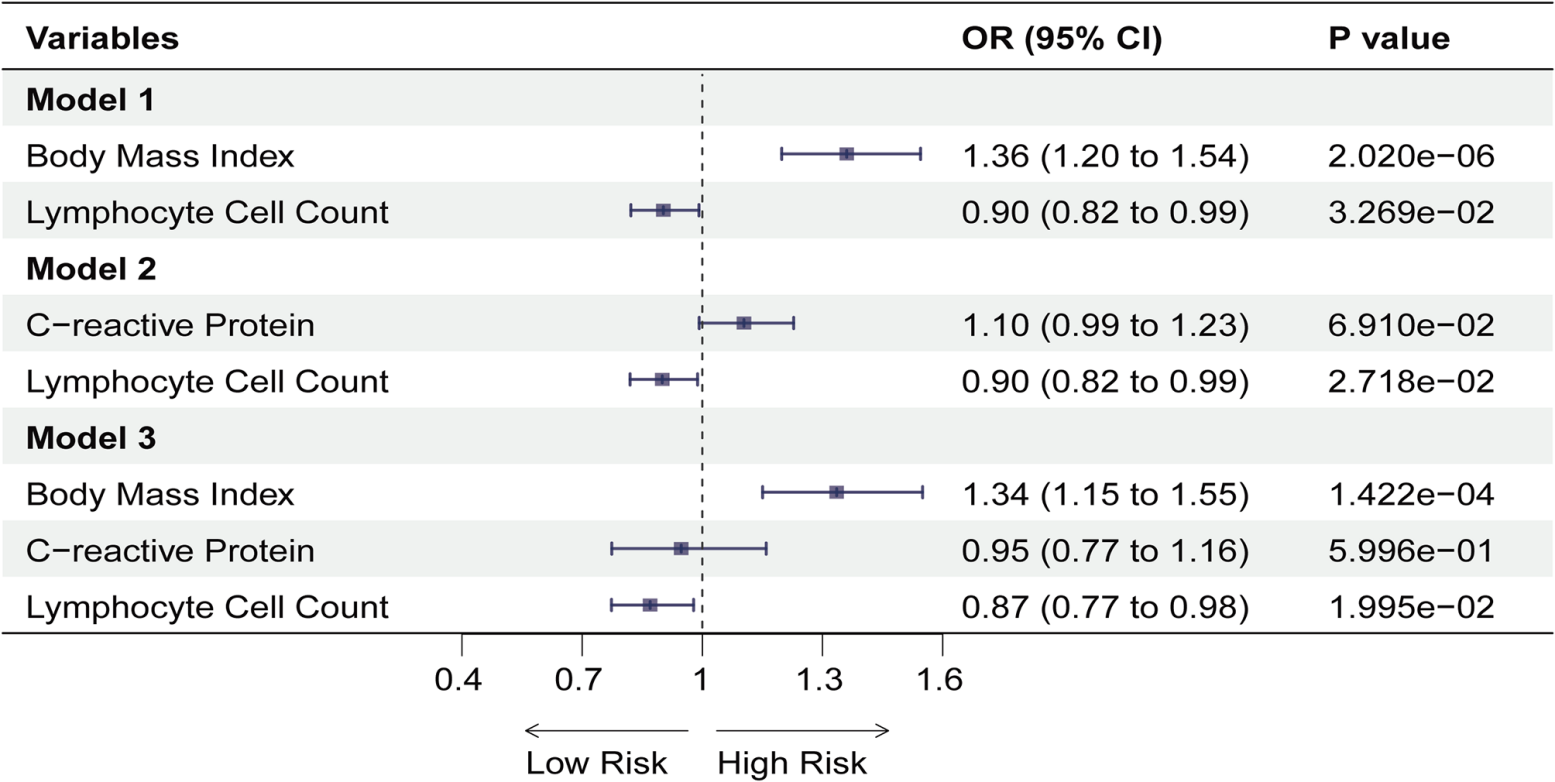
Forest plot for the causal effect of lymphocyte cell counts on the risk of PE derived from IVW with three models. *Abbreviation* PE, pulmonary embolism; IVW, inverse variance weighted; OR, odds ratio; CI, confidence interval; SVMR, single-variable mendelian randomization; MVMR, multivariate mendelian randomization

To further investigate the direct association between lymphocyte counts and PE, we included inflammation and obesity as potential confounders in the MVMR. According to the results, even after adjusting for the effect of obesity (BMI), lymphocyte counts (OR: 0.90, 95% CI = 0.82–0.99, *p* = 0.0327) had a potentially direct effect on PE in Model 1. There remained a potential direct effect on PE (OR: 0.90, 95% CI: 0.82–0.99, *p* = 0.0272) in Model 2 after adjusting only for inflammation (CRP). It is important to note that the lymphocyte counts (OR: 0.87, 95% CI: 0.77–0.98, *p* = 0.0199) continued to exhibit a potential direct effect on PE in Model 3, which was also adjusted for inflammation (CRP) and obesity (BMI). A similar trend was observed with both the MVMR Egger method and the mean value method. Figure 4 and Supplementary Table 12 illustrate our results.

### MR predicts circulating lymphocyte subtypes in PE patients

**Fig. 5:**
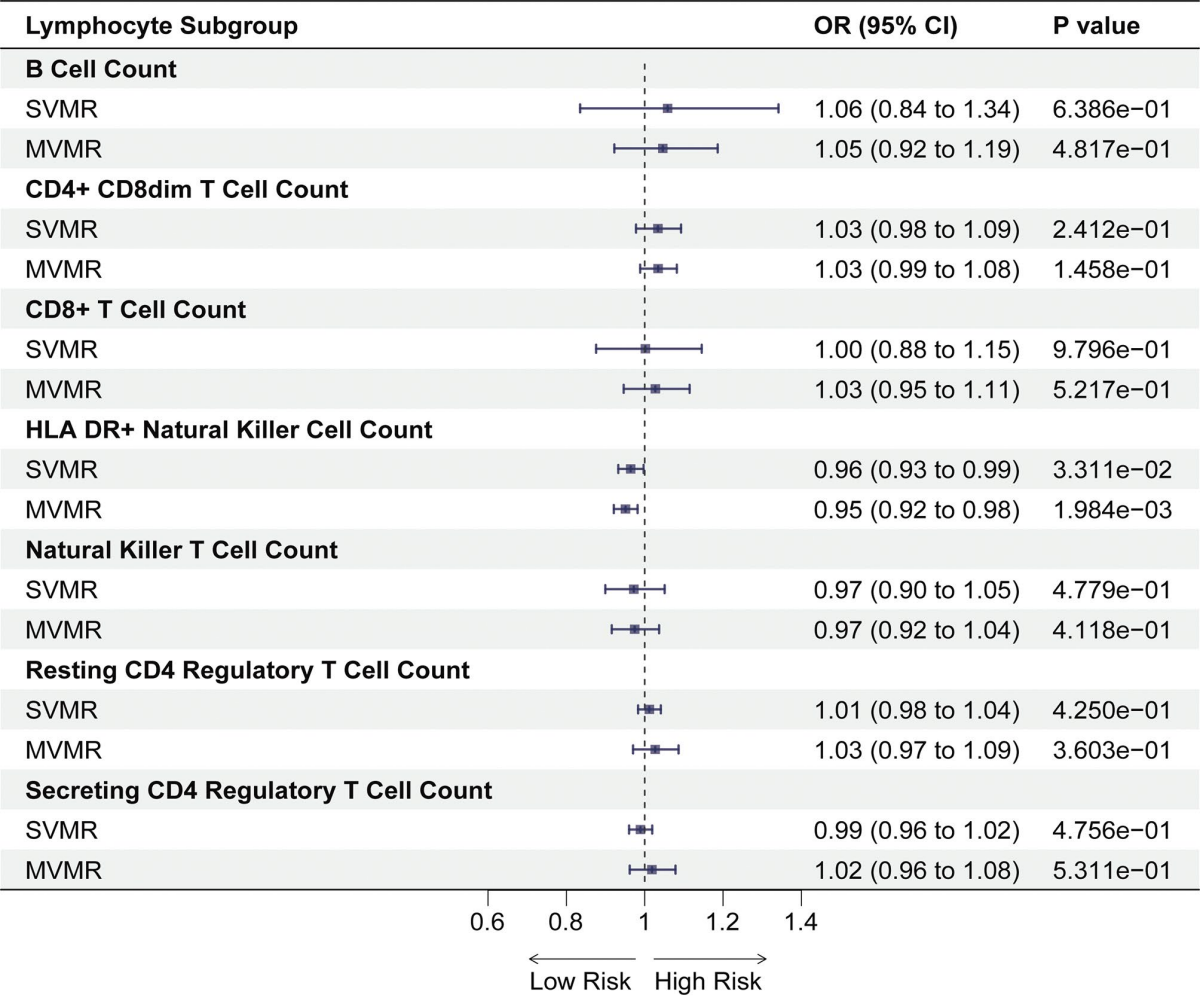
Forest plot for the causal effect of circulating lymphocyte subgroup on the risk of PE in SVMR and MVMR. *Abbreviation* PE, pulmonary embolism; OR, odds ratio; CI, confidence interval; SVMR, single-variable mendelian randomization; MVMR, multivariate mendelian randomization

To further investigate the causal relationship between certain lymphocyte subtypes and PE risk, we performed both SVMR and MVMR analyses on PE and absolute counts of lymphocyte subpopulations, including HLA-DR^+^ NK cells, NKT cells, CD4^+^ CD8^dim^ T cells, CD8^+^ T cells, resting Treg AC cells, secreting Treg AC, and B cells. Our results are summarized in Fig. 5 and Supplementary Tables 13, and SNP selection is listed in Supplementary Table 14.

The results suggested that low levels of HLA-DR^+^ NK cell counts (OR: 0.96, 95% CI: 0.93–0.99, *p* = 0.0331) in the SVMR were associated with a greater risk of PE. Cochran’s Q test indicated heterogeneity in CD8^+^ T cell counts across lymphocyte subpopulations, therefore, we applied an IVW method based on random effects models. The random effects model IVW method was used for the remaining lymphocyte subpopulations. The MR-PROSS test did not reveal any outliers, and neither did the MR-Egger intercept test detect horizontal pleiotropy (Supplementary Table 15). Leave-one-out results indicate that several specific SNPs may affect HLA-DR^+^ NK cell counts (see Supplementary Table 16). MVMR also identified a greater PE risk for patients with low levels of HLA-DR^+^ NK cells (OR: 0.95, 95% CI: 0.92–0.98, *p* = 0.019), and the adjusted *p*-value remained significant (Fig. 5 and Supplementary Table 17). The MVMR Egger method and the mean value method also exhibited similar trends (see Supplementary Table 17). There was no significant association between PE risk and absolute cell counts.

## Discussion

In this study, we conducted SVMR and MVMR analyses to determine whether there is a causal relationship between the circulating blood cell counts and PE from a genetic perspective. SVMR and reverse MR analysis revealed a negative causal relationship between lymphocyte and neutrophil counts and PE. Multiple sensitivity tests were conducted to evaluate the robustness of the results and minimize the possibility of pleiotropic bias. The MVMR results suggest a stronger causal relationship between lymphocytes and PE after correcting for interactions between different leukocytes, whereas the results for neutrophils were not statistically significant. Further MR analysis targeting lymphocyte subpopulations demonstrated that the risk of PE was increased in patients with low HLA-DR^+^ NK cell counts.

It is possible that peripheral blood cells are involved in the development of PE, likely by influencing the vascular endothelium and coagulation-fibrinolytic pathways involved in the disease process. There is a higher concentration of neutrophils in peripheral blood, and thrombosis has been linked to neutrophils in certain studies. According to Peng et al. [31], high neutrophil counts on admission were associated with intermediate- and high-risk acute PE and could serve as indicators of both inflammation and thrombosis associated with acute PE. The localized recruitment of neutrophils in the setting of DVT can contribute to the formation of thrombi in situ within the pulmonary vascular system [32]. On the one hand, neutrophils play a role in the formation of thrombi by expressing tissue factor and protein disulfide bond isomerase; on the other hand, highly activated neutrophils are capable of releasing neutrophil extracellular traps (NETs) into extracellular compartments where NETs act as scaffolds to support platelets and erythrocytes and interact with fibrin fibres to enhance thrombus stability, while histones H3 and H4, which are associated with NETs, directly contribute to platelet aggregation [33, 34]. NETs formation is recognized as a key factor in the development and progression of thromboembolic disease [35, 36]. In practice, PE is generally associated with the obstruction of pulmonary arteries and their branches following the dislodgment of emboli from peripheral circulation. Whether these prothrombotic and increased thrombotic properties of neutrophils and NETs are related to an increased risk of PE remains to be determined. We found a negative genetic correlation between neutrophils and PE in SVMR analysis, although this causal relationship was no longer significant after adjustment for MVMR analysis. In addition, some studies have examined the association between PE and other peripheral blood cells. By degranulating and releasing cytotoxic cations while activating the endogenous coagulation pathway, eosinophils can damage the vascular endothelium, and can also activate the exogenous coagulation pathway by activating tissue factors, platelet activating factors, and other factors, resulting in thrombosis [37–39]. Basophils can adhere to endothelial cells and may mediate endothelial injury that results in thrombosis [40]. However, no genetic causal relationships were found between the eosinophil count or basophil count and PE in our study.

Lymphocytes are important components of the immune system and are associated with a greater risk of contracting PE. Lymphocyte counts are used in the assessment of the incidence and prognosis of PE. A recent study conducted by Elshahaat et al. [41] found that individuals with lower lymphocyte counts were at greater risk for PE. Both the neutrophil-to-lymphocyte ratio and platelet-to-lymphocyte ratio predict all-cause mortality in acute PE patients [42, 43]. The mechanism by which lymphocytes play a role in thrombosis has been explored in several studies. Interferon gamma and interleukin 17 produced by activated T lymphocytes promote endothelial dysfunction by inducing oxidative stress injury [44]. Regulatory T cells regulate the activation and integrity of the vascular endothelium and reduce the transcellular migration of leukocytes [45]. Inactivation of CD1d-dependent NKT cells increases endothelial cell apoptosis and induces microvascular damage [46]. Activated mouse NKT cells have been shown to damage renal vascular endothelial cells through a mechanism mediated by perforin [47]. There is evidence that Treg cells have some endothelial protective effects, diminishing the extent of vascular endothelial damage and preventing hypertension and pulmonary hypertension [48–50]. A possible role for blood lymphocytes in the development of pulmonary embolism has been suggested in these studies. We observed a negative genetic correlation between lymphocytes and PE in both our SVMR and MVMR analyses, and further MR analyses targeting lymphocyte subpopulations indicated that this correlation was strongly correlated with HLA-DR^+^ NK cells. A class of lymphocytes known as HLA-DR^+^ NK cells combines the phenotypic features of NK cells and dendritic cells and plays an important role in the immune response. The function of HLA-DR^+^ NK cells is demonstrated as follows: First, they are capable of producing proinflammatory cytokines, degranulating, and proliferating when stimulated; furthermore, HLA-DR^+^ NK cells can take up antigens (e.g., tetanus toxin) and present them to T cells, inducing T cell activation and proliferation and providing costimulatory signals for central memory T-cell differentiation [51]. In addition to being present in the blood and tissues of healthy mice and humans, these cells have been found to be present in a range of pathological conditions. Conditions associated with chronic inflammation, such as human immunodeficiency virus-associated immunodeficiency, multiple sclerosis, and IgA nephropathy, may significantly increase the number of HLA-DR^+^ NK cells in the peripheral blood. The expression of HLA-DR in NK cells is also increased in response to exogenous stimuli. However, no relevant studies have investigated the role of HLA-DR^+^ NK cells in PE or the association between HLA-DR^+^ NK cells and thrombosis. Considering the importance of lymphocytes in the coagulation cascade and pathophysiology of vascular vessels, it is worthwhile to investigate the potential mechanisms underlying how HLA-DR^+^ NK cells contribute to PE development.

Circulating blood cell counts may hold significant clinical value in the risk assessment and prognosis prediction for patients with PE. Routine blood examinations are more convenient and accessible than other blood tests. Our study suggested a causative relationship between a decrease in lymphocyte counts and the development of PE. Therefore, in clinical practice, vigilant monitoring for potential PEs should be implemented in patients presenting with lymphocyte reduction, possibly as an adjunct to screening based on the Canadian Wells score or the revised Geneva score. Further measurement of HLA-DR^+^ NK cells count in these patients may yield additional information. However, further multicenter trials are needed to validate these findings. Additionally, integrating blood cell counts with markers such as D-dimer and cardiac troponins into a comprehensive risk assessment model, especially for critically ill patients, could refine PE patient stratification and foster personalized treatments. This comprehensive approach may enhance the early diagnosis of PE and improve outcomes.

### Limitations and prospects

The strength of this study is the use of a bidirectional MR approach simultaneously to infer causal relationships between circulating blood cell and immune cell characteristics and PE, which minimizes the bias associated with reverse causality and confounding factors in observational studies. MVMR analysis eliminated potential confounders due to inflammation and obesity, as well as interactions among blood cells. By using a variety of methods, including IVW, weighted median, MR‒Egger, simple mode and weighted mode methods, we were also able to verify that their causal estimates were consistent, and their mutual corroboration improved the argument for causal effects. Additionally, the majority of the study population was of European ancestry, therefore avoiding any bias associated with stratification of the population.

It must be noted, however, that this study has several limitations: First, despite using the largest known summary statistics from the GWAS to date, we were not able to conduct subgroup analyses due to the lack of raw data and subgroup summary statistics, which may have resulted in insufficient statistical power; Second, as all our datasets came from European populations, the results must be validated in other ethnic groups. Finally, MR analyses did not reveal a genetic association between PE and basophils and eosinophils. This does not mean that these exposures do not contribute to the development of PE. Further clinical and basic research is required to determine whether these cells play a role in the development of PE.

## Conclusion

In conclusion, this MR study demonstrated that genetically predicted higher PE risk was causally associated with lower lymphocyte counts. Subsequent analysis focusing on lymphocyte subsets indicated that HLA-DR^+^ NK cells may play a key role in this association. Nevertheless, the risk of PE does not appear to be associated with other leukocyte types, such as neutrophils, basophils, eosinophils, or monocytes. Additional research is required to investigate the role of lymphocytes, especially HLA-DR^+^ NK cells, in the pathogenesis of PE.

### Electronic supplementary material

Below is the link to the electronic supplementary material.

Supplementary Material 1

## Data Availability

Open access is provided to the data in a public repository. Data URLs: GWAS summary statistics for pulmonary embolism were downloaded from the following website: https://pgc.unc.edu/for-researchers/download-results/. GWAS summary statistics for circulating white blood cell counts were downloaded from the BCX Consortium: http://www.mhi-humangenetics.org/en/resources/. GWAS summary statistics for circulating lymphocyte subtypes traits could be download form open GWAS Catalog: https://gwas.mrcieu.ac.uk/. All codes used in the research are available, and the original contributions presented in the study are included in the article/Supplementary Material. Further inquiries can be directed to the corresponding author.

## Abbreviations

BMI: Body mass index
CI: Confidence interval
CRP: C-reactive protein
DVT: Deep vein thrombosis
FDR: False discovery rate
GWAS: Genome-wide association studies
HLA: Human leukocyte antigen
IVs: Instrumental variables
IVW: Inverse variance weighted approach
MR: Mendelian randomization
MVMR: Multivariate mendelian randomization
NETs: Neutrophil extracellular traps
NK: Natural killer
OR: Odds ratio
PE: Pulmonary embolism
SNPs: Single nucleotide polymorphisms
SVMR: Single variable mendelian randomization
VTE: Venous thromboembolism
WBC: White blood cell
Treg: Regulatory T
