# Supplementary material for: Causal association between circulating blood cell traits and pulmonary embolism: a mendelian randomization study
Source: Thromb J. 2024 Jun 11;22:49. doi: 10.1186/s12959-024-00618-3 (PMC11167760; doi:10.1186/s12959-024-00618-3)
Supplement: Supplementary file 1 — Supplementary Material 1 [file 12959_2024_618_MOESM1_ESM.pdf]

Supplementary Table 1: SVMR analysis between circulating white blood cell count and PE
Supplementary Table 2: Description of SNPs for basophil cell counts incorporated after MR-presso deletion
Supplementary Table 3: Description of SNPs for eosinophil cell counts incorporated after MR-presso deletion
Supplementary Table 4: Description of SNPs for lymphocyte cell counts incorporated after MR-presso deletion
Supplementary Table 5: Description of SNPs for monocyte cell counts incorporated after MR-presso deletion
Supplementary Table 6: Description of SNPs for neutrophil cell counts incorporated after MR-presso deletion
Supplementary Table 7: Description of SNPs for WBC cell counts incorporated after MR-presso deletion
Supplementary Table 8: Sensitivity analyses for the SVMR analysis of circulating white blood cell and PE
Supplementary Table 9: Leave-one-out analysis results for SVMR analysis of circulating white blood cell and PE
Supplementary Table 10: Reverse MR results of peripheral blood WBC, neutrophils and lymphocytes with PE
Supplementary Table 11: MVMR results between circulating white blood cell count and PE
Supplementary Table 12: MVMR results between circulating white blood cell count and PE adjusting for confounders BMI and CRP
Supplementary Table 13: SVMR analysis between lymphocyte subtype count and PE
Supplementary Table 14: Description of SNPs for lymphocyte subtype cell counts incorporated after MR-presso deletion
Supplementary Table 15: Sensitivity analyses for the SVMR analysis of lymphocyte subtype cell counts and PE
Supplementary Table 16: Leave-one-out analysis results for SVMR analysis of lymphocyte subtype cell counts and PE
Supplementary Table 17: MVMR results between lymphocyte subtype count and PE

Supplementary Table 1. SVMR analysis between circulating white blood cell count and PE

Blood Cell	outcome	exposure	method	nsnp	b	se	pval
basophil	pulmonary embolism	basophil cell count || id:ieu-b-29	MR Egger	186	-0.1509	0.11624	0.195835
basophil	pulmonary embolism	basophil cell count || id:ieu-b-29	Weighted median	186	-0.18636	0.093154	0.045436
basophil	pulmonary embolism	basophil cell count || id:ieu-b-29	Inverse variance weighted	186	-0.0798	0.063439	0.208448
basophil	pulmonary embolism	basophil cell count || id:ieu-b-29	Simple mode	186	0.164825	0.22598	0.466694
basophil	pulmonary embolism	basophil cell count || id:ieu-b-29	Weighted mode	186	-0.2885	0.112593	0.011195
White blood cell	pulmonary embolism	white blood cell count || id:ieu-b-30	MR Egger	450	-0.11989	0.085256	0.160346
White blood cell	pulmonary embolism	white blood cell count || id:ieu-b-30	Weighted median	450	-0.12726	0.061512	0.03856
White blood cell	pulmonary embolism	white blood cell count || id:ieu-b-30	Inverse variance weighted	450	-0.13227	0.041161	0.001312
White blood cell	pulmonary embolism	white blood cell count || id:ieu-b-30	Simple mode	450	-0.15967	0.175055	0.362211
White blood cell	pulmonary embolism	white blood cell count || id:ieu-b-30	Weighted mode	450	-0.19464	0.107405	0.070628
monocyte	pulmonary embolism	monocyte cell count || id:ieu-b-31	MR Egger	465	-0.0878	0.051074	0.086277
monocyte	pulmonary embolism	monocyte cell count || id:ieu-b-31	Weighted median	465	-0.13123	0.053225	0.013682
monocyte	pulmonary embolism	monocyte cell count || id:ieu-b-31	Inverse variance weighted	465	-0.01531	0.031522	0.627113
monocyte	pulmonary embolism	monocyte cell count || id:ieu-b-31	Simple mode	465	-0.05422	0.121026	0.65438
monocyte	pulmonary embolism	monocyte cell count || id:ieu-b-31	Weighted mode	465	-0.14654	0.060493	0.015796
lymphocyte	pulmonary embolism	lymphocyte cell count || id:ieu-b-32	MR Egger	466	-0.21824	0.073774	0.003252
lymphocyte	pulmonary embolism	lymphocyte cell count || id:ieu-b-32	Weighted median	466	-0.12698	0.060696	0.03643
lymphocyte	pulmonary embolism	lymphocyte cell count || id:ieu-b-32	Inverse variance weighted	466	-0.0999	0.036157	0.005726
lymphocyte	pulmonary embolism	lymphocyte cell count || id:ieu-b-32	Simple mode	466	-0.21766	0.154006	0.158222
lymphocyte	pulmonary embolism	lymphocyte cell count || id:ieu-b-32	Weighted mode	466	-0.16129	0.116182	0.165712
eosinophil	pulmonary embolism	eosinophil cell count || id:ieu-b-33	MR Egger	424	0.022059	0.078004	0.777478
eosinophil	pulmonary embolism	eosinophil cell count || id:ieu-b-33	Weighted median	424	-0.01169	0.061414	0.849067
eosinophil	pulmonary embolism	eosinophil cell count || id:ieu-b-33	Inverse variance weighted	424	-0.02173	0.039926	0.58633
eosinophil	pulmonary embolism	eosinophil cell count || id:ieu-b-33	Simple mode	424	0.224892	0.166864	0.178458
eosinophil	pulmonary embolism	eosinophil cell count || id:ieu-b-33	Weighted mode	424	-0.02907	0.089698	0.74606
neutrophil	pulmonary embolism	neutrophil cell count || id:ieu-b-34	MR Egger	390	-0.07753	0.090332	0.391273
neutrophil	pulmonary embolism	neutrophil cell count || id:ieu-b-34	Weighted median	390	-0.09111	0.070281	0.19486
neutrophil	pulmonary embolism	neutrophil cell count || id:ieu-b-34	Inverse variance weighted	390	-0.12761	0.043832	0.0036
neutrophil	pulmonary embolism	neutrophil cell count || id:ieu-b-34	Simple mode	390	-0.18869	0.170837	0.270058
neutrophil	pulmonary embolism	neutrophil cell count || id:ieu-b-34	Weighted mode	390	-0.12507	0.097665	0.201093

Supplementary Table 2: Description of SNPs for basophil cell counts incorporated after MR-presso deletion

SNP	effect_al	other_all	effect_al	other_all	beta.expc	beta.outc	eaf.expos	eaf.outcc	remove	palindrom	ambiguous	id.outcom	pval.outc	se.outcom	outcome	mr_keep.c	pval_orig	data_sour	chr.expos	pos.expos	se.expos	pval.expc	samplesiz	exposure	mr_keep.e	pval_orig	data_sour	R2	F	action	mr_keep
rs1000683	C	T	C	T	-0.01561	-0.02595	0.209061	0.211082	FALSE	FALSE	FALSE	I9_PULMEM	0.158895	0.018419	pulmonary	TRUE	reported	textfile	4	9953097	0.002521	6.22E-10	473997	basophil	TRUE	reported	igd	8.08E-05	38.3209	2	TRUE
rs1073412	A	G	A	G	-0.02543	-0.00106	0.845295	0.831569	FALSE	FALSE	FALSE	I9_PULMEM	0.957779	0.020105	pulmonary	TRUE	reported	textfile	11	89656239	0.002837	3.28E-19	471519	basophil	TRUE	reported	igd	0.00017	80.36011	2	TRUE
rs1074614	G	A	G	A	-0.0224	0.070967	0.926163	0.880316	FALSE	FALSE	FALSE	I9_PULMEM	0.002616	0.02358	pulmonary	TRUE	reported	textfile	12	80316758	0.003927	1.20E-08	472142	basophil	TRUE	reported	igd	6.89E-05	32.53371	2	TRUE
rs1080623	T	A	T	A	-0.01474	0.018005	0.597775	0.610665	FALSE	TRUE	FALSE	I9_PULMEM	0.241519	0.015373	pulmonary	TRUE	reported	textfile	6	82679060	0.002097	2.17E-12	473999	basophil	TRUE	reported	igd	0.000104	49.40118	2	TRUE
rs1083533	G	A	G	A	0.014555	-0.00265	0.349229	0.391375	FALSE	FALSE	FALSE	I9_PULMEM	0.863462	0.01539	pulmonary	TRUE	reported	textfile	11	3957766	0.002161	1.68E-11	467933	basophil	TRUE	reported	igd	9.69E-05	45.36418	2	TRUE
rs1084465	T	C	T	C	0.012373	0.00621	0.339731	0.35556	FALSE	FALSE	FALSE	I9_PULMEM	0.692901	0.015724	pulmonary	TRUE	reported	textfile	12	9893213	0.002168	1.18E-08	473997	basophil	TRUE	reported	igd	6.87E-05	32.57089	2	TRUE
rs1086893	C	T	C	T	0.029865	0.012573	0.343174	0.216743	FALSE	FALSE	FALSE	I9_PULMEM	0.49	0.018213	pulmonary	TRUE	reported	textfile	1	2.13E+08	0.002162	2.41E-43	473056	basophil	TRUE	reported	igd	0.000403	190.8147	2	TRUE
rs1088335	G	A	G	A	-0.01313	0.001542	0.284616	0.392042	FALSE	FALSE	FALSE	I9_PULMEM	0.920086	0.01537	pulmonary	TRUE	reported	textfile	10	1.01E+08	0.002268	7.30E-09	473062	basophil	TRUE	reported	igd	7.08E-05	33.51518	2	TRUE
rs1090637	G	A	G	A	-0.01249	0.002933	0.300993	0.498592	FALSE	FALSE	FALSE	I9_PULMEM	0.845797	0.01508	pulmonary	TRUE	reported	textfile	10	13498371	0.002234	2.29E-08	474000	basophil	TRUE	reported	igd	6.60E-05	31.26769	2	TRUE
rs1092707	C	T	C	T	0.079216	0.02994	0.892265	0.914271	FALSE	FALSE	FALSE	I9_PULMEM	0.264242	0.026818	pulmonary	TRUE	reported	textfile	1	2.36E+08	0.0033	########	474001	basophil	TRUE	reported	igd	0.001214	576.2303	2	TRUE
rs1106488	A	G	A	G	-0.03227	0.004671	0.073509	0.086328	FALSE	FALSE	FALSE	I9_PULMEM	0.861944	0.026858	pulmonary	TRUE	reported	textfile	12	1.2E+08	0.003923	2.05E-16	473998	basophil	TRUE	reported	igd	0.000143	67.6559	2	TRUE
rs1109778	T	C	T	C	-0.0145	-0.03166	0.402632	0.417235	FALSE	FALSE	FALSE	I9_PULMEM	0.037545	0.015225	pulmonary	TRUE	reported	textfile	4	1.03E+08	0.002087	3.83E-12	473996	basophil	TRUE	reported	igd	0.000102	48.27799	2	TRUE
rs1112124	G	T	G	T	0.012743	0.006897	0.502219	0.515983	FALSE	FALSE	FALSE	I9_PULMEM	0.645315	0.014985	pulmonary	TRUE	reported	textfile	1	8917102	0.002043	4.59E-10	473997	basophil	TRUE	reported	igd	8.21E-05	38.90494	2	TRUE
rs1115815	C	G	C	G	0.028179	0.026685	0.769788	0.785286	FALSE	TRUE	FALSE	I9_PULMEM	0.147558	0.018426	pulmonary	TRUE	reported	textfile	14	57857162	0.002564	4.59E-28	472914	basophil	TRUE	reported	igd	0.000255	120.7851	2	TRUE
rs1118796	A	G	A	G	-0.01677	-0.00518	0.130137	0.080204	FALSE	FALSE	FALSE	I9_PULMEM	0.851637	0.02768	pulmonary	TRUE	reported	textfile	10	96231169	0.003058	4.24E-08	472601	basophil	TRUE	reported	igd	6.36E-05	30.07746	2	TRUE
rs1123523	G	C	G	C	-0.01392	0.008432	0.203153	0.269279	FALSE	TRUE	FALSE	I9_PULMEM	0.617361	0.016878	pulmonary	TRUE	reported	textfile	5	1.19E+08	0.002547	4.71E-08	473971	basophil	TRUE	reported	igd	6.31E-05	29.88604	2	TRUE
rs1128099	A	G	A	G	-0.01456	0.000529	0.28544	0.255273	FALSE	FALSE	FALSE	I9_PULMEM	0.975491	0.017234	pulmonary	TRUE	reported	textfile	19	18307468	0.002268	1.41E-10	473994	basophil	TRUE	reported	igd	8.70E-05	41.22438	2	TRUE
rs116337C	A	G	A	G	0.012395	0.009122	0.636389	0.571393	FALSE	FALSE	FALSE	I9_PULMEM	0.54748	0.015164	pulmonary	TRUE	reported	textfile	15	69617688	0.002136	6.66E-09	473059	basophil	TRUE	reported	igd	7.12E-05	33.67354	2	TRUE
rs1171073	G	A	G	A	0.01157	0.004288	0.438906	0.428186	FALSE	FALSE	FALSE	I9_PULMEM	0.77749	0.015172	pulmonary	TRUE	reported	textfile	3	1.07E+08	0.002065	2.15E-08	472602	basophil	TRUE	reported	igd	6.64E-05	31.39242	2	TRUE
rs1177289	C	G	C	G	0.034127	-0.00582	0.277783	0.335307	FALSE	TRUE	FALSE	I9_PULMEM	0.714992	0.01594	pulmonary	TRUE	reported	textfile	7	1.43E+08	0.0023	9.23E-50	471976	basophil	TRUE	reported	igd	0.000466	220.1602	2	TRUE
rs1180134	A	G	A	G	0.023734	-0.04973	0.066978	0.099362	FALSE	FALSE	FALSE	I9_PULMEM	0.049417	0.02531	pulmonary	TRUE	reported	textfile	19	33726577	0.004201	1.65E-08	473999	basophil	TRUE	reported	igd	6.73E-05	31.91793	2	TRUE
rs1186222	T	C	T	C	-0.02202	-0.01028	0.461919	0.348782	FALSE	FALSE	FALSE	I9_PULMEM	0.514354	0.015771	pulmonary	TRUE	reported	textfile	7	75247329	0.002077	3.21E-26	473057	basophil	TRUE	reported	igd	0.000237	112.3473	2	TRUE
rs12075	A	G	A	G	0.027771	-0.00549	0.579533	0.532891	FALSE	FALSE	FALSE	I9_PULMEM	0.716129	0.01509	pulmonary	TRUE	reported	textfile	1	1.59E+08	0.002071	6.02E-41	473058	basophil	TRUE	reported	igd	0.00038	179.813	2	TRUE
rs1212392	A	G	A	G	-0.02777	-0.01776	0.440557	0.414055	FALSE	FALSE	FALSE	I9_PULMEM	0.243454	0.015224	pulmonary	TRUE	reported	textfile	1	2.05E+08	0.002057	1.73E-41	473983	basophil	TRUE	reported	igd	0.000384	182.282	2	TRUE
rs1214361	A	T	A	T	-0.02316	0.042612	0.116955	0.102946	FALSE	TRUE	FALSE	I9_PULMEM	0.087167	0.024911	pulmonary	TRUE	reported	textfile	1	1.51E+08	0.0032	4.72E-13	473957	basophil	TRUE	reported	igd	0.000111	52.39476	2	TRUE
rs1237651	C	T	C	T	-0.02068	-0.04091	0.162947	0.189159	FALSE	FALSE	FALSE	I9_PULMEM	0.033626	0.019254	pulmonary	TRUE	reported	textfile	9	22142756	0.002779	1.03E-13	473999	basophil	TRUE	reported	igd	0.000117	55.38678	2	TRUE
rs1244346	G	A	G	A	0.014544	0.004505	0.225894	0.18112	FALSE	FALSE	FALSE	I9_PULMEM	0.817532	0.019526	pulmonary	TRUE	reported	textfile	15	81888088	0.002451	3.04E-09	473996	basophil	TRUE	reported	igd	7.43E-05	35.21107	2	TRUE
rs1245368	T	C	T	C	-0.01827	-0.01803	0.695293	0.712584	FALSE	FALSE	FALSE	I9_PULMEM	0.277791	0.016615	pulmonary	TRUE	reported	textfile	17	37770005	0.00224	3.60E-16	473994	basophil	TRUE	reported	igd	0.00014	66.52413	2	TRUE
rs1245941	T	C	T	C	-0.01669	-0.01296	0.322962	0.360777	FALSE	FALSE	FALSE	I9_PULMEM	0.407734	0.015659	pulmonary	TRUE	reported	textfile	19	51728477	0.002188	2.44E-14	473996	basophil	TRUE	reported	igd	0.000123	58.20661	2	TRUE
rs1249769	C	A	C	A	0.014579	0.017838	0.371338	0.363102	FALSE	FALSE	FALSE	I9_PULMEM	0.252874	0.015601	pulmonary	TRUE	reported	textfile	3	27795397	0.002121	6.48E-12	473994	basophil	TRUE	reported	igd	9.97E-05	47.24679	2	TRUE
rs1292187	T	C	T	C	0.015838	0.028817	0.210936	0.161412	FALSE	FALSE	FALSE	I9_PULMEM	0.158288	0.020425	pulmonary	TRUE	reported	textfile	16	87933057	0.002547	5.19E-10	472847	basophil	TRUE	reported	igd	8.18E-05	38.66704	2	TRUE
rs1294181	C	T	C	T	-0.04635	0.019682	0.578198	0.556251	FALSE	FALSE	FALSE	I9_PULMEM	0.192337	0.015097	pulmonary	TRUE	reported	textfile	17	38159335	0.002071	########	473990	basophil	TRUE	reported	igd	0.001056	500.9492	2	TRUE
rs1295927	G	A	G	A	-0.02498	0.009531	0.447843	0.462468	FALSE	FALSE	FALSE	I9_PULMEM	0.526464	0.015048	pulmonary	TRUE	reported	textfile	17	57929535	0.002055	5.83E-34	473058	basophil	TRUE	reported	igd	0.000312	147.7017	2	TRUE
rs1308972	A	G	A	G	-0.08799	-0.03987	0.107756	0.092974	FALSE	FALSE	FALSE	I9_PULMEM	0.121688	0.02576	pulmonary	TRUE	reported	textfile	3	1.28E+08	0.0033	########	473990	basophil	TRUE	reported	igd	0.001498	710.9303	2	TRUE
rs1318896	T	G	T	G	-0.01912	0.016816	0.27945	0.33298	FALSE	FALSE	FALSE	I9_PULMEM	0.291169	0.015931	pulmonary	TRUE	reported	textfile	5	35853319	0.002278	5.08E-17	473985	basophil	TRUE	reported	igd	0.000149	70.42554	2	TRUE
rs1326772	A	G	A	G	-0.01405	0.000934	0.231732	0.199168	FALSE	FALSE	FALSE	I9_PULMEM	0.960556	0.018889	pulmonary	TRUE	reported	textfile	8	1.31E+08	0.002437	8.46E-09	473997	basophil	TRUE	reported	igd	7.01E-05	33.22418	2	TRUE
rs133866C	A	G	A	G	-0.01194	-0.02325	0.358085	0.441831	FALSE	FALSE	FALSE	I9_PULMEM	0.125442	0.015174	pulmonary	TRUE	reported	textfile	2	8440572	0.002131	2.15E-08	473997	basophil	TRUE	reported	igd	6.62E-05	31.39877	2	TRUE
rs1341976	T	C	T	C	0.016215	0.026132	0.562498	0.667485	FALSE	FALSE	FALSE	I9_PULMEM	0.100972	0.015933	pulmonary	TRUE	reported	textfile	2	2.19E+08	0.002066	4.43E-15	473058	basophil	TRUE	reported	igd	0.00013	61.59868	2	TRUE
rs1385952	G	C	G	C	0.064177	0.006853	0.025574	0.060313	FALSE	TRUE	FALSE	I9_PULMEM	0.828591	0.031652	pulmonary	TRUE	reported	textfile	21	36789420	0.006916	1.82E-20	474001	basophil	TRUE	reported	igd	0.000182	86.1087	2	TRUE
rs1424052	G	A	G	A	0.050739	0.050661	0.018962	0.004815	FALSE	FALSE	FALSE	I9_PULMEM	0.652124	0.112377	pulmonary	TRUE	reported	textfile	1	1.61E+08	0.008204	6.41E-10	473144	basophil	TRUE	reported	igd	8.08E-05	38.24993	2	TRUE

rs1427499	G	A	G	A	0.021037	0.016509	0.710264	0.693926	FALSE	FALSE	FALSE	I9_PULMEN	0.310753	0.016287	pulmonary	TRUE	reported textfile	2	1.45E+08	0.002263	1.57E-20	470746	basophil	TRUE	reported igd	0.000184	86.4165	2	TRUE
rs1469706	A	G	A	G	0.022392	0.011165	0.094107	0.120525	FALSE	FALSE	FALSE	I9_PULMEN	0.631245	0.023261	pulmonary	TRUE	reported textfile	12	27103449	0.003534	2.43E-10	473055	basophil	TRUE	reported igd	8.49E-05	40.14679	2	TRUE
rs1490077	T	C	T	C	0.026584	-0.01692	0.162151	0.206838	FALSE	FALSE	FALSE	I9_PULMEN	0.36432	0.018651	pulmonary	TRUE	reported textfile	7	50370254	0.002887	3.48E-20	473998	basophil	TRUE	reported igd	0.000179	84.79012	2	TRUE
rs1537061	C	T	C	T	-0.02508	0.018772	0.098713	0.118299	FALSE	FALSE	FALSE	I9_PULMEN	0.42125	0.02334	pulmonary	TRUE	reported textfile	1	87739219	0.003425	2.55E-13	473992	basophil	TRUE	reported igd	0.000113	53.61637	2	TRUE
rs1539174	G	C	G	C	0.046398	-0.00859	0.237332	0.204573	FALSE	TRUE	FALSE	I9_PULMEN	0.6441	0.018602	pulmonary	TRUE	reported textfile	10	974870	0.00241	1.51E-82	473996	basophil	TRUE	reported igd	0.000781	370.6488	2	TRUE
rs1598207	G	A	G	A	0.020189	0.04351	0.302655	0.335685	FALSE	FALSE	FALSE	I9_PULMEN	0.005835	0.015782	pulmonary	TRUE	reported textfile	2	1.49E+08	0.002229	1.41E-19	473062	basophil	TRUE	reported igd	0.000173	82.0366	2	TRUE
rs1633768	T	C	T	C	-0.01665	0.012294	0.275307	0.219024	FALSE	FALSE	FALSE	I9_PULMEN	0.498957	0.018182	pulmonary	TRUE	reported textfile	9	1.36E+08	0.002317	7.02E-13	471975	basophil	TRUE	reported igd	0.000109	51.62001	2	TRUE
rs1669340	T	G	T	G	-0.034	-0.00193	0.839194	0.809239	FALSE	FALSE	FALSE	I9_PULMEN	0.920056	0.019203	pulmonary	TRUE	reported textfile	3	3198380	0.002785	3.09E-34	472917	basophil	TRUE	reported igd	0.000315	149.0059	2	TRUE
rs1692363	T	G	T	G	0.014168	-0.02847	0.286039	0.360933	FALSE	FALSE	FALSE	I9_PULMEN	0.071128	0.015778	pulmonary	TRUE	reported textfile	8	59567885	0.002274	4.79E-10	473983	basophil	TRUE	reported igd	8.19E-05	38.81811	2	TRUE
rs1692808	G	A	G	A	-0.02773	0.014445	0.11345	0.152529	FALSE	FALSE	FALSE	I9_PULMEN	0.490906	0.020969	pulmonary	TRUE	reported textfile	11	2322954	0.003242	1.24E-17	473996	basophil	TRUE	reported igd	0.000154	73.1808	2	TRUE
rs1698948	C	T	C	T	-0.02109	0.048344	0.274125	0.244612	FALSE	FALSE	FALSE	I9_PULMEN	0.005128	0.017273	pulmonary	TRUE	reported textfile	20	43537200	0.002287	3.15E-20	471507	basophil	TRUE	reported igd	0.00018	85.02304	2	TRUE
rs1761333	T	C	T	C	-0.01682	0.033086	0.128384	0.150933	FALSE	FALSE	FALSE	I9_PULMEN	0.116113	0.021057	pulmonary	TRUE	reported textfile	1	1.18E+08	0.003077	4.71E-08	472602	basophil	TRUE	reported igd	6.32E-05	29.88811	2	TRUE
rs1762558	A	G	A	G	0.01499	0.003374	0.263625	0.286927	FALSE	FALSE	FALSE	I9_PULMEN	0.838826	0.016588	pulmonary	TRUE	reported textfile	1	1.99E+08	0.002328	1.25E-10	473983	basophil	TRUE	reported igd	8.75E-05	41.46059	2	TRUE
rs1775869	T	C	T	C	-0.12474	0.060829	0.029551	0.044003	FALSE	FALSE	FALSE	I9_PULMEN	0.097521	0.036711	pulmonary	TRUE	reported textfile	18	60920854	0.006145	1.47E-91	473056	basophil	TRUE	reported igd	0.00087	412.0783	2	TRUE
rs1786028	T	C	T	C	0.104693	-0.05152	0.011189	0.056686	FALSE	FALSE	FALSE	I9_PULMEN	0.115855	0.032765	pulmonary	TRUE	reported textfile	12	51740350	0.009859	2.62E-26	470511	basophil	TRUE	reported igd	0.00024	112.7633	2	TRUE
rs1804999	G	A	G	A	0.011333	-0.00471	0.505883	0.421314	FALSE	FALSE	FALSE	I9_PULMEN	0.757509	0.015243	pulmonary	TRUE	reported textfile	1	1.56E+08	0.002053	3.48E-08	473996	basophil	TRUE	reported igd	6.43E-05	30.47264	2	TRUE
rs1820909	A	G	A	G	-0.08765	-0.02078	0.009832	0.00298	FALSE	FALSE	FALSE	I9_PULMEN	0.881235	0.139058	pulmonary	TRUE	reported textfile	7	92239892	0.010695	2.62E-16	472665	basophil	TRUE	reported igd	0.000142	67.16613	2	TRUE
rs2028900	T	C	T	C	-0.01396	-0.01997	0.449306	0.400416	FALSE	FALSE	FALSE	I9_PULMEN	0.192784	0.015336	pulmonary	TRUE	reported textfile	2	85767735	0.002059	1.25E-11	472602	basophil	TRUE	reported igd	9.73E-05	45.96807	2	TRUE
rs2070596	A	T	A	T	0.022675	0.041296	0.211403	0.120529	FALSE	TRUE	FALSE	I9_PULMEN	0.074578	0.02316	pulmonary	TRUE	reported textfile	15	50545159	0.002529	3.30E-19	472910	basophil	TRUE	reported igd	0.00017	80.38872	2	TRUE
rs2074585	A	G	A	G	-0.03089	0.006947	0.514859	0.533141	FALSE	FALSE	FALSE	I9_PULMEN	0.644625	0.015061	pulmonary	TRUE	reported textfile	15	91009484	0.00205	2.71E-51	474000	basophil	TRUE	reported igd	0.000479	227.1113	2	TRUE
rs2089979	G	A	G	A	-0.01391	0.016584	0.415685	0.432492	FALSE	FALSE	FALSE	I9_PULMEN	0.2728	0.015123	pulmonary	TRUE	reported textfile	3	1.97E+08	0.002084	2.59E-11	472599	basophil	TRUE	reported igd	9.42E-05	44.53814	2	TRUE
rs2118140	G	A	G	A	0.018347	0.01898	0.528383	0.40315	FALSE	FALSE	FALSE	I9_PULMEN	0.21345	0.015256	pulmonary	TRUE	reported textfile	12	66698461	0.002052	4.03E-19	473990	basophil	TRUE	reported igd	0.000169	79.94174	2	TRUE
rs2229092	C	A	C	A	0.025697	0.034217	0.062139	0.054046	FALSE	FALSE	FALSE	I9_PULMEN	0.303681	0.033266	pulmonary	TRUE	reported textfile	6	31540757	0.004247	1.48E-09	472567	basophil	TRUE	reported igd	7.75E-05	36.6099	2	TRUE
rs2239635	C	G	C	G	0.034388	-0.01092	0.70425	0.625165	FALSE	TRUE	FALSE	I9_PULMEN	0.480819	0.015491	pulmonary	TRUE	reported textfile	14	23588731	0.002255	1.89E-52	473995	basophil	TRUE	reported igd	0.00049	232.5514	2	TRUE
rs2271352	C	G	C	G	0.042265	-0.01092	0.225593	0.225645	FALSE	TRUE	FALSE	I9_PULMEN	0.543865	0.017991	pulmonary	TRUE	reported textfile	5	1.26E+08	0.00247	1.44E-65	473993	basophil	TRUE	reported igd	0.000617	292.7966	2	TRUE
rs2273770	T	C	T	C	0.011659	0.010352	0.398729	0.325687	FALSE	FALSE	FALSE	I9_PULMEN	0.519407	0.016067	pulmonary	TRUE	reported textfile	9	77755469	0.002124	4.16E-08	457734	basophil	TRUE	reported igd	6.58E-05	30.13087	2	TRUE
rs2276066	G	A	G	A	0.013903	-0.03022	0.648326	0.731437	FALSE	FALSE	FALSE	I9_PULMEN	0.074587	0.016951	pulmonary	TRUE	reported textfile	11	70007311	0.002145	9.38E-11	472597	basophil	TRUE	reported igd	8.89E-05	42.01077	2	TRUE
rs2282986	C	T	C	T	-0.08865	-0.04624	0.018065	0.026177	FALSE	FALSE	FALSE	I9_PULMEN	0.324943	0.046977	pulmonary	TRUE	reported textfile	7	92299545	0.007728	1.98E-30	472603	basophil	TRUE	reported igd	0.000278	131.5985	2	TRUE
rs2286599	A	G	A	G	0.023189	-0.05162	0.141728	0.103461	FALSE	FALSE	FALSE	I9_PULMEN	0.040716	0.025225	pulmonary	TRUE	reported textfile	12	6499533	0.003001	1.15E-14	469154	basophil	TRUE	reported igd	0.000127	59.70768	2	TRUE
rs2289511	A	G	A	G	-0.01639	0.002369	0.582293	0.569582	FALSE	FALSE	FALSE	I9_PULMEN	0.875716	0.015148	pulmonary	TRUE	reported textfile	14	88454910	0.002078	3.30E-15	473058	basophil	TRUE	reported igd	0.000131	62.18032	2	TRUE
rs2294199	C	T	C	T	-0.01632	0.006595	0.470274	0.541361	FALSE	FALSE	FALSE	I9_PULMEN	0.661409	0.015058	pulmonary	TRUE	reported textfile	22	45631165	0.00205	1.81E-15	473999	basophil	TRUE	reported igd	0.000134	63.36133	2	TRUE
rs2298989	C	T	C	T	-0.0116	-0.00033	0.425369	0.382552	FALSE	FALSE	FALSE	I9_PULMEN	0.983133	0.015449	pulmonary	TRUE	reported textfile	4	1.11E+08	0.002083	2.65E-08	472599	basophil	TRUE	reported igd	6.56E-05	30.99638	2	TRUE
rs2524079	A	G	A	G	0.031209	-0.03973	0.419059	0.390842	FALSE	FALSE	FALSE	I9_PULMEN	0.009841	0.01539	pulmonary	TRUE	reported textfile	6	31242174	0.002077	5.46E-51	472890	basophil	TRUE	reported igd	0.000477	225.7797	2	TRUE
rs2594836	A	G	A	G	-0.01778	0.026235	0.722276	0.728022	FALSE	FALSE	FALSE	I9_PULMEN	0.119732	0.016862	pulmonary	TRUE	reported textfile	5	1.73E+08	0.002319	1.87E-14	474000	basophil	TRUE	reported igd	0.000124	58.76431	2	TRUE
rs2606724	A	G	A	G	0.015734	0.007694	0.453028	0.503729	FALSE	FALSE	FALSE	I9_PULMEN	0.609485	0.015062	pulmonary	TRUE	reported textfile	11	1.14E+08	0.002124	1.33E-13	473993	basophil	TRUE	reported igd	0.000116	54.8741	2	TRUE
rs2607278	T	C	T	C	0.022731	0.006987	0.306108	0.361609	FALSE	FALSE	FALSE	I9_PULMEN	0.654856	0.01563	pulmonary	TRUE	reported textfile	19	16568197	0.002225	1.79E-24	472910	basophil	TRUE	reported igd	0.000221	104.3699	2	TRUE
rs2738104	A	G	A	G	0.018357	-0.00152	0.67231	0.58174	FALSE	FALSE	FALSE	I9_PULMEN	0.92051	0.015219	pulmonary	TRUE	reported textfile	8	6792257	0.00218	4.01E-17	472911	basophil	TRUE	reported igd	0.00015	70.90692	2	TRUE
rs2805921	A	G	A	G	0.012974	0.034015	0.247972	0.345489	FALSE	FALSE	FALSE	I9_PULMEN	0.031311	0.015798	pulmonary	TRUE	reported textfile	10	70847549	0.002377	4.92E-08	473996	basophil	TRUE	reported igd	6.28E-05	29.79117	2	TRUE
rs2811708	T	G	T	G	-0.01727	-0.00297	0.266088	0.220772	FALSE	FALSE	FALSE	I9_PULMEN	0.86937	0.018062	pulmonary	TRUE	reported textfile	9	21973422	0.00233	1.32E-13	472914	basophil	TRUE	reported igd	0.000116	54.90596	2	TRUE
rs2834670	G	A	G	A	0.028259	-0.02961	0.17203	0.174844	FALSE	FALSE	FALSE	I9_PULMEN	0.138415	0.019982	pulmonary	TRUE	reported textfile	21	36280376	0.003218	1.69E-18	472917	basophil	TRUE	reported igd	0.000163	77.11514	2	TRUE
rs2836241	C	T	C	T	0.013945	-0.0254	0.404821	0.562314	FALSE	FALSE	FALSE	I9_PULMEN	0.093493	0.015143	pulmonary	TRUE	reported textfile	21	39621213	0.002086	2.37E-11	474001	basophil	TRUE	reported igd	9.43E-05	44.68961	2	TRUE
rs2836439	A	T	A	T	0.020338	0.040939	0.132089	0.128632	FALSE	TRUE	FALSE	I9_PULMEN	0.06667	0.022324	pulmonary	TRUE	reported textfile	15	42677173	0.003019	1.69E-11	474000	basophil	TRUE	reported igd	9.57E-05	45.3825	2	TRUE
rs2860773	T	G	T	G	0.019124	-0.00458	0.407715	0.337645	FALSE	FALSE	FALSE	I9_PULMEN	0.773029	0.015875	pulmonary	TRUE	reported textfile	2	65665641	0.002085	5.03E-20	472598	basophil	TRUE	reported igd	0.000178	84.12857	2	TRUE
rs2864782	A	C	A	C	-0.01751	0.006805	0.267348	0.313067	FALSE	FALSE	FALSE	I9_PULMEN	0.673587	0.016156	pulmonary	TRUE	reported textfile	4	83585062	0.002312	3.84E-14	473997	basophil	TRUE	reported igd	0.000121	57.345	2	TRUE

rs2959356	G	A	G	A	0.014017	-0.02025	0.298096	0.325541	FALSE	FALSE	FALSE	I9_PULMEN	0.205794	0.016006	pulmonary	TRUE	reported	textfile	18	23590203	0.002241	4.14E-10	472600	basophil	TRUE	reported	igd	8.28E-05	39.12232	2	TRUE
rs2967595	T	C	T	C	-0.02605	0.025473	0.167857	0.150044	FALSE	FALSE	FALSE	I9_PULMEN	0.225394	0.021012	pulmonary	TRUE	reported	textfile	19	8566299	0.00277	5.59E-21	473039	basophil	TRUE	reported	igd	0.000187	88.41393	2	TRUE
rs2977799	G	A	G	A	0.027476	0.015628	0.50924	0.466327	FALSE	FALSE	FALSE	I9_PULMEN	0.299446	0.015062	pulmonary	TRUE	reported	textfile	8	6696927	0.002057	1.17E-40	473057	basophil	TRUE	reported	igd	0.000377	178.4171	2	TRUE
rs2998286	C	T	C	T	0.020883	-0.0447	0.769831	0.799034	FALSE	FALSE	FALSE	I9_PULMEN	0.017197	0.018761	pulmonary	TRUE	reported	textfile	10	28780373	0.002443	1.31E-17	473999	basophil	TRUE	reported	igd	0.000154	73.06965	2	TRUE
rs3071	C	A	C	A	-0.01635	0.02666	0.298273	0.401029	FALSE	FALSE	FALSE	I9_PULMEN	0.081386	0.015298	pulmonary	TRUE	reported	textfile	10	1.02E+08	0.002239	2.95E-13	473996	basophil	TRUE	reported	igd	0.000112	53.32434	2	TRUE
rs310631	A	C	A	C	0.012281	-0.0038	0.411889	0.343046	FALSE	FALSE	FALSE	I9_PULMEN	0.812801	0.016026	pulmonary	TRUE	reported	textfile	20	62196253	0.002091	4.42E-09	471507	basophil	TRUE	reported	igd	7.32E-05	34.49511	2	TRUE
rs3181077	T	C	T	C	-0.03706	0.021536	0.718492	0.74186	FALSE	FALSE	FALSE	I9_PULMEN	0.212245	0.017264	pulmonary	TRUE	reported	textfile	3	46250652	0.00228	2.23E-59	473997	basophil	TRUE	reported	igd	0.000557	264.261	2	TRUE
rs3184504	C	T	C	T	-0.02822	-0.0286	0.518576	0.59144	FALSE	FALSE	FALSE	I9_PULMEN	0.060823	0.015254	pulmonary	TRUE	reported	textfile	12	1.12E+08	0.002051	5.08E-43	471515	basophil	TRUE	reported	igd	0.000401	189.3132	2	TRUE
rs3409784	T	C	T	C	0.043291	-0.01439	0.056678	0.089315	FALSE	FALSE	FALSE	I9_PULMEN	0.589059	0.026637	pulmonary	TRUE	reported	textfile	17	56358429	0.004593	4.58E-21	472121	basophil	TRUE	reported	igd	0.000188	88.83835	2	TRUE
rs3415872	A	G	A	G	0.094813	-0.00659	0.028263	0.063407	FALSE	FALSE	FALSE	I9_PULMEN	0.831035	0.030901	pulmonary	TRUE	reported	textfile	19	38903032	0.006172	3.24E-53	473996	basophil	TRUE	reported	igd	0.000498	235.9838	2	TRUE
rs3428853	T	C	T	C	-0.01516	-0.00804	0.273948	0.319817	FALSE	FALSE	FALSE	I9_PULMEN	0.619711	0.016195	pulmonary	TRUE	reported	textfile	22	24642009	0.002317	6.34E-11	472911	basophil	TRUE	reported	igd	9.05E-05	42.79299	2	TRUE
rs3437757	C	A	C	A	0.013372	-0.01087	0.251754	0.194987	FALSE	FALSE	FALSE	I9_PULMEN	0.566027	0.018948	pulmonary	TRUE	reported	textfile	10	1.04E+08	0.002363	1.56E-08	473062	basophil	TRUE	reported	igd	6.77E-05	32.02311	2	TRUE
rs34500	A	G	A	G	-0.02629	-0.07051	0.084024	0.092955	FALSE	FALSE	FALSE	I9_PULMEN	0.007921	0.026552	pulmonary	TRUE	reported	textfile	5	98294885	0.003721	1.67E-12	472911	basophil	TRUE	reported	igd	0.000106	49.91073	2	TRUE
rs3463865	C	G	C	G	0.012153	0.01383	0.354416	0.391777	FALSE	TRUE	FALSE	I9_PULMEN	0.370311	0.015437	pulmonary	TRUE	reported	textfile	14	35366850	0.002151	1.66E-08	473996	basophil	TRUE	reported	igd	6.73E-05	31.92158	2	TRUE
rs3478050	G	A	G	A	0.03512	-0.014	0.065683	0.039798	FALSE	FALSE	FALSE	I9_PULMEN	0.715626	0.038429	pulmonary	TRUE	reported	textfile	22	41405753	0.004154	2.96E-17	473057	basophil	TRUE	reported	igd	0.000151	71.4783	2	TRUE
rs3485093	A	G	A	G	0.013806	-0.01438	0.349046	0.319477	FALSE	FALSE	FALSE	I9_PULMEN	0.372318	0.01612	pulmonary	TRUE	reported	textfile	3	47063910	0.002243	7.75E-10	473986	basophil	TRUE	reported	igd	7.99E-05	37.8857	2	TRUE
rs3504501	A	C	A	C	-0.01138	-0.00506	0.455669	0.446454	FALSE	FALSE	FALSE	I9_PULMEN	0.739459	0.015205	pulmonary	TRUE	reported	textfile	6	1.07E+08	0.002069	3.87E-08	473993	basophil	TRUE	reported	igd	6.39E-05	30.26848	2	TRUE
rs357613	A	G	A	G	-0.01519	0.016829	0.507364	0.118942	FALSE	FALSE	FALSE	I9_PULMEN	0.468288	0.023204	pulmonary	TRUE	reported	textfile	5	1.51E+08	0.002179	3.26E-12	433368	basophil	TRUE	reported	igd	0.000112	48.59584	2	TRUE
rs3731332	T	C	T	C	-0.10399	0.041051	0.022182	0.018182	FALSE	FALSE	FALSE	I9_PULMEN	0.467371	0.056484	pulmonary	TRUE	reported	textfile	7	92300568	0.007064	5.36E-49	473062	basophil	TRUE	reported	igd	0.000458	216.6897	2	TRUE
rs377763	A	C	A	C	-0.0273	-0.03651	0.214238	0.174328	FALSE	FALSE	FALSE	I9_PULMEN	0.065167	0.019799	pulmonary	TRUE	reported	textfile	6	32199144	0.002499	9.38E-28	471514	basophil	TRUE	reported	igd	0.000253	119.3239	2	TRUE
rs3781452	T	C	T	C	0.019847	-0.01588	0.632095	0.555431	FALSE	FALSE	FALSE	I9_PULMEN	0.29306	0.015105	pulmonary	TRUE	reported	textfile	10	1.26E+08	0.002131	1.34E-20	472140	basophil	TRUE	reported	igd	0.000184	86.74032	2	TRUE
rs3892360	G	C	G	C	0.043053	0.016366	0.306641	0.359124	FALSE	TRUE	FALSE	I9_PULMEN	0.294087	0.015599	pulmonary	TRUE	reported	textfile	13	41184078	0.005941	4.46E-13	68110	basophil	TRUE	reported	igd	0.00077	52.51399	2	TRUE
rs4060971	T	C	T	C	-0.02066	-0.01366	0.551092	0.575278	FALSE	FALSE	FALSE	I9_PULMEN	0.368877	0.015204	pulmonary	TRUE	reported	textfile	1	21506930	0.002069	1.85E-23	473994	basophil	TRUE	reported	igd	0.00021	99.70979	2	TRUE
rs42030	A	G	A	G	0.030212	-0.00085	0.076053	0.085621	FALSE	FALSE	FALSE	I9_PULMEN	0.97483	0.026849	pulmonary	TRUE	reported	textfile	7	92236421	0.003869	6.09E-15	473061	basophil	TRUE	reported	igd	0.000129	60.9761	2	TRUE
rs4324460	G	T	G	T	0.024167	-0.01622	0.218923	0.132905	FALSE	FALSE	FALSE	I9_PULMEN	0.466101	0.022254	pulmonary	TRUE	reported	textfile	3	3120542	0.002509	6.15E-22	472560	basophil	TRUE	reported	igd	0.000196	92.77743	2	TRUE
rs4475963	G	T	G	T	-0.01352	0.032105	0.370213	0.388451	FALSE	FALSE	FALSE	I9_PULMEN	0.03713	0.015403	pulmonary	TRUE	reported	textfile	12	1.29E+08	0.002127	2.11E-10	472603	basophil	TRUE	reported	igd	8.55E-05	40.42719	2	TRUE
rs4557713	G	A	G	A	-0.04532	-0.02532	0.045208	0.071722	FALSE	FALSE	FALSE	I9_PULMEN	0.390064	0.029461	pulmonary	TRUE	reported	textfile	8	48651633	0.005335	2.08E-17	465986	basophil	TRUE	reported	igd	0.000155	72.16846	2	TRUE
rs4602187	T	C	T	C	0.014094	0.029513	0.683135	0.706625	FALSE	FALSE	FALSE	I9_PULMEN	0.074319	0.016537	pulmonary	TRUE	reported	textfile	2	1.82E+08	0.002227	2.53E-10	472600	basophil	TRUE	reported	igd	8.47E-05	40.05219	2	TRUE
rs4705908	A	G	A	G	-0.01166	-0.02586	0.419818	0.296291	FALSE	FALSE	FALSE	I9_PULMEN	0.11635	0.016468	pulmonary	TRUE	reported	textfile	5	1.31E+08	0.002089	2.43E-08	472911	basophil	TRUE	reported	igd	6.59E-05	31.15433	2	TRUE
rs4763817	T	C	T	C	0.013462	-0.0036	0.738634	0.743298	FALSE	FALSE	FALSE	I9_PULMEN	0.83475	0.017255	pulmonary	TRUE	reported	textfile	12	12481296	0.002359	1.18E-08	474000	basophil	TRUE	reported	igd	6.87E-05	32.56578	2	TRUE
rs4876400	G	A	G	A	-0.01709	0.018737	0.626316	0.641256	FALSE	FALSE	FALSE	I9_PULMEN	0.231273	0.015652	pulmonary	TRUE	reported	textfile	8	1.19E+08	0.002111	6.00E-16	473992	basophil	TRUE	reported	igd	0.000138	65.50923	2	TRUE
rs4911102	T	C	T	C	-0.02169	-0.00744	0.234267	0.308869	FALSE	FALSE	FALSE	I9_PULMEN	0.647335	0.016258	pulmonary	TRUE	reported	textfile	20	31179500	0.00244	6.46E-19	473058	basophil	TRUE	reported	igd	0.000167	79.04926	2	TRUE
rs4912807	C	G	C	G	0.012874	-0.01634	0.617494	0.657036	FALSE	TRUE	FALSE	I9_PULMEN	0.300491	0.015784	pulmonary	TRUE	reported	textfile	5	1.42E+08	0.002107	1.02E-09	472913	basophil	TRUE	reported	igd	7.89E-05	37.33328	2	TRUE
rs4941839	A	G	A	G	-0.01338	-0.00177	0.714109	0.760732	FALSE	FALSE	FALSE	I9_PULMEN	0.919803	0.017616	pulmonary	TRUE	reported	textfile	13	37025916	0.002291	5.43E-09	471973	basophil	TRUE	reported	igd	7.22E-05	34.08792	2	TRUE
rs5569060	A	G	A	G	-0.03494	-0.01237	0.322693	0.314361	FALSE	FALSE	FALSE	I9_PULMEN	0.444416	0.016172	pulmonary	TRUE	reported	textfile	8	6902043	0.0022	8.93E-57	473967	basophil	TRUE	reported	igd	0.000532	252.2311	2	TRUE
rs5604307	A	G	A	G	-0.02603	0.029649	0.071468	0.054102	FALSE	FALSE	FALSE	I9_PULMEN	0.370647	0.033118	pulmonary	TRUE	reported	textfile	1	2.48E+08	0.003975	6.04E-11	472603	basophil	TRUE	reported	igd	9.07E-05	42.86525	2	TRUE
rs561102	T	C	T	C	-0.01406	-0.0304	0.65494	0.668464	FALSE	FALSE	FALSE	I9_PULMEN	0.05596	0.015907	pulmonary	TRUE	reported	textfile	18	9851388	0.002162	8.12E-11	471517	basophil	TRUE	reported	igd	8.97E-05	42.29192	2	TRUE
rs5617956	A	G	A	G	0.014617	-0.01432	0.389066	0.354216	FALSE	FALSE	FALSE	I9_PULMEN	0.363192	0.015751	pulmonary	TRUE	reported	textfile	7	1.3E+08	0.002124	6.10E-12	473989	basophil	TRUE	reported	igd	9.99E-05	47.35934	2	TRUE
rs5638817	T	G	T	G	0.031399	-0.01178	0.293002	0.283397	FALSE	FALSE	FALSE	I9_PULMEN	0.4813	0.016731	pulmonary	TRUE	reported	textfile	7	28724374	0.002277	3.34E-43	472911	basophil	TRUE	reported	igd	0.000402	190.1532	2	TRUE
rs5640612	T	G	T	G	-0.03495	-0.00728	0.190072	0.246028	FALSE	FALSE	FALSE	I9_PULMEN	0.676717	0.017467	pulmonary	TRUE	reported	textfile	4	87940205	0.002607	6.04E-41	473999	basophil	TRUE	reported	igd	0.000379	179.6847	2	TRUE
rs5750494	G	T	G	T	-0.01312	-0.00651	0.629062	0.594913	FALSE	FALSE	FALSE	I9_PULMEN	0.670006	0.015288	pulmonary	TRUE	reported	textfile	22	38176979	0.002121	6.46E-10	473992	basophil	TRUE	reported	igd	8.07E-05	38.24599	2	TRUE
rs5910703	T	C	T	C	-0.01399	-0.05358	0.224774	0.260607	FALSE	FALSE	FALSE	I9_PULMEN	0.00192	0.017271	pulmonary	TRUE	reported	textfile	3	1.23E+08	0.002451	1.17E-08	473055	basophil	TRUE	reported	igd	6.89E-05	32.58901	2	TRUE
rs602229	C	G	C	G	0.012728	0.000942	0.692937	0.71005	FALSE	TRUE	FALSE	I9_PULMEN	0.954577	0.01653	pulmonary	TRUE	reported	textfile	18	51789396	0.002221	1.03E-08	472915	basophil	TRUE	reported	igd	6.94E-05	32.84138	2	TRUE
rs6029234	C	G	C	G	0.012547	0.019199	0.625866	0.595929	FALSE	TRUE	FALSE	I9_PULMEN	0.209043	0.015284	pulmonary	TRUE	reported	textfile	20	39259278	0.002122	3.46E-09	472912	basophil	TRUE	reported	igd	7.39E-05	34.96127	2	TRUE

rs6045612	T	C	T	C	-0.01673	0.001637	0.26377	0.258201	FALSE	FALSE	FALSE	I9_PULMEM	0.92376	0.017103	pulmonary	TRUE	reported	textfile	20	1931001	0.00233	7.18E-13	473999	basophil	TRUE	reported	igd	0.000109	51.58054	2	TRUE
rs6091176	T	C	T	C	0.01369	0.010351	0.261115	0.235061	FALSE	FALSE	FALSE	I9_PULMEM	0.558854	0.017708	pulmonary	TRUE	reported	textfile	20	49150510	0.002337	4.80E-09	473996	basophil	TRUE	reported	igd	7.24E-05	34.31529	2	TRUE
rs6141781	T	C	T	C	0.032512	-0.03052	0.147056	0.173633	FALSE	FALSE	FALSE	I9_PULMEM	0.12522	0.019905	pulmonary	TRUE	reported	textfile	20	31263342	0.002901	4.12E-29	473059	basophil	TRUE	reported	igd	0.000265	125.6001	2	TRUE
rs6202160	G	T	G	T	-0.03429	0.011024	0.050595	0.054525	FALSE	FALSE	FALSE	I9_PULMEM	0.73829	0.032993	pulmonary	TRUE	reported	textfile	15	64548734	0.004663	2.00E-13	473997	basophil	TRUE	reported	igd	0.000114	54.07572	2	TRUE
rs6210547	A	G	A	G	-0.03167	0.075948	0.051684	0.013942	FALSE	FALSE	FALSE	I9_PULMEM	0.22903	0.06314	pulmonary	TRUE	reported	textfile	2	8735869	0.004658	1.09E-11	473060	basophil	TRUE	reported	igd	9.77E-05	46.2182	2	TRUE
rs6216067	C	T	C	T	-0.01663	-0.01012	0.296123	0.372293	FALSE	FALSE	FALSE	I9_PULMEM	0.536336	0.016362	pulmonary	TRUE	reported	textfile	2	1.12E+08	0.002285	3.57E-13	471969	basophil	TRUE	reported	igd	0.000112	52.94212	2	TRUE
rs6421984	C	T	C	T	0.018366	-0.03182	0.514051	0.433185	FALSE	FALSE	FALSE	I9_PULMEM	0.036418	0.015211	pulmonary	TRUE	reported	textfile	11	305619	0.002068	6.97E-19	465819	basophil	TRUE	reported	igd	0.000169	78.87261	2	TRUE
rs6543144	G	A	G	A	0.013669	0.007239	0.312924	0.354949	FALSE	FALSE	FALSE	I9_PULMEM	0.64528	0.015725	pulmonary	TRUE	reported	textfile	2	1.03E+08	0.002219	7.43E-10	473059	basophil	TRUE	reported	igd	8.02E-05	37.94522	2	TRUE
rs6557615	A	G	A	G	-0.01878	0.014135	0.681024	0.617984	FALSE	FALSE	FALSE	I9_PULMEM	0.360685	0.015464	pulmonary	TRUE	reported	textfile	8	22940647	0.002208	1.90E-17	472916	basophil	TRUE	reported	igd	0.000153	72.33441	2	TRUE
rs6671847	A	G	A	G	0.01357	-0.0252	0.508234	0.455808	FALSE	FALSE	FALSE	I9_PULMEM	0.094757	0.015082	pulmonary	TRUE	reported	textfile	1	1.61E+08	0.002058	4.44E-11	472906	basophil	TRUE	reported	igd	9.19E-05	43.47776	2	TRUE
rs6717590	T	C	T	C	0.026075	0.010744	0.108202	0.137132	FALSE	FALSE	FALSE	I9_PULMEM	0.621328	0.02175	pulmonary	TRUE	reported	textfile	15	1.02E+08	0.003356	8.24E-15	458198	basophil	TRUE	reported	igd	0.000132	60.36745	2	TRUE
rs6734238	G	A	G	A	0.012064	-0.02805	0.402301	0.3032	FALSE	FALSE	FALSE	I9_PULMEM	0.086286	0.016353	pulmonary	TRUE	reported	textfile	2	1.14E+08	0.002085	7.37E-09	472600	basophil	TRUE	reported	igd	7.08E-05	33.47871	2	TRUE
rs6780544	A	G	A	G	-0.01636	-0.01284	0.62255	0.66787	FALSE	FALSE	FALSE	I9_PULMEM	0.419796	0.015915	pulmonary	TRUE	reported	textfile	3	1.87E+08	0.00211	9.32E-15	474000	basophil	TRUE	reported	igd	0.000127	60.1394	2	TRUE
rs6927569	C	T	C	T	0.021693	-0.00186	0.523015	0.616858	FALSE	FALSE	FALSE	I9_PULMEM	0.904586	0.015489	pulmonary	TRUE	reported	textfile	6	1.1E+08	0.002051	4.22E-26	473998	basophil	TRUE	reported	igd	0.000236	111.8681	2	TRUE
rs695113	T	C	T	C	0.01697	-0.03519	0.696269	0.659122	FALSE	FALSE	FALSE	I9_PULMEM	0.026662	0.015879	pulmonary	TRUE	reported	textfile	11	1.29E+08	0.002242	3.93E-14	472598	basophil	TRUE	reported	igd	0.000121	57.29156	2	TRUE
rs6993770	T	A	T	A	-0.01824	-0.03815	0.287084	0.224459	FALSE	TRUE	FALSE	I9_PULMEM	0.035185	0.018111	pulmonary	TRUE	reported	textfile	8	1.07E+08	0.00226	7.50E-16	474000	basophil	TRUE	reported	igd	0.000137	65.10891	2	TRUE
rs7044519	G	T	G	T	0.016724	0.009008	0.16139	0.127804	FALSE	FALSE	FALSE	I9_PULMEM	0.688676	0.022483	pulmonary	TRUE	reported	textfile	9	82233842	0.002799	2.37E-09	472600	basophil	TRUE	reported	igd	7.55E-05	35.70037	2	TRUE
rs7078507	G	A	G	A	-0.01607	-0.00872	0.609239	0.61521	FALSE	FALSE	FALSE	I9_PULMEM	0.571651	0.015413	pulmonary	TRUE	reported	textfile	10	77140864	0.002107	2.54E-14	471518	basophil	TRUE	reported	igd	0.000123	58.17748	2	TRUE
rs7139108	T	G	T	G	0.015904	-0.00048	0.170388	0.176248	FALSE	FALSE	FALSE	I9_PULMEM	0.980434	0.019699	pulmonary	TRUE	reported	textfile	16	74523273	0.002723	5.34E-09	473977	basophil	TRUE	reported	igd	7.20E-05	34.11266	2	TRUE
rs7156267	C	T	C	T	0.020951	-0.02773	0.07867	0.086868	FALSE	FALSE	FALSE	I9_PULMEM	0.29729	0.026603	pulmonary	TRUE	reported	textfile	7	1.05E+08	0.003836	4.84E-08	473994	basophil	TRUE	reported	igd	6.29E-05	29.82981	2	TRUE
rs7196129	C	T	C	T	0.016801	-0.0335	0.529408	0.585378	FALSE	FALSE	FALSE	I9_PULMEM	0.029231	0.015363	pulmonary	TRUE	reported	textfile	16	30471109	0.002081	7.19E-16	473980	basophil	TRUE	reported	igd	0.000138	65.18149	2	TRUE
rs7250849	T	G	T	G	0.021532	-0.00892	0.107192	0.162235	FALSE	FALSE	FALSE	I9_PULMEM	0.661893	0.020391	pulmonary	TRUE	reported	textfile	19	52158316	0.003328	1.01E-10	472600	basophil	TRUE	reported	igd	8.86E-05	41.8601	2	TRUE
rs7253094	C	G	C	G	-0.01564	-0.01377	0.671446	0.688374	FALSE	TRUE	FALSE	I9_PULMEM	0.395884	0.016218	pulmonary	TRUE	reported	textfile	19	40223884	0.002267	5.51E-12	436632	basophil	TRUE	reported	igd	0.000109	47.58361	2	TRUE
rs7269729	C	G	C	G	-0.01753	0.031653	0.180908	0.140675	FALSE	TRUE	FALSE	I9_PULMEM	0.143619	0.021644	pulmonary	TRUE	reported	textfile	14	93069980	0.002694	8.00E-11	472914	basophil	TRUE	reported	igd	8.95E-05	42.33673	2	TRUE
rs7272163	A	C	A	C	-0.01932	0.000457	0.237093	0.247564	FALSE	FALSE	FALSE	I9_PULMEM	0.979094	0.017426	pulmonary	TRUE	reported	textfile	14	75807762	0.002422	1.60E-15	473994	basophil	TRUE	reported	igd	0.000134	63.60398	2	TRUE
rs7278690	T	C	T	C	-0.01395	-0.04114	0.222439	0.146749	FALSE	FALSE	FALSE	I9_PULMEM	0.053308	0.021288	pulmonary	TRUE	reported	textfile	10	50376394	0.002461	1.48E-08	473999	basophil	TRUE	reported	igd	6.78E-05	32.11712	2	TRUE
rs7285377	T	G	T	G	0.014495	0.007059	0.284156	0.335729	FALSE	FALSE	FALSE	I9_PULMEM	0.656953	0.015894	pulmonary	TRUE	reported	textfile	22	19987202	0.002273	1.86E-10	472600	basophil	TRUE	reported	igd	8.60E-05	40.6664	2	TRUE
rs7302229	T	C	T	C	-0.05721	-0.08476	0.010925	0.00427	FALSE	FALSE	FALSE	I9_PULMEM	0.459261	0.114529	pulmonary	TRUE	reported	textfile	19	33772811	0.010051	1.29E-08	473139	basophil	TRUE	reported	igd	6.85E-05	32.40066	2	TRUE
rs7304925	A	G	A	G	-0.0372	0.018099	0.058507	0.026619	FALSE	FALSE	FALSE	I9_PULMEM	0.696805	0.046452	pulmonary	TRUE	reported	textfile	7	8016602	0.004388	2.42E-17	471167	basophil	TRUE	reported	igd	0.000152	71.85507	2	TRUE
rs7307837	A	C	A	C	-0.02541	0.021978	0.055641	0.065834	FALSE	FALSE	FALSE	I9_PULMEM	0.472208	0.030572	pulmonary	TRUE	reported	textfile	1	2.06E+08	0.004578	2.94E-08	473055	basophil	TRUE	reported	igd	6.51E-05	30.79534	2	TRUE
rs7311011	G	A	G	A	-0.03741	0.045784	0.026813	0.029045	FALSE	FALSE	FALSE	I9_PULMEM	0.315756	0.045637	pulmonary	TRUE	reported	textfile	20	31267270	0.006689	2.29E-08	473999	basophil	TRUE	reported	igd	6.60E-05	31.27721	2	TRUE
rs7398760	A	G	A	G	0.024035	-0.00592	0.166369	0.134327	FALSE	FALSE	FALSE	I9_PULMEM	0.787454	0.021974	pulmonary	TRUE	reported	textfile	2	2.14E+08	0.002752	2.64E-18	473061	basophil	TRUE	reported	igd	0.000161	76.27629	2	TRUE
rs7420	T	C	T	C	-0.01315	0.000818	0.53772	0.547783	FALSE	FALSE	FALSE	I9_PULMEM	0.956656	0.015049	pulmonary	TRUE	reported	textfile	17	72765215	0.002061	1.81E-10	471519	basophil	TRUE	reported	igd	8.63E-05	40.7093	2	TRUE
rs742631	T	C	T	C	-0.01662	0.012453	0.28374	0.263234	FALSE	FALSE	FALSE	I9_PULMEM	0.466949	0.017119	pulmonary	TRUE	reported	textfile	11	32077472	0.002297	4.83E-13	471978	basophil	TRUE	reported	igd	0.000111	52.35259	2	TRUE
rs7447289	C	T	C	T	0.102642	0.038077	0.04944	0.034402	FALSE	FALSE	FALSE	I9_PULMEM	0.35756	0.041386	pulmonary	TRUE	reported	textfile	11	72946279	0.004745	########	472600	basophil	TRUE	reported	igd	0.000989	467.9247	2	TRUE
rs7452436	T	A	T	A	0.022709	0.017538	0.070312	0.072714	FALSE	TRUE	FALSE	I9_PULMEM	0.544276	0.028924	pulmonary	TRUE	reported	textfile	7	80249950	0.004011	1.54E-08	473998	basophil	TRUE	reported	igd	6.76E-05	32.05449	2	TRUE
rs7453541	A	G	A	G	0.038458	-0.05032	0.034635	0.01079	FALSE	FALSE	FALSE	I9_PULMEM	0.494804	0.073715	pulmonary	TRUE	reported	textfile	3	1.28E+08	0.005749	2.31E-11	472141	basophil	TRUE	reported	igd	9.48E-05	44.74937	2	TRUE
rs748113	C	T	C	T	-0.0117	-0.00215	0.436133	0.586997	FALSE	FALSE	FALSE	I9_PULMEM	0.889644	0.015477	pulmonary	TRUE	reported	textfile	10	73508791	0.002089	2.19E-08	471976	basophil	TRUE	reported	igd	6.64E-05	31.36308	2	TRUE
rs7503461	T	C	T	C	-0.01342	0.015725	0.318513	0.350284	FALSE	FALSE	FALSE	I9_PULMEM	0.317768	0.01574	pulmonary	TRUE	reported	textfile	17	2883320	0.002193	9.60E-10	473992	basophil	TRUE	reported	igd	7.90E-05	37.44777	2	TRUE
rs7508433	G	C	G	C	0.028439	0.005456	0.069244	0.068378	FALSE	TRUE	FALSE	I9_PULMEM	0.854746	0.029805	pulmonary	TRUE	reported	textfile	12	76981753	0.004091	3.75E-12	470330	basophil	TRUE	reported	igd	0.000103	48.32455	2	TRUE
rs7515985	G	A	G	A	0.013605	-0.02546	0.415798	0.461713	FALSE	FALSE	FALSE	I9_PULMEM	0.090966	0.01506	pulmonary	TRUE	reported	textfile	1	28997021	0.002078	6.05E-11	473997	basophil	TRUE	reported	igd	9.04E-05	42.86513	2	TRUE
rs7573465	T	G	T	G	0.015498	-0.02998	0.556148	0.589725	FALSE	FALSE	FALSE	I9_PULMEM	0.049552	0.015265	pulmonary	TRUE	reported	textfile	2	1.82E+08	0.002064	6.26E-14	473058	basophil	TRUE	reported	igd	0.000119	56.38065	2	TRUE
rs7613595	C	A	C	A	-0.03637	-0.01205	0.217603	0.280587	FALSE	FALSE	FALSE	I9_PULMEM	0.471796	0.01674	pulmonary	TRUE	reported	textfile	3	16908518	0.002483	1.55E-48	473999	basophil	TRUE	reported	igd	0.000452	214.5632	2	TRUE
rs7642728	C	T	C	T	-0.02884	0.0132	0.254032	0.261984	FALSE	FALSE	FALSE	I9_PULMEM	0.443358	0.01722	pulmonary	TRUE	reported	textfile	19	837190	0.002472	2.07E-31	461318	basophil	TRUE	reported	igd	0.000295	136.12	2	TRUE

rs7642810	C	T	C	T	0.081896	-0.10983	0.013391	0.012756	FALSE	FALSE	FALSE	I9_PULMEM	0.108723	0.068471	pulmonary	TRUE	reported	textfile	13	28604007	0.009385	2.78E-18	473999	basophil	TRUE	reported	igd	0.000161	76.14739	2	TRUE
rs7684939	A	G	A	G	-0.01185	0.012636	0.488692	0.478083	FALSE	FALSE	FALSE	I9_PULMEM	0.401832	0.015072	pulmonary	TRUE	reported	textfile	4	55509189	0.002048	7.35E-09	473062	basophil	TRUE	reported	igd	7.08E-05	33.5018	2	TRUE
rs7832357	G	A	G	A	-0.01543	0.008774	0.342269	0.276408	FALSE	FALSE	FALSE	I9_PULMEM	0.60184	0.016816	pulmonary	TRUE	reported	textfile	8	1.27E+08	0.002158	8.90E-13	473062	basophil	TRUE	reported	igd	0.000108	51.1309	2	TRUE
rs7874418	T	C	T	C	-0.13021	0.048568	0.081739	0.147098	FALSE	FALSE	FALSE	I9_PULMEM	0.023088	0.021377	pulmonary	TRUE	reported	textfile	19	33754548	0.003757	########	473058	basophil	TRUE	reported	igd	0.002533	1201.225	2	TRUE
rs7914063	A	G	A	G	-0.02748	-0.06312	0.054158	0.039121	FALSE	FALSE	FALSE	I9_PULMEM	0.102114	0.038613	pulmonary	TRUE	reported	textfile	2	65084123	0.004583	2.09E-09	473063	basophil	TRUE	reported	igd	7.60E-05	35.94227	2	TRUE
rs8019482	G	A	G	A	0.060817	-0.10649	0.010834	0.012864	FALSE	FALSE	FALSE	I9_PULMEM	0.106727	0.066017	pulmonary	TRUE	reported	textfile	1	28109632	0.010479	6.66E-09	471683	basophil	TRUE	reported	igd	7.14E-05	33.68282	2	TRUE
rs8113682	G	T	G	T	-0.02161	-0.00675	0.747423	0.835873	FALSE	FALSE	FALSE	I9_PULMEM	0.739907	0.020324	pulmonary	TRUE	reported	textfile	19	19743730	0.002364	6.41E-20	473995	basophil	TRUE	reported	igd	0.000176	83.57822	2	TRUE
rs8178414	T	C	T	C	0.073906	0.076778	0.012939	0.03349	FALSE	FALSE	FALSE	I9_PULMEM	0.067177	0.041944	pulmonary	TRUE	reported	textfile	17	56345363	0.00909	4.48E-16	473539	basophil	TRUE	reported	igd	0.00014	66.10431	2	TRUE
rs875740	A	C	A	C	-0.01794	-0.02433	0.665329	0.585303	FALSE	FALSE	FALSE	I9_PULMEM	0.109569	0.015207	pulmonary	TRUE	reported	textfile	16	16123048	0.002172	1.52E-16	472916	basophil	TRUE	reported	igd	0.000144	68.25226	2	TRUE
rs905670	A	G	A	G	-0.01431	0.02649	0.350304	0.250314	FALSE	FALSE	FALSE	I9_PULMEM	0.125907	0.017309	pulmonary	TRUE	reported	textfile	6	90958502	0.002142	2.49E-11	473996	basophil	TRUE	reported	igd	9.41E-05	44.61248	2	TRUE
rs915125	T	C	T	C	-0.02925	-0.00632	0.281093	0.306559	FALSE	FALSE	FALSE	I9_PULMEM	0.699393	0.016364	pulmonary	TRUE	reported	textfile	6	82463376	0.002288	2.18E-37	472601	basophil	TRUE	reported	igd	0.000346	163.477	2	TRUE
rs9376098	A	T	A	T	0.023167	0.018631	0.349186	0.364287	FALSE	TRUE	FALSE	I9_PULMEM	0.231689	0.015577	pulmonary	TRUE	reported	textfile	6	1.35E+08	0.002147	4.15E-27	473987	basophil	TRUE	reported	igd	0.000246	116.4324	2	TRUE
rs9819371	T	C	T	C	-0.0301	-0.01507	0.064572	0.051937	FALSE	FALSE	FALSE	I9_PULMEM	0.657712	0.034002	pulmonary	TRUE	reported	textfile	3	1.41E+08	0.00419	7.05E-13	473998	basophil	TRUE	reported	igd	0.000109	51.60635	2	TRUE
rs9928015	T	G	T	G	-0.01499	-0.0063	0.302009	0.361691	FALSE	FALSE	FALSE	I9_PULMEM	0.686757	0.015626	pulmonary	TRUE	reported	textfile	16	57570561	0.002233	2.01E-11	472583	basophil	TRUE	reported	igd	9.53E-05	45.03335	2	TRUE

Supplementary Table 3: Description of SNPs for eosinophil cell counts incorporated after MR-presso deletion

SNP	effect_al	other_all	effect_al	other_all	beta.expc	beta.outc	eaf.expo	eaf.outc	remove	palindrom	ambiguous	id.outcom	pval.outc	se.outcom	outcome	mr_keep.c	pval_orig	data_sour	chr.expos	pos.expos	se.expos	pval.expc	samplesiz	exposure	mr_keep.e	pval_orig	data_sour	R2	F	action	mr_keep
rs1004870	T	C	T	C	0.026184	0.007685	0.588952	0.609373	FALSE	FALSE	FALSE	I9_PULMEM	0.617051	0.015369	pulmonary	TRUE	reported	textfile	1	42370787	0.002014	1.27E-38	474233	eosinophi	TRUE	reported	igd	0.000356	169.0251	2	TRUE
rs1005901	T	G	T	G	-0.02336	0.015962	0.200806	0.157615	FALSE	FALSE	FALSE	I9_PULMEM	0.437776	0.020571	pulmonary	TRUE	reported	textfile	5	1.11E+08	0.00247	3.38E-21	474234	eosinophi	TRUE	reported	igd	0.000189	89.42842	2	TRUE
rs1006268	G	T	G	T	0.024145	0.006621	0.232866	0.208741	FALSE	FALSE	FALSE	I9_PULMEM	0.719495	0.018435	pulmonary	TRUE	reported	textfile	5	10624866	0.002349	9.47E-25	474233	eosinophi	TRUE	reported	igd	0.000223	105.6541	2	TRUE
rs1010035	A	G	A	G	-0.01342	0.010328	0.227209	0.24678	FALSE	FALSE	FALSE	I9_PULMEM	0.553785	0.017443	pulmonary	TRUE	reported	textfile	8	1.31E+08	0.002387	1.91E-08	474235	eosinophi	TRUE	reported	igd	6.67E-05	31.61752	2	TRUE
rs1016567	A	G	A	G	-0.01468	0.022455	0.757518	0.691525	FALSE	FALSE	FALSE	I9_PULMEM	0.166972	0.016248	pulmonary	TRUE	reported	textfile	2	30446568	0.002302	1.90E-10	474236	eosinophi	TRUE	reported	igd	8.57E-05	40.63907	2	TRUE
rs1017423	A	G	A	G	0.013265	-0.02144	0.765591	0.764134	FALSE	FALSE	FALSE	I9_PULMEM	0.226796	0.017741	pulmonary	TRUE	reported	textfile	2	1.92E+08	0.002402	3.41E-08	472144	eosinophi	TRUE	reported	igd	6.46E-05	30.49767	2	TRUE
rs1019571	T	C	T	C	0.02494	0.027028	0.864395	0.888046	FALSE	FALSE	FALSE	I9_PULMEM	0.257089	0.023849	pulmonary	TRUE	reported	textfile	2	1.59E+08	0.002947	2.74E-17	471756	eosinophi	TRUE	reported	igd	0.000152	71.61942	2	TRUE
rs1020829	A	G	A	G	-0.07891	0.017501	0.286689	0.264033	FALSE	FALSE	FALSE	I9_PULMEM	0.304084	0.01703	pulmonary	TRUE	reported	textfile	2	1.03E+08	0.002186	########	473298	eosinophi	TRUE	reported	igd	0.002745	1302.887	2	TRUE
rs1024342	C	T	C	T	0.016938	0.005647	0.29026	0.273657	FALSE	FALSE	FALSE	I9_PULMEM	0.737458	0.016846	pulmonary	TRUE	reported	textfile	7	1.02E+08	0.002182	8.82E-15	474233	eosinophi	TRUE	reported	igd	0.000127	60.25775	2	TRUE
rs1027589	C	T	C	T	-0.01377	-0.01321	0.24411	0.187619	FALSE	FALSE	FALSE	I9_PULMEM	0.491874	0.019222	pulmonary	TRUE	reported	textfile	7	26858395	0.002304	2.32E-09	474234	eosinophi	TRUE	reported	igd	7.53E-05	35.73471	2	TRUE
rs1036332	C	A	C	A	-0.0327	-0.00851	0.737405	0.723302	FALSE	FALSE	FALSE	I9_PULMEM	0.612367	0.016787	pulmonary	TRUE	reported	textfile	1	1.99E+08	0.002257	1.61E-47	474236	eosinophi	TRUE	reported	igd	0.000442	209.8574	2	TRUE
rs1037674	T	G	T	G	0.013204	-0.02004	0.292292	0.270379	FALSE	FALSE	FALSE	I9_PULMEM	0.235606	0.016894	pulmonary	TRUE	reported	textfile	7	50217850	0.002188	1.63E-09	473295	eosinophi	TRUE	reported	igd	7.69E-05	36.41787	2	TRUE
rs1047298	G	C	G	C	-0.03499	0.010328	0.339875	0.395033	FALSE	TRUE	FALSE	I9_PULMEM	0.501642	0.015372	pulmonary	TRUE	reported	textfile	5	35843832	0.00209	7.62E-63	474235	eosinophi	TRUE	reported	igd	0.000591	280.2008	2	TRUE
rs1047891	A	C	A	C	0.016967	0.038443	0.315457	0.321403	FALSE	FALSE	FALSE	I9_PULMEM	0.017165	0.016131	pulmonary	TRUE	reported	textfile	2	2.12E+08	0.002127	1.55E-15	473297	eosinophi	TRUE	reported	igd	0.000134	63.63167	2	TRUE
rs1057258	T	C	T	C	-0.03213	0.027151	0.178094	0.140894	FALSE	FALSE	FALSE	I9_PULMEM	0.210237	0.02167	pulmonary	TRUE	reported	textfile	2	2.34E+08	0.002589	2.52E-35	472835	eosinophi	TRUE	reported	igd	0.000326	153.974	2	TRUE
rs1059091	G	A	G	A	0.033841	-0.03218	0.321344	0.321742	FALSE	FALSE	FALSE	I9_PULMEM	0.045115	0.016059	pulmonary	TRUE	reported	textfile	11	309127	0.002141	3.08E-56	467918	eosinophi	TRUE	reported	igd	0.000534	249.8338	2	TRUE
rs1077737	A	G	A	G	-0.02249	-0.00484	0.535724	0.553501	FALSE	FALSE	FALSE	I9_PULMEM	0.748537	0.015104	pulmonary	TRUE	reported	textfile	12	92518680	0.001989	1.28E-29	473151	eosinophi	TRUE	reported	igd	0.00027	127.8293	2	TRUE
rs1078295	T	C	T	C	0.022538	0.002817	0.621606	0.642821	FALSE	FALSE	FALSE	I9_PULMEM	0.857151	0.015651	pulmonary	TRUE	reported	textfile	1	93763896	0.002042	2.81E-28	471257	eosinophi	TRUE	reported	igd	0.000258	121.8197	2	TRUE
rs1087655	A	G	A	G	0.013811	-0.01645	0.559198	0.677572	FALSE	FALSE	FALSE	I9_PULMEM	0.309296	0.016182	pulmonary	TRUE	reported	textfile	12	54712308	0.001998	4.92E-12	471745	eosinophi	TRUE	reported	igd	0.000101	47.78124	2	TRUE
rs1090059	A	C	A	C	0.015482	-0.00292	0.713282	0.797943	FALSE	FALSE	FALSE	I9_PULMEM	0.875333	0.018639	pulmonary	TRUE	reported	textfile	1	2.05E+08	0.002189	1.59E-12	473153	eosinophi	TRUE	reported	igd	0.000106	50.02197	2	TRUE
rs1093033	T	C	T	C	0.01471	-0.00635	0.284759	0.316815	FALSE	FALSE	FALSE	I9_PULMEM	0.693643	0.016108	pulmonary	TRUE	reported	textfile	2	1.7E+08	0.002192	2.03E-11	473292	eosinophi	TRUE	reported	igd	9.51E-05	45.0342	2	TRUE
rs1099448	T	C	T	C	0.017702	-0.01212	0.466357	0.430638	FALSE	FALSE	FALSE	I9_PULMEM	0.423191	0.015137	pulmonary	TRUE	reported	textfile	1	1.73E+08	0.001998	8.32E-19	474225	eosinophi	TRUE	reported	igd	0.000165	78.49679	2	TRUE
rs1099524	C	G	C	G	-0.04384	0.019096	0.368331	0.326775	FALSE	TRUE	FALSE	I9_PULMEM	0.234476	0.016062	pulmonary	TRUE	reported	textfile	10	64388631	0.002049	########	474230	eosinophi	TRUE	reported	igd	0.000964	457.7367	2	TRUE
rs1102464	T	C	T	C	0.017085	-0.02384	0.218739	0.247155	FALSE	FALSE	FALSE	I9_PULMEM	0.171489	0.017434	pulmonary	TRUE	reported	textfile	11	2955929	0.002411	1.45E-12	474234	eosinophi	TRUE	reported	igd	0.000106	50.21503	2	TRUE
rs1107155	T	C	T	C	-0.0222	-0.00478	0.13051	0.154655	FALSE	FALSE	FALSE	I9_PULMEM	0.817636	0.020751	pulmonary	TRUE	reported	textfile	15	61069988	0.002945	4.94E-14	474231	eosinophi	TRUE	reported	igd	0.00012	56.82422	2	TRUE
rs1107934	C	T	C	T	0.012271	-0.02509	0.321638	0.410219	FALSE	FALSE	FALSE	I9_PULMEM	0.100705	0.015285	pulmonary	TRUE	reported	textfile	17	56271060	0.002121	7.49E-09	474231	eosinophi	TRUE	reported	igd	7.06E-05	33.47162	2	TRUE
rs1108823	T	C	T	C	0.018316	-0.0242	0.453012	0.525637	FALSE	FALSE	FALSE	I9_PULMEM	0.106789	0.015006	pulmonary	TRUE	reported	textfile	21	34416187	0.001998	5.17E-20	474235	eosinophi	TRUE	reported	igd	0.000177	84.0366	2	TRUE
rs1112004	T	C	T	C	0.011114	0.009313	0.608251	0.654348	FALSE	FALSE	FALSE	I9_PULMEM	0.554798	0.015769	pulmonary	TRUE	reported	textfile	1	2.13E+08	0.002036	4.93E-08	473294	eosinophi	TRUE	reported	igd	6.30E-05	29.79775	2	TRUE
rs1117593	T	C	T	C	-0.04302	-0.02017	0.110759	0.072937	FALSE	FALSE	FALSE	I9_PULMEM	0.483029	0.028756	pulmonary	TRUE	reported	textfile	1	1.02E+08	0.00316	3.56E-42	474226	eosinophi	TRUE	reported	igd	0.000391	185.3726	2	TRUE
rs1120275	G	T	G	T	-0.02389	0.023812	0.3108	0.393691	FALSE	FALSE	FALSE	I9_PULMEM	0.120783	0.015348	pulmonary	TRUE	reported	textfile	16	48598737	0.002142	7.53E-29	473153	eosinophi	TRUE	reported	igd	0.000263	124.423	2	TRUE
rs1120468	T	G	T	G	-0.04133	0.05813	0.223016	0.20018	FALSE	FALSE	FALSE	I9_PULMEM	0.001784	0.018608	pulmonary	TRUE	reported	textfile	1	1.51E+08	0.002388	4.89E-67	473291	eosinophi	TRUE	reported	igd	0.000633	299.5583	2	TRUE
rs1122554	A	G	A	G	-0.04621	0.021226	0.016576	0.039172	FALSE	FALSE	FALSE	I9_PULMEM	0.582709	0.038633	pulmonary	TRUE	reported	textfile	3	1.28E+08	0.007975	7.05E-09	473296	eosinophi	TRUE	reported	igd	7.09E-05	33.57589	2	TRUE
rs1122899	T	A	T	A	0.02926	-0.02359	0.044508	0.044615	FALSE	TRUE	FALSE	I9_PULMEM	0.520704	0.036731	pulmonary	TRUE	reported	textfile	11	57071786	0.004953	3.56E-09	474235	eosinophi	TRUE	reported	igd	7.36E-05	34.89877	2	TRUE
rs1123681	C	G	C	G	-0.03034	-0.01704	0.101862	0.116621	FALSE	TRUE	FALSE	I9_PULMEM	0.468048	0.023482	pulmonary	TRUE	reported	textfile	11	76343427	0.003306	4.78E-20	474224	eosinophi	TRUE	reported	igd	0.000178	84.20497	2	TRUE
rs1125550	G	T	G	T	0.020196	-0.03752	0.17824	0.220276	FALSE	FALSE	FALSE	I9_PULMEM	0.039384	0.018213	pulmonary	TRUE	reported	textfile	10	8109615	0.00261	1.05E-14	474236	eosinophi	TRUE	reported	igd	0.000126	59.87532	2	TRUE
rs1134736	G	A	G	A	-0.06322	0.012298	0.026223	0.014192	FALSE	FALSE	FALSE	I9_PULMEM	0.847304	0.063866	pulmonary	TRUE	reported	textfile	4	1.03E+08	0.006522	3.47E-22	474233	eosinophi	TRUE	reported	igd	0.000198	93.95164	2	TRUE
rs1134966	A	G	A	G	-0.04485	0.084171	0.030304	0.017491	FALSE	FALSE	FALSE	I9_PULMEM	0.139725	0.056995	pulmonary	TRUE	reported	textfile	6	1.38E+08	0.005802	1.13E-14	474236	eosinophi	TRUE	reported	igd	0.000126	59.74609	2	TRUE
rs1135423	A	G	A	G	-0.02686	0.039933	0.075059	0.034216	FALSE	FALSE	FALSE	I9_PULMEM	0.334412	0.04137	pulmonary	TRUE	reported	textfile	2	43464818	0.003797	1.55E-12	472359	eosinophi	TRUE	reported	igd	0.000106	50.0488	2	TRUE
rs1138594	A	G	A	G	-0.02935	0.047672	0.039661	0.052228	FALSE	FALSE	FALSE	I9_PULMEM	0.164482	0.034293	pulmonary	TRUE	reported	textfile	14	94422198	0.00537	4.75E-08	474234	eosinophi	TRUE	reported	igd	6.30E-05	29.86193	2	TRUE
rs1141527	A	G	A	G	-0.03625	-0.03959	0.030153	0.03895	FALSE	FALSE	FALSE	I9_PULMEM	0.307227	0.038777	pulmonary	TRUE	reported	textfile	21	36434894	0.005897	8.08E-10	474237	eosinophi	TRUE	reported	igd	7.97E-05	37.792	2	TRUE
rs1147415	C	T	C	T	0.119573	0.039309	0.007538	0.00385	FALSE	FALSE	FALSE	I9_PULMEM	0.742532	0.119662	pulmonary	TRUE	reported	textfile	9	1.36E+08	0.011508	2.94E-25	473801	eosinophi	TRUE	reported	igd	0.000228	107.9605	2	TRUE

rs1149586	A	G	A	G	0.028355	0.004406	0.086693	0.058338	FALSE	FALSE	FALSE	I9_PULMEM	0.892403	0.032572	pulmonary	TRUE	reported	textfile	2	2.43E+08	0.003673	1.21E-14	435972	eosinophi	TRUE	reported	igd	0.000137	59.59577	2	TRUE
rs1155554	C	T	C	T	0.06384	0.029959	0.062749	0.01752	FALSE	FALSE	FALSE	I9_PULMEM	0.611277	0.058946	pulmonary	TRUE	reported	textfile	14	94417531	0.004108	2.12E-54	472837	eosinophi	TRUE	reported	igd	0.00051	241.5033	2	TRUE
rs1156476	A	G	A	G	-0.06727	-0.02267	0.026499	0.020561	FALSE	FALSE	FALSE	I9_PULMEM	0.671594	0.053467	pulmonary	TRUE	reported	textfile	12	1.12E+08	0.006524	6.69E-25	474234	eosinophi	TRUE	reported	igd	0.000224	106.329	2	TRUE
rs1156974	C	T	C	T	-0.06506	-0.06417	0.015017	0.014122	FALSE	FALSE	FALSE	I9_PULMEM	0.319446	0.064449	pulmonary	TRUE	reported	textfile	3	1.28E+08	0.008158	1.61E-15	474234	eosinophi	TRUE	reported	igd	0.000134	63.59048	2	TRUE
rs1162856	G	A	G	A	-0.01659	-0.00751	0.289982	0.362056	FALSE	FALSE	FALSE	I9_PULMEM	0.63186	0.015685	pulmonary	TRUE	reported	textfile	14	1.03E+08	0.002198	4.70E-14	474231	eosinophi	TRUE	reported	igd	0.00012	56.95487	2	TRUE
rs1164829	A	C	A	C	-0.01304	-0.01467	0.693342	0.688143	FALSE	FALSE	FALSE	I9_PULMEM	0.365724	0.016216	pulmonary	TRUE	reported	textfile	16	4462897	0.002158	1.59E-09	474235	eosinophi	TRUE	reported	igd	7.69E-05	36.49085	2	TRUE
rs1168477	G	A	G	A	-0.01477	0.009845	0.270352	0.277781	FALSE	FALSE	FALSE	I9_PULMEM	0.55573	0.016709	pulmonary	TRUE	reported	textfile	2	71281496	0.002222	3.10E-11	474195	eosinophi	TRUE	reported	igd	9.31E-05	44.17265	2	TRUE
rs1168888	A	G	A	G	-0.05774	-0.17946	0.009792	0.004017	FALSE	FALSE	FALSE	I9_PULMEM	0.127985	0.117902	pulmonary	TRUE	reported	textfile	7	92633347	0.010199	1.54E-08	472402	eosinophi	TRUE	reported	igd	6.78E-05	32.05174	2	TRUE
rs1169528	T	C	T	C	-0.02298	0.005228	0.514407	0.457418	FALSE	FALSE	FALSE	I9_PULMEM	0.728175	0.015041	pulmonary	TRUE	reported	textfile	2	28686568	0.00198	4.09E-31	473295	eosinophi	TRUE	reported	igd	0.000284	134.6649	2	TRUE
rs1170147	C	T	C	T	0.014907	-0.03456	0.22262	0.252756	FALSE	FALSE	FALSE	I9_PULMEM	0.047327	0.017424	pulmonary	TRUE	reported	textfile	21	16931347	0.002442	1.06E-09	474235	eosinophi	TRUE	reported	igd	7.86E-05	37.26382	2	TRUE
rs1170291	T	C	T	C	-0.02232	0.01029	0.102562	0.093963	FALSE	FALSE	FALSE	I9_PULMEM	0.689217	0.025729	pulmonary	TRUE	reported	textfile	22	17594623	0.00328	1.06E-11	474232	eosinophi	TRUE	reported	igd	9.76E-05	46.28957	2	TRUE
rs1170439	C	T	C	T	0.022511	-0.0139	0.779264	0.823128	FALSE	FALSE	FALSE	I9_PULMEM	0.480098	0.019689	pulmonary	TRUE	reported	textfile	16	68608511	0.002403	7.86E-21	473155	eosinophi	TRUE	reported	igd	0.000185	87.75668	2	TRUE
rs1170685	T	C	T	C	-0.02609	0.031877	0.190092	0.141839	FALSE	FALSE	FALSE	I9_PULMEM	0.139816	0.02159	pulmonary	TRUE	reported	textfile	14	93118229	0.002543	1.17E-24	473026	eosinophi	TRUE	reported	igd	0.000222	105.2657	2	TRUE
rs1174816	C	T	C	T	0.035585	-0.031	0.027904	0.022244	FALSE	FALSE	FALSE	I9_PULMEM	0.536189	0.050111	pulmonary	TRUE	reported	textfile	19	40192953	0.00617	8.25E-09	473297	eosinophi	TRUE	reported	igd	7.03E-05	33.26303	2	TRUE
rs1175773	A	G	A	G	0.017343	0.006781	0.14402	0.05936	FALSE	FALSE	FALSE	I9_PULMEM	0.833197	0.032198	pulmonary	TRUE	reported	textfile	17	2836247	0.002915	2.75E-09	474237	eosinophi	TRUE	reported	igd	7.46E-05	35.39725	2	TRUE
rs1178016	T	C	T	C	0.011342	-0.00321	0.495413	0.526531	FALSE	FALSE	FALSE	I9_PULMEM	0.831138	0.015047	pulmonary	TRUE	reported	textfile	20	2996497	0.001987	1.17E-08	473295	eosinophi	TRUE	reported	igd	6.88E-05	32.5823	2	TRUE
rs1178653	A	G	A	G	-0.02675	0.015408	0.164896	0.180312	FALSE	FALSE	FALSE	I9_PULMEM	0.430855	0.01956	pulmonary	TRUE	reported	textfile	8	1.29E+08	0.002705	5.11E-23	471885	eosinophi	TRUE	reported	igd	0.000207	97.76453	2	TRUE
rs1179555	T	G	T	G	-0.03039	0.029407	0.256399	0.295385	FALSE	FALSE	FALSE	I9_PULMEM	0.07399	0.016459	pulmonary	TRUE	reported	textfile	19	45755368	0.002277	1.44E-40	474228	eosinophi	TRUE	reported	igd	0.000375	178.0699	2	TRUE
rs1179615	A	G	A	G	-0.03098	-0.07411	0.039583	0.031372	FALSE	FALSE	FALSE	I9_PULMEM	0.09092	0.043839	pulmonary	TRUE	reported	textfile	8	1.45E+08	0.0052	2.63E-09	468999	eosinophi	TRUE	reported	igd	7.57E-05	35.49395	2	TRUE
rs1180134	A	G	A	G	-0.0336	-0.04973	0.06696	0.099362	FALSE	FALSE	FALSE	I9_PULMEM	0.049417	0.02531	pulmonary	TRUE	reported	textfile	19	33726577	0.004068	1.54E-16	474235	eosinophi	TRUE	reported	igd	0.000144	68.22861	2	TRUE
rs1186922	T	C	T	C	0.020575	-0.03425	0.840958	0.844922	FALSE	FALSE	FALSE	I9_PULMEM	0.098733	0.020744	pulmonary	TRUE	reported	textfile	17	57800358	0.002712	3.45E-14	472838	eosinophi	TRUE	reported	igd	0.000122	57.55704	2	TRUE
rs1188692	G	T	G	T	-0.01439	0.008767	0.30847	0.232031	FALSE	FALSE	FALSE	I9_PULMEM	0.625432	0.017959	pulmonary	TRUE	reported	textfile	2	74258529	0.00214	1.86E-11	474231	eosinophi	TRUE	reported	igd	9.53E-05	45.19716	2	TRUE
rs1188832	G	A	G	A	-0.01729	-0.00757	0.778072	0.760424	FALSE	FALSE	FALSE	I9_PULMEM	0.667142	0.017599	pulmonary	TRUE	reported	textfile	2	98577882	0.002383	4.18E-13	473154	eosinophi	TRUE	reported	igd	0.000111	52.64293	2	TRUE
rs1189030	T	C	T	C	0.012913	0.023751	0.423414	0.393745	FALSE	FALSE	FALSE	I9_PULMEM	0.123987	0.015441	pulmonary	TRUE	reported	textfile	2	28691488	0.002026	1.91E-10	472208	eosinophi	TRUE	reported	igd	8.60E-05	40.62315	2	TRUE
rs1193171	T	C	T	C	0.019253	-0.01668	0.282445	0.335487	FALSE	FALSE	FALSE	I9_PULMEM	0.293637	0.015884	pulmonary	TRUE	reported	textfile	4	1.53E+08	0.002213	3.48E-18	473297	eosinophi	TRUE	reported	igd	0.00016	75.68889	2	TRUE
rs1210003	A	G	A	G	-0.01705	0.004081	0.359122	0.334779	FALSE	FALSE	FALSE	I9_PULMEM	0.797774	0.015928	pulmonary	TRUE	reported	textfile	13	1.15E+08	0.002079	2.60E-16	473293	eosinophi	TRUE	reported	igd	0.000142	67.21763	2	TRUE
rs1215449	C	A	C	A	-0.03254	-0.0013	0.854434	0.814426	FALSE	FALSE	FALSE	I9_PULMEM	0.946466	0.019348	pulmonary	TRUE	reported	textfile	7	92223518	0.002817	7.76E-31	474232	eosinophi	TRUE	reported	igd	0.000281	133.4483	2	TRUE
rs121564	T	G	T	G	-0.01656	-0.01005	0.270023	0.239679	FALSE	FALSE	FALSE	I9_PULMEM	0.568949	0.017638	pulmonary	TRUE	reported	textfile	16	20717360	0.002233	1.26E-13	474234	eosinophi	TRUE	reported	igd	0.000116	55.02387	2	TRUE
rs1220810	T	C	T	C	-0.03074	-0.00841	0.378399	0.407353	FALSE	FALSE	FALSE	I9_PULMEM	0.585523	0.015425	pulmonary	TRUE	reported	textfile	6	1.07E+08	0.002069	6.83E-50	473100	eosinophi	TRUE	reported	igd	0.000466	220.6845	2	TRUE
rs1240893	A	G	A	G	-0.04132	-0.03679	0.10259	0.114496	FALSE	FALSE	FALSE	I9_PULMEM	0.11762	0.023514	pulmonary	TRUE	reported	textfile	1	65423447	0.00328	2.36E-36	472800	eosinophi	TRUE	reported	igd	0.000336	158.6902	2	TRUE
rs1248798	A	C	A	C	0.012539	-0.00038	0.633844	0.724322	FALSE	FALSE	FALSE	I9_PULMEM	0.981766	0.016792	pulmonary	TRUE	reported	textfile	3	71760041	0.002073	1.50E-09	473155	eosinophi	TRUE	reported	igd	7.73E-05	36.58688	2	TRUE
rs1251518	T	C	T	C	-0.0721	-0.039	0.425948	0.317764	FALSE	FALSE	FALSE	I9_PULMEM	0.015607	0.016129	pulmonary	TRUE	reported	textfile	5	1.32E+08	0.002004	########	473133	eosinophi	TRUE	reported	igd	0.002728	1294.342	2	TRUE
rs1253094	G	A	G	A	0.042755	-0.01978	0.613323	0.675952	FALSE	FALSE	FALSE	I9_PULMEM	0.218096	0.016057	pulmonary	TRUE	reported	textfile	7	1.49E+08	0.002044	3.99E-97	473297	eosinophi	TRUE	reported	igd	0.000924	437.5324	2	TRUE
rs1254028	G	A	G	A	0.014646	-0.01184	0.22062	0.307053	FALSE	FALSE	FALSE	I9_PULMEM	0.466897	0.016278	pulmonary	TRUE	reported	textfile	7	1.5E+08	0.002396	1.01E-09	472826	eosinophi	TRUE	reported	igd	7.90E-05	37.36479	2	TRUE
rs1254573	C	T	C	T	-0.03073	0.009902	0.703401	0.69742	FALSE	FALSE	FALSE	I9_PULMEM	0.54526	0.016371	pulmonary	TRUE	reported	textfile	8	22957003	0.002188	8.90E-45	472824	eosinophi	TRUE	reported	igd	0.000417	197.2938	2	TRUE
rs1257192	G	A	G	A	0.014595	-0.00762	0.812097	0.84066	FALSE	FALSE	FALSE	I9_PULMEM	0.70922	0.020442	pulmonary	TRUE	reported	textfile	2	1.35E+08	0.002535	8.81E-09	471755	eosinophi	TRUE	reported	igd	7.03E-05	33.14747	2	TRUE
rs1258151	G	C	G	C	-0.01764	0.024991	0.173045	0.204909	FALSE	TRUE	FALSE	I9_PULMEM	0.181963	0.018723	pulmonary	TRUE	reported	textfile	12	46573788	0.002631	2.07E-11	474236	eosinophi	TRUE	reported	igd	9.48E-05	44.96777	2	TRUE
rs1270584	A	G	A	G	-0.02058	0.003814	0.406896	0.365113	FALSE	FALSE	FALSE	I9_PULMEM	0.80752	0.015655	pulmonary	TRUE	reported	textfile	7	1.13E+08	0.002021	2.55E-24	474233	eosinophi	TRUE	reported	igd	0.000219	103.6644	2	TRUE
rs1282086	T	C	T	C	0.022466	0.032241	0.351578	0.362467	FALSE	FALSE	FALSE	I9_PULMEM	0.041042	0.01578	pulmonary	TRUE	reported	textfile	12	4318723	0.002142	1.07E-25	471756	eosinophi	TRUE	reported	igd	0.000233	110.0046	2	TRUE
rs1282798	G	A	G	A	-0.01585	0.020789	0.456703	0.463452	FALSE	FALSE	FALSE	I9_PULMEM	0.166653	0.015031	pulmonary	TRUE	reported	textfile	12	9130287	0.001987	1.57E-15	474184	eosinophi	TRUE	reported	igd	0.000134	63.65395	2	TRUE
rs1286182	C	T	C	T	-0.02098	-0.0162	0.275439	0.288206	FALSE	FALSE	FALSE	I9_PULMEM	0.342666	0.017071	pulmonary	TRUE	reported	textfile	13	31079184	0.002402	2.59E-18	473149	eosinophi	TRUE	reported	igd	0.000161	76.2674	2	TRUE
rs1287861	C	T	C	T	-0.01318	-0.01427	0.475864	0.49768	FALSE	FALSE	FALSE	I9_PULMEM	0.342269	0.015022	pulmonary	TRUE	reported	textfile	14	73389048	0.001994	3.95E-11	474230	eosinophi	TRUE	reported	igd	9.22E-05	43.70955	2	TRUE
rs1288228	C	T	C	T	-0.0136	0.016412	0.238599	0.204734	FALSE	FALSE	FALSE	I9_PULMEM	0.376419	0.018555	pulmonary	TRUE	reported	textfile	14	21431304	0.00235	7.37E-09	474233	eosinophi	TRUE	reported	igd	7.06E-05	33.49194	2	TRUE
rs1292850	T	C	T	C	-0.049	0.080891	0.019564	0.004428	FALSE	FALSE	FALSE	I9_PULMEM	0.485666	0.11602	pulmonary	TRUE	reported	textfile	16	67377216	0.007294	1.92E-11	466479	eosinophi	TRUE	reported	igd	9.67E-05	45.12011	2	TRUE

rs1294106	A	G	A	G	-0.01391	-0.03793	0.285851	0.216033	FALSE	FALSE	FALSE	I9_PULMEM	0.038463	0.018322	pulmonary	TRUE	reported	textfile	17	17737857	0.002272	9.59E-10	472214	eosinophi	TRUE	reported	igd	7.93E-05	37.45618	2	TRUE
rs1303333	G	C	G	C	0.012393	0.005862	0.32279	0.366953	FALSE	TRUE	FALSE	I9_PULMEM	0.70677	0.015581	pulmonary	TRUE	reported	textfile	2	1.06E+08	0.002113	4.62E-09	473296	eosinophi	TRUE	reported	igd	7.27E-05	34.3995	2	TRUE
rs1307368	C	T	C	T	0.015579	0.008905	0.399148	0.504537	FALSE	FALSE	FALSE	I9_PULMEM	0.552949	0.015008	pulmonary	TRUE	reported	textfile	3	25393837	0.002021	1.32E-14	474234	eosinophi	TRUE	reported	igd	0.000125	59.42165	2	TRUE
rs1310568	G	T	G	T	-0.02661	-0.04253	0.059685	0.00937	FALSE	FALSE	FALSE	I9_PULMEM	0.593697	0.079721	pulmonary	TRUE	reported	textfile	4	1.03E+08	0.00433	8.22E-10	474236	eosinophi	TRUE	reported	igd	7.96E-05	37.76981	2	TRUE
rs1312037	G	A	G	A	0.015459	-0.03094	0.327238	0.350435	FALSE	FALSE	FALSE	I9_PULMEM	0.048752	0.015701	pulmonary	TRUE	reported	textfile	4	1.39E+08	0.002118	3.00E-13	473296	eosinophi	TRUE	reported	igd	0.000113	53.27323	2	TRUE
rs1313835	T	C	T	C	-0.05482	0.011096	0.185539	0.233382	FALSE	FALSE	FALSE	I9_PULMEM	0.531152	0.017717	pulmonary	TRUE	reported	textfile	4	83545976	0.002551	########	474230	eosinophi	TRUE	reported	igd	0.000973	461.852	2	TRUE
rs1313994	G	A	G	A	-0.01384	0.012818	0.802896	0.788978	FALSE	FALSE	FALSE	I9_PULMEM	0.485808	0.018391	pulmonary	TRUE	reported	textfile	4	7071741	0.002491	2.84E-08	473156	eosinophi	TRUE	reported	igd	6.52E-05	30.86902	2	TRUE
rs1320779	G	A	G	A	0.025659	0.071187	0.088551	0.026931	FALSE	FALSE	FALSE	I9_PULMEM	0.12563	0.04648	pulmonary	TRUE	reported	textfile	6	1.43E+08	0.003539	4.30E-13	473156	eosinophi	TRUE	reported	igd	0.000111	52.5674	2	TRUE
rs1322658	T	A	T	A	-0.06187	-0.00796	0.115701	0.132768	FALSE	TRUE	FALSE	I9_PULMEM	0.718707	0.022086	pulmonary	TRUE	reported	textfile	7	75454152	0.0031	1.59E-88	474234	eosinophi	TRUE	reported	igd	0.000839	398.2583	2	TRUE
rs1323650	T	G	T	G	-0.01361	-0.00531	0.680787	0.702962	FALSE	FALSE	FALSE	I9_PULMEM	0.74798	0.01652	pulmonary	TRUE	reported	textfile	10	6033049	0.002151	2.53E-10	470486	eosinophi	TRUE	reported	igd	8.51E-05	40.05796	2	TRUE
rs1325164	T	A	T	A	0.030412	-0.00446	0.044409	0.05016	FALSE	TRUE	FALSE	I9_PULMEM	0.897378	0.034559	pulmonary	TRUE	reported	textfile	8	1.26E+08	0.004844	3.54E-10	474236	eosinophi	TRUE	reported	igd	8.31E-05	39.41666	2	TRUE
rs13511	C	T	C	T	-0.01636	-0.01236	0.57742	0.512836	FALSE	FALSE	FALSE	I9_PULMEM	0.41128	0.01504	pulmonary	TRUE	reported	textfile	15	86284817	0.002009	4.13E-16	474215	eosinophi	TRUE	reported	igd	0.00014	66.27342	2	TRUE
rs1353286	G	T	G	T	0.020226	0.008959	0.453022	0.47541	FALSE	FALSE	FALSE	I9_PULMEM	0.55206	0.015066	pulmonary	TRUE	reported	textfile	3	27772014	0.00199	3.09E-24	474234	eosinophi	TRUE	reported	igd	0.000218	103.3028	2	TRUE
rs1365623	T	C	T	C	0.011627	0.003413	0.37246	0.363148	FALSE	FALSE	FALSE	I9_PULMEM	0.827054	0.01562	pulmonary	TRUE	reported	textfile	4	1.75E+08	0.00206	1.71E-08	474235	eosinophi	TRUE	reported	igd	6.72E-05	31.85657	2	TRUE
rs1396406	G	A	G	A	0.024854	0.028153	0.092902	0.094614	FALSE	FALSE	FALSE	I9_PULMEM	0.270737	0.025562	pulmonary	TRUE	reported	textfile	5	1.1E+08	0.003415	3.54E-13	474234	eosinophi	TRUE	reported	igd	0.000112	52.96748	2	TRUE
rs1404112	A	G	A	G	0.030273	-0.00862	0.05614	0.054968	FALSE	FALSE	FALSE	I9_PULMEM	0.798128	0.033688	pulmonary	TRUE	reported	textfile	19	16228192	0.004681	1.03E-10	474223	eosinophi	TRUE	reported	igd	8.82E-05	41.8246	2	TRUE
rs1405098	A	G	A	G	0.099069	-0.09788	0.008506	0.004586	FALSE	FALSE	FALSE	I9_PULMEM	0.375555	0.110461	pulmonary	TRUE	reported	textfile	2	1.12E+08	0.012308	8.74E-16	471439	eosinophi	TRUE	reported	igd	0.000137	64.78862	2	TRUE
rs1406449	C	T	C	T	-0.01219	-0.02975	0.461666	0.499795	FALSE	FALSE	FALSE	I9_PULMEM	0.047116	0.014984	pulmonary	TRUE	reported	textfile	2	1.48E+08	0.001986	8.69E-10	472834	eosinophi	TRUE	reported	igd	7.96E-05	37.6621	2	TRUE
rs1414517	G	C	G	C	-0.02498	-0.01493	0.187298	0.165251	FALSE	TRUE	FALSE	I9_PULMEM	0.459544	0.020191	pulmonary	TRUE	reported	textfile	1	2.21E+08	0.002552	1.36E-22	474233	eosinophi	TRUE	reported	igd	0.000202	95.78938	2	TRUE
rs1427499	G	A	G	A	0.018666	0.016509	0.710269	0.693926	FALSE	FALSE	FALSE	I9_PULMEM	0.310753	0.016287	pulmonary	TRUE	reported	textfile	2	1.45E+08	0.002183	1.30E-17	470981	eosinophi	TRUE	reported	igd	0.000155	73.11276	2	TRUE
rs1434917	G	C	G	C	-0.03128	-0.00815	0.035786	0.015964	FALSE	TRUE	FALSE	I9_PULMEM	0.892259	0.060154	pulmonary	TRUE	reported	textfile	9	1.35E+08	0.005517	1.47E-08	474236	eosinophi	TRUE	reported	igd	6.78E-05	32.14591	2	TRUE
rs1458233	A	G	A	G	0.060811	-0.158	0.010365	0.010332	FALSE	FALSE	FALSE	I9_PULMEM	0.031451	0.073442	pulmonary	TRUE	reported	textfile	22	37353961	0.010312	3.81E-09	471917	eosinophi	TRUE	reported	igd	7.37E-05	34.77576	2	TRUE
rs1459478	C	A	C	A	-0.05993	-0.0636	0.0263	0.030241	FALSE	FALSE	FALSE	I9_PULMEM	0.145804	0.043724	pulmonary	TRUE	reported	textfile	17	41809207	0.006329	2.98E-21	474179	eosinophi	TRUE	reported	igd	0.000189	89.67265	2	TRUE
rs1460781	A	G	A	G	0.085887	0.33146	0.009579	0.002485	FALSE	FALSE	FALSE	I9_PULMEM	0.029464	0.15224	pulmonary	TRUE	reported	textfile	1	1.51E+08	0.010438	2.00E-16	473763	eosinophi	TRUE	reported	igd	0.000143	67.70464	2	TRUE
rs14713	A	G	A	G	-0.01359	-0.00991	0.20454	0.22912	FALSE	FALSE	FALSE	I9_PULMEM	0.580841	0.017948	pulmonary	TRUE	reported	textfile	19	2714666	0.002464	3.53E-08	473295	eosinophi	TRUE	reported	igd	6.43E-05	30.43766	2	TRUE
rs1471816	A	G	A	G	0.010911	-0.00638	0.503325	0.482635	FALSE	FALSE	FALSE	I9_PULMEM	0.670474	0.014997	pulmonary	TRUE	reported	textfile	12	94145120	0.001985	3.94E-08	474233	eosinophi	TRUE	reported	igd	6.37E-05	30.21386	2	TRUE
rs1479918	T	A	T	A	-0.01878	0.017113	0.259813	0.14034	FALSE	TRUE	FALSE	I9_PULMEM	0.427845	0.021584	pulmonary	TRUE	reported	textfile	4	1.23E+08	0.002261	1.05E-16	474236	eosinophi	TRUE	reported	igd	0.000145	68.96094	2	TRUE
rs1491105	T	C	T	C	0.038205	0.004169	0.035905	0.025923	FALSE	FALSE	FALSE	I9_PULMEM	0.929948	0.04742	pulmonary	TRUE	reported	textfile	6	1.44E+08	0.005388	1.39E-12	474233	eosinophi	TRUE	reported	igd	0.000106	50.27863	2	TRUE
rs1506400	T	G	T	G	0.099543	0.050086	0.016244	0.015484	FALSE	FALSE	FALSE	I9_PULMEM	0.414643	0.061398	pulmonary	TRUE	reported	textfile	7	50444152	0.008004	1.80E-35	473776	eosinophi	TRUE	reported	igd	0.000326	154.6698	2	TRUE
rs1516527	C	T	C	T	-0.03228	-0.00824	0.951148	0.971547	FALSE	FALSE	FALSE	I9_PULMEM	0.854699	0.04497	pulmonary	TRUE	reported	textfile	3	1.49E+08	0.004596	2.25E-12	474237	eosinophi	TRUE	reported	igd	0.000104	49.3171	2	TRUE
rs152197	T	C	T	C	0.049978	0.020816	0.175499	0.226479	FALSE	FALSE	FALSE	I9_PULMEM	0.245501	0.017924	pulmonary	TRUE	reported	textfile	5	1.31E+08	0.002605	5.48E-82	474236	eosinophi	TRUE	reported	igd	0.000776	368.0785	2	TRUE
rs1539174	G	C	G	C	0.038017	-0.00859	0.23738	0.204573	FALSE	TRUE	FALSE	I9_PULMEM	0.6441	0.018602	pulmonary	TRUE	reported	textfile	10	974870	0.002332	1.12E-59	474232	eosinophi	TRUE	reported	igd	0.00056	265.7644	2	TRUE
rs159963	A	C	A	C	0.01933	0.000625	0.581974	0.656152	FALSE	FALSE	FALSE	I9_PULMEM	0.968442	0.015802	pulmonary	TRUE	reported	textfile	1	8504421	0.002017	9.88E-22	474237	eosinophi	TRUE	reported	igd	0.000194	91.84385	2	TRUE
rs1611236	A	G	A	G	-0.04295	-0.0091	0.322915	0.224527	FALSE	FALSE	FALSE	I9_PULMEM	0.612858	0.017986	pulmonary	TRUE	reported	textfile	6	29748690	0.002125	8.34E-91	471744	eosinophi	TRUE	reported	igd	0.000865	408.4566	2	TRUE
rs1672753	T	C	T	C	-0.01748	-0.01886	0.811014	0.783319	FALSE	FALSE	FALSE	I9_PULMEM	0.299421	0.018178	pulmonary	TRUE	reported	textfile	3	3221430	0.002545	6.74E-12	474234	eosinophi	TRUE	reported	igd	9.95E-05	47.1743	2	TRUE
rs1684578	T	G	T	G	0.016362	0.013335	0.415559	0.370196	FALSE	FALSE	FALSE	I9_PULMEM	0.394041	0.015646	pulmonary	TRUE	reported	textfile	16	57069650	0.002017	5.15E-16	472835	eosinophi	TRUE	reported	igd	0.000139	65.80504	2	TRUE
rs1689510	C	G	C	G	0.026511	-0.02623	0.337746	0.318962	FALSE	TRUE	FALSE	I9_PULMEM	0.10279	0.016076	pulmonary	TRUE	reported	textfile	12	56396768	0.002094	1.06E-36	474210	eosinophi	TRUE	reported	igd	0.000338	160.2865	2	TRUE
rs1690357	G	C	G	C	0.028502	-0.00652	0.077849	0.127526	FALSE	TRUE	FALSE	I9_PULMEM	0.775802	0.022886	pulmonary	TRUE	reported	textfile	5	14610309	0.003838	1.17E-13	472215	eosinophi	TRUE	reported	igd	0.000117	55.14916	2	TRUE
rs1695681	G	T	G	T	-0.02935	0.024513	0.080686	0.050462	FALSE	FALSE	FALSE	I9_PULMEM	0.475434	0.034348	pulmonary	TRUE	reported	textfile	16	57444002	0.003685	1.75E-15	474230	eosinophi	TRUE	reported	igd	0.000134	63.42788	2	TRUE
rs1706150	A	G	A	G	0.035528	0.015697	0.307403	0.359203	FALSE	FALSE	FALSE	I9_PULMEM	0.314579	0.015609	pulmonary	TRUE	reported	textfile	13	41246758	0.002159	8.04E-61	472835	eosinophi	TRUE	reported	igd	0.000572	270.7911	2	TRUE
rs1717583	A	G	A	G	0.032179	0.045184	0.237656	0.237485	FALSE	FALSE	FALSE	I9_PULMEM	0.011115	0.017795	pulmonary	TRUE	reported	textfile	16	88558164	0.002365	4.13E-42	473293	eosinophi	TRUE	reported	igd	0.000391	185.1318	2	TRUE
rs1729363	T	C	T	C	0.02962	0.038387	0.236264	0.261483	FALSE	FALSE	FALSE	I9_PULMEM	0.024955	0.017121	pulmonary	TRUE	reported	textfile	15	67442596	0.002337	8.92E-37	473774	eosinophi	TRUE	reported	igd	0.000339	160.6389	2	TRUE
rs174548	G	C	G	C	-0.02306	-0.04806	0.313571	0.389072	FALSE	TRUE	FALSE	I9_PULMEM	0.001792	0.015391	pulmonary	TRUE	reported	textfile	11	61571348	0.002137	4.08E-27	473290	eosinophi	TRUE	reported	igd	0.000246	116.4213	2	TRUE
rs1748247	A	G	A	G	-0.03148	0.01779	0.098902	0.073697	FALSE	FALSE	FALSE	I9_PULMEM	0.53642	0.028775	pulmonary	TRUE	reported	textfile	10	44859618	0.003346	5.34E-21	472837	eosinophi	TRUE	reported	igd	0.000187	88.52039	2	TRUE

rs175705	G	C	G	C	0.03822	-0.00228	0.718533	0.606379	FALSE	TRUE	FALSE	I9_PULMEM	0.882078	0.015373	pulmonary	TRUE	reported	textfile	14	75975648	0.002213	8.80E-67	473155	eosinophi	TRUE	reported	igd	0.00063	298.2749	2	TRUE
rs1766827	T	G	T	G	-0.04166	-0.01579	0.117796	0.072658	FALSE	FALSE	FALSE	I9_PULMEM	0.583425	0.028796	pulmonary	TRUE	reported	textfile	1	1.99E+08	0.003072	7.48E-42	474236	eosinophi	TRUE	reported	igd	0.000388	183.87	2	TRUE
rs1768257	T	C	T	C	-0.01765	0.01264	0.201301	0.164012	FALSE	FALSE	FALSE	I9_PULMEM	0.532945	0.020272	pulmonary	TRUE	reported	textfile	2	43152304	0.002487	1.33E-12	474233	eosinophi	TRUE	reported	igd	0.000106	50.34871	2	TRUE
rs1768902	C	G	C	G	-0.01428	-0.00339	0.314415	0.310781	FALSE	TRUE	FALSE	I9_PULMEM	0.835049	0.016302	pulmonary	TRUE	reported	textfile	16	79739978	0.002135	2.31E-11	474226	eosinophi	TRUE	reported	igd	9.44E-05	44.7487	2	TRUE
rs1775869	T	C	T	C	-0.12212	0.060829	0.029555	0.044003	FALSE	FALSE	FALSE	I9_PULMEM	0.097521	0.036711	pulmonary	TRUE	reported	textfile	18	60920854	0.005948	1.32E-93	473291	eosinophi	TRUE	reported	igd	0.00089	421.552	2	TRUE
rs1784950	T	C	T	C	-0.02679	-0.00946	0.052001	0.037979	FALSE	FALSE	FALSE	I9_PULMEM	0.810458	0.039453	pulmonary	TRUE	reported	textfile	1	1.84E+08	0.004469	2.10E-09	472836	eosinophi	TRUE	reported	igd	7.60E-05	35.93275	2	TRUE
rs1800692	G	A	G	A	-0.01805	-0.01057	0.58754	0.630052	FALSE	FALSE	FALSE	I9_PULMEM	0.49602	0.015531	pulmonary	TRUE	reported	textfile	12	6442346	0.002039	8.92E-19	472213	eosinophi	TRUE	reported	igd	0.000166	78.37296	2	TRUE
rs180506	A	G	A	G	-0.02299	-0.04258	0.776272	0.819162	FALSE	FALSE	FALSE	I9_PULMEM	0.029312	0.019537	pulmonary	TRUE	reported	textfile	17	68274205	0.002385	5.82E-22	474234	eosinophi	TRUE	reported	igd	0.000196	92.91789	2	TRUE
rs1828803	A	C	A	C	0.013079	0.005351	0.392417	0.338889	FALSE	FALSE	FALSE	I9_PULMEM	0.735241	0.015825	pulmonary	TRUE	reported	textfile	4	2689449	0.002033	1.28E-10	473292	eosinophi	TRUE	reported	igd	8.74E-05	41.38781	2	TRUE
rs1861489	A	T	A	T	0.013285	0.020484	0.782181	0.760669	FALSE	TRUE	FALSE	I9_PULMEM	0.244015	0.017583	pulmonary	TRUE	reported	textfile	12	68610446	0.00242	4.14E-08	474236	eosinophi	TRUE	reported	igd	6.35E-05	30.13634	2	TRUE
rs201798	A	G	A	G	0.020184	0.010052	0.614944	0.679917	FALSE	FALSE	FALSE	I9_PULMEM	0.531928	0.016081	pulmonary	TRUE	reported	textfile	13	50954721	0.002044	5.71E-23	474235	eosinophi	TRUE	reported	igd	0.000206	97.51038	2	TRUE
rs2025489	G	A	G	A	0.015769	-0.00663	0.526005	0.513667	FALSE	FALSE	FALSE	I9_PULMEM	0.658667	0.015017	pulmonary	TRUE	reported	textfile	6	87814959	0.00199	2.42E-15	474232	eosinophi	TRUE	reported	igd	0.000132	62.79142	2	TRUE
rs2043293	A	C	A	C	-0.01488	-0.02006	0.825801	0.88273	FALSE	FALSE	FALSE	I9_PULMEM	0.393043	0.023484	pulmonary	TRUE	reported	textfile	19	19751456	0.002619	1.36E-08	472833	eosinophi	TRUE	reported	igd	6.83E-05	32.2843	2	TRUE
rs2053927	T	G	T	G	-0.01445	-0.05018	0.80327	0.835693	FALSE	FALSE	FALSE	I9_PULMEM	0.013362	0.020284	pulmonary	TRUE	reported	textfile	19	52280983	0.002521	1.02E-08	472828	eosinophi	TRUE	reported	igd	6.95E-05	32.8449	2	TRUE
rs2089979	G	A	G	A	-0.01493	0.016584	0.415851	0.432492	FALSE	FALSE	FALSE	I9_PULMEM	0.2728	0.015123	pulmonary	TRUE	reported	textfile	3	1.97E+08	0.002013	1.25E-13	472834	eosinophi	TRUE	reported	igd	0.000116	55.01592	2	TRUE
rs2095044	C	T	C	T	-0.09386	0.001741	0.742759	0.760245	FALSE	FALSE	FALSE	I9_PULMEM	0.921125	0.017588	pulmonary	TRUE	reported	textfile	9	6192796	0.002265	########	474235	eosinophi	TRUE	reported	igd	0.003608	1717.098	2	TRUE
rs2182885	A	G	A	G	0.026326	########	0.605228	0.552628	FALSE	FALSE	FALSE	I9_PULMEM	0.995972	0.015104	pulmonary	TRUE	reported	textfile	13	99855124	0.00203	2.05E-38	474234	eosinophi	TRUE	reported	igd	0.000355	168.1806	2	TRUE
rs2223043	G	A	G	A	0.019382	-0.00064	0.306981	0.378905	FALSE	FALSE	FALSE	I9_PULMEM	0.967547	0.015627	pulmonary	TRUE	reported	textfile	21	16438793	0.002295	3.23E-17	472836	eosinophi	TRUE	reported	igd	0.000151	71.32306	2	TRUE
rs2228467	C	T	C	T	0.062272	-0.01935	0.061584	0.076263	FALSE	FALSE	FALSE	I9_PULMEM	0.494234	0.028304	pulmonary	TRUE	reported	textfile	3	42906116	0.004111	8.83E-52	472835	eosinophi	TRUE	reported	igd	0.000485	229.4504	2	TRUE
rs2239633	A	G	A	G	0.034771	-0.0058	0.483519	0.454471	FALSE	FALSE	FALSE	I9_PULMEM	0.699906	0.015058	pulmonary	TRUE	reported	textfile	14	23589057	0.001986	1.50E-68	472835	eosinophi	TRUE	reported	igd	0.000648	306.5307	2	TRUE
rs2242193	A	G	A	G	0.012279	-0.04884	0.421302	0.449805	FALSE	FALSE	FALSE	I9_PULMEM	0.001228	0.015109	pulmonary	TRUE	reported	textfile	1	1.55E+08	0.002018	1.20E-09	472835	eosinophi	TRUE	reported	igd	7.83E-05	37.02387	2	TRUE
rs2253427	C	T	C	T	-0.01381	0.017129	0.778112	0.802841	FALSE	FALSE	FALSE	I9_PULMEM	0.364243	0.018879	pulmonary	TRUE	reported	textfile	20	1552430	0.002397	8.68E-09	470964	eosinophi	TRUE	reported	igd	7.04E-05	33.16919	2	TRUE
rs2255531	A	G	A	G	-0.01639	-0.04148	0.350975	0.393186	FALSE	FALSE	FALSE	I9_PULMEM	0.007176	0.015428	pulmonary	TRUE	reported	textfile	12	1.21E+08	0.002079	3.39E-15	473289	eosinophi	TRUE	reported	igd	0.000131	62.11294	2	TRUE
rs2282718	A	G	A	G	-0.01142	0.014741	0.37278	0.404961	FALSE	FALSE	FALSE	I9_PULMEM	0.336133	0.015326	pulmonary	TRUE	reported	textfile	1	25241056	0.002078	3.95E-08	471742	eosinophi	TRUE	reported	igd	6.40E-05	30.21283	2	TRUE
rs2399441	C	T	C	T	-0.01887	0.00827	0.350554	0.313583	FALSE	FALSE	FALSE	I9_PULMEM	0.608566	0.016148	pulmonary	TRUE	reported	textfile	3	1.13E+08	0.002069	8.08E-20	474231	eosinophi	TRUE	reported	igd	0.000175	83.16277	2	TRUE
rs2419313	A	G	A	G	-0.02073	-0.06179	0.142533	0.136456	FALSE	FALSE	FALSE	I9_PULMEM	0.005474	0.022245	pulmonary	TRUE	reported	textfile	10	1.12E+08	0.002869	5.21E-13	474228	eosinophi	TRUE	reported	igd	0.00011	52.19272	2	TRUE
rs2431097	T	C	T	C	0.015023	0.012949	0.485547	0.480231	FALSE	FALSE	FALSE	I9_PULMEM	0.390087	0.015066	pulmonary	TRUE	reported	textfile	5	1.6E+08	0.001999	5.95E-14	472835	eosinophi	TRUE	reported	igd	0.000119	56.47886	2	TRUE
rs244689	G	A	G	A	-0.02124	-0.01139	0.876598	0.748562	FALSE	FALSE	FALSE	I9_PULMEM	0.510423	0.017307	pulmonary	TRUE	reported	textfile	5	1.33E+08	0.00303	2.46E-12	472835	eosinophi	TRUE	reported	igd	0.000104	49.1385	2	TRUE
rs2497318	T	C	T	C	-0.02986	0.016215	0.442619	0.450219	FALSE	FALSE	FALSE	I9_PULMEM	0.282714	0.015094	pulmonary	TRUE	reported	textfile	10	94432000	0.00202	1.96E-49	473293	eosinophi	TRUE	reported	igd	0.000462	218.5704	2	TRUE
rs2502995	C	T	C	T	0.021853	-0.03132	0.57046	0.567496	FALSE	FALSE	FALSE	I9_PULMEM	0.039082	0.01518	pulmonary	TRUE	reported	textfile	1	24199290	0.002002	1.06E-27	471424	eosinophi	TRUE	reported	igd	0.000253	119.1495	2	TRUE
rs2505521	T	C	T	C	-0.01517	0.016161	0.829154	0.775643	FALSE	FALSE	FALSE	I9_PULMEM	0.371504	0.018085	pulmonary	TRUE	reported	textfile	10	43804617	0.002675	1.45E-08	472837	eosinophi	TRUE	reported	igd	6.80E-05	32.16466	2	TRUE
rs2566133	C	T	C	T	-0.01437	0.016113	0.583621	0.523259	FALSE	FALSE	FALSE	I9_PULMEM	0.283837	0.015035	pulmonary	TRUE	reported	textfile	4	39022515	0.002015	1.06E-12	473153	eosinophi	TRUE	reported	igd	0.000107	50.82289	2	TRUE
rs2568928	G	A	G	A	0.015221	-0.01485	0.756846	0.849543	FALSE	FALSE	FALSE	I9_PULMEM	0.479119	0.020976	pulmonary	TRUE	reported	textfile	5	1.27E+08	0.002308	4.38E-11	474237	eosinophi	TRUE	reported	igd	9.17E-05	43.49236	2	TRUE
rs2579506	A	G	A	G	0.047465	-0.01915	0.386176	0.474983	FALSE	FALSE	FALSE	I9_PULMEM	0.203828	0.015069	pulmonary	TRUE	reported	textfile	2	97207321	0.002032	########	473149	eosinophi	TRUE	reported	igd	0.001152	545.6294	2	TRUE
rs2646438	A	G	A	G	-0.02052	-0.0102	0.565689	0.580118	FALSE	FALSE	FALSE	I9_PULMEM	0.502211	0.015198	pulmonary	TRUE	reported	textfile	10	8565675	0.002001	1.20E-24	473155	eosinophi	TRUE	reported	igd	0.000222	105.121	2	TRUE
rs2683913	G	A	G	A	-0.01508	0.003164	0.826856	0.845489	FALSE	FALSE	FALSE	I9_PULMEM	0.879511	0.020872	pulmonary	TRUE	reported	textfile	3	1.12E+08	0.002618	8.68E-09	474235	eosinophi	TRUE	reported	igd	6.99E-05	33.15687	2	TRUE
rs2713548	T	C	T	C	0.012505	0.019611	0.632646	0.618291	FALSE	FALSE	FALSE	I9_PULMEM	0.203186	0.015411	pulmonary	TRUE	reported	textfile	2	2.27E+08	0.002046	1.02E-09	473298	eosinophi	TRUE	reported	igd	7.89E-05	37.35548	2	TRUE
rs2788211	C	T	C	T	0.016029	0.040176	0.81734	0.823239	FALSE	FALSE	FALSE	I9_PULMEM	0.041164	0.019676	pulmonary	TRUE	reported	textfile	6	697500	0.002573	4.79E-10	473155	eosinophi	TRUE	reported	igd	8.20E-05	38.80891	2	TRUE
rs2807740	T	C	T	C	0.035325	-0.04161	0.76966	0.798303	FALSE	FALSE	FALSE	I9_PULMEM	0.026321	0.018729	pulmonary	TRUE	reported	textfile	10	28784483	0.002358	1.05E-50	474231	eosinophi	TRUE	reported	igd	0.000473	224.4269	2	TRUE
rs2836290	A	G	A	G	0.016579	0.030198	0.132809	0.128287	FALSE	FALSE	FALSE	I9_PULMEM	0.179587	0.022502	pulmonary	TRUE	reported	textfile	15	74712178	0.002935	1.66E-08	474235	eosinophi	TRUE	reported	igd	6.73E-05	31.90793	2	TRUE
rs2838317	C	A	C	A	0.012853	-0.02134	0.550072	0.65947	FALSE	FALSE	FALSE	I9_PULMEM	0.179008	0.01588	pulmonary	TRUE	reported	textfile	21	44991791	0.001994	1.20E-10	473149	eosinophi	TRUE	reported	igd	8.78E-05	41.54865	2	TRUE
rs2838331	C	T	C	T	0.061635	0.005422	0.624131	0.687936	FALSE	FALSE	FALSE	I9_PULMEM	0.737439	0.016174	pulmonary	TRUE	reported	textfile	6	32587213	0.002048	########	473151	eosinophi	TRUE	reported	igd	0.001911	905.7181	2	TRUE
rs2842132	T	A	T	A	0.0358	-0.01446	0.102092	0.123576	FALSE	TRUE	FALSE	I9_PULMEM	0.525269	0.022764	pulmonary	TRUE	reported	textfile	21	36286938	0.003291	1.57E-27	474230	eosinophi	TRUE	reported	igd	0.000249	118.3337	2	TRUE
rs2847266	T	C	T	C	-0.01444	0.012597	0.716855	0.701675	FALSE	FALSE	FALSE	I9_PULMEM	0.443978	0.016456	pulmonary	TRUE	reported	textfile	18	12773338	0.002242	1.22E-10	471755	eosinophi	TRUE	reported	igd	8.79E-05	41.49365	2	TRUE

rs2853203	A	G	A	G	-0.02965	-0.06111	0.906507	0.886688	FALSE	FALSE	FALSE	I9_PULMEM	0.009158	0.023451	pulmonary	TRUE	reported	textfile	12	1.24E+08	0.003416	4.19E-18	473296	eosinophi	TRUE	reported	igd	0.000159	75.35286		2	TRUE
rs2894401	G	A	G	A	-0.02054	-0.01307	0.705252	0.776819	FALSE	FALSE	FALSE	I9_PULMEM	0.469111	0.018049	pulmonary	TRUE	reported	textfile	6	35408959	0.002187	6.16E-21	472833	eosinophi	TRUE	reported	igd	0.000187	88.21527		2	TRUE
rs290430	T	C	T	C	-0.01208	-0.00813	0.344859	0.376933	FALSE	FALSE	FALSE	I9_PULMEM	0.599498	0.015475	pulmonary	TRUE	reported	textfile	20	52675648	0.002095	8.31E-09	473148	eosinophi	TRUE	reported	igd	7.03E-05	33.25339		2	TRUE
rs2920505	A	G	A	G	-0.02964	-0.02174	0.601796	0.554959	FALSE	FALSE	FALSE	I9_PULMEM	0.150342	0.015112	pulmonary	TRUE	reported	textfile	3	12335081	0.002019	9.47E-49	474232	eosinophi	TRUE	reported	igd	0.000454	215.5754		2	TRUE
rs2942062	G	A	G	A	-0.02271	0.028672	0.937362	0.944388	FALSE	FALSE	FALSE	I9_PULMEM	0.382145	0.032808	pulmonary	TRUE	reported	textfile	3	1.12E+08	0.004079	2.65E-08	473298	eosinophi	TRUE	reported	igd	6.55E-05	30.99192		2	TRUE
rs295	C	A	C	A	0.015869	-0.00972	0.235064	0.228688	FALSE	FALSE	FALSE	I9_PULMEM	0.585658	0.017839	pulmonary	TRUE	reported	textfile	8	19816238	0.002337	1.16E-11	474228	eosinophi	TRUE	reported	igd	9.72E-05	46.10838		2	TRUE
rs295273	A	G	A	G	0.020579	0.019366	0.255616	0.255011	FALSE	FALSE	FALSE	I9_PULMEM	0.261042	0.017231	pulmonary	TRUE	reported	textfile	9	86466968	0.002274	1.50E-19	472811	eosinophi	TRUE	reported	igd	0.000173	81.89658		2	TRUE
rs2979489	A	G	A	G	0.013752	-0.01743	0.741697	0.709656	FALSE	FALSE	FALSE	I9_PULMEM	0.292149	0.016545	pulmonary	TRUE	reported	textfile	8	30280833	0.002266	1.33E-09	473298	eosinophi	TRUE	reported	igd	7.78E-05	36.83071		2	TRUE
rs2992333	A	G	A	G	-0.03415	0.000291	0.598657	0.685667	FALSE	FALSE	FALSE	I9_PULMEM	0.985597	0.016143	pulmonary	TRUE	reported	textfile	10	26727454	0.002028	1.35E-63	470794	eosinophi	TRUE	reported	igd	0.000602	283.609		2	TRUE
rs301162	G	A	G	A	0.026139	0.011506	0.853049	0.880178	FALSE	FALSE	FALSE	I9_PULMEM	0.617984	0.023071	pulmonary	TRUE	reported	textfile	16	85810647	0.002803	1.16E-20	474237	eosinophi	TRUE	reported	igd	0.000183	86.96208		2	TRUE
rs3024971	G	T	G	T	-0.04023	-0.0021	0.107204	0.044386	FALSE	FALSE	FALSE	I9_PULMEM	0.954077	0.036403	pulmonary	TRUE	reported	textfile	12	57493727	0.003209	5.04E-36	474224	eosinophi	TRUE	reported	igd	0.000331	157.1817		2	TRUE
rs3093023	A	G	A	G	0.012677	-0.00514	0.43381	0.44544	FALSE	FALSE	FALSE	I9_PULMEM	0.734264	0.01514	pulmonary	TRUE	reported	textfile	6	1.68E+08	0.001998	2.27E-10	473773	eosinophi	TRUE	reported	igd	8.50E-05	40.25689		2	TRUE
rs3096309	T	C	T	C	0.014823	-0.001	0.824069	0.873715	FALSE	FALSE	FALSE	I9_PULMEM	0.964791	0.022629	pulmonary	TRUE	reported	textfile	16	89431603	0.002608	1.36E-08	472838	eosinophi	TRUE	reported	igd	6.83E-05	32.30392		2	TRUE
rs3110791	C	T	C	T	0.015409	0.003855	0.623422	0.676136	FALSE	FALSE	FALSE	I9_PULMEM	0.81149	0.016164	pulmonary	TRUE	reported	textfile	7	1.35E+08	0.002059	7.49E-14	474221	eosinophi	TRUE	reported	igd	0.000118	56.00598		2	TRUE
rs3218148	A	G	A	G	-0.01768	0.016899	0.539783	0.654964	FALSE	FALSE	FALSE	I9_PULMEM	0.284714	0.015797	pulmonary	TRUE	reported	textfile	1	23851787	0.001991	7.08E-19	470336	eosinophi	TRUE	reported	igd	0.000168	78.83551		2	TRUE
rs3398266	A	C	A	C	0.020435	-0.00096	0.285267	0.253229	FALSE	FALSE	FALSE	I9_PULMEM	0.955426	0.017246	pulmonary	TRUE	reported	textfile	9	1.39E+08	0.002192	1.19E-20	474229	eosinophi	TRUE	reported	igd	0.000183	86.90932		2	TRUE
rs3417306	A	G	A	G	0.054559	0.064558	0.072995	0.055684	FALSE	FALSE	FALSE	I9_PULMEM	0.055776	0.033751	pulmonary	TRUE	reported	textfile	8	1.45E+08	0.004091	1.55E-40	468770	eosinophi	TRUE	reported	igd	0.000379	177.8574		2	TRUE
rs3421065	A	G	A	G	-0.17557	0.010772	0.020746	0.006882	FALSE	FALSE	FALSE	I9_PULMEM	0.905489	0.090728	pulmonary	TRUE	reported	textfile	17	4535314	0.006973	########	473775	eosinophi	TRUE	reported	igd	0.001336	633.9204		2	TRUE
rs3421286	G	A	G	A	0.020115	-0.03351	0.220743	0.144553	FALSE	FALSE	FALSE	I9_PULMEM	0.116937	0.021376	pulmonary	TRUE	reported	textfile	15	43702964	0.002401	5.74E-17	474227	eosinophi	TRUE	reported	igd	0.000148	70.18655		2	TRUE
rs3429028	A	G	A	G	-0.04589	-0.00376	0.257132	0.216854	FALSE	FALSE	FALSE	I9_PULMEM	0.836585	0.018233	pulmonary	TRUE	reported	textfile	2	2.43E+08	0.002267	4.73E-91	473295	eosinophi	TRUE	reported	igd	0.000865	409.6902		2	TRUE
rs3436317	G	C	G	C	0.01341	0.018234	0.243607	0.18471	FALSE	TRUE	FALSE	I9_PULMEM	0.350217	0.019519	pulmonary	TRUE	reported	textfile	13	1E+08	0.002326	8.43E-09	473153	eosinophi	TRUE	reported	igd	7.02E-05	33.23811		2	TRUE
rs3443969	T	C	T	C	-0.03794	0.020792	0.035263	0.027286	FALSE	FALSE	FALSE	I9_PULMEM	0.651187	0.045987	pulmonary	TRUE	reported	textfile	11	33901483	0.005456	3.67E-12	474229	eosinophi	TRUE	reported	igd	0.000102	48.3655		2	TRUE
rs3444895	T	C	T	C	-0.02525	0.006696	0.106374	0.107035	FALSE	FALSE	FALSE	I9_PULMEM	0.783419	0.024361	pulmonary	TRUE	reported	textfile	1	1.61E+08	0.003211	3.87E-15	473775	eosinophi	TRUE	reported	igd	0.000131	61.85054		2	TRUE
rs3446695	T	C	T	C	-0.01354	-0.01073	0.5676	0.557946	FALSE	FALSE	FALSE	I9_PULMEM	0.478676	0.015152	pulmonary	TRUE	reported	textfile	19	3353622	0.002029	2.60E-11	472213	eosinophi	TRUE	reported	igd	9.43E-05	44.53192		2	TRUE
rs34495	T	G	T	G	-0.01983	-0.04199	0.304172	0.168298	FALSE	FALSE	FALSE	I9_PULMEM	0.036436	0.020069	pulmonary	TRUE	reported	textfile	5	98265807	0.002155	3.67E-20	471741	eosinophi	TRUE	reported	igd	0.000179	84.6738		2	TRUE
rs3450510	G	A	G	A	-0.02613	-0.03635	0.306171	0.278001	FALSE	FALSE	FALSE	I9_PULMEM	0.030892	0.016841	pulmonary	TRUE	reported	textfile	22	24624609	0.00216	1.22E-33	474218	eosinophi	TRUE	reported	igd	0.000308	146.3086		2	TRUE
rs346835	T	C	T	C	-0.02669	-0.0215	0.328293	0.36155	FALSE	FALSE	FALSE	I9_PULMEM	0.172361	0.015752	pulmonary	TRUE	reported	textfile	2	8438693	0.002104	7.55E-37	473295	eosinophi	TRUE	reported	igd	0.00034	160.9177		2	TRUE
rs350836	T	C	T	C	-0.0172	-0.00805	0.709927	0.733533	FALSE	FALSE	FALSE	I9_PULMEM	0.636014	0.017001	pulmonary	TRUE	reported	textfile	19	4080516	0.002205	6.41E-15	474205	eosinophi	TRUE	reported	igd	0.000128	60.85389		2	TRUE
rs3524918	G	A	G	A	0.039991	0.004446	0.099837	0.050412	FALSE	FALSE	FALSE	I9_PULMEM	0.896966	0.034331	pulmonary	TRUE	reported	textfile	1	12099345	0.003364	1.46E-32	473297	eosinophi	TRUE	reported	igd	0.000299	141.3223		2	TRUE
rs3540952	A	G	A	G	0.050096	0.031122	0.075881	0.095465	FALSE	FALSE	FALSE	I9_PULMEM	0.223836	0.025585	pulmonary	TRUE	reported	textfile	2	2.14E+08	0.003754	1.43E-40	473296	eosinophi	TRUE	reported	igd	0.000376	178.0802		2	TRUE
rs3585075	T	C	T	C	0.043576	0.046923	0.019768	0.026978	FALSE	FALSE	FALSE	I9_PULMEM	0.315193	0.046718	pulmonary	TRUE	reported	textfile	17	7578671	0.007273	2.14E-09	474212	eosinophi	TRUE	reported	igd	7.57E-05	35.89763		2	TRUE
rs3608435	A	G	A	G	-0.04537	0.021348	0.091843	0.085763	FALSE	FALSE	FALSE	I9_PULMEM	0.427994	0.026934	pulmonary	TRUE	reported	textfile	19	1079959	0.003441	1.17E-39	472835	eosinophi	TRUE	reported	igd	0.000367	173.8237		2	TRUE
rs3731211	A	T	A	T	0.017545	-0.00558	0.720317	0.772015	FALSE	TRUE	FALSE	I9_PULMEM	0.754505	0.017851	pulmonary	TRUE	reported	textfile	9	21986847	0.002213	2.34E-15	474235	eosinophi	TRUE	reported	igd	0.000133	62.85533		2	TRUE
rs3742704	C	A	C	A	0.01948	0.015716	0.091601	0.081371	FALSE	FALSE	FALSE	I9_PULMEM	0.564442	0.027273	pulmonary	TRUE	reported	textfile	14	88477882	0.003441	1.54E-08	472837	eosinophi	TRUE	reported	igd	6.78E-05	32.04843		2	TRUE
rs3746420	C	G	C	G	-0.03181	-0.00433	0.061219	0.050293	FALSE	TRUE	FALSE	I9_PULMEM	0.900024	0.034436	pulmonary	TRUE	reported	textfile	20	50140627	0.004163	2.26E-14	474234	eosinophi	TRUE	reported	igd	0.000123	58.36818		2	TRUE
rs3747869	C	A	C	A	0.019825	-0.01835	0.90083	0.811551	FALSE	FALSE	FALSE	I9_PULMEM	0.343324	0.019361	pulmonary	TRUE	reported	textfile	10	73520632	0.003316	2.31E-09	472837	eosinophi	TRUE	reported	igd	7.56E-05	35.74337		2	TRUE
rs3756374	T	G	T	G	0.016911	0.028986	0.119107	0.100813	FALSE	FALSE	FALSE	I9_PULMEM	0.24811	0.025097	pulmonary	TRUE	reported	textfile	5	1.43E+08	0.00307	3.71E-08	472837	eosinophi	TRUE	reported	igd	6.42E-05	30.3431		2	TRUE
rs3759332	C	T	C	T	-0.01194	0.013556	0.390595	0.383989	FALSE	FALSE	FALSE	I9_PULMEM	0.382419	0.01552	pulmonary	TRUE	reported	textfile	12	6491078	0.002064	7.54E-09	473153	eosinophi	TRUE	reported	igd	7.07E-05	33.43672		2	TRUE
rs3785356	T	C	T	C	0.030168	-0.01666	0.298035	0.264479	FALSE	FALSE	FALSE	I9_PULMEM	0.325021	0.016924	pulmonary	TRUE	reported	textfile	16	27349168	0.00218	1.70E-43	473258	eosinophi	TRUE	reported	igd	0.000404	191.5042		2	TRUE
rs3786586	G	A	G	A	0.031904	0.027374	0.154646	0.195424	FALSE	FALSE	FALSE	I9_PULMEM	0.149615	0.018998	pulmonary	TRUE	reported	textfile	19	16495287	0.00275	4.42E-31	473290	eosinophi	TRUE	reported	igd	0.000284	134.5932		2	TRUE
rs3790163	G	A	G	A	-0.01529	-0.05054	0.792407	0.82788	FALSE	FALSE	FALSE	I9_PULMEM	0.011033	0.019886	pulmonary	TRUE	reported	textfile	20	10647951	0.002521	1.35E-09	472216	eosinophi	TRUE	reported	igd	7.79E-05	36.79434		2	TRUE
rs3812206	T	C	T	C	-0.01224	0.009811	0.300301	0.338881	FALSE	FALSE	FALSE	I9_PULMEM	0.535826	0.015846	pulmonary	TRUE	reported	textfile	6	16700098	0.002181	2.07E-08	470484	eosinophi	TRUE	reported	igd	6.69E-05	31.48529		2	TRUE
rs3823536	A	G	A	G	-0.01782	-0.00275	0.467209	0.435298	FALSE	FALSE	FALSE	I9_PULMEM	0.856002	0.015135	pulmonary	TRUE	reported	textfile	7	1.29E+08	0.001986	3.16E-19	473292	eosinophi	TRUE	reported	igd	0.00017	80.4929		2	TRUE
rs3824867	G	A	G	A	0.016826	-0.05328	0.712364	0.61103	FALSE	FALSE	FALSE	I9_PULMEM	0.000509	0.015327	pulmonary	TRUE	reported	textfile	11	47468569	0.002193	1.77E-14	471752	eosinophi	TRUE	reported	igd	0.000125	58.86846		2	TRUE

rs3846855	A	G	A	G	0.028194	-0.02555	0.182937	0.310179	FALSE	FALSE	FALSE	I9_PULMEM	0.116829	0.016289	pulmonary	TRUE	reported	textfile	6	33555877	0.002567	5.01E-28	473292	eosinophi	TRUE	reported	igd	0.000255	120.6312	2	TRUE
rs3850107	G	C	G	C	-0.01375	0.029289	0.29438	0.276131	FALSE	TRUE	FALSE	I9_PULMEM	0.081796	0.016829	pulmonary	TRUE	reported	textfile	16	78150341	0.002196	3.94E-10	473290	eosinophi	TRUE	reported	igd	8.28E-05	39.21047	2	TRUE
rs3950296	G	C	G	C	0.023638	-0.01434	0.244421	0.273861	FALSE	TRUE	FALSE	I9_PULMEM	0.395401	0.016869	pulmonary	TRUE	reported	textfile	3	1.69E+08	0.002297	8.30E-25	474236	eosinophi	TRUE	reported	igd	0.000223	105.9004	2	TRUE
rs397187	C	T	C	T	-0.01228	-0.00489	0.429693	0.370127	FALSE	FALSE	FALSE	I9_PULMEM	0.753916	0.015588	pulmonary	TRUE	reported	textfile	17	8784759	0.002022	1.28E-09	473147	eosinophi	TRUE	reported	igd	7.80E-05	36.88955	2	TRUE
rs4074672	T	C	T	C	0.014516	0.030939	0.369285	0.294356	FALSE	FALSE	FALSE	I9_PULMEM	0.059907	0.016444	pulmonary	TRUE	reported	textfile	3	1.84E+08	0.00205	1.48E-12	472834	eosinophi	TRUE	reported	igd	0.000106	50.14	2	TRUE
rs410867	G	A	G	A	-0.05941	-0.05016	0.216683	0.222701	FALSE	FALSE	FALSE	I9_PULMEM	0.005773	0.018171	pulmonary	TRUE	reported	textfile	19	16427111	0.00241	########	474234	eosinophi	TRUE	reported	igd	0.00128	607.7734	2	TRUE
rs412884	C	T	C	T	0.057634	-0.0138	0.672171	0.688484	FALSE	FALSE	FALSE	I9_PULMEM	0.394712	0.01622	pulmonary	TRUE	reported	textfile	19	40219449	0.002109	########	474234	eosinophi	TRUE	reported	igd	0.001572	746.7969	2	TRUE
rs4131338	A	C	A	C	0.050131	0.029334	0.030679	0.017483	FALSE	FALSE	FALSE	I9_PULMEM	0.610536	0.057596	pulmonary	TRUE	reported	textfile	1	79411968	0.005645	7.05E-19	471757	eosinophi	TRUE	reported	igd	0.000167	78.86476	2	TRUE
rs4148757	C	A	C	A	-0.01715	-0.00817	0.210055	0.133719	FALSE	FALSE	FALSE	I9_PULMEM	0.711069	0.022068	pulmonary	TRUE	reported	textfile	1	2.3E+08	0.002441	2.24E-12	472363	eosinophi	TRUE	reported	igd	0.000104	49.34452	2	TRUE
rs4149909	G	A	G	A	-0.0334	0.043502	0.032649	0.031638	FALSE	FALSE	FALSE	I9_PULMEM	0.312552	0.043076	pulmonary	TRUE	reported	textfile	1	2.42E+08	0.005569	2.07E-09	473776	eosinophi	TRUE	reported	igd	7.59E-05	35.96969	2	TRUE
rs4236746	G	A	G	A	0.056854	-0.02294	0.975145	0.97691	FALSE	FALSE	FALSE	I9_PULMEM	0.647292	0.050148	pulmonary	TRUE	reported	textfile	8	1.31E+08	0.006399	6.78E-19	474237	eosinophi	TRUE	reported	igd	0.000166	78.9398	2	TRUE
rs4240624	A	G	A	G	-0.02039	-0.05939	0.909011	0.857237	FALSE	FALSE	FALSE	I9_PULMEM	0.005078	0.021197	pulmonary	TRUE	reported	textfile	8	9184231	0.003453	3.62E-09	473155	eosinophi	TRUE	reported	igd	7.37E-05	34.87242	2	TRUE
rs4243951	G	A	G	A	-0.01391	-0.03945	0.755956	0.811791	FALSE	FALSE	FALSE	I9_PULMEM	0.040909	0.019294	pulmonary	TRUE	reported	textfile	11	9856524	0.00232	2.10E-09	472835	eosinophi	TRUE	reported	igd	7.60E-05	35.93264	2	TRUE
rs4280242	T	C	T	C	-0.03291	0.008294	0.754112	0.749948	FALSE	FALSE	FALSE	I9_PULMEM	0.632946	0.017366	pulmonary	TRUE	reported	textfile	16	2830482	0.002318	1.03E-45	473154	eosinophi	TRUE	reported	igd	0.000426	201.6073	2	TRUE
rs4310436	A	G	A	G	0.018785	-0.03034	0.119611	0.137496	FALSE	FALSE	FALSE	I9_PULMEM	0.16398	0.021796	pulmonary	TRUE	reported	textfile	1	39489221	0.003065	9.15E-10	474227	eosinophi	TRUE	reported	igd	7.92E-05	37.56294	2	TRUE
rs4366169	C	A	C	A	0.012354	0.021352	0.310424	0.299225	FALSE	FALSE	FALSE	I9_PULMEM	0.192319	0.016378	pulmonary	TRUE	reported	textfile	9	16911638	0.002145	8.70E-09	474234	eosinophi	TRUE	reported	igd	6.99E-05	33.17102	2	TRUE
rs4409785	C	T	C	T	0.016435	-0.02223	0.172343	0.166187	FALSE	FALSE	FALSE	I9_PULMEM	0.269439	0.020127	pulmonary	TRUE	reported	textfile	11	95311422	0.00263	4.25E-10	472838	eosinophi	TRUE	reported	igd	8.26E-05	39.05045	2	TRUE
rs4557713	G	A	G	A	-0.05976	-0.02532	0.045218	0.071722	FALSE	FALSE	FALSE	I9_PULMEM	0.390064	0.029461	pulmonary	TRUE	reported	textfile	8	48651633	0.005157	5.10E-31	468083	eosinophi	TRUE	reported	igd	0.000287	134.2753	2	TRUE
rs4652560	T	A	T	A	-0.0154	0.042693	0.617764	0.69414	FALSE	TRUE	FALSE	I9_PULMEM	0.009185	0.016388	pulmonary	TRUE	reported	textfile	1	1.81E+08	0.002044	5.23E-14	474231	eosinophi	TRUE	reported	igd	0.00012	56.74252	2	TRUE
rs466547	G	A	G	A	0.019109	0.015576	0.857978	0.909924	FALSE	FALSE	FALSE	I9_PULMEM	0.550825	0.026111	pulmonary	TRUE	reported	textfile	9	4834034	0.002846	1.95E-11	472838	eosinophi	TRUE	reported	igd	9.53E-05	45.08212	2	TRUE
rs4675190	T	C	T	C	-0.01167	-0.00995	0.591061	0.573984	FALSE	FALSE	FALSE	I9_PULMEM	0.511821	0.015172	pulmonary	TRUE	reported	textfile	2	2.28E+08	0.002017	7.57E-09	472213	eosinophi	TRUE	reported	igd	7.08E-05	33.45263	2	TRUE
rs4680250	G	C	G	C	-0.01573	-0.00558	0.692997	0.706938	FALSE	TRUE	FALSE	I9_PULMEM	0.736229	0.016551	pulmonary	TRUE	reported	textfile	3	1.51E+08	0.002175	4.85E-13	472215	eosinophi	TRUE	reported	igd	0.000111	52.33087	2	TRUE
rs4703589	C	T	C	T	0.014451	0.032649	0.532652	0.591875	FALSE	FALSE	FALSE	I9_PULMEM	0.032602	0.015278	pulmonary	TRUE	reported	textfile	5	72097351	0.001983	3.26E-13	474234	eosinophi	TRUE	reported	igd	0.000112	53.10661	2	TRUE
rs4703730	T	C	T	C	-0.01603	0.01316	0.516976	0.596086	FALSE	FALSE	FALSE	I9_PULMEM	0.389544	0.015295	pulmonary	TRUE	reported	textfile	5	76549688	0.001984	6.91E-16	473295	eosinophi	TRUE	reported	igd	0.000138	65.24768	2	TRUE
rs4722171	G	A	G	A	-0.02725	0.030027	0.592101	0.462686	FALSE	FALSE	FALSE	I9_PULMEM	0.045742	0.01503	pulmonary	TRUE	reported	textfile	7	22785717	0.002019	1.83E-41	474232	eosinophi	TRUE	reported	igd	0.000384	182.2024	2	TRUE
rs473739	G	T	G	T	-0.01388	0.018557	0.31889	0.2613	FALSE	FALSE	FALSE	I9_PULMEM	0.277522	0.017089	pulmonary	TRUE	reported	textfile	11	64147919	0.002128	7.07E-11	473293	eosinophi	TRUE	reported	igd	8.99E-05	42.55582	2	TRUE
rs4739140	T	C	T	C	-0.01455	-0.00139	0.611355	0.618225	FALSE	FALSE	FALSE	I9_PULMEM	0.928354	0.015476	pulmonary	TRUE	reported	textfile	8	79753010	0.002057	1.53E-12	471755	eosinophi	TRUE	reported	igd	0.000106	50.0604	2	TRUE
rs4746153	C	G	C	G	0.015663	-0.0207	0.185407	0.181074	FALSE	TRUE	FALSE	I9_PULMEM	0.286184	0.019405	pulmonary	TRUE	reported	textfile	10	75598282	0.002552	8.58E-10	474234	eosinophi	TRUE	reported	igd	7.94E-05	37.66923	2	TRUE
rs4849748	C	T	C	T	-0.01436	0.027749	0.266942	0.224373	FALSE	FALSE	FALSE	I9_PULMEM	0.131266	0.018387	pulmonary	TRUE	reported	textfile	2	1.12E+08	0.002511	1.09E-08	466515	eosinophi	TRUE	reported	igd	7.01E-05	32.71407	2	TRUE
rs4857909	G	A	G	A	0.120719	0.032881	0.87972	0.867278	FALSE	FALSE	FALSE	I9_PULMEM	0.137062	0.022115	pulmonary	TRUE	reported	textfile	3	1.28E+08	0.003053	########	473295	eosinophi	TRUE	reported	igd	0.003293	1563.493	2	TRUE
rs4870977	C	G	C	G	-0.01875	0.025491	0.870857	0.877028	FALSE	TRUE	FALSE	I9_PULMEM	0.263583	0.022802	pulmonary	TRUE	reported	textfile	8	1.28E+08	0.002958	2.40E-10	474236	eosinophi	TRUE	reported	igd	8.47E-05	40.17949	2	TRUE
rs4908835	C	T	C	T	0.019184	0.026739	0.161355	0.172329	FALSE	FALSE	FALSE	I9_PULMEM	0.17972	0.01993	pulmonary	TRUE	reported	textfile	1	9357460	0.002719	1.80E-12	474234	eosinophi	TRUE	reported	igd	0.000105	49.78037	2	TRUE
rs4931002	A	C	A	C	-0.02158	-0.00791	0.779279	0.805253	FALSE	FALSE	FALSE	I9_PULMEM	0.675845	0.018924	pulmonary	TRUE	reported	textfile	12	32143169	0.002388	1.73E-19	474236	eosinophi	TRUE	reported	igd	0.000172	81.66433	2	TRUE
rs495149	T	C	T	C	0.024275	0.025191	0.164184	0.157181	FALSE	FALSE	FALSE	I9_PULMEM	0.220923	0.020579	pulmonary	TRUE	reported	textfile	10	89795523	0.002674	1.15E-19	474234	eosinophi	TRUE	reported	igd	0.000174	82.41264	2	TRUE
rs496475	G	T	G	T	0.035017	-0.03139	0.387008	0.345997	FALSE	FALSE	FALSE	I9_PULMEM	0.046306	0.015755	pulmonary	TRUE	reported	textfile	9	1.14E+08	0.002033	1.83E-66	474232	eosinophi	TRUE	reported	igd	0.000625	296.6752	2	TRUE
rs520461	G	A	G	A	-0.01375	0.01894	0.2218	0.246359	FALSE	FALSE	FALSE	I9_PULMEM	0.276792	0.017415	pulmonary	TRUE	reported	textfile	11	1.26E+08	0.00239	9.08E-09	474233	eosinophi	TRUE	reported	igd	6.98E-05	33.08883	2	TRUE
rs526369	G	A	G	A	0.018996	0.004774	0.143107	0.202295	FALSE	FALSE	FALSE	I9_PULMEM	0.798966	0.018745	pulmonary	TRUE	reported	textfile	6	1.07E+08	0.002863	3.39E-11	474223	eosinophi	TRUE	reported	igd	9.28E-05	44.02302	2	TRUE
rs556063	G	A	G	A	0.012802	-0.00231	0.573536	0.640433	FALSE	FALSE	FALSE	I9_PULMEM	0.882678	0.015621	pulmonary	TRUE	reported	textfile	1	1.11E+08	0.002003	1.68E-10	472834	eosinophi	TRUE	reported	igd	8.64E-05	40.84999	2	TRUE
rs5586852	A	G	A	G	0.013148	0.008909	0.605887	0.629847	FALSE	FALSE	FALSE	I9_PULMEM	0.565554	0.015505	pulmonary	TRUE	reported	textfile	17	7170665	0.002031	9.91E-11	474206	eosinophi	TRUE	reported	igd	8.84E-05	41.90807	2	TRUE
rs5587974	T	C	T	C	0.077662	0.013663	0.066369	0.035812	FALSE	FALSE	FALSE	I9_PULMEM	0.737026	0.040689	pulmonary	TRUE	reported	textfile	7	75470364	0.004051	7.07E-82	474208	eosinophi	TRUE	reported	igd	0.000774	367.5283	2	TRUE
rs5595521	C	A	C	A	0.031363	0.011949	0.07362	0.106582	FALSE	FALSE	FALSE	I9_PULMEM	0.623847	0.024365	pulmonary	TRUE	reported	textfile	22	50656109	0.003822	2.42E-16	473289	eosinophi	TRUE	reported	igd	0.000142	67.33673	2	TRUE
rs5611772	A	T	A	T	-0.10441	0.027517	0.071639	0.105442	FALSE	TRUE	FALSE	I9_PULMEM	0.265243	0.024699	pulmonary	TRUE	reported	textfile	21	36398586	0.003874	########	474236	eosinophi	TRUE	reported	igd	0.001529	726.3231	2	TRUE
rs5617956	A	G	A	G	0.017858	-0.01432	0.389076	0.354216	FALSE	FALSE	FALSE	I9_PULMEM	0.363192	0.015751	pulmonary	TRUE	reported	textfile	7	1.3E+08	0.002055	3.87E-18	474225	eosinophi	TRUE	reported	igd	0.000159	75.5162	2	TRUE
rs5619533	A	G	A	G	-0.03007	0.021221	0.057715	0.026582	FALSE	FALSE	FALSE	I9_PULMEM	0.648657	0.046576	pulmonary	TRUE	reported	textfile	7	8107922	0.004266	1.88E-12	471423	eosinophi	TRUE	reported	igd	0.000105	49.67816	2	TRUE

rs5633046	C	T	C	T	0.039484	-0.02136	0.55326	0.627587	FALSE	FALSE	FALSE	I9_PULMEM	0.168304	0.015507	pulmonary	TRUE	reported	textfile	5	1.48E+08	0.002008	5.38E-86	474231	eosinophi	TRUE	reported	igd	0.000815	386.6456	2	TRUE
rs574183	G	A	G	A	-0.01381	0.004959	0.388961	0.389608	FALSE	FALSE	FALSE	I9_PULMEM	0.74692	0.015367	pulmonary	TRUE	reported	textfile	8	95973816	0.002036	1.20E-11	473147	eosinophi	TRUE	reported	igd	9.73E-05	46.02763	2	TRUE
rs5747308	C	A	C	A	0.015575	-0.00742	0.504517	0.497656	FALSE	FALSE	FALSE	I9_PULMEM	0.620366	0.014983	pulmonary	TRUE	reported	textfile	22	18133500	0.001987	4.82E-15	474226	eosinophi	TRUE	reported	igd	0.00013	61.44104	2	TRUE
rs5763347	G	A	G	A	-0.02465	0.003742	0.121715	0.163741	FALSE	FALSE	FALSE	I9_PULMEM	0.854649	0.020429	pulmonary	TRUE	reported	textfile	18	46469650	0.003048	6.36E-16	474235	eosinophi	TRUE	reported	igd	0.000138	65.40367	2	TRUE
rs5783478	A	T	A	T	-0.05829	0.019796	0.244809	0.338025	FALSE	TRUE	FALSE	I9_PULMEM	0.211212	0.015833	pulmonary	TRUE	reported	textfile	7	20502828	0.002306	########	474226	eosinophi	TRUE	reported	igd	0.001345	638.8416	2	TRUE
rs5874511	A	G	A	G	-0.0152	0.008431	0.391451	0.309327	FALSE	FALSE	FALSE	I9_PULMEM	0.603543	0.016234	pulmonary	TRUE	reported	textfile	17	33803592	0.002034	8.21E-14	473122	eosinophi	TRUE	reported	igd	0.000118	55.85224	2	TRUE
rs5883393	T	C	T	C	-0.02336	0.010979	0.112968	0.152998	FALSE	FALSE	FALSE	I9_PULMEM	0.599538	0.02091	pulmonary	TRUE	reported	textfile	11	2325997	0.003139	1.03E-13	473290	eosinophi	TRUE	reported	igd	0.000117	55.39528	2	TRUE
rs6017541	A	G	A	G	-0.02978	0.004695	0.166741	0.152508	FALSE	FALSE	FALSE	I9_PULMEM	0.821565	0.020818	pulmonary	TRUE	reported	textfile	22	37311858	0.002681	1.25E-28	473297	eosinophi	TRUE	reported	igd	0.000261	123.3909	2	TRUE
rs6060000	G	T	G	T	0.042906	-0.00617	0.100334	0.102224	FALSE	FALSE	FALSE	I9_PULMEM	0.80351	0.024787	pulmonary	TRUE	reported	textfile	7	37382465	0.003317	3.05E-38	474233	eosinophi	TRUE	reported	igd	0.000353	167.3182	2	TRUE
rs6064639	A	G	A	G	-0.02404	0.006542	0.065569	0.092353	FALSE	FALSE	FALSE	I9_PULMEM	0.799971	0.025818	pulmonary	TRUE	reported	textfile	5	1.35E+08	0.004027	2.45E-09	474235	eosinophi	TRUE	reported	igd	7.51E-05	35.63129	2	TRUE
rs6103572	C	T	C	T	-0.02406	-0.0156	0.729098	0.716558	FALSE	FALSE	FALSE	I9_PULMEM	0.348534	0.016645	pulmonary	TRUE	reported	textfile	20	42657862	0.002237	5.96E-27	473156	eosinophi	TRUE	reported	igd	0.000244	115.6894	2	TRUE
rs6139104	T	A	T	A	-0.01939	0.01114	0.092532	0.086215	FALSE	TRUE	FALSE	I9_PULMEM	0.674834	0.026553	pulmonary	TRUE	reported	textfile	20	390310	0.003441	1.78E-08	470468	eosinophi	TRUE	reported	igd	6.75E-05	31.75953	2	TRUE
rs6141755	T	G	T	G	-0.01682	-0.00735	0.236009	0.31624	FALSE	FALSE	FALSE	I9_PULMEM	0.64867	0.016124	pulmonary	TRUE	reported	textfile	20	31163565	0.002348	8.30E-13	474229	eosinophi	TRUE	reported	igd	0.000108	51.29783	2	TRUE
rs6142639	C	G	C	G	-0.02458	0.00893	0.067297	0.047938	FALSE	TRUE	FALSE	I9_PULMEM	0.799371	0.035133	pulmonary	TRUE	reported	textfile	16	9053519	0.003983	6.99E-10	473155	eosinophi	TRUE	reported	igd	8.05E-05	38.09009	2	TRUE
rs6179883	C	T	C	T	0.014532	-0.01208	0.17025	0.207037	FALSE	FALSE	FALSE	I9_PULMEM	0.513917	0.018513	pulmonary	TRUE	reported	textfile	1	92013622	0.002639	3.75E-08	474235	eosinophi	TRUE	reported	igd	6.39E-05	30.32286	2	TRUE
rs6200503	T	C	T	C	-0.01661	0.051818	0.123915	0.077447	FALSE	FALSE	FALSE	I9_PULMEM	0.065035	0.028085	pulmonary	TRUE	reported	textfile	15	60725339	0.003028	4.26E-08	474184	eosinophi	TRUE	reported	igd	6.34E-05	30.07213	2	TRUE
rs6200617	A	G	A	G	-0.03754	0.101415	0.041969	0.036538	FALSE	FALSE	FALSE	I9_PULMEM	0.011777	0.040264	pulmonary	TRUE	reported	textfile	15	38904527	0.004973	4.62E-14	474236	eosinophi	TRUE	reported	igd	0.00012	56.97144	2	TRUE
rs6201128	G	A	G	A	-0.01269	-0.04275	0.344259	0.387655	FALSE	FALSE	FALSE	I9_PULMEM	0.005481	0.015393	pulmonary	TRUE	reported	textfile	15	63791228	0.002091	1.32E-09	474229	eosinophi	TRUE	reported	igd	7.77E-05	36.83099	2	TRUE
rs6206173	G	A	G	A	-0.0323	0.032994	0.229432	0.086234	FALSE	FALSE	FALSE	I9_PULMEM	0.217172	0.026735	pulmonary	TRUE	reported	textfile	17	44018399	0.002364	1.92E-42	474233	eosinophi	TRUE	reported	igd	0.000393	186.6383	2	TRUE
rs6208690	C	T	C	T	0.030422	0.025532	0.231992	0.280931	FALSE	FALSE	FALSE	I9_PULMEM	0.127652	0.01676	pulmonary	TRUE	reported	textfile	17	66016006	0.00238	2.27E-37	473295	eosinophi	TRUE	reported	igd	0.000345	163.3879	2	TRUE
rs6210548	T	C	T	C	-0.03025	0.068605	0.052894	0.015831	FALSE	FALSE	FALSE	I9_PULMEM	0.249778	0.05961	pulmonary	TRUE	reported	textfile	2	8757326	0.004452	1.13E-11	474234	eosinophi	TRUE	reported	igd	9.73E-05	46.15248	2	TRUE
rs6211716	A	G	A	G	-0.04757	0.042551	0.045256	0.028834	FALSE	FALSE	FALSE	I9_PULMEM	0.336023	0.04423	pulmonary	TRUE	reported	textfile	19	45232161	0.00477	2.15E-23	474231	eosinophi	TRUE	reported	igd	0.00021	99.46779	2	TRUE
rs6218399	T	C	T	C	0.026904	0.095981	0.050879	0.041677	FALSE	FALSE	FALSE	I9_PULMEM	0.010249	0.037386	pulmonary	TRUE	reported	textfile	2	2.05E+08	0.004517	2.65E-09	473295	eosinophi	TRUE	reported	igd	7.49E-05	35.47576	2	TRUE
rs6230811	T	G	T	G	0.015386	-0.03857	0.221457	0.200039	FALSE	FALSE	FALSE	I9_PULMEM	0.039539	0.018734	pulmonary	TRUE	reported	textfile	4	57738491	0.0024	1.50E-10	473298	eosinophi	TRUE	reported	igd	8.68E-05	41.09861	2	TRUE
rs6238550	A	T	A	T	-0.01443	-0.01903	0.308103	0.228439	FALSE	TRUE	FALSE	I9_PULMEM	0.287264	0.017882	pulmonary	TRUE	reported	textfile	5	1.72E+08	0.002142	1.68E-11	474226	eosinophi	TRUE	reported	igd	9.57E-05	45.364	2	TRUE
rs6239583	C	G	C	G	0.036768	0.046209	0.043064	0.044523	FALSE	TRUE	FALSE	I9_PULMEM	0.199797	0.036041	pulmonary	TRUE	reported	textfile	6	31807652	0.004882	5.27E-14	472377	eosinophi	TRUE	reported	igd	0.00012	56.72084	2	TRUE
rs6240822	G	A	G	A	-0.04218	0.026375	0.350368	0.250498	FALSE	FALSE	FALSE	I9_PULMEM	0.127364	0.0173	pulmonary	TRUE	reported	textfile	6	90955995	0.002073	5.69E-92	474234	eosinophi	TRUE	reported	igd	0.000872	413.9922	2	TRUE
rs6247372	G	A	G	A	0.014247	-0.01897	0.317118	0.329228	FALSE	FALSE	FALSE	I9_PULMEM	0.235538	0.015992	pulmonary	TRUE	reported	textfile	7	77254547	0.002136	2.64E-11	473140	eosinophi	TRUE	reported	igd	9.40E-05	44.48796	2	TRUE
rs6253915	A	G	A	G	0.027464	0.039863	0.097259	0.118651	FALSE	FALSE	FALSE	I9_PULMEM	0.086038	0.023221	pulmonary	TRUE	reported	textfile	8	48677930	0.003402	7.20E-16	474234	eosinophi	TRUE	reported	igd	0.000137	65.17141	2	TRUE
rs634534	G	A	G	A	0.032917	0.019769	0.542475	0.587997	FALSE	FALSE	FALSE	I9_PULMEM	0.196914	0.01532	pulmonary	TRUE	reported	textfile	11	65665256	0.001998	5.83E-61	473291	eosinophi	TRUE	reported	igd	0.000573	271.4237	2	TRUE
rs637064	T	C	T	C	0.024251	-0.03034	0.556128	0.622102	FALSE	FALSE	FALSE	I9_PULMEM	0.049931	0.015476	pulmonary	TRUE	reported	textfile	11	1.08E+08	0.001994	5.43E-34	474235	eosinophi	TRUE	reported	igd	0.000312	147.9133	2	TRUE
rs6490291	A	T	A	T	-0.06608	-0.02622	0.964087	0.95538	FALSE	TRUE	FALSE	I9_PULMEM	0.471417	0.036403	pulmonary	TRUE	reported	textfile	12	1.12E+08	0.005837	1.11E-29	448759	eosinophi	TRUE	reported	igd	0.000286	128.1774	2	TRUE
rs6496717	T	C	T	C	0.018867	-0.04129	0.759996	0.732278	FALSE	FALSE	FALSE	I9_PULMEM	0.014596	0.016908	pulmonary	TRUE	reported	textfile	15	91181567	0.00232	4.42E-16	474237	eosinophi	TRUE	reported	igd	0.000139	66.13447	2	TRUE
rs6556313	G	A	G	A	0.019467	-0.01957	0.332338	0.360541	FALSE	FALSE	FALSE	I9_PULMEM	0.210494	0.015627	pulmonary	TRUE	reported	textfile	5	1.77E+08	0.002102	2.16E-20	474227	eosinophi	TRUE	reported	igd	0.000181	85.76909	2	TRUE
rs6573020	T	C	T	C	0.020574	-0.00304	0.433436	0.529381	FALSE	FALSE	FALSE	I9_PULMEM	0.840455	0.015083	pulmonary	TRUE	reported	textfile	14	55871452	0.002019	2.31E-24	474234	eosinophi	TRUE	reported	igd	0.000219	103.8396	2	TRUE
rs6670664	A	T	A	T	-0.01507	0.008618	0.662541	0.598873	FALSE	TRUE	FALSE	I9_PULMEM	0.573282	0.015302	pulmonary	TRUE	reported	textfile	1	26531956	0.002099	7.38E-13	474234	eosinophi	TRUE	reported	igd	0.000109	51.51921	2	TRUE
rs6673532	G	A	G	A	-0.01381	-0.02104	0.220762	0.302887	FALSE	FALSE	FALSE	I9_PULMEM	0.19794	0.01634	pulmonary	TRUE	reported	textfile	5	86298649	0.002386	7.30E-09	473155	eosinophi	TRUE	reported	igd	7.08E-05	33.50969	2	TRUE
rs668248	C	G	C	G	-0.0165	0.023128	0.61554	0.678431	FALSE	TRUE	FALSE	I9_PULMEM	0.150231	0.016075	pulmonary	TRUE	reported	textfile	11	1.29E+08	0.002036	5.49E-16	474234	eosinophi	TRUE	reported	igd	0.000138	65.68454	2	TRUE
rs6684992	T	A	T	A	0.037659	0.009627	0.119465	0.131712	FALSE	TRUE	FALSE	I9_PULMEM	0.665624	0.022277	pulmonary	TRUE	reported	textfile	1	87752000	0.003052	6.06E-35	474233	eosinophi	TRUE	reported	igd	0.000321	152.2533	2	TRUE
rs6731125	C	T	C	T	0.017166	-0.02002	0.564761	0.59915	FALSE	FALSE	FALSE	I9_PULMEM	0.190877	0.015306	pulmonary	TRUE	reported	textfile	2	1.82E+08	0.002002	1.04E-17	473296	eosinophi	TRUE	reported	igd	0.000155	73.52046	2	TRUE
rs6741307	T	C	T	C	0.014022	0.025814	0.639529	0.668764	FALSE	FALSE	FALSE	I9_PULMEM	0.104782	0.015914	pulmonary	TRUE	reported	textfile	2	1.97E+08	0.002144	6.32E-11	435514	eosinophi	TRUE	reported	igd	9.82E-05	42.77287	2	TRUE
rs6750754	G	T	G	T	-0.06398	-0.00462	0.264203	0.270452	FALSE	FALSE	FALSE	I9_PULMEM	0.785095	0.016945	pulmonary	TRUE	reported	textfile	2	2.14E+08	0.002243	########	473293	eosinophi	TRUE	reported	igd	0.001716	813.5558	2	TRUE
rs6785619	G	C	G	C	0.023655	-0.03548	0.308431	0.338045	FALSE	TRUE	FALSE	I9_PULMEM	0.026044	0.015941	pulmonary	TRUE	reported	textfile	14	93024616	0.002175	1.63E-27	474230	eosinophi	TRUE	reported	igd	0.000249	118.2839	2	TRUE
rs6787336	A	G	A	G	0.036335	-0.03271	0.287462	0.264093	FALSE	FALSE	FALSE	I9_PULMEM	0.05658	0.017157	pulmonary	TRUE	reported	textfile	3	3153194	0.002238	3.29E-59	473154	eosinophi	TRUE	reported	igd	0.000557	263.5896	2	TRUE

rs6904506	C	T	C	T	-0.05238	0.039864	0.088477	0.069856	FALSE	FALSE	FALSE	I9_PULMEN	0.175661	0.029437	pulmonary	TRUE	reported	textfile	6	245096	0.003544	2.25E-49	471426	eosinophi	TRUE	reported	igd	0.000463	218.4448	2	TRUE
rs6924350	C	A	C	A	0.050023	0.051554	0.18447	0.22119	FALSE	FALSE	FALSE	I9_PULMEN	0.00416	0.01799	pulmonary	TRUE	reported	textfile	6	31339556	0.002537	1.76E-86	471739	eosinophi	TRUE	reported	igd	0.000823	388.7735	2	TRUE
rs6924387	G	A	G	A	0.016659	-0.02456	0.410173	0.432156	FALSE	FALSE	FALSE	I9_PULMEN	0.106464	0.015212	pulmonary	TRUE	reported	textfile	6	1.37E+08	0.002027	2.19E-16	474233	eosinophi	TRUE	reported	igd	0.000142	67.54427	2	TRUE
rs6930635	C	T	C	T	-0.03771	-0.03539	0.078494	0.076552	FALSE	FALSE	FALSE	I9_PULMEN	0.209899	0.028223	pulmonary	TRUE	reported	textfile	6	395634	0.003739	6.83E-24	470025	eosinophi	TRUE	reported	igd	0.000216	101.7294	2	TRUE
rs6956283	T	C	T	C	-0.01568	0.016794	0.732752	0.771448	FALSE	FALSE	FALSE	I9_PULMEN	0.347411	0.017873	pulmonary	TRUE	reported	textfile	7	98756597	0.002237	2.48E-12	474222	eosinophi	TRUE	reported	igd	0.000104	49.13131	2	TRUE
rs6971710	A	G	A	G	0.028325	0.030162	0.201035	0.217458	FALSE	FALSE	FALSE	I9_PULMEN	0.097203	0.018186	pulmonary	TRUE	reported	textfile	7	3140173	0.002475	2.75E-30	474215	eosinophi	TRUE	reported	igd	0.000276	130.9748	2	TRUE
rs6979947	G	A	G	A	0.013224	-0.01823	0.266399	0.260582	FALSE	FALSE	FALSE	I9_PULMEN	0.287171	0.017127	pulmonary	TRUE	reported	textfile	7	1.57E+08	0.002242	3.75E-09	472836	eosinophi	TRUE	reported	igd	7.36E-05	34.78986	2	TRUE
rs6986109	T	G	T	G	-0.01649	-0.00091	0.704331	0.752144	FALSE	FALSE	FALSE	I9_PULMEN	0.958462	0.017407	pulmonary	TRUE	reported	textfile	8	81286298	0.002181	4.24E-14	474234	eosinophi	TRUE	reported	igd	0.00012	57.137	2	TRUE
rs6989099	C	T	C	T	-0.01776	0.011482	0.317295	0.275488	FALSE	FALSE	FALSE	I9_PULMEN	0.493934	0.016785	pulmonary	TRUE	reported	textfile	8	66898262	0.002133	8.58E-17	474231	eosinophi	TRUE	reported	igd	0.000146	69.35042	2	TRUE
rs699664	T	C	T	C	-0.01565	-0.0246	0.329576	0.31668	FALSE	FALSE	FALSE	I9_PULMEN	0.128073	0.016165	pulmonary	TRUE	reported	textfile	2	85780536	0.002102	1.00E-13	472834	eosinophi	TRUE	reported	igd	0.000117	55.46754	2	TRUE
rs6999452	A	G	A	G	0.011632	-0.00897	0.517717	0.629742	FALSE	FALSE	FALSE	I9_PULMEN	0.563747	0.015535	pulmonary	TRUE	reported	textfile	8	1.06E+08	0.001991	5.33E-09	472836	eosinophi	TRUE	reported	igd	7.22E-05	34.13221	2	TRUE
rs7009579	G	A	G	A	-0.0122	-0.00375	0.428946	0.482732	FALSE	FALSE	FALSE	I9_PULMEN	0.802579	0.014986	pulmonary	TRUE	reported	textfile	8	81064734	0.002006	1.22E-09	474235	eosinophi	TRUE	reported	igd	7.80E-05	37.00578	2	TRUE
rs7026022	C	A	C	A	0.012099	0.000224	0.377752	0.335595	FALSE	FALSE	FALSE	I9_PULMEN	0.988746	0.015875	pulmonary	TRUE	reported	textfile	9	1.03E+08	0.002045	3.38E-09	472833	eosinophi	TRUE	reported	igd	7.40E-05	35.00342	2	TRUE
rs7080536	A	G	A	G	-0.04369	-0.01001	0.043176	0.027764	FALSE	FALSE	FALSE	I9_PULMEN	0.826704	0.045723	pulmonary	TRUE	reported	textfile	10	1.15E+08	0.004952	1.17E-18	472834	eosinophi	TRUE	reported	igd	0.000165	77.85392	2	TRUE
rs708776	T	G	T	G	0.026773	-0.01766	0.940532	0.957002	FALSE	FALSE	FALSE	I9_PULMEN	0.632595	0.036942	pulmonary	TRUE	reported	textfile	1	2.27E+08	0.004244	2.91E-10	472836	eosinophi	TRUE	reported	igd	8.42E-05	39.79619	2	TRUE
rs7123726	C	T	C	T	0.016914	-0.02455	0.210494	0.183063	FALSE	FALSE	FALSE	I9_PULMEN	0.206775	0.019444	pulmonary	TRUE	reported	textfile	11	1.19E+08	0.002436	3.95E-12	474213	eosinophi	TRUE	reported	igd	0.000102	48.2099	2	TRUE
rs7141943	G	A	G	A	0.015879	0.01314	0.394677	0.388817	FALSE	FALSE	FALSE	I9_PULMEN	0.393135	0.015388	pulmonary	TRUE	reported	textfile	14	25438250	0.002038	7.00E-15	473294	eosinophi	TRUE	reported	igd	0.000128	60.70663	2	TRUE
rs7142941	A	G	A	G	-0.01858	0.01655	0.196207	0.158854	FALSE	FALSE	FALSE	I9_PULMEN	0.422681	0.020641	pulmonary	TRUE	reported	textfile	13	42945821	0.002496	1.03E-13	474231	eosinophi	TRUE	reported	igd	0.000117	55.38175	2	TRUE
rs7150896	A	G	A	G	0.036234	-0.05698	0.025836	0.018092	FALSE	FALSE	FALSE	I9_PULMEN	0.315307	0.056746	pulmonary	TRUE	reported	textfile	10	65253869	0.006364	1.28E-08	474233	eosinophi	TRUE	reported	igd	6.84E-05	32.41682	2	TRUE
rs7158239	A	G	A	G	0.01556	-0.01037	0.395176	0.408931	FALSE	FALSE	FALSE	I9_PULMEN	0.497253	0.015276	pulmonary	TRUE	reported	textfile	14	65520616	0.002037	2.28E-14	474233	eosinophi	TRUE	reported	igd	0.000123	58.34925	2	TRUE
rs7162818	T	C	T	C	0.021187	0.05283	0.094165	0.076521	FALSE	FALSE	FALSE	I9_PULMEN	0.061943	0.028301	pulmonary	TRUE	reported	textfile	1	1.61E+08	0.003449	8.32E-10	474235	eosinophi	TRUE	reported	igd	7.96E-05	37.73555	2	TRUE
rs7173571	C	T	C	T	0.013979	-0.01427	0.526604	0.5095	FALSE	FALSE	FALSE	I9_PULMEN	0.342722	0.01504	pulmonary	TRUE	reported	textfile	15	41712807	0.001993	2.40E-12	474233	eosinophi	TRUE	reported	igd	0.000104	49.19668	2	TRUE
rs7215391	T	C	T	C	-0.01267	-0.0157	0.257019	0.203759	FALSE	FALSE	FALSE	I9_PULMEN	0.397983	0.018579	pulmonary	TRUE	reported	textfile	17	64232022	0.002272	2.49E-08	474232	eosinophi	TRUE	reported	igd	6.56E-05	31.10798	2	TRUE
rs7220649	T	C	T	C	-0.02822	0.04129	0.043218	0.021314	FALSE	FALSE	FALSE	I9_PULMEN	0.425513	0.051813	pulmonary	TRUE	reported	textfile	17	76331757	0.004951	1.23E-08	469930	eosinophi	TRUE	reported	igd	6.91E-05	32.49516	2	TRUE
rs725613	G	T	G	T	-0.04745	-0.00018	0.355982	0.33014	FALSE	FALSE	FALSE	I9_PULMEN	0.990849	0.016009	pulmonary	TRUE	reported	textfile	16	11169683	0.002071	########	472836	eosinophi	TRUE	reported	igd	0.001109	524.8524	2	TRUE
rs7257	A	G	A	G	0.033603	0.010363	0.567153	0.535979	FALSE	FALSE	FALSE	I9_PULMEN	0.492258	0.015091	pulmonary	TRUE	reported	textfile	15	80191343	0.002002	3.60E-63	473776	eosinophi	TRUE	reported	igd	0.000594	281.7255	2	TRUE
rs7267752	C	T	C	T	0.020618	0.04384	0.083489	0.060801	FALSE	FALSE	FALSE	I9_PULMEN	0.160431	0.031233	pulmonary	TRUE	reported	textfile	20	17755245	0.003599	1.04E-08	473297	eosinophi	TRUE	reported	igd	6.93E-05	32.81917	2	TRUE
rs7269558	A	G	A	G	-0.05009	0.013701	0.02972	0.038607	FALSE	FALSE	FALSE	I9_PULMEN	0.724488	0.03887	pulmonary	TRUE	reported	textfile	9	6633664	0.005908	2.40E-17	474232	eosinophi	TRUE	reported	igd	0.000152	71.88759	2	TRUE
rs7276663	A	C	A	C	-0.02431	-0.0526	0.164331	0.203268	FALSE	FALSE	FALSE	I9_PULMEN	0.005277	0.018855	pulmonary	TRUE	reported	textfile	9	1.37E+08	0.002673	9.94E-20	474228	eosinophi	TRUE	reported	igd	0.000174	82.71912	2	TRUE
rs7280084	A	G	A	G	-0.01581	-0.01011	0.249931	0.234472	FALSE	FALSE	FALSE	I9_PULMEN	0.569392	0.017769	pulmonary	TRUE	reported	textfile	16	31014247	0.002293	5.63E-12	474232	eosinophi	TRUE	reported	igd	0.0001	47.52738	2	TRUE
rs7283475	T	C	T	C	-0.07174	0.011468	0.01273	0.018407	FALSE	FALSE	FALSE	I9_PULMEN	0.839129	0.056492	pulmonary	TRUE	reported	textfile	10	64559465	0.009325	1.50E-14	474234	eosinophi	TRUE	reported	igd	0.000125	59.19153	2	TRUE
rs7298704	A	C	A	C	-0.02163	0.017738	0.126171	0.152571	FALSE	FALSE	FALSE	I9_PULMEN	0.395279	0.020866	pulmonary	TRUE	reported	textfile	19	3251116	0.00298	4.07E-13	474232	eosinophi	TRUE	reported	igd	0.000111	52.69374	2	TRUE
rs7304603	G	T	G	T	-0.02155	0.014331	0.116838	0.101889	FALSE	FALSE	FALSE	I9_PULMEN	0.562618	0.024753	pulmonary	TRUE	reported	textfile	3	1.72E+08	0.003092	3.29E-12	474234	eosinophi	TRUE	reported	igd	0.000102	48.58412	2	TRUE
rs7307249	C	T	C	T	0.060029	-0.13461	0.009171	0.007035	FALSE	FALSE	FALSE	I9_PULMEN	0.14299	0.091899	pulmonary	TRUE	reported	textfile	3	50783442	0.010455	9.63E-09	473800	eosinophi	TRUE	reported	igd	6.96E-05	32.96646	2	TRUE
rs7308654	A	C	A	C	-0.01179	-0.00367	0.395121	0.483346	FALSE	FALSE	FALSE	I9_PULMEN	0.806765	0.015005	pulmonary	TRUE	reported	textfile	7	20376103	0.002035	7.07E-09	474232	eosinophi	TRUE	reported	igd	7.08E-05	33.5601	2	TRUE
rs7311883	C	T	C	T	-0.02937	-0.00422	0.08553	0.210798	FALSE	FALSE	FALSE	I9_PULMEN	0.81943	0.018468	pulmonary	TRUE	reported	textfile	7	50764948	0.003538	1.08E-16	474236	eosinophi	TRUE	reported	igd	0.000145	68.90191	2	TRUE
rs7319184	G	T	G	T	0.053919	0.332078	0.021394	0.0022	FALSE	FALSE	FALSE	I9_PULMEN	0.037227	0.159403	pulmonary	TRUE	reported	textfile	12	1.11E+08	0.006882	4.90E-15	471441	eosinophi	TRUE	reported	igd	0.00013	61.38362	2	TRUE
rs7320514	C	T	C	T	0.037235	0.020032	0.104781	0.098116	FALSE	FALSE	FALSE	I9_PULMEN	0.429314	0.025345	pulmonary	TRUE	reported	textfile	3	1.28E+08	0.003255	2.91E-30	473296	eosinophi	TRUE	reported	igd	0.000276	130.8576	2	TRUE
rs7323288	C	T	C	T	0.067388	0.004232	0.213178	0.17532	FALSE	FALSE	FALSE	I9_PULMEN	0.830156	0.019729	pulmonary	TRUE	reported	textfile	4	38664131	0.002418	########	474216	eosinophi	TRUE	reported	igd	0.001635	776.6954	2	TRUE
rs7323820	T	C	T	C	-0.01649	-0.00895	0.179352	0.17677	FALSE	FALSE	FALSE	I9_PULMEN	0.648294	0.019624	pulmonary	TRUE	reported	textfile	3	1.42E+08	0.002573	1.52E-10	474236	eosinophi	TRUE	reported	igd	8.66E-05	41.05341	2	TRUE
rs7327284	A	G	A	G	-0.02535	0.032599	0.122779	0.129443	FALSE	FALSE	FALSE	I9_PULMEN	0.145769	0.022411	pulmonary	TRUE	reported	textfile	5	1.5E+08	0.003019	4.84E-17	474234	eosinophi	TRUE	reported	igd	0.000149	70.52854	2	TRUE
rs7327960	C	T	C	T	0.017659	-0.02026	0.826621	0.861398	FALSE	FALSE	FALSE	I9_PULMEN	0.354834	0.021899	pulmonary	TRUE	reported	textfile	13	74682892	0.002629	1.91E-11	474235	eosinophi	TRUE	reported	igd	9.51E-05	45.11793	2	TRUE
rs7332287	T	C	T	C	0.014569	-0.04592	0.241917	0.196658	FALSE	FALSE	FALSE	I9_PULMEN	0.014851	0.018851	pulmonary	TRUE	reported	textfile	20	62694105	0.002327	3.91E-10	473153	eosinophi	TRUE	reported	igd	8.28E-05	39.19809	2	TRUE
rs7342883	T	C	T	C	0.029847	0.033869	0.074788	0.108412	FALSE	FALSE	FALSE	I9_PULMEN	0.160928	0.024158	pulmonary	TRUE	reported	textfile	6	42255274	0.003774	2.72E-15	474234	eosinophi	TRUE	reported	igd	0.000132	62.54545	2	TRUE

rs7382061	C	T	C	T	-0.05458	-0.02372	0.590665	0.518445	FALSE	FALSE	FALSE	I9_PULMEM	0.113232	0.014975	pulmonary	TRUE	reported	textfile	6	30047965	0.002014	########	472694	eosinophi	TRUE	reported	igd	0.001551	734.3961	2	TRUE
rs7396371	T	C	T	C	0.022097	-0.00095	0.210821	0.332309	FALSE	FALSE	FALSE	I9_PULMEM	0.952765	0.015992	pulmonary	TRUE	reported	textfile	18	60874413	0.002465	3.28E-19	472214	eosinophi	TRUE	reported	igd	0.00017	80.35834	2	TRUE
rs7423615	T	C	T	C	0.023183	-0.01235	0.187322	0.131126	FALSE	FALSE	FALSE	I9_PULMEM	0.579077	0.022263	pulmonary	TRUE	reported	textfile	2	2.31E+08	0.002529	5.10E-20	473289	eosinophi	TRUE	reported	igd	0.000178	84.03105	2	TRUE
rs743002	C	T	C	T	-0.05886	-0.01415	0.065649	0.039801	FALSE	FALSE	FALSE	I9_PULMEM	0.712676	0.038419	pulmonary	TRUE	reported	textfile	22	41404939	0.00403	2.90E-48	474235	eosinophi	TRUE	reported	igd	0.00045	213.2968	2	TRUE
rs7433176	A	G	A	G	0.019483	0.015714	0.098145	0.069518	FALSE	FALSE	FALSE	I9_PULMEM	0.599512	0.029925	pulmonary	TRUE	reported	textfile	16	2161793	0.003368	7.49E-09	474231	eosinophi	TRUE	reported	igd	7.06E-05	33.46306	2	TRUE
rs7441808	G	A	G	A	0.013917	0.008747	0.301163	0.282016	FALSE	FALSE	FALSE	I9_PULMEM	0.599616	0.016662	pulmonary	TRUE	reported	textfile	4	26090375	0.002161	1.22E-10	474232	eosinophi	TRUE	reported	igd	8.74E-05	41.47438	2	TRUE
rs7448010	A	G	A	G	-0.08302	-0.00828	0.042751	0.020697	FALSE	FALSE	FALSE	I9_PULMEM	0.87467	0.052526	pulmonary	TRUE	reported	textfile	17	7742601	0.00494	2.52E-63	472379	eosinophi	TRUE	reported	igd	0.000598	282.4157	2	TRUE
rs7461209	A	T	A	T	0.060952	-0.07674	0.063075	0.068521	FALSE	TRUE	FALSE	I9_PULMEM	0.010397	0.02995	pulmonary	TRUE	reported	textfile	9	1.36E+08	0.004113	1.23E-49	473293	eosinophi	TRUE	reported	igd	0.000464	219.6123	2	TRUE
rs746550	T	C	T	C	0.023016	0.006606	0.235899	0.138813	FALSE	FALSE	FALSE	I9_PULMEM	0.760631	0.021683	pulmonary	TRUE	reported	textfile	4	1.24E+08	0.002342	9.03E-23	474233	eosinophi	TRUE	reported	igd	0.000204	96.57931	2	TRUE
rs7556106	A	G	A	G	-0.03331	-0.00441	0.064255	0.053659	FALSE	FALSE	FALSE	I9_PULMEM	0.894657	0.033272	pulmonary	TRUE	reported	textfile	1	28589008	0.004033	1.57E-16	474224	eosinophi	TRUE	reported	igd	0.000144	68.20036	2	TRUE
rs7569084	T	C	T	C	0.018707	0.00136	0.584311	0.646012	FALSE	FALSE	FALSE	I9_PULMEM	0.930924	0.015694	pulmonary	TRUE	reported	textfile	2	65656969	0.002006	1.19E-20	472837	eosinophi	TRUE	reported	igd	0.000184	86.96502	2	TRUE
rs7573721	A	C	A	C	0.051583	0.024323	0.013709	0.071349	FALSE	FALSE	FALSE	I9_PULMEM	0.405914	0.029266	pulmonary	TRUE	reported	textfile	18	45437715	0.008568	1.79E-09	472528	eosinophi	TRUE	reported	igd	7.67E-05	36.24536	2	TRUE
rs7591231	G	C	G	C	0.01319	0.001405	0.237633	0.176914	FALSE	TRUE	FALSE	I9_PULMEM	0.94288	0.019607	pulmonary	TRUE	reported	textfile	2	54829596	0.002318	1.30E-08	474233	eosinophi	TRUE	reported	igd	6.83E-05	32.37881	2	TRUE
rs7593992	C	T	C	T	0.03047	-0.01663	0.768386	0.769828	FALSE	FALSE	FALSE	I9_PULMEM	0.349768	0.017786	pulmonary	TRUE	reported	textfile	2	1.12E+08	0.002346	1.52E-38	472836	eosinophi	TRUE	reported	igd	0.000357	168.6891	2	TRUE
rs7636495	A	G	A	G	-0.02404	-0.02131	0.180819	0.187501	FALSE	FALSE	FALSE	I9_PULMEM	0.268719	0.019267	pulmonary	TRUE	reported	textfile	3	1.96E+08	0.002591	1.84E-20	472827	eosinophi	TRUE	reported	igd	0.000182	86.09311	2	TRUE
rs7646283	T	C	T	C	0.025996	-0.00796	0.367733	0.324799	FALSE	FALSE	FALSE	I9_PULMEM	0.620103	0.016067	pulmonary	TRUE	reported	textfile	3	33046480	0.002063	2.33E-36	474234	eosinophi	TRUE	reported	igd	0.000335	158.7862	2	TRUE
rs7646695	T	C	T	C	0.017597	0.003497	0.17966	0.180447	FALSE	FALSE	FALSE	I9_PULMEM	0.857922	0.019536	pulmonary	TRUE	reported	textfile	3	72172181	0.002617	1.82E-11	474229	eosinophi	TRUE	reported	igd	9.53E-05	45.21349	2	TRUE
rs7649812	G	C	G	C	0.016017	-0.02004	0.242796	0.280211	FALSE	TRUE	FALSE	I9_PULMEM	0.231886	0.016759	pulmonary	TRUE	reported	textfile	3	98449191	0.002304	3.74E-12	474237	eosinophi	TRUE	reported	igd	0.000102	48.32764	2	TRUE
rs7679317	T	C	T	C	-0.04168	-0.00612	0.091583	0.149843	FALSE	FALSE	FALSE	I9_PULMEM	0.770708	0.020995	pulmonary	TRUE	reported	textfile	19	46359794	0.003436	8.07E-34	474234	eosinophi	TRUE	reported	igd	0.00031	147.1174	2	TRUE
rs7683096	A	C	A	C	-0.02341	0.022544	0.117642	0.083445	FALSE	FALSE	FALSE	I9_PULMEM	0.40691	0.027183	pulmonary	TRUE	reported	textfile	3	1.6E+08	0.003083	3.27E-14	473155	eosinophi	TRUE	reported	igd	0.000122	57.64248	2	TRUE
rs7687708	G	T	G	T	-0.01471	0.006441	0.219981	0.204969	FALSE	FALSE	FALSE	I9_PULMEM	0.730861	0.018726	pulmonary	TRUE	reported	textfile	4	6906076	0.002434	1.56E-09	474231	eosinophi	TRUE	reported	igd	7.70E-05	36.51441	2	TRUE
rs7690837	G	A	G	A	-0.03227	-0.02282	0.036338	0.05026	FALSE	FALSE	FALSE	I9_PULMEM	0.509196	0.034577	pulmonary	TRUE	reported	textfile	2	1.63E+08	0.005559	6.57E-09	473298	eosinophi	TRUE	reported	igd	7.12E-05	33.70207	2	TRUE
rs7698158	G	C	G	C	-0.02608	-0.01704	0.047887	0.103138	FALSE	TRUE	FALSE	I9_PULMEM	0.4905	0.024719	pulmonary	TRUE	reported	textfile	4	38416046	0.004658	2.22E-08	474237	eosinophi	TRUE	reported	igd	6.61E-05	31.34352	2	TRUE
rs7700687	T	C	T	C	0.040023	-0.01629	0.617223	0.657032	FALSE	FALSE	FALSE	I9_PULMEM	0.301967	0.015783	pulmonary	TRUE	reported	textfile	5	1.41E+08	0.002035	4.82E-86	474213	eosinophi	TRUE	reported	igd	0.000815	386.8019	2	TRUE
rs7733133	A	G	A	G	0.038344	0.04192	0.020938	0.014055	FALSE	FALSE	FALSE	I9_PULMEM	0.516478	0.064613	pulmonary	TRUE	reported	textfile	11	75438737	0.006985	4.12E-08	473773	eosinophi	TRUE	reported	igd	6.36E-05	30.13424	2	TRUE
rs7762529	C	G	C	G	-0.04591	-0.06727	0.035282	0.019231	FALSE	TRUE	FALSE	I9_PULMEM	0.216966	0.054485	pulmonary	TRUE	reported	textfile	1	65362766	0.005607	2.79E-16	473297	eosinophi	TRUE	reported	igd	0.000142	67.04856	2	TRUE
rs7764278	T	G	T	G	0.01731	-0.00969	0.205108	0.202818	FALSE	FALSE	FALSE	I9_PULMEM	0.604116	0.018696	pulmonary	TRUE	reported	textfile	6	42157817	0.002455	1.84E-12	472836	eosinophi	TRUE	reported	igd	0.000105	49.71521	2	TRUE
rs778756	G	A	G	A	-0.01596	0.01483	0.579738	0.518506	FALSE	FALSE	FALSE	I9_PULMEM	0.324852	0.015063	pulmonary	TRUE	reported	textfile	2	61781994	0.002001	1.57E-15	474232	eosinophi	TRUE	reported	igd	0.000134	63.60853	2	TRUE
rs7797428	T	C	T	C	0.012392	0.008309	0.469244	0.4392	FALSE	FALSE	FALSE	I9_PULMEM	0.583998	0.015175	pulmonary	TRUE	reported	textfile	7	1.25E+08	0.001999	5.85E-10	473289	eosinophi	TRUE	reported	igd	8.12E-05	38.42867	2	TRUE
rs783646	C	G	C	G	-0.0188	0.014682	0.816656	0.718796	FALSE	TRUE	FALSE	I9_PULMEM	0.380529	0.016742	pulmonary	TRUE	reported	textfile	6	1.17E+08	0.002566	2.41E-13	474234	eosinophi	TRUE	reported	igd	0.000113	53.70137	2	TRUE
rs7839946	C	T	C	T	0.024301	-0.00151	0.056991	0.058522	FALSE	FALSE	FALSE	I9_PULMEM	0.962506	0.032065	pulmonary	TRUE	reported	textfile	8	22901943	0.004327	2.00E-08	473297	eosinophi	TRUE	reported	igd	6.66E-05	31.5408	2	TRUE
rs7840212	T	C	T	C	-0.0402	-0.00957	0.336961	0.295058	FALSE	FALSE	FALSE	I9_PULMEM	0.561031	0.01646	pulmonary	TRUE	reported	textfile	8	1.31E+08	0.00211	7.11E-81	472832	eosinophi	TRUE	reported	igd	0.000767	363.0545	2	TRUE
rs7846314	T	A	T	A	-0.03285	-0.00159	0.186954	0.194016	FALSE	TRUE	FALSE	I9_PULMEM	0.933401	0.01898	pulmonary	TRUE	reported	textfile	8	61650831	0.002543	3.92E-38	474237	eosinophi	TRUE	reported	igd	0.000352	166.8794	2	TRUE
rs7868130	T	C	T	C	0.03239	-0.02598	0.258801	0.323633	FALSE	FALSE	FALSE	I9_PULMEM	0.103743	0.01597	pulmonary	TRUE	reported	textfile	9	4998401	0.002269	3.35E-46	474228	eosinophi	TRUE	reported	igd	0.00043	203.7751	2	TRUE
rs7897422	C	T	C	T	0.020768	0.023883	0.192058	0.303821	FALSE	FALSE	FALSE	I9_PULMEM	0.143168	0.016312	pulmonary	TRUE	reported	textfile	10	90849704	0.002521	1.85E-16	473135	eosinophi	TRUE	reported	igd	0.000143	67.86437	2	TRUE
rs7971658	A	G	A	G	-0.02446	0.036537	0.126529	0.084122	FALSE	FALSE	FALSE	I9_PULMEM	0.177452	0.027091	pulmonary	TRUE	reported	textfile	2	1.44E+08	0.003003	3.99E-16	473296	eosinophi	TRUE	reported	igd	0.00014	66.31669	2	TRUE
rs7975	A	G	A	G	-0.01212	-0.01963	0.324166	0.390685	FALSE	FALSE	FALSE	I9_PULMEM	0.199913	0.015313	pulmonary	TRUE	reported	textfile	14	77793207	0.002124	1.17E-08	472834	eosinophi	TRUE	reported	igd	6.89E-05	32.57687	2	TRUE
rs7986796	T	G	T	G	0.024263	0.014782	0.6277	0.624894	FALSE	FALSE	FALSE	I9_PULMEM	0.340796	0.015517	pulmonary	TRUE	reported	textfile	13	40351064	0.002058	4.91E-32	474237	eosinophi	TRUE	reported	igd	0.000293	138.9941	2	TRUE
rs7988120	T	C	T	C	0.040325	-0.01207	0.35982	0.311588	FALSE	FALSE	FALSE	I9_PULMEM	0.456352	0.016201	pulmonary	TRUE	reported	textfile	5	1.1E+08	0.002063	4.72E-85	474235	eosinophi	TRUE	reported	igd	0.000805	382.0749	2	TRUE
rs8	T	C	T	C	0.021919	0.004041	0.204252	0.234304	FALSE	FALSE	FALSE	I9_PULMEM	0.820857	0.017847	pulmonary	TRUE	reported	textfile	7	92408329	0.002545	7.63E-18	473147	eosinophi	TRUE	reported	igd	0.000157	74.17611	2	TRUE
rs8005417	C	T	C	T	-0.08179	-0.06534	0.022394	0.008581	FALSE	FALSE	FALSE	I9_PULMEM	0.418274	0.080728	pulmonary	TRUE	reported	textfile	20	30294682	0.006746	8.57E-34	474237	eosinophi	TRUE	reported	igd	0.00031	146.9925	2	TRUE
rs8006620	T	C	T	C	-0.02413	-0.00946	0.067935	0.041717	FALSE	FALSE	FALSE	I9_PULMEM	0.800739	0.037472	pulmonary	TRUE	reported	textfile	2	26220710	0.003931	8.53E-10	474233	eosinophi	TRUE	reported	igd	7.95E-05	37.68901	2	TRUE
rs8012643	T	C	T	C	0.015461	-0.00641	0.286432	0.249126	FALSE	FALSE	FALSE	I9_PULMEM	0.711731	0.017342	pulmonary	TRUE	reported	textfile	14	37692864	0.002194	1.91E-12	474233	eosinophi	TRUE	reported	igd	0.000105	49.65924	2	TRUE
rs8020739	T	G	T	G	0.02471	0.011142	0.64383	0.679813	FALSE	FALSE	FALSE	I9_PULMEM	0.4877	0.016056	pulmonary	TRUE	reported	textfile	14	35882492	0.002087	2.57E-32	472205	eosinophi	TRUE	reported	igd	0.000297	140.1841	2	TRUE

rs8026803	C	T	C	T	-0.03126	0.009097	0.260856	0.223404	FALSE	FALSE	FALSE	I9_PULMEM	0.613609	0.018016	pulmonary	TRUE	reported	textfile	15	80260014	0.002259	1.67E-43	473295	eosinophi	TRUE	reported	igd	0.000404	191.4399	2	TRUE
rs8044920	T	C	T	C	0.012096	0.041401	0.376333	0.366249	FALSE	FALSE	FALSE	I9_PULMEM	0.007591	0.015508	pulmonary	TRUE	reported	textfile	16	69838676	0.002048	3.59E-09	474235	eosinophi	TRUE	reported	igd	7.36E-05	34.88364	2	TRUE
rs8050508	T	C	T	C	0.034941	0.001754	0.030896	0.089278	FALSE	FALSE	FALSE	I9_PULMEM	0.947201	0.026484	pulmonary	TRUE	reported	textfile	16	67303358	0.005716	1.01E-09	474234	eosinophi	TRUE	reported	igd	7.88E-05	37.36667	2	TRUE
rs8076052	C	A	C	A	-0.01834	0.041448	0.296433	0.222149	FALSE	FALSE	FALSE	I9_PULMEM	0.02113	0.017977	pulmonary	TRUE	reported	textfile	17	73779198	0.00218	4.33E-17	473295	eosinophi	TRUE	reported	igd	0.000149	70.73706	2	TRUE
rs8083368	A	G	A	G	0.015482	-0.01328	0.230071	0.291352	FALSE	FALSE	FALSE	I9_PULMEM	0.421672	0.016528	pulmonary	TRUE	reported	textfile	18	56219590	0.002364	5.99E-11	472833	eosinophi	TRUE	reported	igd	9.07E-05	42.89013	2	TRUE
rs8113367	G	A	G	A	0.017185	0.01635	0.632307	0.665577	FALSE	FALSE	FALSE	I9_PULMEM	0.305649	0.01596	pulmonary	TRUE	reported	textfile	19	18388250	0.002056	6.68E-17	474222	eosinophi	TRUE	reported	igd	0.000147	69.86361	2	TRUE
rs884634	C	T	C	T	0.012422	-0.00952	0.415032	0.41853	FALSE	FALSE	FALSE	I9_PULMEM	0.532361	0.015251	pulmonary	TRUE	reported	textfile	9	34861298	0.002012	6.83E-10	474235	eosinophi	TRUE	reported	igd	8.04E-05	38.11758	2	TRUE
rs900382	T	C	T	C	-0.05093	-0.01515	0.027281	0.05593	FALSE	FALSE	FALSE	I9_PULMEM	0.643238	0.0327	pulmonary	TRUE	reported	textfile	3	1.29E+08	0.006129	1.00E-16	474215	eosinophi	TRUE	reported	igd	0.000146	69.06126	2	TRUE
rs911603	A	C	A	C	-0.02468	0.008451	0.40355	0.46321	FALSE	FALSE	FALSE	I9_PULMEM	0.575678	0.015099	pulmonary	TRUE	reported	textfile	9	1.18E+08	0.002043	1.42E-33	474232	eosinophi	TRUE	reported	igd	0.000308	145.9442	2	TRUE
rs925966	G	C	G	C	-0.01829	0.015847	0.759472	0.675558	FALSE	TRUE	FALSE	I9_PULMEM	0.326102	0.016137	pulmonary	TRUE	reported	textfile	2	1.37E+08	0.002373	1.32E-14	472217	eosinophi	TRUE	reported	igd	0.000126	59.43845	2	TRUE
rs9349322	C	A	C	A	0.022029	0.00487	0.146915	0.203615	FALSE	FALSE	FALSE	I9_PULMEM	0.794253	0.018674	pulmonary	TRUE	reported	textfile	6	45634092	0.002803	4.04E-15	474236	eosinophi	TRUE	reported	igd	0.00013	61.76487	2	TRUE
rs9389268	G	A	G	A	-0.04537	0.03014	0.2561	0.339959	FALSE	FALSE	FALSE	I9_PULMEM	0.057666	0.015878	pulmonary	TRUE	reported	textfile	6	1.35E+08	0.002292	3.69E-87	474216	eosinophi	TRUE	reported	igd	0.000825	391.7861	2	TRUE
rs9392525	C	T	C	T	0.01321	-0.03128	0.57553	0.552924	FALSE	FALSE	FALSE	I9_PULMEM	0.039377	0.015181	pulmonary	TRUE	reported	textfile	6	435677	0.002024	6.95E-11	471424	eosinophi	TRUE	reported	igd	9.04E-05	42.59737	2	TRUE
rs941616	T	C	T	C	-0.0118	-0.00053	0.609564	0.600225	FALSE	FALSE	FALSE	I9_PULMEM	0.972193	0.01533	pulmonary	TRUE	reported	textfile	14	52621945	0.002034	6.69E-09	474236	eosinophi	TRUE	reported	igd	7.10E-05	33.66153	2	TRUE
rs9428321	C	T	C	T	0.015638	0.017291	0.666938	0.692126	FALSE	FALSE	FALSE	I9_PULMEM	0.290408	0.016355	pulmonary	TRUE	reported	textfile	1	1.21E+08	0.002185	8.56E-13	444895	eosinophi	TRUE	reported	igd	0.000115	51.22212	2	TRUE
rs9430574	A	G	A	G	-0.01969	-0.0025	0.325122	0.417591	FALSE	FALSE	FALSE	I9_PULMEM	0.869364	0.015229	pulmonary	TRUE	reported	textfile	1	9709072	0.002128	2.35E-20	474227	eosinophi	TRUE	reported	igd	0.000181	85.61431	2	TRUE
rs954954	C	A	C	A	-0.03522	0.015445	0.104889	0.120213	FALSE	FALSE	FALSE	I9_PULMEM	0.504286	0.02313	pulmonary	TRUE	reported	textfile	18	60902328	0.00326	3.57E-27	472836	eosinophi	TRUE	reported	igd	0.000247	116.719	2	TRUE
rs962993	T	C	T	C	-0.04043	0.045412	0.422478	0.302731	FALSE	FALSE	FALSE	I9_PULMEM	0.005144	0.016231	pulmonary	TRUE	reported	textfile	10	9053132	0.002009	4.92E-90	474236	eosinophi	TRUE	reported	igd	0.000853	405.0715	2	TRUE
rs964184	C	G	C	G	0.028842	-0.00652	0.866592	0.854981	FALSE	TRUE	FALSE	I9_PULMEM	0.759097	0.021266	pulmonary	TRUE	reported	textfile	11	1.17E+08	0.002913	4.39E-23	473776	eosinophi	TRUE	reported	igd	0.000207	98.03202	2	TRUE
rs9666598	G	C	G	C	-0.01971	-0.00972	0.842874	0.8213	FALSE	TRUE	FALSE	I9_PULMEM	0.621722	0.019692	pulmonary	TRUE	reported	textfile	11	325386	0.002778	1.36E-12	464642	eosinophi	TRUE	reported	igd	0.000108	50.31884	2	TRUE
rs9675999	A	G	A	G	0.016403	-0.00994	0.626697	0.643482	FALSE	FALSE	FALSE	I9_PULMEM	0.525597	0.015656	pulmonary	TRUE	reported	textfile	18	20627691	0.002057	1.59E-15	474218	eosinophi	TRUE	reported	igd	0.000134	63.58814	2	TRUE
rs9815874	T	C	T	C	0.028359	0.027949	0.299538	0.216362	FALSE	FALSE	FALSE	I9_PULMEM	0.125774	0.018256	pulmonary	TRUE	reported	textfile	3	1.88E+08	0.002157	1.93E-39	474234	eosinophi	TRUE	reported	igd	0.000364	172.8541	2	TRUE
rs9818987	T	C	T	C	0.013608	-0.00684	0.305904	0.41835	FALSE	FALSE	FALSE	I9_PULMEM	0.65399	0.015271	pulmonary	TRUE	reported	textfile	3	1.94E+08	0.002151	2.60E-10	473152	eosinophi	TRUE	reported	igd	8.46E-05	40.02266	2	TRUE
rs9835307	C	T	C	T	0.026306	0.001455	0.658298	0.664668	FALSE	FALSE	FALSE	I9_PULMEM	0.92713	0.015908	pulmonary	TRUE	reported	textfile	3	48857805	0.002187	2.69E-33	473153	eosinophi	TRUE	reported	igd	0.000306	144.6806	2	TRUE
rs9837045	A	G	A	G	0.011264	0.001004	0.455368	0.472646	FALSE	FALSE	FALSE	I9_PULMEM	0.946655	0.015004	pulmonary	TRUE	reported	textfile	3	3069773	0.001984	1.41E-08	471754	eosinophi	TRUE	reported	igd	6.83E-05	32.23295	2	TRUE
rs9840310	G	A	G	A	-0.01274	-0.02553	0.734621	0.740643	FALSE	FALSE	FALSE	I9_PULMEM	0.137743	0.017197	pulmonary	TRUE	reported	textfile	3	71452860	0.002239	1.30E-08	474236	eosinophi	TRUE	reported	igd	6.83E-05	32.38659	2	TRUE
rs9872485	G	T	G	T	-0.01835	-0.01771	0.164017	0.226262	FALSE	FALSE	FALSE	I9_PULMEM	0.326234	0.018043	pulmonary	TRUE	reported	textfile	3	16918405	0.00269	9.26E-12	473153	eosinophi	TRUE	reported	igd	9.84E-05	46.55379	2	TRUE
rs9894839	C	T	C	T	0.012116	0.018287	0.63524	0.598746	FALSE	FALSE	FALSE	I9_PULMEM	0.232149	0.015305	pulmonary	TRUE	reported	textfile	17	80491743	0.00213	1.33E-08	474211	eosinophi	TRUE	reported	igd	6.82E-05	32.3562	2	TRUE
rs9939774	T	C	T	C	-0.02924	-0.00477	0.404524	0.36895	FALSE	FALSE	FALSE	I9_PULMEM	0.758937	0.015533	pulmonary	TRUE	reported	textfile	16	30068354	0.00202	1.98E-47	474219	eosinophi	TRUE	reported	igd	0.000442	209.4746	2	TRUE
rs9965489	A	G	A	G	0.016264	0.00662	0.53313	0.496344	FALSE	FALSE	FALSE	I9_PULMEM	0.659148	0.015009	pulmonary	TRUE	reported	textfile	18	61356047	0.001991	3.30E-16	474232	eosinophi	TRUE	reported	igd	0.000141	66.72835	2	TRUE
rs9979383	T	C	T	C	0.028371	-0.0152	0.629829	0.652934	FALSE	FALSE	FALSE	I9_PULMEM	0.334103	0.015739	pulmonary	TRUE	reported	textfile	21	36715761	0.002067	8.00E-43	471753	eosinophi	TRUE	reported	igd	0.000399	188.3938	2	TRUE

Supplementary Table 4: Description of SNPs for lymphocyte cell counts incorporated after MR-presso deletion

SNP	effect_al	other_all	effect_al	other_all	beta.expo	beta.outc	eaf.expos	eaf.outco	remove	palindrom	ambiguous	id.outcom	pval.outc	se.outcom	outcome	mr_keep.c	pval_orig	data_sour	chr.expos	pos.expos	se.expos	pval.expos	samplesiz	exposure	mr_keep.e	pval_orig	data_sour	R2	F	action	mr_keep
rs1000329	T	C	T	C	-0.08239	0.006826	0.676463	0.656381	FALSE	FALSE	FALSE	I9_PULMEM	0.666285	0.015829	pulmonary	TRUE	reported	textfile	19	16449517	0.002111	1.00E-200	478964	lymphocyt	TRUE	reported	igd	0.00317	1523.137	2	TRUE
rs1002607	C	T	C	T	-0.01299	0.012635	0.592852	0.597624	FALSE	FALSE	FALSE	I9_PULMEM	0.409235	0.01531	pulmonary	TRUE	reported	textfile	9	1.14E+08	0.001934	1.93E-11	523523	lymphocyt	TRUE	reported	igd	8.61E-05	45.09237	2	TRUE
rs1002741	T	C	T	C	-0.01633	-0.01265	0.529463	0.624735	FALSE	FALSE	FALSE	I9_PULMEM	0.414831	0.01551	pulmonary	TRUE	reported	textfile	4	80898027	0.001366	6.33E-33	523843	lymphocyt	TRUE	reported	igd	0.000273	142.9647	2	TRUE
rs1007670	C	T	C	T	-0.02283	-0.02261	0.55724	0.396452	FALSE	FALSE	FALSE	I9_PULMEM	0.141391	0.015372	pulmonary	TRUE	reported	textfile	5	1.32E+08	0.001907	5.52E-33	523841	lymphocyt	TRUE	reported	igd	0.000273	143.283	2	TRUE
rs1010937	A	C	A	C	-0.0189	0.015332	0.238495	0.297322	FALSE	FALSE	FALSE	I9_PULMEM	0.351254	0.016447	pulmonary	TRUE	reported	textfile	8	72909349	0.002227	2.26E-17	524920	lymphocyt	TRUE	reported	igd	0.000137	72.03995	2	TRUE
rs1013875	T	C	T	C	-0.02978	0.018285	0.077325	0.115927	FALSE	FALSE	FALSE	I9_PULMEM	0.435687	0.023458	pulmonary	TRUE	reported	textfile	14	69179971	0.003547	4.89E-17	524917	lymphocyt	TRUE	reported	igd	0.000134	70.47072	2	TRUE
rs1014527	A	T	A	T	-0.01486	0.000641	0.624665	0.632314	FALSE	TRUE	FALSE	I9_PULMEM	0.96726	0.015606	pulmonary	TRUE	reported	textfile	14	99793449	0.001956	3.19E-14	524916	lymphocyt	TRUE	reported	igd	0.00011	57.72404	2	TRUE
rs1017405	A	T	A	T	0.013981	-0.01471	0.384429	0.39277	FALSE	TRUE	FALSE	I9_PULMEM	0.33958	0.015406	pulmonary	TRUE	reported	textfile	2	1.63E+08	0.001942	6.38E-13	523981	lymphocyt	TRUE	reported	igd	9.89E-05	51.82942	2	TRUE
rs1017875	T	C	T	C	-0.03609	0.013882	0.201673	0.214661	FALSE	FALSE	FALSE	I9_PULMEM	0.448605	0.01832	pulmonary	TRUE	reported	textfile	11	47999218	0.002353	5.01E-53	523837	lymphocyt	TRUE	reported	igd	0.000449	235.1841	2	TRUE
rs1017970	A	G	A	G	-0.0213	-0.00624	0.294374	0.310271	FALSE	FALSE	FALSE	I9_PULMEM	0.699916	0.016193	pulmonary	TRUE	reported	textfile	2	1.88E+08	0.002079	1.29E-24	522902	lymphocyt	TRUE	reported	igd	0.000201	104.9758	2	TRUE
rs1019132	A	C	A	C	-0.0142	-0.01924	0.175233	0.173142	FALSE	FALSE	FALSE	I9_PULMEM	0.332975	0.019872	pulmonary	TRUE	reported	textfile	2	71676999	0.0025	1.39E-08	524921	lymphocyt	TRUE	reported	igd	6.14E-05	32.25773	2	TRUE
rs1020696	T	C	T	C	-0.01062	-0.01158	0.418223	0.378415	FALSE	FALSE	FALSE	I9_PULMEM	0.454013	0.015472	pulmonary	TRUE	reported	textfile	2	85814984	0.001917	3.10E-08	523518	lymphocyt	TRUE	reported	igd	5.86E-05	30.69044	2	TRUE
rs1021219	T	A	T	A	-0.01182	0.008541	0.379385	0.397048	FALSE	TRUE	FALSE	I9_PULMEM	0.577547	0.015335	pulmonary	TRUE	reported	textfile	3	1.87E+08	0.001948	1.32E-09	524918	lymphocyt	TRUE	reported	igd	7.02E-05	36.83005	2	TRUE
rs1023050	A	T	A	T	0.021153	0.037107	0.1609	0.178429	FALSE	TRUE	FALSE	I9_PULMEM	0.058868	0.019642	pulmonary	TRUE	reported	textfile	7	92471518	0.002584	2.86E-16	524920	lymphocyt	TRUE	reported	igd	0.000128	67.01272	2	TRUE
rs1023752	A	G	A	G	-0.01918	0.007915	0.330226	0.368726	FALSE	FALSE	FALSE	I9_PULMEM	0.612871	0.015643	pulmonary	TRUE	reported	textfile	7	56036024	0.002008	1.35E-21	523832	lymphocyt	TRUE	reported	igd	0.000174	91.25543	2	TRUE
rs1024547	A	G	A	G	-0.01588	-0.00438	0.176635	0.217974	FALSE	FALSE	FALSE	I9_PULMEM	0.809999	0.018235	pulmonary	TRUE	reported	textfile	7	55147478	0.002483	1.65E-10	523513	lymphocyt	TRUE	reported	igd	7.82E-05	40.91758	2	TRUE
rs1043996	A	G	A	G	0.013372	0.007917	0.716638	0.759885	FALSE	FALSE	FALSE	I9_PULMEM	0.651561	0.01753	pulmonary	TRUE	reported	textfile	19	15295134	0.002139	4.21E-10	522444	lymphocyt	TRUE	reported	igd	7.48E-05	39.08135	2	TRUE
rs1044530	T	C	T	C	-0.02997	-0.00551	0.479095	0.528262	FALSE	FALSE	FALSE	I9_PULMEM	0.714102	0.015028	pulmonary	TRUE	reported	textfile	17	37938047	0.001892	1.91E-56	523521	lymphocyt	TRUE	reported	igd	0.000479	250.8832	2	TRUE
rs1046690	A	G	A	G	0.061958	-0.04723	0.190033	0.200491	FALSE	FALSE	FALSE	I9_PULMEM	0.012985	0.019012	pulmonary	TRUE	reported	textfile	12	6502832	0.00246	7.10E-140	524918	lymphocyt	TRUE	reported	igd	0.001207	634.3412	2	TRUE
rs1048000	G	A	G	A	-0.01334	0.010848	0.44679	0.422918	FALSE	FALSE	FALSE	I9_PULMEM	0.474515	0.015169	pulmonary	TRUE	reported	textfile	7	2761153	0.00193	4.97E-12	524905	lymphocyt	TRUE	reported	igd	9.10E-05	47.76009	2	TRUE
rs1049478	A	G	A	G	-0.0541	0.047897	0.051764	0.067459	FALSE	FALSE	FALSE	I9_PULMEM	0.109072	0.029891	pulmonary	TRUE	reported	textfile	1	1.99E+08	0.004268	8.95E-37	523521	lymphocyt	TRUE	reported	igd	0.000307	160.6437	2	TRUE
rs1050314	T	C	T	C	0.02071	0.000185	0.088907	0.070906	FALSE	FALSE	FALSE	I9_PULMEM	0.994943	0.029152	pulmonary	TRUE	reported	textfile	8	421777	0.003362	7.48E-10	520682	lymphocyt	TRUE	reported	igd	7.29E-05	37.94575	2	TRUE
rs1050979	G	A	G	A	0.024519	0.011517	0.525212	0.474812	FALSE	FALSE	FALSE	I9_PULMEM	0.442892	0.01501	pulmonary	TRUE	reported	textfile	6	410417	0.001898	3.97E-38	521152	lymphocyt	TRUE	reported	igd	0.00032	166.8827	2	TRUE
rs1074852	C	T	C	T	0.01522	0.005816	0.793501	0.721331	FALSE	FALSE	FALSE	I9_PULMEM	0.728625	0.016763	pulmonary	TRUE	reported	textfile	10	82273079	0.002339	7.88E-11	523979	lymphocyt	TRUE	reported	igd	8.08E-05	42.34159	2	TRUE
rs1076112	T	C	T	C	0.011391	0.008803	0.669401	0.728014	FALSE	FALSE	FALSE	I9_PULMEM	0.601136	0.016839	pulmonary	TRUE	reported	textfile	9	94486321	0.002015	1.62E-08	523517	lymphocyt	TRUE	reported	igd	6.10E-05	31.95744	2	TRUE
rs1076226	A	G	A	G	-0.0204	-0.00918	0.680729	0.746064	FALSE	FALSE	FALSE	I9_PULMEM	0.59422	0.017233	pulmonary	TRUE	reported	textfile	10	70976833	0.002032	1.06E-23	524922	lymphocyt	TRUE	reported	igd	0.000192	100.8182	2	TRUE
rs1077462	A	G	A	G	-0.08779	-0.02908	0.514001	0.597917	FALSE	FALSE	FALSE	I9_PULMEM	0.058338	0.015361	pulmonary	TRUE	reported	textfile	12	1.12E+08	0.001917	1.00E-200	523841	lymphocyt	TRUE	reported	igd	0.003987	2097.03	2	TRUE
rs1078334	G	A	G	A	-0.01192	-0.01721	0.639354	0.700878	FALSE	FALSE	FALSE	I9_PULMEM	0.292611	0.016353	pulmonary	TRUE	reported	textfile	12	50559128	0.001964	1.34E-09	524892	lymphocyt	TRUE	reported	igd	7.01E-05	36.80472	2	TRUE
rs1079417	T	G	T	G	-0.02125	-0.01464	0.416037	0.482429	FALSE	FALSE	FALSE	I9_PULMEM	0.329053	0.015002	pulmonary	TRUE	reported	textfile	10	1.26E+08	0.002001	2.50E-26	479584	lymphocyt	TRUE	reported	igd	0.000235	112.7986	2	TRUE
rs1081413	A	C	A	C	-0.01368	-0.01927	0.313929	0.286214	FALSE	FALSE	FALSE	I9_PULMEM	0.246401	0.016621	pulmonary	TRUE	reported	textfile	9	34723035	0.00205	2.55E-11	523521	lymphocyt	TRUE	reported	igd	8.51E-05	44.55057	2	TRUE
rs1082363	G	A	G	A	-0.01701	-0.01105	0.325475	0.428893	FALSE	FALSE	FALSE	I9_PULMEM	0.466924	0.015186	pulmonary	TRUE	reported	textfile	10	72625741	0.002013	3.11E-17	524920	lymphocyt	TRUE	reported	igd	0.000136	71.41188	2	TRUE
rs1082872	T	G	T	G	-0.03206	0.016362	0.366094	0.384907	FALSE	FALSE	FALSE	I9_PULMEM	0.289204	0.015437	pulmonary	TRUE	reported	textfile	10	25218243	0.001968	1.29E-59	524920	lymphocyt	TRUE	reported	igd	0.000505	265.4174	2	TRUE
rs1083863	G	A	G	A	-0.02611	0.026399	0.901514	0.921558	FALSE	FALSE	FALSE	I9_PULMEM	0.344309	0.027915	pulmonary	TRUE	reported	textfile	11	46929954	0.003192	3.00E-16	523844	lymphocyt	TRUE	reported	igd	0.000128	66.8939	2	TRUE
rs1084657	C	T	C	T	-0.01411	0.025665	0.442146	0.459195	FALSE	FALSE	FALSE	I9_PULMEM	0.090278	0.015151	pulmonary	TRUE	reported	textfile	12	1.24E+08	0.001924	2.37E-13	524920	lymphocyt	TRUE	reported	igd	0.000102	53.75979	2	TRUE
rs1086533	G	A	G	A	0.013177	-0.00942	0.625008	0.651646	FALSE	FALSE	FALSE	I9_PULMEM	0.550846	0.0158	pulmonary	TRUE	reported	textfile	2	62551472	0.002022	7.38E-11	480043	lymphocyt	TRUE	reported	igd	8.85E-05	42.4687	2	TRUE
rs1087537	T	C	T	C	-0.02083	-0.0072	0.417813	0.344801	FALSE	FALSE	FALSE	I9_PULMEM	0.649551	0.015852	pulmonary	TRUE	reported	textfile	1	1.02E+08	0.001927	3.37E-27	524906	lymphocyt	TRUE	reported	igd	0.000222	116.7898	2	TRUE
rs1093201	A	G	A	G	-0.0146	-0.02477	0.729687	0.789343	FALSE	FALSE	FALSE	I9_PULMEM	0.178302	0.0184	pulmonary	TRUE	reported	textfile	2	2.05E+08	0.002118	5.65E-12	523983	lymphocyt	TRUE	reported	igd	9.07E-05	47.51734	2	TRUE
rs1095640	A	G	A	G	-0.02534	0.024458	0.345058	0.338168	FALSE	FALSE	FALSE	I9_PULMEM	0.122439	0.015835	pulmonary	TRUE	reported	textfile	8	1.29E+08	0.001992	5.05E-37	523982	lymphocyt	TRUE	reported	igd	0.000309	161.7947	2	TRUE
rs1096512	G	T	G	T	-0.0234	0.006482	0.094818	0.09946	FALSE	FALSE	FALSE	I9_PULMEM	0.795375	0.024996	pulmonary	TRUE	reported	textfile	9	21776615	0.00336	3.42E-12	481443	lymphocyt	TRUE	reported	igd	0.000101	48.50522	2	TRUE
rs1098591	G	A	G	A	-0.0226	-0.0032	0.110633	0.085063	FALSE	FALSE	FALSE	I9_PULMEM	0.905421	0.026954	pulmonary	TRUE	reported	textfile	9	1.26E+08	0.003015	6.90E-14	524920	lymphocyt	TRUE	reported	igd	0.000107	56.1727	2	TRUE
rs1098633	A	G	A	G	-0.01161	-0.00257	0.649791	0.684027	FALSE	FALSE	FALSE	I9_PULMEM	0.873144	0.016105	pulmonary	TRUE	reported	textfile	9	1.27E+08	0.002008	7.60E-09	523840	lymphocyt	TRUE	reported	igd	6.38E-05	33.42416	2	TRUE

rs1103338	T	A	T	A	0.019588	-0.01483	0.418839	0.368349	FALSE	TRUE	FALSE	I9_PULMEN	0.340197	0.015554	pulmonary	TRUE	reported	textfile	11	36074218	0.00192	2.07E-24	524922	lymphocyt	TRUE	reported	igd	0.000198	104.0821	2	TRUE
rs1105287	G	A	G	A	0.068864	0.01284	0.371691	0.410108	FALSE	FALSE	FALSE	I9_PULMEN	0.400585	0.015276	pulmonary	TRUE	reported	textfile	12	9905690	0.001958	1.00E-200	523521	lymphocyt	TRUE	reported	igd	0.002357	1236.965	2	TRUE
rs1107390	A	G	A	G	-0.0127	0.025591	0.567498	0.534178	FALSE	FALSE	FALSE	I9_PULMEN	0.089726	0.015082	pulmonary	TRUE	reported	textfile	15	90653194	0.001925	4.41E-11	523839	lymphocyt	TRUE	reported	igd	8.30E-05	43.49814	2	TRUE
rs1108409	A	G	A	G	-0.01838	0.007511	0.296205	0.266952	FALSE	FALSE	FALSE	I9_PULMEN	0.658951	0.017018	pulmonary	TRUE	reported	textfile	19	52128795	0.002067	6.42E-19	523842	lymphocyt	TRUE	reported	igd	0.000151	79.03498	2	TRUE
rs1113447	G	A	G	A	-0.01422	0.031324	0.635835	0.668241	FALSE	FALSE	FALSE	I9_PULMEN	0.050683	0.016029	pulmonary	TRUE	reported	textfile	5	1.56E+08	0.001962	4.42E-13	524919	lymphocyt	TRUE	reported	igd	0.0001	52.4995	2	TRUE
rs1116070	A	G	A	G	0.030499	-0.02372	0.22222	0.261331	FALSE	FALSE	FALSE	I9_PULMEN	0.164564	0.017069	pulmonary	TRUE	reported	textfile	14	1.03E+08	0.002291	2.12E-40	524921	lymphocyt	TRUE	reported	igd	0.000338	177.2227	2	TRUE
rs1116264	A	G	A	G	0.027778	-0.002	0.096614	0.135862	FALSE	FALSE	FALSE	I9_PULMEN	0.927492	0.021994	pulmonary	TRUE	reported	textfile	18	77250669	0.003337	8.97E-17	480500	lymphocyt	TRUE	reported	igd	0.000144	69.29274	2	TRUE
rs1116824	C	T	C	T	-0.01949	-0.00433	0.461347	0.508996	FALSE	FALSE	FALSE	I9_PULMEN	0.773906	0.01506	pulmonary	TRUE	reported	textfile	12	48208368	0.00193	6.04E-24	523519	lymphocyt	TRUE	reported	igd	0.000195	101.9468	2	TRUE
rs1118866	T	C	T	C	0.014779	0.041592	0.703532	0.614706	FALSE	FALSE	FALSE	I9_PULMEN	0.007097	0.015449	pulmonary	TRUE	reported	textfile	12	68520751	0.002075	1.10E-12	523522	lymphocyt	TRUE	reported	igd	9.69E-05	50.72853	2	TRUE
rs1121872	G	A	G	A	0.033008	-0.00652	0.376523	0.376194	FALSE	FALSE	FALSE	I9_PULMEN	0.674025	0.015503	pulmonary	TRUE	reported	textfile	11	1.23E+08	0.001958	1.03E-63	523984	lymphocyt	TRUE	reported	igd	0.000542	284.1917	2	TRUE
rs1122430	T	C	T	C	-0.0383	-0.07896	0.098823	0.080292	FALSE	FALSE	FALSE	I9_PULMEN	0.00487	0.028045	pulmonary	TRUE	reported	textfile	11	1E+08	0.003174	1.73E-33	523517	lymphocyt	TRUE	reported	igd	0.000278	145.6069	2	TRUE
rs1123180	T	C	T	C	0.07386	0.028201	0.007228	0.016011	FALSE	FALSE	FALSE	I9_PULMEN	0.639476	0.060203	pulmonary	TRUE	reported	textfile	11	64291461	0.012425	2.85E-09	473735	lymphocyt	TRUE	reported	igd	7.46E-05	35.33654	2	TRUE
rs1124614	T	C	T	C	0.011393	0.00545	0.54935	0.510899	FALSE	FALSE	FALSE	I9_PULMEN	0.71736	0.015055	pulmonary	TRUE	reported	textfile	11	396701	0.001984	9.52E-09	478607	lymphocyt	TRUE	reported	igd	6.89E-05	32.97547	2	TRUE
rs1124771	T	C	T	C	-0.0199	0.026927	0.368908	0.380972	FALSE	FALSE	FALSE	I9_PULMEN	0.081442	0.015454	pulmonary	TRUE	reported	textfile	1	28229681	0.00196	3.38E-24	524919	lymphocyt	TRUE	reported	igd	0.000196	103.105	2	TRUE
rs1129926	A	G	A	G	-0.02671	-0.0244	0.045938	0.030172	FALSE	FALSE	FALSE	I9_PULMEN	0.578835	0.043951	pulmonary	TRUE	reported	textfile	14	50412380	0.004632	8.35E-09	523976	lymphocyt	TRUE	reported	igd	6.34E-05	33.24386	2	TRUE
rs1131162	C	T	C	T	0.041426	0.04676	0.019939	0.010524	FALSE	FALSE	FALSE	I9_PULMEN	0.522041	0.073039	pulmonary	TRUE	reported	textfile	1	1.99E+08	0.006895	1.93E-09	523984	lymphocyt	TRUE	reported	igd	6.89E-05	36.09739	2	TRUE
rs1131426	C	T	C	T	-0.01709	-0.02094	0.247465	0.216978	FALSE	FALSE	FALSE	I9_PULMEN	0.250136	0.018206	pulmonary	TRUE	reported	textfile	8	30242076	0.00222	1.46E-14	523965	lymphocyt	TRUE	reported	igd	0.000113	59.24123	2	TRUE
rs1132352	C	A	C	A	-0.02398	0.012827	0.538949	0.511998	FALSE	FALSE	FALSE	I9_PULMEN	0.392742	0.015009	pulmonary	TRUE	reported	textfile	6	24806070	0.001895	1.15E-36	522445	lymphocyt	TRUE	reported	igd	0.000306	160.092	2	TRUE
rs1135139	C	T	C	T	-0.03775	0.043027	0.097132	0.113124	FALSE	FALSE	FALSE	I9_PULMEN	0.070189	0.023763	pulmonary	TRUE	reported	textfile	17	2190390	0.003208	6.37E-32	524904	lymphocyt	TRUE	reported	igd	0.000264	138.4652	2	TRUE
rs1135423	A	G	A	G	-0.06753	0.039933	0.074926	0.034216	FALSE	FALSE	FALSE	I9_PULMEN	0.334412	0.04137	pulmonary	TRUE	reported	textfile	2	43464818	0.003634	4.90E-77	523045	lymphocyt	TRUE	reported	igd	0.00066	345.2998	2	TRUE
rs1150501	G	T	G	T	-0.01338	-0.01011	0.410932	0.447441	FALSE	FALSE	FALSE	I9_PULMEN	0.503808	0.015117	pulmonary	TRUE	reported	textfile	3	1.88E+08	0.001918	3.18E-12	523515	lymphocyt	TRUE	reported	igd	9.29E-05	48.63553	2	TRUE
rs1156770	T	G	T	G	0.054358	-0.02694	0.261577	0.295435	FALSE	FALSE	FALSE	I9_PULMEN	0.10071	0.016412	pulmonary	TRUE	reported	textfile	5	35859863	0.002147	2.40E-141	524921	lymphocyt	TRUE	reported	igd	0.00122	641.0047	2	TRUE
rs1165276	G	T	G	T	-0.02096	0.03534	0.100157	0.078781	FALSE	FALSE	FALSE	I9_PULMEN	0.211677	0.028295	pulmonary	TRUE	reported	textfile	17	16786819	0.003152	3.01E-11	524921	lymphocyt	TRUE	reported	igd	8.43E-05	44.23161	2	TRUE
rs1167062	T	C	T	C	0.010673	-0.01976	0.48557	0.484351	FALSE	FALSE	FALSE	I9_PULMEN	0.189431	0.015057	pulmonary	TRUE	reported	textfile	19	12189379	0.001927	3.11E-08	523980	lymphocyt	TRUE	reported	igd	5.85E-05	30.67665	2	TRUE
rs1167629	G	C	G	C	0.026968	0.015304	0.19074	0.199221	FALSE	TRUE	FALSE	I9_PULMEN	0.414356	0.01875	pulmonary	TRUE	reported	textfile	2	2.27E+08	0.0024	2.86E-29	523980	lymphocyt	TRUE	reported	igd	0.000241	126.2622	2	TRUE
rs1168446	T	A	T	A	-0.04727	0.005903	0.327486	0.254799	FALSE	TRUE	FALSE	I9_PULMEN	0.732243	0.017253	pulmonary	TRUE	reported	textfile	2	1.82E+08	0.002015	1.30E-121	522903	lymphocyt	TRUE	reported	igd	0.001051	550.3253	2	TRUE
rs1168760	T	C	T	C	0.020824	-0.04064	0.09019	0.052321	FALSE	FALSE	FALSE	I9_PULMEN	0.227458	0.03367	pulmonary	TRUE	reported	textfile	17	76393762	0.003402	9.57E-10	524912	lymphocyt	TRUE	reported	igd	7.14E-05	37.46779	2	TRUE
rs1168828	C	T	C	T	-0.01484	0.006976	0.18925	0.156981	FALSE	FALSE	FALSE	I9_PULMEN	0.735761	0.020672	pulmonary	TRUE	reported	textfile	2	40698811	0.002405	6.98E-10	524921	lymphocyt	TRUE	reported	igd	7.26E-05	38.09005	2	TRUE
rs1168830	T	C	T	C	0.01644	-0.02066	0.119835	0.100143	FALSE	FALSE	FALSE	I9_PULMEN	0.410838	0.025123	pulmonary	TRUE	reported	textfile	2	1.28E+08	0.002956	2.74E-08	523982	lymphocyt	TRUE	reported	igd	5.90E-05	30.93094	2	TRUE
rs1170638	T	G	T	G	0.026978	0.014297	0.235888	0.22266	FALSE	FALSE	FALSE	I9_PULMEN	0.428803	0.018069	pulmonary	TRUE	reported	textfile	3	39296881	0.002223	7.44E-34	524920	lymphocyt	TRUE	reported	igd	0.00028	147.2784	2	TRUE
rs1171233	C	T	C	T	0.015222	0.02316	0.269338	0.233927	FALSE	FALSE	FALSE	I9_PULMEN	0.197799	0.017983	pulmonary	TRUE	reported	textfile	3	1.28E+08	0.002143	1.26E-12	523974	lymphocyt	TRUE	reported	igd	9.63E-05	50.45421	2	TRUE
rs1174287	T	C	T	C	-0.01661	0.006132	0.837406	0.840238	FALSE	FALSE	FALSE	I9_PULMEN	0.766563	0.020655	pulmonary	TRUE	reported	textfile	5	1.79E+08	0.002601	1.75E-10	524923	lymphocyt	TRUE	reported	igd	7.77E-05	40.78091	2	TRUE
rs1175561	A	G	A	G	0.044471	-0.04855	0.05762	0.046682	FALSE	FALSE	FALSE	I9_PULMEN	0.171645	0.035518	pulmonary	TRUE	reported	textfile	16	67680806	0.004114	3.31E-27	519069	lymphocyt	TRUE	reported	igd	0.000225	116.8486	2	TRUE
rs1176119	G	A	G	A	-0.01076	-0.01994	0.45427	0.493467	FALSE	FALSE	FALSE	I9_PULMEN	0.184426	0.015022	pulmonary	TRUE	reported	textfile	7	1.29E+08	0.001911	1.84E-08	523522	lymphocyt	TRUE	reported	igd	6.06E-05	31.70898	2	TRUE
rs1177268	G	A	G	A	-0.01363	-0.01291	0.566052	0.540231	FALSE	FALSE	FALSE	I9_PULMEN	0.391115	0.015058	pulmonary	TRUE	reported	textfile	2	61286593	0.001975	5.45E-12	481436	lymphocyt	TRUE	reported	igd	9.88E-05	47.59234	2	TRUE
rs1179341	A	G	A	G	-0.04456	-0.03745	0.040534	0.049255	FALSE	FALSE	FALSE	I9_PULMEN	0.280128	0.034671	pulmonary	TRUE	reported	textfile	8	79069520	0.004906	1.12E-19	524921	lymphocyt	TRUE	reported	igd	0.000157	82.51095	2	TRUE
rs1180005	T	C	T	C	-0.02561	0.001445	0.509954	0.501476	FALSE	FALSE	FALSE	I9_PULMEN	0.923221	0.014997	pulmonary	TRUE	reported	textfile	1	1.51E+08	0.001882	3.88E-42	524922	lymphocyt	TRUE	reported	igd	0.000353	185.2166	2	TRUE
rs1202180	A	G	A	G	-0.02634	-0.01402	0.109842	0.133265	FALSE	FALSE	FALSE	I9_PULMEN	0.527668	0.022206	pulmonary	TRUE	reported	textfile	1	2.26E+08	0.003018	2.69E-18	524822	lymphocyt	TRUE	reported	igd	0.000145	76.19444	2	TRUE
rs1203801	C	G	C	G	-0.01649	0.004713	0.268002	0.250632	FALSE	TRUE	FALSE	I9_PULMEN	0.786249	0.01738	pulmonary	TRUE	reported	textfile	1	1.62E+08	0.002129	1.01E-14	524919	lymphocyt	TRUE	reported	igd	0.000114	59.96948	2	TRUE
rs1204589	T	C	T	C	0.026783	-0.00576	0.248069	0.294091	FALSE	FALSE	FALSE	I9_PULMEN	0.726721	0.016472	pulmonary	TRUE	reported	textfile	1	1.59E+08	0.002177	9.70E-35	524922	lymphocyt	TRUE	reported	igd	0.000288	151.3561	2	TRUE
rs1205956	T	C	T	C	0.016276	0.022916	0.150315	0.150317	FALSE	FALSE	FALSE	I9_PULMEN	0.275813	0.021028	pulmonary	TRUE	reported	textfile	1	1.59E+08	0.002646	7.96E-10	524915	lymphocyt	TRUE	reported	igd	7.21E-05	37.83677	2	TRUE
rs1208888	T	C	T	C	0.034026	-0.00304	0.408499	0.444624	FALSE	FALSE	FALSE	I9_PULMEN	0.840689	0.015114	pulmonary	TRUE	reported	textfile	1	1.01E+08	0.001914	1.30E-70	523979	lymphocyt	TRUE	reported	igd	0.000603	316.0358	2	TRUE
rs1212728	T	C	T	C	-0.01675	0.010418	0.710139	0.650482	FALSE	FALSE	FALSE	I9_PULMEN	0.508824	0.015769	pulmonary	TRUE	reported	textfile	1	65431599	0.002089	1.11E-15	524920	lymphocyt	TRUE	reported	igd	0.000122	64.30649	2	TRUE
rs1213391	A	C	A	C	0.015624	-0.01069	0.435692	0.376015	FALSE	FALSE	FALSE	I9_PULMEN	0.488462	0.015428	pulmonary	TRUE	reported	textfile	1	1.01E+08	0.001901	2.15E-16	524916	lymphocyt	TRUE	reported	igd	0.000129	67.54895	2	TRUE

rs1214203	A	G	A	G	-0.01298	0.017619	0.332829	0.347534	FALSE	FALSE	FALSE	I9_PULMEM	0.264402	0.015787	pulmonary	TRUE	reported	textfile	1	39542129	0.002031	1.73E-10	523517	lymphocyt	TRUE	reported	igd	7.80E-05	40.81879	2	TRUE
rs1219013	A	G	A	G	0.01184	0.019475	0.526092	0.521036	FALSE	FALSE	FALSE	I9_PULMEM	0.195334	0.015039	pulmonary	TRUE	reported	textfile	6	44767072	0.001892	3.99E-10	523516	lymphocyt	TRUE	reported	igd	7.48E-05	39.16151	2	TRUE
rs1233049	G	T	G	T	0.019277	0.009821	0.36489	0.353726	FALSE	FALSE	FALSE	I9_PULMEM	0.530804	0.015669	pulmonary	TRUE	reported	textfile	3	27791614	0.001744	2.36E-28	524922	lymphocyt	TRUE	reported	igd	0.000233	122.1755	2	TRUE
rs1246325	A	G	A	G	0.014581	-0.01414	0.274793	0.19884	FALSE	FALSE	FALSE	I9_PULMEM	0.453225	0.018845	pulmonary	TRUE	reported	textfile	19	297217	0.00221	4.32E-11	480340	lymphocyt	TRUE	reported	igd	9.06E-05	43.52996	2	TRUE
rs1252669	A	G	A	G	0.037387	-0.02844	0.160592	0.128926	FALSE	FALSE	FALSE	I9_PULMEM	0.203782	0.022381	pulmonary	TRUE	reported	textfile	6	1.08E+08	0.002688	6.06E-44	480503	lymphocyt	TRUE	reported	igd	0.000402	193.4554	2	TRUE
rs1254290	G	C	G	C	-0.01115	-0.00111	0.39893	0.329651	FALSE	TRUE	FALSE	I9_PULMEM	0.944685	0.015937	pulmonary	TRUE	reported	textfile	8	68813188	0.00193	7.87E-09	524920	lymphocyt	TRUE	reported	igd	6.35E-05	33.35798	2	TRUE
rs1256717	C	T	C	T	0.013418	-0.02661	0.235351	0.273846	FALSE	FALSE	FALSE	I9_PULMEM	0.114045	0.016837	pulmonary	TRUE	reported	textfile	1	62103005	0.002229	1.78E-09	524922	lymphocyt	TRUE	reported	igd	6.90E-05	36.23713	2	TRUE
rs1258600	G	T	G	T	-0.01372	-0.004	0.352779	0.391402	FALSE	FALSE	FALSE	I9_PULMEM	0.795396	0.015416	pulmonary	TRUE	reported	textfile	13	1E+08	0.001984	4.87E-12	524919	lymphocyt	TRUE	reported	igd	9.11E-05	47.81453	2	TRUE
rs1259897	G	T	G	T	0.055094	-0.03173	0.519622	0.565009	FALSE	FALSE	FALSE	I9_PULMEM	0.037554	0.015255	pulmonary	TRUE	reported	textfile	16	30482540	0.001917	1.58E-181	523983	lymphocyt	TRUE	reported	igd	0.001574	825.9671	2	TRUE
rs1260326	C	T	C	T	-0.02397	0.007219	0.602112	0.650688	FALSE	FALSE	FALSE	I9_PULMEM	0.647251	0.015776	pulmonary	TRUE	reported	textfile	2	27730940	0.001925	1.44E-35	523520	lymphocyt	TRUE	reported	igd	0.000296	155.0504	2	TRUE
rs1262748	A	G	A	G	0.017139	0.011783	0.106785	0.168598	FALSE	FALSE	FALSE	I9_PULMEM	0.55985	0.020208	pulmonary	TRUE	reported	textfile	21	37766505	0.003116	3.88E-08	524918	lymphocyt	TRUE	reported	igd	5.76E-05	30.25342	2	TRUE
rs1263692	C	T	C	T	-0.01363	0.008799	0.729623	0.740103	FALSE	FALSE	FALSE	I9_PULMEM	0.607476	0.01713	pulmonary	TRUE	reported	textfile	3	1.42E+08	0.002139	1.92E-10	523983	lymphocyt	TRUE	reported	igd	7.75E-05	40.61589	2	TRUE
rs1264018	T	C	T	C	0.016369	0.001278	0.175849	0.187879	FALSE	FALSE	FALSE	I9_PULMEM	0.947149	0.019282	pulmonary	TRUE	reported	textfile	4	6893181	0.002605	3.40E-10	481439	lymphocyt	TRUE	reported	igd	8.20E-05	39.48454	2	TRUE
rs1264593	T	G	T	G	0.0146	0.025117	0.494948	0.493252	FALSE	FALSE	FALSE	I9_PULMEM	0.09428	0.015011	pulmonary	TRUE	reported	textfile	8	1.02E+08	0.001909	2.15E-14	522903	lymphocyt	TRUE	reported	igd	0.000112	58.49142	2	TRUE
rs1269273	T	G	T	G	-0.01247	0.028322	0.361134	0.339919	FALSE	FALSE	FALSE	I9_PULMEM	0.073546	0.015827	pulmonary	TRUE	reported	textfile	2	1.66E+08	0.001963	2.17E-10	523981	lymphocyt	TRUE	reported	igd	7.70E-05	40.36732	2	TRUE
rs1274541	T	G	T	G	0.016574	0.016423	0.60014	0.507565	FALSE	FALSE	FALSE	I9_PULMEM	0.276309	0.015086	pulmonary	TRUE	reported	textfile	1	2.14E+08	0.00192	6.32E-18	524921	lymphocyt	TRUE	reported	igd	0.000142	74.51618	2	TRUE
rs1281183	A	G	A	G	-0.01621	-0.0237	0.353889	0.325529	FALSE	FALSE	FALSE	I9_PULMEM	0.140865	0.016092	pulmonary	TRUE	reported	textfile	12	48376213	0.002002	5.99E-16	523517	lymphocyt	TRUE	reported	igd	0.000125	65.55959	2	TRUE
rs1291414	T	C	T	C	0.0123	-0.01647	0.297616	0.245784	FALSE	FALSE	FALSE	I9_PULMEM	0.35114	0.017659	pulmonary	TRUE	reported	textfile	6	23443234	0.002075	3.18E-09	524919	lymphocyt	TRUE	reported	igd	6.69E-05	35.13762	2	TRUE
rs1292069	C	T	C	T	-0.0225	0.009588	0.449582	0.462536	FALSE	FALSE	FALSE	I9_PULMEM	0.524009	0.015047	pulmonary	TRUE	reported	textfile	17	57928290	0.001903	3.09E-32	524909	lymphocyt	TRUE	reported	igd	0.000266	139.8428	2	TRUE
rs1297265	G	A	G	A	0.011767	0.011608	0.443092	0.399483	FALSE	FALSE	FALSE	I9_PULMEM	0.447924	0.015296	pulmonary	TRUE	reported	textfile	21	16817051	0.00191	7.44E-10	523522	lymphocyt	TRUE	reported	igd	7.25E-05	37.95449	2	TRUE
rs1304098	C	T	C	T	0.018955	0.018575	0.291865	0.307964	FALSE	FALSE	FALSE	I9_PULMEM	0.253121	0.016254	pulmonary	TRUE	reported	textfile	20	8616195	0.002084	9.96E-20	523981	lymphocyt	TRUE	reported	igd	0.000158	82.72761	2	TRUE
rs1310598	A	G	A	G	0.045686	-0.03477	0.274165	0.337344	FALSE	FALSE	FALSE	I9_PULMEM	0.028307	0.015856	pulmonary	TRUE	reported	textfile	4	38602467	0.002118	3.64E-103	524917	lymphocyt	TRUE	reported	igd	0.000886	465.2782	2	TRUE
rs1313364	C	T	C	T	0.066249	-0.00849	0.680761	0.546145	FALSE	FALSE	FALSE	I9_PULMEM	0.575429	0.015158	pulmonary	TRUE	reported	textfile	4	38366090	0.002022	1.00E-200	523520	lymphocyt	TRUE	reported	igd	0.002046	1073.482	2	TRUE
rs1317575	C	G	C	G	0.013696	-0.00855	0.696319	0.723747	FALSE	TRUE	FALSE	I9_PULMEM	0.610184	0.016766	pulmonary	TRUE	reported	textfile	16	78572255	0.002056	2.80E-11	524918	lymphocyt	TRUE	reported	igd	8.45E-05	44.37513	2	TRUE
rs1319951	A	G	A	G	-0.01409	0.001147	0.298486	0.311407	FALSE	FALSE	FALSE	I9_PULMEM	0.943609	0.016209	pulmonary	TRUE	reported	textfile	6	1.1E+08	0.002146	5.37E-11	481440	lymphocyt	TRUE	reported	igd	8.95E-05	43.08993	2	TRUE
rs1320562	C	G	C	G	0.012709	-0.00371	0.758464	0.721467	FALSE	TRUE	FALSE	I9_PULMEM	0.824619	0.01676	pulmonary	TRUE	reported	textfile	1	90174762	0.002208	8.86E-09	524920	lymphocyt	TRUE	reported	igd	6.31E-05	33.13012	2	TRUE
rs1326207	G	C	G	C	-0.01189	-0.00428	0.687591	0.70013	FALSE	TRUE	FALSE	I9_PULMEM	0.793986	0.016377	pulmonary	TRUE	reported	textfile	8	56760360	0.002052	7.10E-09	524880	lymphocyt	TRUE	reported	igd	6.39E-05	33.55739	2	TRUE
rs1344603	C	T	C	T	0.029335	-0.00941	0.67571	0.670109	FALSE	FALSE	FALSE	I9_PULMEM	0.556934	0.016017	pulmonary	TRUE	reported	textfile	4	38109847	0.002022	1.19E-47	523521	lymphocyt	TRUE	reported	igd	0.000402	210.4787	2	TRUE
rs1371076	C	T	C	T	0.014929	0.002736	0.740378	0.733143	FALSE	FALSE	FALSE	I9_PULMEM	0.872087	0.016992	pulmonary	TRUE	reported	textfile	12	90561450	0.002153	4.22E-12	524923	lymphocyt	TRUE	reported	igd	9.16E-05	48.0808	2	TRUE
rs1384804	A	C	A	C	-0.05025	-0.01059	0.748054	0.733706	FALSE	FALSE	FALSE	I9_PULMEM	0.532395	0.016965	pulmonary	TRUE	reported	textfile	8	79572222	0.002188	1.08E-116	523840	lymphocyt	TRUE	reported	igd	0.001006	527.4224	2	TRUE
rs1399180	C	T	C	T	0.025838	-0.01631	0.828961	0.833363	FALSE	FALSE	FALSE	I9_PULMEM	0.417743	0.020124	pulmonary	TRUE	reported	textfile	10	8098719	0.002529	1.82E-24	522904	lymphocyt	TRUE	reported	igd	0.0002	104.3803	2	TRUE
rs1399746	C	T	C	T	0.054097	-0.20046	0.026033	0.005102	FALSE	FALSE	FALSE	I9_PULMEM	0.056476	0.105101	pulmonary	TRUE	reported	textfile	15	44027885	0.005994	1.89E-19	524922	lymphocyt	TRUE	reported	igd	0.000155	81.45378	2	TRUE
rs1407634	T	C	T	C	-0.0489	-0.04698	0.01433	0.003269	FALSE	FALSE	FALSE	I9_PULMEM	0.719633	0.130892	pulmonary	TRUE	reported	textfile	3	1.13E+08	0.007981	9.26E-10	524523	lymphocyt	TRUE	reported	igd	7.16E-05	37.53601	2	TRUE
rs1418010	T	C	T	C	-0.06143	0.078152	0.009564	0.01243	FALSE	FALSE	FALSE	I9_PULMEM	0.253087	0.068381	pulmonary	TRUE	reported	textfile	19	10374952	0.010349	3.01E-09	478898	lymphocyt	TRUE	reported	igd	7.36E-05	35.2306	2	TRUE
rs1431545	T	C	T	C	0.021141	0.007412	0.653307	0.696286	FALSE	FALSE	FALSE	I9_PULMEM	0.65016	0.016343	pulmonary	TRUE	reported	textfile	4	90220659	0.002045	5.05E-25	523843	lymphocyt	TRUE	reported	igd	0.000204	106.8717	2	TRUE
rs1438751	A	G	A	G	-0.02988	-0.01203	0.0442	0.038619	FALSE	FALSE	FALSE	I9_PULMEM	0.756868	0.038854	pulmonary	TRUE	reported	textfile	16	67606308	0.004626	1.08E-10	524921	lymphocyt	TRUE	reported	igd	7.95E-05	41.72876	2	TRUE
rs1445207	A	C	A	C	0.066726	-0.25172	0.007638	0.001376	FALSE	FALSE	FALSE	I9_PULMEM	0.214262	0.202689	pulmonary	TRUE	reported	textfile	1	1.02E+08	0.011459	5.93E-09	521981	lymphocyt	TRUE	reported	igd	6.50E-05	33.90745	2	TRUE
rs1448318	A	G	A	G	0.017849	0.008872	0.614888	0.551218	FALSE	FALSE	FALSE	I9_PULMEM	0.559044	0.015185	pulmonary	TRUE	reported	textfile	2	2.24E+08	0.001949	5.49E-20	523979	lymphocyt	TRUE	reported	igd	0.00016	83.86919	2	TRUE
rs1456059	G	A	G	A	0.01286	-0.008	0.766259	0.717908	FALSE	FALSE	FALSE	I9_PULMEM	0.631758	0.016686	pulmonary	TRUE	reported	textfile	18	48954037	0.002236	9.06E-09	524921	lymphocyt	TRUE	reported	igd	6.30E-05	33.07781	2	TRUE
rs1457194	G	A	G	A	-0.03089	0.031335	0.058785	0.039156	FALSE	FALSE	FALSE	I9_PULMEM	0.417228	0.038627	pulmonary	TRUE	reported	textfile	16	24044921	0.004077	3.71E-14	524918	lymphocyt	TRUE	reported	igd	0.000109	57.39425	2	TRUE
rs1459478	C	A	C	A	-0.03541	-0.0636	0.026364	0.030241	FALSE	FALSE	FALSE	I9_PULMEM	0.145804	0.043724	pulmonary	TRUE	reported	textfile	17	41809207	0.006051	4.97E-09	524866	lymphocyt	TRUE	reported	igd	6.52E-05	34.24877	2	TRUE
rs1477008	A	G	A	G	0.025886	-0.0665	0.043802	0.022199	FALSE	FALSE	FALSE	I9_PULMEM	0.18866	0.05059	pulmonary	TRUE	reported	textfile	12	32635390	0.004635	2.39E-08	523984	lymphocyt	TRUE	reported	igd	5.95E-05	31.19096	2	TRUE
rs1486015	G	A	G	A	-0.04879	0.094185	0.019608	0.01664	FALSE	FALSE	FALSE	I9_PULMEM	0.112926	0.059416	pulmonary	TRUE	reported	textfile	2	1.44E+08	0.00702	3.79E-12	523981	lymphocyt	TRUE	reported	igd	9.22E-05	48.30628	2	TRUE
rs1491664	A	G	A	G	-0.01535	-0.00165	0.200884	0.192296	FALSE	FALSE	FALSE	I9_PULMEM	0.931082	0.019079	pulmonary	TRUE	reported	textfile	3	1.71E+08	0.002395	1.54E-10	522443	lymphocyt	TRUE	reported	igd	7.86E-05	41.0561	2	TRUE

rs1491765	A	G	A	G	-0.01727	-0.00995	0.293219	0.349658	FALSE	FALSE	FALSE	I9_PULMEN	0.527106	0.015735	pulmonary	TRUE	reported	textfile	17	73028923	0.002093	1.67E-16	523979	lymphocyt	TRUE	reported	igd	0.00013	68.0445	2	TRUE
rs1493694	T	C	T	C	0.020046	0.015643	0.10785	0.136616	FALSE	FALSE	FALSE	I9_PULMEN	0.473844	0.021841	pulmonary	TRUE	reported	textfile	1	1.21E+08	0.003045	4.78E-11	523521	lymphocyt	TRUE	reported	igd	8.28E-05	43.33903	2	TRUE
rs1494539	T	C	T	C	0.048621	-0.02662	0.023606	0.019997	FALSE	FALSE	FALSE	I9_PULMEN	0.620337	0.053732	pulmonary	TRUE	reported	textfile	15	69583434	0.006697	4.04E-13	503472	lymphocyt	TRUE	reported	igd	0.000105	52.70908	2	TRUE
rs1499845	T	C	T	C	0.047879	0.044482	0.018609	0.015726	FALSE	FALSE	FALSE	I9_PULMEN	0.464831	0.060858	pulmonary	TRUE	reported	textfile	7	50539036	0.007054	1.18E-11	524523	lymphocyt	TRUE	reported	igd	8.78E-05	46.06993	2	TRUE
rs1523178	C	A	C	A	-0.02569	0.024805	0.22113	0.175228	FALSE	FALSE	FALSE	I9_PULMEN	0.209032	0.019746	pulmonary	TRUE	reported	textfile	13	50652456	0.002292	3.86E-29	523058	lymphocyt	TRUE	reported	igd	0.00024	125.6703	2	TRUE
rs1533065	T	C	T	C	0.01668	-0.0061	0.345231	0.296749	FALSE	FALSE	FALSE	I9_PULMEN	0.710102	0.016417	pulmonary	TRUE	reported	textfile	8	22785768	0.001987	4.95E-17	524918	lymphocyt	TRUE	reported	igd	0.000134	70.46845	2	TRUE
rs1533298	T	C	T	C	0.030959	########	0.715263	0.795695	FALSE	FALSE	FALSE	I9_PULMEN	0.999839	0.018595	pulmonary	TRUE	reported	textfile	2	1.12E+08	0.002089	1.19E-49	523981	lymphocyt	TRUE	reported	igd	0.000419	219.6318	2	TRUE
rs1611236	A	G	A	G	-0.04235	-0.0091	0.322933	0.224527	FALSE	FALSE	FALSE	I9_PULMEN	0.612858	0.017986	pulmonary	TRUE	reported	textfile	6	29748690	0.002028	9.76E-97	522432	lymphocyt	TRUE	reported	igd	0.000834	436.0831	2	TRUE
rs1637366	G	C	G	C	0.01416	0.016558	0.289361	0.259822	FALSE	TRUE	FALSE	I9_PULMEN	0.338797	0.017311	pulmonary	TRUE	reported	textfile	7	74360784	0.002205	1.40E-10	480314	lymphocyt	TRUE	reported	igd	8.59E-05	41.23894	2	TRUE
rs1677490	C	G	C	G	0.015901	0.057518	0.7912	0.824666	FALSE	TRUE	FALSE	I9_PULMEN	0.00392	0.01994	pulmonary	TRUE	reported	textfile	16	9028541	0.002345	1.25E-11	524921	lymphocyt	TRUE	reported	igd	8.76E-05	45.97921	2	TRUE
rs1698630	A	G	A	G	-0.03227	0.031223	0.116161	0.179759	FALSE	FALSE	FALSE	I9_PULMEN	0.109828	0.019527	pulmonary	TRUE	reported	textfile	22	28717032	0.002955	9.95E-28	524920	lymphocyt	TRUE	reported	igd	0.000227	119.2859	2	TRUE
rs1700589	A	G	A	G	-0.02431	0.010211	0.184405	0.233377	FALSE	FALSE	FALSE	I9_PULMEN	0.564443	0.017719	pulmonary	TRUE	reported	textfile	4	83547862	0.001766	4.43E-43	523978	lymphocyt	TRUE	reported	igd	0.000362	189.5214	2	TRUE
rs1704143	C	A	C	A	0.040938	0.010695	0.056517	0.030641	FALSE	FALSE	FALSE	I9_PULMEN	0.805116	0.043346	pulmonary	TRUE	reported	textfile	12	1.02E+08	0.004104	2.11E-23	523981	lymphocyt	TRUE	reported	igd	0.00019	99.50316	2	TRUE
rs1705581	C	T	C	T	-0.02973	0.030942	0.252258	0.317451	FALSE	FALSE	FALSE	I9_PULMEN	0.055299	0.016145	pulmonary	TRUE	reported	textfile	5	1.58E+08	0.002177	2.15E-42	523521	lymphocyt	TRUE	reported	igd	0.000356	186.4466	2	TRUE
rs1709302	T	C	T	C	-0.02187	0.023466	0.101478	0.077253	FALSE	FALSE	FALSE	I9_PULMEN	0.40293	0.028056	pulmonary	TRUE	reported	textfile	20	34318911	0.003134	3.13E-12	524923	lymphocyt	TRUE	reported	igd	9.27E-05	48.68761	2	TRUE
rs1713874	G	A	G	A	-0.01345	0.003229	0.214538	0.198514	FALSE	FALSE	FALSE	I9_PULMEN	0.864155	0.018872	pulmonary	TRUE	reported	textfile	7	18224597	0.002312	6.13E-09	523520	lymphocyt	TRUE	reported	igd	6.46E-05	33.84288	2	TRUE
rs1718015	C	T	C	T	-0.01113	-0.00564	0.394048	0.344958	FALSE	FALSE	FALSE	I9_PULMEN	0.720731	0.015768	pulmonary	TRUE	reported	textfile	8	1.06E+08	0.001936	9.21E-09	524918	lymphocyt	TRUE	reported	igd	6.30E-05	33.05049	2	TRUE
rs1722904	T	C	T	C	-0.01917	-0.0149	0.210111	0.171096	FALSE	FALSE	FALSE	I9_PULMEN	0.45562	0.019969	pulmonary	TRUE	reported	textfile	16	11062936	0.002326	1.78E-16	524920	lymphocyt	TRUE	reported	igd	0.000129	67.93816	2	TRUE
rs1736664	C	T	C	T	0.014557	-0.00552	0.537795	0.562316	FALSE	FALSE	FALSE	I9_PULMEN	0.714687	0.01509	pulmonary	TRUE	reported	textfile	2	11344504	0.001889	1.35E-14	523981	lymphocyt	TRUE	reported	igd	0.000113	59.3852	2	TRUE
rs1750769	T	A	T	A	0.016236	-0.00549	0.222733	0.19767	FALSE	TRUE	FALSE	I9_PULMEN	0.770369	0.018817	pulmonary	TRUE	reported	textfile	15	83793622	0.002275	9.86E-13	524917	lymphocyt	TRUE	reported	igd	9.70E-05	50.93234	2	TRUE
rs175714	C	T	C	T	0.026141	0.0025	0.577477	0.514134	FALSE	FALSE	FALSE	I9_PULMEN	0.867828	0.015024	pulmonary	TRUE	reported	textfile	14	75981856	0.00193	9.20E-42	523523	lymphocyt	TRUE	reported	igd	0.00035	183.4544	2	TRUE
rs1770326	T	A	T	A	0.009635	-0.01652	0.188591	0.136068	FALSE	TRUE	FALSE	I9_PULMEN	0.45583	0.022154	pulmonary	TRUE	reported	textfile	4	88058337	0.001731	2.67E-08	524921	lymphocyt	TRUE	reported	igd	5.90E-05	30.98187	2	TRUE
rs1771000	A	G	A	G	0.020604	0.041257	0.18168	0.125884	FALSE	FALSE	FALSE	I9_PULMEN	0.06705	0.022528	pulmonary	TRUE	reported	textfile	6	1.53E+08	0.002451	4.45E-17	523514	lymphocyt	TRUE	reported	igd	0.000135	70.6667	2	TRUE
rs1807669	T	C	T	C	-0.02707	0.003168	0.793541	0.741196	FALSE	FALSE	FALSE	I9_PULMEN	0.853397	0.017144	pulmonary	TRUE	reported	textfile	22	42122926	0.002346	9.00E-31	524895	lymphocyt	TRUE	reported	igd	0.000254	133.1825	2	TRUE
rs1822534	G	A	G	A	-0.03441	-0.01267	0.393993	0.352954	FALSE	FALSE	FALSE	I9_PULMEN	0.421003	0.015741	pulmonary	TRUE	reported	textfile	3	12266804	0.001932	7.22E-71	524914	lymphocyt	TRUE	reported	igd	0.000604	317.1779	2	TRUE
rs1878569	A	G	A	G	0.048221	0.016203	0.038815	0.065901	FALSE	FALSE	FALSE	I9_PULMEN	0.594807	0.030463	pulmonary	TRUE	reported	textfile	15	90555181	0.005006	6.24E-22	524918	lymphocyt	TRUE	reported	igd	0.000177	92.78742	2	TRUE
rs1883801	G	A	G	A	-0.01702	-0.03178	0.195421	0.142536	FALSE	FALSE	FALSE	I9_PULMEN	0.141802	0.021631	pulmonary	TRUE	reported	textfile	20	10647679	0.002443	3.39E-12	524922	lymphocyt	TRUE	reported	igd	9.24E-05	48.50822	2	TRUE
rs1893033	T	C	T	C	-0.02819	0.061282	0.074578	0.027291	FALSE	FALSE	FALSE	I9_PULMEN	0.178518	0.045552	pulmonary	TRUE	reported	textfile	11	1.19E+08	0.003607	5.70E-15	523065	lymphocyt	TRUE	reported	igd	0.000117	61.0926	2	TRUE
rs1893592	C	A	C	A	0.014656	0.013468	0.295994	0.315946	FALSE	FALSE	FALSE	I9_PULMEN	0.404989	0.016173	pulmonary	TRUE	reported	textfile	21	43855067	0.002084	2.13E-12	523521	lymphocyt	TRUE	reported	igd	9.45E-05	49.45769	2	TRUE
rs1923403	G	A	G	A	0.020005	-0.00242	0.922075	0.948737	FALSE	FALSE	FALSE	I9_PULMEN	0.943065	0.033943	pulmonary	TRUE	reported	textfile	10	4160744	0.003543	1.68E-08	524921	lymphocyt	TRUE	reported	igd	6.07E-05	31.88109	2	TRUE
rs1966865	A	G	A	G	0.027118	0.017601	0.317295	0.286887	FALSE	FALSE	FALSE	I9_PULMEN	0.288136	0.01657	pulmonary	TRUE	reported	textfile	14	64975861	0.002032	1.36E-40	524921	lymphocyt	TRUE	reported	igd	0.000339	178.101	2	TRUE
rs1971429	A	C	A	C	0.012768	0.018283	0.728952	0.680555	FALSE	FALSE	FALSE	I9_PULMEN	0.256365	0.016108	pulmonary	TRUE	reported	textfile	9	33776182	0.002127	2.01E-09	524922	lymphocyt	TRUE	reported	igd	6.86E-05	36.03372	2	TRUE
rs1976055	A	C	A	C	-0.01716	-0.0065	0.156208	0.137119	FALSE	FALSE	FALSE	I9_PULMEN	0.765258	0.021757	pulmonary	TRUE	reported	textfile	2	1.12E+08	0.002597	4.05E-11	523982	lymphocyt	TRUE	reported	igd	8.33E-05	43.67071	2	TRUE
rs1991431	A	G	A	G	0.023776	-0.00673	0.440507	0.453114	FALSE	FALSE	FALSE	I9_PULMEN	0.654969	0.015066	pulmonary	TRUE	reported	textfile	3	1.41E+08	0.001903	8.91E-36	524918	lymphocyt	TRUE	reported	igd	0.000297	156.0984	2	TRUE
rs2055101	T	C	T	C	-0.01341	0.013299	0.527164	0.497489	FALSE	FALSE	FALSE	I9_PULMEN	0.375595	0.015009	pulmonary	TRUE	reported	textfile	8	1.2E+08	0.001901	1.84E-12	523978	lymphocyt	TRUE	reported	igd	9.49E-05	49.73903	2	TRUE
rs2057340	G	A	G	A	0.013805	0.022403	0.65339	0.756111	FALSE	FALSE	FALSE	I9_PULMEN	0.201271	0.01753	pulmonary	TRUE	reported	textfile	14	35848774	0.001995	4.66E-12	524919	lymphocyt	TRUE	reported	igd	9.12E-05	47.88344	2	TRUE
rs2065500	G	A	G	A	-0.04804	-0.02283	0.159764	0.161614	FALSE	FALSE	FALSE	I9_PULMEN	0.264414	0.020456	pulmonary	TRUE	reported	textfile	9	22145694	0.002582	3.29E-77	523520	lymphocyt	TRUE	reported	igd	0.000661	346.1719	2	TRUE
rs2068485	G	T	G	T	0.010843	0.008403	0.446837	0.474131	FALSE	FALSE	FALSE	I9_PULMEN	0.57584	0.015019	pulmonary	TRUE	reported	textfile	12	1868527	0.001945	2.55E-08	524921	lymphocyt	TRUE	reported	igd	5.92E-05	31.07836	2	TRUE
rs2089979	G	A	G	A	-0.03454	0.016584	0.414403	0.432492	FALSE	FALSE	FALSE	I9_PULMEN	0.2728	0.015123	pulmonary	TRUE	reported	textfile	3	1.97E+08	0.001922	4.10E-72	523520	lymphocyt	TRUE	reported	igd	0.000616	322.932	2	TRUE
rs2100139	C	T	C	T	0.015201	0.022529	0.65839	0.632802	FALSE	FALSE	FALSE	I9_PULMEN	0.149504	0.015631	pulmonary	TRUE	reported	textfile	3	1.09E+08	0.00199	2.32E-14	523524	lymphocyt	TRUE	reported	igd	0.000111	58.34941	2	TRUE
rs2145270	T	C	T	C	-0.01083	0.01285	0.618853	0.688116	FALSE	FALSE	FALSE	I9_PULMEN	0.427541	0.016196	pulmonary	TRUE	reported	textfile	20	6621685	0.001946	2.70E-08	524452	lymphocyt	TRUE	reported	igd	5.90E-05	30.95487	2	TRUE
rs2161118	C	T	C	T	0.011343	0.002386	0.327972	0.432994	FALSE	FALSE	FALSE	I9_PULMEN	0.875459	0.015225	pulmonary	TRUE	reported	textfile	5	1.73E+08	0.002075	4.74E-08	523841	lymphocyt	TRUE	reported	igd	5.70E-05	29.88257	2	TRUE
rs2180369	C	T	C	T	0.020125	0.005377	0.110884	0.092305	FALSE	FALSE	FALSE	I9_PULMEN	0.835408	0.02588	pulmonary	TRUE	reported	textfile	14	93516465	0.003138	1.47E-10	480040	lymphocyt	TRUE	reported	igd	8.57E-05	41.13051	2	TRUE
rs2230604	T	C	T	C	0.030004	0.024624	0.084354	0.127473	FALSE	FALSE	FALSE	I9_PULMEN	0.273347	0.022479	pulmonary	TRUE	reported	textfile	20	49196284	0.003451	3.65E-18	523518	lymphocyt	TRUE	reported	igd	0.000144	75.59042	2	TRUE

rs2270968	G	T	G	T	-0.01283	0.001273	0.738399	0.73994	FALSE	FALSE	FALSE	I9_PULMEN	0.940519	0.017063	pulmonary	TRUE	reported	textfile	3	1.83E+08	0.002149	2.46E-09	523523	lymphocyt	TRUE	reported	igd	6.81E-05	35.63782	2	TRUE
rs2273215	A	G	A	G	-0.01496	0.015993	0.458202	0.39013	FALSE	FALSE	FALSE	I9_PULMEN	0.300117	0.015434	pulmonary	TRUE	reported	textfile	6	1.71E+08	0.001984	5.04E-14	480499	lymphocyt	TRUE	reported	igd	0.000118	56.82583	2	TRUE
rs2275902	C	G	C	G	-0.01611	0.002533	0.297647	0.273305	FALSE	TRUE	FALSE	I9_PULMEN	0.880114	0.016793	pulmonary	TRUE	reported	textfile	1	79357360	0.002059	5.22E-15	524462	lymphocyt	TRUE	reported	igd	0.000117	61.24046	2	TRUE
rs2276566	C	G	C	G	-0.02525	0.028367	0.100134	0.063199	FALSE	TRUE	FALSE	I9_PULMEN	0.355338	0.030691	pulmonary	TRUE	reported	textfile	2	2.34E+08	0.003159	1.40E-15	523983	lymphocyt	TRUE	reported	igd	0.000122	63.8732	2	TRUE
rs2282680	T	C	T	C	-0.01367	-0.03146	0.289428	0.20049	FALSE	FALSE	FALSE	I9_PULMEN	0.093304	0.018746	pulmonary	TRUE	reported	textfile	4	72608364	0.002079	4.98E-11	524916	lymphocyt	TRUE	reported	igd	8.24E-05	43.24675	2	TRUE
rs2282718	A	G	A	G	-0.01756	0.014741	0.372092	0.404961	FALSE	FALSE	FALSE	I9_PULMEN	0.336133	0.015326	pulmonary	TRUE	reported	textfile	1	25241056	0.002016	3.25E-18	522430	lymphocyt	TRUE	reported	igd	0.000145	75.8607	2	TRUE
rs2291900	A	G	A	G	0.016877	-0.03023	0.29054	0.355309	FALSE	FALSE	FALSE	I9_PULMEN	0.05471	0.015733	pulmonary	TRUE	reported	textfile	3	1.71E+08	0.002114	1.49E-15	523518	lymphocyt	TRUE	reported	igd	0.000122	63.73513	2	TRUE
rs2294421	C	T	C	T	-0.01072	-0.01281	0.431921	0.367042	FALSE	FALSE	FALSE	I9_PULMEN	0.413325	0.015654	pulmonary	TRUE	reported	textfile	6	11753146	0.001911	2.07E-08	524922	lymphocyt	TRUE	reported	igd	6.00E-05	31.47368	2	TRUE
rs2294630	G	A	G	A	0.012992	-0.00752	0.493105	0.572431	FALSE	FALSE	FALSE	I9_PULMEN	0.621159	0.015207	pulmonary	TRUE	reported	textfile	1	19981791	0.001899	8.15E-12	520691	lymphocyt	TRUE	reported	igd	8.99E-05	46.80588	2	TRUE
rs2294752	G	T	G	T	0.012451	-0.01687	0.238115	0.204721	FALSE	FALSE	FALSE	I9_PULMEN	0.363829	0.018576	pulmonary	TRUE	reported	textfile	1	40879952	0.002215	1.95E-08	523982	lymphocyt	TRUE	reported	igd	6.03E-05	31.59798	2	TRUE
rs2294861	T	C	T	C	0.019028	-0.00372	0.294117	0.238155	FALSE	FALSE	FALSE	I9_PULMEN	0.832597	0.017613	pulmonary	TRUE	reported	textfile	6	53136516	0.002069	3.84E-20	524919	lymphocyt	TRUE	reported	igd	0.000161	84.57921	2	TRUE
rs2295797	C	T	C	T	0.020015	-0.01427	0.295417	0.245191	FALSE	FALSE	FALSE	I9_PULMEN	0.412998	0.017426	pulmonary	TRUE	reported	textfile	9	35722282	0.002073	4.99E-22	524918	lymphocyt	TRUE	reported	igd	0.000178	93.22039	2	TRUE
rs2297508	G	C	G	C	-0.02243	-0.02136	0.647622	0.60578	FALSE	TRUE	FALSE	I9_PULMEN	0.165411	0.015398	pulmonary	TRUE	reported	textfile	17	17715317	0.002068	2.20E-27	481438	lymphocyt	TRUE	reported	igd	0.000244	117.6612	2	TRUE
rs2298552	T	C	T	C	0.016509	-0.02483	0.52553	0.511244	FALSE	FALSE	FALSE	I9_PULMEN	0.09829	0.015019	pulmonary	TRUE	reported	textfile	11	60264947	0.001891	2.71E-18	523520	lymphocyt	TRUE	reported	igd	0.000146	76.21787	2	TRUE
rs2298979	A	G	A	G	-0.01957	-0.01087	0.405953	0.370064	FALSE	FALSE	FALSE	I9_PULMEN	0.484781	0.015554	pulmonary	TRUE	reported	textfile	4	1.11E+08	0.001926	3.20E-24	524919	lymphocyt	TRUE	reported	igd	0.000197	103.2341	2	TRUE
rs2313532	A	G	A	G	-0.01431	-0.012	0.40082	0.378475	FALSE	FALSE	FALSE	I9_PULMEN	0.437933	0.015467	pulmonary	TRUE	reported	textfile	9	1.34E+08	0.001943	1.87E-13	524909	lymphocyt	TRUE	reported	igd	0.000103	54.20364	2	TRUE
rs2322830	C	T	C	T	-0.0107	-0.01585	0.580473	0.541455	FALSE	FALSE	FALSE	I9_PULMEN	0.293484	0.015083	pulmonary	TRUE	reported	textfile	3	4745230	0.001916	2.42E-08	524919	lymphocyt	TRUE	reported	igd	5.94E-05	31.18709	2	TRUE
rs2327531	G	A	G	A	0.016613	0.026015	0.20699	0.108265	FALSE	FALSE	FALSE	I9_PULMEN	0.280565	0.02411	pulmonary	TRUE	reported	textfile	6	1.35E+08	0.002328	9.93E-13	524921	lymphocyt	TRUE	reported	igd	9.70E-05	50.92471	2	TRUE
rs2369391	C	A	C	A	-0.01699	0.012129	0.494127	0.457777	FALSE	FALSE	FALSE	I9_PULMEN	0.42153	0.01509	pulmonary	TRUE	reported	textfile	12	751609	0.001914	7.43E-19	522087	lymphocyt	TRUE	reported	igd	0.000151	78.75837	2	TRUE
rs2403246	G	C	G	C	0.011959	-0.00227	0.414485	0.395745	FALSE	TRUE	FALSE	I9_PULMEN	0.882479	0.015326	pulmonary	TRUE	reported	textfile	11	18101078	0.00192	4.81E-10	524904	lymphocyt	TRUE	reported	igd	7.39E-05	38.79588	2	TRUE
rs2412544	T	C	T	C	-0.01617	0.004832	0.393588	0.455648	FALSE	FALSE	FALSE	I9_PULMEN	0.748873	0.015093	pulmonary	TRUE	reported	textfile	15	40949526	0.001949	1.13E-16	523521	lymphocyt	TRUE	reported	igd	0.000131	68.82417	2	TRUE
rs2453582	T	C	T	C	0.017742	-0.02645	0.391346	0.281528	FALSE	FALSE	FALSE	I9_PULMEN	0.116302	0.01684	pulmonary	TRUE	reported	textfile	17	19439066	0.001989	4.88E-19	523980	lymphocyt	TRUE	reported	igd	0.000152	79.56717	2	TRUE
rs2453628	A	G	A	G	0.010777	0.020212	0.52576	0.57261	FALSE	FALSE	FALSE	I9_PULMEN	0.183445	0.015194	pulmonary	TRUE	reported	textfile	8	1.01E+08	0.001898	1.39E-08	524918	lymphocyt	TRUE	reported	igd	6.14E-05	32.24049	2	TRUE
rs2462017	A	G	A	G	0.013995	-0.03061	0.652335	0.723221	FALSE	FALSE	FALSE	I9_PULMEN	0.068	0.016775	pulmonary	TRUE	reported	textfile	10	30486133	0.002002	2.87E-12	524922	lymphocyt	TRUE	reported	igd	9.31E-05	48.86704	2	TRUE
rs246524	C	G	C	G	0.012404	0.000462	0.318481	0.345694	FALSE	TRUE	FALSE	I9_PULMEN	0.976649	0.015776	pulmonary	TRUE	reported	textfile	5	52272953	0.002022	8.79E-10	524919	lymphocyt	TRUE	reported	igd	7.17E-05	37.6322	2	TRUE
rs247557	G	T	G	T	-0.011	-0.02416	0.52424	0.494477	FALSE	FALSE	FALSE	I9_PULMEN	0.10729	0.015002	pulmonary	TRUE	reported	textfile	5	88710112	0.00189	6.05E-09	524920	lymphocyt	TRUE	reported	igd	6.45E-05	33.85503	2	TRUE
rs247826	T	C	T	C	0.038651	-0.00998	0.220693	0.289212	FALSE	FALSE	FALSE	I9_PULMEN	0.552291	0.016786	pulmonary	TRUE	reported	textfile	16	84582965	0.002294	1.19E-63	523516	lymphocyt	TRUE	reported	igd	0.000542	283.8788	2	TRUE
rs2489279	G	C	G	C	0.017602	0.022944	0.333667	0.287677	FALSE	TRUE	FALSE	I9_PULMEN	0.166127	0.016569	pulmonary	TRUE	reported	textfile	1	1.82E+08	0.001997	1.28E-18	524891	lymphocyt	TRUE	reported	igd	0.000148	77.6902	2	TRUE
rs251391	T	C	T	C	0.019773	-0.01799	0.747429	0.831522	FALSE	FALSE	FALSE	I9_PULMEN	0.37079	0.020097	pulmonary	TRUE	reported	textfile	5	1.28E+08	0.002179	1.21E-19	524923	lymphocyt	TRUE	reported	igd	0.000157	82.34344	2	TRUE
rs251398	C	T	C	T	-0.0173	-0.00475	0.446922	0.404495	FALSE	FALSE	FALSE	I9_PULMEN	0.756086	0.015298	pulmonary	TRUE	reported	textfile	5	67509801	0.001922	2.39E-19	524921	lymphocyt	TRUE	reported	igd	0.000154	81.03716	2	TRUE
rs2542573	C	T	C	T	-0.01482	-0.02229	0.330988	0.359057	FALSE	FALSE	FALSE	I9_PULMEN	0.15512	0.015678	pulmonary	TRUE	reported	textfile	2	54046646	0.002005	1.52E-13	523524	lymphocyt	TRUE	reported	igd	0.000104	54.627	2	TRUE
rs2548256	T	G	T	G	0.014003	0.014497	0.62009	0.597698	FALSE	FALSE	FALSE	I9_PULMEN	0.343761	0.015312	pulmonary	TRUE	reported	textfile	5	1E+08	0.001946	6.49E-13	523841	lymphocyt	TRUE	reported	igd	9.88E-05	51.77914	2	TRUE
rs2569693	T	C	T	C	-0.042	-0.01675	0.384414	0.372719	FALSE	FALSE	FALSE	I9_PULMEN	0.280996	0.015535	pulmonary	TRUE	reported	textfile	19	10399904	0.002018	3.36E-96	480040	lymphocyt	TRUE	reported	igd	0.000902	433.2073	2	TRUE
rs259981	A	T	A	T	-0.02711	-0.00067	0.360441	0.35935	FALSE	TRUE	FALSE	I9_PULMEN	0.96584	0.015683	pulmonary	TRUE	reported	textfile	20	57748369	0.001992	3.91E-42	523982	lymphocyt	TRUE	reported	igd	0.000353	185.1478	2	TRUE
rs2665960	G	A	G	A	0.012196	-0.02285	0.668615	0.738446	FALSE	FALSE	FALSE	I9_PULMEN	0.183035	0.017165	pulmonary	TRUE	reported	textfile	17	74024711	0.002071	4.03E-09	523980	lymphocyt	TRUE	reported	igd	6.62E-05	34.67951	2	TRUE
rs2710804	C	T	C	T	0.013802	0.02718	0.376124	0.353858	FALSE	FALSE	FALSE	I9_PULMEN	0.083123	0.015685	pulmonary	TRUE	reported	textfile	7	36084529	0.001953	1.63E-12	523521	lymphocyt	TRUE	reported	igd	9.54E-05	49.94338	2	TRUE
rs2727487	C	A	C	A	-0.01263	0.008525	0.629239	0.63908	FALSE	FALSE	FALSE	I9_PULMEN	0.585024	0.015612	pulmonary	TRUE	reported	textfile	7	1.3E+08	0.001955	1.08E-10	524921	lymphocyt	TRUE	reported	igd	7.95E-05	41.74268	2	TRUE
rs2738783	G	T	G	T	-0.01565	0.027158	0.78963	0.843684	FALSE	FALSE	FALSE	I9_PULMEN	0.189489	0.020698	pulmonary	TRUE	reported	textfile	20	62308612	0.00243	1.24E-10	481444	lymphocyt	TRUE	reported	igd	8.61E-05	41.47237	2	TRUE
rs2755253	T	C	T	C	-0.0309	0.019069	0.70898	0.630079	FALSE	FALSE	FALSE	I9_PULMEN	0.219248	0.015522	pulmonary	TRUE	reported	textfile	1	67470843	0.002072	2.87E-50	524922	lymphocyt	TRUE	reported	igd	0.000424	222.4437	2	TRUE
rs2807303	A	G	A	G	0.021641	-0.00012	0.346234	0.317684	FALSE	FALSE	FALSE	I9_PULMEN	0.993929	0.016142	pulmonary	TRUE	reported	textfile	9	82187095	0.001999	2.79E-27	524903	lymphocyt	TRUE	reported	igd	0.000223	117.1999	2	TRUE
rs2847468	T	C	T	C	-0.01194	0.014725	0.26371	0.310783	FALSE	FALSE	FALSE	I9_PULMEN	0.365031	0.016256	pulmonary	TRUE	reported	textfile	13	1.11E+08	0.002151	2.94E-08	523975	lymphocyt	TRUE	reported	igd	5.88E-05	30.78665	2	TRUE
rs2853343	T	C	T	C	-0.02507	0.0078	0.699647	0.677209	FALSE	FALSE	FALSE	I9_PULMEN	0.628265	0.016111	pulmonary	TRUE	reported	textfile	12	1.24E+08	0.002098	7.17E-33	523506	lymphocyt	TRUE	reported	igd	0.000273	142.835	2	TRUE
rs2853937	A	T	A	T	0.038051	0.005044	0.319096	0.327228	FALSE	TRUE	FALSE	I9_PULMEN	0.751943	0.015958	pulmonary	TRUE	reported	textfile	15	91021412	0.002032	3.38E-78	524919	lymphocyt	TRUE	reported	igd	0.000668	350.6575	2	TRUE
rs2853951	T	C	T	C	0.075987	-0.04311	0.597093	0.62119	FALSE	FALSE	FALSE	I9_PULMEN	0.005004	0.015361	pulmonary	TRUE	reported	textfile	6	31236115	0.001929	1.00E-200	523844	lymphocyt	TRUE	reported	igd	0.002953	1551.717	2	TRUE

rs2858874	T	A	T	A	0.02245	0.021023	0.205718	0.15691	FALSE	TRUE	FALSE	I9_PULMEM	0.30631	0.02055	pulmonary	TRUE	reported	textfile	8	10647044	0.002348	1.25E-21	521983	lymphocyt	TRUE	reported	igd	0.000175	91.41866	2	TRUE
rs2871010	G	A	G	A	-0.01433	0.007794	0.683305	0.694313	FALSE	FALSE	FALSE	I9_PULMEM	0.633381	0.01634	pulmonary	TRUE	reported	textfile	5	54879845	0.002031	1.75E-12	524920	lymphocyt	TRUE	reported	igd	9.49E-05	49.80268	2	TRUE
rs2908425	A	G	A	G	0.014205	-0.00293	0.332187	0.380785	FALSE	FALSE	FALSE	I9_PULMEM	0.849954	0.015507	pulmonary	TRUE	reported	textfile	7	5569327	0.002044	3.78E-12	524917	lymphocyt	TRUE	reported	igd	9.20E-05	48.29688	2	TRUE
rs2980821	A	C	A	C	-0.01325	-0.02351	0.425051	0.413513	FALSE	FALSE	FALSE	I9_PULMEM	0.123109	0.015246	pulmonary	TRUE	reported	textfile	8	40028306	0.001916	4.97E-12	523516	lymphocyt	TRUE	reported	igd	9.13E-05	47.78716	2	TRUE
rs2980888	C	T	C	T	-0.01534	-0.00112	0.697079	0.74081	FALSE	FALSE	FALSE	I9_PULMEM	0.947911	0.017095	pulmonary	TRUE	reported	textfile	8	1.27E+08	0.002067	1.21E-13	522903	lymphocyt	TRUE	reported	igd	0.000105	55.07672	2	TRUE
rs2992263	A	G	A	G	-0.02029	0.00761	0.610009	0.719866	FALSE	FALSE	FALSE	I9_PULMEM	0.649056	0.016722	pulmonary	TRUE	reported	textfile	10	26741207	0.001941	1.58E-25	523844	lymphocyt	TRUE	reported	igd	0.000208	109.2295	2	TRUE
rs3012415	T	C	T	C	0.014327	-0.0219	0.822853	0.868747	FALSE	FALSE	FALSE	I9_PULMEM	0.326015	0.022296	pulmonary	TRUE	reported	textfile	6	1.7E+08	0.002574	2.65E-08	481443	lymphocyt	TRUE	reported	igd	6.43E-05	30.98073	2	TRUE
rs304154	C	T	C	T	0.011965	-0.01844	0.307959	0.326027	FALSE	FALSE	FALSE	I9_PULMEM	0.248799	0.015993	pulmonary	TRUE	reported	textfile	5	88120416	0.002045	5.00E-09	524920	lymphocyt	TRUE	reported	igd	6.52E-05	34.23238	2	TRUE
rs309375	A	C	A	C	0.019206	0.024254	0.574699	0.436222	FALSE	FALSE	FALSE	I9_PULMEM	0.110476	0.015196	pulmonary	TRUE	reported	textfile	4	1.24E+08	0.001951	7.84E-23	524919	lymphocyt	TRUE	reported	igd	0.000185	96.90756	2	TRUE
rs3118469	T	A	T	A	0.029198	-0.01187	0.299989	0.363973	FALSE	TRUE	FALSE	I9_PULMEM	0.446737	0.015595	pulmonary	TRUE	reported	textfile	10	6101129	0.00206	1.50E-45	524922	lymphocyt	TRUE	reported	igd	0.000383	200.8955	2	TRUE
rs3134754	G	A	G	A	-0.05045	0.01938	0.445616	0.403398	FALSE	FALSE	FALSE	I9_PULMEM	0.20484	0.015285	pulmonary	TRUE	reported	textfile	6	31216329	0.001903	8.67E-155	523842	lymphocyt	TRUE	reported	igd	0.00134	702.7905	2	TRUE
rs3136685	T	C	T	C	-0.02032	0.00036	0.16434	0.154714	FALSE	FALSE	FALSE	I9_PULMEM	0.986293	0.020979	pulmonary	TRUE	reported	textfile	17	38719799	0.002708	6.51E-14	522442	lymphocyt	TRUE	reported	igd	0.000108	56.27748	2	TRUE
rs332507	C	T	C	T	-0.01527	-0.01633	0.222163	0.168819	FALSE	FALSE	FALSE	I9_PULMEM	0.415364	0.020056	pulmonary	TRUE	reported	textfile	3	1.24E+08	0.002298	3.12E-11	523982	lymphocyt	TRUE	reported	igd	8.43E-05	44.15464	2	TRUE
rs3402507	G	A	G	A	-0.03281	0.00308	0.101398	0.062187	FALSE	FALSE	FALSE	I9_PULMEM	0.920702	0.03094	pulmonary	TRUE	reported	textfile	15	70033578	0.003133	1.25E-25	524921	lymphocyt	TRUE	reported	igd	0.000209	109.6438	2	TRUE
rs3403081	C	T	C	T	-0.0182	-0.00241	0.367406	0.374224	FALSE	FALSE	FALSE	I9_PULMEM	0.876153	0.015485	pulmonary	TRUE	reported	textfile	2	1.44E+08	0.001956	1.49E-20	523980	lymphocyt	TRUE	reported	igd	0.000165	86.52961	2	TRUE
rs3414054	C	G	C	G	0.017044	-0.01456	0.648484	0.601226	FALSE	TRUE	FALSE	I9_PULMEM	0.343049	0.015351	pulmonary	TRUE	reported	textfile	16	4105786	0.001996	1.40E-17	524889	lymphocyt	TRUE	reported	igd	0.000139	72.91558	2	TRUE
rs3422922	A	G	A	G	-0.04788	-0.10071	0.014131	0.007714	FALSE	FALSE	FALSE	I9_PULMEM	0.236859	0.085141	pulmonary	TRUE	reported	textfile	12	1.12E+08	0.008049	2.78E-09	523522	lymphocyt	TRUE	reported	igd	6.76E-05	35.38825	2	TRUE
rs3432394	C	T	C	T	0.022339	-0.03046	0.110592	0.143276	FALSE	FALSE	FALSE	I9_PULMEM	0.154629	0.021399	pulmonary	TRUE	reported	textfile	20	52192637	0.00304	2.08E-13	523521	lymphocyt	TRUE	reported	igd	0.000103	53.99812	2	TRUE
rs3442373	A	C	A	C	-0.01082	0.024557	0.266467	0.18228	FALSE	FALSE	FALSE	I9_PULMEM	0.205441	0.019394	pulmonary	TRUE	reported	textfile	3	27519761	0.001965	3.80E-08	488552	lymphocyt	TRUE	reported	igd	6.20E-05	30.29188	2	TRUE
rs3449937	A	G	A	G	0.011141	-0.01242	0.329853	0.329454	FALSE	FALSE	FALSE	I9_PULMEM	0.4384	0.016034	pulmonary	TRUE	reported	textfile	4	1.03E+08	0.002015	3.27E-08	524919	lymphocyt	TRUE	reported	igd	5.82E-05	30.57008	2	TRUE
rs3466205	C	G	C	G	0.014295	0.022389	0.299106	0.292681	FALSE	TRUE	FALSE	I9_PULMEM	0.176804	0.016576	pulmonary	TRUE	reported	textfile	11	68908815	0.002067	4.80E-12	524922	lymphocyt	TRUE	reported	igd	9.11E-05	47.82838	2	TRUE
rs3466488	A	C	A	C	0.020387	0.003152	0.159961	0.182833	FALSE	FALSE	FALSE	I9_PULMEM	0.870847	0.019388	pulmonary	TRUE	reported	textfile	10	94515203	0.002574	2.47E-15	524918	lymphocyt	TRUE	reported	igd	0.000119	62.73182	2	TRUE
rs3485093	A	G	A	G	0.048075	-0.01438	0.346116	0.319477	FALSE	FALSE	FALSE	I9_PULMEM	0.372318	0.01612	pulmonary	TRUE	reported	textfile	3	47063910	0.002086	1.63E-117	524908	lymphocyt	TRUE	reported	igd	0.001011	531.1392	2	TRUE
rs3501078	A	G	A	G	-0.02131	0.016302	0.130713	0.139711	FALSE	FALSE	FALSE	I9_PULMEM	0.451618	0.021657	pulmonary	TRUE	reported	textfile	9	86499923	0.002875	1.30E-13	523842	lymphocyt	TRUE	reported	igd	0.000105	54.9298	2	TRUE
rs3511415	G	A	G	A	0.024676	-0.01186	0.225583	0.318632	FALSE	FALSE	FALSE	I9_PULMEM	0.461103	0.016096	pulmonary	TRUE	reported	textfile	7	1.5E+08	0.00227	1.73E-27	524922	lymphocyt	TRUE	reported	igd	0.000225	118.167	2	TRUE
rs3512182	A	G	A	G	0.013147	-0.01134	0.258961	0.193787	FALSE	FALSE	FALSE	I9_PULMEM	0.551611	0.019045	pulmonary	TRUE	reported	textfile	7	23634985	0.002162	1.22E-09	524921	lymphocyt	TRUE	reported	igd	7.04E-05	36.97773	2	TRUE
rs3518687	A	G	A	G	0.031862	0.051686	0.213822	0.166183	FALSE	FALSE	FALSE	I9_PULMEM	0.009552	0.019943	pulmonary	TRUE	reported	textfile	17	4625892	0.002332	1.94E-42	523838	lymphocyt	TRUE	reported	igd	0.000356	186.6755	2	TRUE
rs3534575	G	C	G	C	-0.02652	-0.00238	0.207147	0.172708	FALSE	TRUE	FALSE	I9_PULMEM	0.904383	0.019799	pulmonary	TRUE	reported	textfile	7	22740513	0.002334	6.95E-30	524919	lymphocyt	TRUE	reported	igd	0.000246	129.0565	2	TRUE
rs3559243	C	G	C	G	0.108539	-0.132	0.027317	0.02496	FALSE	TRUE	FALSE	I9_PULMEM	0.008066	0.049823	pulmonary	TRUE	reported	textfile	3	71355240	0.006203	1.68E-68	523066	lymphocyt	TRUE	reported	igd	0.000585	306.1727	2	TRUE
rs3599384	C	A	C	A	0.038227	-0.00789	0.070154	0.102667	FALSE	FALSE	FALSE	I9_PULMEM	0.753881	0.025167	pulmonary	TRUE	reported	textfile	1	2.04E+08	0.00382	1.50E-23	524912	lymphocyt	TRUE	reported	igd	0.000191	100.141	2	TRUE
rs360017	G	A	G	A	-0.02111	0.021582	0.775253	0.777605	FALSE	FALSE	FALSE	I9_PULMEM	0.230819	0.018011	pulmonary	TRUE	reported	textfile	5	1.73E+08	0.002273	1.69E-20	523984	lymphocyt	TRUE	reported	igd	0.000165	86.22066	2	TRUE
rs3608435	A	G	A	G	-0.06072	0.021348	0.091708	0.085763	FALSE	FALSE	FALSE	I9_PULMEM	0.427994	0.026934	pulmonary	TRUE	reported	textfile	19	1079959	0.003409	6.60E-71	480042	lymphocyt	TRUE	reported	igd	0.00066	317.2231	2	TRUE
rs3610806	G	C	G	C	-0.02531	-0.00982	0.057188	0.021054	FALSE	TRUE	FALSE	I9_PULMEM	0.85026	0.052001	pulmonary	TRUE	reported	textfile	12	14409191	0.004077	5.54E-10	524923	lymphocyt	TRUE	reported	igd	7.34E-05	38.53298	2	TRUE
rs3731211	A	T	A	T	0.043598	-0.00558	0.719794	0.772015	FALSE	TRUE	FALSE	I9_PULMEM	0.754505	0.017851	pulmonary	TRUE	reported	textfile	9	21986847	0.002107	5.14E-95	524921	lymphocyt	TRUE	reported	igd	0.000815	428.1564	2	TRUE
rs3735485	G	A	G	A	0.044361	0.033871	0.844816	0.876107	FALSE	FALSE	FALSE	I9_PULMEM	0.136486	0.022747	pulmonary	TRUE	reported	textfile	7	45009341	0.002643	3.56E-63	524463	lymphocyt	TRUE	reported	igd	0.000537	281.7129	2	TRUE
rs3739756	C	T	C	T	-0.03185	0.020702	0.036302	0.017499	FALSE	FALSE	FALSE	I9_PULMEM	0.720779	0.05792	pulmonary	TRUE	reported	textfile	9	98775235	0.005047	2.88E-10	524918	lymphocyt	TRUE	reported	igd	7.58E-05	39.81202	2	TRUE
rs3744399	C	T	C	T	-0.02207	0.034139	0.143155	0.101834	FALSE	FALSE	FALSE	I9_PULMEM	0.169388	0.024843	pulmonary	TRUE	reported	textfile	17	7154582	0.002716	4.61E-16	523828	lymphocyt	TRUE	reported	igd	0.000126	66.0423	2	TRUE
rs3760456	T	C	T	C	-0.01472	0.017111	0.439121	0.475958	FALSE	FALSE	FALSE	I9_PULMEM	0.253973	0.015	pulmonary	TRUE	reported	textfile	17	27948844	0.001911	1.40E-14	524914	lymphocyt	TRUE	reported	igd	0.000113	59.33249	2	TRUE
rs3761846	T	C	T	C	0.020501	-0.01887	0.569525	0.515268	FALSE	FALSE	FALSE	I9_PULMEM	0.208985	0.015019	pulmonary	TRUE	reported	textfile	9	1.24E+08	0.001907	6.26E-27	524920	lymphocyt	TRUE	reported	igd	0.00022	115.5705	2	TRUE
rs3797727	A	T	A	T	0.019727	0.030585	0.112161	0.098089	FALSE	TRUE	FALSE	I9_PULMEM	0.226279	0.025277	pulmonary	TRUE	reported	textfile	5	1.11E+08	0.002999	4.94E-11	523508	lymphocyt	TRUE	reported	igd	8.26E-05	43.26807	2	TRUE
rs3810818	C	A	C	A	-0.01825	-0.01965	0.773356	0.755776	FALSE	FALSE	FALSE	I9_PULMEM	0.261199	0.017485	pulmonary	TRUE	reported	textfile	16	4432029	0.002353	9.13E-15	480038	lymphocyt	TRUE	reported	igd	0.000125	60.14297	2	TRUE
rs3812316	G	C	G	C	-0.01666	0.017007	0.127464	0.128182	FALSE	TRUE	FALSE	I9_PULMEM	0.447276	0.022379	pulmonary	TRUE	reported	textfile	7	73020337	0.002841	4.63E-09	524460	lymphocyt	TRUE	reported	igd	6.56E-05	34.39617	2	TRUE
rs3812849	C	A	C	A	0.04349	0.012813	0.266158	0.243082	FALSE	FALSE	FALSE	I9_PULMEM	0.466626	0.0176	pulmonary	TRUE	reported	textfile	13	74701736	0.002142	1.32E-91	524921	lymphocyt	TRUE	reported	igd	0.000785	412.2287	2	TRUE
rs3822030	T	G	T	G	0.011121	-0.0154	0.570762	0.630452	FALSE	FALSE	FALSE	I9_PULMEM	0.323673	0.015608	pulmonary	TRUE	reported	textfile	4	987343	0.002015	3.48E-08	473830	lymphocyt	TRUE	reported	igd	6.43E-05	30.46041	2	TRUE

rs3848132	A	T	A	T	-0.01177	0.014231	0.292109	0.299906	FALSE	TRUE	FALSE	I9_PULMEN	0.384613	0.016368	pulmonary	TRUE	reported	textfile	15	51106727	0.002084	1.66E-08	524920	lymphocyt	TRUE	reported	igd	6.08E-05	31.89202	2	TRUE
rs385182C	T	A	T	A	-0.01755	-0.01677	0.744961	0.725386	FALSE	TRUE	FALSE	I9_PULMEN	0.317948	0.016792	pulmonary	TRUE	reported	textfile	18	20461322	0.002175	7.37E-16	524919	lymphocyt	TRUE	reported	igd	0.000124	65.11538	2	TRUE
rs3992621	C	T	C	T	-0.01038	-0.03147	0.535606	0.611314	FALSE	FALSE	FALSE	I9_PULMEN	0.040483	0.015362	pulmonary	TRUE	reported	textfile	5	1.19E+08	0.001894	4.31E-08	524918	lymphocyt	TRUE	reported	igd	5.73E-05	30.05853	2	TRUE
rs412956C	A	C	A	C	-0.01118	0.00155	0.369955	0.369506	FALSE	FALSE	FALSE	I9_PULMEN	0.920301	0.015492	pulmonary	TRUE	reported	textfile	15	81978452	0.001961	1.21E-08	524915	lymphocyt	TRUE	reported	igd	6.19E-05	32.51488	2	TRUE
rs4131727	T	C	T	C	-0.01847	0.010631	0.177256	0.104916	FALSE	FALSE	FALSE	I9_PULMEN	0.664912	0.024544	pulmonary	TRUE	reported	textfile	10	98369755	0.002479	9.82E-14	524921	lymphocyt	TRUE	reported	igd	0.000106	55.50499	2	TRUE
rs419470	G	A	G	A	0.014977	-0.01134	0.716626	0.723318	FALSE	FALSE	FALSE	I9_PULMEN	0.499698	0.016802	pulmonary	TRUE	reported	textfile	5	16625591	0.002097	9.50E-13	524922	lymphocyt	TRUE	reported	igd	9.72E-05	51.00951	2	TRUE
rs4319365	A	G	A	G	-0.01934	0.006461	0.544871	0.483971	FALSE	FALSE	FALSE	I9_PULMEN	0.668236	0.015076	pulmonary	TRUE	reported	textfile	1	56907720	0.001904	3.26E-24	524920	lymphocyt	TRUE	reported	igd	0.000197	103.1757	2	TRUE
rs4368798	G	A	G	A	0.012354	0.014193	0.426501	0.361414	FALSE	FALSE	FALSE	I9_PULMEN	0.362977	0.015601	pulmonary	TRUE	reported	textfile	6	26593471	0.001912	1.07E-10	522436	lymphocyt	TRUE	reported	igd	7.99E-05	41.7482	2	TRUE
rs4406174	T	C	T	C	0.018355	0.01443	0.101238	0.131436	FALSE	FALSE	FALSE	I9_PULMEN	0.516306	0.022233	pulmonary	TRUE	reported	textfile	5	1519079	0.003281	2.27E-08	477215	lymphocyt	TRUE	reported	igd	6.56E-05	31.29641	2	TRUE
rs4411786	C	T	C	T	-0.0451	0.00333	0.265295	0.257725	FALSE	FALSE	FALSE	I9_PULMEN	0.845756	0.017118	pulmonary	TRUE	reported	textfile	20	1930897	0.002147	6.39E-98	524923	lymphocyt	TRUE	reported	igd	0.00084	441.3114	2	TRUE
rs444613	G	A	G	A	0.015352	0.002128	0.42489	0.361327	FALSE	FALSE	FALSE	I9_PULMEN	0.891415	0.015585	pulmonary	TRUE	reported	textfile	5	1.51E+08	0.001911	1.00E-15	524917	lymphocyt	TRUE	reported	igd	0.000123	64.53672	2	TRUE
rs4497886	A	G	A	G	-0.07174	0.036176	0.075265	0.03843	FALSE	FALSE	FALSE	I9_PULMEN	0.365427	0.039971	pulmonary	TRUE	reported	textfile	2	1.12E+08	0.003963	3.47E-73	516197	lymphocyt	TRUE	reported	igd	0.000634	327.7247	2	TRUE
rs4530278	T	G	T	G	0.017139	-0.01819	0.598216	0.603036	FALSE	FALSE	FALSE	I9_PULMEN	0.236327	0.015357	pulmonary	TRUE	reported	textfile	19	33752994	0.00198	5.24E-18	524915	lymphocyt	TRUE	reported	igd	0.000143	74.9271	2	TRUE
rs4552893	T	C	T	C	-0.02053	0.030955	0.161765	0.132818	FALSE	FALSE	FALSE	I9_PULMEN	0.164479	0.022267	pulmonary	TRUE	reported	textfile	14	23793305	0.00258	1.85E-15	523980	lymphocyt	TRUE	reported	igd	0.000121	63.33174	2	TRUE
rs4578474	G	A	G	A	-0.0124	-0.00478	0.265376	0.263745	FALSE	FALSE	FALSE	I9_PULMEN	0.779071	0.017022	pulmonary	TRUE	reported	textfile	12	4346821	0.002144	7.47E-09	524920	lymphocyt	TRUE	reported	igd	6.38E-05	33.47128	2	TRUE
rs4619033	G	A	G	A	-0.01904	-0.00338	0.267859	0.283493	FALSE	FALSE	FALSE	I9_PULMEN	0.839247	0.016662	pulmonary	TRUE	reported	textfile	1	2.04E+08	0.002139	5.73E-19	523522	lymphocyt	TRUE	reported	igd	0.000151	79.2588	2	TRUE
rs464609	A	G	A	G	0.011801	0.019263	0.544347	0.54392	FALSE	FALSE	FALSE	I9_PULMEN	0.200341	0.015042	pulmonary	TRUE	reported	textfile	5	34654477	0.001899	5.30E-10	523520	lymphocyt	TRUE	reported	igd	7.38E-05	38.61767	2	TRUE
rs4660128	C	T	C	T	-0.02477	0.032628	0.896961	0.913693	FALSE	FALSE	FALSE	I9_PULMEN	0.22354	0.026806	pulmonary	TRUE	reported	textfile	1	2.36E+08	0.003138	3.12E-15	524922	lymphocyt	TRUE	reported	igd	0.000119	62.293	2	TRUE
rs4685105	G	A	G	A	-0.01073	0.010249	0.414708	0.41102	FALSE	FALSE	FALSE	I9_PULMEN	0.502405	0.015281	pulmonary	TRUE	reported	textfile	3	14320176	0.00194	3.21E-08	523981	lymphocyt	TRUE	reported	igd	5.84E-05	30.61386	2	TRUE
rs4696314	C	T	C	T	0.014209	0.019324	0.515076	0.481719	FALSE	FALSE	FALSE	I9_PULMEN	0.19817	0.015017	pulmonary	TRUE	reported	textfile	4	1.53E+08	0.001906	9.33E-14	522443	lymphocyt	TRUE	reported	igd	0.000106	55.57501	2	TRUE
rs4714431	C	A	C	A	-0.01081	0.018485	0.371057	0.318466	FALSE	FALSE	FALSE	I9_PULMEN	0.254868	0.016235	pulmonary	TRUE	reported	textfile	6	41168337	0.001969	4.15E-08	523520	lymphocyt	TRUE	reported	igd	5.75E-05	30.12989	2	TRUE
rs4718976	C	T	C	T	-0.01787	-0.00542	0.694537	0.602397	FALSE	FALSE	FALSE	I9_PULMEN	0.725808	0.015452	pulmonary	TRUE	reported	textfile	7	70113409	0.002085	1.10E-17	523522	lymphocyt	TRUE	reported	igd	0.00014	73.44914	2	TRUE
rs473701C	A	G	A	G	0.026819	-0.00929	0.228863	0.250207	FALSE	FALSE	FALSE	I9_PULMEN	0.594083	0.017432	pulmonary	TRUE	reported	textfile	8	41630447	0.002264	2.47E-32	524917	lymphocyt	TRUE	reported	igd	0.000267	140.3236	2	TRUE
rs4760278	A	C	A	C	-0.01403	0.016316	0.222685	0.220318	FALSE	FALSE	FALSE	I9_PULMEN	0.366536	0.018069	pulmonary	TRUE	reported	textfile	12	57771153	0.002269	6.48E-10	524916	lymphocyt	TRUE	reported	igd	7.28E-05	38.22811	2	TRUE
rs4789294	G	A	G	A	0.02652	-0.01145	0.219391	0.146738	FALSE	FALSE	FALSE	I9_PULMEN	0.588631	0.02118	pulmonary	TRUE	reported	textfile	17	74419177	0.002301	1.05E-30	524920	lymphocyt	TRUE	reported	igd	0.000253	132.8349	2	TRUE
rs4805881	C	A	C	A	-0.01503	0.016882	0.665288	0.6578	FALSE	FALSE	FALSE	I9_PULMEN	0.285299	0.0158	pulmonary	TRUE	reported	textfile	19	33896432	0.00203	1.41E-13	524922	lymphocyt	TRUE	reported	igd	0.000104	54.80354	2	TRUE
rs4812804	T	C	T	C	0.013444	-0.02097	0.198107	0.233973	FALSE	FALSE	FALSE	I9_PULMEN	0.238486	0.017785	pulmonary	TRUE	reported	textfile	20	42852273	0.002397	2.10E-08	523518	lymphocyt	TRUE	reported	igd	6.01E-05	31.45714	2	TRUE
rs4833031	C	T	C	T	0.017943	-0.00784	0.139012	0.10876	FALSE	FALSE	FALSE	I9_PULMEN	0.748755	0.024485	pulmonary	TRUE	reported	textfile	4	38292186	0.002737	5.70E-11	524921	lymphocyt	TRUE	reported	igd	8.19E-05	42.97727	2	TRUE
rs4849169	C	A	C	A	0.020898	-0.01285	0.482168	0.417741	FALSE	FALSE	FALSE	I9_PULMEN	0.398082	0.01521	pulmonary	TRUE	reported	textfile	2	1.14E+08	0.0019	4.14E-28	523977	lymphocyt	TRUE	reported	igd	0.000231	120.9764	2	TRUE
rs4869745	T	C	T	C	0.012563	-0.00048	0.28937	0.294912	FALSE	FALSE	FALSE	I9_PULMEN	0.976604	0.016485	pulmonary	TRUE	reported	textfile	6	1.52E+08	0.002081	1.61E-09	523507	lymphocyt	TRUE	reported	igd	6.96E-05	36.44525	2	TRUE
rs4880192	G	A	G	A	0.029356	-0.0228	0.720804	0.703602	FALSE	FALSE	FALSE	I9_PULMEN	0.170076	0.016616	pulmonary	TRUE	reported	textfile	9	1.4E+08	0.002198	1.16E-40	480501	lymphocyt	TRUE	reported	igd	0.000371	178.3761	2	TRUE
rs4907236	T	C	T	C	0.011477	0.034368	0.283222	0.199512	FALSE	FALSE	FALSE	I9_PULMEN	0.068333	0.018855	pulmonary	TRUE	reported	textfile	2	97187605	0.002095	4.41E-08	522893	lymphocyt	TRUE	reported	igd	5.74E-05	30.01145	2	TRUE
rs4925547	T	A	T	A	0.012002	-0.01111	0.402366	0.388805	FALSE	TRUE	FALSE	I9_PULMEN	0.471064	0.015417	pulmonary	TRUE	reported	textfile	1	2.48E+08	0.001924	4.55E-10	524916	lymphocyt	TRUE	reported	igd	7.41E-05	38.91307	2	TRUE
rs4940572	A	G	A	G	0.023283	-0.01491	0.136477	0.157981	FALSE	FALSE	FALSE	I9_PULMEN	0.472103	0.020735	pulmonary	TRUE	reported	textfile	18	60826343	0.002808	1.17E-16	522444	lymphocyt	TRUE	reported	igd	0.000132	68.75147	2	TRUE
rs494091	C	T	C	T	0.02706	0.007719	0.425556	0.476968	FALSE	FALSE	FALSE	I9_PULMEN	0.606979	0.015006	pulmonary	TRUE	reported	textfile	1	1.61E+08	0.001901	6.35E-46	524921	lymphocyt	TRUE	reported	igd	0.000386	202.6234	2	TRUE
rs4948492	T	C	T	C	-0.02192	0.000797	0.661671	0.752707	FALSE	FALSE	FALSE	I9_PULMEN	0.963438	0.017382	pulmonary	TRUE	reported	textfile	10	63719739	0.001998	5.69E-28	524920	lymphocyt	TRUE	reported	igd	0.000229	120.3398	2	TRUE
rs4948822	G	T	G	T	0.012732	0.018927	0.231555	0.266918	FALSE	FALSE	FALSE	I9_PULMEN	0.265015	0.016981	pulmonary	TRUE	reported	textfile	10	44513645	0.002238	1.32E-08	524920	lymphocyt	TRUE	reported	igd	6.17E-05	32.36469	2	TRUE
rs497295C	A	C	A	C	0.021567	-0.02763	0.107585	0.153782	FALSE	FALSE	FALSE	I9_PULMEN	0.185029	0.020848	pulmonary	TRUE	reported	textfile	2	2.31E+08	0.003043	1.42E-12	522575	lymphocyt	TRUE	reported	igd	9.61E-05	50.23124	2	TRUE
rs4976642	A	T	A	T	0.024419	-0.0056	0.831367	0.768249	FALSE	TRUE	FALSE	I9_PULMEN	0.751879	0.017723	pulmonary	TRUE	reported	textfile	5	1.77E+08	0.002519	3.44E-22	524910	lymphocyt	TRUE	reported	igd	0.000179	93.97185	2	TRUE
rs4987855	T	C	T	C	-0.02408	0.015933	0.096307	0.049514	FALSE	FALSE	FALSE	I9_PULMEN	0.646341	0.034724	pulmonary	TRUE	reported	textfile	18	60793549	0.003242	1.16E-13	523519	lymphocyt	TRUE	reported	igd	0.000105	55.15401	2	TRUE
rs501461	T	G	T	G	-0.01774	-0.00755	0.599659	0.586039	FALSE	FALSE	FALSE	I9_PULMEN	0.620166	0.015242	pulmonary	TRUE	reported	textfile	9	4039727	0.001941	6.76E-20	523522	lymphocyt	TRUE	reported	igd	0.00016	83.52289	2	TRUE
rs502647C	G	A	G	A	0.036884	-0.00966	0.517317	0.467937	FALSE	FALSE	FALSE	I9_PULMEN	0.520398	0.01503	pulmonary	TRUE	reported	textfile	4	1.04E+08	0.001892	1.45E-84	524922	lymphocyt	TRUE	reported	igd	0.000723	380.0424	2	TRUE
rs529002	G	T	G	T	-0.01135	-0.00534	0.550891	0.516151	FALSE	FALSE	FALSE	I9_PULMEN	0.723466	0.015084	pulmonary	TRUE	reported	textfile	2	1.7E+08	0.001933	4.46E-09	522443	lymphocyt	TRUE	reported	igd	6.60E-05	34.4646	2	TRUE
rs533483	A	G	A	G	-0.01433	-0.01186	0.244075	0.192282	FALSE	FALSE	FALSE	I9_PULMEN	0.534172	0.019078	pulmonary	TRUE	reported	textfile	1	2.35E+08	0.002238	1.58E-10	523519	lymphocyt	TRUE	reported	igd	7.83E-05	40.98158	2	TRUE

rs5593809	T	G	T	G	0.015967	0.009539	0.530458	0.513341	FALSE	FALSE	FALSE	I9_PULMEM	0.524955	0.015005	pulmonary	TRUE	reported	textfile	3	56620172	0.00189	3.16E-17	524921	lymphocyt	TRUE	reported	igd	0.000136	71.37094	2	TRUE
rs5597794	A	G	A	G	-0.03046	-0.01406	0.097555	0.08329	FALSE	FALSE	FALSE	I9_PULMEM	0.605197	0.027202	pulmonary	TRUE	reported	textfile	5	1.58E+08	0.003183	1.15E-21	524920	lymphocyt	TRUE	reported	igd	0.000174	91.5887	2	TRUE
rs5598228	A	G	A	G	0.061557	-0.00166	0.135291	0.096373	FALSE	FALSE	FALSE	I9_PULMEM	0.947543	0.025293	pulmonary	TRUE	reported	textfile	2	24188128	0.002764	8.71E-110	521154	lymphocyt	TRUE	reported	igd	0.000951	495.9948	2	TRUE
rs5609400	G	A	G	A	-0.0401	0.047746	0.042917	0.046825	FALSE	FALSE	FALSE	I9_PULMEM	0.17959	0.035578	pulmonary	TRUE	reported	textfile	8	21769432	0.004704	1.61E-17	523504	lymphocyt	TRUE	reported	igd	0.000139	72.6768	2	TRUE
rs5617961	T	G	T	G	0.031193	-0.02654	0.258737	0.263447	FALSE	FALSE	FALSE	I9_PULMEM	0.11935	0.017039	pulmonary	TRUE	reported	textfile	19	47695751	0.002167	6.22E-47	524913	lymphocyt	TRUE	reported	igd	0.000395	207.2024	2	TRUE
rs5625210	T	C	T	C	0.018921	0.007914	0.260661	0.229465	FALSE	FALSE	FALSE	I9_PULMEM	0.658103	0.017884	pulmonary	TRUE	reported	textfile	19	13123188	0.002172	3.12E-18	523978	lymphocyt	TRUE	reported	igd	0.000145	75.88691	2	TRUE
rs5630832	T	A	T	A	0.017813	0.008532	0.130681	0.150546	FALSE	TRUE	FALSE	I9_PULMEM	0.686302	0.021126	pulmonary	TRUE	reported	textfile	17	45819206	0.002809	2.36E-10	524916	lymphocyt	TRUE	reported	igd	7.66E-05	40.21323	2	TRUE
rs5685078	T	G	T	G	-0.02142	0.029526	0.077731	0.067209	FALSE	FALSE	FALSE	I9_PULMEM	0.327963	0.030183	pulmonary	TRUE	reported	textfile	3	1.96E+08	0.003625	3.55E-09	524923	lymphocyt	TRUE	reported	igd	6.65E-05	34.91574	2	TRUE
rs5826575	C	T	C	T	-0.01225	0.00123	0.52078	0.491804	FALSE	FALSE	FALSE	I9_PULMEM	0.934742	0.015021	pulmonary	TRUE	reported	textfile	5	1.24E+08	0.001914	1.61E-10	523981	lymphocyt	TRUE	reported	igd	7.81E-05	40.94246	2	TRUE
rs5840842	C	T	C	T	-0.02362	0.040635	0.186094	0.233658	FALSE	FALSE	FALSE	I9_PULMEM	0.021838	0.01772	pulmonary	TRUE	reported	textfile	4	57769824	0.002428	2.42E-22	523974	lymphocyt	TRUE	reported	igd	0.000181	94.62093	2	TRUE
rs5866731	T	C	T	C	-0.02322	0.013439	0.122978	0.084329	FALSE	FALSE	FALSE	I9_PULMEM	0.617735	0.026928	pulmonary	TRUE	reported	textfile	10	96524395	0.00289	9.91E-16	524920	lymphocyt	TRUE	reported	igd	0.000123	64.55458	2	TRUE
rs6001858	G	A	G	A	-0.01526	0.026498	0.735573	0.786022	FALSE	FALSE	FALSE	I9_PULMEM	0.14728	0.018285	pulmonary	TRUE	reported	textfile	22	40672078	0.002175	2.37E-12	524904	lymphocyt	TRUE	reported	igd	9.38E-05	49.24472	2	TRUE
rs6021231	C	T	C	T	-0.01146	-0.01903	0.470967	0.335215	FALSE	FALSE	FALSE	I9_PULMEM	0.232472	0.015942	pulmonary	TRUE	reported	textfile	20	50092287	0.001915	2.21E-09	523523	lymphocyt	TRUE	reported	igd	6.84E-05	35.81837	2	TRUE
rs603424	A	G	A	G	0.020453	-0.00415	0.174511	0.116051	FALSE	FALSE	FALSE	I9_PULMEM	0.860474	0.023618	pulmonary	TRUE	reported	textfile	10	1.02E+08	0.002498	2.78E-16	523520	lymphocyt	TRUE	reported	igd	0.000128	67.039	2	TRUE
rs6063335	C	T	C	T	-0.02173	0.003096	0.131836	0.115627	FALSE	FALSE	FALSE	I9_PULMEM	0.894804	0.023411	pulmonary	TRUE	reported	textfile	1	91500508	0.00279	7.19E-15	524922	lymphocyt	TRUE	reported	igd	0.000116	60.66098	2	TRUE
rs6072080	T	C	T	C	0.019842	0.018038	0.585279	0.581694	FALSE	FALSE	FALSE	I9_PULMEM	0.235738	0.015213	pulmonary	TRUE	reported	textfile	20	39260927	0.001942	1.77E-24	523835	lymphocyt	TRUE	reported	igd	0.000199	104.3929	2	TRUE
rs607465	G	A	G	A	0.034284	-0.02724	0.065545	0.074983	FALSE	FALSE	FALSE	I9_PULMEM	0.338339	0.028452	pulmonary	TRUE	reported	textfile	6	7129563	0.003842	4.83E-19	524919	lymphocyt	TRUE	reported	igd	0.000152	79.62814	2	TRUE
rs6103270	G	A	G	A	-0.01367	-0.0274	0.226397	0.192852	FALSE	FALSE	FALSE	I9_PULMEM	0.151185	0.019092	pulmonary	TRUE	reported	textfile	20	42015635	0.002275	1.91E-09	524922	lymphocyt	TRUE	reported	igd	6.88E-05	36.116	2	TRUE
rs611847	G	A	G	A	0.014579	-0.00625	0.635502	0.732047	FALSE	FALSE	FALSE	I9_PULMEM	0.712582	0.016965	pulmonary	TRUE	reported	textfile	20	3684022	0.002003	3.53E-13	522444	lymphocyt	TRUE	reported	igd	0.000101	52.97755	2	TRUE
rs6138719	T	C	T	C	0.031603	-0.00613	0.157437	0.188152	FALSE	FALSE	FALSE	I9_PULMEM	0.750437	0.019268	pulmonary	TRUE	reported	textfile	19	44260929	0.0026	5.81E-34	523981	lymphocyt	TRUE	reported	igd	0.000282	147.7435	2	TRUE
rs6175092	T	C	T	C	-0.0703	-0.06367	0.056331	0.033924	FALSE	FALSE	FALSE	I9_PULMEM	0.125305	0.041538	pulmonary	TRUE	reported	textfile	9	91495135	0.004108	1.36E-65	524920	lymphocyt	TRUE	reported	igd	0.000558	292.8186	2	TRUE
rs6177821	T	C	T	C	0.023251	0.044772	0.140899	0.122981	FALSE	FALSE	FALSE	I9_PULMEM	0.050436	0.022887	pulmonary	TRUE	reported	textfile	1	24235932	0.002746	2.67E-17	523977	lymphocyt	TRUE	reported	igd	0.000137	71.69363	2	TRUE
rs6185068	A	G	A	G	-0.03246	-0.03673	0.233502	0.14976	FALSE	FALSE	FALSE	I9_PULMEM	0.081248	0.021067	pulmonary	TRUE	reported	textfile	10	50252936	0.002245	2.59E-47	523843	lymphocyt	TRUE	reported	igd	0.000399	209.0303	2	TRUE
rs6186714	A	G	A	G	-0.01713	0.004747	0.194944	0.216033	FALSE	FALSE	FALSE	I9_PULMEM	0.795781	0.018342	pulmonary	TRUE	reported	textfile	11	1874892	0.002531	1.37E-11	481443	lymphocyt	TRUE	reported	igd	9.51E-05	45.79596	2	TRUE
rs6209199	G	A	G	A	-0.02233	0.017015	0.307469	0.312252	FALSE	FALSE	FALSE	I9_PULMEM	0.29387	0.01621	pulmonary	TRUE	reported	textfile	17	2881625	0.002049	1.33E-27	523976	lymphocyt	TRUE	reported	igd	0.000227	118.7127	2	TRUE
rs6216091	C	G	C	G	-0.02435	-0.03993	0.05783	0.033443	FALSE	TRUE	FALSE	I9_PULMEM	0.355915	0.043254	pulmonary	TRUE	reported	textfile	2	1.37E+08	0.004149	4.53E-09	523978	lymphocyt	TRUE	reported	igd	6.57E-05	34.42952	2	TRUE
rs6224121	G	A	G	A	-0.01241	0.00877	0.492468	0.455319	FALSE	FALSE	FALSE	I9_PULMEM	0.561665	0.015112	pulmonary	TRUE	reported	textfile	22	50744821	0.001982	3.88E-10	480498	lymphocyt	TRUE	reported	igd	8.16E-05	39.22964	2	TRUE
rs6233409	G	A	G	A	-0.01881	-0.01093	0.112515	0.222558	FALSE	FALSE	FALSE	I9_PULMEM	0.54523	0.018068	pulmonary	TRUE	reported	textfile	4	1.69E+08	0.002993	3.38E-10	524920	lymphocyt	TRUE	reported	igd	7.53E-05	39.50525	2	TRUE
rs6238963	G	A	G	A	0.017946	-0.03248	0.165938	0.074283	FALSE	FALSE	FALSE	I9_PULMEM	0.263991	0.029082	pulmonary	TRUE	reported	textfile	6	14734463	0.002675	2.03E-11	523982	lymphocyt	TRUE	reported	igd	8.59E-05	45.00763	2	TRUE
rs6248590	T	C	T	C	-0.0177	0.000157	0.551492	0.595775	FALSE	FALSE	FALSE	I9_PULMEM	0.991838	0.015301	pulmonary	TRUE	reported	textfile	7	1.39E+08	0.001909	1.97E-20	523981	lymphocyt	TRUE	reported	igd	0.000164	85.96725	2	TRUE
rs6262181	A	G	A	G	0.055993	0.00657	0.020688	0.042468	FALSE	FALSE	FALSE	I9_PULMEM	0.861913	0.037769	pulmonary	TRUE	reported	textfile	7	1.27E+08	0.006836	2.73E-16	524462	lymphocyt	TRUE	reported	igd	0.000128	67.09061	2	TRUE
rs631864	C	T	C	T	0.013864	0.026389	0.471706	0.479833	FALSE	FALSE	FALSE	I9_PULMEM	0.08101	0.015124	pulmonary	TRUE	reported	textfile	15	70376441	0.001958	1.48E-12	523521	lymphocyt	TRUE	reported	igd	9.58E-05	50.13604	2	TRUE
rs6445826	C	T	C	T	0.019885	0.011263	0.498133	0.556726	FALSE	FALSE	FALSE	I9_PULMEM	0.459453	0.015226	pulmonary	TRUE	reported	textfile	3	56848999	0.001931	7.68E-25	523833	lymphocyt	TRUE	reported	igd	0.000202	106.0437	2	TRUE
rs6474359	C	T	C	T	-0.05137	0.082458	0.037682	0.016943	FALSE	FALSE	FALSE	I9_PULMEM	0.154226	0.057875	pulmonary	TRUE	reported	textfile	8	41549194	0.005006	1.13E-24	523520	lymphocyt	TRUE	reported	igd	0.000201	105.3059	2	TRUE
rs6475476	G	A	G	A	0.010844	-0.03037	0.373093	0.405182	FALSE	FALSE	FALSE	I9_PULMEM	0.047239	0.015307	pulmonary	TRUE	reported	textfile	9	20802452	0.001958	3.12E-08	523980	lymphocyt	TRUE	reported	igd	5.85E-05	30.6727	2	TRUE
rs6487543	A	G	A	G	0.019723	-0.02556	0.770994	0.789244	FALSE	FALSE	FALSE	I9_PULMEM	0.168067	0.01854	pulmonary	TRUE	reported	textfile	12	26438189	0.002284	6.20E-18	523983	lymphocyt	TRUE	reported	igd	0.000142	74.56793	2	TRUE
rs6488548	A	C	A	C	0.017148	0.00579	0.598761	0.536083	FALSE	FALSE	FALSE	I9_PULMEM	0.701904	0.015129	pulmonary	TRUE	reported	textfile	12	12882508	0.001938	9.43E-19	523836	lymphocyt	TRUE	reported	igd	0.000149	78.29206	2	TRUE
rs6502555	C	T	C	T	0.028939	-0.02616	0.269464	0.258477	FALSE	FALSE	FALSE	I9_PULMEM	0.127344	0.017159	pulmonary	TRUE	reported	textfile	17	2729652	0.002252	9.26E-38	480018	lymphocyt	TRUE	reported	igd	0.000344	165.1309	2	TRUE
rs6546881	T	C	T	C	0.012654	-0.01435	0.657162	0.738621	FALSE	FALSE	FALSE	I9_PULMEM	0.403178	0.017162	pulmonary	TRUE	reported	textfile	2	74187420	0.001992	2.17E-10	523791	lymphocyt	TRUE	reported	igd	7.70E-05	40.35295	2	TRUE
rs6554158	A	G	A	G	0.014203	0.00226	0.231214	0.265237	FALSE	FALSE	FALSE	I9_PULMEM	0.894504	0.017044	pulmonary	TRUE	reported	textfile	4	55066077	0.00224	2.34E-10	524917	lymphocyt	TRUE	reported	igd	7.66E-05	40.20337	2	TRUE
rs6586777	A	C	A	C	-0.01282	-0.01546	0.437803	0.570857	FALSE	FALSE	FALSE	I9_PULMEM	0.307498	0.01515	pulmonary	TRUE	reported	textfile	8	18702471	0.001906	1.79E-11	523981	lymphocyt	TRUE	reported	igd	8.63E-05	45.24063	2	TRUE
rs6679677	A	C	A	C	-0.05384	0.00222	0.100262	0.146443	FALSE	FALSE	FALSE	I9_PULMEM	0.917043	0.02131	pulmonary	TRUE	reported	textfile	1	1.14E+08	0.00313	2.96E-66	524462	lymphocyt	TRUE	reported	igd	0.000564	295.8938	2	TRUE
rs6700896	T	C	T	C	-0.01736	0.007788	0.378957	0.463709	FALSE	FALSE	FALSE	I9_PULMEM	0.605044	0.015059	pulmonary	TRUE	reported	textfile	1	66089782	0.001939	3.67E-19	524461	lymphocyt	TRUE	reported	igd	0.000153	80.1756	2	TRUE
rs6721663	A	G	A	G	-0.02613	0.003854	0.158697	0.1854	FALSE	FALSE	FALSE	I9_PULMEM	0.841949	0.019326	pulmonary	TRUE	reported	textfile	2	7615061	0.0026	9.83E-24	524919	lymphocyt	TRUE	reported	igd	0.000192	101.0253	2	TRUE

rs6742799	C	A	C	A	-0.03924	-0.00462	0.199766	0.200727	FALSE	FALSE	FALSE	I9_PULMEM	0.805214	0.018728	pulmonary	TRUE	reported	textfile	2	1.61E+08	0.002357	3.54E-62	523521	lymphocyt	TRUE	reported	igd	0.000529	277.1501	2	TRUE
rs6743068	G	A	G	A	0.031438	0.001684	0.729732	0.676248	FALSE	FALSE	FALSE	I9_PULMEM	0.916389	0.016045	pulmonary	TRUE	reported	textfile	2	2.02E+08	0.002125	1.69E-49	523983	lymphocyt	TRUE	reported	igd	0.000418	218.872	2	TRUE
rs6748379	T	C	T	C	-0.03536	-0.04396	0.042874	0.020196	FALSE	FALSE	FALSE	I9_PULMEM	0.407443	0.053065	pulmonary	TRUE	reported	textfile	13	72503638	0.00473	8.06E-14	523982	lymphocyt	TRUE	reported	igd	0.000107	55.87936	2	TRUE
rs6751671	A	G	A	G	0.014929	0.018427	0.422528	0.311953	FALSE	FALSE	FALSE	I9_PULMEM	0.256078	0.016225	pulmonary	TRUE	reported	textfile	12	1.24E+08	0.001925	9.11E-15	524910	lymphocyt	TRUE	reported	igd	0.000115	60.14483	2	TRUE
rs6755786	T	C	T	C	0.026573	0.045476	0.773393	0.808418	FALSE	FALSE	FALSE	I9_PULMEM	0.017113	0.019074	pulmonary	TRUE	reported	textfile	2	1.03E+08	0.002252	4.20E-32	523978	lymphocyt	TRUE	reported	igd	0.000266	139.2332	2	TRUE
rs6755895	C	T	C	T	-0.02342	0.044877	0.230958	0.215181	FALSE	FALSE	FALSE	I9_PULMEM	0.0141	0.018282	pulmonary	TRUE	reported	textfile	2	2.33E+08	0.002291	1.65E-24	523976	lymphocyt	TRUE	reported	igd	0.000199	104.5281	2	TRUE
rs678393	G	T	G	T	-0.01542	0.016521	0.261711	0.219348	FALSE	FALSE	FALSE	I9_PULMEM	0.364761	0.018229	pulmonary	TRUE	reported	textfile	5	72418309	0.002196	2.32E-12	524921	lymphocyt	TRUE	reported	igd	9.39E-05	49.28072	2	TRUE
rs6808949	C	T	C	T	0.022715	0.014737	0.11098	0.074796	FALSE	FALSE	FALSE	I9_PULMEM	0.605883	0.028563	pulmonary	TRUE	reported	textfile	3	18883999	0.003046	9.16E-14	523523	lymphocyt	TRUE	reported	igd	0.000106	55.61143	2	TRUE
rs6926219	A	G	A	G	0.026814	0.000297	0.547273	0.536312	FALSE	FALSE	FALSE	I9_PULMEM	0.984254	0.015031	pulmonary	TRUE	reported	textfile	6	1.23E+08	0.001896	2.23E-45	524911	lymphocyt	TRUE	reported	igd	0.000381	200.0069	2	TRUE
rs6929523	G	A	G	A	0.017001	0.019639	0.707092	0.760715	FALSE	FALSE	FALSE	I9_PULMEM	0.264124	0.017587	pulmonary	TRUE	reported	textfile	6	35625705	0.002074	2.58E-16	524918	lymphocyt	TRUE	reported	igd	0.000128	67.19389	2	TRUE
rs6955702	G	A	G	A	-0.02228	-0.00134	0.522122	0.528943	FALSE	FALSE	FALSE	I9_PULMEM	0.929364	0.015095	pulmonary	TRUE	reported	textfile	7	3157702	0.001916	3.36E-31	523522	lymphocyt	TRUE	reported	igd	0.000258	135.1704	2	TRUE
rs696	T	C	T	C	0.025359	-0.03477	0.364762	0.388934	FALSE	FALSE	FALSE	I9_PULMEM	0.02388	0.01539	pulmonary	TRUE	reported	textfile	14	35871093	0.001966	4.94E-38	523983	lymphocyt	TRUE	reported	igd	0.000317	166.3779	2	TRUE
rs6966893	A	G	A	G	0.018708	-0.01393	0.178557	0.155335	FALSE	FALSE	FALSE	I9_PULMEM	0.501695	0.020735	pulmonary	TRUE	reported	textfile	7	37385357	0.002471	3.91E-14	524908	lymphocyt	TRUE	reported	igd	0.000109	57.32018	2	TRUE
rs7007986	A	G	A	G	-0.022	0.018343	0.601181	0.630536	FALSE	FALSE	FALSE	I9_PULMEM	0.238311	0.015555	pulmonary	TRUE	reported	textfile	8	1.42E+08	0.002012	8.50E-28	481435	lymphocyt	TRUE	reported	igd	0.000248	119.5713	2	TRUE
rs7071131	G	A	G	A	-0.01604	-0.02896	0.495115	0.433502	FALSE	FALSE	FALSE	I9_PULMEM	0.05576	0.015139	pulmonary	TRUE	reported	textfile	10	1.21E+08	0.001968	3.85E-16	480008	lymphocyt	TRUE	reported	igd	0.000138	66.38744	2	TRUE
rs707833	A	G	A	G	0.015021	-0.00676	0.636292	0.538814	FALSE	FALSE	FALSE	I9_PULMEM	0.654623	0.015112	pulmonary	TRUE	reported	textfile	6	15341375	0.001969	2.51E-14	522089	lymphocyt	TRUE	reported	igd	0.000111	58.19754	2	TRUE
rs7093481	G	A	G	A	-0.01659	0.029383	0.307178	0.296645	FALSE	FALSE	FALSE	I9_PULMEM	0.073245	0.016403	pulmonary	TRUE	reported	textfile	10	44879711	0.002053	6.84E-16	524911	lymphocyt	TRUE	reported	igd	0.000124	65.28426	2	TRUE
rs7127911	T	G	T	G	0.024096	-0.00765	0.266421	0.291982	FALSE	FALSE	FALSE	I9_PULMEM	0.644416	0.016572	pulmonary	TRUE	reported	textfile	11	1.28E+08	0.002147	3.44E-29	523512	lymphocyt	TRUE	reported	igd	0.000241	125.9575	2	TRUE
rs7131897	T	G	T	G	-0.01545	-0.02098	0.240872	0.225121	FALSE	FALSE	FALSE	I9_PULMEM	0.243941	0.018007	pulmonary	TRUE	reported	textfile	22	24658265	0.002253	7.29E-12	524913	lymphocyt	TRUE	reported	igd	8.96E-05	47.03762	2	TRUE
rs714027	G	A	G	A	-0.03491	0.017389	0.552066	0.478435	FALSE	FALSE	FALSE	I9_PULMEM	0.246624	0.015009	pulmonary	TRUE	reported	textfile	22	30577771	0.001905	5.96E-75	523522	lymphocyt	TRUE	reported	igd	0.000641	335.7442	2	TRUE
rs7142083	T	C	T	C	0.030214	0.014358	0.097097	0.071203	FALSE	FALSE	FALSE	I9_PULMEM	0.621777	0.029104	pulmonary	TRUE	reported	textfile	2	1.29E+08	0.003343	1.67E-19	480503	lymphocyt	TRUE	reported	igd	0.00017	81.68492	2	TRUE
rs7161799	T	C	T	C	0.034112	0.040475	0.076225	0.043343	FALSE	FALSE	FALSE	I9_PULMEM	0.274163	0.037014	pulmonary	TRUE	reported	textfile	15	58770523	0.0036	2.79E-21	524922	lymphocyt	TRUE	reported	igd	0.000171	89.78581	2	TRUE
rs716723	T	A	T	A	-0.017	0.036841	0.134335	0.111507	FALSE	TRUE	FALSE	I9_PULMEM	0.124008	0.023951	pulmonary	TRUE	reported	textfile	1	1.57E+08	0.002762	7.76E-10	524922	lymphocyt	TRUE	reported	igd	7.22E-05	37.88785	2	TRUE
rs7192652	G	A	G	A	0.022219	0.016422	0.480623	0.564703	FALSE	FALSE	FALSE	I9_PULMEM	0.281592	0.015251	pulmonary	TRUE	reported	textfile	16	57075180	0.001915	4.32E-31	523516	lymphocyt	TRUE	reported	igd	0.000257	134.6201	2	TRUE
rs721131	C	T	C	T	-0.0189	0.011758	0.31956	0.29142	FALSE	FALSE	FALSE	I9_PULMEM	0.476381	0.016511	pulmonary	TRUE	reported	textfile	21	47957767	0.00203	1.36E-20	524919	lymphocyt	TRUE	reported	igd	0.000165	86.70054	2	TRUE
rs721377	T	C	T	C	0.019951	0.020542	0.102169	0.16136	FALSE	FALSE	FALSE	I9_PULMEM	0.315254	0.020454	pulmonary	TRUE	reported	textfile	3	14435063	0.003209	5.23E-10	522442	lymphocyt	TRUE	reported	igd	7.40E-05	38.65345	2	TRUE
rs7249236	C	G	C	G	0.025664	-0.0119	0.801652	0.781678	FALSE	TRUE	FALSE	I9_PULMEM	0.513621	0.018213	pulmonary	TRUE	reported	textfile	19	16363417	0.002443	8.62E-26	523841	lymphocyt	TRUE	reported	igd	0.000211	110.3571	2	TRUE
rs7252565	A	G	A	G	0.027591	-0.00865	0.787553	0.72967	FALSE	FALSE	FALSE	I9_PULMEM	0.613543	0.017126	pulmonary	TRUE	reported	textfile	19	1192769	0.002536	1.55E-27	474672	lymphocyt	TRUE	reported	igd	0.000249	118.3681	2	TRUE
rs7254517	A	G	A	G	0.029033	0.003563	0.384999	0.429576	FALSE	FALSE	FALSE	I9_PULMEM	0.813875	0.015136	pulmonary	TRUE	reported	textfile	19	13929049	0.002022	1.05E-46	480501	lymphocyt	TRUE	reported	igd	0.000429	206.1673	2	TRUE
rs7263481	T	C	T	C	-0.01432	-0.01009	0.263613	0.227769	FALSE	FALSE	FALSE	I9_PULMEM	0.578266	0.018158	pulmonary	TRUE	reported	textfile	1	1596500	0.002296	4.67E-10	451140	lymphocyt	TRUE	reported	igd	8.62E-05	38.87201	2	TRUE
rs7274055	A	G	A	G	-0.01873	0.01265	0.222059	0.176508	FALSE	FALSE	FALSE	I9_PULMEM	0.520167	0.01967	pulmonary	TRUE	reported	textfile	1	1.97E+08	0.002269	1.58E-16	523983	lymphocyt	TRUE	reported	igd	0.00013	68.15499	2	TRUE
rs7283531	C	T	C	T	0.035058	0.016931	0.060241	0.104717	FALSE	FALSE	FALSE	I9_PULMEM	0.492348	0.02466	pulmonary	TRUE	reported	textfile	6	231878	0.004198	7.09E-17	521155	lymphocyt	TRUE	reported	igd	0.000134	69.74093	2	TRUE
rs7292803	A	G	A	G	-0.03332	0.00188	0.176926	0.114833	FALSE	FALSE	FALSE	I9_PULMEM	0.936375	0.023548	pulmonary	TRUE	reported	textfile	6	90976768	0.002481	4.24E-41	524918	lymphocyt	TRUE	reported	igd	0.000344	180.3877	2	TRUE
rs7300096	A	T	A	T	0.012926	-0.01657	0.315412	0.375965	FALSE	TRUE	FALSE	I9_PULMEM	0.287892	0.015591	pulmonary	TRUE	reported	textfile	11	1.14E+08	0.002044	2.63E-10	524921	lymphocyt	TRUE	reported	igd	7.62E-05	39.99124	2	TRUE
rs7308380	C	A	C	A	0.015969	-0.00373	0.750511	0.747749	FALSE	FALSE	FALSE	I9_PULMEM	0.829731	0.017332	pulmonary	TRUE	reported	textfile	12	89851232	0.00218	2.47E-13	524920	lymphocyt	TRUE	reported	igd	0.000102	53.65878	2	TRUE
rs7310578	A	G	A	G	-0.01523	0.003444	0.319436	0.216291	FALSE	FALSE	FALSE	I9_PULMEM	0.850659	0.018292	pulmonary	TRUE	reported	textfile	12	49138958	0.002036	7.76E-14	523792	lymphocyt	TRUE	reported	igd	0.000107	55.93343	2	TRUE
rs7312770	T	C	T	C	0.012056	0.020121	0.505626	0.526616	FALSE	FALSE	FALSE	I9_PULMEM	0.182183	0.015082	pulmonary	TRUE	reported	textfile	12	56467587	0.001909	2.80E-10	524916	lymphocyt	TRUE	reported	igd	7.60E-05	39.88347	2	TRUE
rs7314229	T	C	T	C	0.015158	-0.00316	0.247603	0.278304	FALSE	FALSE	FALSE	I9_PULMEM	0.850812	0.016795	pulmonary	TRUE	reported	textfile	4	40301624	0.002212	7.51E-12	523840	lymphocyt	TRUE	reported	igd	8.96E-05	46.95825	2	TRUE
rs7316551	A	G	A	G	0.020861	-0.00189	0.086367	0.187888	FALSE	FALSE	FALSE	I9_PULMEM	0.922397	0.019385	pulmonary	TRUE	reported	textfile	7	1.51E+08	0.003359	5.47E-10	524914	lymphocyt	TRUE	reported	igd	7.35E-05	38.56991	2	TRUE
rs7320761	T	G	T	G	-0.0341	0.077981	0.027199	0.010201	FALSE	FALSE	FALSE	I9_PULMEM	0.298808	0.075055	pulmonary	TRUE	reported	textfile	12	1.13E+08	0.005875	6.65E-09	523984	lymphocyt	TRUE	reported	igd	6.43E-05	33.6853	2	TRUE
rs7323928	A	G	A	G	-0.03152	0.009652	0.203256	0.274368	FALSE	FALSE	FALSE	I9_PULMEM	0.565477	0.016794	pulmonary	TRUE	reported	textfile	5	1.19E+08	0.002348	4.72E-41	524918	lymphocyt	TRUE	reported	igd	0.000343	180.2311	2	TRUE
rs7396171	C	T	C	T	-0.04895	0.010406	0.127705	0.080666	FALSE	FALSE	FALSE	I9_PULMEM	0.704583	0.027446	pulmonary	TRUE	reported	textfile	2	1.44E+08	0.002823	2.79E-67	523983	lymphocyt	TRUE	reported	igd	0.000573	300.6644	2	TRUE
rs7422770	A	G	A	G	-0.0289	0.029751	0.071527	0.05413	FALSE	FALSE	FALSE	I9_PULMEM	0.368966	0.033115	pulmonary	TRUE	reported	textfile	1	2.48E+08	0.00365	2.51E-15	524921	lymphocyt	TRUE	reported	igd	0.000119	62.70446	2	TRUE
rs7428015	T	C	T	C	0.028848	0.000555	0.174782	0.153178	FALSE	FALSE	FALSE	I9_PULMEM	0.97887	0.020966	pulmonary	TRUE	reported	textfile	3	28287735	0.002226	2.22E-38	524901	lymphocyt	TRUE	reported	igd	0.00032	167.9498	2	TRUE

rs7434461	A	G	A	G	-0.01321	0.001896	0.192087	0.193936	FALSE	FALSE	FALSE	I9_PULMEM	0.920252	0.018941	pulmonary	TRUE	reported	textfile	12	96281510	0.002399	3.79E-08	524921	lymphocyt	TRUE	reported	igd	5.77E-05	30.31182	2	TRUE
rs7440881	A	C	A	C	-0.02337	-0.05932	0.08075	0.051578	FALSE	FALSE	FALSE	I9_PULMEM	0.081018	0.033995	pulmonary	TRUE	reported	textfile	4	1.58E+08	0.003486	2.10E-11	524920	lymphocyt	TRUE	reported	igd	8.56E-05	44.93135	2	TRUE
rs744222	C	T	C	T	-0.01174	-0.01181	0.666159	0.640827	FALSE	FALSE	FALSE	I9_PULMEM	0.451901	0.015701	pulmonary	TRUE	reported	textfile	11	44603597	0.002061	1.26E-08	522900	lymphocyt	TRUE	reported	igd	6.20E-05	32.43624	2	TRUE
rs7452686	A	G	A	G	0.034829	0.004749	0.125385	0.095519	FALSE	FALSE	FALSE	I9_PULMEM	0.851848	0.025426	pulmonary	TRUE	reported	textfile	6	42024634	0.002856	3.60E-34	524922	lymphocyt	TRUE	reported	igd	0.000283	148.7181	2	TRUE
rs7467931	G	A	G	A	0.031268	-0.00303	0.056702	0.043998	FALSE	FALSE	FALSE	I9_PULMEM	0.934638	0.036929	pulmonary	TRUE	reported	textfile	11	62194434	0.004262	2.28E-13	524923	lymphocyt	TRUE	reported	igd	0.000103	53.82347	2	TRUE
rs748113	C	T	C	T	-0.03428	-0.00215	0.436258	0.586997	FALSE	FALSE	FALSE	I9_PULMEM	0.889644	0.015477	pulmonary	TRUE	reported	textfile	10	73508791	0.002004	1.53E-65	479423	lymphocyt	TRUE	reported	igd	0.00061	292.6068	2	TRUE
rs7491126	A	G	A	G	0.044672	-0.03525	0.025691	0.008037	FALSE	FALSE	FALSE	I9_PULMEM	0.675409	0.084178	pulmonary	TRUE	reported	textfile	11	1.08E+08	0.005984	8.68E-14	524066	lymphocyt	TRUE	reported	igd	0.000106	55.72961	2	TRUE
rs7501849	G	C	G	C	0.024538	-0.04913	0.075981	0.060012	FALSE	TRUE	FALSE	I9_PULMEM	0.118342	0.031458	pulmonary	TRUE	reported	textfile	19	40785683	0.003639	1.60E-11	524906	lymphocyt	TRUE	reported	igd	8.66E-05	45.4687	2	TRUE
rs7504921	T	C	T	C	-0.03071	0.030807	0.039779	0.025919	FALSE	FALSE	FALSE	I9_PULMEM	0.51076	0.046844	pulmonary	TRUE	reported	textfile	7	1.16E+08	0.004887	3.42E-10	523066	lymphocyt	TRUE	reported	igd	7.55E-05	39.47588	2	TRUE
rs7529794	T	G	T	G	0.016098	0.025482	0.26241	0.238527	FALSE	FALSE	FALSE	I9_PULMEM	0.149083	0.017662	pulmonary	TRUE	reported	textfile	1	40403370	0.002151	7.57E-14	523842	lymphocyt	TRUE	reported	igd	0.000107	56.00946	2	TRUE
rs7548606	G	A	G	A	0.016235	0.006023	0.625851	0.634788	FALSE	FALSE	FALSE	I9_PULMEM	0.700058	0.015636	pulmonary	TRUE	reported	textfile	1	2.12E+08	0.001951	9.26E-17	524922	lymphocyt	TRUE	reported	igd	0.000132	69.245	2	TRUE
rs7562883	A	G	A	G	0.030433	0.061555	0.085709	0.082497	FALSE	FALSE	FALSE	I9_PULMEM	0.024865	0.027437	pulmonary	TRUE	reported	textfile	6	22316185	0.003456	1.38E-18	524919	lymphocyt	TRUE	reported	igd	0.000148	77.54261	2	TRUE
rs7565358	T	C	T	C	0.061126	-0.563	0.012672	0.003001	FALSE	FALSE	FALSE	I9_PULMEM	0.00078	0.167564	pulmonary	TRUE	reported	textfile	8	78363128	0.00886	5.45E-12	521981	lymphocyt	TRUE	reported	igd	9.12E-05	47.59736	2	TRUE
rs7572278	A	T	A	T	0.020437	0.009696	0.206818	0.219939	FALSE	TRUE	FALSE	I9_PULMEM	0.595552	0.018266	pulmonary	TRUE	reported	textfile	2	8563029	0.002383	1.04E-17	523841	lymphocyt	TRUE	reported	igd	0.00014	73.55032	2	TRUE
rs7596846	T	G	T	G	-0.02122	-0.0475	0.920631	0.932434	FALSE	FALSE	FALSE	I9_PULMEM	0.112772	0.029952	pulmonary	TRUE	reported	textfile	2	2.2E+08	0.003547	2.27E-09	523984	lymphocyt	TRUE	reported	igd	6.83E-05	35.79041	2	TRUE
rs760908	T	G	T	G	-0.0152	0.02954	0.15316	0.145257	FALSE	FALSE	FALSE	I9_PULMEM	0.164647	0.021258	pulmonary	TRUE	reported	textfile	22	32057501	0.002649	9.70E-09	523522	lymphocyt	TRUE	reported	igd	6.29E-05	32.94201	2	TRUE
rs7642810	C	T	C	T	0.062814	-0.10983	0.013444	0.012756	FALSE	FALSE	FALSE	I9_PULMEM	0.108723	0.068471	pulmonary	TRUE	reported	textfile	13	28604007	0.008702	5.49E-13	524921	lymphocyt	TRUE	reported	igd	9.93E-05	52.10424	2	TRUE
rs7661349	C	T	C	T	0.02523	-0.01323	0.63683	0.685183	FALSE	FALSE	FALSE	I9_PULMEM	0.412543	0.016148	pulmonary	TRUE	reported	textfile	4	1.06E+08	0.001961	7.87E-38	524918	lymphocyt	TRUE	reported	igd	0.000315	165.5304	2	TRUE
rs766228	T	G	T	G	-0.01051	-0.01602	0.502115	0.516095	FALSE	FALSE	FALSE	I9_PULMEM	0.285835	0.015008	pulmonary	TRUE	reported	textfile	20	11371883	0.001903	3.37E-08	523522	lymphocyt	TRUE	reported	igd	5.83E-05	30.51927	2	TRUE
rs7672879	A	T	A	T	0.013474	-0.00024	0.352792	0.403152	FALSE	TRUE	FALSE	I9_PULMEM	0.987554	0.015307	pulmonary	TRUE	reported	textfile	4	1.23E+08	0.001981	1.08E-11	524919	lymphocyt	TRUE	reported	igd	8.81E-05	46.2618	2	TRUE
rs7683096	A	C	A	C	-0.02078	0.022544	0.116683	0.083445	FALSE	FALSE	FALSE	I9_PULMEM	0.40691	0.027183	pulmonary	TRUE	reported	textfile	3	1.6E+08	0.002956	2.13E-12	523843	lymphocyt	TRUE	reported	igd	9.44E-05	49.43175	2	TRUE
rs7696969	G	T	G	T	-0.01247	0.000373	0.619889	0.723103	FALSE	FALSE	FALSE	I9_PULMEM	0.982205	0.016718	pulmonary	TRUE	reported	textfile	4	1.43E+08	0.002023	7.36E-10	481440	lymphocyt	TRUE	reported	igd	7.89E-05	37.97176	2	TRUE
rs7705730	A	G	A	G	0.02818	-0.01365	0.040114	0.062543	FALSE	FALSE	FALSE	I9_PULMEM	0.661608	0.03119	pulmonary	TRUE	reported	textfile	3	71637104	0.005024	2.08E-08	524922	lymphocyt	TRUE	reported	igd	5.99E-05	31.46162	2	TRUE
rs7710156	A	G	A	G	0.031592	0.021062	0.036931	0.041308	FALSE	FALSE	FALSE	I9_PULMEM	0.57877	0.037937	pulmonary	TRUE	reported	textfile	3	71358223	0.005074	4.94E-10	524922	lymphocyt	TRUE	reported	igd	7.38E-05	38.76606	2	TRUE
rs7726538	T	C	T	C	-0.03317	-0.01395	0.045014	0.053573	FALSE	FALSE	FALSE	I9_PULMEM	0.678104	0.03362	pulmonary	TRUE	reported	textfile	3	1.86E+08	0.004662	1.17E-12	524921	lymphocyt	TRUE	reported	igd	9.64E-05	50.61958	2	TRUE
rs7757316	G	A	G	A	-0.04939	-0.04219	0.078773	0.06298	FALSE	FALSE	FALSE	I9_PULMEM	0.173869	0.031022	pulmonary	TRUE	reported	textfile	6	35305537	0.003516	8.70E-45	523065	lymphocyt	TRUE	reported	igd	0.000377	197.3234	2	TRUE
rs7776054	G	A	G	A	-0.04014	0.030199	0.260832	0.339957	FALSE	FALSE	FALSE	I9_PULMEM	0.057187	0.015878	pulmonary	TRUE	reported	textfile	6	1.35E+08	0.00215	9.81E-78	524458	lymphocyt	TRUE	reported	igd	0.000664	348.5589	2	TRUE
rs7780328	G	A	G	A	0.037325	0.044841	0.06709	0.072852	FALSE	FALSE	FALSE	I9_PULMEM	0.119034	0.028765	pulmonary	TRUE	reported	textfile	7	99256844	0.003773	4.75E-23	524914	lymphocyt	TRUE	reported	igd	0.000186	97.86432	2	TRUE
rs7787622	T	C	T	C	0.044726	-0.06576	0.019648	0.005506	FALSE	FALSE	FALSE	I9_PULMEM	0.511678	0.100212	pulmonary	TRUE	reported	textfile	11	67133248	0.007065	2.53E-10	524526	lymphocyt	TRUE	reported	igd	7.64E-05	40.0769	2	TRUE
rs7790229	A	G	A	G	0.012114	0.011432	0.492994	0.464818	FALSE	FALSE	FALSE	I9_PULMEM	0.44752	0.015051	pulmonary	TRUE	reported	textfile	7	97919338	0.001896	1.71E-10	524918	lymphocyt	TRUE	reported	igd	7.78E-05	40.82224	2	TRUE
rs7795444	G	A	G	A	-0.03677	0.008932	0.028289	0.028523	FALSE	FALSE	FALSE	I9_PULMEM	0.843849	0.045349	pulmonary	TRUE	reported	textfile	1	10242292	0.005678	9.78E-11	524921	lymphocyt	TRUE	reported	igd	7.99E-05	41.93447	2	TRUE
rs7805819	A	G	A	G	0.047272	0.021052	0.051732	0.082817	FALSE	FALSE	FALSE	I9_PULMEM	0.441625	0.02736	pulmonary	TRUE	reported	textfile	2	2.2E+08	0.00483	1.36E-22	516732	lymphocyt	TRUE	reported	igd	0.000185	95.7882	2	TRUE
rs780669	T	C	T	C	-0.02226	-0.01266	0.887133	0.838936	FALSE	FALSE	FALSE	I9_PULMEM	0.537678	0.020537	pulmonary	TRUE	reported	textfile	10	73112241	0.003089	5.95E-13	524923	lymphocyt	TRUE	reported	igd	9.89E-05	51.92935	2	TRUE
rs7827009	A	G	A	G	-0.03447	0.022019	0.070435	0.056431	FALSE	FALSE	FALSE	I9_PULMEM	0.500207	0.032661	pulmonary	TRUE	reported	textfile	5	1.11E+08	0.003887	7.79E-19	524922	lymphocyt	TRUE	reported	igd	0.00015	78.64607	2	TRUE
rs7839489	A	T	A	T	-0.02558	-0.06164	0.046663	0.058121	FALSE	TRUE	FALSE	I9_PULMEM	0.054776	0.032094	pulmonary	TRUE	reported	textfile	2	1.53E+08	0.004479	1.14E-08	523984	lymphocyt	TRUE	reported	igd	6.23E-05	32.6267	2	TRUE
rs7839516	C	T	C	T	0.015461	-0.0061	0.369365	0.405161	FALSE	FALSE	FALSE	I9_PULMEM	0.690098	0.015293	pulmonary	TRUE	reported	textfile	8	61386533	0.001959	3.08E-15	523984	lymphocyt	TRUE	reported	igd	0.000119	62.28804	2	TRUE
rs7848793	G	A	G	A	-0.02365	0.00849	0.077712	0.090332	FALSE	FALSE	FALSE	I9_PULMEM	0.746485	0.026263	pulmonary	TRUE	reported	textfile	16	3651579	0.00356	3.20E-11	523982	lymphocyt	TRUE	reported	igd	8.42E-05	44.12525	2	TRUE
rs7855586	G	A	G	A	0.014877	-0.01249	0.290115	0.316431	FALSE	FALSE	FALSE	I9_PULMEM	0.438652	0.016126	pulmonary	TRUE	reported	textfile	9	1.13E+08	0.002086	1.03E-12	523978	lymphocyt	TRUE	reported	igd	9.71E-05	50.86282	2	TRUE
rs7893800	A	G	A	G	0.010749	0.019057	0.389841	0.465746	FALSE	FALSE	FALSE	I9_PULMEM	0.205741	0.01506	pulmonary	TRUE	reported	textfile	10	27125289	0.001946	3.43E-08	524912	lymphocyt	TRUE	reported	igd	5.81E-05	30.51046	2	TRUE
rs7897695	A	G	A	G	0.032841	0.004485	0.041092	0.036621	FALSE	FALSE	FALSE	I9_PULMEM	0.91009	0.039714	pulmonary	TRUE	reported	textfile	7	1E+08	0.004804	8.41E-12	524917	lymphocyt	TRUE	reported	igd	8.90E-05	46.73315	2	TRUE
rs7898307	A	G	A	G	-0.06237	-0.0247	0.009511	0.004976	FALSE	FALSE	FALSE	I9_PULMEM	0.818087	0.107366	pulmonary	TRUE	reported	textfile	12	1.12E+08	0.009806	2.07E-10	523126	lymphocyt	TRUE	reported	igd	7.73E-05	40.45962	2	TRUE
rs7923752	T	C	T	C	0.08538	0.118862	0.022839	0.020131	FALSE	FALSE	FALSE	I9_PULMEM	0.026917	0.053718	pulmonary	TRUE	reported	textfile	5	1.35E+08	0.006437	4.14E-40	522125	lymphocyt	TRUE	reported	igd	0.000337	175.9315	2	TRUE
rs7924213	A	G	A	G	-0.01332	0.005727	0.307157	0.233793	FALSE	FALSE	FALSE	I9_PULMEM	0.747438	0.017784	pulmonary	TRUE	reported	textfile	10	14974563	0.002066	1.18E-10	524909	lymphocyt	TRUE	reported	igd	7.92E-05	41.57924	2	TRUE
rs79503	G	A	G	A	-0.02789	0.01803	0.447419	0.467649	FALSE	FALSE	FALSE	I9_PULMEM	0.230337	0.015031	pulmonary	TRUE	reported	textfile	22	39528440	0.001942	1.02E-46	523523	lymphocyt	TRUE	reported	igd	0.000394	206.2218	2	TRUE

rs7980539	C	T	C	T	0.017037	-0.02095	0.173015	0.160931	FALSE	FALSE	FALSE	I9_PULMEN	0.307416	0.020531	pulmonary	TRUE	reported	textfile	11	1.28E+08	0.002503	1.04E-11	524917	lymphocyt	TRUE	reported	igd	8.83E-05	46.33006	2	TRUE
rs7983682	T	C	T	C	0.013727	-0.00023	0.322752	0.349532	FALSE	FALSE	FALSE	I9_PULMEN	0.988484	0.015747	pulmonary	TRUE	reported	textfile	13	49178830	0.002028	1.34E-11	524921	lymphocyt	TRUE	reported	igd	8.73E-05	45.81564	2	TRUE
rs8000638	G	A	G	A	-0.03172	0.033051	0.031539	0.018493	FALSE	FALSE	FALSE	I9_PULMEN	0.550948	0.055424	pulmonary	TRUE	reported	textfile	7	40437273	0.005464	6.59E-09	524923	lymphocyt	TRUE	reported	igd	6.42E-05	33.70529	2	TRUE
rs8004780	C	A	C	A	0.018324	-0.04233	0.366436	0.370208	FALSE	FALSE	FALSE	I9_PULMEN	0.006646	0.015596	pulmonary	TRUE	reported	textfile	14	1.04E+08	0.001972	1.60E-20	524919	lymphocyt	TRUE	reported	igd	0.000164	86.34259	2	TRUE
rs8017724	G	A	G	A	-0.01435	-0.00963	0.167793	0.160619	FALSE	FALSE	FALSE	I9_PULMEN	0.638139	0.020485	pulmonary	TRUE	reported	textfile	10	5296243	0.002545	1.76E-08	524920	lymphocyt	TRUE	reported	igd	6.06E-05	31.79264	2	TRUE
rs8046391	C	G	C	G	-0.02447	0.024491	0.273776	0.336178	FALSE	TRUE	FALSE	I9_PULMEN	0.123849	0.015915	pulmonary	TRUE	reported	textfile	16	30836648	0.002122	9.68E-31	524918	lymphocyt	TRUE	reported	igd	0.000253	132.9984	2	TRUE
rs8052370	T	C	T	C	-0.02103	0.002672	0.609467	0.587282	FALSE	FALSE	FALSE	I9_PULMEN	0.861683	0.015334	pulmonary	TRUE	reported	textfile	16	88837298	0.002036	5.82E-25	481437	lymphocyt	TRUE	reported	igd	0.000221	106.6589	2	TRUE
rs8075090	C	T	C	T	0.020988	0.001695	0.523681	0.616933	FALSE	FALSE	FALSE	I9_PULMEN	0.912481	0.015418	pulmonary	TRUE	reported	textfile	17	4969108	0.001897	2.01E-28	524918	lymphocyt	TRUE	reported	igd	0.000233	122.4069	2	TRUE
rs8096327	G	A	G	A	-0.01264	-0.00522	0.391461	0.347911	FALSE	FALSE	FALSE	I9_PULMEN	0.740745	0.015767	pulmonary	TRUE	reported	textfile	18	12887750	0.001974	1.59E-10	520920	lymphocyt	TRUE	reported	igd	7.87E-05	40.98189	2	TRUE
rs8181326	A	G	A	G	0.012303	-0.03164	0.659461	0.639643	FALSE	FALSE	FALSE	I9_PULMEN	0.043217	0.015653	pulmonary	TRUE	reported	textfile	10	98998195	0.001994	6.99E-10	524921	lymphocyt	TRUE	reported	igd	7.25E-05	38.06888	2	TRUE
rs9328393	A	C	A	C	0.0234	-0.00907	0.668424	0.630215	FALSE	FALSE	FALSE	I9_PULMEN	0.563001	0.015689	pulmonary	TRUE	reported	textfile	6	6902939	0.002021	5.74E-31	522902	lymphocyt	TRUE	reported	igd	0.000256	134.0594	2	TRUE
rs937283	G	A	G	A	0.010584	0.000591	0.405224	0.415049	FALSE	FALSE	FALSE	I9_PULMEN	0.969006	0.015211	pulmonary	TRUE	reported	textfile	12	69202164	0.001922	3.72E-08	523977	lymphocyt	TRUE	reported	igd	5.79E-05	30.32433	2	TRUE
rs9375486	T	C	T	C	-0.01381	########	0.379599	0.379966	FALSE	FALSE	FALSE	I9_PULMEN	0.996949	0.01546	pulmonary	TRUE	reported	textfile	6	1.27E+08	0.001948	1.40E-12	524921	lymphocyt	TRUE	reported	igd	9.57E-05	50.24374	2	TRUE
rs949349	C	T	C	T	0.014189	0.007554	0.271122	0.227949	FALSE	FALSE	FALSE	I9_PULMEN	0.672589	0.017875	pulmonary	TRUE	reported	textfile	11	86297189	0.002137	3.26E-11	523523	lymphocyt	TRUE	reported	igd	8.42E-05	44.08521	2	TRUE
rs9525619	T	C	T	C	-0.02029	-0.03106	0.534266	0.615573	FALSE	FALSE	FALSE	I9_PULMEN	0.043894	0.015415	pulmonary	TRUE	reported	textfile	13	42992135	0.001896	1.08E-26	524920	lymphocyt	TRUE	reported	igd	0.000218	114.5663	2	TRUE
rs9532679	C	A	C	A	-0.03234	0.018972	0.148984	0.175873	FALSE	FALSE	FALSE	I9_PULMEN	0.338886	0.019837	pulmonary	TRUE	reported	textfile	13	41522338	0.002704	6.33E-33	524921	lymphocyt	TRUE	reported	igd	0.000272	143.0071	2	TRUE
rs9534338	C	T	C	T	0.015145	0.003438	0.459888	0.594044	FALSE	FALSE	FALSE	I9_PULMEN	0.821981	0.01528	pulmonary	TRUE	reported	textfile	13	46707137	0.001895	1.38E-15	524920	lymphocyt	TRUE	reported	igd	0.000122	63.87317	2	TRUE
rs9592965	C	A	C	A	-0.01445	0.007575	0.326994	0.224422	FALSE	FALSE	FALSE	I9_PULMEN	0.674426	0.018032	pulmonary	TRUE	reported	textfile	13	74615925	0.00202	8.83E-13	523517	lymphocyt	TRUE	reported	igd	9.77E-05	51.15771	2	TRUE
rs9605047	T	G	T	G	0.019972	0.0084	0.327768	0.373799	FALSE	FALSE	FALSE	I9_PULMEN	0.589518	0.015569	pulmonary	TRUE	reported	textfile	22	20002848	0.002109	2.99E-21	481442	lymphocyt	TRUE	reported	igd	0.000186	89.67846	2	TRUE
rs9662343	C	G	C	G	-0.01499	0.021818	0.737197	0.771325	FALSE	TRUE	FALSE	I9_PULMEN	0.224248	0.017953	pulmonary	TRUE	reported	textfile	1	23574051	0.002166	4.61E-12	524920	lymphocyt	TRUE	reported	igd	9.13E-05	47.91357	2	TRUE
rs968478	G	A	G	A	0.014727	-0.01462	0.397325	0.338114	FALSE	FALSE	FALSE	I9_PULMEN	0.356472	0.015859	pulmonary	TRUE	reported	textfile	20	47339742	0.001938	3.11E-14	524918	lymphocyt	TRUE	reported	igd	0.00011	57.74566	2	TRUE
rs9835571	G	T	G	T	0.022014	0.014253	0.689834	0.6918	FALSE	FALSE	FALSE	I9_PULMEN	0.3799	0.016232	pulmonary	TRUE	reported	textfile	3	1.37E+08	0.002037	3.49E-27	524919	lymphocyt	TRUE	reported	igd	0.000222	116.7923	2	TRUE
rs9864087	C	T	C	T	0.013996	-0.00357	0.575539	0.532215	FALSE	FALSE	FALSE	I9_PULMEN	0.812389	0.015047	pulmonary	TRUE	reported	textfile	3	1.53E+08	0.001909	2.40E-13	524917	lymphocyt	TRUE	reported	igd	0.000102	53.75197	2	TRUE
rs9864216	A	G	A	G	0.023706	-0.02294	0.776354	0.775213	FALSE	FALSE	FALSE	I9_PULMEN	0.201471	0.017955	pulmonary	TRUE	reported	textfile	3	1.51E+08	0.002268	1.57E-25	524921	lymphocyt	TRUE	reported	igd	0.000208	109.2519	2	TRUE
rs9898958	G	T	G	T	0.015551	0.032218	0.153458	0.232338	FALSE	FALSE	FALSE	I9_PULMEN	0.07028	0.017799	pulmonary	TRUE	reported	textfile	17	55242242	0.002632	3.56E-09	523520	lymphocyt	TRUE	reported	igd	6.67E-05	34.90947	2	TRUE
rs9906320	A	G	A	G	0.024622	-0.00815	0.775113	0.736006	FALSE	FALSE	FALSE	I9_PULMEN	0.631926	0.017019	pulmonary	TRUE	reported	textfile	17	72690829	0.002296	8.67E-27	522443	lymphocyt	TRUE	reported	igd	0.00022	115.0009	2	TRUE
rs9920	C	T	C	T	0.02939	-0.01877	0.104682	0.075265	FALSE	FALSE	FALSE	I9_PULMEN	0.509462	0.028456	pulmonary	TRUE	reported	textfile	7	1.16E+08	0.003092	2.15E-21	523521	lymphocyt	TRUE	reported	igd	0.000173	90.348	2	TRUE
rs9937837	G	T	G	T	-0.01509	0.001581	0.269678	0.254856	FALSE	FALSE	FALSE	I9_PULMEN	0.926779	0.017207	pulmonary	TRUE	reported	textfile	16	31298939	0.002136	1.69E-12	523983	lymphocyt	TRUE	reported	igd	9.52E-05	49.90186	2	TRUE
rs9939124	T	C	T	C	-0.01673	0.013369	0.306976	0.315985	FALSE	FALSE	FALSE	I9_PULMEN	0.407064	0.016125	pulmonary	TRUE	reported	textfile	16	75185764	0.002051	3.60E-16	523520	lymphocyt	TRUE	reported	igd	0.000127	66.56816	2	TRUE

Supplementary Table 5: Description of SNPs for monocyte cell counts incorporated after MR-presso deletion

SNP	effect_al	other_all	effect_al	other_all	beta.expo	beta.outc	eaf.expos	eaf.outc	remove	palindrom	ambiguous	id.outcom	pval.outc	se.outcom	outcome	mr_keep.	pval_orig	data_sour	chr.expos	pos.expos	se.expos	pval.expo	samplesiz	exposure	mr_keep.e	pval_orig	data_sour	R2	F	action	mr_keep
rs1002774	A	G	A	G	-0.05399	0.044909	0.111651	0.075536	FALSE	FALSE	FALSE	I9_PULMEM	0.11454	0.028457	pulmonary	TRUE	reported	textfile	15	42261781	0.002985	4.48E-73	521592	monocyte	TRUE	reported	igd	0.000627	327.1662	2	TRUE
rs1009403	G	A	G	A	-0.09129	0.016485	0.616642	0.620434	FALSE	FALSE	FALSE	I9_PULMEM	0.286171	0.015457	pulmonary	TRUE	reported	textfile	8	1.31E+08	0.001924	########	521594	monocyte	TRUE	reported	igd	0.004297	2251.109	2	TRUE
rs1019780	C	T	C	T	-0.04157	0.020873	0.15658	0.136618	FALSE	FALSE	FALSE	I9_PULMEM	0.339546	0.021855	pulmonary	TRUE	reported	textfile	2	2.26E+08	0.002564	4.70E-59	520192	monocyte	TRUE	reported	igd	0.000505	262.884	2	TRUE
rs1023843	T	C	T	C	-0.02357	-0.0154	0.239724	0.266506	FALSE	FALSE	FALSE	I9_PULMEM	0.364821	0.016991	pulmonary	TRUE	reported	textfile	7	28724491	0.002276	4.23E-25	521583	monocyte	TRUE	reported	igd	0.000206	107.2349	2	TRUE
rs1031091	G	A	G	A	-0.01093	-0.00105	0.336191	0.281242	FALSE	FALSE	FALSE	I9_PULMEM	0.950073	0.016721	pulmonary	TRUE	reported	textfile	2	24680948	0.001989	3.93E-08	519575	monocyte	TRUE	reported	igd	5.81E-05	30.21395	2	TRUE
rs1040390	G	A	G	A	0.018742	0.002373	0.724302	0.735334	FALSE	FALSE	FALSE	I9_PULMEM	0.889402	0.017067	pulmonary	TRUE	reported	textfile	19	1008358	0.002204	1.95E-17	469584	monocyte	TRUE	reported	igd	0.000154	72.31141	2	TRUE
rs1040975	G	T	G	T	0.013022	0.001788	0.794453	0.80877	FALSE	FALSE	FALSE	I9_PULMEM	0.925805	0.019201	pulmonary	TRUE	reported	textfile	19	6297374	0.002383	4.74E-08	521589	monocyte	TRUE	reported	igd	5.72E-05	29.86109	2	TRUE
rs1042070	C	T	C	T	0.018408	0.046288	0.808132	0.845877	FALSE	FALSE	FALSE	I9_PULMEM	0.026272	0.02083	pulmonary	TRUE	reported	textfile	19	33758488	0.00249	1.49E-13	476099	monocyte	TRUE	reported	igd	0.000115	54.65283	2	TRUE
rs1043795	A	G	A	G	-0.01788	-0.00202	0.905195	0.893562	FALSE	FALSE	FALSE	I9_PULMEM	0.934058	0.024467	pulmonary	TRUE	reported	textfile	12	58003922	0.003268	4.59E-08	521589	monocyte	TRUE	reported	igd	5.74E-05	29.93431	2	TRUE
rs1046015	C	T	C	T	-0.01579	-0.0089	0.428951	0.462808	FALSE	FALSE	FALSE	I9_PULMEM	0.55434	0.015055	pulmonary	TRUE	reported	textfile	18	60996360	0.001899	9.88E-17	520192	monocyte	TRUE	reported	igd	0.000133	69.10232	2	TRUE
rs1047805	G	A	G	A	0.014834	-0.00711	0.196989	0.211913	FALSE	FALSE	FALSE	I9_PULMEM	0.698845	0.01838	pulmonary	TRUE	reported	textfile	5	1.11E+08	0.002354	3.01E-10	521591	monocyte	TRUE	reported	igd	7.61E-05	39.71019	2	TRUE
rs1051832	G	A	G	A	-0.01373	0.012617	0.330665	0.309873	FALSE	FALSE	FALSE	I9_PULMEM	0.436901	0.01623	pulmonary	TRUE	reported	textfile	4	1.2E+08	0.001995	6.18E-12	520193	monocyte	TRUE	reported	igd	9.10E-05	47.35767	2	TRUE
rs1052484	C	G	C	G	0.013854	0.000805	0.215829	0.17081	FALSE	TRUE	FALSE	I9_PULMEM	0.967699	0.019867	pulmonary	TRUE	reported	textfile	14	25281444	0.002281	1.28E-09	521590	monocyte	TRUE	reported	igd	7.07E-05	36.88911	2	TRUE
rs1078020	A	G	A	G	0.045431	-0.00742	0.479354	0.547063	FALSE	FALSE	FALSE	I9_PULMEM	0.622997	0.015095	pulmonary	TRUE	reported	textfile	9	91472127	0.001865	########	521582	monocyte	TRUE	reported	igd	0.001136	593.3969	2	TRUE
rs1079682	G	T	G	T	-0.01797	0.007751	0.635004	0.743213	FALSE	FALSE	FALSE	I9_PULMEM	0.651849	0.017179	pulmonary	TRUE	reported	textfile	11	69490346	0.00195	3.26E-20	520511	monocyte	TRUE	reported	igd	0.000163	84.95134	2	TRUE
rs1081419	C	A	C	A	0.014748	0.003861	0.715571	0.611537	FALSE	FALSE	FALSE	I9_PULMEM	0.802213	0.015413	pulmonary	TRUE	reported	textfile	9	35018686	0.002069	1.07E-12	521594	monocyte	TRUE	reported	igd	9.74E-05	50.80935	2	TRUE
rs1082872	T	G	T	G	-0.04727	0.016362	0.367802	0.384907	FALSE	FALSE	FALSE	I9_PULMEM	0.289204	0.015437	pulmonary	TRUE	reported	textfile	10	25218243	0.002019	########	478116	monocyte	TRUE	reported	igd	0.001145	548.0307	2	TRUE
rs1083150	C	A	C	A	0.010798	-0.01994	0.38974	0.383702	FALSE	FALSE	FALSE	I9_PULMEM	0.193853	0.015349	pulmonary	TRUE	reported	textfile	11	96060068	0.001927	2.15E-08	521590	monocyte	TRUE	reported	igd	6.02E-05	31.39941	2	TRUE
rs1084902	G	C	G	C	-0.02407	0.001986	0.210195	0.212713	FALSE	TRUE	FALSE	I9_PULMEM	0.913868	0.018359	pulmonary	TRUE	reported	textfile	12	4332009	0.002299	1.28E-25	518763	monocyte	TRUE	reported	igd	0.000211	109.5792	2	TRUE
rs1084944	G	A	G	A	-0.05014	0.020063	0.753266	0.756249	FALSE	FALSE	FALSE	I9_PULMEM	0.254878	0.017621	pulmonary	TRUE	reported	textfile	12	6493351	0.002236	########	521594	monocyte	TRUE	reported	igd	0.000963	502.9128	2	TRUE
rs1089234	C	T	C	T	-0.01811	0.023223	0.276119	0.298845	FALSE	FALSE	FALSE	I9_PULMEM	0.155668	0.016357	pulmonary	TRUE	reported	textfile	11	1.19E+08	0.002101	7.02E-18	521589	monocyte	TRUE	reported	igd	0.000142	74.29082	2	TRUE
rs1091815	A	G	A	G	-0.01425	-0.02227	0.76516	0.764008	FALSE	FALSE	FALSE	I9_PULMEM	0.207505	0.017668	pulmonary	TRUE	reported	textfile	7	73489643	0.002263	3.14E-10	519576	monocyte	TRUE	reported	igd	7.63E-05	39.65144	2	TRUE
rs1093547	T	G	T	G	0.025021	0.022191	0.441522	0.444747	FALSE	FALSE	FALSE	I9_PULMEM	0.141209	0.015083	pulmonary	TRUE	reported	textfile	3	98416900	0.00188	2.30E-40	520192	monocyte	TRUE	reported	igd	0.00034	177.1299	2	TRUE
rs1094831	G	A	G	A	-0.03358	-0.01554	0.10293	0.132363	FALSE	FALSE	FALSE	I9_PULMEM	0.484085	0.022206	pulmonary	TRUE	reported	textfile	6	46911131	0.003076	1.04E-27	520190	monocyte	TRUE	reported	igd	0.000229	119.1684	2	TRUE
rs1105784	T	C	T	C	0.020359	0.020768	0.143569	0.140369	FALSE	FALSE	FALSE	I9_PULMEM	0.339848	0.021759	pulmonary	TRUE	reported	textfile	12	1.25E+08	0.002724	8.16E-14	521592	monocyte	TRUE	reported	igd	0.000107	55.8595	2	TRUE
rs1108239	G	T	G	T	0.018719	-0.0125	0.128716	0.126866	FALSE	FALSE	FALSE	I9_PULMEM	0.579306	0.022543	pulmonary	TRUE	reported	textfile	18	42094299	0.002766	1.36E-11	521591	monocyte	TRUE	reported	igd	8.78E-05	45.79935	2	TRUE
rs1108610	C	G	C	G	0.031397	0.017014	0.632941	0.665326	FALSE	TRUE	FALSE	I9_PULMEM	0.286313	0.015957	pulmonary	TRUE	reported	textfile	19	18398628	0.002021	2.26E-54	478115	monocyte	TRUE	reported	igd	0.000505	241.347	2	TRUE
rs1110488	C	T	C	T	-0.01557	-0.03933	0.702076	0.596551	FALSE	FALSE	FALSE	I9_PULMEM	0.010327	0.015333	pulmonary	TRUE	reported	textfile	12	88843474	0.002051	3.35E-14	520653	monocyte	TRUE	reported	igd	0.000111	57.61461	2	TRUE
rs1113061	T	C	T	C	-0.01237	0.02115	0.334436	0.297581	FALSE	FALSE	FALSE	I9_PULMEM	0.19808	0.016433	pulmonary	TRUE	reported	textfile	3	58029016	0.001985	4.71E-10	521568	monocyte	TRUE	reported	igd	7.45E-05	38.85325	2	TRUE
rs1114565	A	G	A	G	-0.01432	-0.05145	0.163516	0.111871	FALSE	FALSE	FALSE	I9_PULMEM	0.029876	0.023689	pulmonary	TRUE	reported	textfile	10	1.26E+08	0.002538	1.70E-08	521571	monocyte	TRUE	reported	igd	6.11E-05	31.85251	2	TRUE
rs1115569	G	C	G	C	-0.0358	0.079643	0.02697	0.004739	FALSE	TRUE	FALSE	I9_PULMEM	0.466287	0.10932	pulmonary	TRUE	reported	textfile	3	42945097	0.005896	1.30E-09	521194	monocyte	TRUE	reported	igd	7.07E-05	36.87211	2	TRUE
rs1115578	T	C	T	C	0.013538	-0.00043	0.637971	0.66756	FALSE	FALSE	FALSE	I9_PULMEM	0.978306	0.015954	pulmonary	TRUE	reported	textfile	6	1.52E+08	0.001949	3.92E-12	520194	monocyte	TRUE	reported	igd	9.27E-05	48.24848	2	TRUE
rs1117065	A	G	A	G	-0.01808	-0.03122	0.194716	0.239709	FALSE	FALSE	FALSE	I9_PULMEM	0.077174	0.017667	pulmonary	TRUE	reported	textfile	12	54099076	0.002374	2.77E-14	521553	monocyte	TRUE	reported	igd	0.000111	57.96869	2	TRUE
rs1118915	A	G	A	G	0.050518	-0.03105	0.255718	0.284386	FALSE	FALSE	FALSE	I9_PULMEM	0.061852	0.016628	pulmonary	TRUE	reported	textfile	10	99108922	0.002145	########	520154	monocyte	TRUE	reported	igd	0.001065	554.6717	2	TRUE
rs1119014	T	C	T	C	-0.04368	0.002093	0.371953	0.458805	FALSE	FALSE	FALSE	I9_PULMEM	0.889386	0.015046	pulmonary	TRUE	reported	textfile	10	1.01E+08	0.001932	########	521588	monocyte	TRUE	reported	igd	0.000979	511.1512	2	TRUE
rs1122482	C	A	C	A	0.0221	0.011622	0.128726	0.074058	FALSE	FALSE	FALSE	I9_PULMEM	0.685572	0.028706	pulmonary	TRUE	reported	textfile	7	44770213	0.00282	4.83E-15	521560	monocyte	TRUE	reported	igd	0.000118	61.41644	2	TRUE
rs1123568	T	C	T	C	-0.01609	-0.0124	0.413912	0.419447	FALSE	FALSE	FALSE	I9_PULMEM	0.415695	0.015233	pulmonary	TRUE	reported	textfile	11	72949747	0.001981	4.79E-16	475638	monocyte	TRUE	reported	igd	0.000139	65.94462	2	TRUE
rs1124210	T	G	T	G	-0.02246	-0.03345	0.478171	0.344453	FALSE	FALSE	FALSE	I9_PULMEM	0.034357	0.015811	pulmonary	TRUE	reported	textfile	5	1.32E+08	0.001875	4.75E-33	520504	monocyte	TRUE	reported	igd	0.000276	143.5262	2	TRUE
rs1124606	G	A	G	A	-0.02208	-0.00885	0.339085	0.256376	FALSE	FALSE	FALSE	I9_PULMEM	0.608655	0.017292	pulmonary	TRUE	reported	textfile	11	324170	0.00207	1.60E-26	475283	monocyte	TRUE	reported	igd	0.000239	113.7258	2	TRUE
rs1124790	A	G	A	G	-0.02925	0.000253	0.172916	0.20808	FALSE	FALSE	FALSE	I9_PULMEM	0.989066	0.018457	pulmonary	TRUE	reported	textfile	1	26638222	0.002464	1.92E-32	521592	monocyte	TRUE	reported	igd	0.00027	140.8705	2	TRUE
rs1125214	A	G	A	G	0.011664	0.007596	0.483966	0.454822	FALSE	FALSE	FALSE	I9_PULMEM	0.615171	0.01511	pulmonary	TRUE	reported	textfile	10	3917237	0.001872	4.79E-10	520191	monocyte	TRUE	reported	igd	7.46E-05	38.82234	2	TRUE

rs1125554	C	T	C	T	0.02006	0.012345	0.379159	0.368036	FALSE	FALSE	FALSE	I9_PULMEM	0.427742	0.015567	pulmonary	TRUE	reported	textfile	10	5894090	0.001931	2.99E-25	521585	monocyte	TRUE	reported	igd	0.000207	107.9184	2	TRUE
rs1127101	G	A	G	A	0.014398	-0.02019	0.603537	0.612869	FALSE	FALSE	FALSE	I9_PULMEM	0.189801	0.015396	pulmonary	TRUE	reported	textfile	2	2.2E+08	0.001903	3.98E-14	520189	monocyte	TRUE	reported	igd	0.00011	57.24336	2	TRUE
rs1130152	T	C	T	C	0.018546	-0.00926	0.210935	0.166999	FALSE	FALSE	FALSE	I9_PULMEM	0.64577	0.020155	pulmonary	TRUE	reported	textfile	8	57037166	0.002311	1.06E-15	521565	monocyte	TRUE	reported	igd	0.000123	64.40194	2	TRUE
rs1131438	T	C	T	C	0.034668	-0.06484	0.070781	0.055641	FALSE	FALSE	FALSE	I9_PULMEM	0.046907	0.03263	pulmonary	TRUE	reported	textfile	14	35338344	0.003669	3.61E-21	521584	monocyte	TRUE	reported	igd	0.000171	89.2813	2	TRUE
rs1131550	A	G	A	G	-0.04771	-0.0613	0.015283	0.037163	FALSE	FALSE	FALSE	I9_PULMEM	0.122477	0.039688	pulmonary	TRUE	reported	textfile	8	43719739	0.007969	2.21E-09	476441	monocyte	TRUE	reported	igd	7.52E-05	35.8374	2	TRUE
rs1132920	T	C	T	C	-0.02478	-0.04984	0.065718	0.055593	FALSE	FALSE	FALSE	I9_PULMEM	0.127205	0.032676	pulmonary	TRUE	reported	textfile	1	2.23E+08	0.003776	5.45E-11	521594	monocyte	TRUE	reported	igd	8.26E-05	43.08015	2	TRUE
rs1146933	C	T	C	T	-0.01403	0.015042	0.786362	0.835612	FALSE	FALSE	FALSE	I9_PULMEM	0.456467	0.020199	pulmonary	TRUE	reported	textfile	13	78404049	0.002287	8.88E-10	521591	monocyte	TRUE	reported	igd	7.21E-05	37.62335	2	TRUE
rs1150802	T	C	T	C	-0.01195	0.031709	0.431325	0.415466	FALSE	FALSE	FALSE	I9_PULMEM	0.03748	0.015241	pulmonary	TRUE	reported	textfile	16	56999328	0.00189	2.70E-10	520647	monocyte	TRUE	reported	igd	7.67E-05	39.95027	2	TRUE
rs1152028	A	G	A	G	0.027219	########	0.067747	0.078264	FALSE	FALSE	FALSE	I9_PULMEM	0.998565	0.028339	pulmonary	TRUE	reported	textfile	6	45533239	0.003757	4.52E-13	520654	monocyte	TRUE	reported	igd	0.000101	52.48803	2	TRUE
rs1153400	A	G	A	G	-0.09432	0.048328	0.027188	0.009232	FALSE	FALSE	FALSE	I9_PULMEM	0.538618	0.078594	pulmonary	TRUE	reported	textfile	1	92766438	0.005802	2.22E-59	520494	monocyte	TRUE	reported	igd	0.000507	264.2772	2	TRUE
rs1155715	T	C	T	C	0.031988	0.031478	0.127576	0.131548	FALSE	FALSE	FALSE	I9_PULMEM	0.155641	0.02217	pulmonary	TRUE	reported	textfile	9	34107505	0.002794	2.64E-30	520193	monocyte	TRUE	reported	igd	0.000252	131.075	2	TRUE
rs1159038	C	T	C	T	0.024736	0.003285	0.679418	0.717554	FALSE	FALSE	FALSE	I9_PULMEM	0.844222	0.016718	pulmonary	TRUE	reported	textfile	1	1.85E+08	0.002083	1.69E-32	478116	monocyte	TRUE	reported	igd	0.000295	141.0193	2	TRUE
rs1160232	G	T	G	T	0.06519	-0.03392	0.123096	0.161312	FALSE	FALSE	FALSE	I9_PULMEM	0.096137	0.020387	pulmonary	TRUE	reported	textfile	11	1.23E+08	0.002862	########	521562	monocyte	TRUE	reported	igd	0.000994	518.8252	2	TRUE
rs1162929	G	C	G	C	0.061045	-0.02148	0.732661	0.597681	FALSE	TRUE	FALSE	I9_PULMEM	0.16142	0.015342	pulmonary	TRUE	reported	textfile	14	1.04E+08	0.002145	########	521591	monocyte	TRUE	reported	igd	0.00155	809.9234	2	TRUE
rs1163141	T	C	T	C	-0.01168	-0.01584	0.572619	0.46962	FALSE	FALSE	FALSE	I9_PULMEM	0.292699	0.015051	pulmonary	TRUE	reported	textfile	15	86288673	0.001894	7.16E-10	520192	monocyte	TRUE	reported	igd	7.31E-05	38.0298	2	TRUE
rs1164412	T	C	T	C	-0.01945	-0.00486	0.597948	0.580096	FALSE	FALSE	FALSE	I9_PULMEM	0.750253	0.015279	pulmonary	TRUE	reported	textfile	16	57058974	0.001921	4.73E-24	520654	monocyte	TRUE	reported	igd	0.000197	102.4612	2	TRUE
rs1165588	T	C	T	C	-0.01414	-0.01485	0.180123	0.255406	FALSE	FALSE	FALSE	I9_PULMEM	0.388802	0.017233	pulmonary	TRUE	reported	textfile	17	79389411	0.002545	2.83E-08	477037	monocyte	TRUE	reported	igd	6.47E-05	30.87765	2	TRUE
rs1166199	A	G	A	G	-0.04026	0.057673	0.026454	0.022566	FALSE	FALSE	FALSE	I9_PULMEM	0.248546	0.049981	pulmonary	TRUE	reported	textfile	5	32214314	0.005861	6.73E-12	521131	monocyte	TRUE	reported	igd	9.05E-05	47.18249	2	TRUE
rs1171334	A	G	A	G	-0.02626	-0.03902	0.196605	0.215512	FALSE	FALSE	FALSE	I9_PULMEM	0.035801	0.018586	pulmonary	TRUE	reported	textfile	3	1.28E+08	0.002392	5.24E-28	519112	monocyte	TRUE	reported	igd	0.000232	120.5126	2	TRUE
rs1172362	G	A	G	A	-0.01321	-0.03222	0.290773	0.200351	FALSE	FALSE	FALSE	I9_PULMEM	0.085645	0.018743	pulmonary	TRUE	reported	textfile	4	72615362	0.002061	1.48E-10	521593	monocyte	TRUE	reported	igd	7.88E-05	41.10032	2	TRUE
rs1179203	A	G	A	G	0.02217	-0.05025	0.103551	0.093498	FALSE	FALSE	FALSE	I9_PULMEM	0.052534	0.025918	pulmonary	TRUE	reported	textfile	9	1.31E+08	0.003107	1.00E-12	521579	monocyte	TRUE	reported	igd	9.76E-05	50.91517	2	TRUE
rs1180658	A	C	A	C	-0.01254	-0.01287	0.633894	0.695642	FALSE	FALSE	FALSE	I9_PULMEM	0.429462	0.016291	pulmonary	TRUE	reported	textfile	10	61645945	0.001945	1.19E-10	520655	monocyte	TRUE	reported	igd	7.98E-05	41.53439	2	TRUE
rs1180838	A	G	A	G	-0.0965	0.026318	0.016672	0.007637	FALSE	FALSE	FALSE	I9_PULMEM	0.767519	0.089027	pulmonary	TRUE	reported	textfile	17	16522922	0.008517	1.01E-29	477020	monocyte	TRUE	reported	igd	0.000269	128.38	2	TRUE
rs1184435	G	A	G	A	-0.01254	0.000122	0.226227	0.2503	FALSE	FALSE	FALSE	I9_PULMEM	0.994369	0.017353	pulmonary	TRUE	reported	textfile	14	35356986	0.002243	2.32E-08	521593	monocyte	TRUE	reported	igd	5.99E-05	31.25112	2	TRUE
rs1185439	T	C	T	C	0.04248	0.015377	0.562044	0.594364	FALSE	FALSE	FALSE	I9_PULMEM	0.316212	0.015342	pulmonary	TRUE	reported	textfile	15	80224817	0.001892	########	521592	monocyte	TRUE	reported	igd	0.000966	504.1097	2	TRUE
rs1185760	C	T	C	T	0.023258	-0.01396	0.759798	0.708431	FALSE	FALSE	FALSE	I9_PULMEM	0.398815	0.016549	pulmonary	TRUE	reported	textfile	15	66095270	0.002198	3.99E-26	521591	monocyte	TRUE	reported	igd	0.000215	111.9664	2	TRUE
rs1196588	T	G	T	G	-0.0108	-0.01747	0.581843	0.58416	FALSE	FALSE	FALSE	I9_PULMEM	0.254191	0.015323	pulmonary	TRUE	reported	textfile	6	43693094	0.001925	2.09E-08	519571	monocyte	TRUE	reported	igd	6.05E-05	31.45304	2	TRUE
rs1205564	C	T	C	T	0.018089	0.03069	0.199011	0.106652	FALSE	FALSE	FALSE	I9_PULMEM	0.205631	0.024248	pulmonary	TRUE	reported	textfile	6	1.35E+08	0.002337	1.04E-14	521588	monocyte	TRUE	reported	igd	0.000115	59.91148	2	TRUE
rs1211844	A	G	A	G	-0.01803	-0.00848	0.489432	0.48955	FALSE	FALSE	FALSE	I9_PULMEM	0.571563	0.014989	pulmonary	TRUE	reported	textfile	1	90038588	0.001863	4.00E-22	521588	monocyte	TRUE	reported	igd	0.000179	93.64136	2	TRUE
rs1214411	T	C	T	C	-0.0141	0.003751	0.215407	0.230292	FALSE	FALSE	FALSE	I9_PULMEM	0.833391	0.01783	pulmonary	TRUE	reported	textfile	1	9182141	0.002289	7.63E-10	520509	monocyte	TRUE	reported	igd	7.28E-05	37.91726	2	TRUE
rs1219823	C	T	C	T	-0.01036	-0.02926	0.562093	0.471879	FALSE	FALSE	FALSE	I9_PULMEM	0.051636	0.015034	pulmonary	TRUE	reported	textfile	6	1.11E+08	0.001892	4.46E-08	521594	monocyte	TRUE	reported	igd	5.75E-05	29.98882	2	TRUE
rs1220769	G	A	G	A	-0.01167	0.0015	0.304304	0.230812	FALSE	FALSE	FALSE	I9_PULMEM	0.932672	0.01776	pulmonary	TRUE	reported	textfile	6	89832717	0.002031	9.49E-09	521593	monocyte	TRUE	reported	igd	6.33E-05	33.00436	2	TRUE
rs1230679	T	C	T	C	0.019129	0.006929	0.714634	0.695545	FALSE	FALSE	FALSE	I9_PULMEM	0.671373	0.016331	pulmonary	TRUE	reported	textfile	12	89864047	0.002066	2.22E-20	521594	monocyte	TRUE	reported	igd	0.000164	85.72791	2	TRUE
rs1232725	G	A	G	A	0.017782	-0.01157	0.293564	0.320813	FALSE	FALSE	FALSE	I9_PULMEM	0.473376	0.01613	pulmonary	TRUE	reported	textfile	18	20719164	0.002112	4.05E-17	521581	monocyte	TRUE	reported	igd	0.000136	70.88784	2	TRUE
rs1233267	G	T	G	T	-0.0165	-0.01597	0.152382	0.154914	FALSE	FALSE	FALSE	I9_PULMEM	0.445606	0.020931	pulmonary	TRUE	reported	textfile	5	1.32E+08	0.002625	3.33E-10	521592	monocyte	TRUE	reported	igd	7.58E-05	39.52921	2	TRUE
rs1237651	C	T	C	T	-0.04859	-0.04091	0.163934	0.189159	FALSE	FALSE	FALSE	I9_PULMEM	0.033626	0.019254	pulmonary	TRUE	reported	textfile	9	22142756	0.002529	3.44E-82	521592	monocyte	TRUE	reported	igd	0.000707	369.0669	2	TRUE
rs1246142	T	C	T	C	-0.01601	-0.01221	0.283017	0.353609	FALSE	FALSE	FALSE	I9_PULMEM	0.439195	0.015785	pulmonary	TRUE	reported	textfile	19	3167542	0.002202	3.77E-13	478115	monocyte	TRUE	reported	igd	0.000111	52.84911	2	TRUE
rs1248046	T	C	T	C	-0.03099	-0.01933	0.31601	0.453016	FALSE	FALSE	FALSE	I9_PULMEM	0.200252	0.01509	pulmonary	TRUE	reported	textfile	20	31065178	0.00203	1.44E-52	521591	monocyte	TRUE	reported	igd	0.000447	233.0652	2	TRUE
rs1248073	T	C	T	C	0.054282	-0.00866	0.257776	0.244585	FALSE	FALSE	FALSE	I9_PULMEM	0.619872	0.017466	pulmonary	TRUE	reported	textfile	20	31191015	0.002188	########	520647	monocyte	TRUE	reported	igd	0.001181	615.482	2	TRUE
rs1252249	G	A	G	A	-0.01094	-0.004	0.360634	0.321723	FALSE	FALSE	FALSE	I9_PULMEM	0.803437	0.016053	pulmonary	TRUE	reported	textfile	5	1.57E+08	0.001949	2.05E-08	520190	monocyte	TRUE	reported	igd	6.05E-05	31.48989	2	TRUE
rs1253007	C	T	C	T	-0.01469	-0.02205	0.224608	0.350953	FALSE	FALSE	FALSE	I9_PULMEM	0.160652	0.015721	pulmonary	TRUE	reported	textfile	6	2886067	0.002241	5.78E-11	520190	monocyte	TRUE	reported	igd	8.26E-05	42.96344	2	TRUE
rs1254290	G	C	G	C	-0.0242	-0.00111	0.398926	0.329651	FALSE	TRUE	FALSE	I9_PULMEM	0.944685	0.015937	pulmonary	TRUE	reported	textfile	8	68813188	0.001911	1.01E-36	521591	monocyte	TRUE	reported	igd	0.000307	160.351	2	TRUE
rs1269473	A	G	A	G	-0.01112	-0.00513	0.582142	0.573696	FALSE	FALSE	FALSE	I9_PULMEM	0.735104	0.015172	pulmonary	TRUE	reported	textfile	2	2.28E+08	0.001889	4.07E-09	520549	monocyte	TRUE	reported	igd	6.66E-05	34.64702	2	TRUE
rs1274242	G	A	G	A	-0.01675	0.024059	0.650574	0.586606	FALSE	FALSE	FALSE	I9_PULMEM	0.114022	0.015224	pulmonary	TRUE	reported	textfile	1	67426487	0.00203	1.68E-16	478115	monocyte	TRUE	reported	igd	0.000142	68.05815	2	TRUE

rs1274743	T	G	T	G	-0.02404	0.046246	0.066638	0.096737	FALSE	FALSE	FALSE	I9_PULMEN	0.069996	0.025523	pulmonary	TRUE	reported	textfile	1	1.99E+08	0.003738	1.31E-10	521590	monocyte	TRUE	reported	igd	7.93E-05	41.36077	2	TRUE
rs1283945	C	T	C	T	-0.03475	-0.07744	0.975589	0.991283	FALSE	FALSE	FALSE	I9_PULMEN	0.344635	0.081948	pulmonary	TRUE	reported	textfile	6	44572306	0.006118	1.38E-08	521198	monocyte	TRUE	reported	igd	6.19E-05	32.25812	2	TRUE
rs1287440	G	A	G	A	-0.0645	0.018599	0.055525	0.035735	FALSE	FALSE	FALSE	I9_PULMEN	0.650696	0.041076	pulmonary	TRUE	reported	textfile	13	1.09E+08	0.004124	4.19E-55	520652	monocyte	TRUE	reported	igd	0.00047	244.6363	2	TRUE
rs1293448	T	G	T	G	0.016179	0.007921	0.202264	0.163453	FALSE	FALSE	FALSE	I9_PULMEN	0.697798	0.0204	pulmonary	TRUE	reported	textfile	16	10988396	0.002344	5.28E-12	520653	monocyte	TRUE	reported	igd	9.15E-05	47.64161	2	TRUE
rs1294135	G	A	G	A	-0.01739	-0.02064	0.588457	0.536077	FALSE	FALSE	FALSE	I9_PULMEN	0.171822	0.015105	pulmonary	TRUE	reported	textfile	17	17716531	0.001989	2.45E-18	478051	monocyte	TRUE	reported	igd	0.00016	76.41488	2	TRUE
rs1299369	G	A	G	A	-0.01356	0.00328	0.241702	0.317808	FALSE	FALSE	FALSE	I9_PULMEN	0.83876	0.016118	pulmonary	TRUE	reported	textfile	2	2.32E+08	0.002191	6.32E-10	520650	monocyte	TRUE	reported	igd	7.35E-05	38.29172	2	TRUE
rs1302741	C	A	C	A	0.012109	-0.01241	0.317451	0.371783	FALSE	FALSE	FALSE	I9_PULMEN	0.425813	0.01558	pulmonary	TRUE	reported	textfile	2	1.29E+08	0.00207	5.02E-09	484298	monocyte	TRUE	reported	igd	7.07E-05	34.21953	2	TRUE
rs1303249	T	C	T	C	0.016018	0.003306	0.139101	0.117978	FALSE	FALSE	FALSE	I9_PULMEN	0.887067	0.023279	pulmonary	TRUE	reported	textfile	2	1.61E+08	0.002733	4.72E-09	520653	monocyte	TRUE	reported	igd	6.60E-05	34.3507	2	TRUE
rs1303278	G	C	G	C	0.020299	0.044348	0.299909	0.334458	FALSE	TRUE	FALSE	I9_PULMEN	0.005018	0.015806	pulmonary	TRUE	reported	textfile	2	1.49E+08	0.002038	2.49E-23	520616	monocyte	TRUE	reported	igd	0.000191	99.2063	2	TRUE
rs1307388	C	T	C	T	0.012652	-0.00655	0.315326	0.310776	FALSE	FALSE	FALSE	I9_PULMEN	0.68672	0.016234	pulmonary	TRUE	reported	textfile	3	1.2E+08	0.00201	3.16E-10	521564	monocyte	TRUE	reported	igd	7.60E-05	39.62092	2	TRUE
rs1307768	G	T	G	T	0.041426	0.030592	0.02315	0.021871	FALSE	FALSE	FALSE	I9_PULMEN	0.547703	0.050885	pulmonary	TRUE	reported	textfile	3	39149843	0.006385	8.99E-11	521590	monocyte	TRUE	reported	igd	8.07E-05	42.09423	2	TRUE
rs1309439	G	A	G	A	-0.02084	-0.02483	0.115983	0.16479	FALSE	FALSE	FALSE	I9_PULMEN	0.221268	0.020304	pulmonary	TRUE	reported	textfile	3	71817778	0.002927	1.11E-12	520654	monocyte	TRUE	reported	igd	9.74E-05	50.7125	2	TRUE
rs1316728	A	G	A	G	0.017435	0.034843	0.128106	0.156824	FALSE	FALSE	FALSE	I9_PULMEN	0.092075	0.020684	pulmonary	TRUE	reported	textfile	5	1280477	0.002996	6.07E-09	474344	monocyte	TRUE	reported	igd	7.14E-05	33.86558	2	TRUE
rs1322532	A	G	A	G	0.012802	-0.02497	0.305023	0.314646	FALSE	FALSE	FALSE	I9_PULMEN	0.122824	0.016183	pulmonary	TRUE	reported	textfile	7	1.07E+08	0.002041	3.69E-10	521576	monocyte	TRUE	reported	igd	7.54E-05	39.34305	2	TRUE
rs1322986	A	G	A	G	0.015633	-0.01013	0.197255	0.138314	FALSE	FALSE	FALSE	I9_PULMEN	0.649775	0.0223	pulmonary	TRUE	reported	textfile	7	6881302	0.002594	1.73E-09	475605	monocyte	TRUE	reported	igd	7.64E-05	36.31975	2	TRUE
rs1323819	T	C	T	C	-0.0216	-0.03743	0.107602	0.144476	FALSE	FALSE	FALSE	I9_PULMEN	0.079868	0.021369	pulmonary	TRUE	reported	textfile	7	49837504	0.003013	7.88E-13	521591	monocyte	TRUE	reported	igd	9.85E-05	51.38867	2	TRUE
rs1326746	T	C	T	C	-0.02048	0.003616	0.399388	0.446191	FALSE	FALSE	FALSE	I9_PULMEN	0.810851	0.015107	pulmonary	TRUE	reported	textfile	8	6906925	0.001911	8.90E-27	521591	monocyte	TRUE	reported	igd	0.00022	114.8852	2	TRUE
rs1327122	G	T	G	T	-0.03058	0.014708	0.567054	0.688987	FALSE	FALSE	FALSE	I9_PULMEN	0.366411	0.016284	pulmonary	TRUE	reported	textfile	8	1.17E+08	0.001886	4.35E-59	521592	monocyte	TRUE	reported	igd	0.000504	262.8996	2	TRUE
rs1335929	C	T	C	T	0.016628	-0.01196	0.570881	0.594311	FALSE	FALSE	FALSE	I9_PULMEN	0.434181	0.01529	pulmonary	TRUE	reported	textfile	1	2.21E+08	0.001894	1.77E-18	521591	monocyte	TRUE	reported	igd	0.000148	77.07587	2	TRUE
rs1367153	A	G	A	G	-0.01136	-0.00654	0.707555	0.648281	FALSE	FALSE	FALSE	I9_PULMEN	0.67732	0.015725	pulmonary	TRUE	reported	textfile	2	16641675	0.002056	3.39E-08	520649	monocyte	TRUE	reported	igd	5.86E-05	30.52336	2	TRUE
rs1380281	G	C	G	C	0.135387	-0.03935	0.03476	0.014493	FALSE	TRUE	FALSE	I9_PULMEN	0.534019	0.063273	pulmonary	TRUE	reported	textfile	13	28712689	0.005559	########	517343	monocyte	TRUE	reported	igd	0.001145	593.1423	2	TRUE
rs139271	C	T	C	T	-0.0153	0.012415	0.44222	0.530395	FALSE	FALSE	FALSE	I9_PULMEN	0.409948	0.015067	pulmonary	TRUE	reported	textfile	22	39487595	0.001907	1.11E-15	521593	monocyte	TRUE	reported	igd	0.000123	64.32739	2	TRUE
rs1400003	T	C	T	C	-0.01248	-0.00924	0.212658	0.151803	FALSE	FALSE	FALSE	I9_PULMEN	0.65835	0.020902	pulmonary	TRUE	reported	textfile	2	1.83E+08	0.002282	4.70E-08	520653	monocyte	TRUE	reported	igd	5.74E-05	29.88465	2	TRUE
rs1419361	G	A	G	A	-0.01865	-0.02446	0.350763	0.349692	FALSE	FALSE	FALSE	I9_PULMEN	0.120128	0.015736	pulmonary	TRUE	reported	textfile	4	1.03E+08	0.001964	2.29E-21	521589	monocyte	TRUE	reported	igd	0.000173	90.21096	2	TRUE
rs1432731	C	T	C	T	-0.06678	0.02718	0.259095	0.294996	FALSE	FALSE	FALSE	I9_PULMEN	0.098742	0.016463	pulmonary	TRUE	reported	textfile	19	45744552	0.002145	########	520150	monocyte	TRUE	reported	igd	0.00186	969.3678	2	TRUE
rs1443170	T	A	T	A	0.061822	0.010383	0.034154	0.057398	FALSE	TRUE	FALSE	I9_PULMEN	0.749604	0.032531	pulmonary	TRUE	reported	textfile	4	1.06E+08	0.005227	3.10E-32	521592	monocyte	TRUE	reported	igd	0.000268	139.8877	2	TRUE
rs1454474	C	T	C	T	0.045684	-0.00414	0.023067	0.016505	FALSE	FALSE	FALSE	I9_PULMEN	0.945141	0.06013	pulmonary	TRUE	reported	textfile	2	1.83E+08	0.006336	5.80E-13	520654	monocyte	TRUE	reported	igd	9.98E-05	51.98718	2	TRUE
rs1457180	A	G	A	G	-0.08024	-0.12606	0.01018	0.010214	FALSE	FALSE	FALSE	I9_PULMEN	0.095085	0.075522	pulmonary	TRUE	reported	textfile	8	48641192	0.009874	4.68E-16	518605	monocyte	TRUE	reported	igd	0.000127	66.02977	2	TRUE
rs1460396	A	G	A	G	0.071441	0.008041	0.019361	0.040749	FALSE	FALSE	FALSE	I9_PULMEN	0.834445	0.038473	pulmonary	TRUE	reported	textfile	7	92323422	0.007029	3.07E-24	521134	monocyte	TRUE	reported	igd	0.000198	103.3014	2	TRUE
rs1464741	A	G	A	G	-0.02678	0.021633	0.040528	0.067385	FALSE	FALSE	FALSE	I9_PULMEN	0.471275	0.030029	pulmonary	TRUE	reported	textfile	22	31493622	0.004828	2.99E-08	521592	monocyte	TRUE	reported	igd	5.90E-05	30.76009	2	TRUE
rs1466812	C	T	C	T	-0.012	0.004247	0.399438	0.383536	FALSE	FALSE	FALSE	I9_PULMEN	0.783071	0.015426	pulmonary	TRUE	reported	textfile	22	50767407	0.001999	2.01E-09	478109	monocyte	TRUE	reported	igd	7.53E-05	36.01786	2	TRUE
rs1468488	C	T	C	T	0.032537	0.000401	0.272855	0.269002	FALSE	FALSE	FALSE	I9_PULMEN	0.981132	0.01694	pulmonary	TRUE	reported	textfile	22	17590744	0.002139	3.36E-52	520190	monocyte	TRUE	reported	igd	0.000445	231.3832	2	TRUE
rs1473410	G	A	G	A	-0.07466	0.045327	0.027837	0.013201	FALSE	FALSE	FALSE	I9_PULMEN	0.491828	0.06594	pulmonary	TRUE	reported	textfile	7	92381696	0.005841	2.25E-37	521594	monocyte	TRUE	reported	igd	0.000313	163.376	2	TRUE
rs1490077	T	C	T	C	-0.07223	-0.01692	0.16205	0.206838	FALSE	FALSE	FALSE	I9_PULMEN	0.36432	0.018651	pulmonary	TRUE	reported	textfile	7	50370254	0.002651	########	521591	monocyte	TRUE	reported	igd	0.001421	742.4209	2	TRUE
rs1491105	T	C	T	C	0.074605	0.004169	0.036036	0.025923	FALSE	FALSE	FALSE	I9_PULMEN	0.929948	0.04742	pulmonary	TRUE	reported	textfile	6	1.44E+08	0.005093	1.51E-48	521590	monocyte	TRUE	reported	igd	0.000411	214.5788	2	TRUE
rs1499029	A	G	A	G	-0.01877	-0.01568	0.095147	0.108233	FALSE	FALSE	FALSE	I9_PULMEN	0.521518	0.024454	pulmonary	TRUE	reported	textfile	8	74928829	0.003291	1.21E-08	519733	monocyte	TRUE	reported	igd	6.26E-05	32.5221	2	TRUE
rs1504496	C	T	C	T	0.14024	0.070335	0.022455	0.008522	FALSE	FALSE	FALSE	I9_PULMEN	0.387167	0.081334	pulmonary	TRUE	reported	textfile	2	1.12E+08	0.006704	4.24E-97	520651	monocyte	TRUE	reported	igd	0.00084	437.5968	2	TRUE
rs1535701	G	T	G	T	0.020771	0.003454	0.621414	0.642725	FALSE	FALSE	FALSE	I9_PULMEN	0.825339	0.01565	pulmonary	TRUE	reported	textfile	1	93805201	0.001925	4.06E-27	521571	monocyte	TRUE	reported	igd	0.000223	116.4265	2	TRUE
rs1587222	G	A	G	A	0.013851	0.001892	0.701806	0.688318	FALSE	FALSE	FALSE	I9_PULMEN	0.907067	0.01621	pulmonary	TRUE	reported	textfile	4	79515724	0.002057	1.72E-11	520512	monocyte	TRUE	reported	igd	8.71E-05	45.3411	2	TRUE
rs1594541	C	T	C	T	0.015743	0.019273	0.195904	0.169068	FALSE	FALSE	FALSE	I9_PULMEN	0.335493	0.020011	pulmonary	TRUE	reported	textfile	17	32654403	0.002369	3.12E-11	520194	monocyte	TRUE	reported	igd	8.49E-05	44.16144	2	TRUE
rs1611236	A	G	A	G	-0.03374	-0.0091	0.322944	0.224527	FALSE	FALSE	FALSE	I9_PULMEN	0.612858	0.017986	pulmonary	TRUE	reported	textfile	6	29748690	0.002007	2.28E-63	519103	monocyte	TRUE	reported	igd	0.000544	282.614	2	TRUE
rs1683113	T	C	T	C	-0.01358	-0.03458	0.39739	0.45866	FALSE	FALSE	FALSE	I9_PULMEN	0.021536	0.015047	pulmonary	TRUE	reported	textfile	3	1.6E+08	0.001931	2.07E-12	520651	monocyte	TRUE	reported	igd	9.50E-05	49.48677	2	TRUE
rs1693960	A	G	A	G	-0.02521	-0.00969	0.146042	0.150267	FALSE	FALSE	FALSE	I9_PULMEN	0.643942	0.020967	pulmonary	TRUE	reported	textfile	8	79013333	0.002656	2.43E-21	520192	monocyte	TRUE	reported	igd	0.000173	90.07805	2	TRUE
rs1694032	A	G	A	G	0.038726	-0.01866	0.1123	0.114166	FALSE	FALSE	FALSE	I9_PULMEN	0.429512	0.023622	pulmonary	TRUE	reported	textfile	16	86076108	0.003003	5.15E-38	521588	monocyte	TRUE	reported	igd	0.000319	166.3003	2	TRUE

rs1697817	C	T	C	T	-0.02536	-0.0014	0.465252	0.460549	FALSE	FALSE	FALSE	I9_PULMEN	0.92569	0.015023	pulmonary	TRUE	reported	textfile	17	72761242	0.001908	2.89E-40	520508	monocyte	TRUE	reported	igd	0.000339	176.6468	2	TRUE
rs1700589	A	G	A	G	-0.05096	0.010211	0.184381	0.233377	FALSE	FALSE	FALSE	I9_PULMEN	0.564443	0.017719	pulmonary	TRUE	reported	textfile	4	83547862	0.002417	1.15E-98	520648	monocyte	TRUE	reported	igd	0.000853	444.5852	2	TRUE
rs1701172	G	C	G	C	-0.02277	-0.03654	0.233294	0.149798	FALSE	TRUE	FALSE	I9_PULMEN	0.0826	0.02105	pulmonary	TRUE	reported	textfile	10	50264204	0.002221	1.23E-24	521591	monocyte	TRUE	reported	igd	0.000202	105.1244	2	TRUE
rs1708623	C	G	C	G	-0.06856	-0.01836	0.041159	0.022024	FALSE	TRUE	FALSE	I9_PULMEN	0.717575	0.050752	pulmonary	TRUE	reported	textfile	13	28611186	0.00476	5.45E-47	521590	monocyte	TRUE	reported	igd	0.000398	207.45	2	TRUE
rs1715653	A	C	A	C	0.024733	-0.0062	0.159358	0.187654	FALSE	FALSE	FALSE	I9_PULMEN	0.747476	0.019262	pulmonary	TRUE	reported	textfile	7	28279488	0.002577	8.66E-22	520652	monocyte	TRUE	reported	igd	0.000177	92.11346	2	TRUE
rs1719675	T	C	T	C	-0.07245	0.007295	0.189945	0.233127	FALSE	FALSE	FALSE	I9_PULMEN	0.681346	0.017765	pulmonary	TRUE	reported	textfile	20	48887268	0.002408	########	520194	monocyte	TRUE	reported	igd	0.001737	905.3366	2	TRUE
rs1738788	C	G	C	G	-0.02778	0.015849	0.173567	0.243311	FALSE	TRUE	FALSE	I9_PULMEN	0.365283	0.017507	pulmonary	TRUE	reported	textfile	1	43419705	0.002504	1.45E-28	521590	monocyte	TRUE	reported	igd	0.000236	123.0908	2	TRUE
rs1749824	A	C	A	C	-0.01191	0.022747	0.432497	0.442342	FALSE	FALSE	FALSE	I9_PULMEN	0.131897	0.015097	pulmonary	TRUE	reported	textfile	10	80923862	0.00196	1.26E-09	478114	monocyte	TRUE	reported	igd	7.72E-05	36.91787	2	TRUE
rs1749874	G	T	G	T	-0.01826	-0.01047	0.17382	0.216506	FALSE	FALSE	FALSE	I9_PULMEN	0.566889	0.018286	pulmonary	TRUE	reported	textfile	13	1.09E+08	0.002489	2.27E-13	521592	monocyte	TRUE	reported	igd	0.000103	53.8149	2	TRUE
rs1754407	T	C	T	C	0.011294	-0.02738	0.449385	0.39421	FALSE	FALSE	FALSE	I9_PULMEN	0.074184	0.015333	pulmonary	TRUE	reported	textfile	7	47451572	0.001879	1.92E-09	521593	monocyte	TRUE	reported	igd	6.93E-05	36.1277	2	TRUE
rs1757549	T	C	T	C	0.366555	0.128382	0.013208	0.027255	FALSE	FALSE	FALSE	I9_PULMEN	0.004043	0.044658	pulmonary	TRUE	reported	textfile	1	92315896	0.009065	########	521131	monocyte	TRUE	reported	igd	0.003128	1635.088	2	TRUE
rs1765620	T	C	T	C	-0.05415	0.004973	0.321977	0.203856	FALSE	FALSE	FALSE	I9_PULMEN	0.789649	0.018641	pulmonary	TRUE	reported	textfile	5	1.5E+08	0.002035	########	519114	monocyte	TRUE	reported	igd	0.001362	708.0802	2	TRUE
rs1770043	T	C	T	C	-0.0285	-0.01555	0.057432	0.051073	FALSE	FALSE	FALSE	I9_PULMEN	0.647286	0.033995	pulmonary	TRUE	reported	textfile	7	17167825	0.004027	1.53E-12	521577	monocyte	TRUE	reported	igd	9.60E-05	50.07994	2	TRUE
rs17731	A	G	A	G	-0.01434	-0.0139	0.373507	0.263626	FALSE	FALSE	FALSE	I9_PULMEN	0.41393	0.017018	pulmonary	TRUE	reported	textfile	10	3821561	0.001938	1.45E-13	519568	monocyte	TRUE	reported	igd	0.000105	54.72772	2	TRUE
rs1775869	T	C	T	C	-0.10927	0.060829	0.028765	0.044003	FALSE	FALSE	FALSE	I9_PULMEN	0.097521	0.036711	pulmonary	TRUE	reported	textfile	18	60920854	0.005762	3.89E-80	519549	monocyte	TRUE	reported	igd	0.000692	359.6413	2	TRUE
rs1777620	C	A	C	A	-0.0106	0.001923	0.443406	0.465211	FALSE	FALSE	FALSE	I9_PULMEN	0.898629	0.015096	pulmonary	TRUE	reported	textfile	10	1.16E+08	0.001913	3.07E-08	520113	monocyte	TRUE	reported	igd	5.91E-05	30.72613	2	TRUE
rs1781799	T	C	T	C	0.011157	-0.00682	0.612681	0.588509	FALSE	FALSE	FALSE	I9_PULMEN	0.654785	0.01526	pulmonary	TRUE	reported	textfile	10	79697211	0.001931	7.83E-09	520653	monocyte	TRUE	reported	igd	6.41E-05	33.38325	2	TRUE
rs1786042	A	G	A	G	-0.01561	0.004387	0.179529	0.198917	FALSE	FALSE	FALSE	I9_PULMEN	0.815693	0.018819	pulmonary	TRUE	reported	textfile	2	2.02E+08	0.002431	1.39E-10	520651	monocyte	TRUE	reported	igd	7.92E-05	41.25312	2	TRUE
rs1800973	A	C	A	C	0.113424	-0.04108	0.061915	0.050233	FALSE	FALSE	FALSE	I9_PULMEN	0.228514	0.034113	pulmonary	TRUE	reported	textfile	12	69744014	0.003883	########	520190	monocyte	TRUE	reported	igd	0.001638	853.2444	2	TRUE
rs1822534	G	A	G	A	-0.03098	-0.01267	0.394011	0.352954	FALSE	FALSE	FALSE	I9_PULMEN	0.421003	0.015741	pulmonary	TRUE	reported	textfile	3	12266804	0.001911	4.69E-59	521585	monocyte	TRUE	reported	igd	0.000504	262.7918	2	TRUE
rs1833631	T	C	T	C	0.062591	-0.00345	0.020836	0.022307	FALSE	FALSE	FALSE	I9_PULMEN	0.945899	0.050884	pulmonary	TRUE	reported	textfile	9	1.14E+08	0.00662	3.44E-21	521566	monocyte	TRUE	reported	igd	0.000171	89.39354	2	TRUE
rs1863651	T	A	T	A	-0.01673	-0.01876	0.699445	0.704611	FALSE	TRUE	FALSE	I9_PULMEN	0.254794	0.016478	pulmonary	TRUE	reported	textfile	8	82066998	0.002057	4.32E-16	520513	monocyte	TRUE	reported	igd	0.000127	66.18038	2	TRUE
rs1869365	C	T	C	T	0.020982	0.009657	0.231166	0.243578	FALSE	FALSE	FALSE	I9_PULMEN	0.581553	0.017523	pulmonary	TRUE	reported	textfile	17	40293865	0.002226	4.54E-21	521583	monocyte	TRUE	reported	igd	0.00017	88.8468	2	TRUE
rs1881754	T	C	T	C	-0.0929	-0.07166	0.024394	0.025289	FALSE	FALSE	FALSE	I9_PULMEN	0.142007	0.048803	pulmonary	TRUE	reported	textfile	13	28595940	0.006733	2.93E-43	520119	monocyte	TRUE	reported	igd	0.000366	190.3802	2	TRUE
rs1883933	G	C	G	C	0.086506	-0.12866	0.010149	0.012598	FALSE	TRUE	FALSE	I9_PULMEN	0.052962	0.066483	pulmonary	TRUE	reported	textfile	1	28254136	0.0102	2.36E-17	474701	monocyte	TRUE	reported	igd	0.000151	71.92673	2	TRUE
rs1885525	A	G	A	G	0.012936	-0.00016	0.506048	0.518605	FALSE	FALSE	FALSE	I9_PULMEN	0.991368	0.015028	pulmonary	TRUE	reported	textfile	11	33904180	0.001888	7.62E-12	520511	monocyte	TRUE	reported	igd	9.02E-05	46.94554	2	TRUE
rs1889033	G	A	G	A	-0.01573	-0.02351	0.485351	0.541376	FALSE	FALSE	FALSE	I9_PULMEN	0.119514	0.015099	pulmonary	TRUE	reported	textfile	1	53211887	0.001863	3.18E-17	521570	monocyte	TRUE	reported	igd	0.000137	71.2993	2	TRUE
rs1892548	C	T	C	T	-0.0628	-0.00025	0.654819	0.695652	FALSE	FALSE	FALSE	I9_PULMEN	0.987701	0.016358	pulmonary	TRUE	reported	textfile	13	41002641	0.001987	########	520653	monocyte	TRUE	reported	igd	0.001915	998.8043	2	TRUE
rs1902796	G	A	G	A	-0.0118	-0.01636	0.735738	0.662659	FALSE	FALSE	FALSE	I9_PULMEN	0.304435	0.015935	pulmonary	TRUE	reported	textfile	5	1.61E+08	0.002127	2.97E-08	521593	monocyte	TRUE	reported	igd	5.90E-05	30.78229	2	TRUE
rs190982	A	G	A	G	-0.01241	0.033867	0.602656	0.695949	FALSE	FALSE	FALSE	I9_PULMEN	0.039159	0.01642	pulmonary	TRUE	reported	textfile	5	88223420	0.001944	1.77E-10	520652	monocyte	TRUE	reported	igd	7.83E-05	40.77832	2	TRUE
rs1930303	A	G	A	G	-0.06177	0.016008	0.66324	0.636241	FALSE	FALSE	FALSE	I9_PULMEN	0.304931	0.015604	pulmonary	TRUE	reported	textfile	9	1.14E+08	0.002001	########	520514	monocyte	TRUE	reported	igd	0.001827	952.9572	2	TRUE
rs1933295	G	A	G	A	-0.02438	0.028152	0.776592	0.74384	FALSE	FALSE	FALSE	I9_PULMEN	0.102229	0.017227	pulmonary	TRUE	reported	textfile	1	62107021	0.002335	1.74E-25	478114	monocyte	TRUE	reported	igd	0.000228	109.0164	2	TRUE
rs1954735	C	T	C	T	0.020807	-0.00037	0.168423	0.226835	FALSE	FALSE	FALSE	I9_PULMEN	0.983452	0.018054	pulmonary	TRUE	reported	textfile	8	1.08E+08	0.002504	1.01E-16	521590	monocyte	TRUE	reported	igd	0.000132	69.04761	2	TRUE
rs1967309	G	A	G	A	-0.02213	-0.02059	0.603077	0.617693	FALSE	FALSE	FALSE	I9_PULMEN	0.182135	0.015429	pulmonary	TRUE	reported	textfile	16	4065583	0.001924	1.43E-30	520655	monocyte	TRUE	reported	igd	0.000254	132.3212	2	TRUE
rs1970364	T	C	T	C	0.019264	-0.00072	0.264825	0.414579	FALSE	FALSE	FALSE	I9_PULMEN	0.96245	0.015285	pulmonary	TRUE	reported	textfile	6	1.14E+08	0.002125	1.32E-19	520644	monocyte	TRUE	reported	igd	0.000158	82.18137	2	TRUE
rs1976633	G	A	G	A	-0.01616	0.024104	0.834784	0.825248	FALSE	FALSE	FALSE	I9_PULMEN	0.222581	0.019762	pulmonary	TRUE	reported	textfile	3	1.42E+08	0.002511	1.25E-10	521592	monocyte	TRUE	reported	igd	7.94E-05	41.43324	2	TRUE
rs1992057	A	G	A	G	0.012056	0.011902	0.431615	0.360895	FALSE	FALSE	FALSE	I9_PULMEN	0.449019	0.015721	pulmonary	TRUE	reported	textfile	10	98427670	0.001901	2.34E-10	521589	monocyte	TRUE	reported	igd	7.71E-05	40.21986	2	TRUE
rs1997577	T	A	T	A	-0.02959	0.006936	0.154954	0.176331	FALSE	TRUE	FALSE	I9_PULMEN	0.723649	0.019616	pulmonary	TRUE	reported	textfile	21	16371102	0.002601	5.80E-30	521593	monocyte	TRUE	reported	igd	0.000248	129.4306	2	TRUE
rs2013605	T	G	T	G	0.018187	-0.03686	0.102362	0.115686	FALSE	FALSE	FALSE	I9_PULMEN	0.11662	0.023488	pulmonary	TRUE	reported	textfile	18	19726151	0.003113	5.27E-09	520649	monocyte	TRUE	reported	igd	6.56E-05	34.13204	2	TRUE
rs2022003	T	A	T	A	0.016809	0.008051	0.279384	0.347343	FALSE	TRUE	FALSE	I9_PULMEN	0.608859	0.015734	pulmonary	TRUE	reported	textfile	1	1.59E+08	0.002073	5.41E-16	521591	monocyte	TRUE	reported	igd	0.000126	65.74814	2	TRUE
rs2038700	C	T	C	T	0.039975	0.012197	0.394192	0.388643	FALSE	FALSE	FALSE	I9_PULMEN	0.428232	0.015396	pulmonary	TRUE	reported	textfile	14	25461989	0.001919	2.81E-96	521588	monocyte	TRUE	reported	igd	0.000831	433.9356	2	TRUE
rs205422	C	T	C	T	0.012805	-0.01489	0.697421	0.69186	FALSE	FALSE	FALSE	I9_PULMEN	0.36081	0.016296	pulmonary	TRUE	reported	textfile	16	28076858	0.002092	9.49E-10	520509	monocyte	TRUE	reported	igd	7.20E-05	37.46572	2	TRUE
rs2063996	C	T	C	T	-0.01133	0.005663	0.625943	0.550929	FALSE	FALSE	FALSE	I9_PULMEN	0.708223	0.015131	pulmonary	TRUE	reported	textfile	14	75820645	0.001949	6.20E-09	520192	monocyte	TRUE	reported	igd	6.50E-05	33.8115	2	TRUE
rs2084312	T	C	T	C	0.037684	-0.01467	0.800313	0.751197	FALSE	FALSE	FALSE	I9_PULMEN	0.396846	0.017315	pulmonary	TRUE	reported	textfile	17	72695211	0.002351	8.97E-58	520512	monocyte	TRUE	reported	igd	0.000493	256.9254	2	TRUE

rs2106135	T	C	T	C	-0.05253	-0.04593	0.018357	0.026294	FALSE	FALSE	FALSE	I9_PULMEN	0.325937	0.046757	pulmonary	TRUE	reported	textfile	7	92299964	0.007005	6.76E-14	521588	monocyte	TRUE	reported	igd	0.000108	56.22516	2	TRUE
rs2136451	A	G	A	G	0.015051	-0.01824	0.298482	0.313641	FALSE	FALSE	FALSE	I9_PULMEN	0.258895	0.016153	pulmonary	TRUE	reported	textfile	2	64993045	0.002047	2.00E-13	520194	monocyte	TRUE	reported	igd	0.000104	54.06216	2	TRUE
rs2176777	C	G	C	G	0.054647	-0.01176	0.812825	0.834564	FALSE	TRUE	FALSE	I9_PULMEN	0.560013	0.020169	pulmonary	TRUE	reported	textfile	16	86012521	0.002429	########	521594	monocyte	TRUE	reported	igd	0.000969	506.1462	2	TRUE
rs2184697	G	T	G	T	-0.01471	-0.0079	0.292709	0.286891	FALSE	FALSE	FALSE	I9_PULMEN	0.634468	0.016609	pulmonary	TRUE	reported	textfile	10	89851815	0.00207	1.23E-12	520192	monocyte	TRUE	reported	igd	9.71E-05	50.50589	2	TRUE
rs2213290	T	C	T	C	0.035585	0.046127	0.407826	0.32896	FALSE	FALSE	FALSE	I9_PULMEN	0.003699	0.015891	pulmonary	TRUE	reported	textfile	3	46406367	0.001932	1.11E-75	521590	monocyte	TRUE	reported	igd	0.00065	339.2486	2	TRUE
rs2228467	C	T	C	T	0.157057	-0.01935	0.061974	0.076263	FALSE	FALSE	FALSE	I9_PULMEN	0.494234	0.028304	pulmonary	TRUE	reported	textfile	3	42906116	0.003864	########	520192	monocyte	TRUE	reported	igd	0.003166	1652.109	2	TRUE
rs2239630	G	A	G	A	0.048843	-0.01233	0.555952	0.471316	FALSE	FALSE	FALSE	I9_PULMEN	0.411604	0.015016	pulmonary	TRUE	reported	textfile	14	23589349	0.001897	########	521592	monocyte	TRUE	reported	igd	0.001269	662.931	2	TRUE
rs224111	A	G	A	G	-0.02093	-0.00092	0.390158	0.400337	FALSE	FALSE	FALSE	I9_PULMEN	0.95206	0.015327	pulmonary	TRUE	reported	textfile	10	64552010	0.00192	1.16E-27	521589	monocyte	TRUE	reported	igd	0.000228	118.8777	2	TRUE
rs2253843	G	A	G	A	-0.01306	-0.02046	0.278303	0.180301	FALSE	FALSE	FALSE	I9_PULMEN	0.295211	0.01955	pulmonary	TRUE	reported	textfile	9	1.3E+08	0.002133	9.39E-10	520473	monocyte	TRUE	reported	igd	7.21E-05	37.51189	2	TRUE
rs2273215	A	G	A	G	-0.01813	0.015993	0.458222	0.39013	FALSE	FALSE	FALSE	I9_PULMEN	0.300117	0.015434	pulmonary	TRUE	reported	textfile	6	1.71E+08	0.001963	2.72E-20	477174	monocyte	TRUE	reported	igd	0.000179	85.30082	2	TRUE
rs2274664	T	C	T	C	-0.01517	0.003884	0.580335	0.590922	FALSE	FALSE	FALSE	I9_PULMEN	0.800075	0.015337	pulmonary	TRUE	reported	textfile	1	54704629	0.001918	2.75E-15	519571	monocyte	TRUE	reported	igd	0.00012	62.53174	2	TRUE
rs2295481	T	C	T	C	-0.01525	-0.02421	0.207397	0.209664	FALSE	FALSE	FALSE	I9_PULMEN	0.187097	0.018352	pulmonary	TRUE	reported	textfile	20	61574727	0.002338	7.15E-11	521591	monocyte	TRUE	reported	igd	8.16E-05	42.55063	2	TRUE
rs2302774	T	G	T	G	-0.0242	0.021043	0.382509	0.335206	FALSE	FALSE	FALSE	I9_PULMEN	0.185171	0.015882	pulmonary	TRUE	reported	textfile	17	38183090	0.001932	5.68E-36	521587	monocyte	TRUE	reported	igd	0.000301	156.936	2	TRUE
rs231988	C	T	C	T	0.025769	0.057143	0.868197	0.90529	FALSE	FALSE	FALSE	I9_PULMEN	0.025987	0.025666	pulmonary	TRUE	reported	textfile	3	1.72E+08	0.002888	4.80E-19	478118	monocyte	TRUE	reported	igd	0.000166	79.61574	2	TRUE
rs2327028	T	C	T	C	-0.0112	0.013066	0.474846	0.499614	FALSE	FALSE	FALSE	I9_PULMEN	0.38369	0.015	pulmonary	TRUE	reported	textfile	20	8140925	0.001883	2.80E-09	521592	monocyte	TRUE	reported	igd	6.78E-05	35.35909	2	TRUE
rs2338021	C	T	C	T	-0.0317	-0.01528	0.879688	0.875888	FALSE	FALSE	FALSE	I9_PULMEN	0.50114	0.022718	pulmonary	TRUE	reported	textfile	5	71749094	0.002879	3.64E-28	520513	monocyte	TRUE	reported	igd	0.000233	121.2595	2	TRUE
rs2371108	T	G	T	G	0.01062	0.019244	0.38831	0.378826	FALSE	FALSE	FALSE	I9_PULMEN	0.215111	0.015524	pulmonary	TRUE	reported	textfile	3	27757018	0.001915	3.01E-08	520647	monocyte	TRUE	reported	igd	5.91E-05	30.75458	2	TRUE
rs2385094	C	T	C	T	-0.02392	0.001728	0.122214	0.137852	FALSE	FALSE	FALSE	I9_PULMEN	0.936742	0.021768	pulmonary	TRUE	reported	textfile	8	1.26E+08	0.002877	9.73E-17	521592	monocyte	TRUE	reported	igd	0.000133	69.13749	2	TRUE
rs2504209	G	A	G	A	0.018355	-0.00451	0.81111	0.760857	FALSE	FALSE	FALSE	I9_PULMEN	0.798861	0.017703	pulmonary	TRUE	reported	textfile	13	28546766	0.002421	3.58E-14	521592	monocyte	TRUE	reported	igd	0.00011	57.4801	2	TRUE
rs2523562	T	C	T	C	-0.06847	-0.01192	0.245752	0.232807	FALSE	FALSE	FALSE	I9_PULMEN	0.50154	0.017737	pulmonary	TRUE	reported	textfile	6	31330618	0.00217	########	520514	monocyte	TRUE	reported	igd	0.001909	995.704	2	TRUE
rs2535393	C	T	C	T	-0.01523	0.013961	0.598517	0.546548	FALSE	FALSE	FALSE	I9_PULMEN	0.355243	0.015102	pulmonary	TRUE	reported	textfile	12	711662	0.001929	3.14E-15	518765	monocyte	TRUE	reported	igd	0.00012	62.30251	2	TRUE
rs2548257	A	C	A	C	0.012909	0.001091	0.653021	0.646022	FALSE	FALSE	FALSE	I9_PULMEN	0.944488	0.015672	pulmonary	TRUE	reported	textfile	5	1E+08	0.001966	5.35E-11	520514	monocyte	TRUE	reported	igd	8.28E-05	43.11382	2	TRUE
rs2665405	A	G	A	G	0.038394	-0.00399	0.548346	0.537767	FALSE	FALSE	FALSE	I9_PULMEN	0.790582	0.015037	pulmonary	TRUE	reported	textfile	17	57875292	0.001876	5.37E-93	521592	monocyte	TRUE	reported	igd	0.000802	418.8508	2	TRUE
rs2711981	T	C	T	C	-0.0216	0.013376	0.663087	0.579854	FALSE	FALSE	FALSE	I9_PULMEN	0.378599	0.015192	pulmonary	TRUE	reported	textfile	4	39039258	0.001986	1.59E-27	520191	monocyte	TRUE	reported	igd	0.000227	118.2898	2	TRUE
rs2729450	C	T	C	T	-0.03064	0.013578	0.524171	0.507166	FALSE	FALSE	FALSE	I9_PULMEN	0.364323	0.014968	pulmonary	TRUE	reported	textfile	17	28088459	0.001881	1.36E-59	521584	monocyte	TRUE	reported	igd	0.000508	265.2507	2	TRUE
rs2734047	G	A	G	A	0.077151	0.028246	0.635458	0.678777	FALSE	FALSE	FALSE	I9_PULMEN	0.081003	0.016188	pulmonary	TRUE	reported	textfile	3	1.28E+08	0.001952	########	521587	monocyte	TRUE	reported	igd	0.002986	1562.147	2	TRUE
rs2734442	A	G	A	G	-0.042	0.029369	0.875502	0.875412	FALSE	FALSE	FALSE	I9_PULMEN	0.195627	0.022694	pulmonary	TRUE	reported	textfile	12	10600368	0.002846	3.00E-49	520195	monocyte	TRUE	reported	igd	0.000418	217.7742	2	TRUE
rs2784250	G	T	G	T	-0.01691	-0.00513	0.140201	0.112431	FALSE	FALSE	FALSE	I9_PULMEN	0.828343	0.023674	pulmonary	TRUE	reported	textfile	1	2.21E+08	0.002685	3.11E-10	521583	monocyte	TRUE	reported	igd	7.60E-05	39.64997	2	TRUE
rs2797681	C	G	C	G	-0.01482	-0.01423	0.255654	0.164806	FALSE	TRUE	FALSE	I9_PULMEN	0.492672	0.020739	pulmonary	TRUE	reported	textfile	6	1.38E+08	0.002297	1.14E-10	520509	monocyte	TRUE	reported	igd	8.00E-05	41.62677	2	TRUE
rs2810883	T	C	T	C	-0.01261	0.013077	0.544768	0.644133	FALSE	FALSE	FALSE	I9_PULMEN	0.404677	0.015693	pulmonary	TRUE	reported	textfile	1	89332165	0.001878	1.94E-11	521591	monocyte	TRUE	reported	igd	8.65E-05	45.11415	2	TRUE
rs2817441	T	C	T	C	-0.02173	-0.01702	0.279268	0.279612	FALSE	FALSE	FALSE	I9_PULMEN	0.312001	0.016834	pulmonary	TRUE	reported	textfile	6	1.57E+08	0.0021	4.67E-25	519571	monocyte	TRUE	reported	igd	0.000206	107.0728	2	TRUE
rs2834670	G	A	G	A	0.026935	-0.02961	0.176408	0.174844	FALSE	FALSE	FALSE	I9_PULMEN	0.138415	0.019982	pulmonary	TRUE	reported	textfile	21	36280376	0.002965	1.11E-19	520515	monocyte	TRUE	reported	igd	0.000159	82.5245	2	TRUE
rs2836220	C	A	C	A	-0.01137	-0.02221	0.338458	0.519538	FALSE	FALSE	FALSE	I9_PULMEN	0.139578	0.015034	pulmonary	TRUE	reported	textfile	21	39600499	0.001982	1.01E-08	521592	monocyte	TRUE	reported	igd	6.31E-05	32.89143	2	TRUE
rs2836759	G	C	G	C	-0.02032	0.0156	0.272849	0.364593	FALSE	TRUE	FALSE	I9_PULMEN	0.317309	0.0156	pulmonary	TRUE	reported	textfile	6	31260262	0.002101	4.39E-22	520509	monocyte	TRUE	reported	igd	0.00018	93.49317	2	TRUE
rs284324	A	G	A	G	0.02119	-0.00407	0.495314	0.605187	FALSE	FALSE	FALSE	I9_PULMEN	0.791384	0.015374	pulmonary	TRUE	reported	textfile	1	10724236	0.00197	5.94E-27	477177	monocyte	TRUE	reported	igd	0.000242	115.6985	2	TRUE
rs2846573	T	C	T	C	-0.01079	0.018767	0.351779	0.413269	FALSE	FALSE	FALSE	I9_PULMEN	0.218673	0.015257	pulmonary	TRUE	reported	textfile	18	841779	0.001972	4.51E-08	519100	monocyte	TRUE	reported	igd	5.77E-05	29.94942	2	TRUE
rs2849828	T	A	T	A	0.021103	0.023866	0.257557	0.261438	FALSE	TRUE	FALSE	I9_PULMEN	0.163186	0.017115	pulmonary	TRUE	reported	textfile	2	43360065	0.002169	2.41E-22	521586	monocyte	TRUE	reported	igd	0.000181	94.66025	2	TRUE
rs2861249	A	G	A	G	0.014459	0.006866	0.172531	0.168553	FALSE	FALSE	FALSE	I9_PULMEN	0.731375	0.02	pulmonary	TRUE	reported	textfile	4	1.48E+08	0.002482	5.82E-09	521591	monocyte	TRUE	reported	igd	6.51E-05	33.93683	2	TRUE
rs2872270	T	A	T	A	0.034302	-0.02988	0.149045	0.099144	FALSE	TRUE	FALSE	I9_PULMEN	0.236471	0.025237	pulmonary	TRUE	reported	textfile	5	55453942	0.002634	1.00E-38	521582	monocyte	TRUE	reported	igd	0.000325	169.5921	2	TRUE
rs2872353	A	C	A	C	-0.01531	0.003476	0.134572	0.169224	FALSE	FALSE	FALSE	I9_PULMEN	0.862916	0.020132	pulmonary	TRUE	reported	textfile	4	1.06E+08	0.002758	2.93E-08	521592	monocyte	TRUE	reported	igd	5.91E-05	30.80681	2	TRUE
rs290243	A	G	A	G	0.014337	0.014708	0.214516	0.365817	FALSE	FALSE	FALSE	I9_PULMEN	0.346429	0.015621	pulmonary	TRUE	reported	textfile	9	93571337	0.002274	3.00E-10	520651	monocyte	TRUE	reported	igd	7.63E-05	39.74971	2	TRUE
rs2927	C	T	C	T	-0.01656	-0.01894	0.580121	0.510095	FALSE	FALSE	FALSE	I9_PULMEN	0.206734	0.015001	pulmonary	TRUE	reported	textfile	6	16758112	0.001898	2.85E-18	520189	monocyte	TRUE	reported	igd	0.000146	76.11568	2	TRUE
rs2978889	A	G	A	G	-0.01914	0.017568	0.503921	0.465813	FALSE	FALSE	FALSE	I9_PULMEN	0.243339	0.015058	pulmonary	TRUE	reported	textfile	8	6697316	0.001885	3.43E-24	518763	monocyte	TRUE	reported	igd	0.000199	103.0786	2	TRUE
rs30102	C	G	C	G	-0.01304	-0.0262	0.316993	0.223333	FALSE	TRUE	FALSE	I9_PULMEN	0.148015	0.018112	pulmonary	TRUE	reported	textfile	2	2.38E+08	0.002014	9.89E-11	520647	monocyte	TRUE	reported	igd	8.05E-05	41.89557	2	TRUE

rs3012415	T	C	T	C	0.020349	-0.0219	0.822848	0.868747	FALSE	FALSE	FALSE	I9_PULMEN	0.326015	0.022296	pulmonary	TRUE	reported	textfile	6	1.7E+08	0.002546	1.37E-15	478118	monocyte	TRUE	reported	igd	0.000134	63.88039	2	TRUE
rs3014807	T	G	T	G	0.025554	0.018304	0.118614	0.08677	FALSE	FALSE	FALSE	I9_PULMEN	0.491841	0.026629	pulmonary	TRUE	reported	textfile	1	1.53E+08	0.002989	1.31E-17	486333	monocyte	TRUE	reported	igd	0.00015	73.09104	2	TRUE
rs3027012	T	C	T	C	0.028123	0.000861	0.182687	0.159871	FALSE	FALSE	FALSE	I9_PULMEN	0.966522	0.020519	pulmonary	TRUE	reported	textfile	1	1.59E+08	0.002424	4.37E-31	521589	monocyte	TRUE	reported	igd	0.000258	134.6035	2	TRUE
rs3111414	G	C	G	C	0.017402	0.002501	0.794447	0.795579	FALSE	TRUE	FALSE	I9_PULMEN	0.893152	0.018623	pulmonary	TRUE	reported	textfile	2	8443859	0.002313	5.62E-14	519576	monocyte	TRUE	reported	igd	0.000109	56.60377	2	TRUE
rs31243	G	A	G	A	-0.02256	-0.00648	0.09625	0.117979	FALSE	FALSE	FALSE	I9_PULMEN	0.780836	0.023288	pulmonary	TRUE	reported	textfile	5	75594360	0.003179	1.35E-12	520193	monocyte	TRUE	reported	igd	9.68E-05	50.33881	2	TRUE
rs3128959	A	G	A	G	0.031818	-0.04358	0.120518	0.134494	FALSE	FALSE	FALSE	I9_PULMEN	0.047586	0.021997	pulmonary	TRUE	reported	textfile	6	33048380	0.00289	3.74E-28	521584	monocyte	TRUE	reported	igd	0.000232	121.2128	2	TRUE
rs3177609	C	T	C	T	0.031206	0.005565	0.077556	0.084545	FALSE	FALSE	FALSE	I9_PULMEN	0.837639	0.027159	pulmonary	TRUE	reported	textfile	18	74071078	0.003521	8.26E-19	521593	monocyte	TRUE	reported	igd	0.000151	78.54933	2	TRUE
rs3184504	C	T	C	T	-0.04708	-0.0286	0.517396	0.59144	FALSE	FALSE	FALSE	I9_PULMEN	0.060823	0.015254	pulmonary	TRUE	reported	textfile	12	1.12E+08	0.001872	########	519112	monocyte	TRUE	reported	igd	0.001217	632.3642	2	TRUE
rs329125	A	C	A	C	-0.01664	0.018471	0.207081	0.186437	FALSE	FALSE	FALSE	I9_PULMEN	0.340078	0.019361	pulmonary	TRUE	reported	textfile	5	1.34E+08	0.002346	1.38E-12	520190	monocyte	TRUE	reported	igd	9.67E-05	50.29729	2	TRUE
rs333947	A	G	A	G	0.036848	0.007125	0.150214	0.16624	FALSE	FALSE	FALSE	I9_PULMEN	0.724753	0.020234	pulmonary	TRUE	reported	textfile	1	1.1E+08	0.002635	2.21E-44	519569	monocyte	TRUE	reported	igd	0.000376	195.5532	2	TRUE
rs3409784	T	C	T	C	-0.10055	-0.01439	0.05601	0.089315	FALSE	FALSE	FALSE	I9_PULMEN	0.589059	0.026637	pulmonary	TRUE	reported	textfile	17	56358429	0.00424	########	519714	monocyte	TRUE	reported	igd	0.001081	562.4259	2	TRUE
rs3423635	T	C	T	C	-0.03224	-0.00649	0.182541	0.267415	FALSE	FALSE	FALSE	I9_PULMEN	0.703286	0.01704	pulmonary	TRUE	reported	textfile	2	2.42E+08	0.002507	8.44E-38	477175	monocyte	TRUE	reported	igd	0.000346	165.3274	2	TRUE
rs3428141	T	C	T	C	-0.02108	-0.01387	0.137178	0.089587	FALSE	FALSE	FALSE	I9_PULMEN	0.596857	0.026225	pulmonary	TRUE	reported	textfile	3	1.36E+08	0.002706	7.01E-15	521588	monocyte	TRUE	reported	igd	0.000116	60.70264	2	TRUE
rs3441762	A	G	A	G	0.013122	########	0.23399	0.235788	FALSE	FALSE	FALSE	I9_PULMEN	0.999215	0.01779	pulmonary	TRUE	reported	textfile	1	54955589	0.002203	2.65E-09	521590	monocyte	TRUE	reported	igd	6.80E-05	35.47884	2	TRUE
rs3447861	A	G	A	G	0.013464	-0.0109	0.241781	0.16925	FALSE	FALSE	FALSE	I9_PULMEN	0.586707	0.020043	pulmonary	TRUE	reported	textfile	9	35753898	0.002185	7.35E-10	520652	monocyte	TRUE	reported	igd	7.29E-05	37.97027	2	TRUE
rs3450510	G	A	G	A	-0.03313	-0.03635	0.305345	0.278001	FALSE	FALSE	FALSE	I9_PULMEN	0.030892	0.016841	pulmonary	TRUE	reported	textfile	22	24624609	0.002083	6.38E-57	521575	monocyte	TRUE	reported	igd	0.000485	252.9816	2	TRUE
rs3460012	T	G	T	G	0.022368	-0.01939	0.217724	0.196404	FALSE	FALSE	FALSE	I9_PULMEN	0.305025	0.018908	pulmonary	TRUE	reported	textfile	20	1597433	0.002266	6.03E-23	521590	monocyte	TRUE	reported	igd	0.000187	97.439	2	TRUE
rs3474312	A	G	A	G	-0.04673	-0.07574	0.020053	0.009001	FALSE	FALSE	FALSE	I9_PULMEN	0.345619	0.080312	pulmonary	TRUE	reported	textfile	2	1.82E+08	0.00679	6.15E-12	520654	monocyte	TRUE	reported	igd	9.09E-05	47.35611	2	TRUE
rs3474688	C	G	C	G	-0.05823	0.095925	0.017439	0.004471	FALSE	TRUE	FALSE	I9_PULMEN	0.407792	0.115881	pulmonary	TRUE	reported	textfile	16	67221444	0.007327	2.00E-15	517523	monocyte	TRUE	reported	igd	0.000122	63.15745	2	TRUE
rs3506849	C	T	C	T	0.014581	-0.03912	0.20924	0.161423	FALSE	FALSE	FALSE	I9_PULMEN	0.056003	0.020469	pulmonary	TRUE	reported	textfile	21	36238307	0.00235	5.68E-10	521588	monocyte	TRUE	reported	igd	7.38E-05	38.49792	2	TRUE
rs3510872	A	G	A	G	-0.02286	-0.00069	0.056937	0.056589	FALSE	FALSE	FALSE	I9_PULMEN	0.982896	0.032274	pulmonary	TRUE	reported	textfile	8	38888285	0.004105	2.63E-08	521594	monocyte	TRUE	reported	igd	5.94E-05	31.00619	2	TRUE
rs3597982	T	C	T	C	-0.04606	0.060701	0.070849	0.022673	FALSE	FALSE	FALSE	I9_PULMEN	0.238623	0.05151	pulmonary	TRUE	reported	textfile	12	54685880	0.003696	1.28E-35	520191	monocyte	TRUE	reported	igd	0.000298	155.3106	2	TRUE
rs360017	G	A	G	A	-0.02448	0.021582	0.775236	0.777605	FALSE	FALSE	FALSE	I9_PULMEN	0.230819	0.018011	pulmonary	TRUE	reported	textfile	5	1.73E+08	0.002251	1.62E-27	520655	monocyte	TRUE	reported	igd	0.000227	118.2785	2	TRUE
rs3602651	C	G	C	G	-0.0167	-0.0024	0.198672	0.194292	FALSE	TRUE	FALSE	I9_PULMEN	0.899326	0.018963	pulmonary	TRUE	reported	textfile	16	74597758	0.002341	1.02E-12	520649	monocyte	TRUE	reported	igd	9.77E-05	50.88344	2	TRUE
rs3731211	A	T	A	T	0.028382	-0.00558	0.719766	0.772015	FALSE	TRUE	FALSE	I9_PULMEN	0.754505	0.017851	pulmonary	TRUE	reported	textfile	9	21986847	0.002081	2.51E-42	521592	monocyte	TRUE	reported	igd	0.000356	186.0117	2	TRUE
rs3731332	T	C	T	C	-0.10801	0.041051	0.022895	0.018182	FALSE	FALSE	FALSE	I9_PULMEN	0.467371	0.056484	pulmonary	TRUE	reported	textfile	7	92300568	0.006386	4.01E-64	520654	monocyte	TRUE	reported	igd	0.000549	286.0621	2	TRUE
rs3761986	C	T	C	T	-0.01215	-0.0231	0.394272	0.431045	FALSE	FALSE	FALSE	I9_PULMEN	0.130898	0.015295	pulmonary	TRUE	reported	textfile	6	6680510	0.00195	4.88E-10	516280	monocyte	TRUE	reported	igd	7.51E-05	38.79678	2	TRUE
rs3762281	G	A	G	A	0.013022	-0.00569	0.505158	0.420499	FALSE	FALSE	FALSE	I9_PULMEN	0.708287	0.015217	pulmonary	TRUE	reported	textfile	1	1.56E+08	0.001864	2.97E-12	521592	monocyte	TRUE	reported	igd	9.36E-05	48.80473	2	TRUE
rs3789062	T	C	T	C	-0.02008	0.002215	0.245372	0.256046	FALSE	FALSE	FALSE	I9_PULMEN	0.897568	0.017209	pulmonary	TRUE	reported	textfile	2	1.12E+08	0.002179	3.22E-20	520653	monocyte	TRUE	reported	igd	0.000163	84.93717	2	TRUE
rs3795503	T	C	T	C	-0.01889	0.006107	0.314513	0.35507	FALSE	FALSE	FALSE	I9_PULMEN	0.697707	0.015724	pulmonary	TRUE	reported	textfile	1	1.81E+08	0.002034	1.68E-20	521586	monocyte	TRUE	reported	igd	0.000165	86.25025	2	TRUE
rs3808609	C	G	C	G	0.016911	-0.01876	0.318017	0.35713	FALSE	TRUE	FALSE	I9_PULMEN	0.230482	0.015648	pulmonary	TRUE	reported	textfile	8	59465377	0.002008	3.86E-17	521591	monocyte	TRUE	reported	igd	0.000136	70.92666	2	TRUE
rs3856364	G	C	G	C	0.012622	0.005623	0.67981	0.631252	FALSE	TRUE	FALSE	I9_PULMEN	0.717634	0.015551	pulmonary	TRUE	reported	textfile	2	1.45E+08	0.002009	3.45E-10	519575	monocyte	TRUE	reported	igd	7.60E-05	39.47252	2	TRUE
rs3859570	C	T	C	T	-0.02785	0.016783	0.424757	0.472657	FALSE	FALSE	FALSE	I9_PULMEN	0.267753	0.015144	pulmonary	TRUE	reported	textfile	19	18510925	0.001998	3.97E-44	478116	monocyte	TRUE	reported	igd	0.000406	194.2793	2	TRUE
rs391023	T	C	T	C	-0.0759	0.00998	0.366325	0.36254	FALSE	FALSE	FALSE	I9_PULMEN	0.523122	0.01563	pulmonary	TRUE	reported	textfile	16	85927814	0.001942	########	520192	monocyte	TRUE	reported	igd	0.002928	1527.629	2	TRUE
rs4126809	A	G	A	G	0.029048	-0.00365	0.110855	0.081478	FALSE	FALSE	FALSE	I9_PULMEN	0.893761	0.027348	pulmonary	TRUE	reported	textfile	1	41328644	0.002986	2.47E-22	521591	monocyte	TRUE	reported	igd	0.000181	94.63487	2	TRUE
rs4131338	A	C	A	C	0.043019	0.029334	0.030485	0.017483	FALSE	FALSE	FALSE	I9_PULMEN	0.610536	0.057596	pulmonary	TRUE	reported	textfile	1	79411968	0.005345	8.85E-16	519116	monocyte	TRUE	reported	igd	0.000125	64.7774	2	TRUE
rs413141	G	A	G	A	0.043013	-0.02738	0.862523	0.757495	FALSE	FALSE	FALSE	I9_PULMEN	0.121951	0.017702	pulmonary	TRUE	reported	textfile	19	6675989	0.002764	1.42E-54	521593	monocyte	TRUE	reported	igd	0.000464	242.1708	2	TRUE
rs4138134	A	G	A	G	0.029528	0.106789	0.036862	0.042083	FALSE	FALSE	FALSE	I9_PULMEN	0.003223	0.036253	pulmonary	TRUE	reported	textfile	9	1.31E+08	0.005259	2.02E-08	478116	monocyte	TRUE	reported	igd	6.59E-05	31.52535	2	TRUE
rs4140954	A	G	A	G	-0.09649	0.015827	0.033848	0.025941	FALSE	FALSE	FALSE	I9_PULMEN	0.739367	0.047573	pulmonary	TRUE	reported	textfile	22	17579495	0.005685	1.49E-64	520655	monocyte	TRUE	reported	igd	0.000553	288.0905	2	TRUE
rs4142441	G	A	G	A	0.031012	0.017269	0.151573	0.153506	FALSE	FALSE	FALSE	I9_PULMEN	0.4074	0.020844	pulmonary	TRUE	reported	textfile	20	42839620	0.002648	1.22E-31	520187	monocyte	TRUE	reported	igd	0.000264	137.1582	2	TRUE
rs4143044	G	C	G	C	-0.03001	-0.0006	0.066381	0.106348	FALSE	TRUE	FALSE	I9_PULMEN	0.98034	0.024441	pulmonary	TRUE	reported	textfile	7	50798525	0.00377	1.82E-15	521592	monocyte	TRUE	reported	igd	0.000121	63.35207	2	TRUE
rs4269828	G	A	G	A	0.012209	-0.02138	0.560056	0.584478	FALSE	FALSE	FALSE	I9_PULMEN	0.163793	0.015357	pulmonary	TRUE	reported	textfile	1	2.35E+08	0.001915	1.89E-10	521591	monocyte	TRUE	reported	igd	7.79E-05	40.6463	2	TRUE
rs4335411	A	G	A	G	-0.01741	0.018664	0.762473	0.752302	FALSE	FALSE	FALSE	I9_PULMEN	0.293293	0.017759	pulmonary	TRUE	reported	textfile	1	2.49E+08	0.002412	5.51E-13	499223	monocyte	TRUE	reported	igd	0.000104	52.11837	2	TRUE
rs4365101	A	G	A	G	-0.02677	-0.00126	0.068386	0.081953	FALSE	FALSE	FALSE	I9_PULMEN	0.962975	0.027235	pulmonary	TRUE	reported	textfile	12	1.09E+08	0.003761	1.14E-12	521590	monocyte	TRUE	reported	igd	9.71E-05	50.66649	2	TRUE

rs4385425	G	A	G	A	-0.0601	-0.02033	0.323914	0.30151	FALSE	FALSE	FALSE	I9_PULMEN	0.211526	0.016271	pulmonary	TRUE	reported	textfile	7	50307334	0.001999	########	521593	monocyte	TRUE	reported	igd	0.00173	904.023	2	TRUE
rs4411554	G	C	G	C	0.012567	-0.01089	0.714191	0.663815	FALSE	TRUE	FALSE	I9_PULMEN	0.492369	0.015861	pulmonary	TRUE	reported	textfile	17	1319765	0.002108	2.58E-09	521590	monocyte	TRUE	reported	igd	6.81E-05	35.54024	2	TRUE
rs445611	G	A	G	A	0.016004	0.037929	0.844726	0.866851	FALSE	FALSE	FALSE	I9_PULMEN	0.086523	0.022128	pulmonary	TRUE	reported	textfile	5	35396119	0.002607	8.58E-10	521594	monocyte	TRUE	reported	igd	7.22E-05	37.68542	2	TRUE
rs4462758	A	G	A	G	-0.0329	-0.01901	0.750205	0.765419	FALSE	FALSE	FALSE	I9_PULMEN	0.284015	0.017743	pulmonary	TRUE	reported	textfile	2	1.82E+08	0.002155	1.43E-52	519112	monocyte	TRUE	reported	igd	0.000449	233.1174	2	TRUE
rs4557713	G	A	G	A	-0.06461	-0.02532	0.043857	0.071722	FALSE	FALSE	FALSE	I9_PULMEN	0.390064	0.029461	pulmonary	TRUE	reported	textfile	8	48651633	0.004959	9.05E-39	514341	monocyte	TRUE	reported	igd	0.00033	169.7709	2	TRUE
rs4566648	G	T	G	T	-0.0254	0.008013	0.342027	0.256308	FALSE	FALSE	FALSE	I9_PULMEN	0.640687	0.017169	pulmonary	TRUE	reported	textfile	4	84159219	0.001975	8.14E-38	521587	monocyte	TRUE	reported	igd	0.000317	165.3464	2	TRUE
rs4577206	T	C	T	C	-0.02077	0.005487	0.88902	0.864325	FALSE	FALSE	FALSE	I9_PULMEN	0.803613	0.022066	pulmonary	TRUE	reported	textfile	19	47545719	0.003177	6.50E-11	475639	monocyte	TRUE	reported	igd	8.98E-05	42.71964	2	TRUE
rs458699	A	T	A	T	0.013577	0.006796	0.757042	0.770925	FALSE	TRUE	FALSE	I9_PULMEN	0.704061	0.01789	pulmonary	TRUE	reported	textfile	1	1.13E+08	0.002176	4.52E-10	521594	monocyte	TRUE	reported	igd	7.46E-05	38.93034	2	TRUE
rs4626924	T	C	T	C	-0.01835	-0.00161	0.551005	0.643478	FALSE	FALSE	FALSE	I9_PULMEN	0.918635	0.015728	pulmonary	TRUE	reported	textfile	1	2.35E+08	0.00191	8.20E-22	520181	monocyte	TRUE	reported	igd	0.000177	92.27025	2	TRUE
rs4658231	G	A	G	A	-0.016	-0.00907	0.24867	0.282544	FALSE	FALSE	FALSE	I9_PULMEN	0.586977	0.016702	pulmonary	TRUE	reported	textfile	1	92041502	0.002181	2.23E-13	520190	monocyte	TRUE	reported	igd	0.000104	53.85157	2	TRUE
rs4669869	C	T	C	T	0.020401	-0.0018	0.44511	0.533668	FALSE	FALSE	FALSE	I9_PULMEN	0.90497	0.015051	pulmonary	TRUE	reported	textfile	2	12898460	0.001889	3.76E-27	517821	monocyte	TRUE	reported	igd	0.000225	116.6373	2	TRUE
rs4712652	A	G	A	G	0.015034	-0.03582	0.579923	0.682755	FALSE	FALSE	FALSE	I9_PULMEN	0.026373	0.016129	pulmonary	TRUE	reported	textfile	6	22078615	0.001898	2.49E-15	521590	monocyte	TRUE	reported	igd	0.00012	62.74152	2	TRUE
rs4721764	A	G	A	G	0.015617	-0.01741	0.209536	0.242548	FALSE	FALSE	FALSE	I9_PULMEN	0.320644	0.017532	pulmonary	TRUE	reported	textfile	7	2578455	0.00233	2.12E-11	521587	monocyte	TRUE	reported	igd	8.61E-05	44.92434	2	TRUE
rs47341	T	C	T	C	0.031081	0.000456	0.396	0.370223	FALSE	FALSE	FALSE	I9_PULMEN	0.976585	0.01553	pulmonary	TRUE	reported	textfile	22	43560763	0.001997	1.46E-54	476098	monocyte	TRUE	reported	igd	0.000509	242.2323	2	TRUE
rs4737009	A	G	A	G	0.015384	-0.00995	0.235763	0.249953	FALSE	FALSE	FALSE	I9_PULMEN	0.568146	0.017425	pulmonary	TRUE	reported	textfile	8	41630405	0.002209	3.44E-12	520188	monocyte	TRUE	reported	igd	9.32E-05	48.50041	2	TRUE
rs475616	G	A	G	A	0.01572	-0.03351	0.65632	0.724505	FALSE	FALSE	FALSE	I9_PULMEN	0.046054	0.016798	pulmonary	TRUE	reported	textfile	10	30496905	0.00199	2.97E-15	520514	monocyte	TRUE	reported	igd	0.00012	62.40182	2	TRUE
rs4763944	A	G	A	G	0.013087	-0.00251	0.204423	0.267177	FALSE	FALSE	FALSE	I9_PULMEN	0.882143	0.016926	pulmonary	TRUE	reported	textfile	12	13357972	0.002322	1.77E-08	520511	monocyte	TRUE	reported	igd	6.10E-05	31.76538	2	TRUE
rs4810832	A	G	A	G	0.012663	-0.04145	0.325034	0.261667	FALSE	FALSE	FALSE	I9_PULMEN	0.015314	0.017092	pulmonary	TRUE	reported	textfile	20	47226433	0.002027	4.34E-10	520653	monocyte	TRUE	reported	igd	7.50E-05	39.0269	2	TRUE
rs4813619	T	G	T	G	0.016505	0.002048	0.510832	0.556477	FALSE	FALSE	FALSE	I9_PULMEN	0.892341	0.015129	pulmonary	TRUE	reported	textfile	20	2815715	0.001902	4.24E-18	520190	monocyte	TRUE	reported	igd	0.000145	75.30232	2	TRUE
rs4865956	A	T	A	T	-0.01892	0.024668	0.696281	0.719773	FALSE	TRUE	FALSE	I9_PULMEN	0.14095	0.016755	pulmonary	TRUE	reported	textfile	5	54882505	0.002041	1.97E-20	521588	monocyte	TRUE	reported	igd	0.000165	85.90469	2	TRUE
rs4871844	C	T	C	T	0.015503	-0.01333	0.343089	0.36735	FALSE	FALSE	FALSE	I9_PULMEN	0.392954	0.015607	pulmonary	TRUE	reported	textfile	8	22879734	0.00201	1.27E-14	521589	monocyte	TRUE	reported	igd	0.000114	59.48914	2	TRUE
rs4886615	G	A	G	A	-0.01896	-0.00345	0.724425	0.690943	FALSE	FALSE	FALSE	I9_PULMEN	0.831985	0.016252	pulmonary	TRUE	reported	textfile	15	75131661	0.00212	4.10E-19	520190	monocyte	TRUE	reported	igd	0.000154	79.95872	2	TRUE
rs4905043	A	G	A	G	0.018703	0.016606	0.399537	0.387294	FALSE	FALSE	FALSE	I9_PULMEN	0.284289	0.015509	pulmonary	TRUE	reported	textfile	14	93550009	0.002008	1.31E-20	475634	monocyte	TRUE	reported	igd	0.000182	86.75476	2	TRUE
rs4907230	A	G	A	G	0.015584	-0.02599	0.323539	0.470685	FALSE	FALSE	FALSE	I9_PULMEN	0.084138	0.015049	pulmonary	TRUE	reported	textfile	2	96855241	0.002002	7.24E-15	520502	monocyte	TRUE	reported	igd	0.000116	60.59378	2	TRUE
rs4952782	A	T	A	T	0.019255	-0.00353	0.759932	0.684006	FALSE	TRUE	FALSE	I9_PULMEN	0.826859	0.016144	pulmonary	TRUE	reported	textfile	2	46074096	0.002182	1.17E-18	521594	monocyte	TRUE	reported	igd	0.000149	77.87102	2	TRUE
rs4970966	T	G	T	G	-0.07264	0.050928	0.222433	0.208462	FALSE	FALSE	FALSE	I9_PULMEN	0.00554	0.01836	pulmonary	TRUE	reported	textfile	1	1.51E+08	0.002252	########	520192	monocyte	TRUE	reported	igd	0.001996	1040.374	2	TRUE
rs4983387	A	G	A	G	0.019449	-0.03684	0.899135	0.894235	FALSE	FALSE	FALSE	I9_PULMEN	0.133053	0.024522	pulmonary	TRUE	reported	textfile	14	1.05E+08	0.003249	2.20E-09	476100	monocyte	TRUE	reported	igd	7.53E-05	35.83384	2	TRUE
rs4987353	A	G	A	G	-0.02189	0.011609	0.310381	0.255635	FALSE	FALSE	FALSE	I9_PULMEN	0.49932	0.017185	pulmonary	TRUE	reported	textfile	1	1.7E+08	0.002015	1.93E-27	521592	monocyte	TRUE	reported	igd	0.000226	117.9726	2	TRUE
rs5568423	A	G	A	G	0.01153	0.009695	0.348464	0.357414	FALSE	FALSE	FALSE	I9_PULMEN	0.536153	0.015671	pulmonary	TRUE	reported	textfile	1	44080009	0.001956	3.89E-09	521590	monocyte	TRUE	reported	igd	6.66E-05	34.74715	2	TRUE
rs5586032	A	G	A	G	-0.01367	0.000187	0.682365	0.623156	FALSE	FALSE	FALSE	I9_PULMEN	0.990377	0.01554	pulmonary	TRUE	reported	textfile	13	1.15E+08	0.002053	2.88E-11	520193	monocyte	TRUE	reported	igd	8.52E-05	44.3361	2	TRUE
rs5600779	T	A	T	A	0.039085	0.021305	0.24434	0.257979	FALSE	TRUE	FALSE	I9_PULMEN	0.21428	0.017155	pulmonary	TRUE	reported	textfile	6	41990827	0.002177	4.77E-72	521592	monocyte	TRUE	reported	igd	0.000618	322.3307	2	TRUE
rs5605842	G	A	G	A	0.01173	0.00727	0.356231	0.234778	FALSE	FALSE	FALSE	I9_PULMEN	0.683145	0.017811	pulmonary	TRUE	reported	textfile	4	1.85E+08	0.001975	2.96E-09	519573	monocyte	TRUE	reported	igd	6.79E-05	35.27444	2	TRUE
rs5634489	A	C	A	C	-0.04434	-0.02358	0.372987	0.344436	FALSE	FALSE	FALSE	I9_PULMEN	0.136206	0.015828	pulmonary	TRUE	reported	textfile	19	44282529	0.00195	########	521577	monocyte	TRUE	reported	igd	0.00099	516.9657	2	TRUE
rs5679560	T	A	T	A	-0.01493	0.037212	0.17201	0.132286	FALSE	TRUE	FALSE	I9_PULMEN	0.092085	0.022091	pulmonary	TRUE	reported	textfile	1	1.11E+08	0.002483	1.87E-09	520513	monocyte	TRUE	reported	igd	6.95E-05	36.15468	2	TRUE
rs5692590	G	C	G	C	0.01367	0.01	0.218542	0.208745	FALSE	TRUE	FALSE	I9_PULMEN	0.587945	0.018456	pulmonary	TRUE	reported	textfile	3	1.87E+08	0.002253	1.33E-09	521592	monocyte	TRUE	reported	igd	7.06E-05	36.814	2	TRUE
rs571497	A	G	A	G	-0.02817	0.035427	0.154612	0.124651	FALSE	FALSE	FALSE	I9_PULMEN	0.119376	0.022748	pulmonary	TRUE	reported	textfile	19	7827830	0.002628	8.94E-27	519115	monocyte	TRUE	reported	igd	0.000221	114.933	2	TRUE
rs5722139	G	T	G	T	-0.02234	-0.03214	0.07144	0.0683	FALSE	FALSE	FALSE	I9_PULMEN	0.280321	0.029775	pulmonary	TRUE	reported	textfile	21	16583936	0.003693	1.51E-09	520653	monocyte	TRUE	reported	igd	7.03E-05	36.58385	2	TRUE
rs573790	C	T	C	T	-0.01609	0.027288	0.613652	0.469994	FALSE	FALSE	FALSE	I9_PULMEN	0.070669	0.015096	pulmonary	TRUE	reported	textfile	11	59855385	0.001947	1.48E-16	520191	monocyte	TRUE	reported	igd	0.000131	68.31883	2	TRUE
rs5801182	C	A	C	A	0.018373	0.001227	0.137161	0.185522	FALSE	FALSE	FALSE	I9_PULMEN	0.949412	0.019342	pulmonary	TRUE	reported	textfile	3	1.23E+08	0.002728	1.69E-11	520650	monocyte	TRUE	reported	igd	8.71E-05	45.35966	2	TRUE
rs5881415	G	T	G	T	-0.02351	-0.00845	0.15736	0.116486	FALSE	FALSE	FALSE	I9_PULMEN	0.717198	0.023327	pulmonary	TRUE	reported	textfile	13	1.11E+08	0.002622	3.24E-19	521593	monocyte	TRUE	reported	igd	0.000154	80.41038	2	TRUE
rs5929568	T	C	T	C	-0.02004	-0.02342	0.173015	0.191654	FALSE	FALSE	FALSE	I9_PULMEN	0.218907	0.019053	pulmonary	TRUE	reported	textfile	2	1.37E+08	0.002487	7.99E-16	520653	monocyte	TRUE	reported	igd	0.000125	64.94896	2	TRUE
rs6044081	G	A	G	A	0.012275	0.035589	0.758074	0.79612	FALSE	FALSE	FALSE	I9_PULMEN	0.056078	0.018629	pulmonary	TRUE	reported	textfile	20	16513342	0.002186	2.00E-08	520193	monocyte	TRUE	reported	igd	6.06E-05	31.53124	2	TRUE
rs6055955	T	C	T	C	-0.02899	-0.00551	0.508474	0.485191	FALSE	FALSE	FALSE	I9_PULMEN	0.713587	0.015001	pulmonary	TRUE	reported	textfile	20	8604181	0.001876	8.72E-54	520194	monocyte	TRUE	reported	igd	0.000459	238.7312	2	TRUE
rs6069534	T	C	T	C	-0.01816	0.009234	0.197081	0.215851	FALSE	FALSE	FALSE	I9_PULMEN	0.612439	0.018228	pulmonary	TRUE	reported	textfile	15	51010271	0.00235	1.14E-14	521560	monocyte	TRUE	reported	igd	0.000115	59.72965	2	TRUE

rs6069990	C	T	C	T	0.021517	0.033316	0.118119	0.127308	FALSE	FALSE	FALSE	I9_PULMEN	0.139253	0.022533	pulmonary	TRUE	reported	textfile	13	92009193	0.002926	2.02E-13	521593	monocyte	TRUE	reported	igd	0.000104	54.07707	2	TRUE
rs6116564	G	A	G	A	-0.0264	0.033113	0.14078	0.210367	FALSE	FALSE	FALSE	I9_PULMEN	0.072684	0.01845	pulmonary	TRUE	reported	textfile	1	2.44E+08	0.002716	2.59E-22	521592	monocyte	TRUE	reported	igd	0.000181	94.49579	2	TRUE
rs6120950	T	C	T	C	-0.01936	0.011939	0.202811	0.266939	FALSE	FALSE	FALSE	I9_PULMEN	0.481478	0.016961	pulmonary	TRUE	reported	textfile	20	30225070	0.002341	1.45E-16	521589	monocyte	TRUE	reported	igd	0.000131	68.35682	2	TRUE
rs6198554	A	G	A	G	-0.01149	-0.01751	0.495161	0.539496	FALSE	FALSE	FALSE	I9_PULMEN	0.244247	0.015036	pulmonary	TRUE	reported	textfile	14	60040825	0.001887	1.18E-09	520511	monocyte	TRUE	reported	igd	7.12E-05	37.07615	2	TRUE
rs6201133	G	A	G	A	0.011932	-0.00765	0.355185	0.264691	FALSE	FALSE	FALSE	I9_PULMEN	0.652988	0.017022	pulmonary	TRUE	reported	textfile	15	63837491	0.00199	2.10E-09	521593	monocyte	TRUE	reported	igd	6.89E-05	35.95164	2	TRUE
rs6201815	A	G	A	G	0.015004	-0.00331	0.209149	0.188014	FALSE	FALSE	FALSE	I9_PULMEN	0.863393	0.019235	pulmonary	TRUE	reported	textfile	15	40397421	0.002338	1.42E-10	519573	monocyte	TRUE	reported	igd	7.93E-05	41.18352	2	TRUE
rs6205480	T	C	T	C	0.020073	0.024691	0.294764	0.189307	FALSE	FALSE	FALSE	I9_PULMEN	0.196943	0.019136	pulmonary	TRUE	reported	textfile	17	43915497	0.002064	2.45E-22	521589	monocyte	TRUE	reported	igd	0.000181	94.58092	2	TRUE
rs6205779	A	G	A	G	-0.01948	-0.04075	0.111608	0.114883	FALSE	FALSE	FALSE	I9_PULMEN	0.084213	0.023597	pulmonary	TRUE	reported	textfile	17	25856325	0.002995	8.00E-11	521588	monocyte	TRUE	reported	igd	8.11E-05	42.32149	2	TRUE
rs6214320	A	G	A	G	-0.05757	0.016697	0.104321	0.102301	FALSE	FALSE	FALSE	I9_PULMEN	0.504285	0.025004	pulmonary	TRUE	reported	textfile	19	54325353	0.003245	2.18E-70	516125	monocyte	TRUE	reported	igd	0.00061	314.7906	2	TRUE
rs6217678	G	A	G	A	0.02263	-0.00444	0.319489	0.3936	FALSE	FALSE	FALSE	I9_PULMEN	0.773056	0.015399	pulmonary	TRUE	reported	textfile	2	1.7E+08	0.002002	1.36E-29	519574	monocyte	TRUE	reported	igd	0.000246	127.7731	2	TRUE
rs6226197	G	A	G	A	-0.01657	0.00131	0.183464	0.148574	FALSE	FALSE	FALSE	I9_PULMEN	0.950523	0.021107	pulmonary	TRUE	reported	textfile	3	1.07E+08	0.002414	7.01E-12	521592	monocyte	TRUE	reported	igd	9.03E-05	47.11036	2	TRUE
rs6245442	G	A	G	A	-0.03489	0.005862	0.070245	0.098379	FALSE	FALSE	FALSE	I9_PULMEN	0.8167	0.025291	pulmonary	TRUE	reported	textfile	7	27191804	0.003675	2.39E-21	521578	monocyte	TRUE	reported	igd	0.000173	90.10753	2	TRUE
rs6247067	G	A	G	A	-0.02333	-0.00962	0.092471	0.135479	FALSE	FALSE	FALSE	I9_PULMEN	0.659869	0.021853	pulmonary	TRUE	reported	textfile	7	1.15E+08	0.00323	5.30E-13	521594	monocyte	TRUE	reported	igd	0.0001	52.18365	2	TRUE
rs6249192	A	C	A	C	-0.01299	0.014934	0.256754	0.205783	FALSE	FALSE	FALSE	I9_PULMEN	0.422955	0.018637	pulmonary	TRUE	reported	textfile	7	1.49E+08	0.002145	1.42E-09	521586	monocyte	TRUE	reported	igd	7.03E-05	36.69126	2	TRUE
rs6250113	A	G	A	G	0.020496	0.045812	0.221444	0.229464	FALSE	FALSE	FALSE	I9_PULMEN	0.011163	0.018053	pulmonary	TRUE	reported	textfile	8	23089429	0.002268	1.72E-19	521571	monocyte	TRUE	reported	igd	0.000157	81.66773	2	TRUE
rs6250239	A	G	A	G	-0.01791	0.02523	0.143097	0.147207	FALSE	FALSE	FALSE	I9_PULMEN	0.238455	0.021402	pulmonary	TRUE	reported	textfile	8	27190945	0.00267	2.08E-11	520653	monocyte	TRUE	reported	igd	8.64E-05	44.98021	2	TRUE
rs6251334	T	C	T	C	-0.0151	0.027979	0.142064	0.104906	FALSE	FALSE	FALSE	I9_PULMEN	0.256564	0.024661	pulmonary	TRUE	reported	textfile	8	1.17E+08	0.002695	2.17E-08	520504	monocyte	TRUE	reported	igd	6.03E-05	31.38066	2	TRUE
rs628615	C	T	C	T	-0.01697	-0.06989	0.112511	0.131075	FALSE	FALSE	FALSE	I9_PULMEN	0.002007	0.022625	pulmonary	TRUE	reported	textfile	1	23685408	0.002951	9.16E-09	521592	monocyte	TRUE	reported	igd	6.34E-05	33.0653	2	TRUE
rs628965	G	A	G	A	-0.01265	-0.00907	0.630893	0.624154	FALSE	FALSE	FALSE	I9_PULMEN	0.558632	0.015514	pulmonary	TRUE	reported	textfile	20	3649713	0.001982	1.84E-10	520651	monocyte	TRUE	reported	igd	7.82E-05	40.70321	2	TRUE
rs6429432	C	A	C	A	-0.07965	0.029165	0.891513	0.91421	FALSE	FALSE	FALSE	I9_PULMEN	0.276827	0.026819	pulmonary	TRUE	reported	textfile	1	2.36E+08	0.002993	########	521593	monocyte	TRUE	reported	igd	0.001356	708.2364	2	TRUE
rs6429438	G	A	G	A	0.02083	-0.05745	0.886382	0.862017	FALSE	FALSE	FALSE	I9_PULMEN	0.007341	0.021428	pulmonary	TRUE	reported	textfile	1	2.44E+08	0.002957	1.93E-12	521593	monocyte	TRUE	reported	igd	9.51E-05	49.622	2	TRUE
rs6434817	G	A	G	A	0.0116	0.024632	0.718131	0.734299	FALSE	FALSE	FALSE	I9_PULMEN	0.14702	0.016986	pulmonary	TRUE	reported	textfile	2	1.97E+08	0.002071	2.17E-08	520195	monocyte	TRUE	reported	igd	6.03E-05	31.37286	2	TRUE
rs644492	G	A	G	A	-0.02007	0.012565	0.195876	0.139672	FALSE	FALSE	FALSE	I9_PULMEN	0.563459	0.02175	pulmonary	TRUE	reported	textfile	6	53444319	0.002358	1.84E-17	521592	monocyte	TRUE	reported	igd	0.000139	72.43729	2	TRUE
rs6470759	A	G	A	G	-0.01819	-0.00196	0.588502	0.637798	FALSE	FALSE	FALSE	I9_PULMEN	0.900424	0.015689	pulmonary	TRUE	reported	textfile	8	1.31E+08	0.001932	5.04E-21	519111	monocyte	TRUE	reported	igd	0.000171	88.68302	2	TRUE
rs6493575	T	C	T	C	-0.01218	-0.01458	0.395673	0.361194	FALSE	FALSE	FALSE	I9_PULMEN	0.350289	0.015614	pulmonary	TRUE	reported	textfile	15	53013475	0.00191	1.86E-10	521587	monocyte	TRUE	reported	igd	7.80E-05	40.66551	2	TRUE
rs6512627	A	G	A	G	-0.04744	0.004463	0.19363	0.160617	FALSE	FALSE	FALSE	I9_PULMEN	0.827551	0.020488	pulmonary	TRUE	reported	textfile	20	48967591	0.002378	1.83E-88	521592	monocyte	TRUE	reported	igd	0.000762	397.949	2	TRUE
rs6545873	C	T	C	T	-0.01534	0.028666	0.610495	0.610502	FALSE	FALSE	FALSE	I9_PULMEN	0.062763	0.015405	pulmonary	TRUE	reported	textfile	2	61733012	0.001915	1.18E-15	521593	monocyte	TRUE	reported	igd	0.000123	64.19207	2	TRUE
rs6579771	T	C	T	C	0.033644	0.010793	0.268553	0.232793	FALSE	FALSE	FALSE	I9_PULMEN	0.544802	0.017823	pulmonary	TRUE	reported	textfile	5	1.49E+08	0.002115	6.13E-57	521591	monocyte	TRUE	reported	igd	0.000485	253.0421	2	TRUE
rs6581124	A	G	A	G	-0.01277	-0.0248	0.24468	0.28169	FALSE	FALSE	FALSE	I9_PULMEN	0.136995	0.016674	pulmonary	TRUE	reported	textfile	12	57545756	0.002214	8.40E-09	520647	monocyte	TRUE	reported	igd	6.38E-05	33.24182	2	TRUE
rs6591578	A	G	A	G	0.021869	-0.01462	0.633592	0.631314	FALSE	FALSE	FALSE	I9_PULMEN	0.346832	0.015542	pulmonary	TRUE	reported	textfile	11	60158649	0.001941	2.03E-29	521590	monocyte	TRUE	reported	igd	0.000243	126.9419	2	TRUE
rs662333	A	G	A	G	-0.01382	-0.00369	0.779527	0.784439	FALSE	FALSE	FALSE	I9_PULMEN	0.839906	0.018275	pulmonary	TRUE	reported	textfile	11	1.22E+08	0.002263	1.05E-09	519113	monocyte	TRUE	reported	igd	7.18E-05	37.29454	2	TRUE
rs663202	C	G	C	G	-0.01507	0.009603	0.170566	0.24868	FALSE	TRUE	FALSE	I9_PULMEN	0.580916	0.017396	pulmonary	TRUE	reported	textfile	3	1.24E+08	0.002485	1.39E-09	521591	monocyte	TRUE	reported	igd	7.05E-05	36.75224	2	TRUE
rs6664626	T	G	T	G	0.018875	-0.04754	0.142992	0.137767	FALSE	FALSE	FALSE	I9_PULMEN	0.02912	0.021789	pulmonary	TRUE	reported	textfile	1	66128239	0.002862	4.38E-11	478108	monocyte	TRUE	reported	igd	9.10E-05	43.49435	2	TRUE
rs6687430	A	G	A	G	0.011892	-0.00075	0.54337	0.51749	FALSE	FALSE	FALSE	I9_PULMEN	0.960227	0.015072	pulmonary	TRUE	reported	textfile	1	10633245	0.001876	2.41E-10	521593	monocyte	TRUE	reported	igd	7.70E-05	40.18301	2	TRUE
rs6696074	T	C	T	C	0.018322	0.022047	0.562926	0.594196	FALSE	FALSE	FALSE	I9_PULMEN	0.15047	0.015333	pulmonary	TRUE	reported	textfile	1	2.26E+08	0.00192	1.49E-21	520513	monocyte	TRUE	reported	igd	0.000175	91.06293	2	TRUE
rs6722495	C	T	C	T	0.026909	0.039293	0.170315	0.15627	FALSE	FALSE	FALSE	I9_PULMEN	0.058055	0.020732	pulmonary	TRUE	reported	textfile	1	1.18E+08	0.002479	2.01E-27	520651	monocyte	TRUE	reported	igd	0.000226	117.8258	2	TRUE
rs6736362	T	C	T	C	0.022174	0.016292	0.559146	0.669526	FALSE	FALSE	FALSE	I9_PULMEN	0.307503	0.015965	pulmonary	TRUE	reported	textfile	2	2.19E+08	0.001885	6.45E-32	520648	monocyte	TRUE	reported	igd	0.000266	138.3769	2	TRUE
rs6740847	G	A	G	A	0.127269	-0.01951	0.562078	0.59925	FALSE	FALSE	FALSE	I9_PULMEN	0.202405	0.015302	pulmonary	TRUE	reported	textfile	2	1.82E+08	0.001885	########	520195	monocyte	TRUE	reported	igd	0.008687	4558.489	2	TRUE
rs6745920	C	T	C	T	0.012709	-0.0142	0.619377	0.688847	FALSE	FALSE	FALSE	I9_PULMEN	0.388538	0.016465	pulmonary	TRUE	reported	textfile	2	1.02E+08	0.001997	2.02E-10	520194	monocyte	TRUE	reported	igd	7.79E-05	40.50093	2	TRUE
rs6753534	T	C	T	C	0.012908	0.011998	0.562248	0.601317	FALSE	FALSE	FALSE	I9_PULMEN	0.434736	0.015361	pulmonary	TRUE	reported	textfile	2	27752871	0.001889	8.58E-12	521587	monocyte	TRUE	reported	igd	8.95E-05	46.69306	2	TRUE
rs6772164	A	C	A	C	-0.01154	0.000296	0.358647	0.303956	FALSE	FALSE	FALSE	I9_PULMEN	0.985532	0.01633	pulmonary	TRUE	reported	textfile	3	1.96E+08	0.002024	1.21E-08	475812	monocyte	TRUE	reported	igd	6.84E-05	32.52479	2	TRUE
rs6796	C	T	C	T	0.045504	0.012312	0.274261	0.320664	FALSE	FALSE	FALSE	I9_PULMEN	0.446639	0.016178	pulmonary	TRUE	reported	textfile	7	6502367	0.002116	########	519575	monocyte	TRUE	reported	igd	0.000889	462.4515	2	TRUE
rs6799804	A	T	A	T	-0.02742	-0.02198	0.075378	0.130826	FALSE	TRUE	FALSE	I9_PULMEN	0.326135	0.022386	pulmonary	TRUE	reported	textfile	3	46820833	0.003565	1.52E-14	520180	monocyte	TRUE	reported	igd	0.000114	59.14515	2	TRUE
rs6800122	T	C	T	C	-0.02178	-0.00718	0.399684	0.365604	FALSE	FALSE	FALSE	I9_PULMEN	0.644025	0.015549	pulmonary	TRUE	reported	textfile	3	1.41E+08	0.001911	4.75E-30	520649	monocyte	TRUE	reported	igd	0.000249	129.8354	2	TRUE

rs6856799	C	T	C	T	-0.0111	0.011257	0.315049	0.283404	FALSE	FALSE	FALSE	I9_PULMEN	0.499582	0.016673	pulmonary	TRUE	reported	textfile	4	38081983	0.002017	3.81E-08	521591	monocyte	TRUE	reported	igd	5.81E-05	30.2908	2	TRUE
rs6869021	T	C	T	C	-0.01691	0.001147	0.313259	0.287691	FALSE	FALSE	FALSE	I9_PULMEN	0.944775	0.016561	pulmonary	TRUE	reported	textfile	5	1.11E+08	0.002017	5.47E-17	521587	monocyte	TRUE	reported	igd	0.000135	70.24524	2	TRUE
rs6883116	C	T	C	T	-0.01799	0.015876	0.417349	0.443297	FALSE	FALSE	FALSE	I9_PULMEN	0.294473	0.015144	pulmonary	TRUE	reported	textfile	5	1.79E+08	0.001921	8.21E-21	521586	monocyte	TRUE	reported	igd	0.000168	87.65254	2	TRUE
rs695113	T	C	T	C	0.021098	-0.03519	0.696592	0.659122	FALSE	FALSE	FALSE	I9_PULMEN	0.026662	0.015879	pulmonary	TRUE	reported	textfile	11	1.29E+08	0.002054	1.04E-24	520190	monocyte	TRUE	reported	igd	0.000203	105.5067	2	TRUE
rs701905	C	G	C	G	0.021672	0.013526	0.352317	0.214159	FALSE	TRUE	FALSE	I9_PULMEN	0.459357	0.018281	pulmonary	TRUE	reported	textfile	1	2.13E+08	0.001955	1.63E-28	521591	monocyte	TRUE	reported	igd	0.000236	122.8861	2	TRUE
rs7030655	T	G	T	G	0.020088	0.00167	0.308549	0.274749	FALSE	FALSE	FALSE	I9_PULMEN	0.920853	0.016812	pulmonary	TRUE	reported	textfile	9	1.14E+08	0.002019	2.66E-23	521590	monocyte	TRUE	reported	igd	0.00019	98.99177	2	TRUE
rs706819	C	T	C	T	0.018421	-0.00291	0.738253	0.745733	FALSE	FALSE	FALSE	I9_PULMEN	0.866315	0.017311	pulmonary	TRUE	reported	textfile	12	52315923	0.002154	1.29E-17	520195	monocyte	TRUE	reported	igd	0.000141	73.13636	2	TRUE
rs707793	C	T	C	T	-0.03032	0.006993	0.531284	0.522505	FALSE	FALSE	FALSE	I9_PULMEN	0.642479	0.015064	pulmonary	TRUE	reported	textfile	6	10520009	0.001881	2.13E-58	521503	monocyte	TRUE	reported	igd	0.000498	259.8753	2	TRUE
rs7095778	G	A	G	A	-0.02041	-0.01611	0.091164	0.065053	FALSE	FALSE	FALSE	I9_PULMEN	0.597863	0.030545	pulmonary	TRUE	reported	textfile	10	1.12E+08	0.003252	3.56E-10	521589	monocyte	TRUE	reported	igd	7.55E-05	39.39747	2	TRUE
rs7097656	C	T	C	T	0.020177	0.005403	0.794776	0.778719	FALSE	FALSE	FALSE	I9_PULMEN	0.765417	0.018108	pulmonary	TRUE	reported	textfile	10	82250831	0.002312	2.80E-18	520188	monocyte	TRUE	reported	igd	0.000146	76.16156	2	TRUE
rs7111769	C	A	C	A	-0.02916	0.040787	0.074664	0.022932	FALSE	FALSE	FALSE	I9_PULMEN	0.42169	0.050762	pulmonary	TRUE	reported	textfile	11	88023721	0.003669	1.99E-15	521589	monocyte	TRUE	reported	igd	0.000121	63.17388	2	TRUE
rs7133161	A	C	A	C	-0.04978	-0.04048	0.036301	0.053854	FALSE	FALSE	FALSE	I9_PULMEN	0.227666	0.033552	pulmonary	TRUE	reported	textfile	3	1.29E+08	0.005022	3.98E-23	520653	monocyte	TRUE	reported	igd	0.000189	98.23527	2	TRUE
rs7136811	C	T	C	T	-0.02359	0.019631	0.086908	0.069603	FALSE	FALSE	FALSE	I9_PULMEN	0.507418	0.029615	pulmonary	TRUE	reported	textfile	17	27329227	0.003497	1.58E-11	521548	monocyte	TRUE	reported	igd	8.72E-05	45.48997	2	TRUE
rs7180079	G	A	G	A	0.059435	0.004956	0.876937	0.877447	FALSE	FALSE	FALSE	I9_PULMEN	0.828224	0.022843	pulmonary	TRUE	reported	textfile	15	64629873	0.00287	3.41E-95	521593	monocyte	TRUE	reported	igd	0.000822	428.8635	2	TRUE
rs7180804	A	G	A	G	-0.07744	0.010161	0.256783	0.222636	FALSE	FALSE	FALSE	I9_PULMEN	0.573536	0.018053	pulmonary	TRUE	reported	textfile	15	80259074	0.002141	########	521587	monocyte	TRUE	reported	igd	0.002502	1308.332	2	TRUE
rs7185007	T	C	T	C	-0.01672	0.039915	0.240169	0.270293	FALSE	FALSE	FALSE	I9_PULMEN	0.018017	0.016876	pulmonary	TRUE	reported	textfile	16	30927509	0.002191	2.40E-14	521587	monocyte	TRUE	reported	igd	0.000112	58.24224	2	TRUE
rs718515	A	G	A	G	-0.01994	-0.01343	0.552238	0.561605	FALSE	FALSE	FALSE	I9_PULMEN	0.375338	0.015144	pulmonary	TRUE	reported	textfile	18	43856297	0.001891	5.66E-26	521591	monocyte	TRUE	reported	igd	0.000213	111.2011	2	TRUE
rs7196129	C	T	C	T	0.025016	-0.0335	0.530279	0.585378	FALSE	FALSE	FALSE	I9_PULMEN	0.029231	0.015363	pulmonary	TRUE	reported	textfile	16	30471109	0.001905	2.45E-39	521573	monocyte	TRUE	reported	igd	0.000331	172.4424	2	TRUE
rs723585	G	A	G	A	-0.01654	0.017829	0.483425	0.46907	FALSE	FALSE	FALSE	I9_PULMEN	0.237315	0.015087	pulmonary	TRUE	reported	textfile	4	55503194	0.00187	9.43E-19	521592	monocyte	TRUE	reported	igd	0.00015	78.25123	2	TRUE
rs7249692	C	T	C	T	-0.01722	-0.01741	0.670676	0.773446	FALSE	FALSE	FALSE	I9_PULMEN	0.332206	0.01795	pulmonary	TRUE	reported	textfile	19	19670688	0.002004	8.83E-18	521593	monocyte	TRUE	reported	igd	0.000142	73.85333	2	TRUE
rs7267375	C	T	C	T	-0.01656	-0.02322	0.189986	0.142866	FALSE	FALSE	FALSE	I9_PULMEN	0.283386	0.021644	pulmonary	TRUE	reported	textfile	8	1.07E+08	0.002389	4.35E-12	521592	monocyte	TRUE	reported	igd	9.21E-05	48.02025	2	TRUE
rs7267557	T	C	T	C	0.015967	-0.00348	0.366495	0.306557	FALSE	FALSE	FALSE	I9_PULMEN	0.830431	0.016235	pulmonary	TRUE	reported	textfile	1	56636881	0.001937	1.76E-16	521592	monocyte	TRUE	reported	igd	0.00013	67.94942	2	TRUE
rs7272020	A	G	A	G	-0.01618	-0.02147	0.326922	0.394368	FALSE	FALSE	FALSE	I9_PULMEN	0.162384	0.015367	pulmonary	TRUE	reported	textfile	4	1.46E+08	0.001998	5.82E-16	521554	monocyte	TRUE	reported	igd	0.000126	65.60326	2	TRUE
rs7274379	A	G	A	G	0.040748	0.023466	0.030784	0.014149	FALSE	FALSE	FALSE	I9_PULMEN	0.708538	0.062774	pulmonary	TRUE	reported	textfile	15	80679076	0.005471	9.92E-14	521591	monocyte	TRUE	reported	igd	0.000106	55.47247	2	TRUE
rs7275926	T	C	T	C	0.021443	0.001355	0.158886	0.254151	FALSE	FALSE	FALSE	I9_PULMEN	0.938685	0.017612	pulmonary	TRUE	reported	textfile	9	1.27E+08	0.002644	5.36E-16	518163	monocyte	TRUE	reported	igd	0.000127	65.77283	2	TRUE
rs7279086	C	T	C	T	-0.02029	0.02938	0.307082	0.296648	FALSE	FALSE	FALSE	I9_PULMEN	0.073276	0.016403	pulmonary	TRUE	reported	textfile	10	44880260	0.002032	1.92E-23	521590	monocyte	TRUE	reported	igd	0.000191	99.69473	2	TRUE
rs7280513	A	G	A	G	0.029548	-0.04132	0.090934	0.07307	FALSE	FALSE	FALSE	I9_PULMEN	0.15559	0.029099	pulmonary	TRUE	reported	textfile	16	85858558	0.003399	3.70E-18	521589	monocyte	TRUE	reported	igd	0.000145	75.57048	2	TRUE
rs7283630	T	C	T	C	-0.04091	-0.0132	0.156407	0.122337	FALSE	FALSE	FALSE	I9_PULMEN	0.562793	0.022807	pulmonary	TRUE	reported	textfile	2	1.12E+08	0.002579	1.37E-56	520652	monocyte	TRUE	reported	igd	0.000483	251.5763	2	TRUE
rs7297040	G	A	G	A	-0.01853	0.013677	0.087376	0.066645	FALSE	FALSE	FALSE	I9_PULMEN	0.650366	0.030175	pulmonary	TRUE	reported	textfile	18	33119155	0.00335	3.24E-08	521594	monocyte	TRUE	reported	igd	5.87E-05	30.60555	2	TRUE
rs7298704	A	C	A	C	-0.02478	0.017738	0.124193	0.152571	FALSE	FALSE	FALSE	I9_PULMEN	0.395279	0.020866	pulmonary	TRUE	reported	textfile	19	3251116	0.002838	2.71E-18	521589	monocyte	TRUE	reported	igd	0.000146	76.22651	2	TRUE
rs7305859	C	G	C	G	0.037818	0.01858	0.217554	0.211241	FALSE	TRUE	FALSE	I9_PULMEN	0.315421	0.018507	pulmonary	TRUE	reported	textfile	3	39281374	0.002267	1.98E-62	521592	monocyte	TRUE	reported	igd	0.000533	278.2867	2	TRUE
rs7306708	C	T	C	T	-0.01572	-0.01161	0.160101	0.2972	FALSE	FALSE	FALSE	I9_PULMEN	0.481777	0.0165	pulmonary	TRUE	reported	textfile	12	21388829	0.002549	7.11E-10	521589	monocyte	TRUE	reported	igd	7.29E-05	38.03812	2	TRUE
rs7308348	C	T	C	T	0.015377	0.042834	0.182113	0.158251	FALSE	FALSE	FALSE	I9_PULMEN	0.037408	0.02058	pulmonary	TRUE	reported	textfile	12	1.22E+08	0.002468	4.78E-10	521590	monocyte	TRUE	reported	igd	7.44E-05	38.81962	2	TRUE
rs7314213	G	A	G	A	0.012783	-0.00813	0.399541	0.410209	FALSE	FALSE	FALSE	I9_PULMEN	0.594159	0.015262	pulmonary	TRUE	reported	textfile	7	65347962	0.001915	2.57E-11	519575	monocyte	TRUE	reported	igd	8.58E-05	44.55807	2	TRUE
rs731707	C	T	C	T	0.04713	-0.0152	0.489214	0.582381	FALSE	FALSE	FALSE	I9_PULMEN	0.319379	0.015266	pulmonary	TRUE	reported	textfile	16	85980968	0.001913	########	520192	monocyte	TRUE	reported	igd	0.001165	606.9645	2	TRUE
rs7320196	C	A	C	A	0.027169	0.017239	0.089234	0.09045	FALSE	FALSE	FALSE	I9_PULMEN	0.510231	0.026181	pulmonary	TRUE	reported	textfile	12	1.17E+08	0.003286	1.44E-16	521593	monocyte	TRUE	reported	igd	0.000131	68.36133	2	TRUE
rs7320305	G	C	G	C	-0.02609	0.030055	0.072008	0.105403	FALSE	TRUE	FALSE	I9_PULMEN	0.224563	0.024747	pulmonary	TRUE	reported	textfile	21	36407530	0.003673	1.27E-12	521591	monocyte	TRUE	reported	igd	9.67E-05	50.46665	2	TRUE
rs7321747	G	A	G	A	0.064788	-0.04578	0.046722	0.02038	FALSE	FALSE	FALSE	I9_PULMEN	0.385224	0.052727	pulmonary	TRUE	reported	textfile	13	72513420	0.004478	2.11E-47	521591	monocyte	TRUE	reported	igd	0.000401	209.3241	2	TRUE
rs7346759	C	T	C	T	0.018458	-0.0037	0.199459	0.352189	FALSE	FALSE	FALSE	I9_PULMEN	0.81546	0.015858	pulmonary	TRUE	reported	textfile	15	99088453	0.002363	5.95E-15	521590	monocyte	TRUE	reported	igd	0.000117	61.0155	2	TRUE
rs7378428	G	A	G	A	0.015482	0.026886	0.14927	0.130277	FALSE	FALSE	FALSE	I9_PULMEN	0.228265	0.022315	pulmonary	TRUE	reported	textfile	6	1.61E+08	0.002645	4.93E-09	520654	monocyte	TRUE	reported	igd	6.58E-05	34.26108	2	TRUE
rs7380916	C	T	C	T	0.046464	0.068335	0.023149	0.010066	FALSE	FALSE	FALSE	I9_PULMEN	0.361783	0.074931	pulmonary	TRUE	reported	textfile	4	36305937	0.006392	3.78E-13	521594	monocyte	TRUE	reported	igd	0.000101	52.83941	2	TRUE
rs739241	A	G	A	G	0.018082	0.028332	0.606788	0.621175	FALSE	FALSE	FALSE	I9_PULMEN	0.067481	0.015495	pulmonary	TRUE	reported	textfile	22	50052921	0.001948	1.72E-20	521586	monocyte	TRUE	reported	igd	0.000165	86.16152	2	TRUE
rs7423380	C	T	C	T	-0.02715	0.046991	0.080602	0.084327	FALSE	FALSE	FALSE	I9_PULMEN	0.081007	0.026931	pulmonary	TRUE	reported	textfile	10	1.05E+08	0.003444	3.34E-15	521589	monocyte	TRUE	reported	igd	0.000119	62.14579	2	TRUE
rs7425956	G	C	G	C	0.028706	-0.02306	0.100542	0.051955	FALSE	TRUE	FALSE	I9_PULMEN	0.495068	0.033801	pulmonary	TRUE	reported	textfile	19	42735177	0.003145	7.51E-20	521593	monocyte	TRUE	reported	igd	0.00016	83.31101	2	TRUE

rs745570	G	A	G	A	0.014068	0.032184	0.513574	0.50638	FALSE	FALSE	FALSE	I9_PULMEN	0.032048	0.015013	pulmonary	TRUE	reported	textfile	17	77781725	0.001948	5.38E-13	475322	monocyte	TRUE	reported	igd	0.00011	52.15368	2	TRUE
rs745822	G	T	G	T	0.016402	0.007741	0.375667	0.292097	FALSE	FALSE	FALSE	I9_PULMEN	0.638641	0.016484	pulmonary	TRUE	reported	textfile	18	48142822	0.001942	3.16E-17	520652	monocyte	TRUE	reported	igd	0.000137	71.33349	2	TRUE
rs7473500	C	T	C	T	-0.01698	-0.01909	0.206468	0.179576	FALSE	FALSE	FALSE	I9_PULMEN	0.328209	0.019525	pulmonary	TRUE	reported	textfile	5	57202727	0.002327	3.14E-13	521592	monocyte	TRUE	reported	igd	0.000102	53.22645	2	TRUE
rs7476524	T	C	T	C	0.012761	-0.00858	0.281465	0.28772	FALSE	FALSE	FALSE	I9_PULMEN	0.60396	0.016545	pulmonary	TRUE	reported	textfile	1	31194784	0.002093	1.12E-09	520653	monocyte	TRUE	reported	igd	7.14E-05	37.17314	2	TRUE
rs7516138	G	A	G	A	-0.02259	0.010163	0.391091	0.503833	FALSE	FALSE	FALSE	I9_PULMEN	0.498176	0.015004	pulmonary	TRUE	reported	textfile	1	9711642	0.00193	1.31E-31	521589	monocyte	TRUE	reported	igd	0.000263	137.0229	2	TRUE
rs7522307	C	G	C	G	-0.02328	-0.02291	0.124104	0.114032	FALSE	TRUE	FALSE	I9_PULMEN	0.331186	0.023574	pulmonary	TRUE	reported	textfile	1	2.08E+08	0.002862	4.36E-16	521591	monocyte	TRUE	reported	igd	0.000127	66.14745	2	TRUE
rs7524046	A	G	A	G	0.014654	0.030502	0.251336	0.165313	FALSE	FALSE	FALSE	I9_PULMEN	0.130446	0.020169	pulmonary	TRUE	reported	textfile	1	9307033	0.002156	1.11E-11	520185	monocyte	TRUE	reported	igd	8.88E-05	46.19694	2	TRUE
rs754388	C	G	C	G	0.030305	-0.03554	0.810895	0.857617	FALSE	TRUE	FALSE	I9_PULMEN	0.09888	0.021534	pulmonary	TRUE	reported	textfile	14	93115410	0.002421	6.43E-36	521592	monocyte	TRUE	reported	igd	0.0003	156.6886	2	TRUE
rs7547562	G	C	G	C	0.02413	0.003751	0.076659	0.088127	FALSE	TRUE	FALSE	I9_PULMEN	0.888162	0.026676	pulmonary	TRUE	reported	textfile	2	54787592	0.003587	1.80E-11	521593	monocyte	TRUE	reported	igd	8.68E-05	45.2533	2	TRUE
rs7569084	T	C	T	C	0.027559	0.00136	0.583833	0.646012	FALSE	FALSE	FALSE	I9_PULMEN	0.930924	0.015694	pulmonary	TRUE	reported	textfile	2	65656969	0.001895	7.40E-48	520194	monocyte	TRUE	reported	igd	0.000406	211.4983	2	TRUE
rs7572278	A	T	A	T	0.021687	0.009696	0.206833	0.219939	FALSE	TRUE	FALSE	I9_PULMEN	0.595552	0.018266	pulmonary	TRUE	reported	textfile	2	8563029	0.002355	3.44E-20	520512	monocyte	TRUE	reported	igd	0.000163	84.80383	2	TRUE
rs7574456	T	C	T	C	-0.04026	-0.01198	0.739962	0.859248	FALSE	FALSE	FALSE	I9_PULMEN	0.583542	0.02185	pulmonary	TRUE	reported	textfile	2	1.37E+08	0.002125	5.20E-80	519574	monocyte	TRUE	reported	igd	0.00069	358.8738	2	TRUE
rs7593080	T	C	T	C	0.010661	0.020211	0.574099	0.571997	FALSE	FALSE	FALSE	I9_PULMEN	0.182704	0.015168	pulmonary	TRUE	reported	textfile	2	86145787	0.001892	1.80E-08	521592	monocyte	TRUE	reported	igd	6.09E-05	31.7506	2	TRUE
rs761841	C	T	C	T	0.012242	0.015584	0.576839	0.599105	FALSE	FALSE	FALSE	I9_PULMEN	0.308141	0.015291	pulmonary	TRUE	reported	textfile	6	1.31E+08	0.001903	1.31E-10	520192	monocyte	TRUE	reported	igd	7.95E-05	41.38334	2	TRUE
rs7620353	T	C	T	C	0.014253	-0.01906	0.823086	0.847492	FALSE	FALSE	FALSE	I9_PULMEN	0.362028	0.020906	pulmonary	TRUE	reported	textfile	3	1.51E+08	0.002453	6.42E-09	520653	monocyte	TRUE	reported	igd	6.48E-05	33.76104	2	TRUE
rs7633965	C	A	C	A	0.025005	-0.04017	0.929301	0.962313	FALSE	FALSE	FALSE	I9_PULMEN	0.305776	0.039225	pulmonary	TRUE	reported	textfile	3	1.69E+08	0.003657	8.35E-12	520655	monocyte	TRUE	reported	igd	8.98E-05	46.75221	2	TRUE
rs7642728	C	T	C	T	0.024864	0.0132	0.254121	0.261984	FALSE	FALSE	FALSE	I9_PULMEN	0.443358	0.01722	pulmonary	TRUE	reported	textfile	19	837190	0.002345	3.04E-26	467281	monocyte	TRUE	reported	igd	0.000241	112.4228	2	TRUE
rs7642810	C	T	C	T	0.53552	-0.10983	0.013445	0.012756	FALSE	FALSE	FALSE	I9_PULMEN	0.108723	0.068471	pulmonary	TRUE	reported	textfile	13	28604007	0.00861	########	521592	monocyte	TRUE	reported	igd	0.007362	3868.507	2	TRUE
rs7773374	A	G	A	G	-0.01403	-0.00722	0.171887	0.181076	FALSE	FALSE	FALSE	I9_PULMEN	0.711055	0.019481	pulmonary	TRUE	reported	textfile	13	52344004	0.002496	1.96E-08	519734	monocyte	TRUE	reported	igd	6.08E-05	31.57743	2	TRUE
rs7776054	G	A	G	A	-0.03791	0.030199	0.260831	0.339957	FALSE	FALSE	FALSE	I9_PULMEN	0.057187	0.015878	pulmonary	TRUE	reported	textfile	6	1.35E+08	0.002128	6.09E-71	521129	monocyte	TRUE	reported	igd	0.000608	317.2839	2	TRUE
rs7785014	C	T	C	T	-0.02528	-0.02662	0.670805	0.690456	FALSE	FALSE	FALSE	I9_PULMEN	0.100693	0.016217	pulmonary	TRUE	reported	textfile	7	1.38E+08	0.002005	2.04E-36	520514	monocyte	TRUE	reported	igd	0.000305	158.9228	2	TRUE
rs7786376	G	A	G	A	0.012376	0.021944	0.275978	0.21573	FALSE	FALSE	FALSE	I9_PULMEN	0.229822	0.018274	pulmonary	TRUE	reported	textfile	7	73042614	0.002112	4.73E-09	521583	monocyte	TRUE	reported	igd	6.58E-05	34.33769	2	TRUE
rs7787179	G	A	G	A	0.020818	0.016153	0.206875	0.199524	FALSE	FALSE	FALSE	I9_PULMEN	0.391672	0.018857	pulmonary	TRUE	reported	textfile	7	1.43E+08	0.002322	3.31E-19	520508	monocyte	TRUE	reported	igd	0.000154	80.38072	2	TRUE
rs7803075	G	A	G	A	-0.01437	-0.00957	0.735284	0.640978	FALSE	FALSE	FALSE	I9_PULMEN	0.542716	0.01573	pulmonary	TRUE	reported	textfile	7	1.31E+08	0.002148	2.28E-11	519112	monocyte	TRUE	reported	igd	8.62E-05	44.76764	2	TRUE
rs7821885	T	C	T	C	0.021951	-0.01749	0.068442	0.072985	FALSE	FALSE	FALSE	I9_PULMEN	0.547509	0.029073	pulmonary	TRUE	reported	textfile	2	43556575	0.003688	2.73E-09	521594	monocyte	TRUE	reported	igd	6.79E-05	35.42625	2	TRUE
rs7824937	G	A	G	A	0.01766	-0.00616	0.368567	0.405042	FALSE	FALSE	FALSE	I9_PULMEN	0.686997	0.0153	pulmonary	TRUE	reported	textfile	8	61392724	0.001942	1.03E-19	521587	monocyte	TRUE	reported	igd	0.000159	82.69539	2	TRUE
rs7826487	G	A	G	A	-0.041	-0.00661	0.115769	0.073815	FALSE	FALSE	FALSE	I9_PULMEN	0.819811	0.029025	pulmonary	TRUE	reported	textfile	8	6880925	0.002967	2.17E-43	520195	monocyte	TRUE	reported	igd	0.000367	190.9363	2	TRUE
rs7836456	G	T	G	T	-0.01505	0.014151	0.514396	0.588298	FALSE	FALSE	FALSE	I9_PULMEN	0.356045	0.015333	pulmonary	TRUE	reported	textfile	8	11829175	0.00195	1.26E-14	477033	monocyte	TRUE	reported	igd	0.000125	59.53482	2	TRUE
rs7836786	A	G	A	G	-0.01324	-0.02256	0.814951	0.801357	FALSE	FALSE	FALSE	I9_PULMEN	0.23255	0.018896	pulmonary	TRUE	reported	textfile	8	1.21E+08	0.002419	4.46E-08	521591	monocyte	TRUE	reported	igd	5.75E-05	29.9754	2	TRUE
rs7861055	G	A	G	A	-0.01406	-0.01674	0.487561	0.475884	FALSE	FALSE	FALSE	I9_PULMEN	0.2674	0.015095	pulmonary	TRUE	reported	textfile	9	38197033	0.00187	5.66E-14	521588	monocyte	TRUE	reported	igd	0.000108	56.54697	2	TRUE
rs7919533	C	T	C	T	0.02038	0.012426	0.487915	0.415125	FALSE	FALSE	FALSE	I9_PULMEN	0.415559	0.015263	pulmonary	TRUE	reported	textfile	10	73525525	0.00191	1.55E-26	520190	monocyte	TRUE	reported	igd	0.000219	113.8518	2	TRUE
rs7933584	C	G	C	G	-0.02463	-0.02354	0.093816	0.055327	FALSE	TRUE	FALSE	I9_PULMEN	0.472626	0.03278	pulmonary	TRUE	reported	textfile	8	1.3E+08	0.003256	4.14E-14	521592	monocyte	TRUE	reported	igd	0.00011	57.19816	2	TRUE
rs7940771	A	G	A	G	-0.06435	0.000617	0.030003	0.018304	FALSE	FALSE	FALSE	I9_PULMEN	0.991256	0.056305	pulmonary	TRUE	reported	textfile	19	33745290	0.005795	1.28E-28	478114	monocyte	TRUE	reported	igd	0.000258	123.3225	2	TRUE
rs7944692	A	G	A	G	-0.02288	0.013989	0.053807	0.06463	FALSE	FALSE	FALSE	I9_PULMEN	0.648022	0.030643	pulmonary	TRUE	reported	textfile	9	91652798	0.004141	3.38E-08	521591	monocyte	TRUE	reported	igd	5.85E-05	30.52544	2	TRUE
rs7971382	A	G	A	G	0.011735	-0.00528	0.373961	0.305991	FALSE	FALSE	FALSE	I9_PULMEN	0.746985	0.016374	pulmonary	TRUE	reported	textfile	12	1079129	0.001983	3.34E-09	518758	monocyte	TRUE	reported	igd	6.75E-05	35.02024	2	TRUE
rs7975680	C	A	C	A	0.012577	0.003985	0.60413	0.567598	FALSE	FALSE	FALSE	I9_PULMEN	0.793535	0.015225	pulmonary	TRUE	reported	textfile	12	1.02E+08	0.001978	2.12E-10	520486	monocyte	TRUE	reported	igd	7.77E-05	40.42964	2	TRUE
rs798563	C	A	C	A	0.017561	-0.03524	0.297123	0.31072	FALSE	FALSE	FALSE	I9_PULMEN	0.030401	0.01628	pulmonary	TRUE	reported	textfile	7	2757867	0.00208	3.23E-17	521591	monocyte	TRUE	reported	igd	0.000137	71.2804	2	TRUE
rs7989433	A	G	A	G	-0.04821	0.042091	0.10046	0.107293	FALSE	FALSE	FALSE	I9_PULMEN	0.082718	0.024258	pulmonary	TRUE	reported	textfile	6	44591721	0.003116	6.30E-54	520515	monocyte	TRUE	reported	igd	0.00046	239.3247	2	TRUE
rs8049116	T	C	T	C	0.022989	0.025127	0.118269	0.127874	FALSE	FALSE	FALSE	I9_PULMEN	0.26566	0.022574	pulmonary	TRUE	reported	textfile	16	4150530	0.002903	2.52E-15	521561	monocyte	TRUE	reported	igd	0.00012	62.7111	2	TRUE
rs8077619	A	T	A	T	-0.01908	0.033514	0.132816	0.110125	FALSE	TRUE	FALSE	I9_PULMEN	0.161	0.02391	pulmonary	TRUE	reported	textfile	17	68141181	0.002765	5.39E-12	521594	monocyte	TRUE	reported	igd	9.13E-05	47.6223	2	TRUE
rs8207	G	A	G	A	0.013527	0.018429	0.26667	0.228397	FALSE	FALSE	FALSE	I9_PULMEN	0.302491	0.017873	pulmonary	TRUE	reported	textfile	2	1.7E+08	0.002103	1.29E-10	520195	monocyte	TRUE	reported	igd	7.95E-05	41.37355	2	TRUE
rs869785	C	T	C	T	-0.0172	-0.00787	0.669622	0.658851	FALSE	FALSE	FALSE	I9_PULMEN	0.619475	0.015839	pulmonary	TRUE	reported	textfile	3	24347800	0.001981	3.97E-18	521592	monocyte	TRUE	reported	igd	0.000145	75.4203	2	TRUE
rs871134	T	C	T	C	0.038297	0.040478	0.571095	0.556868	FALSE	FALSE	FALSE	I9_PULMEN	0.00737	0.015106	pulmonary	TRUE	reported	textfile	4	7044380	0.001915	7.06E-89	517676	monocyte	TRUE	reported	igd	0.000772	399.9358	2	TRUE
rs875890	A	T	A	T	-0.01686	-0.00948	0.262208	0.289573	FALSE	TRUE	FALSE	I9_PULMEN	0.565774	0.016502	pulmonary	TRUE	reported	textfile	3	45945287	0.002139	3.38E-15	521590	monocyte	TRUE	reported	igd	0.000119	62.13602	2	TRUE

rs907612	T	C	T	C	-0.03305	0.002651	0.380435	0.384785	FALSE	FALSE	FALSE	I9_PULMEM	0.863996	0.015477	pulmonary	TRUE	reported	textfile	11	1874221	0.002022	5.19E-60	477132	monocyte	TRUE	reported	igd	0.00056	267.213	2	TRUE
rs915125	T	C	T	C	0.033833	-0.00632	0.282198	0.306559	FALSE	FALSE	FALSE	I9_PULMEM	0.699393	0.016364	pulmonary	TRUE	reported	textfile	6	82463376	0.002086	4.34E-59	520193	monocyte	TRUE	reported	igd	0.000505	263.0575	2	TRUE
rs919217	C	T	C	T	-0.01462	-0.01298	0.148898	0.162639	FALSE	FALSE	FALSE	I9_PULMEM	0.527013	0.020513	pulmonary	TRUE	reported	textfile	12	1.09E+08	0.002646	3.38E-08	520638	monocyte	TRUE	reported	igd	5.86E-05	30.51653	2	TRUE
rs9295484	T	G	T	G	-0.02857	-0.13316	0.955853	0.984192	FALSE	FALSE	FALSE	I9_PULMEM	0.026207	0.059899	pulmonary	TRUE	reported	textfile	6	20838445	0.004614	6.10E-10	521593	monocyte	TRUE	reported	igd	7.35E-05	38.35175	2	TRUE
rs932641	A	G	A	G	-0.01353	0.019896	0.251475	0.178727	FALSE	FALSE	FALSE	I9_PULMEM	0.310453	0.019616	pulmonary	TRUE	reported	textfile	20	38567137	0.002174	4.99E-10	521592	monocyte	TRUE	reported	igd	7.43E-05	38.73244	2	TRUE
rs932905	A	G	A	G	0.021032	-0.00153	0.28178	0.35328	FALSE	FALSE	FALSE	I9_PULMEM	0.922556	0.015749	pulmonary	TRUE	reported	textfile	20	48796131	0.002083	6.11E-24	520187	monocyte	TRUE	reported	igd	0.000196	101.9485	2	TRUE
rs9375150	G	T	G	T	-0.01841	0.000573	0.446815	0.470017	FALSE	FALSE	FALSE	I9_PULMEM	0.969556	0.015024	pulmonary	TRUE	reported	textfile	6	1.23E+08	0.001886	1.84E-22	521592	monocyte	TRUE	reported	igd	0.000183	95.24316	2	TRUE
rs9379077	G	A	G	A	0.02858	-0.02256	0.200867	0.22264	FALSE	FALSE	FALSE	I9_PULMEM	0.209691	0.017988	pulmonary	TRUE	reported	textfile	6	7167170	0.00233	1.46E-34	521593	monocyte	TRUE	reported	igd	0.000288	150.4565	2	TRUE
rs9390460	C	T	C	T	0.011811	0.035132	0.53796	0.483678	FALSE	FALSE	FALSE	I9_PULMEM	0.019752	0.015071	pulmonary	TRUE	reported	textfile	6	1.48E+08	0.001874	3.01E-10	521560	monocyte	TRUE	reported	igd	7.62E-05	39.72213	2	TRUE
rs9410425	A	G	A	G	-0.03003	0.00568	0.324599	0.309139	FALSE	FALSE	FALSE	I9_PULMEM	0.725533	0.01618	pulmonary	TRUE	reported	textfile	9	91562311	0.001994	3.23E-51	520190	monocyte	TRUE	reported	igd	0.000436	226.7931	2	TRUE
rs9480737	G	A	G	A	-0.02168	0.020141	0.321131	0.373686	FALSE	FALSE	FALSE	I9_PULMEM	0.198122	0.01565	pulmonary	TRUE	reported	textfile	6	1.07E+08	0.002003	2.74E-27	520639	monocyte	TRUE	reported	igd	0.000225	117.1967	2	TRUE
rs9494573	C	T	C	T	-0.01566	0.002376	0.588851	0.559015	FALSE	FALSE	FALSE	I9_PULMEM	0.875278	0.015136	pulmonary	TRUE	reported	textfile	6	1.37E+08	0.001905	2.18E-16	520192	monocyte	TRUE	reported	igd	0.00013	67.54139	2	TRUE
rs9526475	C	T	C	T	-0.01232	0.00134	0.727988	0.71378	FALSE	FALSE	FALSE	I9_PULMEM	0.935562	0.016571	pulmonary	TRUE	reported	textfile	13	48964748	0.002103	4.90E-09	521592	monocyte	TRUE	reported	igd	6.57E-05	34.29167	2	TRUE
rs9532580	C	T	C	T	-0.02809	0.010204	0.263677	0.268832	FALSE	FALSE	FALSE	I9_PULMEM	0.545951	0.016899	pulmonary	TRUE	reported	textfile	13	41244260	0.002128	9.72E-40	521482	monocyte	TRUE	reported	igd	0.000334	174.2567	2	TRUE
rs954954	C	A	C	A	-0.02898	0.015445	0.105169	0.120213	FALSE	FALSE	FALSE	I9_PULMEM	0.504286	0.02313	pulmonary	TRUE	reported	textfile	18	60902328	0.003072	4.20E-21	520193	monocyte	TRUE	reported	igd	0.000171	89.00463	2	TRUE
rs9555596	C	T	C	T	0.022922	0.028801	0.383331	0.404662	FALSE	FALSE	FALSE	I9_PULMEM	0.061011	0.015374	pulmonary	TRUE	reported	textfile	13	1.1E+08	0.001929	1.55E-32	521568	monocyte	TRUE	reported	igd	0.000271	141.2014	2	TRUE
rs9564870	C	A	C	A	0.03165	-0.03004	0.05279	0.034215	FALSE	FALSE	FALSE	I9_PULMEM	0.464321	0.041053	pulmonary	TRUE	reported	textfile	13	72618818	0.004584	5.24E-12	428971	monocyte	TRUE	reported	igd	0.000111	47.67121	2	TRUE
rs9625746	C	G	C	G	-0.02126	-0.04658	0.412737	0.305821	FALSE	TRUE	FALSE	I9_PULMEM	0.004447	0.016374	pulmonary	TRUE	reported	textfile	22	29637658	0.001942	7.42E-28	521572	monocyte	TRUE	reported	igd	0.00023	119.8806	2	TRUE
rs9734613	G	A	G	A	0.028777	0.018474	0.963842	0.965495	FALSE	FALSE	FALSE	I9_PULMEM	0.653586	0.041166	pulmonary	TRUE	reported	textfile	11	46249863	0.00505	1.24E-08	521594	monocyte	TRUE	reported	igd	6.23E-05	32.47182	2	TRUE
rs9783374	C	T	C	T	0.020919	-0.01241	0.849046	0.715645	FALSE	FALSE	FALSE	I9_PULMEM	0.456332	0.016662	pulmonary	TRUE	reported	textfile	11	2340619	0.002683	6.69E-15	520507	monocyte	TRUE	reported	igd	0.000117	60.79094	2	TRUE
rs9787298	C	A	C	A	-0.01603	0.024796	0.210661	0.229119	FALSE	FALSE	FALSE	I9_PULMEM	0.166327	0.017915	pulmonary	TRUE	reported	textfile	1	2.04E+08	0.00229	2.66E-12	520653	monocyte	TRUE	reported	igd	9.41E-05	49.01204	2	TRUE
rs9809116	G	A	G	A	0.01741	0.001458	0.408841	0.333905	FALSE	FALSE	FALSE	I9_PULMEM	0.926982	0.015912	pulmonary	TRUE	reported	textfile	3	72397279	0.00191	8.21E-20	521589	monocyte	TRUE	reported	igd	0.000159	83.08625	2	TRUE
rs9815073	A	C	A	C	-0.0218	0.003488	0.345844	0.329305	FALSE	FALSE	FALSE	I9_PULMEM	0.828588	0.01611	pulmonary	TRUE	reported	textfile	3	1.88E+08	0.002061	4.16E-26	519566	monocyte	TRUE	reported	igd	0.000215	111.8705	2	TRUE
rs9898876	T	G	T	G	0.017631	-0.0102	0.197491	0.120786	FALSE	FALSE	FALSE	I9_PULMEM	0.658707	0.02309	pulmonary	TRUE	reported	textfile	17	7526962	0.002367	9.89E-14	518761	monocyte	TRUE	reported	igd	0.000107	55.48246	2	TRUE
rs9915112	G	A	G	A	-0.02656	0.019809	0.203142	0.242161	FALSE	FALSE	FALSE	I9_PULMEM	0.258472	0.01753	pulmonary	TRUE	reported	textfile	17	2007826	0.002328	4.21E-30	521564	monocyte	TRUE	reported	igd	0.000249	130.1241	2	TRUE
rs9943753	G	A	G	A	0.013507	0.004515	0.627675	0.564798	FALSE	FALSE	FALSE	I9_PULMEM	0.768237	0.015322	pulmonary	TRUE	reported	textfile	12	1.1E+08	0.001953	4.79E-12	519115	monocyte	TRUE	reported	igd	9.21E-05	47.83124	2	TRUE
rs9963693	C	T	C	T	0.013323	0.007596	0.260621	0.310157	FALSE	FALSE	FALSE	I9_PULMEM	0.640221	0.016252	pulmonary	TRUE	reported	textfile	18	45599257	0.002145	5.39E-10	520506	monocyte	TRUE	reported	igd	7.41E-05	38.57872	2	TRUE
rs9992013	G	C	G	C	0.022401	0.009894	0.136509	0.141235	FALSE	TRUE	FALSE	I9_PULMEM	0.645473	0.021507	pulmonary	TRUE	reported	textfile	4	2259463	0.002796	1.18E-15	521591	monocyte	TRUE	reported	igd	0.000123	64.18873	2	TRUE

Supplementary Table 6: Description of SNPs for neutrophil cell counts incorporated after MR-presso deletion

SNP	effect_al	other_all	effect_al	other_all	beta.expc	beta.outc	eaf.expo	eaf.outcc	remove	palindron	ambiguous	id.outcon	pval.outc	se.outcon	outcome	mr_keep.	pval_orig	data_sour	chr.expo	pos.expo	se.expo	pval.expc	samplesiz	exposure	mr_keep.	pval_orig	data_sour	R2	F	action	mr_keep
rs1025245	G	A	G	A	-0.01384	0.008563	0.425632	0.392726	FALSE	FALSE	FALSE	I9_PULMEI	0.577918	0.01539	pulmonary	TRUE	reported	textfile	7	47337530	0.001962	1.82E-12	519248	neutrophi	TRUE	reported	igd	9.58E-05	49.75191	2	TRUE
rs1042725	T	C	T	C	0.012765	-0.00608	0.494436	0.474992	FALSE	FALSE	FALSE	I9_PULMEI	0.686068	0.015048	pulmonary	TRUE	reported	textfile	12	66358347	0.001924	3.33E-11	517881	neutrophi	TRUE	reported	igd	8.50E-05	44.01795	2	TRUE
rs1044730	A	G	A	G	-0.01659	-0.00422	0.505743	0.511038	FALSE	FALSE	FALSE	I9_PULMEI	0.778725	0.015017	pulmonary	TRUE	reported	textfile	5	1.79E+08	0.001919	5.58E-18	519276	neutrophi	TRUE	reported	igd	0.000144	74.74695	2	TRUE
rs1047891	A	C	A	C	-0.01931	0.038443	0.315122	0.321403	FALSE	FALSE	FALSE	I9_PULMEI	0.017165	0.016131	pulmonary	TRUE	reported	textfile	2	2.12E+08	0.002064	8.79E-21	518348	neutrophi	TRUE	reported	igd	0.000169	87.5001	2	TRUE
rs1049863	T	C	T	C	-0.0302	0.03142	0.183683	0.134993	FALSE	FALSE	FALSE	I9_PULMEI	0.152593	0.021966	pulmonary	TRUE	reported	textfile	14	93103309	0.002491	8.73E-34	518825	neutrophi	TRUE	reported	igd	0.000283	146.9725	2	TRUE
rs1050991	A	T	A	T	-0.02105	0.009763	0.134894	0.153846	FALSE	TRUE	FALSE	I9_PULMEI	0.638218	0.020763	pulmonary	TRUE	reported	textfile	10	1.12E+08	0.00281	7.02E-14	519287	neutrophi	TRUE	reported	igd	0.000108	56.12708	2	TRUE
rs1075062	A	G	A	G	-0.01097	-0.01296	0.457717	0.443941	FALSE	FALSE	FALSE	I9_PULMEI	0.391607	0.015124	pulmonary	TRUE	reported	textfile	11	1.02E+08	0.001926	1.28E-08	519285	neutrophi	TRUE	reported	igd	6.24E-05	32.41179	2	TRUE
rs1076069	T	G	T	G	0.011474	0.006109	0.374011	0.38887	FALSE	FALSE	FALSE	I9_PULMEI	0.693416	0.015498	pulmonary	TRUE	reported	textfile	9	1.02E+08	0.002038	1.85E-08	518348	neutrophi	TRUE	reported	igd	6.11E-05	31.69711	2	TRUE
rs1078632	G	C	G	C	0.042348	-0.02236	0.595938	0.581729	FALSE	TRUE	FALSE	I9_PULMEI	0.142115	0.015235	pulmonary	TRUE	reported	textfile	10	99068738	0.001955	########	519281	neutrophi	TRUE	reported	igd	0.000903	469.2136	2	TRUE
rs1083302	T	C	T	C	0.01633	0.000511	0.310102	0.23989	FALSE	FALSE	FALSE	I9_PULMEI	0.976805	0.017575	pulmonary	TRUE	reported	textfile	11	3010390	0.002081	4.51E-15	519285	neutrophi	TRUE	reported	igd	0.000119	61.57814	2	TRUE
rs1084641	G	A	G	A	0.016659	0.000637	0.687379	0.655835	FALSE	FALSE	FALSE	I9_PULMEI	0.967893	0.015833	pulmonary	TRUE	reported	textfile	12	8206167	0.002076	1.08E-15	517886	neutrophi	TRUE	reported	igd	0.000124	64.39342	2	TRUE
rs1084902	G	C	G	C	-0.02406	0.001986	0.210147	0.212713	FALSE	TRUE	FALSE	I9_PULMEI	0.913868	0.018359	pulmonary	TRUE	reported	textfile	12	4332009	0.002361	2.34E-24	516457	neutrophi	TRUE	reported	igd	0.000201	103.8651	2	TRUE
rs1086436	C	T	C	T	0.023683	0.007716	0.502691	0.516335	FALSE	FALSE	FALSE	I9_PULMEI	0.606576	0.014984	pulmonary	TRUE	reported	textfile	1	8918313	0.001914	4.01E-35	519281	neutrophi	TRUE	reported	igd	0.000295	153.1045	2	TRUE
rs1086882	A	T	A	T	0.011508	0.007242	0.338117	0.414941	FALSE	TRUE	FALSE	I9_PULMEI	0.636435	0.015321	pulmonary	TRUE	reported	textfile	9	73031065	0.002066	2.60E-08	504396	neutrophi	TRUE	reported	igd	6.15E-05	31.02683	2	TRUE
rs1094554	T	C	T	C	0.011993	-0.0045	0.521167	0.459388	FALSE	FALSE	FALSE	I9_PULMEI	0.765464	0.015086	pulmonary	TRUE	reported	textfile	6	1.59E+08	0.001925	4.82E-10	519235	neutrophi	TRUE	reported	igd	7.47E-05	38.81437	2	TRUE
rs1094803	A	C	A	C	0.029687	-0.01628	0.210175	0.230288	FALSE	FALSE	FALSE	I9_PULMEI	0.361425	0.017836	pulmonary	TRUE	reported	textfile	6	42510305	0.002362	3.49E-36	517849	neutrophi	TRUE	reported	igd	0.000305	157.9687	2	TRUE
rs1099239	A	G	A	G	0.01389	0.003706	0.230846	0.217736	FALSE	FALSE	FALSE	I9_PULMEI	0.838344	0.018164	pulmonary	TRUE	reported	textfile	9	95433830	0.002279	1.14E-09	519282	neutrophi	TRUE	reported	igd	7.15E-05	37.14618	2	TRUE
rs1099547	C	T	C	T	-0.03042	0.00683	0.473501	0.472229	FALSE	FALSE	FALSE	I9_PULMEI	0.649545	0.015031	pulmonary	TRUE	reported	textfile	10	65010672	0.001923	2.86E-56	519284	neutrophi	TRUE	reported	igd	0.000482	250.1586	2	TRUE
rs1103919	A	G	A	G	0.018481	-0.03653	0.317401	0.309687	FALSE	FALSE	FALSE	I9_PULMEI	0.02452	0.016244	pulmonary	TRUE	reported	textfile	11	47375193	0.00207	4.53E-19	518155	neutrophi	TRUE	reported	igd	0.000154	79.70922	2	TRUE
rs1106488	A	G	A	G	-0.03416	0.004671	0.073489	0.086328	FALSE	FALSE	FALSE	I9_PULMEI	0.861944	0.026858	pulmonary	TRUE	reported	textfile	12	1.2E+08	0.003693	2.37E-20	519285	neutrophi	TRUE	reported	igd	0.000165	85.57595	2	TRUE
rs1108013	A	G	A	G	0.01197	-0.01799	0.305941	0.286085	FALSE	FALSE	FALSE	I9_PULMEI	0.281232	0.016691	pulmonary	TRUE	reported	textfile	17	29056519	0.002148	2.59E-08	518204	neutrophi	TRUE	reported	igd	5.99E-05	31.05404	2	TRUE
rs1110488	C	T	C	T	-0.02594	-0.03933	0.701718	0.596551	FALSE	FALSE	FALSE	I9_PULMEI	0.010327	0.015333	pulmonary	TRUE	reported	textfile	12	88843474	0.002106	7.84E-35	518347	neutrophi	TRUE	reported	igd	0.000293	151.736	2	TRUE
rs1122037	A	C	A	C	-0.01192	-0.06788	0.082773	0.077873	FALSE	FALSE	FALSE	I9_PULMEI	0.015407	0.02802	pulmonary	TRUE	reported	textfile	1	27019131	0.002062	7.70E-09	519283	neutrophi	TRUE	reported	igd	6.43E-05	33.39504	2	TRUE
rs1135172	G	A	G	A	0.012078	-0.03096	0.729972	0.744618	FALSE	FALSE	FALSE	I9_PULMEI	0.071942	0.017205	pulmonary	TRUE	reported	textfile	1	11301714	0.002164	2.46E-08	517263	neutrophi	TRUE	reported	igd	6.02E-05	31.15113	2	TRUE
rs1140506	T	C	T	C	-0.16255	-0.01307	0.011126	0.016906	FALSE	FALSE	FALSE	I9_PULMEI	0.820194	0.057504	pulmonary	TRUE	reported	textfile	2	2.19E+08	0.010051	8.74E-59	512494	neutrophi	TRUE	reported	igd	0.00051	261.5494	2	TRUE
rs1144271	A	G	A	G	0.031948	0.011622	0.036578	0.054391	FALSE	FALSE	FALSE	I9_PULMEI	0.726798	0.033263	pulmonary	TRUE	reported	textfile	1	1.59E+08	0.005372	2.81E-09	519262	neutrophi	TRUE	reported	igd	6.81E-05	35.36826	2	TRUE
rs1158022	A	G	A	G	-0.02321	-0.02114	0.46164	0.376662	FALSE	FALSE	FALSE	I9_PULMEI	0.171987	0.015475	pulmonary	TRUE	reported	textfile	1	2.27E+08	0.002028	2.76E-30	517887	neutrophi	TRUE	reported	igd	0.000253	130.9598	2	TRUE
rs1159649	G	C	G	C	-0.01361	-0.03246	0.315669	0.326846	FALSE	TRUE	FALSE	I9_PULMEI	0.042302	0.015988	pulmonary	TRUE	reported	textfile	11	12135604	0.002083	6.57E-11	519285	neutrophi	TRUE	reported	igd	8.22E-05	42.70352	2	TRUE
rs1162586	A	G	A	G	-0.01528	-0.01121	0.61034	0.627178	FALSE	FALSE	FALSE	I9_PULMEI	0.469645	0.015502	pulmonary	TRUE	reported	textfile	14	1.06E+08	0.002061	1.26E-13	473325	neutrophi	TRUE	reported	igd	0.000116	54.99412	2	TRUE
rs1164474	G	A	G	A	-0.03705	-0.08416	0.035073	0.027865	FALSE	FALSE	FALSE	I9_PULMEI	0.065035	0.045615	pulmonary	TRUE	reported	textfile	2	37501547	0.005232	1.47E-12	519286	neutrophi	TRUE	reported	igd	9.66E-05	50.15717	2	TRUE
rs1164866	A	G	A	G	-0.01418	0.028697	0.402087	0.409013	FALSE	FALSE	FALSE	I9_PULMEI	0.059972	0.015256	pulmonary	TRUE	reported	textfile	16	53122486	0.001963	5.43E-13	519273	neutrophi	TRUE	reported	igd	0.0001	52.1513	2	TRUE
rs1166319	A	G	A	G	0.040344	-0.01142	0.02482	0.007122	FALSE	FALSE	FALSE	I9_PULMEI	0.898891	0.089877	pulmonary	TRUE	reported	textfile	1	93229909	0.006236	1.02E-10	516330	neutrophi	TRUE	reported	igd	8.11E-05	41.85468	2	TRUE
rs1166453	T	C	T	C	-0.016	-0.00661	0.524958	0.529697	FALSE	FALSE	FALSE	I9_PULMEI	0.660628	0.015067	pulmonary	TRUE	reported	textfile	18	43724520	0.00193	1.16E-16	518320	neutrophi	TRUE	reported	igd	0.000133	68.76078	2	TRUE
rs1167309	A	G	A	G	-0.04884	0.027261	0.259947	0.295126	FALSE	FALSE	FALSE	I9_PULMEI	0.097963	0.016474	pulmonary	TRUE	reported	textfile	19	45742094	0.002196	########	519284	neutrophi	TRUE	reported	igd	0.000952	494.7162	2	TRUE
rs1168393	A	C	A	C	0.030649	-0.02369	0.055134	0.028642	FALSE	FALSE	FALSE	I9_PULMEI	0.599214	0.045082	pulmonary	TRUE	reported	textfile	2	1.28E+08	0.004249	5.72E-13	518348	neutrophi	TRUE	reported	igd	0.0001	52.03044	2	TRUE
rs1168925	A	G	A	G	-0.02456	0.014517	0.069886	0.084949	FALSE	FALSE	FALSE	I9_PULMEI	0.595772	0.027365	pulmonary	TRUE	reported	textfile	2	1.63E+08	0.00388	2.51E-10	518346	neutrophi	TRUE	reported	igd	7.73E-05	40.07397	2	TRUE
rs1169036	G	T	G	T	0.011899	-0.01247	0.63957	0.686722	FALSE	FALSE	FALSE	I9_PULMEI	0.440884	0.016178	pulmonary	TRUE	reported	textfile	2	2.2E+08	0.002008	3.21E-09	517267	neutrophi	TRUE	reported	igd	6.79E-05	35.11493	2	TRUE
rs1169119	T	C	T	C	0.015007	0.020977	0.217738	0.20922	FALSE	FALSE	FALSE	I9_PULMEI	0.259075	0.018587	pulmonary	TRUE	reported	textfile	2	1.12E+08	0.002371	2.52E-10	517888	neutrophi	TRUE	reported	igd	7.73E-05	40.06111	2	TRUE
rs1171255	G	A	G	A	0.030473	-0.00755	0.126219	0.108359	FALSE	FALSE	FALSE	I9_PULMEI	0.755541	0.024234	pulmonary	TRUE	reported	textfile	3	1.41E+08	0.002893	6.45E-26	519274	neutrophi	TRUE	reported	igd	0.000214	110.9512	2	TRUE
rs1171601	T	C	T	C	-0.01285	-0.01245	0.635724	0.708156	FALSE	FALSE	FALSE	I9_PULMEI	0.45097	0.016515	pulmonary	TRUE	reported	textfile	3	1.69E+08	0.001992	1.16E-10	517889	neutrophi	TRUE	reported	igd	8.04E-05	41.61918	2	TRUE
rs1172106	T	G	T	G	0.01439	0.009072	0.609084	0.672117	FALSE	FALSE	FALSE	I9_PULMEI	0.570907	0.016008	pulmonary	TRUE	reported	textfile	3	98408826	0.001974	3.22E-13	519283	neutrophi	TRUE	reported	igd	0.000102	53.1405	2	TRUE

rs1172362	G	A	G	A	-0.02693	-0.03222	0.290775	0.200351	FALSE	FALSE	FALSE	I9_PULMEM	0.085645	0.018743	pulmonary	TRUE	reported	textfile	4	72615362	0.002109	2.67E-37	519287	neutrophi	TRUE	reported	igd	0.000314	163.0731	2	TRUE
rs1173566	T	C	T	C	0.047562	0.03813	0.033581	0.022719	FALSE	FALSE	FALSE	I9_PULMEM	0.451831	0.05068	pulmonary	TRUE	reported	textfile	4	1.45E+08	0.00534	5.59E-19	519284	neutrophi	TRUE	reported	igd	0.000153	79.32974	2	TRUE
rs1174686	T	A	T	A	0.019488	-0.0505	0.133922	0.113931	FALSE	TRUE	FALSE	I9_PULMEM	0.033158	0.023705	pulmonary	TRUE	reported	textfile	9	1.13E+08	0.002853	8.74E-12	519283	neutrophi	TRUE	reported	igd	8.98E-05	46.65834	2	TRUE
rs1175304	C	T	C	T	0.011206	-0.00262	0.395413	0.46489	FALSE	FALSE	FALSE	I9_PULMEM	0.861811	0.01503	pulmonary	TRUE	reported	textfile	6	20735494	0.001968	1.27E-08	519283	neutrophi	TRUE	reported	igd	6.24E-05	32.42271	2	TRUE
rs1189419	A	C	A	C	0.011047	-0.02271	0.387646	0.317841	FALSE	FALSE	FALSE	I9_PULMEM	0.159209	0.016133	pulmonary	TRUE	reported	textfile	2	1.29E+08	0.001969	2.07E-08	518349	neutrophi	TRUE	reported	igd	6.07E-05	31.47716	2	TRUE
rs1199763	G	C	G	C	0.016578	0.025976	0.273556	0.319033	FALSE	TRUE	FALSE	I9_PULMEM	0.106166	0.016078	pulmonary	TRUE	reported	textfile	8	1.29E+08	0.00216	1.74E-14	519287	neutrophi	TRUE	reported	igd	0.000113	58.9054	2	TRUE
rs1211827	T	C	T	C	0.014887	-0.01218	0.351878	0.290283	FALSE	FALSE	FALSE	I9_PULMEM	0.460454	0.016506	pulmonary	TRUE	reported	textfile	1	56609296	0.002004	1.15E-13	519279	neutrophi	TRUE	reported	igd	0.000106	55.18452	2	TRUE
rs1218988	T	C	T	C	-0.01606	-0.01415	0.240741	0.274019	FALSE	FALSE	FALSE	I9_PULMEM	0.401688	0.016876	pulmonary	TRUE	reported	textfile	6	52366206	0.002269	1.54E-12	519285	neutrophi	TRUE	reported	igd	9.64E-05	50.07929	2	TRUE
rs1220359	T	C	T	C	0.013585	0.02304	0.214291	0.030878	FALSE	FALSE	FALSE	I9_PULMEM	0.602718	0.044266	pulmonary	TRUE	reported	textfile	6	396321	0.002405	1.66E-08	515057	neutrophi	TRUE	reported	igd	6.19E-05	31.90711	2	TRUE
rs1221426	A	G	A	G	0.019394	-0.0139	0.545471	0.615824	FALSE	FALSE	FALSE	I9_PULMEM	0.370825	0.015534	pulmonary	TRUE	reported	textfile	6	1.36E+08	0.001922	6.60E-24	519286	neutrophi	TRUE	reported	igd	0.000196	101.8184	2	TRUE
rs1232834	G	T	G	T	-0.0161	0.029563	0.372663	0.388883	FALSE	FALSE	FALSE	I9_PULMEM	0.054255	0.015359	pulmonary	TRUE	reported	textfile	2	43351269	0.001997	7.97E-16	519284	neutrophi	TRUE	reported	igd	0.000125	64.95673	2	TRUE
rs1237806	T	C	T	C	-0.0137	-0.00197	0.649709	0.683852	FALSE	FALSE	FALSE	I9_PULMEM	0.902719	0.016103	pulmonary	TRUE	reported	textfile	9	1.27E+08	0.002041	1.99E-11	517261	neutrophi	TRUE	reported	igd	8.71E-05	45.04293	2	TRUE
rs1245035	A	C	A	C	0.01631	-0.0062	0.628096	0.546265	FALSE	FALSE	FALSE	I9_PULMEM	0.680683	0.015064	pulmonary	TRUE	reported	textfile	12	64976049	0.001998	3.39E-16	516805	neutrophi	TRUE	reported	igd	0.000129	66.63697	2	TRUE
rs1246027	G	A	G	A	-0.01125	-0.02678	0.569495	0.449356	FALSE	FALSE	FALSE	I9_PULMEM	0.075877	0.015083	pulmonary	TRUE	reported	textfile	19	9411354	0.001936	6.43E-09	519286	neutrophi	TRUE	reported	igd	6.50E-05	33.76101	2	TRUE
rs1248126	C	T	C	T	-0.01461	0.002977	0.460009	0.474451	FALSE	FALSE	FALSE	I9_PULMEM	0.842798	0.015013	pulmonary	TRUE	reported	textfile	20	8126920	0.002019	4.79E-13	475803	neutrophi	TRUE	reported	igd	0.00011	52.35601	2	TRUE
rs1248765	T	C	T	C	0.017015	0.001051	0.801636	0.823408	FALSE	FALSE	FALSE	I9_PULMEM	0.957491	0.019712	pulmonary	TRUE	reported	textfile	3	71528943	0.002418	2.06E-12	517265	neutrophi	TRUE	reported	igd	9.57E-05	49.51647	2	TRUE
rs1250568	C	T	C	T	-0.01382	-0.01811	0.435133	0.51778	FALSE	FALSE	FALSE	I9_PULMEM	0.229044	0.015055	pulmonary	TRUE	reported	textfile	10	81045280	0.002024	8.89E-12	475803	neutrophi	TRUE	reported	igd	9.80E-05	46.62225	2	TRUE
rs1253060	G	A	G	A	-0.01617	-0.0288	0.146298	0.097372	FALSE	FALSE	FALSE	I9_PULMEM	0.253455	0.02522	pulmonary	TRUE	reported	textfile	7	18023617	0.002739	3.63E-09	516802	neutrophi	TRUE	reported	igd	6.75E-05	34.86115	2	TRUE
rs1254152	G	A	G	A	0.012739	0.008948	0.450195	0.338649	FALSE	FALSE	FALSE	I9_PULMEM	0.572789	0.015867	pulmonary	TRUE	reported	textfile	8	1.3E+08	0.001935	4.77E-11	519285	neutrophi	TRUE	reported	igd	8.35E-05	43.34181	2	TRUE
rs1255061	A	G	A	G	-0.03084	-0.01539	0.821991	0.767985	FALSE	FALSE	FALSE	I9_PULMEM	0.388734	0.017851	pulmonary	TRUE	reported	textfile	8	22966769	0.002548	1.09E-33	517887	neutrophi	TRUE	reported	igd	0.000283	146.4778	2	TRUE
rs1260326	C	T	C	T	-0.03251	0.007219	0.602032	0.650688	FALSE	FALSE	FALSE	I9_PULMEM	0.647251	0.015776	pulmonary	TRUE	reported	textfile	2	27730940	0.001958	6.91E-62	517885	neutrophi	TRUE	reported	igd	0.000532	275.715	2	TRUE
rs1265894	G	A	G	A	0.01303	0.02669	0.256773	0.307726	FALSE	FALSE	FALSE	I9_PULMEM	0.099878	0.016221	pulmonary	TRUE	reported	textfile	5	57552464	0.002195	3.01E-09	519284	neutrophi	TRUE	reported	igd	6.79E-05	35.23856	2	TRUE
rs1271664	C	G	C	G	0.014273	0.0136	0.637362	0.656897	FALSE	TRUE	FALSE	I9_PULMEM	0.389025	0.015789	pulmonary	TRUE	reported	textfile	8	6901304	0.002001	1.03E-12	519286	neutrophi	TRUE	reported	igd	9.80E-05	50.87854	2	TRUE
rs1280215	C	A	C	A	0.012586	-0.0104	0.252333	0.276914	FALSE	FALSE	FALSE	I9_PULMEM	0.535347	0.016775	pulmonary	TRUE	reported	textfile	11	28060285	0.002206	1.20E-08	518343	neutrophi	TRUE	reported	igd	6.28E-05	32.55088	2	TRUE
rs1290950	C	T	C	T	-0.02143	0.016751	0.428567	0.41501	FALSE	FALSE	FALSE	I9_PULMEM	0.271879	0.015245	pulmonary	TRUE	reported	textfile	15	51104350	0.001943	3.00E-28	519283	neutrophi	TRUE	reported	igd	0.000234	121.6004	2	TRUE
rs1292391	G	A	G	A	-0.0105	0.006849	0.509359	0.403138	FALSE	FALSE	FALSE	I9_PULMEM	0.654544	0.015306	pulmonary	TRUE	reported	textfile	16	24844403	0.001921	4.81E-08	517884	neutrophi	TRUE	reported	igd	5.76E-05	29.85322	2	TRUE
rs1292735	A	C	A	C	0.021608	-0.00259	0.198578	0.194279	FALSE	FALSE	FALSE	I9_PULMEM	0.891475	0.018965	pulmonary	TRUE	reported	textfile	16	74596618	0.002406	2.83E-19	519286	neutrophi	TRUE	reported	igd	0.000155	80.65592	2	TRUE
rs1293085	G	A	G	A	0.018982	0.026045	0.537098	0.579992	FALSE	FALSE	FALSE	I9_PULMEM	0.08667	0.015202	pulmonary	TRUE	reported	textfile	16	81602212	0.001938	1.29E-22	519283	neutrophi	TRUE	reported	igd	0.000185	95.93448	2	TRUE
rs1319003	G	A	G	A	-0.02181	0.01197	0.891442	0.820324	FALSE	FALSE	FALSE	I9_PULMEM	0.540084	0.019537	pulmonary	TRUE	reported	textfile	5	1.77E+08	0.003135	3.63E-12	519287	neutrophi	TRUE	reported	igd	9.32E-05	48.37661	2	TRUE
rs1329166	G	A	G	A	0.029314	-0.00389	0.187987	0.123252	FALSE	FALSE	FALSE	I9_PULMEM	0.8645	0.022797	pulmonary	TRUE	reported	textfile	9	282738	0.002462	1.19E-32	519280	neutrophi	TRUE	reported	igd	0.000273	141.7661	2	TRUE
rs1339297	A	G	A	G	0.028237	0.03065	0.053967	0.031722	FALSE	FALSE	FALSE	I9_PULMEM	0.475149	0.042919	pulmonary	TRUE	reported	textfile	2	1.93E+08	0.004266	3.73E-11	518349	neutrophi	TRUE	reported	igd	8.45E-05	43.81207	2	TRUE
rs1362623	T	C	T	C	0.018278	-0.01087	0.207792	0.189625	FALSE	FALSE	FALSE	I9_PULMEM	0.570163	0.019146	pulmonary	TRUE	reported	textfile	16	49885785	0.002376	1.49E-14	519285	neutrophi	TRUE	reported	igd	0.000114	59.17834	2	TRUE
rs1366045	C	T	C	T	-0.02188	0.012216	0.38497	0.343534	FALSE	FALSE	FALSE	I9_PULMEM	0.439232	0.015793	pulmonary	TRUE	reported	textfile	3	42909050	0.001969	1.20E-28	519283	neutrophi	TRUE	reported	igd	0.000238	123.4927	2	TRUE
rs1371794	C	A	C	A	-0.07253	0.006381	0.622286	0.671365	FALSE	FALSE	FALSE	I9_PULMEM	0.689349	0.015962	pulmonary	TRUE	reported	textfile	4	74948054	0.00198	########	519286	neutrophi	TRUE	reported	igd	0.002577	1341.848	2	TRUE
rs1403111	G	C	G	C	-0.02488	0.070106	0.051153	0.05324	FALSE	TRUE	FALSE	I9_PULMEM	0.035787	0.033394	pulmonary	TRUE	reported	textfile	4	26295540	0.004378	1.36E-08	519275	neutrophi	TRUE	reported	igd	6.22E-05	32.28812	2	TRUE
rs1410106	A	G	A	G	-0.04364	0.154085	0.01679	0.009951	FALSE	FALSE	FALSE	I9_PULMEM	0.040696	0.075288	pulmonary	TRUE	reported	textfile	5	1.32E+08	0.00778	2.08E-08	519284	neutrophi	TRUE	reported	igd	6.06E-05	31.46361	2	TRUE
rs1411424	A	G	A	G	-0.01149	0.009166	0.523053	0.506197	FALSE	FALSE	FALSE	I9_PULMEM	0.540525	0.014977	pulmonary	TRUE	reported	textfile	9	1.14E+08	0.001926	2.51E-09	517885	neutrophi	TRUE	reported	igd	6.87E-05	35.57743	2	TRUE
rs1412445	T	C	T	C	0.021069	-0.0167	0.338068	0.411798	FALSE	FALSE	FALSE	I9_PULMEM	0.272944	0.015232	pulmonary	TRUE	reported	textfile	10	91002804	0.002026	2.64E-25	519288	neutrophi	TRUE	reported	igd	0.000208	108.1452	2	TRUE
rs14243	G	A	G	A	0.013507	0.00389	0.210721	0.169512	FALSE	FALSE	FALSE	I9_PULMEM	0.846741	0.020125	pulmonary	TRUE	reported	textfile	2	2.34E+08	0.00247	4.66E-08	474409	neutrophi	TRUE	reported	igd	6.30E-05	29.9035	2	TRUE
rs1474419	C	T	C	T	0.017679	0.00487	0.578829	0.619091	FALSE	FALSE	FALSE	I9_PULMEM	0.754395	0.015566	pulmonary	TRUE	reported	textfile	7	6692605	0.001966	2.55E-19	517258	neutrophi	TRUE	reported	igd	0.000156	80.86241	2	TRUE
rs1487131	T	G	T	G	0.088156	0.071538	0.008947	0.004935	FALSE	FALSE	FALSE	I9_PULMEM	0.501753	0.106497	pulmonary	TRUE	reported	textfile	11	1.18E+08	0.010527	5.85E-17	515788	neutrophi	TRUE	reported	igd	0.000136	70.12822	2	TRUE
rs1490384	T	C	T	C	-0.01465	0.012583	0.501387	0.477961	FALSE	FALSE	FALSE	I9_PULMEM	0.401183	0.014989	pulmonary	TRUE	reported	textfile	6	1.27E+08	0.001917	2.19E-14	519281	neutrophi	TRUE	reported	igd	0.000113	58.43412	2	TRUE
rs1491570	T	C	T	C	-0.0312	-0.05073	0.039439	0.044855	FALSE	FALSE	FALSE	I9_PULMEM	0.161715	0.036255	pulmonary	TRUE	reported	textfile	17	56816881	0.005067	7.67E-10	519280	neutrophi	TRUE	reported	igd	7.30E-05	37.90481	2	TRUE
rs1508617	T	C	T	C	-0.05259	0.007787	0.019777	0.007946	FALSE	FALSE	FALSE	I9_PULMEM	0.928428	0.086694	pulmonary	TRUE	reported	textfile	13	1.09E+08	0.007502	2.47E-12	518863	neutrophi	TRUE	reported	igd	9.47E-05	49.14548	2	TRUE

rs1555275	G	A	G	A	-0.01369	0.003521	0.649639	0.695696	FALSE	FALSE	FALSE	I9_PULMEM	0.828976	0.0163	pulmonary	TRUE	reported	textfile	20	55969270	0.002046	2.30E-11	518346	neutrophi	TRUE	reported	igd	8.64E-05	44.77071	2	TRUE
rs156334	T	C	T	C	-0.01178	0.0023	0.429251	0.420987	FALSE	FALSE	FALSE	I9_PULMEM	0.880134	0.015253	pulmonary	TRUE	reported	textfile	20	1827809	0.002033	6.93E-09	472976	neutrophi	TRUE	reported	igd	7.10E-05	33.59765	2	TRUE
rs1571956	A	G	A	G	0.013715	-0.03647	0.646094	0.713481	FALSE	FALSE	FALSE	I9_PULMEM	0.028275	0.016626	pulmonary	TRUE	reported	textfile	10	30504708	0.002029	1.43E-11	517267	neutrophi	TRUE	reported	igd	8.83E-05	45.69049	2	TRUE
rs1611236	A	G	A	G	-0.01895	-0.0091	0.322908	0.224527	FALSE	FALSE	FALSE	I9_PULMEM	0.612858	0.017986	pulmonary	TRUE	reported	textfile	6	29748690	0.002057	3.35E-20	516795	neutrophi	TRUE	reported	igd	0.000164	84.88674	2	TRUE
rs1684335	C	G	C	G	-0.03587	0.085991	0.030427	0.035146	FALSE	TRUE	FALSE	I9_PULMEM	0.035541	0.040906	pulmonary	TRUE	reported	textfile	1	1.99E+08	0.005849	8.84E-10	484071	neutrophi	TRUE	reported	igd	7.77E-05	37.6179	2	TRUE
rs1685007	T	C	T	C	0.052054	0.001218	0.374485	0.385454	FALSE	FALSE	FALSE	I9_PULMEM	0.937135	0.01544	pulmonary	TRUE	reported	textfile	4	74703999	0.001999	########	517888	neutrophi	TRUE	reported	igd	0.001308	678.08	2	TRUE
rs1693960	A	G	A	G	-0.02845	-0.00969	0.146029	0.150267	FALSE	FALSE	FALSE	I9_PULMEM	0.643942	0.020967	pulmonary	TRUE	reported	textfile	8	79013333	0.002728	1.99E-25	517886	neutrophi	TRUE	reported	igd	0.00021	108.7307	2	TRUE
rs1695864	A	G	A	G	0.022296	-0.02418	0.082607	0.082258	FALSE	FALSE	FALSE	I9_PULMEM	0.379602	0.027518	pulmonary	TRUE	reported	textfile	16	11864032	0.00353	2.77E-10	517884	neutrophi	TRUE	reported	igd	7.70E-05	39.89356	2	TRUE
rs1696147	A	G	A	G	-0.01704	-0.04882	0.136787	0.105353	FALSE	FALSE	FALSE	I9_PULMEM	0.044595	0.024308	pulmonary	TRUE	reported	textfile	17	17146619	0.002794	1.11E-09	517886	neutrophi	TRUE	reported	igd	7.18E-05	37.17319	2	TRUE
rs1702621	G	T	G	T	-0.01684	-0.00288	0.512906	0.582845	FALSE	FALSE	FALSE	I9_PULMEM	0.850028	0.015232	pulmonary	TRUE	reported	textfile	2	85551696	0.001917	1.62E-18	518182	neutrophi	TRUE	reported	igd	0.000149	77.19568	2	TRUE
rs1713959	G	T	G	T	-0.03407	0.033405	0.039897	0.054584	FALSE	FALSE	FALSE	I9_PULMEM	0.310678	0.03295	pulmonary	TRUE	reported	textfile	7	64731746	0.004886	3.23E-12	517885	neutrophi	TRUE	reported	igd	9.39E-05	48.61376	2	TRUE
rs1721304	G	C	G	C	0.046028	0.030298	0.028346	0.014773	FALSE	TRUE	FALSE	I9_PULMEM	0.626151	0.062194	pulmonary	TRUE	reported	textfile	4	1.06E+08	0.00579	1.97E-15	519287	neutrophi	TRUE	reported	igd	0.000122	63.19539	2	TRUE
rs172240	A	G	A	G	-0.02044	0.020047	0.850405	0.875173	FALSE	FALSE	FALSE	I9_PULMEM	0.378788	0.022777	pulmonary	TRUE	reported	textfile	5	1.56E+08	0.002698	3.71E-14	519287	neutrophi	TRUE	reported	igd	0.000111	57.39529	2	TRUE
rs1738182	A	T	A	T	0.012856	-0.03926	0.280593	0.276787	FALSE	TRUE	FALSE	I9_PULMEM	0.019051	0.016746	pulmonary	TRUE	reported	textfile	15	77403477	0.002138	1.88E-09	519285	neutrophi	TRUE	reported	igd	6.96E-05	36.15719	2	TRUE
rs1744039	T	C	T	C	0.016462	0.02596	0.126191	0.117939	FALSE	FALSE	FALSE	I9_PULMEM	0.264777	0.023279	pulmonary	TRUE	reported	textfile	1	67190563	0.002896	1.35E-08	519287	neutrophi	TRUE	reported	igd	6.22E-05	32.31219	2	TRUE
rs174548	G	C	G	C	-0.02393	-0.04806	0.312967	0.389072	FALSE	TRUE	FALSE	I9_PULMEM	0.001792	0.015391	pulmonary	TRUE	reported	textfile	11	61571348	0.002072	7.91E-31	518341	neutrophi	TRUE	reported	igd	0.000257	133.3619	2	TRUE
rs17728	C	T	C	T	0.023764	-0.01801	0.909008	0.918373	FALSE	FALSE	FALSE	I9_PULMEM	0.510695	0.027388	pulmonary	TRUE	reported	textfile	1	2.05E+08	0.003344	1.23E-12	518349	neutrophi	TRUE	reported	igd	9.74E-05	50.50157	2	TRUE
rs1774200	A	C	A	C	-0.0178	0.001648	0.212295	0.208225	FALSE	FALSE	FALSE	I9_PULMEM	0.928924	0.018471	pulmonary	TRUE	reported	textfile	18	48124763	0.002354	4.10E-14	519283	neutrophi	TRUE	reported	igd	0.00011	57.20323	2	TRUE
rs1783150	C	A	C	A	-0.01991	-0.01855	0.223581	0.237509	FALSE	FALSE	FALSE	I9_PULMEM	0.291757	0.0176	pulmonary	TRUE	reported	textfile	9	1.16E+08	0.002302	5.64E-18	519278	neutrophi	TRUE	reported	igd	0.000144	74.76736	2	TRUE
rs1800961	T	C	T	C	-0.05194	0.030752	0.031018	0.045441	FALSE	FALSE	FALSE	I9_PULMEM	0.393441	0.036035	pulmonary	TRUE	reported	textfile	20	43042364	0.005553	8.99E-21	517885	neutrophi	TRUE	reported	igd	0.000169	87.49441	2	TRUE
rs180941	A	G	A	G	-0.01302	-0.00517	0.625196	0.753643	FALSE	FALSE	FALSE	I9_PULMEM	0.766526	0.017396	pulmonary	TRUE	reported	textfile	10	1.16E+08	0.002003	8.33E-11	518205	neutrophi	TRUE	reported	igd	8.15E-05	42.2401	2	TRUE
rs1885474	G	T	G	T	-0.02352	-0.00185	0.103145	0.168869	FALSE	FALSE	FALSE	I9_PULMEM	0.926476	0.020082	pulmonary	TRUE	reported	textfile	10	69566751	0.003162	1.08E-13	519272	neutrophi	TRUE	reported	igd	0.000106	55.30502	2	TRUE
rs1886654	C	T	C	T	-0.05846	0.029542	0.892013	0.914285	FALSE	FALSE	FALSE	I9_PULMEM	0.27078	0.026826	pulmonary	TRUE	reported	textfile	1	2.36E+08	0.003081	3.08E-80	519287	neutrophi	TRUE	reported	igd	0.000693	360.0126	2	TRUE
rs1887428	C	G	C	G	-0.01428	0.009308	0.625223	0.616205	FALSE	TRUE	FALSE	I9_PULMEM	0.547233	0.015464	pulmonary	TRUE	reported	textfile	9	4984530	0.002014	1.39E-12	517267	neutrophi	TRUE	reported	igd	9.72E-05	50.2872	2	TRUE
rs1939212	T	C	T	C	-0.01421	-0.02841	0.297421	0.285142	FALSE	FALSE	FALSE	I9_PULMEM	0.087807	0.016645	pulmonary	TRUE	reported	textfile	11	65626701	0.002108	1.62E-11	517876	neutrophi	TRUE	reported	igd	8.78E-05	45.45352	2	TRUE
rs1966479	G	A	G	A	0.014401	-0.00156	0.660217	0.664701	FALSE	FALSE	FALSE	I9_PULMEM	0.921613	0.015854	pulmonary	TRUE	reported	textfile	5	1.19E+08	0.002026	1.21E-12	518345	neutrophi	TRUE	reported	igd	9.75E-05	50.52482	2	TRUE
rs1977289	C	T	C	T	-0.01823	-0.01179	0.478516	0.397537	FALSE	FALSE	FALSE	I9_PULMEM	0.441777	0.015328	pulmonary	TRUE	reported	textfile	10	96301907	0.001918	2.14E-21	519280	neutrophi	TRUE	reported	igd	0.000174	90.3091	2	TRUE
rs1985157	C	T	C	T	0.026496	0.019847	0.410403	0.459475	FALSE	FALSE	FALSE	I9_PULMEM	0.189454	0.015125	pulmonary	TRUE	reported	textfile	19	18513594	0.002038	1.29E-38	473787	neutrophi	TRUE	reported	igd	0.000357	169.0248	2	TRUE
rs2001613	T	C	T	C	0.012012	-0.03751	0.472052	0.414861	FALSE	FALSE	FALSE	I9_PULMEM	0.013801	0.015234	pulmonary	TRUE	reported	textfile	17	75383679	0.002004	2.12E-09	474723	neutrophi	TRUE	reported	igd	7.57E-05	35.92803	2	TRUE
rs2038700	C	T	C	T	0.033462	0.012197	0.394281	0.388643	FALSE	FALSE	FALSE	I9_PULMEM	0.428232	0.015396	pulmonary	TRUE	reported	textfile	14	25461989	0.00197	1.25E-64	519282	neutrophi	TRUE	reported	igd	0.000555	288.5158	2	TRUE
rs2046934	A	G	A	G	0.015004	-0.02475	0.82324	0.839127	FALSE	FALSE	FALSE	I9_PULMEM	0.223853	0.020351	pulmonary	TRUE	reported	textfile	3	1.51E+08	0.002487	1.66E-09	519288	neutrophi	TRUE	reported	igd	7.01E-05	36.3966	2	TRUE
rs2052249	A	C	A	C	-0.01267	-0.00184	0.250937	0.254143	FALSE	FALSE	FALSE	I9_PULMEM	0.915213	0.017237	pulmonary	TRUE	reported	textfile	2	2.03E+08	0.002223	1.23E-08	517884	neutrophi	TRUE	reported	igd	6.27E-05	32.48423	2	TRUE
rs2062250	A	G	A	G	0.047004	-0.00811	0.938519	0.939946	FALSE	FALSE	FALSE	I9_PULMEM	0.796938	0.031531	pulmonary	TRUE	reported	textfile	15	64672002	0.004075	9.62E-31	517889	neutrophi	TRUE	reported	igd	0.000257	133.0493	2	TRUE
rs207253	A	G	A	G	-0.01236	0.020551	0.407863	0.340012	FALSE	FALSE	FALSE	I9_PULMEM	0.197798	0.015957	pulmonary	TRUE	reported	textfile	6	90808423	0.002018	9.42E-10	518345	neutrophi	TRUE	reported	igd	7.23E-05	37.48362	2	TRUE
rs2082382	A	G	A	G	-0.02948	-0.0223	0.552392	0.646122	FALSE	FALSE	FALSE	I9_PULMEM	0.154167	0.015651	pulmonary	TRUE	reported	textfile	5	1.48E+08	0.001942	5.39E-52	519287	neutrophi	TRUE	reported	igd	0.000443	230.3602	2	TRUE
rs212409	A	G	A	G	-0.02122	0.001153	0.554194	0.580723	FALSE	FALSE	FALSE	I9_PULMEM	0.9396	0.015214	pulmonary	TRUE	reported	textfile	6	1.59E+08	0.001932	4.71E-28	519285	neutrophi	TRUE	reported	igd	0.000232	120.6809	2	TRUE
rs2157770	G	A	G	A	0.02099	-0.02741	0.287199	0.411513	FALSE	FALSE	FALSE	I9_PULMEM	0.072093	0.01524	pulmonary	TRUE	reported	textfile	9	1.37E+08	0.002214	2.67E-21	475803	neutrophi	TRUE	reported	igd	0.000189	89.88098	2	TRUE
rs2179593	A	C	A	C	-0.01233	-0.01077	0.715877	0.714838	FALSE	FALSE	FALSE	I9_PULMEM	0.517315	0.016636	pulmonary	TRUE	reported	textfile	20	42660286	0.002141	8.62E-09	518203	neutrophi	TRUE	reported	igd	6.40E-05	33.16585	2	TRUE
rs218264	T	A	T	A	0.031802	-0.03441	0.250834	0.249337	FALSE	TRUE	FALSE	I9_PULMEM	0.048502	0.017443	pulmonary	TRUE	reported	textfile	4	55408875	0.002233	5.59E-46	519286	neutrophi	TRUE	reported	igd	0.00039	202.8289	2	TRUE
rs2241621	C	A	C	A	-0.01495	0.026768	0.588178	0.511861	FALSE	FALSE	FALSE	I9_PULMEM	0.075495	0.01506	pulmonary	TRUE	reported	textfile	14	81737076	0.001958	2.37E-14	516805	neutrophi	TRUE	reported	igd	0.000113	58.28263	2	TRUE
rs2254458	T	C	T	C	-0.01341	0.016308	0.644615	0.601959	FALSE	FALSE	FALSE	I9_PULMEM	0.288771	0.015373	pulmonary	TRUE	reported	textfile	20	1551485	0.0021	1.76E-10	472038	neutrophi	TRUE	reported	igd	8.64E-05	40.8015	2	TRUE
rs2260850	G	T	G	T	0.02432	-0.01134	0.280458	0.295406	FALSE	FALSE	FALSE	I9_PULMEM	0.489887	0.016427	pulmonary	TRUE	reported	textfile	13	1.14E+08	0.002212	4.43E-28	482991	neutrophi	TRUE	reported	igd	0.00025	120.8802	2	TRUE
rs2282611	G	T	G	T	-0.0126	0.018743	0.319925	0.32761	FALSE	FALSE	FALSE	I9_PULMEM	0.239963	0.015951	pulmonary	TRUE	reported	textfile	11	76154846	0.002063	1.03E-09	517886	neutrophi	TRUE	reported	igd	7.21E-05	37.32052	2	TRUE
rs2290846	A	G	A	G	0.018804	0.004349	0.284312	0.221811	FALSE	FALSE	FALSE	I9_PULMEM	0.809269	0.018019	pulmonary	TRUE	reported	textfile	4	1.51E+08	0.002127	9.92E-19	517885	neutrophi	TRUE	reported	igd	0.000151	78.15627	2	TRUE

rs2296028	C	G	C	G	-0.01529	-0.00765	0.17498	0.181589	FALSE	TRUE	FALSE	I9_PULMEM	0.694178	0.019468	pulmonary	TRUE	reported	textfile	13	52345637	0.002553	2.16E-09	519283	neutrophi	TRUE	reported	igd	6.91E-05	35.86837	2	TRUE
rs2301557	T	C	T	C	-0.12578	-0.04885	0.018212	0.026248	FALSE	FALSE	FALSE	I9_PULMEM	0.296844	0.046824	pulmonary	TRUE	reported	textfile	7	92300863	0.007198	2.53E-68	519288	neutrophi	TRUE	reported	igd	0.000588	305.3694	2	TRUE
rs2312675	C	T	C	T	-0.01985	-0.00308	0.211949	0.178431	FALSE	FALSE	FALSE	I9_PULMEM	0.874969	0.019565	pulmonary	TRUE	reported	textfile	1	1.67E+08	0.002343	2.61E-17	519287	neutrophi	TRUE	reported	igd	0.000138	71.73911	2	TRUE
rs2314339	T	C	T	C	0.033036	0.01637	0.124538	0.17646	FALSE	FALSE	FALSE	I9_PULMEM	0.406056	0.019703	pulmonary	TRUE	reported	textfile	17	38253212	0.002965	8.32E-29	517884	neutrophi	TRUE	reported	igd	0.00024	124.1435	2	TRUE
rs2315008	G	T	G	T	0.016403	0.020134	0.666951	0.740529	FALSE	FALSE	FALSE	I9_PULMEM	0.238483	0.01708	pulmonary	TRUE	reported	textfile	20	62343956	0.002058	1.64E-15	518828	neutrophi	TRUE	reported	igd	0.000122	63.52639	2	TRUE
rs2421200	T	G	T	G	-0.02053	0.004681	0.486641	0.567526	FALSE	FALSE	FALSE	I9_PULMEM	0.757602	0.015167	pulmonary	TRUE	reported	textfile	2	61711815	0.001917	1.03E-26	519279	neutrophi	TRUE	reported	igd	0.000221	114.6358	2	TRUE
rs2432142	A	G	A	G	0.01491	-0.04724	0.435047	0.402627	FALSE	FALSE	FALSE	I9_PULMEM	0.002066	0.015334	pulmonary	TRUE	reported	textfile	5	96275201	0.001945	1.89E-14	519264	neutrophi	TRUE	reported	igd	0.000113	58.76441	2	TRUE
rs2468832	A	C	A	C	0.015199	-0.0073	0.238866	0.262014	FALSE	FALSE	FALSE	I9_PULMEM	0.670213	0.01715	pulmonary	TRUE	reported	textfile	11	18163882	0.002272	2.33E-11	519284	neutrophi	TRUE	reported	igd	8.62E-05	44.75192	2	TRUE
rs2494747	T	G	T	G	0.012976	-0.03184	0.615478	0.606435	FALSE	FALSE	FALSE	I9_PULMEM	0.038854	0.015413	pulmonary	TRUE	reported	textfile	14	1.05E+08	0.002064	3.37E-10	474723	neutrophi	TRUE	reported	igd	8.33E-05	39.52396	2	TRUE
rs249677	A	C	A	C	0.02335	-0.02359	0.634081	0.694665	FALSE	FALSE	FALSE	I9_PULMEM	0.147142	0.016271	pulmonary	TRUE	reported	textfile	5	1.42E+08	0.001997	1.47E-31	519286	neutrophi	TRUE	reported	igd	0.000263	136.7149	2	TRUE
rs2522051	C	T	C	T	0.024	0.021312	0.454362	0.578309	FALSE	FALSE	FALSE	I9_PULMEM	0.161576	0.015225	pulmonary	TRUE	reported	textfile	5	1.32E+08	0.00193	1.88E-35	516807	neutrophi	TRUE	reported	igd	0.000299	154.6344	2	TRUE
rs2522440	C	T	C	T	0.018925	0.004699	0.911887	0.901774	FALSE	FALSE	FALSE	I9_PULMEM	0.852073	0.025201	pulmonary	TRUE	reported	textfile	4	1.09E+08	0.003409	2.90E-08	519288	neutrophi	TRUE	reported	igd	5.93E-05	30.81883	2	TRUE
rs2561758	G	A	G	A	-0.03401	0.024315	0.72298	0.726442	FALSE	FALSE	FALSE	I9_PULMEM	0.148312	0.016821	pulmonary	TRUE	reported	textfile	5	1.73E+08	0.002173	3.49E-55	518348	neutrophi	TRUE	reported	igd	0.000472	244.9727	2	TRUE
rs2632372	C	T	C	T	-0.02098	-0.00014	0.503861	0.476249	FALSE	FALSE	FALSE	I9_PULMEM	0.992422	0.015078	pulmonary	TRUE	reported	textfile	2	1.7E+08	0.001915	6.68E-28	518345	neutrophi	TRUE	reported	igd	0.000232	120.0479	2	TRUE
rs2665405	A	G	A	G	0.02951	-0.00399	0.54844	0.537767	FALSE	FALSE	FALSE	I9_PULMEM	0.790582	0.015037	pulmonary	TRUE	reported	textfile	17	57875292	0.001927	6.74E-53	519286	neutrophi	TRUE	reported	igd	0.000451	234.5165	2	TRUE
rs2710804	C	T	C	T	0.019677	0.02718	0.376402	0.353858	FALSE	FALSE	FALSE	I9_PULMEM	0.083123	0.015685	pulmonary	TRUE	reported	textfile	7	36084529	0.001984	3.76E-23	517886	neutrophi	TRUE	reported	igd	0.00019	98.36322	2	TRUE
rs2729707	G	A	G	A	-0.03566	-0.00605	0.830755	0.791994	FALSE	FALSE	FALSE	I9_PULMEM	0.74417	0.018544	pulmonary	TRUE	reported	textfile	2	1.61E+08	0.002553	2.68E-44	518337	neutrophi	TRUE	reported	igd	0.000376	195.0462	2	TRUE
rs2734031	C	T	C	T	-0.06752	0.039148	0.907968	0.914447	FALSE	FALSE	FALSE	I9_PULMEM	0.142793	0.026714	pulmonary	TRUE	reported	textfile	3	1.28E+08	0.003335	4.20E-91	519288	neutrophi	TRUE	reported	igd	0.000789	409.9061	2	TRUE
rs2807742	A	G	A	G	0.032553	-0.04096	0.771408	0.799297	FALSE	FALSE	FALSE	I9_PULMEM	0.029065	0.018767	pulmonary	TRUE	reported	textfile	10	28781367	0.002294	1.16E-45	518339	neutrophi	TRUE	reported	igd	0.000388	201.3694	2	TRUE
rs2808519	T	G	T	G	0.016109	-0.01732	0.737582	0.801201	FALSE	FALSE	FALSE	I9_PULMEM	0.35602	0.01876	pulmonary	TRUE	reported	textfile	1	2E+08	0.002179	1.50E-13	518347	neutrophi	TRUE	reported	igd	0.000105	54.65388	2	TRUE
rs2824372	C	G	C	G	-0.01551	0.015065	0.202687	0.241588	FALSE	TRUE	FALSE	I9_PULMEM	0.39323	0.017645	pulmonary	TRUE	reported	textfile	21	18947037	0.002443	2.21E-10	518203	neutrophi	TRUE	reported	igd	7.78E-05	40.32728	2	TRUE
rs2839183	A	G	A	G	-0.01313	########	0.404817	0.333634	FALSE	FALSE	FALSE	I9_PULMEM	0.998555	0.015893	pulmonary	TRUE	reported	textfile	21	47687144	0.001958	2.10E-11	519284	neutrophi	TRUE	reported	igd	8.66E-05	44.96103	2	TRUE
rs284317	G	A	G	A	0.01361	-0.00205	0.495955	0.606881	FALSE	FALSE	FALSE	I9_PULMEM	0.893881	0.015401	pulmonary	TRUE	reported	textfile	1	10731625	0.002018	1.60E-11	473456	neutrophi	TRUE	reported	igd	9.61E-05	45.48541	2	TRUE
rs2852800	G	C	G	C	0.0161	0.017833	0.738635	0.801448	FALSE	TRUE	FALSE	I9_PULMEM	0.344926	0.018882	pulmonary	TRUE	reported	textfile	11	1.14E+08	0.002224	4.65E-13	519287	neutrophi	TRUE	reported	igd	0.000101	52.40594	2	TRUE
rs2853075	A	G	A	G	0.05608	0.025965	0.043105	0.02539	FALSE	FALSE	FALSE	I9_PULMEM	0.584933	0.047537	pulmonary	TRUE	reported	textfile	4	36312542	0.004812	2.36E-31	518347	neutrophi	TRUE	reported	igd	0.000262	135.8198	2	TRUE
rs2854010	C	T	C	T	0.01708	-0.01791	0.658597	0.716488	FALSE	FALSE	FALSE	I9_PULMEM	0.281694	0.016641	pulmonary	TRUE	reported	textfile	19	4975763	0.00205	8.51E-17	519253	neutrophi	TRUE	reported	igd	0.000134	69.41708	2	TRUE
rs2857176	C	T	C	T	-0.02237	0.020521	0.206001	0.233297	FALSE	FALSE	FALSE	I9_PULMEM	0.247921	0.017761	pulmonary	TRUE	reported	textfile	8	55449281	0.002368	3.80E-21	519281	neutrophi	TRUE	reported	igd	0.000172	89.20162	2	TRUE
rs2858814	T	C	T	C	0.015218	-0.01393	0.188391	0.178429	FALSE	FALSE	FALSE	I9_PULMEM	0.479585	0.019706	pulmonary	TRUE	reported	textfile	12	10097609	0.002462	6.60E-10	518349	neutrophi	TRUE	reported	igd	7.37E-05	38.20651	2	TRUE
rs2923416	G	A	G	A	0.011252	0.010786	0.609864	0.648208	FALSE	FALSE	FALSE	I9_PULMEM	0.49299	0.015734	pulmonary	TRUE	reported	textfile	8	42457038	0.002051	4.21E-08	475806	neutrophi	TRUE	reported	igd	6.33E-05	30.09721	2	TRUE
rs2955958	A	G	A	G	0.012279	0.007572	0.315166	0.266942	FALSE	FALSE	FALSE	I9_PULMEM	0.656987	0.01705	pulmonary	TRUE	reported	textfile	15	1.02E+08	0.002139	9.67E-09	503312	neutrophi	TRUE	reported	igd	6.55E-05	32.95359	2	TRUE
rs3014874	A	G	A	G	0.012124	0.037222	0.265115	0.252737	FALSE	FALSE	FALSE	I9_PULMEM	0.031026	0.017258	pulmonary	TRUE	reported	textfile	1	1.53E+08	0.002182	2.81E-08	518345	neutrophi	TRUE	reported	igd	5.96E-05	30.87313	2	TRUE
rs301817	A	C	A	C	-0.01407	0.00252	0.583346	0.657335	FALSE	FALSE	FALSE	I9_PULMEM	0.873311	0.015806	pulmonary	TRUE	reported	textfile	1	8503379	0.001952	5.89E-13	519258	neutrophi	TRUE	reported	igd	0.0001	51.94018	2	TRUE
rs303753	A	G	A	G	-0.02276	0.01157	0.345097	0.35143	FALSE	FALSE	FALSE	I9_PULMEM	0.462481	0.015746	pulmonary	TRUE	reported	textfile	18	21074922	0.002045	9.57E-29	519278	neutrophi	TRUE	reported	igd	0.000239	123.8781	2	TRUE
rs305082	C	T	C	T	0.036633	0.007259	0.172255	0.243702	FALSE	FALSE	FALSE	I9_PULMEM	0.678199	0.017495	pulmonary	TRUE	reported	textfile	16	85936978	0.002594	3.04E-45	519284	neutrophi	TRUE	reported	igd	0.000384	199.4359	2	TRUE
rs3184504	C	T	C	T	-0.02939	-0.0286	0.517462	0.59144	FALSE	FALSE	FALSE	I9_PULMEM	0.060823	0.015254	pulmonary	TRUE	reported	textfile	12	1.12E+08	0.001927	1.90E-52	516804	neutrophi	TRUE	reported	igd	0.00045	232.5814	2	TRUE
rs3395198	T	C	T	C	-0.02763	0.015309	0.12936	0.129284	FALSE	FALSE	FALSE	I9_PULMEM	0.492595	0.022309	pulmonary	TRUE	reported	textfile	7	73029437	0.00287	6.63E-22	519280	neutrophi	TRUE	reported	igd	0.000178	92.66207	2	TRUE
rs3421589	A	G	A	G	0.038125	0.0166	0.028499	0.02765	FALSE	FALSE	FALSE	I9_PULMEM	0.723311	0.046886	pulmonary	TRUE	reported	textfile	8	21767240	0.006009	2.30E-10	518823	neutrophi	TRUE	reported	igd	7.76E-05	40.25442	2	TRUE
rs342242	C	T	C	T	-0.01577	0.016571	0.453135	0.461669	FALSE	FALSE	FALSE	I9_PULMEM	0.27052	0.015039	pulmonary	TRUE	reported	textfile	7	1.06E+08	0.001933	3.62E-16	519285	neutrophi	TRUE	reported	igd	0.000128	66.54077	2	TRUE
rs3459908	T	C	T	C	-0.17302	0.201138	0.013435	0.006126	FALSE	FALSE	FALSE	I9_PULMEM	0.034186	0.094972	pulmonary	TRUE	reported	textfile	1	1.59E+08	0.008416	7.27E-94	518431	neutrophi	TRUE	reported	igd	0.000815	422.6535	2	TRUE
rs3476566	C	T	C	T	0.041722	0.028924	0.065332	0.102216	FALSE	FALSE	FALSE	I9_PULMEM	0.242752	0.024761	pulmonary	TRUE	reported	textfile	14	69852940	0.003891	8.58E-27	518206	neutrophi	TRUE	reported	igd	0.000222	114.9757	2	TRUE
rs3499059	C	T	C	T	0.013989	-0.01249	0.188767	0.274115	FALSE	FALSE	FALSE	I9_PULMEM	0.457298	0.016799	pulmonary	TRUE	reported	textfile	1	1.7E+08	0.002448	1.13E-08	519282	neutrophi	TRUE	reported	igd	6.29E-05	32.65494	2	TRUE
rs3502013	C	T	C	T	-0.01762	0.001381	0.675273	0.680879	FALSE	FALSE	FALSE	I9_PULMEM	0.931883	0.016153	pulmonary	TRUE	reported	textfile	1	2.48E+08	0.002063	1.42E-17	517267	neutrophi	TRUE	reported	igd	0.000141	72.93943	2	TRUE
rs3528407	A	G	A	G	0.014664	0.035804	0.216975	0.218898	FALSE	FALSE	FALSE	I9_PULMEM	0.049117	0.018197	pulmonary	TRUE	reported	textfile	22	17624425	0.002358	5.15E-10	518346	neutrophi	TRUE	reported	igd	7.46E-05	38.67369	2	TRUE
rs354703	C	T	C	T	0.01478	-0.00765	0.593464	0.617409	FALSE	FALSE	FALSE	I9_PULMEM	0.620696	0.015451	pulmonary	TRUE	reported	textfile	2	1.44E+08	0.001954	4.08E-14	518345	neutrophi	TRUE	reported	igd	0.00011	57.21344	2	TRUE

rs3573424	C	T	C	T	0.023416	0.004898	0.427831	0.453915	FALSE	FALSE	FALSE	I9_PULMEN	0.746605	0.015157	pulmonary	TRUE	reported	textfile	4	706700	0.002022	5.42E-31	474868	neutrophi	TRUE	reported	igd	0.000282	134.11	2	TRUE
rs3575934	T	C	T	C	0.012607	-0.01917	0.449913	0.390441	FALSE	FALSE	FALSE	I9_PULMEN	0.212351	0.015369	pulmonary	TRUE	reported	textfile	7	1.16E+08	0.001926	6.19E-11	519287	neutrophi	TRUE	reported	igd	8.25E-05	42.8459	2	TRUE
rs3578917	G	T	G	T	0.017959	-0.01393	0.186171	0.155507	FALSE	FALSE	FALSE	I9_PULMEN	0.502557	0.02077	pulmonary	TRUE	reported	textfile	2	1.03E+08	0.002466	3.41E-13	518346	neutrophi	TRUE	reported	igd	0.000102	53.0367	2	TRUE
rs3586491	T	C	T	C	-0.01137	0.00274	0.37651	0.375186	FALSE	FALSE	FALSE	I9_PULMEN	0.860023	0.015537	pulmonary	TRUE	reported	textfile	12	1.08E+08	0.001982	1.01E-08	519283	neutrophi	TRUE	reported	igd	6.33E-05	32.88564	2	TRUE
rs3591518	C	T	C	T	0.024312	0.034098	0.215051	0.231317	FALSE	FALSE	FALSE	I9_PULMEN	0.055116	0.017778	pulmonary	TRUE	reported	textfile	1	2.25E+08	0.002345	3.67E-25	519287	neutrophi	TRUE	reported	igd	0.000207	107.4865	2	TRUE
rs3592965	C	T	C	T	-0.01955	0.013892	0.179844	0.183403	FALSE	FALSE	FALSE	I9_PULMEN	0.477619	0.019562	pulmonary	TRUE	reported	textfile	16	2165630	0.002634	1.21E-13	474389	neutrophi	TRUE	reported	igd	0.000116	55.09403	2	TRUE
rs3731332	T	C	T	C	-0.1196	0.041051	0.022894	0.018182	FALSE	FALSE	FALSE	I9_PULMEN	0.467371	0.056484	pulmonary	TRUE	reported	textfile	7	92300568	0.006536	9.69E-75	518348	neutrophi	TRUE	reported	igd	0.000646	334.8506	2	TRUE
rs3735311	C	T	C	T	-0.01953	-0.0091	0.648694	0.702765	FALSE	FALSE	FALSE	I9_PULMEN	0.579302	0.016411	pulmonary	TRUE	reported	textfile	7	1.49E+08	0.002011	2.82E-22	518347	neutrophi	TRUE	reported	igd	0.000182	94.33386	2	TRUE
rs3735485	G	A	G	A	0.029483	0.033871	0.844938	0.876107	FALSE	FALSE	FALSE	I9_PULMEN	0.136486	0.022747	pulmonary	TRUE	reported	textfile	7	45009341	0.002681	4.20E-28	518828	neutrophi	TRUE	reported	igd	0.000233	120.9339	2	TRUE
rs3739873	A	G	A	G	-0.01388	0.005822	0.22025	0.281479	FALSE	FALSE	FALSE	I9_PULMEN	0.727676	0.01672	pulmonary	TRUE	reported	textfile	9	34978431	0.002347	3.45E-09	517888	neutrophi	TRUE	reported	igd	6.75E-05	34.96439	2	TRUE
rs3747869	C	A	C	A	0.032659	-0.01835	0.900896	0.811551	FALSE	FALSE	FALSE	I9_PULMEN	0.343324	0.019361	pulmonary	TRUE	reported	textfile	10	73520632	0.003343	1.62E-22	474409	neutrophi	TRUE	reported	igd	0.000201	95.44016	2	TRUE
rs3749440	G	A	G	A	0.017362	0.032122	0.372369	0.292531	FALSE	FALSE	FALSE	I9_PULMEN	0.051271	0.01648	pulmonary	TRUE	reported	textfile	3	1.84E+08	0.001984	2.20E-18	517883	neutrophi	TRUE	reported	igd	0.000148	76.57985	2	TRUE
rs3754224	C	T	C	T	-0.01671	0.014417	0.270043	0.318878	FALSE	FALSE	FALSE	I9_PULMEN	0.370881	0.016112	pulmonary	TRUE	reported	textfile	1	43423622	0.002194	2.70E-14	519274	neutrophi	TRUE	reported	igd	0.000112	58.03445	2	TRUE
rs3762297	T	C	T	C	0.023896	-0.02005	0.183345	0.208873	FALSE	FALSE	FALSE	I9_PULMEN	0.278807	0.018516	pulmonary	TRUE	reported	textfile	1	31231680	0.002522	2.83E-21	517881	neutrophi	TRUE	reported	igd	0.000173	89.77565	2	TRUE
rs3777755	T	C	T	C	0.0137	-0.04864	0.311419	0.285528	FALSE	FALSE	FALSE	I9_PULMEN	0.003573	0.016695	pulmonary	TRUE	reported	textfile	6	12159699	0.002081	4.78E-11	517876	neutrophi	TRUE	reported	igd	8.37E-05	43.34064	2	TRUE
rs3781454	A	G	A	G	0.024234	-0.01043	0.677892	0.590535	FALSE	FALSE	FALSE	I9_PULMEN	0.495473	0.015299	pulmonary	TRUE	reported	textfile	10	1.26E+08	0.002094	6.26E-31	519286	neutrophi	TRUE	reported	igd	0.000258	133.9353	2	TRUE
rs3793537	C	G	C	G	0.012622	-0.02074	0.291154	0.237272	FALSE	TRUE	FALSE	I9_PULMEN	0.240466	0.017666	pulmonary	TRUE	reported	textfile	9	35687556	0.002117	2.55E-09	519279	neutrophi	TRUE	reported	igd	6.85E-05	35.54782	2	TRUE
rs3843301	G	T	G	T	-0.01891	-0.00769	0.364763	0.396227	FALSE	FALSE	FALSE	I9_PULMEN	0.631296	0.016028	pulmonary	TRUE	reported	textfile	1	1.62E+08	0.002076	8.70E-20	518195	neutrophi	TRUE	reported	igd	0.00016	82.98862	2	TRUE
rs3856364	G	C	G	C	0.014282	0.005623	0.679714	0.631252	FALSE	TRUE	FALSE	I9_PULMEN	0.717634	0.015551	pulmonary	TRUE	reported	textfile	2	1.45E+08	0.002069	5.28E-12	517267	neutrophi	TRUE	reported	igd	9.21E-05	47.64918	2	TRUE
rs3861100	G	A	G	A	0.018074	0.020506	0.347896	0.271281	FALSE	FALSE	FALSE	I9_PULMEN	0.22376	0.016855	pulmonary	TRUE	reported	textfile	12	50599219	0.002017	3.43E-19	519281	neutrophi	TRUE	reported	igd	0.000155	80.29622	2	TRUE
rs386243	T	C	T	C	-0.0264	-0.02139	0.250107	0.359791	FALSE	FALSE	FALSE	I9_PULMEN	0.173791	0.015727	pulmonary	TRUE	reported	textfile	19	49127490	0.00229	1.03E-30	518347	neutrophi	TRUE	reported	igd	0.000256	132.9434	2	TRUE
rs4074882	C	T	C	T	0.02794	0.044558	0.741751	0.853794	FALSE	FALSE	FALSE	I9_PULMEN	0.03627	0.02128	pulmonary	TRUE	reported	textfile	2	2.38E+08	0.002198	5.61E-37	517889	neutrophi	TRUE	reported	igd	0.000312	161.583	2	TRUE
rs409801	C	T	C	T	0.021089	0.009875	0.502787	0.569642	FALSE	FALSE	FALSE	I9_PULMEN	0.516026	0.015204	pulmonary	TRUE	reported	textfile	9	4744743	0.001929	8.59E-28	517264	neutrophi	TRUE	reported	igd	0.000231	119.5214	2	TRUE
rs4127253	G	A	G	A	-0.03588	-0.00506	0.046043	0.03776	FALSE	FALSE	FALSE	I9_PULMEN	0.898508	0.039637	pulmonary	TRUE	reported	textfile	1	1.83E+08	0.004892	2.33E-13	475807	neutrophi	TRUE	reported	igd	0.000113	53.79354	2	TRUE
rs4131338	A	C	A	C	0.058668	0.029334	0.030511	0.017483	FALSE	FALSE	FALSE	I9_PULMEN	0.610536	0.057596	pulmonary	TRUE	reported	textfile	1	79411968	0.005486	1.16E-26	516808	neutrophi	TRUE	reported	igd	0.000221	114.364	2	TRUE
rs4143314	A	T	A	T	-0.03459	-0.00081	0.042937	0.038275	FALSE	TRUE	FALSE	I9_PULMEN	0.983556	0.039225	pulmonary	TRUE	reported	textfile	22	17569993	0.004882	1.45E-12	519284	neutrophi	TRUE	reported	igd	9.67E-05	50.20002	2	TRUE
rs4145952	A	C	A	C	-0.01333	0.014396	0.399341	0.347736	FALSE	FALSE	FALSE	I9_PULMEN	0.361816	0.015787	pulmonary	TRUE	reported	textfile	4	1.2E+08	0.001959	1.06E-11	519287	neutrophi	TRUE	reported	igd	8.91E-05	46.27315	2	TRUE
rs4276676	C	T	C	T	-0.01801	0.003631	0.470378	0.371537	FALSE	FALSE	FALSE	I9_PULMEN	0.815079	0.015526	pulmonary	TRUE	reported	textfile	8	68795618	0.00193	1.16E-20	519285	neutrophi	TRUE	reported	igd	0.000168	87.0302	2	TRUE
rs4347951	C	T	C	T	0.011766	0.026126	0.310834	0.253458	FALSE	FALSE	FALSE	I9_PULMEN	0.130512	0.017278	pulmonary	TRUE	reported	textfile	22	29513326	0.002091	1.87E-08	519282	neutrophi	TRUE	reported	igd	6.10E-05	31.6627	2	TRUE
rs4413892	A	G	A	G	0.033654	0.000505	0.277885	0.252654	FALSE	FALSE	FALSE	I9_PULMEN	0.976676	0.017261	pulmonary	TRUE	reported	textfile	9	1.39E+08	0.002228	1.65E-51	493528	neutrophi	TRUE	reported	igd	0.000462	228.1609	2	TRUE
rs4468717	T	C	T	C	-0.02498	0.024196	0.077663	0.138231	FALSE	FALSE	FALSE	I9_PULMEN	0.274114	0.022124	pulmonary	TRUE	reported	textfile	18	3457606	0.003608	4.59E-12	517887	neutrophi	TRUE	reported	igd	9.25E-05	47.91936	2	TRUE
rs4535497	A	C	A	C	-0.01439	0.006775	0.569887	0.61981	FALSE	FALSE	FALSE	I9_PULMEN	0.661791	0.015488	pulmonary	TRUE	reported	textfile	5	1107428	0.002038	1.75E-12	472977	neutrophi	TRUE	reported	igd	0.000105	49.82759	2	TRUE
rs4557713	G	A	G	A	0.036447	-0.02532	0.04513	0.071722	FALSE	FALSE	FALSE	I9_PULMEN	0.390064	0.029461	pulmonary	TRUE	reported	textfile	8	48651633	0.005206	2.63E-12	468556	neutrophi	TRUE	reported	igd	0.000105	49.01324	2	TRUE
rs4626924	T	C	T	C	0.019408	-0.00161	0.551299	0.643478	FALSE	FALSE	FALSE	I9_PULMEN	0.918635	0.015728	pulmonary	TRUE	reported	textfile	1	2.35E+08	0.001963	4.99E-23	517875	neutrophi	TRUE	reported	igd	0.000189	97.75057	2	TRUE
rs4632345	A	G	A	G	-0.0122	-0.00167	0.677982	0.641838	FALSE	FALSE	FALSE	I9_PULMEN	0.91516	0.01564	pulmonary	TRUE	reported	textfile	2	16702654	0.00206	3.24E-09	518345	neutrophi	TRUE	reported	igd	6.76E-05	35.06811	2	TRUE
rs464609	A	G	A	G	0.010683	0.019263	0.544404	0.54392	FALSE	FALSE	FALSE	I9_PULMEN	0.200341	0.015042	pulmonary	TRUE	reported	textfile	5	34654477	0.001927	3.02E-08	517885	neutrophi	TRUE	reported	igd	5.93E-05	30.73416	2	TRUE
rs4727392	G	A	G	A	-0.01763	0.014553	0.736446	0.776061	FALSE	FALSE	FALSE	I9_PULMEN	0.420774	0.018077	pulmonary	TRUE	reported	textfile	7	97977670	0.002176	5.65E-16	519280	neutrophi	TRUE	reported	igd	0.000126	65.62762	2	TRUE
rs4729046	C	T	C	T	-0.04277	-0.00382	0.92416	0.913171	FALSE	FALSE	FALSE	I9_PULMEN	0.886431	0.026715	pulmonary	TRUE	reported	textfile	7	92223957	0.003637	6.84E-32	519288	neutrophi	TRUE	reported	igd	0.000266	138.3028	2	TRUE
rs47341	T	C	T	C	0.013023	0.000456	0.395885	0.370223	FALSE	FALSE	FALSE	I9_PULMEN	0.976585	0.01553	pulmonary	TRUE	reported	textfile	22	43560763	0.002052	2.26E-10	473786	neutrophi	TRUE	reported	igd	8.50E-05	40.27778	2	TRUE
rs4734879	G	A	G	A	-0.02134	-0.03689	0.276452	0.221988	FALSE	FALSE	FALSE	I9_PULMEN	0.042416	0.01818	pulmonary	TRUE	reported	textfile	8	1.07E+08	0.002144	2.63E-23	517889	neutrophi	TRUE	reported	igd	0.000191	99.06896	2	TRUE
rs4760	G	A	G	A	-0.07521	0.019451	0.153372	0.167591	FALSE	FALSE	FALSE	I9_PULMEN	0.337875	0.020296	pulmonary	TRUE	reported	textfile	19	44153100	0.002693	########	517886	neutrophi	TRUE	reported	igd	0.001504	780.0302	2	TRUE
rs4761234	C	T	C	T	0.021599	0.015702	0.483663	0.412369	FALSE	FALSE	FALSE	I9_PULMEN	0.303279	0.015253	pulmonary	TRUE	reported	textfile	12	69732105	0.001928	4.25E-29	519285	neutrophi	TRUE	reported	igd	0.000242	125.5023	2	TRUE
rs4794820	G	A	G	A	0.076088	-0.00395	0.551375	0.52571	FALSE	FALSE	FALSE	I9_PULMEN	0.792914	0.015044	pulmonary	TRUE	reported	textfile	17	38089344	0.001942	########	518341	neutrophi	TRUE	reported	igd	0.002953	1535.084	2	TRUE
rs4805881	C	A	C	A	-0.01444	0.016882	0.665289	0.6578	FALSE	FALSE	FALSE	I9_PULMEN	0.285299	0.0158	pulmonary	TRUE	reported	textfile	19	33896432	0.002068	3.05E-12	519287	neutrophi	TRUE	reported	igd	9.38E-05	48.72264	2	TRUE

rs4812447	G	A	G	A	0.017142	0.002797	0.440364	0.434339	FALSE	FALSE	FALSE	I9_PULMEM	0.853504	0.015148	pulmonary	TRUE	reported	textfile	20	39272620	0.001949	1.49E-18	519283	neutrophi	TRUE	reported	igd	0.000149	77.35665	2	TRUE
rs4843073	T	C	T	C	0.013665	0.010369	0.644889	0.52539	FALSE	FALSE	FALSE	I9_PULMEM	0.490686	0.015044	pulmonary	TRUE	reported	textfile	15	86124419	0.002007	1.01E-11	518339	neutrophi	TRUE	reported	igd	8.94E-05	46.3578	2	TRUE
rs4844622	T	C	T	C	-0.02338	-0.02817	0.240327	0.22366	FALSE	FALSE	FALSE	I9_PULMEM	0.117343	0.017987	pulmonary	TRUE	reported	textfile	1	2.08E+08	0.002236	1.44E-25	519288	neutrophi	TRUE	reported	igd	0.00021	109.3218	2	TRUE
rs486650	T	C	T	C	-0.01389	-0.00766	0.469271	0.419592	FALSE	FALSE	FALSE	I9_PULMEM	0.613991	0.015186	pulmonary	TRUE	reported	textfile	1	2.35E+08	0.001923	5.19E-13	517885	neutrophi	TRUE	reported	igd	0.000101	52.18784	2	TRUE
rs4903580	T	C	T	C	0.016519	0.016446	0.456084	0.458954	FALSE	FALSE	FALSE	I9_PULMEM	0.27371	0.015025	pulmonary	TRUE	reported	textfile	14	77850978	0.001929	1.16E-17	519283	neutrophi	TRUE	reported	igd	0.000141	73.33333	2	TRUE
rs4909932	G	A	G	A	0.013425	0.006243	0.597552	0.570502	FALSE	FALSE	FALSE	I9_PULMEM	0.680359	0.015153	pulmonary	TRUE	reported	textfile	11	10475967	0.001963	8.36E-12	516804	neutrophi	TRUE	reported	igd	9.05E-05	46.77204	2	TRUE
rs4924450	A	G	A	G	0.013811	-0.0121	0.70545	0.78409	FALSE	FALSE	FALSE	I9_PULMEM	0.510632	0.018387	pulmonary	TRUE	reported	textfile	15	40597229	0.002198	3.40E-10	475807	neutrophi	TRUE	reported	igd	8.30E-05	39.48145	2	TRUE
rs4925756	T	C	T	C	-0.013	0.011622	0.691891	0.721822	FALSE	FALSE	FALSE	I9_PULMEM	0.489822	0.016828	pulmonary	TRUE	reported	textfile	1	2.48E+08	0.002091	5.14E-10	518202	neutrophi	TRUE	reported	igd	7.46E-05	38.66434	2	TRUE
rs4984803	A	G	A	G	-0.01752	0.013233	0.595619	0.666783	FALSE	FALSE	FALSE	I9_PULMEM	0.405497	0.015908	pulmonary	TRUE	reported	textfile	16	1349929	0.00205	1.34E-17	473309	neutrophi	TRUE	reported	igd	0.000154	73.06466	2	TRUE
rs520910	C	T	C	T	-0.02241	-0.01785	0.066787	0.053213	FALSE	FALSE	FALSE	I9_PULMEM	0.594918	0.033562	pulmonary	TRUE	reported	textfile	1	1.56E+08	0.003861	6.63E-09	519284	neutrophi	TRUE	reported	igd	6.49E-05	33.70059	2	TRUE
rs5572910	G	A	G	A	-0.01751	0.046459	0.225567	0.214436	FALSE	FALSE	FALSE	I9_PULMEM	0.011169	0.01831	pulmonary	TRUE	reported	textfile	2	2.33E+08	0.002341	7.87E-14	518335	neutrophi	TRUE	reported	igd	0.000108	55.91389	2	TRUE
rs5576780	C	T	C	T	-0.01145	-0.01359	0.633688	0.598946	FALSE	FALSE	FALSE	I9_PULMEM	0.374752	0.01531	pulmonary	TRUE	reported	textfile	17	17674967	0.002082	3.87E-08	475807	neutrophi	TRUE	reported	igd	6.36E-05	30.25516	2	TRUE
rs5585139	A	C	A	C	0.012404	-0.01674	0.364781	0.375975	FALSE	FALSE	FALSE	I9_PULMEM	0.279222	0.015472	pulmonary	TRUE	reported	textfile	1	1.09E+08	0.002038	1.18E-09	517875	neutrophi	TRUE	reported	igd	7.15E-05	37.04362	2	TRUE
rs5587327	C	T	C	T	0.058951	-0.05131	0.020243	0.019804	FALSE	FALSE	FALSE	I9_PULMEM	0.341264	0.053917	pulmonary	TRUE	reported	textfile	1	36972428	0.006827	6.24E-18	519288	neutrophi	TRUE	reported	igd	0.000144	74.56257	2	TRUE
rs5596481	C	T	C	T	-0.03646	0.014137	0.567723	0.579086	FALSE	FALSE	FALSE	I9_PULMEM	0.352617	0.015209	pulmonary	TRUE	reported	textfile	8	1.31E+08	0.001948	3.94E-78	519282	neutrophi	TRUE	reported	igd	0.000674	350.3686	2	TRUE
rs5617417	C	T	C	T	0.016826	-0.01971	0.139347	0.105158	FALSE	FALSE	FALSE	I9_PULMEM	0.425189	0.024722	pulmonary	TRUE	reported	textfile	3	1.84E+08	0.002805	2.04E-09	519283	neutrophi	TRUE	reported	igd	6.93E-05	35.98275	2	TRUE
rs5618886	C	T	C	T	-0.02743	0.035779	0.373715	0.358899	FALSE	FALSE	FALSE	I9_PULMEM	0.022638	0.015696	pulmonary	TRUE	reported	textfile	1	2.48E+08	0.001984	1.93E-43	518206	neutrophi	TRUE	reported	igd	0.000369	191.1048	2	TRUE
rs5621714	A	G	A	G	0.017409	-0.01014	0.150714	0.170435	FALSE	FALSE	FALSE	I9_PULMEM	0.611613	0.019978	pulmonary	TRUE	reported	textfile	3	12962914	0.002789	4.45E-10	475805	neutrophi	TRUE	reported	igd	8.19E-05	38.96268	2	TRUE
rs5637871	G	A	G	A	0.129237	0.082825	0.012681	0.032123	FALSE	FALSE	FALSE	I9_PULMEM	0.05262	0.042737	pulmonary	TRUE	reported	textfile	17	56356502	0.008674	3.72E-50	518824	neutrophi	TRUE	reported	igd	0.000428	221.9899	2	TRUE
rs5638817	T	G	T	G	0.070039	-0.01178	0.293956	0.283397	FALSE	FALSE	FALSE	I9_PULMEM	0.4813	0.016731	pulmonary	TRUE	reported	textfile	7	28724374	0.002132	########	518201	neutrophi	TRUE	reported	igd	0.002078	1079.205	2	TRUE
rs5640811	C	T	C	T	0.031091	-0.01121	0.083714	0.057858	FALSE	FALSE	FALSE	I9_PULMEM	0.727684	0.032188	pulmonary	TRUE	reported	textfile	19	19793545	0.003521	1.11E-18	518349	neutrophi	TRUE	reported	igd	0.00015	77.97146	2	TRUE
rs567385	C	G	C	G	-0.01073	-0.02119	0.610373	0.619115	FALSE	TRUE	FALSE	I9_PULMEM	0.171885	0.015514	pulmonary	TRUE	reported	textfile	11	1.26E+08	0.001967	4.99E-08	519283	neutrophi	TRUE	reported	igd	5.73E-05	29.76254	2	TRUE
rs571497	A	G	A	G	-0.03793	0.035427	0.154629	0.124651	FALSE	FALSE	FALSE	I9_PULMEM	0.119376	0.022748	pulmonary	TRUE	reported	textfile	19	7827830	0.002701	9.76E-45	516807	neutrophi	TRUE	reported	igd	0.000381	197.1828	2	TRUE
rs5747308	C	A	C	A	0.022058	-0.00742	0.504471	0.497656	FALSE	FALSE	FALSE	I9_PULMEM	0.620366	0.014983	pulmonary	TRUE	reported	textfile	22	18133500	0.001925	2.30E-30	519277	neutrophi	TRUE	reported	igd	0.000253	131.3013	2	TRUE
rs5753576	T	C	T	C	-0.01785	0.014201	0.480292	0.555715	FALSE	FALSE	FALSE	I9_PULMEM	0.3471	0.015104	pulmonary	TRUE	reported	textfile	22	31757735	0.00193	2.43E-20	519283	neutrophi	TRUE	reported	igd	0.000165	85.51904	2	TRUE
rs5800548	T	C	T	C	0.032744	-0.04828	0.143931	0.160438	FALSE	FALSE	FALSE	I9_PULMEM	0.017887	0.020391	pulmonary	TRUE	reported	textfile	6	29822432	0.002734	5.14E-33	518198	neutrophi	TRUE	reported	igd	0.000277	143.4382	2	TRUE
rs5860523	A	T	A	T	-0.0139	-0.00245	0.373119	0.38596	FALSE	TRUE	FALSE	I9_PULMEM	0.873156	0.015367	pulmonary	TRUE	reported	textfile	3	1.2E+08	0.001984	2.53E-12	519283	neutrophi	TRUE	reported	igd	9.45E-05	49.07746	2	TRUE
rs5898452	C	T	C	T	-0.01103	0.002888	0.346098	0.456613	FALSE	FALSE	FALSE	I9_PULMEM	0.847971	0.015065	pulmonary	TRUE	reported	textfile	11	43490624	0.002017	4.71E-08	519287	neutrophi	TRUE	reported	igd	5.76E-05	29.8883	2	TRUE
rs5951978	A	G	A	G	0.011747	0.011994	0.410141	0.42836	FALSE	FALSE	FALSE	I9_PULMEM	0.429099	0.015168	pulmonary	TRUE	reported	textfile	3	1.86E+08	0.001953	1.85E-09	519275	neutrophi	TRUE	reported	igd	6.97E-05	36.17827	2	TRUE
rs6012493	T	C	T	C	-0.01781	-0.01213	0.176181	0.156358	FALSE	FALSE	FALSE	I9_PULMEM	0.556607	0.020627	pulmonary	TRUE	reported	textfile	1	1.74E+08	0.002517	1.52E-12	519272	neutrophi	TRUE	reported	igd	9.65E-05	50.09043	2	TRUE
rs6067411	T	C	T	C	-0.01162	0.013033	0.528523	0.558896	FALSE	FALSE	FALSE	I9_PULMEM	0.393229	0.015265	pulmonary	TRUE	reported	textfile	20	48975411	0.00195	2.64E-09	518202	neutrophi	TRUE	reported	igd	6.85E-05	35.47869	2	TRUE
rs609264	G	T	G	T	0.012618	-0.00309	0.528467	0.499383	FALSE	FALSE	FALSE	I9_PULMEM	0.836462	0.014978	pulmonary	TRUE	reported	textfile	8	61548546	0.001939	7.93E-11	518205	neutrophi	TRUE	reported	igd	8.17E-05	42.34711	2	TRUE
rs610578	G	A	G	A	-0.01463	-0.01508	0.662713	0.642522	FALSE	FALSE	FALSE	I9_PULMEM	0.334741	0.015636	pulmonary	TRUE	reported	textfile	12	1.21E+08	0.002031	6.27E-13	517872	neutrophi	TRUE	reported	igd	0.0001	51.85256	2	TRUE
rs6173928	T	C	T	C	-0.04013	-0.02133	0.03348	0.05242	FALSE	FALSE	FALSE	I9_PULMEM	0.528354	0.033836	pulmonary	TRUE	reported	textfile	16	27480797	0.005402	1.15E-13	517886	neutrophi	TRUE	reported	igd	0.000107	55.18032	2	TRUE
rs6175423	T	C	T	C	0.039977	-0.09614	0.019363	0.005585	FALSE	FALSE	FALSE	I9_PULMEM	0.337325	0.100199	pulmonary	TRUE	reported	textfile	12	72179446	0.007114	1.96E-08	515996	neutrophi	TRUE	reported	igd	6.12E-05	31.57846	2	TRUE
rs619450	C	T	C	T	0.014214	0.000329	0.503233	0.541298	FALSE	FALSE	FALSE	I9_PULMEM	0.982578	0.015059	pulmonary	TRUE	reported	textfile	20	2894790	0.002002	1.29E-12	475806	neutrophi	TRUE	reported	igd	0.000106	50.40837	2	TRUE
rs6201128	A	G	A	G	0.012067	-0.04052	0.310021	0.327796	FALSE	FALSE	FALSE	I9_PULMEM	0.011357	0.016005	pulmonary	TRUE	reported	textfile	15	63781143	0.002082	7.03E-09	519284	neutrophi	TRUE	reported	igd	6.47E-05	33.59197	2	TRUE
rs6218985	C	T	C	T	0.014074	0.032675	0.244372	0.26019	FALSE	FALSE	FALSE	I9_PULMEM	0.056732	0.017149	pulmonary	TRUE	reported	textfile	2	1.82E+08	0.002232	2.96E-10	518349	neutrophi	TRUE	reported	igd	7.67E-05	39.75988	2	TRUE
rs6236018	G	A	G	A	-0.01595	-0.01422	0.190708	0.181967	FALSE	FALSE	FALSE	I9_PULMEM	0.464747	0.019445	pulmonary	TRUE	reported	textfile	5	57274612	0.002452	8.05E-11	519288	neutrophi	TRUE	reported	igd	8.14E-05	42.29757	2	TRUE
rs6237538	C	T	C	T	0.014183	0.022002	0.193932	0.170348	FALSE	FALSE	FALSE	I9_PULMEM	0.27148	0.020008	pulmonary	TRUE	reported	textfile	5	86268229	0.00243	5.46E-09	519285	neutrophi	TRUE	reported	igd	6.56E-05	34.06607	2	TRUE
rs6242998	A	G	A	G	0.016402	-0.00725	0.144922	0.225056	FALSE	FALSE	FALSE	I9_PULMEM	0.687436	0.018025	pulmonary	TRUE	reported	textfile	6	1.22E+08	0.002723	1.77E-09	519285	neutrophi	TRUE	reported	igd	6.99E-05	36.28245	2	TRUE
rs626416	C	G	C	G	-0.01969	0.007511	0.761969	0.809558	FALSE	TRUE	FALSE	I9_PULMEM	0.6941	0.019097	pulmonary	TRUE	reported	textfile	9	79326680	0.002278	5.77E-18	504400	neutrophi	TRUE	reported	igd	0.000148	74.67267	2	TRUE
rs632887	G	A	G	A	0.014283	0.016519	0.410714	0.385539	FALSE	FALSE	FALSE	I9_PULMEM	0.285524	0.015468	pulmonary	TRUE	reported	textfile	12	3392351	0.002004	1.06E-12	517882	neutrophi	TRUE	reported	igd	9.81E-05	50.79743	2	TRUE
rs636317	T	C	T	C	0.014297	-0.01819	0.591234	0.704659	FALSE	FALSE	FALSE	I9_PULMEM	0.269678	0.016475	pulmonary	TRUE	reported	textfile	11	60019150	0.00195	2.34E-13	519286	neutrophi	TRUE	reported	igd	0.000104	53.75501	2	TRUE

rs6414435	G	A	G	A	0.01709	-0.00099	0.591762	0.622981	FALSE	FALSE	FALSE	I9_PULMEN	0.948733	0.015465	pulmonary	TRUE	reported	textfile	3	47121140	0.002039	5.57E-17	519286	neutrophi	TRUE	reported	igd	0.000135	70.25027	2	TRUE
rs6432335	G	T	G	T	0.012236	-0.00199	0.451562	0.550453	FALSE	FALSE	FALSE	I9_PULMEN	0.895074	0.01509	pulmonary	TRUE	reported	textfile	2	12928971	0.001931	2.42E-10	515519	neutrophi	TRUE	reported	igd	7.79E-05	40.15251	2	TRUE
rs6463566	G	C	G	C	-0.017	0.004588	0.229674	0.266934	FALSE	TRUE	FALSE	I9_PULMEN	0.787109	0.016989	pulmonary	TRUE	reported	textfile	7	6528174	0.002314	2.16E-13	519282	neutrophi	TRUE	reported	igd	0.000104	53.95307	2	TRUE
rs6468341	C	T	C	T	0.018973	-0.01756	0.742846	0.709636	FALSE	FALSE	FALSE	I9_PULMEN	0.288527	0.016544	pulmonary	TRUE	reported	textfile	8	30279355	0.002201	7.10E-18	519283	neutrophi	TRUE	reported	igd	0.000143	74.30709	2	TRUE
rs6476883	A	G	A	G	-0.01401	-0.00471	0.438614	0.344588	FALSE	FALSE	FALSE	I9_PULMEN	0.765098	0.015778	pulmonary	TRUE	reported	textfile	9	4654774	0.001937	4.96E-13	519286	neutrophi	TRUE	reported	igd	0.000101	52.30622	2	TRUE
rs6500550	T	C	T	C	-0.02236	0.009223	0.304296	0.289731	FALSE	FALSE	FALSE	I9_PULMEN	0.576874	0.016529	pulmonary	TRUE	reported	textfile	16	3746241	0.00212	5.53E-26	517883	neutrophi	TRUE	reported	igd	0.000215	111.2722	2	TRUE
rs6503727	C	T	C	T	0.018796	-0.00733	0.660546	0.615019	FALSE	FALSE	FALSE	I9_PULMEN	0.635128	0.015455	pulmonary	TRUE	reported	textfile	17	41312859	0.002032	2.39E-20	519287	neutrophi	TRUE	reported	igd	0.000165	85.56217	2	TRUE
rs6672898	A	G	A	G	-0.01919	########	0.435247	0.421725	FALSE	FALSE	FALSE	I9_PULMEN	0.994838	0.015203	pulmonary	TRUE	reported	textfile	1	46290588	0.00193	2.92E-23	518344	neutrophi	TRUE	reported	igd	0.000191	98.88358	2	TRUE
rs6678033	A	G	A	G	-0.04271	0.007287	0.367919	0.460602	FALSE	FALSE	FALSE	I9_PULMEN	0.628695	0.01507	pulmonary	TRUE	reported	textfile	1	66077624	0.001986	########	517887	neutrophi	TRUE	reported	igd	0.000892	462.4431	2	TRUE
rs6679677	A	C	A	C	-0.03789	0.00222	0.100303	0.146443	FALSE	FALSE	FALSE	I9_PULMEN	0.917043	0.02131	pulmonary	TRUE	reported	textfile	1	1.14E+08	0.003175	8.69E-33	518827	neutrophi	TRUE	reported	igd	0.000274	142.3788	2	TRUE
rs6717590	T	C	T	C	0.043563	0.010744	0.107472	0.137132	FALSE	FALSE	FALSE	I9_PULMEN	0.621328	0.02175	pulmonary	TRUE	reported	textfile	15	1.02E+08	0.003151	1.99E-43	503462	neutrophi	TRUE	reported	igd	0.000379	191.1336	2	TRUE
rs671835	G	A	G	A	0.015013	0.009387	0.359974	0.456588	FALSE	FALSE	FALSE	I9_PULMEN	0.533641	0.01508	pulmonary	TRUE	reported	textfile	18	51734729	0.002003	6.86E-14	519282	neutrophi	TRUE	reported	igd	0.000108	56.17866	2	TRUE
rs6723009	C	T	C	T	0.012066	0.005534	0.717442	0.695974	FALSE	FALSE	FALSE	I9_PULMEN	0.73524	0.016366	pulmonary	TRUE	reported	textfile	2	42474615	0.002153	2.15E-08	518349	neutrophi	TRUE	reported	igd	6.06E-05	31.40776	2	TRUE
rs6731993	T	A	T	A	0.022846	-0.0036	0.408305	0.337415	FALSE	TRUE	FALSE	I9_PULMEN	0.821137	0.015911	pulmonary	TRUE	reported	textfile	2	65642097	0.001961	2.45E-31	519280	neutrophi	TRUE	reported	igd	0.000261	135.7261	2	TRUE
rs6734238	G	A	G	A	0.03936	-0.02805	0.40298	0.3032	FALSE	FALSE	FALSE	I9_PULMEN	0.086286	0.016353	pulmonary	TRUE	reported	textfile	2	1.14E+08	0.00195	1.58E-90	517886	neutrophi	TRUE	reported	igd	0.000786	407.4171	2	TRUE
rs6740847	G	A	G	A	0.021748	-0.01951	0.562056	0.59925	FALSE	FALSE	FALSE	I9_PULMEN	0.202405	0.015302	pulmonary	TRUE	reported	textfile	2	1.82E+08	0.00194	3.95E-29	517889	neutrophi	TRUE	reported	igd	0.000243	125.6705	2	TRUE
rs6764912	A	G	A	G	-0.03725	-0.01513	0.065313	0.085378	FALSE	FALSE	FALSE	I9_PULMEN	0.574238	0.026937	pulmonary	TRUE	reported	textfile	3	1.28E+08	0.004039	3.14E-20	517423	neutrophi	TRUE	reported	igd	0.000164	85.03282	2	TRUE
rs6779340	G	C	G	C	-0.01525	0.021879	0.336877	0.29803	FALSE	TRUE	FALSE	I9_PULMEN	0.183163	0.016437	pulmonary	TRUE	reported	textfile	3	58033701	0.002042	8.69E-14	518346	neutrophi	TRUE	reported	igd	0.000108	55.75138	2	TRUE
rs6800256	G	A	G	A	0.020207	-0.06605	0.091374	0.058405	FALSE	FALSE	FALSE	I9_PULMEN	0.040325	0.032212	pulmonary	TRUE	reported	textfile	1	1.5E+08	0.003384	2.43E-09	519284	neutrophi	TRUE	reported	igd	6.87E-05	35.65671	2	TRUE
rs6801638	T	C	T	C	-0.03378	0.020038	0.051299	0.041956	FALSE	FALSE	FALSE	I9_PULMEN	0.594404	0.037632	pulmonary	TRUE	reported	textfile	6	43761645	0.00442	2.23E-14	519284	neutrophi	TRUE	reported	igd	0.000112	58.41847	2	TRUE
rs6813703	G	A	G	A	-0.01305	0.000634	0.285148	0.305864	FALSE	FALSE	FALSE	I9_PULMEN	0.969001	0.016322	pulmonary	TRUE	reported	textfile	6	43820215	0.002135	1.02E-09	518344	neutrophi	TRUE	reported	igd	7.21E-05	37.34999	2	TRUE
rs6817881	T	C	T	C	0.014302	0.008983	0.499266	0.481054	FALSE	FALSE	FALSE	I9_PULMEN	0.550325	0.01504	pulmonary	TRUE	reported	textfile	4	1.52E+08	0.001931	1.35E-13	518344	neutrophi	TRUE	reported	igd	0.000106	54.8564	2	TRUE
rs6831590	T	G	T	G	-0.02038	-0.03694	0.367295	0.392018	FALSE	FALSE	FALSE	I9_PULMEN	0.016185	0.015363	pulmonary	TRUE	reported	textfile	4	6943776	0.001987	1.15E-24	519283	neutrophi	TRUE	reported	igd	0.000203	105.2195	2	TRUE
rs6855981	A	G	A	G	-0.01637	-0.02846	0.372933	0.4635	FALSE	FALSE	FALSE	I9_PULMEN	0.059319	0.01509	pulmonary	TRUE	reported	textfile	4	3148276	0.001987	1.85E-16	517885	neutrophi	TRUE	reported	igd	0.000131	67.85687	2	TRUE
rs6859727	C	T	C	T	-0.04036	-0.01532	0.878025	0.875915	FALSE	FALSE	FALSE	I9_PULMEN	0.50005	0.022714	pulmonary	TRUE	reported	textfile	5	71742622	0.002931	4.29E-43	519287	neutrophi	TRUE	reported	igd	0.000365	189.6134	2	TRUE
rs6878780	C	T	C	T	0.013346	-0.02481	0.426457	0.528423	FALSE	FALSE	FALSE	I9_PULMEN	0.099156	0.015044	pulmonary	TRUE	reported	textfile	5	1.22E+08	0.001938	6.01E-12	519264	neutrophi	TRUE	reported	igd	9.13E-05	47.42344	2	TRUE
rs6891328	C	G	C	G	0.012329	-0.03151	0.583149	0.647913	FALSE	TRUE	FALSE	I9_PULMEN	0.045067	0.015724	pulmonary	TRUE	reported	textfile	5	1.34E+08	0.001947	2.47E-10	519288	neutrophi	TRUE	reported	igd	7.72E-05	40.09795	2	TRUE
rs6915310	T	C	T	C	-0.03231	-0.01398	0.173979	0.139227	FALSE	FALSE	FALSE	I9_PULMEN	0.519668	0.021716	pulmonary	TRUE	reported	textfile	6	16758002	0.00256	1.71E-36	516445	neutrophi	TRUE	reported	igd	0.000308	159.3111	2	TRUE
rs6924387	G	A	G	A	0.017269	-0.02456	0.412169	0.432156	FALSE	FALSE	FALSE	I9_PULMEN	0.106464	0.015212	pulmonary	TRUE	reported	textfile	6	1.37E+08	0.001967	1.73E-18	519284	neutrophi	TRUE	reported	igd	0.000148	77.07686	2	TRUE
rs6927569	C	T	C	T	0.025882	-0.00186	0.523171	0.616858	FALSE	FALSE	FALSE	I9_PULMEN	0.904586	0.015489	pulmonary	TRUE	reported	textfile	6	1.1E+08	0.001921	2.40E-41	519285	neutrophi	TRUE	reported	igd	0.000349	181.5262	2	TRUE
rs6936191	C	T	C	T	0.018088	-0.0213	0.608046	0.601332	FALSE	FALSE	FALSE	I9_PULMEN	0.167145	0.015417	pulmonary	TRUE	reported	textfile	6	7201660	0.001988	9.87E-20	518347	neutrophi	TRUE	reported	igd	0.00016	82.78405	2	TRUE
rs694180	G	A	G	A	0.018797	-0.01341	0.656276	0.674086	FALSE	FALSE	FALSE	I9_PULMEN	0.401595	0.01599	pulmonary	TRUE	reported	textfile	1	1.12E+08	0.002017	1.24E-20	516807	neutrophi	TRUE	reported	igd	0.000168	86.84876	2	TRUE
rs6985508	A	G	A	G	-0.02738	-0.00872	0.357978	0.369326	FALSE	FALSE	FALSE	I9_PULMEN	0.574115	0.015524	pulmonary	TRUE	reported	textfile	8	1.42E+08	0.002089	3.36E-39	474407	neutrophi	TRUE	reported	igd	0.000362	171.736	2	TRUE
rs6998846	A	G	A	G	0.01538	-0.04248	0.782787	0.732807	FALSE	FALSE	FALSE	I9_PULMEN	0.012278	0.016963	pulmonary	TRUE	reported	textfile	8	9193341	0.002334	4.54E-11	518207	neutrophi	TRUE	reported	igd	8.38E-05	43.42195	2	TRUE
rs7005996	T	C	T	C	0.022897	-0.02985	0.905961	0.900881	FALSE	FALSE	FALSE	I9_PULMEN	0.237152	0.025253	pulmonary	TRUE	reported	textfile	8	1.42E+08	0.00345	3.33E-11	475806	neutrophi	TRUE	reported	igd	9.26E-05	44.04708	2	TRUE
rs706809	C	T	C	T	0.02055	0.008932	0.777232	0.796839	FALSE	FALSE	FALSE	I9_PULMEN	0.633524	0.018735	pulmonary	TRUE	reported	textfile	12	52294257	0.002355	2.78E-18	518347	neutrophi	TRUE	reported	igd	0.000147	76.14478	2	TRUE
rs7183988	G	T	G	T	-0.01703	-0.01491	0.526501	0.592467	FALSE	FALSE	FALSE	I9_PULMEN	0.328417	0.01526	pulmonary	TRUE	reported	textfile	15	91428589	0.001973	6.26E-18	518205	neutrophi	TRUE	reported	igd	0.000144	74.53795	2	TRUE
rs7214290	A	G	A	G	0.018062	-0.00818	0.172996	0.173415	FALSE	FALSE	FALSE	I9_PULMEN	0.680129	0.01984	pulmonary	TRUE	reported	textfile	17	27611880	0.00254	1.20E-12	517881	neutrophi	TRUE	reported	igd	9.76E-05	50.56646	2	TRUE
rs7225843	C	T	C	T	-0.02208	0.020132	0.203156	0.242343	FALSE	FALSE	FALSE	I9_PULMEN	0.250579	0.017523	pulmonary	TRUE	reported	textfile	17	2001825	0.002391	2.69E-20	519286	neutrophi	TRUE	reported	igd	0.000164	85.30124	2	TRUE
rs723585	G	A	G	A	-0.02228	0.017829	0.483522	0.46907	FALSE	FALSE	FALSE	I9_PULMEN	0.237315	0.015087	pulmonary	TRUE	reported	textfile	4	55503194	0.001915	2.97E-31	519286	neutrophi	TRUE	reported	igd	0.000261	135.3967	2	TRUE
rs7235882	T	C	T	C	0.02332	-0.03344	0.093478	0.069846	FALSE	FALSE	FALSE	I9_PULMEN	0.257111	0.029507	pulmonary	TRUE	reported	textfile	18	19684119	0.003325	2.40E-12	518346	neutrophi	TRUE	reported	igd	9.49E-05	49.18947	2	TRUE
rs7237878	A	G	A	G	-0.01184	-0.00122	0.68994	0.730758	FALSE	FALSE	FALSE	I9_PULMEN	0.942737	0.016978	pulmonary	TRUE	reported	textfile	18	74249473	0.00209	1.53E-08	518183	neutrophi	TRUE	reported	igd	6.19E-05	32.07123	2	TRUE
rs7266484	T	C	T	C	0.020857	-0.00855	0.178952	0.12859	FALSE	FALSE	FALSE	I9_PULMEN	0.702839	0.022418	pulmonary	TRUE	reported	textfile	14	35596323	0.002507	9.21E-17	519279	neutrophi	TRUE	reported	igd	0.000133	69.2139	2	TRUE
rs7272602	C	T	C	T	-0.04322	0.044881	0.11173	0.075539	FALSE	FALSE	FALSE	I9_PULMEN	0.114731	0.028455	pulmonary	TRUE	reported	textfile	15	42248826	0.003051	1.71E-45	518349	neutrophi	TRUE	reported	igd	0.000387	200.6519	2	TRUE

rs7273156	T	C	T	C	0.020208	0.009306	0.187005	0.172248	FALSE	FALSE	FALSE	I9_PULMEM	0.639015	0.019839	pulmonary	TRUE	reported	textfile	14	69280158	0.00247	2.95E-16	519285	neutrophi	TRUE	reported	igd	0.000129	66.93466	2	TRUE
rs7278992	C	A	C	A	0.020456	0.010065	0.307359	0.262905	FALSE	FALSE	FALSE	I9_PULMEM	0.553669	0.016994	pulmonary	TRUE	reported	textfile	2	24394504	0.002076	6.95E-23	518346	neutrophi	TRUE	reported	igd	0.000187	97.09236	2	TRUE
rs7279086	C	T	C	T	-0.02103	0.02938	0.307083	0.296648	FALSE	FALSE	FALSE	I9_PULMEM	0.073276	0.016403	pulmonary	TRUE	reported	textfile	10	44880260	0.002085	6.71E-24	519284	neutrophi	TRUE	reported	igd	0.000196	101.7434	2	TRUE
rs7280332	T	C	T	C	0.034499	0.010251	0.039743	0.054168	FALSE	FALSE	FALSE	I9_PULMEM	0.757986	0.033269	pulmonary	TRUE	reported	textfile	16	89373324	0.004957	3.56E-12	517879	neutrophi	TRUE	reported	igd	9.35E-05	48.43658	2	TRUE
rs7282530	G	A	G	A	0.015083	-0.02523	0.167458	0.144461	FALSE	FALSE	FALSE	I9_PULMEM	0.238212	0.021394	pulmonary	TRUE	reported	textfile	10	93748263	0.002571	4.56E-09	519287	neutrophi	TRUE	reported	igd	6.63E-05	34.41673	2	TRUE
rs7283205	A	G	A	G	-0.01235	-0.03491	0.313384	0.389727	FALSE	FALSE	FALSE	I9_PULMEM	0.023172	0.015375	pulmonary	TRUE	reported	textfile	16	81869387	0.002082	3.09E-09	519282	neutrophi	TRUE	reported	igd	6.77E-05	35.18037	2	TRUE
rs7283662	T	C	T	C	-0.01545	-0.02295	0.248064	0.290466	FALSE	FALSE	FALSE	I9_PULMEM	0.165778	0.016562	pulmonary	TRUE	reported	textfile	10	1.14E+08	0.002227	4.07E-12	518199	neutrophi	TRUE	reported	igd	9.29E-05	48.14858	2	TRUE
rs7283930	T	C	T	C	-0.01273	0.006042	0.739014	0.809842	FALSE	FALSE	FALSE	I9_PULMEM	0.751583	0.019086	pulmonary	TRUE	reported	textfile	21	30461373	0.0022	7.43E-09	519279	neutrophi	TRUE	reported	igd	6.44E-05	33.45558	2	TRUE
rs7290175	C	G	C	G	-0.01453	0.000996	0.326884	0.282067	FALSE	TRUE	FALSE	I9_PULMEM	0.95241	0.016695	pulmonary	TRUE	reported	textfile	17	76244283	0.002142	1.23E-11	474718	neutrophi	TRUE	reported	igd	9.69E-05	45.98872	2	TRUE
rs7297371	T	A	T	A	-0.03648	0.051523	0.06693	0.058737	FALSE	TRUE	FALSE	I9_PULMEM	0.107947	0.032052	pulmonary	TRUE	reported	textfile	18	74072245	0.003879	5.53E-21	518202	neutrophi	TRUE	reported	igd	0.000171	88.45856	2	TRUE
rs7297875	C	T	C	T	-0.0256	0.013826	0.065785	0.099397	FALSE	FALSE	FALSE	I9_PULMEM	0.582449	0.025147	pulmonary	TRUE	reported	textfile	6	1.38E+08	0.003899	5.38E-11	519285	neutrophi	TRUE	reported	igd	8.30E-05	43.11275	2	TRUE
rs7300096	A	T	A	T	0.029201	-0.01657	0.315728	0.375965	FALSE	TRUE	FALSE	I9_PULMEM	0.287892	0.015591	pulmonary	TRUE	reported	textfile	11	1.14E+08	0.002076	6.63E-45	519286	neutrophi	TRUE	reported	igd	0.000381	197.8514	2	TRUE
rs7304927	A	G	A	G	-0.03953	0.020802	0.058516	0.026555	FALSE	FALSE	FALSE	I9_PULMEM	0.654871	0.046536	pulmonary	TRUE	reported	textfile	7	8022016	0.004095	5.13E-22	518347	neutrophi	TRUE	reported	igd	0.00018	93.1846	2	TRUE
rs7322886	C	T	C	T	0.013244	-0.00914	0.623891	0.484066	FALSE	FALSE	FALSE	I9_PULMEM	0.542849	0.015019	pulmonary	TRUE	reported	textfile	13	50179454	0.001987	2.71E-11	519285	neutrophi	TRUE	reported	igd	8.55E-05	44.42638	2	TRUE
rs7327139	C	T	C	T	0.0255	0.015845	0.045484	0.040271	FALSE	FALSE	FALSE	I9_PULMEM	0.679207	0.038314	pulmonary	TRUE	reported	textfile	8	1.01E+08	0.004616	3.39E-08	519287	neutrophi	TRUE	reported	igd	5.88E-05	30.51737	2	TRUE
rs7407632	C	T	C	T	-0.0387	-0.048	0.044994	0.019347	FALSE	FALSE	FALSE	I9_PULMEM	0.378813	0.054538	pulmonary	TRUE	reported	textfile	1	56979431	0.004641	7.85E-17	519285	neutrophi	TRUE	reported	igd	0.000134	69.54477	2	TRUE
rs7409459	T	C	T	C	0.013123	-0.04509	0.29703	0.283966	FALSE	FALSE	FALSE	I9_PULMEM	0.007471	0.016854	pulmonary	TRUE	reported	textfile	19	7792199	0.002231	4.15E-09	518197	neutrophi	TRUE	reported	igd	6.68E-05	34.59915	2	TRUE
rs7488780	C	G	C	G	-0.01367	-0.03164	0.204545	0.254512	FALSE	TRUE	FALSE	I9_PULMEM	0.067194	0.017285	pulmonary	TRUE	reported	textfile	12	20579392	0.002391	1.12E-08	519284	neutrophi	TRUE	reported	igd	6.29E-05	32.66318	2	TRUE
rs749780	A	C	A	C	0.018874	0.006634	0.731874	0.65993	FALSE	FALSE	FALSE	I9_PULMEM	0.675683	0.015856	pulmonary	TRUE	reported	textfile	17	72699384	0.002179	4.94E-18	518203	neutrophi	TRUE	reported	igd	0.000145	75.026	2	TRUE
rs7547562	G	C	G	C	0.033029	0.003751	0.076758	0.088127	FALSE	TRUE	FALSE	I9_PULMEM	0.888162	0.026676	pulmonary	TRUE	reported	textfile	2	54787592	0.00369	3.76E-19	519287	neutrophi	TRUE	reported	igd	0.000154	80.11917	2	TRUE
rs7555995	G	C	G	C	-0.0149	-0.01634	0.284874	0.25046	FALSE	TRUE	FALSE	I9_PULMEM	0.345368	0.01732	pulmonary	TRUE	reported	textfile	1	1.5E+08	0.002132	2.86E-12	519283	neutrophi	TRUE	reported	igd	9.41E-05	48.85545	2	TRUE
rs7575465	A	G	A	G	-0.01273	0.02495	0.553396	0.600204	FALSE	FALSE	FALSE	I9_PULMEM	0.104782	0.015381	pulmonary	TRUE	reported	textfile	2	27291661	0.001942	5.71E-11	518344	neutrophi	TRUE	reported	igd	8.29E-05	42.9894	2	TRUE
rs7590263	T	C	T	C	0.01172	-0.00102	0.496568	0.519498	FALSE	FALSE	FALSE	I9_PULMEM	0.945917	0.015031	pulmonary	TRUE	reported	textfile	2	28645802	0.001946	1.76E-09	517888	neutrophi	TRUE	reported	igd	7.00E-05	36.2717	2	TRUE
rs7597441	T	C	T	C	-0.01657	0.010781	0.211414	0.195592	FALSE	FALSE	FALSE	I9_PULMEM	0.569053	0.018932	pulmonary	TRUE	reported	textfile	16	57063494	0.002361	2.32E-12	519280	neutrophi	TRUE	reported	igd	9.49E-05	49.2789	2	TRUE
rs763362	G	A	G	A	0.015565	0.002358	0.398747	0.318136	FALSE	FALSE	FALSE	I9_PULMEM	0.883703	0.016121	pulmonary	TRUE	reported	textfile	18	67531797	0.001962	2.23E-15	519288	neutrophi	TRUE	reported	igd	0.000121	62.93592	2	TRUE
rs7639292	T	C	T	C	-0.02227	-0.00153	0.169103	0.118962	FALSE	FALSE	FALSE	I9_PULMEM	0.947378	0.023201	pulmonary	TRUE	reported	textfile	3	1.07E+08	0.00257	4.83E-18	519283	neutrophi	TRUE	reported	igd	0.000145	75.07486	2	TRUE
rs7642810	C	T	C	T	0.104475	-0.10983	0.013438	0.012756	FALSE	FALSE	FALSE	I9_PULMEM	0.108723	0.068471	pulmonary	TRUE	reported	textfile	13	28604007	0.008878	6.25E-32	519286	neutrophi	TRUE	reported	igd	0.000267	138.4818	2	TRUE
rs7658676	T	C	T	C	0.011122	0.015123	0.353026	0.320109	FALSE	FALSE	FALSE	I9_PULMEM	0.348014	0.016114	pulmonary	TRUE	reported	textfile	4	39603132	0.002017	3.58E-08	516807	neutrophi	TRUE	reported	igd	5.88E-05	30.40551	2	TRUE
rs7660368	A	G	A	G	0.040772	0.085847	0.03196	0.028679	FALSE	FALSE	FALSE	I9_PULMEM	0.061624	0.045932	pulmonary	TRUE	reported	textfile	13	28496468	0.005573	2.65E-13	518346	neutrophi	TRUE	reported	igd	0.000103	53.52347	2	TRUE
rs7679673	A	C	A	C	-0.02382	0.004507	0.379766	0.488889	FALSE	FALSE	FALSE	I9_PULMEM	0.764466	0.015041	pulmonary	TRUE	reported	textfile	4	1.06E+08	0.001977	2.15E-33	517887	neutrophi	TRUE	reported	igd	0.00028	145.216	2	TRUE
rs7684253	T	C	T	C	0.014152	-0.0339	0.550374	0.535189	FALSE	FALSE	FALSE	I9_PULMEM	0.024679	0.015091	pulmonary	TRUE	reported	textfile	4	57727311	0.001942	3.29E-13	517886	neutrophi	TRUE	reported	igd	0.000103	53.10501	2	TRUE
rs7687347	A	C	A	C	-0.01512	-0.003	0.155842	0.29155	FALSE	FALSE	FALSE	I9_PULMEM	0.856382	0.016596	pulmonary	TRUE	reported	textfile	7	75829199	0.002649	1.18E-08	519283	neutrophi	TRUE	reported	igd	6.27E-05	32.57471	2	TRUE
rs7687658	G	A	G	A	0.011065	-0.00579	0.434196	0.338423	FALSE	FALSE	FALSE	I9_PULMEM	0.715066	0.015859	pulmonary	TRUE	reported	textfile	4	88220509	0.001937	1.14E-08	519282	neutrophi	TRUE	reported	igd	6.28E-05	32.63187	2	TRUE
rs7705526	A	C	A	C	0.033578	0.004212	0.327035	0.323917	FALSE	FALSE	FALSE	I9_PULMEM	0.793127	0.01606	pulmonary	TRUE	reported	textfile	5	1285974	0.002174	9.19E-54	470937	neutrophi	TRUE	reported	igd	0.000506	238.5551	2	TRUE
rs7738554	A	T	A	T	0.014527	-0.01183	0.250954	0.266757	FALSE	TRUE	FALSE	I9_PULMEM	0.484576	0.016927	pulmonary	TRUE	reported	textfile	6	41891995	0.002216	5.70E-11	517783	neutrophi	TRUE	reported	igd	8.30E-05	42.97449	2	TRUE
rs7755226	A	G	A	G	0.048554	0.051066	0.077954	0.033888	FALSE	FALSE	FALSE	I9_PULMEM	0.219074	0.041551	pulmonary	TRUE	reported	textfile	2	43786818	0.00358	7.19E-42	519287	neutrophi	TRUE	reported	igd	0.000354	183.9426	2	TRUE
rs7776857	T	G	T	G	-0.01483	0.016337	0.657131	0.593381	FALSE	FALSE	FALSE	I9_PULMEM	0.285956	0.015311	pulmonary	TRUE	reported	textfile	7	22754768	0.002034	3.16E-13	519283	neutrophi	TRUE	reported	igd	0.000102	53.18075	2	TRUE
rs7777484	G	A	G	A	0.011681	-0.02079	0.420278	0.450596	FALSE	FALSE	FALSE	I9_PULMEM	0.167216	0.015054	pulmonary	TRUE	reported	textfile	7	2814271	0.001945	1.96E-09	518348	neutrophi	TRUE	reported	igd	6.96E-05	36.06776	2	TRUE
rs778732	T	C	T	C	0.026848	-0.00497	0.107318	0.0636	FALSE	FALSE	FALSE	I9_PULMEM	0.871339	0.030679	pulmonary	TRUE	reported	textfile	7	65822360	0.003111	6.53E-18	516807	neutrophi	TRUE	reported	igd	0.000144	74.477	2	TRUE
rs780142	G	T	G	T	0.012729	-0.0142	0.274175	0.253496	FALSE	FALSE	FALSE	I9_PULMEM	0.411821	0.017307	pulmonary	TRUE	reported	textfile	15	62797964	0.002159	3.84E-09	518204	neutrophi	TRUE	reported	igd	6.71E-05	34.76015	2	TRUE
rs7803075	G	A	G	A	-0.01927	-0.00957	0.73519	0.640978	FALSE	FALSE	FALSE	I9_PULMEM	0.542716	0.01573	pulmonary	TRUE	reported	textfile	7	1.31E+08	0.002201	2.19E-18	516804	neutrophi	TRUE	reported	igd	0.000148	76.63577	2	TRUE
rs7815046	C	A	C	A	-0.01314	0.019918	0.303314	0.32309	FALSE	FALSE	FALSE	I9_PULMEM	0.217394	0.016148	pulmonary	TRUE	reported	textfile	8	87054885	0.002108	4.65E-10	519284	neutrophi	TRUE	reported	igd	7.49E-05	38.87283	2	TRUE
rs7816785	T	C	T	C	-0.02122	0.029155	0.605849	0.562374	FALSE	FALSE	FALSE	I9_PULMEM	0.054326	0.015151	pulmonary	TRUE	reported	textfile	8	56797418	0.001967	4.31E-27	519286	neutrophi	TRUE	reported	igd	0.000224	116.3915	2	TRUE
rs7828590	T	A	T	A	0.021937	-0.00136	0.089011	0.088396	FALSE	TRUE	FALSE	I9_PULMEM	0.959248	0.02669	pulmonary	TRUE	reported	textfile	18	21622003	0.003483	3.11E-10	519288	neutrophi	TRUE	reported	igd	7.64E-05	39.66851	2	TRUE

rs7846314	T	A	T	A	0.063921	-0.00159	0.187341	0.194016	FALSE	TRUE	FALSE	I9_PULMEM	0.933401	0.01898	pulmonary	TRUE	reported	textfile	8	61650831	0.002458	########	519288	neutrophi	TRUE	reported	igd	0.001301	676.2724	2	TRUE
rs7852409	G	C	G	C	0.017853	-0.02939	0.76511	0.729015	FALSE	TRUE	FALSE	I9_PULMEM	0.086596	0.01715	pulmonary	TRUE	reported	textfile	9	2621482	0.002386	7.62E-14	517264	neutrophi	TRUE	reported	igd	0.000108	55.98605	2	TRUE
rs7866863	A	G	A	G	0.013079	-0.00838	0.341388	0.371237	FALSE	FALSE	FALSE	I9_PULMEM	0.589853	0.015552	pulmonary	TRUE	reported	textfile	9	1.15E+08	0.002021	1.00E-10	519277	neutrophi	TRUE	reported	igd	8.06E-05	41.88078	2	TRUE
rs7873858	A	G	A	G	0.021293	-0.00534	0.231943	0.29147	FALSE	FALSE	FALSE	I9_PULMEM	0.746535	0.01651	pulmonary	TRUE	reported	textfile	13	42843630	0.002282	1.13E-20	519283	neutrophi	TRUE	reported	igd	0.000168	87.06443	2	TRUE
rs7881315	T	G	T	G	0.01872	0.036395	0.144465	0.214733	FALSE	FALSE	FALSE	I9_PULMEM	0.047666	0.018378	pulmonary	TRUE	reported	textfile	17	5267002	0.002729	7.13E-12	519283	neutrophi	TRUE	reported	igd	9.06E-05	47.05469	2	TRUE
rs789858	T	C	T	C	0.013974	-0.01946	0.403723	0.376974	FALSE	FALSE	FALSE	I9_PULMEM	0.2088	0.015483	pulmonary	TRUE	reported	textfile	3	1.94E+08	0.001961	1.07E-12	519284	neutrophi	TRUE	reported	igd	9.78E-05	50.77906	2	TRUE
rs7904793	T	C	T	C	0.053423	0.023006	0.014695	0.008312	FALSE	FALSE	FALSE	I9_PULMEM	0.783904	0.083893	pulmonary	TRUE	reported	textfile	2	2.2E+08	0.008352	1.64E-10	471581	neutrophi	TRUE	reported	igd	8.68E-05	40.91417	2	TRUE
rs7917772	A	G	A	G	0.016148	-0.00095	0.631343	0.584067	FALSE	FALSE	FALSE	I9_PULMEM	0.950511	0.015264	pulmonary	TRUE	reported	textfile	10	1.04E+08	0.001992	5.41E-16	518346	neutrophi	TRUE	reported	igd	0.000127	65.71388	2	TRUE
rs7934719	T	C	T	C	0.018084	-0.01839	0.413488	0.396588	FALSE	FALSE	FALSE	I9_PULMEM	0.2297	0.015307	pulmonary	TRUE	reported	textfile	11	1.08E+08	0.001959	2.82E-20	519271	neutrophi	TRUE	reported	igd	0.000164	85.21547	2	TRUE
rs7940176	T	C	T	C	-0.01718	0.004862	0.123632	0.13958	FALSE	FALSE	FALSE	I9_PULMEM	0.821992	0.021609	pulmonary	TRUE	reported	textfile	11	1.23E+08	0.002922	4.23E-09	517885	neutrophi	TRUE	reported	igd	6.67E-05	34.55672	2	TRUE
rs7947419	T	C	T	C	0.02247	0.011759	0.059601	0.107153	FALSE	FALSE	FALSE	I9_PULMEM	0.629318	0.024361	pulmonary	TRUE	reported	textfile	11	1.34E+08	0.004063	3.27E-08	519222	neutrophi	TRUE	reported	igd	5.89E-05	30.58516	2	TRUE
rs796007	A	G	A	G	-0.02067	0.015793	0.252821	0.254449	FALSE	FALSE	FALSE	I9_PULMEM	0.359234	0.017226	pulmonary	TRUE	reported	textfile	9	86577541	0.002211	9.41E-21	519281	neutrophi	TRUE	reported	igd	0.000168	87.39806	2	TRUE
rs796056	C	A	C	A	0.022344	-0.00791	0.62431	0.689541	FALSE	FALSE	FALSE	I9_PULMEM	0.625561	0.016203	pulmonary	TRUE	reported	textfile	2	1.02E+08	0.001977	1.43E-29	518348	neutrophi	TRUE	reported	igd	0.000246	127.7341	2	TRUE
rs7969023	C	T	C	T	-0.01139	0.01082	0.405651	0.349247	FALSE	FALSE	FALSE	I9_PULMEM	0.491981	0.015746	pulmonary	TRUE	reported	textfile	12	26803949	0.001959	6.23E-09	519284	neutrophi	TRUE	reported	igd	6.51E-05	33.79875	2	TRUE
rs7987258	A	G	A	G	-0.01133	0.005117	0.661585	0.70732	FALSE	FALSE	FALSE	I9_PULMEM	0.756134	0.016478	pulmonary	TRUE	reported	textfile	13	49611122	0.002036	2.67E-08	517887	neutrophi	TRUE	reported	igd	5.98E-05	30.96725	2	TRUE
rs8030089	T	C	T	C	-0.01887	0.012447	0.195361	0.213635	FALSE	FALSE	FALSE	I9_PULMEM	0.495735	0.018271	pulmonary	TRUE	reported	textfile	15	75290377	0.002426	7.79E-15	519286	neutrophi	TRUE	reported	igd	0.000116	60.50072	2	TRUE
rs8079215	T	C	T	C	0.012121	0.017057	0.721802	0.756142	FALSE	FALSE	FALSE	I9_PULMEM	0.329727	0.0175	pulmonary	TRUE	reported	textfile	17	44064851	0.002178	2.68E-08	517887	neutrophi	TRUE	reported	igd	5.98E-05	30.9713	2	TRUE
rs8112528	A	G	A	G	-0.01383	-0.02589	0.190498	0.258014	FALSE	FALSE	FALSE	I9_PULMEM	0.131098	0.017148	pulmonary	TRUE	reported	textfile	19	39977173	0.002475	2.35E-08	517885	neutrophi	TRUE	reported	igd	6.03E-05	31.23782	2	TRUE
rs820384	C	G	C	G	0.01193	-0.00341	0.663193	0.738224	FALSE	TRUE	FALSE	I9_PULMEM	0.842986	0.017206	pulmonary	TRUE	reported	textfile	17	73755392	0.002136	2.40E-08	518185	neutrophi	TRUE	reported	igd	6.02E-05	31.19441	2	TRUE
rs8705	A	G	A	G	-0.01833	0.025523	0.31676	0.435593	FALSE	FALSE	FALSE	I9_PULMEM	0.091207	0.01511	pulmonary	TRUE	reported	textfile	11	1.28E+08	0.002092	1.97E-18	516637	neutrophi	TRUE	reported	igd	0.000149	76.79661	2	TRUE
rs915125	T	C	T	C	-0.01764	-0.00632	0.281985	0.306559	FALSE	FALSE	FALSE	I9_PULMEM	0.699393	0.016364	pulmonary	TRUE	reported	textfile	6	82463376	0.002142	1.92E-16	517887	neutrophi	TRUE	reported	igd	0.000131	67.81212	2	TRUE
rs9265980	A	G	A	G	0.057457	-0.00315	0.176601	0.093784	FALSE	FALSE	FALSE	I9_PULMEM	0.902348	0.025697	pulmonary	TRUE	reported	textfile	6	31316045	0.002514	########	518205	neutrophi	TRUE	reported	igd	0.001007	522.3405	2	TRUE
rs9267575	C	T	C	T	-0.02641	0.002226	0.397728	0.486035	FALSE	FALSE	FALSE	I9_PULMEM	0.882214	0.015021	pulmonary	TRUE	reported	textfile	6	31811850	0.001965	3.82E-41	518194	neutrophi	TRUE	reported	igd	0.000348	180.611	2	TRUE
rs9277764	A	G	A	G	0.023875	-0.04174	0.121794	0.142449	FALSE	FALSE	FALSE	I9_PULMEM	0.051673	0.021449	pulmonary	TRUE	reported	textfile	6	33095762	0.002928	3.68E-16	519282	neutrophi	TRUE	reported	igd	0.000128	66.48795	2	TRUE
rs930232	A	G	A	G	0.021247	0.008607	0.460333	0.390506	FALSE	FALSE	FALSE	I9_PULMEM	0.578024	0.015473	pulmonary	TRUE	reported	textfile	19	1036018	0.002045	2.91E-25	470568	neutrophi	TRUE	reported	igd	0.000229	107.9461	2	TRUE
rs9390461	G	A	G	A	0.017735	0.035402	0.538879	0.485128	FALSE	FALSE	FALSE	I9_PULMEM	0.018782	0.015066	pulmonary	TRUE	reported	textfile	6	1.48E+08	0.001926	3.49E-20	519285	neutrophi	TRUE	reported	igd	0.000163	84.79069	2	TRUE
rs9402685	C	T	C	T	-0.03385	0.030434	0.256024	0.339943	FALSE	FALSE	FALSE	I9_PULMEM	0.055266	0.015878	pulmonary	TRUE	reported	textfile	6	1.35E+08	0.002214	1.05E-52	519285	neutrophi	TRUE	reported	igd	0.00045	233.6995	2	TRUE
rs9411293	G	C	G	C	-0.01398	0.021556	0.337095	0.34205	FALSE	TRUE	FALSE	I9_PULMEM	0.178307	0.016015	pulmonary	TRUE	reported	textfile	9	1.4E+08	0.002118	4.19E-11	475801	neutrophi	TRUE	reported	igd	9.16E-05	43.5673	2	TRUE
rs9419387	A	G	A	G	-0.0124	-0.01576	0.405212	0.316857	FALSE	FALSE	FALSE	I9_PULMEM	0.328353	0.016128	pulmonary	TRUE	reported	textfile	10	1.34E+08	0.001962	2.66E-10	517886	neutrophi	TRUE	reported	igd	7.71E-05	39.95616	2	TRUE
rs9508005	G	T	G	T	-0.01994	0.020874	0.09447	0.061801	FALSE	FALSE	FALSE	I9_PULMEM	0.505701	0.031364	pulmonary	TRUE	reported	textfile	13	28789794	0.00356	2.18E-08	516030	neutrophi	TRUE	reported	igd	6.08E-05	31.36932	2	TRUE
rs9543219	T	C	T	C	0.012822	-0.00211	0.574059	0.641329	FALSE	FALSE	FALSE	I9_PULMEM	0.892668	0.01565	pulmonary	TRUE	reported	textfile	13	73638478	0.00195	5.06E-11	518342	neutrophi	TRUE	reported	igd	8.34E-05	43.23552	2	TRUE
rs9656395	G	A	G	A	-0.02284	-0.01172	0.095253	0.074239	FALSE	FALSE	FALSE	I9_PULMEM	0.682438	0.028656	pulmonary	TRUE	reported	textfile	7	1.31E+08	0.003291	4.08E-12	519284	neutrophi	TRUE	reported	igd	9.27E-05	48.14849	2	TRUE
rs9804265	C	A	C	A	-0.04002	0.016608	0.366957	0.384742	FALSE	FALSE	FALSE	I9_PULMEM	0.281998	0.015437	pulmonary	TRUE	reported	textfile	10	25214050	0.001997	2.79E-89	519285	neutrophi	TRUE	reported	igd	0.000773	401.6025	2	TRUE
rs981132	G	T	G	T	0.021149	0.009007	0.678258	0.725321	FALSE	FALSE	FALSE	I9_PULMEM	0.592364	0.016823	pulmonary	TRUE	reported	textfile	7	50281539	0.002054	7.88E-25	517887	neutrophi	TRUE	reported	igd	0.000205	106.0174	2	TRUE
rs9819371	T	C	T	C	-0.04312	-0.01507	0.064869	0.051937	FALSE	FALSE	FALSE	I9_PULMEM	0.657712	0.034002	pulmonary	TRUE	reported	textfile	3	1.41E+08	0.003916	3.64E-28	519285	neutrophi	TRUE	reported	igd	0.000233	121.2637	2	TRUE
rs9842724	C	T	C	T	-0.01429	0.011238	0.800478	0.734745	FALSE	FALSE	FALSE	I9_PULMEM	0.508596	0.017	pulmonary	TRUE	reported	textfile	3	63804761	0.00242	3.67E-09	519286	neutrophi	TRUE	reported	igd	6.71E-05	34.84888	2	TRUE
rs9885207	C	T	C	T	0.016187	-0.02548	0.415999	0.418843	FALSE	FALSE	FALSE	I9_PULMEM	0.096206	0.015319	pulmonary	TRUE	reported	textfile	5	1.73E+08	0.001953	1.22E-16	519285	neutrophi	TRUE	reported	igd	0.000132	68.69523	2	TRUE
rs9905106	C	T	C	T	0.022774	-0.01683	0.733598	0.666605	FALSE	FALSE	FALSE	I9_PULMEM	0.28977	0.015896	pulmonary	TRUE	reported	textfile	17	1373518	0.002227	1.62E-24	518198	neutrophi	TRUE	reported	igd	0.000202	104.5771	2	TRUE
rs9965539	A	G	A	G	0.031107	-0.00378	0.133702	0.148807	FALSE	FALSE	FALSE	I9_PULMEM	0.858049	0.021109	pulmonary	TRUE	reported	textfile	18	41973779	0.002856	1.35E-27	519277	neutrophi	TRUE	reported	igd	0.000228	118.631	2	TRUE
rs9977672	A	G	A	G	-0.02232	-0.02718	0.2588	0.237007	FALSE	FALSE	FALSE	I9_PULMEM	0.12502	0.017718	pulmonary	TRUE	reported	textfile	21	40463283	0.002215	7.34E-24	519287	neutrophi	TRUE	reported	igd	0.000196	101.5678	2	TRUE
rs9988894	G	C	G	C	0.014978	-0.00784	0.323507	0.271697	FALSE	TRUE	FALSE	I9_PULMEM	0.643101	0.016928	pulmonary	TRUE	reported	textfile	11	12876944	0.002075	5.52E-13	516450	neutrophi	TRUE	reported	igd	0.000101	52.10384	2	TRUE

Supplementary Table 7: Description of SNPs for WBC cell counts incorporated after MR-presso deletion

SNP	effect_al	other_alle	effect_al	other_all	beta.expo	beta.outc	eaf.expo	eaf.outco	remove	palindrom	ambiguous	id.outcom	pval.outc	se.outcom	outcome	mr_keep.	pval_orig	data_sour	chr.expos	pos.expos	se.expos	pval.expo	samplesiz	exposure	mr_keep.	pval_orig	data_sour	R2	F	action	mr_keep
rs1000649	G	T	G	T	-0.01567	0.017596	0.353947	0.338623	FALSE	FALSE	FALSE	I9_PULMEM	0.268106	0.015889		TRUE	reported	textfile	4	1.2E+08	0.001959	1.35E-15	558743	white blc	TRUE	reported	igd	0.000114	63.95894	2	TRUE
rs1007938	G	A	G	A	-0.01116	0.010768	0.40493	0.349333	FALSE	FALSE	FALSE	I9_PULMEM	0.494108	0.015748	pulmonary	TRUE	reported	textfile	12	26802549	0.001884	3.26E-09	557353	white blc	TRUE	reported	igd	6.29E-05	35.06337	2	TRUE
rs1008724	T	C	T	C	0.019951	-0.00236	0.457631	0.506548	FALSE	FALSE	FALSE	I9_PULMEM	0.874839	0.015005	pulmonary	TRUE	reported	textfile	8	1.29E+08	0.001855	6.13E-27	560510	white blc	TRUE	reported	igd	0.000206	115.6751	2	TRUE
rs1010287	A	G	A	G	-0.01321	-0.01268	0.684662	0.674078	FALSE	FALSE	FALSE	I9_PULMEM	0.427806	0.01599	pulmonary	TRUE	reported	textfile	8	79598362	0.001997	3.91E-11	560219	white blc	TRUE	reported	igd	7.81E-05	43.74379	2	TRUE
rs1013875	T	C	T	C	-0.04032	0.018285	0.077277	0.115927	FALSE	FALSE	FALSE	I9_PULMEM	0.435687	0.023458	pulmonary	TRUE	reported	textfile	14	69179971	0.003453	1.85E-31	562237	white blc	TRUE	reported	igd	0.000242	136.3405	2	TRUE
rs1014696	C	T	C	T	-0.01537	0.009071	0.338281	0.447414	FALSE	FALSE	FALSE	I9_PULMEM	0.547974	0.015099	pulmonary	TRUE	reported	textfile	14	1.01E+08	0.00197	6.45E-15	557343	white blc	TRUE	reported	igd	0.000109	60.8477	2	TRUE
rs1016476	T	C	T	C	0.027162	0.044573	0.74307	0.853799	FALSE	FALSE	FALSE	I9_PULMEM	0.036254	0.021285	pulmonary	TRUE	reported	textfile	2	2.38E+08	0.002111	7.73E-38	558142	white blc	TRUE	reported	igd	0.000297	165.5562	2	TRUE
rs1020383	C	T	C	T	0.012292	0.003545	0.417873	0.410142	FALSE	FALSE	FALSE	I9_PULMEM	0.816096	0.015243	pulmonary	TRUE	reported	textfile	2	2.14E+08	0.001866	4.66E-11	557354	white blc	TRUE	reported	igd	7.78E-05	43.39306	2	TRUE
rs1020547	A	C	A	C	-0.01295	0.002549	0.201408	0.137668	FALSE	FALSE	FALSE	I9_PULMEM	0.907417	0.021918	pulmonary	TRUE	reported	textfile	2	1.98E+08	0.002325	2.65E-08	558130	white blc	TRUE	reported	igd	5.55E-05	30.99964	2	TRUE
rs1024091	T	C	T	C	0.013797	0.009236	0.557743	0.535749	FALSE	FALSE	FALSE	I9_PULMEM	0.539106	0.015039	pulmonary	TRUE	reported	textfile	17	40771994	0.001848	8.71E-14	562232	white blc	TRUE	reported	igd	9.91E-05	55.7396	2	TRUE
rs1026028	C	G	C	G	0.013403	-0.02594	0.236987	0.196487	FALSE	TRUE	FALSE	I9_PULMEM	0.16982	0.018897	pulmonary	TRUE	reported	textfile	7	38270550	0.002157	5.33E-10	562241	white blc	TRUE	reported	igd	6.87E-05	38.61021	2	TRUE
rs1033415	G	A	G	A	0.012676	0.025673	0.650368	0.710779	FALSE	FALSE	FALSE	I9_PULMEM	0.120826	0.016549	pulmonary	TRUE	reported	textfile	22	39888774	0.002046	6.01E-10	507333	white blc	TRUE	reported	igd	7.57E-05	38.38411	2	TRUE
rs1042725	T	C	T	C	0.010899	-0.00608	0.494933	0.474992	FALSE	FALSE	FALSE	I9_PULMEM	0.686068	0.015048	pulmonary	TRUE	reported	textfile	12	66358347	0.001847	3.71E-09	557347	white blc	TRUE	reported	igd	6.25E-05	34.82074	2	TRUE
rs1047891	A	C	A	C	-0.02111	0.038443	0.314955	0.321403	FALSE	FALSE	FALSE	I9_PULMEM	0.017165	0.016131	pulmonary	TRUE	reported	textfile	2	2.12E+08	0.001979	1.60E-26	558143	white blc	TRUE	reported	igd	0.000204	113.7307	2	TRUE
rs1049478	A	G	A	G	-0.04381	0.047897	0.052086	0.067459	FALSE	FALSE	FALSE	I9_PULMEM	0.109072	0.029891	pulmonary	TRUE	reported	textfile	1	1.99E+08	0.004145	4.43E-26	560511	white blc	TRUE	reported	igd	0.000199	111.7263	2	TRUE
rs1049863	T	C	T	C	-0.03117	0.03142	0.18322	0.134993	FALSE	FALSE	FALSE	I9_PULMEM	0.152593	0.021966	pulmonary	TRUE	reported	textfile	14	93103309	0.002388	6.58E-39	561451	white blc	TRUE	reported	igd	0.000303	170.3631	2	TRUE
rs1080813	A	G	A	G	0.017618	-0.03094	0.118535	0.160104	FALSE	FALSE	FALSE	I9_PULMEM	0.129898	0.020427	pulmonary	TRUE	reported	textfile	7	1.05E+08	0.002845	6.06E-10	562238	white blc	TRUE	reported	igd	6.82E-05	38.34839	2	TRUE
rs1080853	A	G	A	G	0.011839	-0.00079	0.295428	0.378992	FALSE	FALSE	FALSE	I9_PULMEM	0.959205	0.015465	pulmonary	TRUE	reported	textfile	8	87169049	0.002018	4.56E-09	560832	white blc	TRUE	reported	igd	6.14E-05	34.41804	2	TRUE
rs1081419	T	C	T	C	0.011216	0.004028	0.713413	0.611324	FALSE	FALSE	FALSE	I9_PULMEM	0.793831	0.015412	pulmonary	TRUE	reported	textfile	9	34999007	0.002038	3.84E-08	562235	white blc	TRUE	reported	igd	5.39E-05	30.28769	2	TRUE
rs1082872	T	G	T	G	-0.04973	0.016362	0.364782	0.384907	FALSE	FALSE	FALSE	I9_PULMEM	0.289204	0.015437	pulmonary	TRUE	reported	textfile	10	25218243	0.001914	########	562240	white blc	TRUE	reported	igd	0.001199	674.9656	2	TRUE
rs1084468	C	T	C	T	0.025362	0.010698	0.341835	0.375153	FALSE	FALSE	FALSE	I9_PULMEM	0.491148	0.015538	pulmonary	TRUE	reported	textfile	12	9899624	0.001952	1.43E-38	558141	white blc	TRUE	reported	igd	0.000302	168.813	2	TRUE
rs1086436	C	T	C	T	0.021532	0.007716	0.502821	0.516335	FALSE	FALSE	FALSE	I9_PULMEM	0.606576	0.014984	pulmonary	TRUE	reported	textfile	1	8918313	0.001838	1.15E-31	559077	white blc	TRUE	reported	igd	0.000245	137.2386	2	TRUE
rs1087222	T	C	T	C	0.014143	-0.00497	0.18332	0.286957	FALSE	FALSE	FALSE	I9_PULMEM	0.764839	0.016611	pulmonary	TRUE	reported	textfile	6	1.22E+08	0.002394	3.57E-09	561300	white blc	TRUE	reported	igd	6.22E-05	34.90063	2	TRUE
rs1088957	A	G	A	G	-0.04369	0.000827	0.356606	0.450074	FALSE	FALSE	FALSE	I9_PULMEM	0.956371	0.015116	pulmonary	TRUE	reported	textfile	1	66149341	0.001998	########	508416	white blc	TRUE	reported	igd	0.00094	478.1142	2	TRUE
rs1093193	C	T	C	T	0.01504	-0.00936	0.600163	0.556712	FALSE	FALSE	FALSE	I9_PULMEM	0.536645	0.015149	pulmonary	TRUE	reported	textfile	2	2.02E+08	0.001882	1.38E-15	557352	white blc	TRUE	reported	igd	0.000115	63.86382	2	TRUE
rs1093658	A	G	A	G	-0.01431	-0.01258	0.636322	0.708082	FALSE	FALSE	FALSE	I9_PULMEM	0.446353	0.016515	pulmonary	TRUE	reported	textfile	3	1.69E+08	0.001909	6.95E-14	561157	white blc	TRUE	reported	igd	0.0001	56.18303	2	TRUE
rs1094047	T	C	T	C	-0.01337	0.018726	0.659356	0.684286	FALSE	FALSE	FALSE	I9_PULMEM	0.247357	0.016188	pulmonary	TRUE	reported	textfile	5	54880701	0.001946	6.53E-12	557354	white blc	TRUE	reported	igd	8.47E-05	47.2319	2	TRUE
rs1098079	G	A	G	A	-0.02698	0.002422	0.487935	0.470379	FALSE	FALSE	FALSE	I9_PULMEM	0.871711	0.014998	pulmonary	TRUE	reported	textfile	9	1.14E+08	0.00184	1.19E-48	562242	white blc	TRUE	reported	igd	0.000382	215.02	2	TRUE
rs1098633	A	G	A	G	-0.01661	-0.00257	0.649839	0.684027	FALSE	FALSE	FALSE	I9_PULMEM	0.873144	0.016105	pulmonary	TRUE	reported	textfile	9	1.27E+08	0.001953	1.93E-17	561155	white blc	TRUE	reported	igd	0.000129	72.34988	2	TRUE
rs1100230	T	C	T	C	0.012677	-0.00823	0.340114	0.274265	FALSE	FALSE	FALSE	I9_PULMEM	0.624606	0.016817	pulmonary	TRUE	reported	textfile	10	79594931	0.00195	8.28E-11	561296	white blc	TRUE	reported	igd	7.53E-05	42.26318	2	TRUE
rs1102217	G	C	G	C	-0.01806	-0.02867	0.311138	0.314952	FALSE	TRUE	FALSE	I9_PULMEM	0.076431	0.016184	pulmonary	TRUE	reported	textfile	11	12133478	0.002016	3.43E-19	562238	white blc	TRUE	reported	igd	0.000143	80.27811	2	TRUE
rs1106488	A	G	A	G	-0.03227	0.004671	0.073548	0.086328	FALSE	FALSE	FALSE	I9_PULMEM	0.861944	0.026858	pulmonary	TRUE	reported	textfile	12	1.2E+08	0.003547	9.92E-20	559081	white blc	TRUE	reported	igd	0.000148	82.74968	2	TRUE
rs1109278	G	A	G	A	-0.0144	-0.00049	0.483817	0.485829	FALSE	FALSE	FALSE	I9_PULMEM	0.974316	0.015085	pulmonary	TRUE	reported	textfile	17	76248229	0.001932	9.32E-14	506688	white blc	TRUE	reported	igd	0.00011	55.58406	2	TRUE
rs1110488	C	T	C	T	-0.02474	-0.03933	0.702031	0.596551	FALSE	FALSE	FALSE	I9_PULMEM	0.010327	0.015333	pulmonary	TRUE	reported	textfile	12	88843474	0.002017	1.59E-34	561301	white blc	TRUE	reported	igd	0.000268	150.3871	2	TRUE
rs1111414	T	A	T	A	-0.01365	0.007675	0.683244	0.753328	FALSE	TRUE	FALSE	I9_PULMEM	0.659502	0.017419	pulmonary	TRUE	reported	textfile	12	79908743	0.001991	7.30E-12	562238	white blc	TRUE	reported	igd	8.36E-05	47.02319	2	TRUE
rs1115926	C	T	C	T	-0.01437	-0.01432	0.534241	0.53467	FALSE	FALSE	FALSE	I9_PULMEM	0.340052	0.015012	pulmonary	TRUE	reported	textfile	14	77921120	0.001851	8.68E-15	559077	white blc	TRUE	reported	igd	0.000108	60.26972	2	TRUE
rs1116930	G	T	G	T	0.018563	0.016446	0.392444	0.331053	FALSE	FALSE	FALSE	I9_PULMEM	0.301613	0.015921	pulmonary	TRUE	reported	textfile	12	50578705	0.001889	9.45E-23	561149	white blc	TRUE	reported	igd	0.000172	96.56747	2	TRUE
rs1119878	C	T	C	T	0.011894	-0.00766	0.430983	0.465671	FALSE	FALSE	FALSE	I9_PULMEM	0.610666	0.015046	pulmonary	TRUE	reported	textfile	10	1.21E+08	0.001855	1.47E-10	562239	white blc	TRUE	reported	igd	7.31E-05	41.1118	2	TRUE
rs1122139	C	T	C	T	0.016288	-0.01285	0.117605	0.150733	FALSE	FALSE	FALSE	I9_PULMEM	0.542886	0.021127	pulmonary	TRUE	reported	textfile	11	1.28E+08	0.002877	1.54E-08	562235	white blc	TRUE	reported	igd	5.70E-05	32.05193	2	TRUE
rs1122820	T	C	T	C	-0.01779	0.019518	0.271206	0.327997	FALSE	FALSE	FALSE	I9_PULMEM	0.222779	0.016009	pulmonary	TRUE	reported	textfile	16	30826936	0.002073	9.93E-18	562228	white blc	TRUE	reported	igd	0.000131	73.64644	2	TRUE
rs1124210	T	G	T	G	-0.03307	-0.03345	0.477227	0.344453	FALSE	FALSE	FALSE	I9_PULMEM	0.034357	0.015811	pulmonary	TRUE	reported	textfile	5	1.32E+08	0.001842	5.08E-72	561148	white blc	TRUE	reported	igd	0.000574	322.3008	2	TRUE

rs1125007	G	A	G	A	0.021379	-0.02145	0.574673	0.488843	FALSE	FALSE	FALSE	I9_PULMEM	0.154929	0.015084	pulmonary	TRUE	reported	textfile	8	10647823	0.001867	2.44E-30	559428	white blc	TRUE	reported	igd	0.000234	131.1247	2	TRUE
rs1125233	G	T	G	T	0.016749	0.02183	0.836619	0.866491	FALSE	FALSE	FALSE	I9_PULMEM	0.320197	0.02196	pulmonary	TRUE	reported	textfile	10	4129296	0.00249	1.80E-11	559084	white blc	TRUE	reported	igd	8.09E-05	45.24572	2	TRUE
rs1127501	C	T	C	T	0.015327	-0.01317	0.23854	0.195576	FALSE	FALSE	FALSE	I9_PULMEM	0.486343	0.018919	pulmonary	TRUE	reported	textfile	1	26859695	0.002179	2.06E-12	562239	white blc	TRUE	reported	igd	8.80E-05	49.47642	2	TRUE
rs1128808	A	G	A	G	0.025752	0.026788	0.090039	0.083652	FALSE	FALSE	FALSE	I9_PULMEM	0.32537	0.027238	pulmonary	TRUE	reported	textfile	3	1.32E+08	0.003204	9.68E-16	562233	white blc	TRUE	reported	igd	0.000115	64.60042	2	TRUE
rs1134736	G	A	G	A	-0.05083	0.012298	0.025546	0.014192	FALSE	FALSE	FALSE	I9_PULMEM	0.847304	0.063866	pulmonary	TRUE	reported	textfile	4	1.03E+08	0.006222	3.24E-16	562239	white blc	TRUE	reported	igd	0.000119	66.74409	2	TRUE
rs1135198	A	C	A	C	-0.03244	0.012059	0.028736	0.081174	FALSE	FALSE	FALSE	I9_PULMEM	0.662935	0.027667	pulmonary	TRUE	reported	textfile	11	76020088	0.005656	9.92E-09	562232	white blc	TRUE	reported	igd	5.85E-05	32.90398	2	TRUE
rs1140506	T	C	T	C	-0.13824	-0.01307	0.011062	0.016906	FALSE	FALSE	FALSE	I9_PULMEM	0.820194	0.057504	pulmonary	TRUE	reported	textfile	2	2.19E+08	0.00975	1.38E-45	546609	white blc	TRUE	reported	igd	0.000368	201.0251	2	TRUE
rs1144273	G	C	G	C	-0.03629	0.022655	0.03761	0.084111	FALSE	TRUE	FALSE	I9_PULMEM	0.40573	0.027248	pulmonary	TRUE	reported	textfile	2	1.53E+08	0.004834	6.36E-14	558141	white blc	TRUE	reported	igd	0.000101	56.35224	2	TRUE
rs1154332	A	G	A	G	-0.02421	-0.02535	0.053082	0.037214	FALSE	FALSE	FALSE	I9_PULMEM	0.523397	0.039729	pulmonary	TRUE	reported	textfile	3	70940474	0.004232	1.09E-08	561302	white blc	TRUE	reported	igd	5.83E-05	32.71547	2	TRUE
rs1164412	T	C	T	C	-0.01778	-0.00486	0.59737	0.580096	FALSE	FALSE	FALSE	I9_PULMEM	0.750253	0.015279	pulmonary	TRUE	reported	textfile	16	57058974	0.001893	6.36E-21	561302	white blc	TRUE	reported	igd	0.000157	88.19887	2	TRUE
rs1166944	A	G	A	G	0.013882	-0.04319	0.236724	0.25739	FALSE	FALSE	FALSE	I9_PULMEM	0.012198	0.017232	pulmonary	TRUE	reported	textfile	19	1109215	0.002267	9.50E-10	508415	white blc	TRUE	reported	igd	7.37E-05	37.49724	2	TRUE
rs1168830	T	C	T	C	0.021084	-0.02066	0.120295	0.100143	FALSE	FALSE	FALSE	I9_PULMEM	0.410838	0.025123	pulmonary	TRUE	reported	textfile	2	1.28E+08	0.002892	3.19E-13	558142	white blc	TRUE	reported	igd	9.52E-05	53.15056	2	TRUE
rs1172362	G	A	G	A	-0.02908	-0.03222	0.29001	0.200351	FALSE	FALSE	FALSE	I9_PULMEM	0.085645	0.018743	pulmonary	TRUE	reported	textfile	4	72615362	0.002022	7.57E-47	562242	white blc	TRUE	reported	igd	0.000368	206.8212	2	TRUE
rs1173566	T	C	T	C	0.052247	0.03813	0.033197	0.022719	FALSE	FALSE	FALSE	I9_PULMEM	0.451831	0.05068	pulmonary	TRUE	reported	textfile	4	1.45E+08	0.005162	4.74E-24	562239	white blc	TRUE	reported	igd	0.000182	102.4437	2	TRUE
rs1174466	A	G	A	G	-0.01873	-0.01469	0.190499	0.182069	FALSE	FALSE	FALSE	I9_PULMEM	0.449609	0.01943	pulmonary	TRUE	reported	textfile	5	57315635	0.002351	1.72E-15	562241	white blc	TRUE	reported	igd	0.000113	63.48359	2	TRUE
rs1175561	A	G	A	G	0.027237	-0.04855	0.056886	0.046682	FALSE	FALSE	FALSE	I9_PULMEM	0.171645	0.035518	pulmonary	TRUE	reported	textfile	16	67680806	0.004058	1.99E-11	553958	white blc	TRUE	reported	igd	8.13E-05	45.0498	2	TRUE
rs1185439	T	C	T	C	0.013665	0.015377	0.562198	0.594364	FALSE	FALSE	FALSE	I9_PULMEM	0.316212	0.015342	pulmonary	TRUE	reported	textfile	15	80224817	0.00186	2.13E-13	562241	white blc	TRUE	reported	igd	9.60E-05	53.9749	2	TRUE
rs1187445	A	G	A	G	-0.01155	0.022812	0.532635	0.465058	FALSE	FALSE	FALSE	I9_PULMEM	0.132973	0.015183	pulmonary	TRUE	reported	textfile	18	20750400	0.001904	1.36E-09	560213	white blc	TRUE	reported	igd	6.57E-05	36.80474	2	TRUE
rs1192725	T	C	T	C	-0.01288	0.012244	0.282198	0.241685	FALSE	FALSE	FALSE	I9_PULMEM	0.486016	0.017576	pulmonary	TRUE	reported	textfile	3	27334294	0.002042	2.97E-10	560809	white blc	TRUE	reported	igd	7.09E-05	39.76641	2	TRUE
rs1196588	T	G	T	G	-0.01534	-0.01747	0.58175	0.58416	FALSE	FALSE	FALSE	I9_PULMEM	0.254191	0.015323	pulmonary	TRUE	reported	textfile	6	43693094	0.001892	5.41E-16	560214	white blc	TRUE	reported	igd	0.000117	65.73655	2	TRUE
rs1213878	C	T	C	T	-0.01462	0.00194	0.237746	0.244974	FALSE	FALSE	FALSE	I9_PULMEM	0.91153	0.01746	pulmonary	TRUE	reported	textfile	1	9192307	0.00218	2.08E-11	562238	white blc	TRUE	reported	igd	8.00E-05	44.95748	2	TRUE
rs1214247	G	A	G	A	-0.02165	0.00031	0.086122	0.093175	FALSE	FALSE	FALSE	I9_PULMEM	0.990418	0.025835	pulmonary	TRUE	reported	textfile	1	68056314	0.003435	3.05E-10	507475	white blc	TRUE	reported	igd	7.83E-05	39.71369	2	TRUE
rs1215742	A	G	A	G	-0.02125	-0.0192	0.064431	0.049404	FALSE	FALSE	FALSE	I9_PULMEM	0.578032	0.03451	pulmonary	TRUE	reported	textfile	22	32083756	0.003755	1.56E-08	561250	white blc	TRUE	reported	igd	5.70E-05	32.01951	2	TRUE
rs1220359	T	C	T	C	0.022394	0.02304	0.210255	0.030878	FALSE	FALSE	FALSE	I9_PULMEM	0.602718	0.044266	pulmonary	TRUE	reported	textfile	6	396321	0.002349	1.61E-21	544160	white blc	TRUE	reported	igd	0.000167	90.88573	2	TRUE
rs1221426	A	G	A	G	0.018185	-0.0139	0.545937	0.615824	FALSE	FALSE	FALSE	I9_PULMEM	0.370825	0.015534	pulmonary	TRUE	reported	textfile	6	1.36E+08	0.001839	5.06E-23	562241	white blc	TRUE	reported	igd	0.000174	97.78261	2	TRUE
rs1221686	T	C	T	C	0.011449	0.009071	0.290091	0.303924	FALSE	FALSE	FALSE	I9_PULMEM	0.578429	0.016324	pulmonary	TRUE	reported	textfile	8	41828063	0.002032	1.81E-08	562239	white blc	TRUE	reported	igd	5.65E-05	31.74579	2	TRUE
rs1228024	A	C	A	C	0.025191	-0.03513	0.661308	0.656618	FALSE	FALSE	FALSE	I9_PULMEM	0.026314	0.015813	pulmonary	TRUE	reported	textfile	11	47951353	0.001944	2.32E-38	561154	white blc	TRUE	reported	igd	0.000299	167.9178	2	TRUE
rs1237651	C	T	C	T	-0.0342	-0.04091	0.164417	0.189159	FALSE	FALSE	FALSE	I9_PULMEM	0.033626	0.019254	pulmonary	TRUE	reported	textfile	9	22142756	0.002489	6.16E-43	562241	white blc	TRUE	reported	igd	0.000336	188.8216	2	TRUE
rs1242971	T	C	T	C	-0.01113	-0.00709	0.424441	0.417841	FALSE	FALSE	FALSE	I9_PULMEM	0.641058	0.015206	pulmonary	TRUE	reported	textfile	13	99649831	0.001864	2.45E-09	562242	white blc	TRUE	reported	igd	6.34E-05	35.64667	2	TRUE
rs1245035	A	C	A	C	0.014435	-0.0062	0.627427	0.546265	FALSE	FALSE	FALSE	I9_PULMEM	0.680683	0.015064	pulmonary	TRUE	reported	textfile	12	64976049	0.001914	4.79E-14	559427	white blc	TRUE	reported	igd	0.000102	56.8785	2	TRUE
rs1250200	T	G	T	G	0.011097	-0.01713	0.361382	0.371796	FALSE	FALSE	FALSE	I9_PULMEM	0.273287	0.015633	pulmonary	TRUE	reported	textfile	4	55991042	0.001947	1.24E-08	560506	white blc	TRUE	reported	igd	5.80E-05	32.48461	2	TRUE
rs1250215	C	G	C	G	0.011698	-0.02932	0.64108	0.682911	FALSE	TRUE	FALSE	I9_PULMEM	0.069523	0.016153	pulmonary	TRUE	reported	textfile	2	2.16E+08	0.001917	1.08E-09	558142	white blc	TRUE	reported	igd	6.67E-05	37.23724	2	TRUE
rs1254030	T	C	T	C	-0.03868	0.005933	0.048819	0.148209	FALSE	FALSE	FALSE	I9_PULMEM	0.779356	0.021177	pulmonary	TRUE	reported	textfile	7	65662767	0.004464	4.76E-18	508398	white blc	TRUE	reported	igd	0.000148	75.09528	2	TRUE
rs1255061	A	G	A	G	-0.03275	-0.01539	0.821328	0.767985	FALSE	FALSE	FALSE	I9_PULMEM	0.388734	0.017851	pulmonary	TRUE	reported	textfile	8	22966769	0.00242	1.09E-41	560512	white blc	TRUE	reported	igd	0.000327	183.1542	2	TRUE
rs1256644	G	A	G	A	-0.01477	0.026953	0.364982	0.390402	FALSE	FALSE	FALSE	I9_PULMEM	0.079642	0.015377	pulmonary	TRUE	reported	textfile	1	1.01E+08	0.001903	8.79E-15	562242	white blc	TRUE	reported	igd	0.000107	60.25589	2	TRUE
rs1260326	C	T	C	T	-0.03286	0.007219	0.601256	0.650688	FALSE	FALSE	FALSE	I9_PULMEM	0.647251	0.015776	pulmonary	TRUE	reported	textfile	2	27730940	0.001875	9.83E-69	560510	white blc	TRUE	reported	igd	0.000548	307.1923	2	TRUE
rs1263059	T	G	T	G	-0.01233	-0.00251	0.3731	0.38678	FALSE	FALSE	FALSE	I9_PULMEM	0.870438	0.01536	pulmonary	TRUE	reported	textfile	3	1.2E+08	0.001992	6.27E-10	505259	white blc	TRUE	reported	igd	7.58E-05	38.30054	2	TRUE
rs1268369	G	A	G	A	0.021786	-0.02187	0.160286	0.184625	FALSE	FALSE	FALSE	I9_PULMEM	0.258319	0.019346	pulmonary	TRUE	reported	textfile	9	1.13E+08	0.002517	5.16E-18	559084	white blc	TRUE	reported	igd	0.000134	74.91814	2	TRUE
rs1271664	C	G	C	G	0.012379	0.0136	0.637508	0.656897	FALSE	TRUE	FALSE	I9_PULMEM	0.389025	0.015789	pulmonary	TRUE	reported	textfile	8	6901304	0.001918	1.12E-10	562241	white blc	TRUE	reported	igd	7.41E-05	41.6555	2	TRUE
rs1278407	T	C	T	C	0.018141	-0.04248	0.222099	0.20519	FALSE	FALSE	FALSE	I9_PULMEM	0.022531	0.01862	pulmonary	TRUE	reported	textfile	10	1.16E+08	0.002233	4.79E-16	562240	white blc	TRUE	reported	igd	0.000117	65.99993	2	TRUE
rs1279246	G	C	G	C	0.015654	0.000201	0.271243	0.283181	FALSE	TRUE	FALSE	I9_PULMEM	0.990417	0.016698	pulmonary	TRUE	reported	textfile	11	18076439	0.00207	4.13E-14	562235	white blc	TRUE	reported	igd	0.000102	57.18846	2	TRUE
rs1285254	C	T	C	T	-0.01231	0.031567	0.775119	0.818408	FALSE	FALSE	FALSE	I9_PULMEM	0.105434	0.019497	pulmonary	TRUE	reported	textfile	17	77874672	0.002252	4.70E-08	562240	white blc	TRUE	reported	igd	5.31E-05	29.8701	2	TRUE
rs1285886	A	G	A	G	0.026498	-0.02245	0.196802	0.19449	FALSE	FALSE	FALSE	I9_PULMEM	0.235946	0.01894	pulmonary	TRUE	reported	textfile	6	7140831	0.002326	5.10E-30	562241	white blc	TRUE	reported	igd	0.000231	129.7792	2	TRUE
rs1290586	A	G	A	G	0.011091	-0.01247	0.316261	0.275714	FALSE	FALSE	FALSE	I9_PULMEM	0.458064	0.016799	pulmonary	TRUE	reported	textfile	15	78545507	0.001979	2.15E-08	562236	white blc	TRUE	reported	igd	5.59E-05	31.40858	2	TRUE

rs1292995	A	G	A	G	-0.03016	-0.01101	0.077395	0.142545	FALSE	FALSE	FALSE	I9_PULMEN	0.609952	0.021591	pulmonary	TRUE	reported	textfile	16	2144894	0.003616	7.83E-17	508420	white blc	TRUE	reported	igd	0.000137	69.57175	2	TRUE
rs1293652	T	C	T	C	-0.02516	0.015404	0.478786	0.504224	FALSE	FALSE	FALSE	I9_PULMEN	0.304475	0.015001	pulmonary	TRUE	reported	textfile	17	16168784	0.001845	2.74E-42	561298	white blc	TRUE	reported	igd	0.000331	185.9188	2	TRUE
rs1295632	A	C	A	C	0.013776	0.012563	0.402959	0.329351	FALSE	FALSE	FALSE	I9_PULMEN	0.4317	0.015978	pulmonary	TRUE	reported	textfile	18	67537270	0.001875	2.10E-13	562197	white blc	TRUE	reported	igd	9.60E-05	53.98116	2	TRUE
rs1299710	C	T	C	T	0.02676	0.026682	0.070514	0.036626	FALSE	FALSE	FALSE	I9_PULMEN	0.503371	0.039872	pulmonary	TRUE	reported	textfile	2	1.93E+08	0.00359	9.38E-14	558144	white blc	TRUE	reported	igd	9.95E-05	55.5625	2	TRUE
rs1299990	A	G	A	G	0.025242	0.037329	0.061608	0.036603	FALSE	FALSE	FALSE	I9_PULMEN	0.353308	0.040217	pulmonary	TRUE	reported	textfile	2	1.12E+08	0.004067	5.58E-10	504320	white blc	TRUE	reported	igd	7.64E-05	38.52099	2	TRUE
rs1313285	G	A	G	A	0.020133	-0.04496	0.355109	0.379141	FALSE	FALSE	FALSE	I9_PULMEN	0.003671	0.015477	pulmonary	TRUE	reported	textfile	4	38680015	0.00193	1.88E-25	562241	white blc	TRUE	reported	igd	0.000194	108.818	2	TRUE
rs1314920	C	T	C	T	-0.01469	0.00421	0.217886	0.324805	FALSE	FALSE	FALSE	I9_PULMEN	0.792711	0.01602	pulmonary	TRUE	reported	textfile	4	89750668	0.002228	4.50E-11	562243	white blc	TRUE	reported	igd	7.73E-05	43.46037	2	TRUE
rs1318072	A	G	A	G	-0.02131	-0.00672	0.825591	0.836435	FALSE	FALSE	FALSE	I9_PULMEN	0.741181	0.02034	pulmonary	TRUE	reported	textfile	5	1.79E+08	0.002441	2.71E-18	561298	white blc	TRUE	reported	igd	0.000136	76.21313	2	TRUE
rs1322599	T	C	T	C	-0.03066	-0.0155	0.174313	0.1395	FALSE	FALSE	FALSE	I9_PULMEN	0.474785	0.021691	pulmonary	TRUE	reported	textfile	6	16758425	0.002456	9.97E-36	560216	white blc	TRUE	reported	igd	0.000278	155.873	2	TRUE
rs1323763	C	T	C	T	-0.01041	0.021859	0.565316	0.49605	FALSE	FALSE	FALSE	I9_PULMEN	0.146252	0.015045	pulmonary	TRUE	reported	textfile	7	1.49E+08	0.00186	2.24E-08	562241	white blc	TRUE	reported	igd	5.57E-05	31.31174	2	TRUE
rs1324893	G	A	G	A	0.013094	0.00153	0.498342	0.45917	FALSE	FALSE	FALSE	I9_PULMEN	0.919205	0.015083	pulmonary	TRUE	reported	textfile	8	30316162	0.001841	1.17E-12	559076	white blc	TRUE	reported	igd	9.05E-05	50.58661	2	TRUE
rs1329166	G	A	G	A	0.026196	-0.00389	0.186933	0.123252	FALSE	FALSE	FALSE	I9_PULMEN	0.8645	0.022797	pulmonary	TRUE	reported	textfile	9	282738	0.002363	1.57E-28	562235	white blc	TRUE	reported	igd	0.000219	122.8969	2	TRUE
rs1366045	C	T	C	T	-0.02465	0.012216	0.384323	0.343534	FALSE	FALSE	FALSE	I9_PULMEN	0.439232	0.015793	pulmonary	TRUE	reported	textfile	3	42909050	0.001886	5.44E-39	562238	white blc	TRUE	reported	igd	0.000304	170.8795	2	TRUE
rs1371045	C	T	C	T	-0.01433	0.024503	0.744485	0.742818	FALSE	FALSE	FALSE	I9_PULMEN	0.154502	0.01721	pulmonary	TRUE	reported	textfile	2	1.46E+08	0.002114	1.26E-11	558139	white blc	TRUE	reported	igd	8.23E-05	45.93668	2	TRUE
rs1371794	C	A	C	A	-0.06324	0.006381	0.622486	0.671365	FALSE	FALSE	FALSE	I9_PULMEN	0.689349	0.015962	pulmonary	TRUE	reported	textfile	4	74948054	0.001899	########	562241	white blc	TRUE	reported	igd	0.001969	1109.142	2	TRUE
rs1380281	G	C	G	C	0.054052	-0.03935	0.03427	0.014493	FALSE	TRUE	FALSE	I9_PULMEN	0.534019	0.063273	pulmonary	TRUE	reported	textfile	13	28712689	0.005559	2.54E-22	557992	white blc	TRUE	reported	igd	0.000169	94.54284	2	TRUE
rs1383265	C	T	C	T	0.019199	0.006785	0.17909	0.233678	FALSE	FALSE	FALSE	I9_PULMEN	0.7007	0.017653	pulmonary	TRUE	reported	textfile	6	32739888	0.002409	1.68E-15	556269	white blc	TRUE	reported	igd	0.000114	63.51584	2	TRUE
rs1393694	C	T	C	T	0.025273	0.033822	0.047983	0.015472	FALSE	FALSE	FALSE	I9_PULMEN	0.579331	0.061011	pulmonary	TRUE	reported	textfile	2	26054731	0.004383	8.34E-09	562240	white blc	TRUE	reported	igd	5.91E-05	33.24827	2	TRUE
rs1403111	G	C	G	C	-0.02561	0.070106	0.051183	0.05324	FALSE	TRUE	FALSE	I9_PULMEN	0.035787	0.033394	pulmonary	TRUE	reported	textfile	4	26295540	0.004196	1.08E-09	562230	white blc	TRUE	reported	igd	6.62E-05	37.2372	2	TRUE
rs1443170	T	A	T	A	0.049756	0.010383	0.034356	0.057398	FALSE	TRUE	FALSE	I9_PULMEN	0.749604	0.032531	pulmonary	TRUE	reported	textfile	4	1.06E+08	0.005126	3.00E-22	562241	white blc	TRUE	reported	igd	0.000168	94.21763	2	TRUE
rs1452099	A	C	A	C	-0.07536	-0.01823	0.01404	0.008938	FALSE	FALSE	FALSE	I9_PULMEN	0.824317	0.082139	pulmonary	TRUE	reported	textfile	8	1.31E+08	0.008651	3.19E-18	562239	white blc	TRUE	reported	igd	0.000135	75.87549	2	TRUE
rs1462077	T	A	T	A	-0.05427	0.019958	0.017496	0.007507	FALSE	TRUE	FALSE	I9_PULMEN	0.824614	0.090056	pulmonary	TRUE	reported	textfile	17	16472850	0.008237	4.58E-11	507056	white blc	TRUE	reported	igd	8.56E-05	43.4154	2	TRUE
rs1468102	G	C	G	C	0.014895	-0.00238	0.306335	0.235081	FALSE	TRUE	FALSE	I9_PULMEN	0.892784	0.017695	pulmonary	TRUE	reported	textfile	11	3004526	0.002	9.95E-14	562233	white blc	TRUE	reported	igd	9.86E-05	55.46506	2	TRUE
rs1474419	C	T	C	T	0.018298	0.00487	0.579762	0.619091	FALSE	FALSE	FALSE	I9_PULMEN	0.754395	0.015566	pulmonary	TRUE	reported	textfile	7	6692605	0.001886	3.11E-22	560209	white blc	TRUE	reported	igd	0.000168	94.12876	2	TRUE
rs1476081	T	G	T	G	0.011441	0.008055	0.294671	0.400835	FALSE	FALSE	FALSE	I9_PULMEN	0.602529	0.015467	pulmonary	TRUE	reported	textfile	7	17279294	0.00208	3.85E-08	560512	white blc	TRUE	reported	igd	5.40E-05	30.25518	2	TRUE
rs1487131	T	G	T	G	0.096379	0.071538	0.008951	0.004935	FALSE	FALSE	FALSE	I9_PULMEN	0.501753	0.106497	pulmonary	TRUE	reported	textfile	11	1.18E+08	0.010089	1.35E-21	558150	white blc	TRUE	reported	igd	0.000163	91.25718	2	TRUE
rs1506609	G	A	G	A	0.021031	-0.00808	0.799464	0.715694	FALSE	FALSE	FALSE	I9_PULMEN	0.629057	0.016726	pulmonary	TRUE	reported	textfile	4	83632973	0.002326	1.61E-19	558139	white blc	TRUE	reported	igd	0.000146	81.75209	2	TRUE
rs1508617	T	C	T	C	-0.06519	0.007787	0.020249	0.007946	FALSE	FALSE	FALSE	I9_PULMEN	0.928428	0.086694	pulmonary	TRUE	reported	textfile	13	1.09E+08	0.007096	4.31E-20	562214	white blc	TRUE	reported	igd	0.00015	84.40079	2	TRUE
rs1611236	A	G	A	G	-0.03586	-0.0091	0.322501	0.224527	FALSE	FALSE	FALSE	I9_PULMEN	0.612858	0.017986	pulmonary	TRUE	reported	textfile	6	29748690	0.001971	7.26E-74	559747	white blc	TRUE	reported	igd	0.000591	330.9212	2	TRUE
rs1621453	G	A	G	A	0.010499	-0.02724	0.56322	0.643253	FALSE	FALSE	FALSE	I9_PULMEN	0.081752	0.015647	pulmonary	TRUE	reported	textfile	3	1.94E+08	0.00186	1.71E-08	561158	white blc	TRUE	reported	igd	5.68E-05	31.86166	2	TRUE
rs1685007	T	C	T	C	0.042606	0.001218	0.374415	0.385454	FALSE	FALSE	FALSE	I9_PULMEN	0.937135	0.01544	pulmonary	TRUE	reported	textfile	4	74703999	0.001922	########	557354	white blc	TRUE	reported	igd	0.000881	491.3978	2	TRUE
rs1696498	C	T	C	T	0.021856	-0.00797	0.172918	0.173455	FALSE	FALSE	FALSE	I9_PULMEN	0.687893	0.019838	pulmonary	TRUE	reported	textfile	17	27610862	0.002434	2.89E-19	559082	white blc	TRUE	reported	igd	0.000144	80.63037	2	TRUE
rs1701172	G	C	G	C	-0.02363	-0.03654	0.232584	0.149798	FALSE	TRUE	FALSE	I9_PULMEN	0.0826	0.02105	pulmonary	TRUE	reported	textfile	10	50264204	0.002187	3.58E-27	559081	white blc	TRUE	reported	igd	0.000209	116.7127	2	TRUE
rs1702621	G	T	G	T	-0.01472	-0.00288	0.513548	0.582845	FALSE	FALSE	FALSE	I9_PULMEN	0.850028	0.015232	pulmonary	TRUE	reported	textfile	2	85551696	0.001834	1.06E-15	561134	white blc	TRUE	reported	igd	0.000115	64.41046	2	TRUE
rs1704143	C	A	C	A	0.037876	0.010695	0.056554	0.030641	FALSE	FALSE	FALSE	I9_PULMEN	0.805116	0.043346	pulmonary	TRUE	reported	textfile	12	1.02E+08	0.003999	2.96E-21	561300	white blc	TRUE	reported	igd	0.00016	89.70649	2	TRUE
rs1713859	A	G	A	G	-0.01328	-0.01162	0.194778	0.193335	FALSE	FALSE	FALSE	I9_PULMEN	0.543368	0.019116	pulmonary	TRUE	reported	textfile	7	18148091	0.002333	1.30E-08	562241	white blc	TRUE	reported	igd	5.76E-05	32.38198	2	TRUE
rs1720963	A	G	A	G	0.014259	0.006627	0.281259	0.298202	FALSE	FALSE	FALSE	I9_PULMEN	0.687437	0.016472	pulmonary	TRUE	reported	textfile	8	1.06E+08	0.002045	3.25E-12	560513	white blc	TRUE	reported	igd	8.67E-05	48.6172	2	TRUE
rs1723282	T	C	T	C	-0.02183	0.007409	0.10177	0.082329	FALSE	FALSE	FALSE	I9_PULMEN	0.785172	0.027182	pulmonary	TRUE	reported	textfile	16	89842784	0.003087	1.60E-12	562163	white blc	TRUE	reported	igd	8.90E-05	50.0118	2	TRUE
rs1727088	C	G	C	G	0.030784	0.029324	0.239086	0.247692	FALSE	TRUE	FALSE	I9_PULMEN	0.091863	0.017396	pulmonary	TRUE	reported	textfile	2	1.82E+08	0.002158	4.07E-46	558141	white blc	TRUE	reported	igd	0.000364	203.4912	2	TRUE
rs1738074	C	T	C	T	-0.0204	-0.0009	0.567796	0.582489	FALSE	FALSE	FALSE	I9_PULMEN	0.952764	0.015211	pulmonary	TRUE	reported	textfile	6	1.59E+08	0.001853	3.74E-28	560506	white blc	TRUE	reported	igd	0.000216	121.2371	2	TRUE
rs174548	G	C	G	C	-0.02493	-0.04806	0.313935	0.389072	FALSE	TRUE	FALSE	I9_PULMEN	0.001792	0.015391	pulmonary	TRUE	reported	textfile	11	61571348	0.001991	6.38E-36	558136	white blc	TRUE	reported	igd	0.000281	156.7207	2	TRUE
rs175714	C	T	C	T	0.010451	0.0025	0.577258	0.514134	FALSE	FALSE	FALSE	I9_PULMEN	0.867828	0.015024	pulmonary	TRUE	reported	textfile	14	75981856	0.001877	2.64E-08	560513	white blc	TRUE	reported	igd	5.53E-05	31.00171	2	TRUE
rs1775869	T	C	T	C	-0.05595	0.060829	0.028609	0.044003	FALSE	FALSE	FALSE	I9_PULMEN	0.097521	0.036711	pulmonary	TRUE	reported	textfile	18	60920854	0.005728	1.67E-22	559933	white blc	TRUE	reported	igd	0.00017	95.39608	2	TRUE
rs1779809	T	C	T	C	0.014395	0.02323	0.333689	0.287233	FALSE	FALSE	FALSE	I9_PULMEN	0.160997	0.016572	pulmonary	TRUE	reported	textfile	1	1.82E+08	0.001944	1.36E-13	562225	white blc	TRUE	reported	igd	9.75E-05	54.83139	2	TRUE

rs180941	A	G	A	G	-0.01687	-0.00517	0.625014	0.753643	FALSE	FALSE	FALSE	I9_PULMEN	0.766526	0.017396	pulmonary	TRUE	reported	textfile	10	1.16E+08	0.001919	1.63E-18	561157	white blc	TRUE	reported	igd	0.000138	77.24541	2	TRUE
rs1820909	A	G	A	G	-0.07578	-0.02078	0.009784	0.00298	FALSE	FALSE	FALSE	I9_PULMEN	0.881235	0.139058	pulmonary	TRUE	reported	textfile	7	92239892	0.009684	5.33E-15	560391	white blc	TRUE	reported	igd	0.000109	61.22831	2	TRUE
rs1822534	G	A	G	A	-0.02582	-0.01267	0.394237	0.352954	FALSE	FALSE	FALSE	I9_PULMEN	0.421003	0.015741	pulmonary	TRUE	reported	textfile	3	12266804	0.001882	8.29E-43	562234	white blc	TRUE	reported	igd	0.000335	188.2517	2	TRUE
rs1885474	G	T	G	T	-0.02158	-0.00185	0.102894	0.168869	FALSE	FALSE	FALSE	I9_PULMEN	0.926476	0.020082	pulmonary	TRUE	reported	textfile	10	69566751	0.003031	1.11E-12	562227	white blc	TRUE	reported	igd	9.02E-05	50.70493	2	TRUE
rs1886654	C	T	C	T	-0.05749	0.029542	0.892184	0.914285	FALSE	FALSE	FALSE	I9_PULMEN	0.27078	0.026826	pulmonary	TRUE	reported	textfile	1	2.36E+08	0.002959	5.08E-84	562242	white blc	TRUE	reported	igd	0.000671	377.4268	2	TRUE
rs1887428	C	G	C	G	-0.01496	0.009308	0.625091	0.616205	FALSE	TRUE	FALSE	I9_PULMEN	0.547233	0.015464	pulmonary	TRUE	reported	textfile	9	4984530	0.00193	9.58E-15	560218	white blc	TRUE	reported	igd	0.000107	60.05024	2	TRUE
rs1891033	C	T	C	T	0.015029	0.003755	0.16941	0.226708	FALSE	FALSE	FALSE	I9_PULMEN	0.835428	0.018077	pulmonary	TRUE	reported	textfile	20	36900466	0.002482	1.44E-09	559079	white blc	TRUE	reported	igd	6.56E-05	36.66528	2	TRUE
rs1895994	A	G	A	G	-0.01296	-0.00518	0.32446	0.232185	FALSE	FALSE	FALSE	I9_PULMEN	0.771092	0.017809	pulmonary	TRUE	reported	textfile	12	49056663	0.001964	4.35E-11	560151	white blc	TRUE	reported	igd	7.77E-05	43.51012	2	TRUE
rs1933295	G	A	G	A	-0.01555	0.028152	0.776835	0.74384	FALSE	FALSE	FALSE	I9_PULMEN	0.102229	0.017227	pulmonary	TRUE	reported	textfile	1	62107021	0.002311	1.81E-11	508416	white blc	TRUE	reported	igd	8.90E-05	45.25179	2	TRUE
rs1947897	C	G	C	G	-0.01436	-0.00383	0.336356	0.371613	FALSE	TRUE	FALSE	I9_PULMEN	0.804238	0.015471	pulmonary	TRUE	reported	textfile	8	78946874	0.001947	1.71E-13	562239	white blc	TRUE	reported	igd	9.68E-05	54.40464	2	TRUE
rs2012011	A	G	A	G	-0.0255	-0.01725	0.136123	0.171473	FALSE	FALSE	FALSE	I9_PULMEN	0.387716	0.019973	pulmonary	TRUE	reported	textfile	6	46893355	0.002683	2.15E-21	561157	white blc	TRUE	reported	igd	0.000161	90.34531	2	TRUE
rs2038700	C	T	C	T	0.034424	0.012197	0.394624	0.388643	FALSE	FALSE	FALSE	I9_PULMEN	0.428232	0.015396	pulmonary	TRUE	reported	textfile	14	25461989	0.00189	4.61E-74	562237	white blc	TRUE	reported	igd	0.00059	331.7397	2	TRUE
rs2062250	A	G	A	G	0.050625	-0.00811	0.938363	0.939946	FALSE	FALSE	FALSE	I9_PULMEN	0.796938	0.031531	pulmonary	TRUE	reported	textfile	15	64672002	0.003905	2.12E-38	560514	white blc	TRUE	reported	igd	0.0003	168.0685	2	TRUE
rs2068330	G	C	G	C	0.018714	-0.02175	0.360551	0.385941	FALSE	TRUE	FALSE	I9_PULMEN	0.159475	0.015463	pulmonary	TRUE	reported	textfile	2	1.63E+08	0.001927	2.87E-22	558138	white blc	TRUE	reported	igd	0.000169	94.31228	2	TRUE
rs2082382	A	G	A	G	-0.01786	-0.0223	0.55412	0.646122	FALSE	FALSE	FALSE	I9_PULMEN	0.154167	0.015651	pulmonary	TRUE	reported	textfile	5	1.48E+08	0.00186	8.67E-22	562242	white blc	TRUE	reported	igd	0.000164	92.17	2	TRUE
rs2084312	T	C	T	C	0.028894	-0.01467	0.800412	0.751197	FALSE	FALSE	FALSE	I9_PULMEN	0.396846	0.017315	pulmonary	TRUE	reported	textfile	17	72695211	0.002311	7.67E-36	561156	white blc	TRUE	reported	igd	0.000278	156.3198	2	TRUE
rs2091084	C	T	C	T	0.01735	-0.03045	0.653201	0.723906	FALSE	FALSE	FALSE	I9_PULMEN	0.069562	0.01678	pulmonary	TRUE	reported	textfile	10	30492267	0.001951	6.25E-19	562241	white blc	TRUE	reported	igd	0.000141	79.08295	2	TRUE
rs2104415	A	G	A	G	-0.01237	0.025468	0.676227	0.59858	FALSE	FALSE	FALSE	I9_PULMEN	0.095951	0.015298	pulmonary	TRUE	reported	textfile	1	67369398	0.002061	1.99E-09	508418	white blc	TRUE	reported	igd	7.09E-05	36.04063	2	TRUE
rs2148537	A	G	A	G	0.017567	0.015207	0.166266	0.181269	FALSE	FALSE	FALSE	I9_PULMEN	0.434584	0.019462	pulmonary	TRUE	reported	textfile	9	95380726	0.002475	1.31E-12	559080	white blc	TRUE	reported	igd	9.01E-05	50.37827	2	TRUE
rs2180369	C	T	C	T	0.02298	0.005377	0.110762	0.092305	FALSE	FALSE	FALSE	I9_PULMEN	0.835408	0.02588	pulmonary	TRUE	reported	textfile	14	93516465	0.003071	7.65E-14	506687	white blc	TRUE	reported	igd	0.00011	55.99364	2	TRUE
rs218264	T	A	T	A	0.025769	-0.03441	0.250868	0.249337	FALSE	TRUE	FALSE	I9_PULMEN	0.048502	0.017443	pulmonary	TRUE	reported	textfile	4	55408875	0.002141	2.52E-33	562241	white blc	TRUE	reported	igd	0.000258	144.8639	2	TRUE
rs2194067	T	C	T	C	0.012618	-0.00412	0.415502	0.422664	FALSE	FALSE	FALSE	I9_PULMEN	0.786316	0.015183	pulmonary	TRUE	reported	textfile	19	35464727	0.001886	2.31E-11	561155	white blc	TRUE	reported	igd	7.98E-05	44.76062	2	TRUE
rs2259855	C	T	C	T	-0.02361	0.013468	0.52533	0.50704	FALSE	FALSE	FALSE	I9_PULMEN	0.368234	0.014968	pulmonary	TRUE	reported	textfile	17	28097860	0.001849	2.66E-37	562240	white blc	TRUE	reported	igd	0.00029	163.076	2	TRUE
rs2260850	G	T	G	T	0.023885	-0.01134	0.280997	0.295406	FALSE	FALSE	FALSE	I9_PULMEN	0.489887	0.016427	pulmonary	TRUE	reported	textfile	13	1.14E+08	0.002116	1.68E-29	524745	white blc	TRUE	reported	igd	0.000243	127.4141	2	TRUE
rs2282677	C	T	C	T	0.01138	0.034427	0.405364	0.398279	FALSE	FALSE	FALSE	I9_PULMEN	0.024909	0.01535	pulmonary	TRUE	reported	textfile	17	61986539	0.001929	3.79E-09	560831	white blc	TRUE	reported	igd	6.21E-05	34.80315	2	TRUE
rs2282986	C	T	C	T	-0.12435	-0.04624	0.018529	0.026177	FALSE	FALSE	FALSE	I9_PULMEN	0.324943	0.046977	pulmonary	TRUE	reported	textfile	7	92299545	0.006855	1.80E-73	560514	white blc	TRUE	reported	igd	0.000587	329.0494	2	TRUE
rs2284837	G	A	G	A	0.01378	-0.00495	0.220169	0.191517	FALSE	FALSE	FALSE	I9_PULMEN	0.79564	0.019098	pulmonary	TRUE	reported	textfile	3	1.86E+08	0.002231	6.76E-10	562241	white blc	TRUE	reported	igd	6.78E-05	38.15028	2	TRUE
rs2286599	A	G	A	G	0.03864	-0.05162	0.141477	0.103461	FALSE	FALSE	FALSE	I9_PULMEN	0.040716	0.025225	pulmonary	TRUE	reported	textfile	12	6499533	0.00279	1.43E-43	543851	white blc	TRUE	reported	igd	0.000353	191.8069	2	TRUE
rs2290669	C	A	C	A	-0.04762	-0.00539	0.786444	0.722966	FALSE	FALSE	FALSE	I9_PULMEN	0.747426	0.016748	pulmonary	TRUE	reported	textfile	19	16495774	0.002308	1.52E-94	560514	white blc	TRUE	reported	igd	0.000759	425.6304	2	TRUE
rs2298695	C	A	C	A	-0.01573	-0.00901	0.396473	0.366792	FALSE	FALSE	FALSE	I9_PULMEN	0.562596	0.015556	pulmonary	TRUE	reported	textfile	21	47655027	0.001881	6.51E-17	560513	white blc	TRUE	reported	igd	0.000125	69.94127	2	TRUE
rs2305980	A	T	A	T	0.013867	0.009951	0.255073	0.170774	FALSE	TRUE	FALSE	I9_PULMEN	0.618831	0.020001	pulmonary	TRUE	reported	textfile	4	1.51E+08	0.002123	6.67E-11	561297	white blc	TRUE	reported	igd	7.60E-05	42.66419	2	TRUE
rs2312675	C	T	C	T	-0.01756	-0.00308	0.211587	0.178431	FALSE	FALSE	FALSE	I9_PULMEN	0.874969	0.019565	pulmonary	TRUE	reported	textfile	1	1.67E+08	0.002242	5.11E-15	562242	white blc	TRUE	reported	igd	0.000109	61.32363	2	TRUE
rs2314339	T	C	T	C	0.027194	0.01637	0.124894	0.17646	FALSE	FALSE	FALSE	I9_PULMEN	0.406056	0.019703	pulmonary	TRUE	reported	textfile	17	38253212	0.002851	1.52E-21	557350	white blc	TRUE	reported	igd	0.000163	90.98089	2	TRUE
rs2327028	T	C	T	C	-0.01348	0.013066	0.475724	0.499614	FALSE	FALSE	FALSE	I9_PULMEN	0.38369	0.015	pulmonary	TRUE	reported	textfile	20	8140925	0.001851	3.36E-13	562241	white blc	TRUE	reported	igd	9.44E-05	53.06683	2	TRUE
rs2328667	G	A	G	A	0.012282	-0.04553	0.561405	0.674511	FALSE	FALSE	FALSE	I9_PULMEN	0.004301	0.015948	pulmonary	TRUE	reported	textfile	6	22104421	0.001866	4.85E-11	561295	white blc	TRUE	reported	igd	7.72E-05	43.32248	2	TRUE
rs2358581	G	T	G	T	-0.02822	-0.01358	0.73286	0.697596	FALSE	FALSE	FALSE	I9_PULMEN	0.407515	0.016396	pulmonary	TRUE	reported	textfile	19	10391611	0.002196	9.36E-38	506961	white blc	TRUE	reported	igd	0.000326	165.1851	2	TRUE
rs2371108	T	G	T	G	0.019217	0.019244	0.388965	0.378826	FALSE	FALSE	FALSE	I9_PULMEN	0.215111	0.015524	pulmonary	TRUE	reported	textfile	3	27757018	0.001891	3.11E-24	561295	white blc	TRUE	reported	igd	0.000184	103.2729	2	TRUE
rs2394990	A	C	A	C	-0.02032	0.022775	0.632626	0.546319	FALSE	FALSE	FALSE	I9_PULMEN	0.133224	0.015168	pulmonary	TRUE	reported	textfile	6	31340559	0.001892	7.10E-27	560204	white blc	TRUE	reported	igd	0.000206	115.369	2	TRUE
rs2412771	C	T	C	T	-0.0182	0.03283	0.416739	0.440451	FALSE	FALSE	FALSE	I9_PULMEN	0.030801	0.015201	pulmonary	TRUE	reported	textfile	4	57761417	0.001883	4.42E-22	562233	white blc	TRUE	reported	igd	0.000166	93.43042	2	TRUE
rs2427599	T	C	T	C	0.055692	-0.06668	0.010727	0.006681	FALSE	FALSE	FALSE	I9_PULMEN	0.463494	0.090954	pulmonary	TRUE	reported	textfile	20	62687921	0.009529	5.21E-09	508417	white blc	TRUE	reported	igd	6.72E-05	34.15775	2	TRUE
rs2428608	T	C	T	C	-0.01131	-0.01518	0.56284	0.5991	FALSE	FALSE	FALSE	I9_PULMEN	0.321295	0.015301	pulmonary	TRUE	reported	textfile	17	46040795	0.001852	1.05E-09	562230	white blc	TRUE	reported	igd	6.63E-05	37.28764	2	TRUE
rs2432142	A	G	A	G	0.012676	-0.04724	0.433689	0.402627	FALSE	FALSE	FALSE	I9_PULMEN	0.002066	0.015334	pulmonary	TRUE	reported	textfile	5	96275201	0.001864	1.09E-11	562219	white blc	TRUE	reported	igd	8.22E-05	46.24567	2	TRUE
rs2439963	G	A	G	A	-0.01238	0.00346	0.736551	0.796587	FALSE	FALSE	FALSE	I9_PULMEN	0.852612	0.018622	pulmonary	TRUE	reported	textfile	13	78638416	0.002091	3.29E-09	562241	white blc	TRUE	reported	igd	6.23E-05	35.05352	2	TRUE
rs2469147	G	A	G	A	-0.0153	-0.01915	0.157884	0.160965	FALSE	FALSE	FALSE	I9_PULMEN	0.349336	0.020458	pulmonary	TRUE	reported	textfile	15	66910224	0.002536	1.67E-09	562234	white blc	TRUE	reported	igd	6.47E-05	36.37466	2	TRUE

rs247826	T	C	T	C	0.033815	-0.00998	0.220606	0.289212	FALSE	FALSE	FALSE	I9_PULMEM	0.552291	0.016786	pulmonary	TRUE	reported textfile	16	84582965	0.002233	9.59E-52	560506	white blc	TRUE	reported igd	0.000409	229.3189	2	TRUE
rs2506699	G	A	G	A	0.01427	-0.02051	0.483895	0.587011	FALSE	FALSE	FALSE	I9_PULMEM	0.179875	0.015291	pulmonary	TRUE	reported textfile	9	1.37E+08	0.001892	4.87E-14	562240	white blc	TRUE	reported igd	0.000101	56.88583	2	TRUE
rs2561758	G	A	G	A	-0.03629	0.024315	0.723119	0.726442	FALSE	FALSE	FALSE	I9_PULMEM	0.148312	0.016821	pulmonary	TRUE	reported textfile	5	1.73E+08	0.00208	4.20E-68	561302	white blc	TRUE	reported igd	0.000542	304.4008	2	TRUE
rs257063	T	C	T	C	-0.01382	-0.00512	0.740602	0.724767	FALSE	FALSE	FALSE	I9_PULMEM	0.760391	0.016791	pulmonary	TRUE	reported textfile	5	1.15E+08	0.0021	4.91E-11	559081	white blc	TRUE	reported igd	7.74E-05	43.29625	2	TRUE
rs2615061	A	G	A	G	-0.02764	0.000821	0.117457	0.174034	FALSE	FALSE	FALSE	I9_PULMEM	0.967075	0.019893	pulmonary	TRUE	reported textfile	1	2.26E+08	0.002854	3.82E-22	561302	white blc	TRUE	reported igd	0.000167	93.77181	2	TRUE
rs2665405	A	G	A	G	0.035373	-0.00399	0.547743	0.537767	FALSE	FALSE	FALSE	I9_PULMEM	0.790582	0.015037	pulmonary	TRUE	reported textfile	17	57875292	0.001844	5.63E-82	562241	white blc	TRUE	reported igd	0.000654	367.9768	2	TRUE
rs2710804	C	T	C	T	0.020606	0.02718	0.375925	0.353858	FALSE	FALSE	FALSE	I9_PULMEM	0.083123	0.015685	pulmonary	TRUE	reported textfile	7	36084529	0.001897	1.92E-27	560511	white blc	TRUE	reported igd	0.00021	117.9916	2	TRUE
rs2713573	T	C	T	C	0.024566	0.040007	0.689211	0.716233	FALSE	FALSE	FALSE	I9_PULMEM	0.015977	0.016604	pulmonary	TRUE	reported textfile	3	1.28E+08	0.001984	3.46E-35	562241	white blc	TRUE	reported igd	0.000273	153.3148	2	TRUE
rs2729707	G	A	G	A	-0.03093	-0.00605	0.830063	0.791994	FALSE	FALSE	FALSE	I9_PULMEM	0.74417	0.018544	pulmonary	TRUE	reported textfile	2	1.61E+08	0.002446	1.28E-36	558132	white blc	TRUE	reported igd	0.000286	159.9195	2	TRUE
rs2790122	C	T	C	T	0.013402	-0.0105	0.712432	0.780213	FALSE	FALSE	FALSE	I9_PULMEM	0.56115	0.018075	pulmonary	TRUE	reported textfile	1	2E+08	0.002029	4.08E-11	562242	white blc	TRUE	reported igd	7.76E-05	43.62883	2	TRUE
rs284317	G	A	G	A	0.017425	-0.00205	0.497553	0.606881	FALSE	FALSE	FALSE	I9_PULMEM	0.893881	0.015401	pulmonary	TRUE	reported textfile	1	10731625	0.001945	3.52E-19	506067	white blc	TRUE	reported igd	0.000159	80.26101	2	TRUE
rs2853075	A	G	A	G	0.055643	0.025965	0.043032	0.02539	FALSE	FALSE	FALSE	I9_PULMEM	0.584933	0.047537	pulmonary	TRUE	reported textfile	4	36312542	0.004613	1.83E-33	561301	white blc	TRUE	reported igd	0.000259	145.4965	2	TRUE
rs2853951	T	C	T	C	0.077234	-0.04311	0.599507	0.62119	FALSE	FALSE	FALSE	I9_PULMEM	0.005004	0.015361	pulmonary	TRUE	reported textfile	6	31236115	0.001878	########	561159	white blc	TRUE	reported igd	0.003005	1691.314	2	TRUE
rs2857176	C	T	C	T	-0.02207	0.020521	0.206778	0.233297	FALSE	FALSE	FALSE	I9_PULMEM	0.247921	0.017761	pulmonary	TRUE	reported textfile	8	55449281	0.00237	1.32E-20	508414	white blc	TRUE	reported igd	0.000171	86.7174	2	TRUE
rs2857481	G	A	G	A	-0.01604	0.006655	0.15791	0.181293	FALSE	FALSE	FALSE	I9_PULMEM	0.731993	0.019432	pulmonary	TRUE	reported textfile	21	16384940	0.002537	2.66E-10	562202	white blc	TRUE	reported igd	7.11E-05	39.97794	2	TRUE
rs2857622	A	G	A	G	0.031285	0.009965	0.122129	0.138989	FALSE	FALSE	FALSE	I9_PULMEM	0.645201	0.021642	pulmonary	TRUE	reported textfile	15	1.02E+08	0.002853	6.14E-28	547354	white blc	TRUE	reported igd	0.00022	120.245	2	TRUE
rs2857633	C	T	C	T	-0.01328	0.000252	0.727142	0.645928	FALSE	FALSE	FALSE	I9_PULMEM	0.987271	0.015806	pulmonary	TRUE	reported textfile	22	30114333	0.002098	2.52E-10	560513	white blc	TRUE	reported igd	7.15E-05	40.08481	2	TRUE
rs2858874	G	A	G	A	-0.01047	0.001329	0.377846	0.381378	FALSE	FALSE	FALSE	I9_PULMEM	0.931626	0.015492	pulmonary	TRUE	reported textfile	4	1.11E+08	0.001899	3.61E-08	560831	white blc	TRUE	reported igd	5.42E-05	30.40359	2	TRUE
rs2875974	A	G	A	G	0.021365	-0.00672	0.373406	0.405354	FALSE	FALSE	FALSE	I9_PULMEM	0.660424	0.015296	pulmonary	TRUE	reported textfile	8	61419728	0.001904	3.54E-29	559083	white blc	TRUE	reported igd	0.000225	125.9129	2	TRUE
rs2904270	A	G	A	G	-0.01532	-0.0099	0.517092	0.531413	FALSE	FALSE	FALSE	I9_PULMEM	0.510404	0.015041	pulmonary	TRUE	reported textfile	20	49185272	0.001844	1.03E-16	562241	white blc	TRUE	reported igd	0.000123	69.00503	2	TRUE
rs2935104	G	A	G	A	0.01955	-0.03877	0.906903	0.935046	FALSE	FALSE	FALSE	I9_PULMEM	0.204098	0.030531	pulmonary	TRUE	reported textfile	6	1.7E+08	0.003311	3.62E-09	508420	white blc	TRUE	reported igd	6.86E-05	34.8637	2	TRUE
rs2943650	T	C	T	C	0.01116	0.012652	0.644643	0.640545	FALSE	FALSE	FALSE	I9_PULMEM	0.417616	0.015608	pulmonary	TRUE	reported textfile	2	2.27E+08	0.00192	6.36E-09	557354	white blc	TRUE	reported igd	6.06E-05	33.78504	2	TRUE
rs2993986	T	C	T	C	0.030146	-0.04903	0.774092	0.808017	FALSE	FALSE	FALSE	I9_PULMEM	0.010084	0.019055	pulmonary	TRUE	reported textfile	10	28800023	0.00219	4.75E-43	562240	white blc	TRUE	reported igd	0.000337	189.4827	2	TRUE
rs301817	A	C	A	C	-0.01588	0.00252	0.582111	0.657335	FALSE	FALSE	FALSE	I9_PULMEM	0.873311	0.015806	pulmonary	TRUE	reported textfile	1	8503379	0.001868	2.02E-17	562213	white blc	TRUE	reported igd	0.000128	72.24974	2	TRUE
rs303753	A	G	A	G	-0.01959	0.01157	0.34473	0.35143	FALSE	FALSE	FALSE	I9_PULMEM	0.462481	0.015746	pulmonary	TRUE	reported textfile	18	21074922	0.00196	1.78E-23	562233	white blc	TRUE	reported igd	0.000178	99.86704	2	TRUE
rs309190	C	T	C	T	-0.03046	0.008388	0.893852	0.855382	FALSE	FALSE	FALSE	I9_PULMEM	0.695561	0.021436	pulmonary	TRUE	reported textfile	19	47629264	0.003167	7.05E-22	507330	white blc	TRUE	reported igd	0.000182	92.51038	2	TRUE
rs3184504	C	T	C	T	-0.06719	-0.0286	0.517084	0.59144	FALSE	FALSE	FALSE	I9_PULMEM	0.060823	0.015254	pulmonary	TRUE	reported textfile	12	1.12E+08	0.001836	########	559426	white blc	TRUE	reported igd	0.002388	1339.292	2	TRUE
rs342294	C	T	C	T	-0.01741	0.01569	0.455851	0.478389	FALSE	FALSE	FALSE	I9_PULMEM	0.295389	0.014995	pulmonary	TRUE	reported textfile	7	1.06E+08	0.001848	4.90E-21	558136	white blc	TRUE	reported igd	0.000159	88.72426	2	TRUE
rs3429835	T	C	T	C	-0.02421	-0.00066	0.123549	0.102195	FALSE	FALSE	FALSE	I9_PULMEM	0.979099	0.025037	pulmonary	TRUE	reported textfile	1	2.48E+08	0.002801	5.90E-18	561299	white blc	TRUE	reported igd	0.000133	74.67623	2	TRUE
rs3433200	T	C	T	C	-0.0107	0.014038	0.389704	0.351053	FALSE	FALSE	FALSE	I9_PULMEM	0.373812	0.015784	pulmonary	TRUE	reported textfile	2	2.34E+08	0.001888	1.52E-08	558138	white blc	TRUE	reported igd	5.75E-05	32.08899	2	TRUE
rs3450510	G	A	G	A	-0.02041	-0.03635	0.304866	0.278001	FALSE	FALSE	FALSE	I9_PULMEM	0.030892	0.016841	pulmonary	TRUE	reported textfile	22	24624609	0.002056	3.36E-23	562224	white blc	TRUE	reported igd	0.000175	98.54583	2	TRUE
rs3456273	A	G	A	G	0.018655	0.01325	0.77203	0.830206	FALSE	FALSE	FALSE	I9_PULMEM	0.506329	0.019938	pulmonary	TRUE	reported textfile	2	1.02E+08	0.002195	1.99E-17	558136	white blc	TRUE	reported igd	0.000129	72.23038	2	TRUE
rs3459908	T	C	T	C	-0.15148	0.201138	0.013363	0.006126	FALSE	FALSE	FALSE	I9_PULMEM	0.034186	0.094972	pulmonary	TRUE	reported textfile	1	1.59E+08	0.008129	1.93E-77	561453	white blc	TRUE	reported igd	0.000618	347.2486	2	TRUE
rs3495231	A	G	A	G	-0.0331	0.01788	0.050283	0.061164	FALSE	FALSE	FALSE	I9_PULMEM	0.573517	0.031765	pulmonary	TRUE	reported textfile	20	11177055	0.004378	4.24E-14	562240	white blc	TRUE	reported igd	0.000102	57.15105	2	TRUE
rs3511144	T	C	T	C	-0.01789	-0.02371	0.098264	0.07051	FALSE	FALSE	FALSE	I9_PULMEM	0.415889	0.029145	pulmonary	TRUE	reported textfile	17	62372190	0.003105	8.56E-09	562235	white blc	TRUE	reported igd	5.90E-05	33.18564	2	TRUE
rs3511294	A	G	A	G	-0.02597	-0.01137	0.215982	0.261946	FALSE	FALSE	FALSE	I9_PULMEM	0.505806	0.017085	pulmonary	TRUE	reported textfile	19	51738917	0.002241	5.21E-31	561448	white blc	TRUE	reported igd	0.000239	134.2739	2	TRUE
rs3557108	C	T	C	T	0.024903	0.028997	0.212693	0.230329	FALSE	FALSE	FALSE	I9_PULMEM	0.102786	0.017773	pulmonary	TRUE	reported textfile	1	2.25E+08	0.002243	1.32E-28	561156	white blc	TRUE	reported igd	0.00022	123.266	2	TRUE
rs3559243	C	G	C	G	0.039902	-0.132	0.027242	0.02496	FALSE	TRUE	FALSE	I9_PULMEM	0.008066	0.049823	pulmonary	TRUE	reported textfile	3	71355240	0.00608	5.47E-11	560386	white blc	TRUE	reported igd	7.69E-05	43.07057	2	TRUE
rs3559264	A	C	A	C	0.017479	0.033743	0.248719	0.197115	FALSE	FALSE	FALSE	I9_PULMEM	0.072566	0.018792	pulmonary	TRUE	reported textfile	17	4616387	0.00213	2.38E-16	561159	white blc	TRUE	reported igd	0.00012	67.33989	2	TRUE
rs3571388	T	C	T	C	0.028328	0.021685	0.043464	0.023703	FALSE	FALSE	FALSE	I9_PULMEM	0.661115	0.049468	pulmonary	TRUE	reported textfile	3	49162583	0.004568	5.79E-10	560509	white blc	TRUE	reported igd	6.86E-05	38.45724	2	TRUE
rs3573424	C	T	C	T	0.02176	0.004898	0.427987	0.453915	FALSE	FALSE	FALSE	I9_PULMEM	0.746605	0.015157	pulmonary	TRUE	reported textfile	4	706700	0.001948	6.30E-29	507479	white blc	TRUE	reported igd	0.000246	124.778	2	TRUE
rs3575586	T	C	T	C	-0.04149	-0.01789	0.034195	0.026657	FALSE	FALSE	FALSE	I9_PULMEM	0.703152	0.046945	pulmonary	TRUE	reported textfile	1	27140060	0.005077	3.18E-16	562241	white blc	TRUE	reported igd	0.000119	66.79022	2	TRUE
rs3731332	T	C	T	C	-0.1212	0.041051	0.023234	0.018182	FALSE	FALSE	FALSE	I9_PULMEM	0.467371	0.056484	pulmonary	TRUE	reported textfile	7	92300568	0.00624	5.53E-84	561302	white blc	TRUE	reported igd	0.000672	377.2483	2	TRUE
rs3732378	A	G	A	G	0.020274	0.007882	0.172487	0.159061	FALSE	FALSE	FALSE	I9_PULMEM	0.70142	0.020557	pulmonary	TRUE	reported textfile	3	39307162	0.00243	7.70E-17	560513	white blc	TRUE	reported igd	0.000124	69.6089	2	TRUE

rs3735485	G	A	G	A	0.041036	0.033871	0.844672	0.876107	FALSE	FALSE	FALSE	I9_PULMEM	0.136486	0.022747	pulmonary	TRUE	reported	textfile	7	45009341	0.00257	2.34E-57	561454	white blc	TRUE	reported	igd	0.000454	254.9542	2	TRUE
rs3747869	C	A	C	A	0.038842	-0.01835	0.900449	0.811551	FALSE	FALSE	FALSE	I9_PULMEM	0.343324	0.019361	pulmonary	TRUE	reported	textfile	10	73520632	0.00322	1.79E-33	503532	white blc	TRUE	reported	igd	0.000289	145.5089	2	TRUE
rs3752562	A	G	A	G	0.013547	-0.00068	0.774844	0.771912	FALSE	FALSE	FALSE	I9_PULMEM	0.969461	0.01786	pulmonary	TRUE	reported	textfile	20	42219831	0.002209	8.97E-10	557992	white blc	TRUE	reported	igd	6.74E-05	37.60913	2	TRUE
rs3754224	C	T	C	T	-0.01458	0.014417	0.270936	0.318878	FALSE	FALSE	FALSE	I9_PULMEM	0.370881	0.016112	pulmonary	TRUE	reported	textfile	1	43423622	0.002103	4.30E-12	562229	white blc	TRUE	reported	igd	8.54E-05	48.04589	2	TRUE
rs3773312	A	G	A	G	0.017874	0.000255	0.134067	0.146751	FALSE	FALSE	FALSE	I9_PULMEM	0.990451	0.021298	pulmonary	TRUE	reported	textfile	3	12946142	0.00285	3.67E-10	506685	white blc	TRUE	reported	igd	7.76E-05	39.33255	2	TRUE
rs3781454	A	G	A	G	0.025211	-0.01043	0.676766	0.590535	FALSE	FALSE	FALSE	I9_PULMEM	0.495473	0.015299	pulmonary	TRUE	reported	textfile	10	1.26E+08	0.002007	3.68E-36	562241	white blc	TRUE	reported	igd	0.000281	157.7916	2	TRUE
rs3793537	C	G	C	G	0.019834	-0.02074	0.290389	0.237272	FALSE	TRUE	FALSE	I9_PULMEM	0.240466	0.017666	pulmonary	TRUE	reported	textfile	9	35687556	0.002029	1.53E-22	562234	white blc	TRUE	reported	igd	0.00017	95.55534	2	TRUE
rs3795503	T	C	T	C	-0.01541	0.006107	0.314627	0.35507	FALSE	FALSE	FALSE	I9_PULMEM	0.697707	0.015724	pulmonary	TRUE	reported	textfile	1	1.81E+08	0.00201	1.83E-14	559077	white blc	TRUE	reported	igd	0.000105	58.76994	2	TRUE
rs3812849	C	A	C	A	0.01961	0.012813	0.266532	0.243082	FALSE	FALSE	FALSE	I9_PULMEM	0.466626	0.0176	pulmonary	TRUE	reported	textfile	13	74701736	0.002086	5.85E-21	562241	white blc	TRUE	reported	igd	0.000157	88.37411	2	TRUE
rs3847147	A	G	A	G	-0.01189	-0.01231	0.306976	0.302198	FALSE	FALSE	FALSE	I9_PULMEM	0.450675	0.016315	pulmonary	TRUE	reported	textfile	8	1.06E+08	0.002003	2.99E-09	560509	white blc	TRUE	reported	igd	6.29E-05	35.24304	2	TRUE
rs3857488	G	A	G	A	-0.01596	-0.00954	0.527618	0.510294	FALSE	FALSE	FALSE	I9_PULMEM	0.524862	0.014997	pulmonary	TRUE	reported	textfile	6	88001064	0.001847	5.95E-18	558142	white blc	TRUE	reported	igd	0.000134	74.68601	2	TRUE
rs38859	T	C	T	C	0.011328	-0.0189	0.448861	0.390388	FALSE	FALSE	FALSE	I9_PULMEM	0.218829	0.015368	pulmonary	TRUE	reported	textfile	7	1.16E+08	0.001851	9.71E-10	560513	white blc	TRUE	reported	igd	6.68E-05	37.45347	2	TRUE
rs399757	C	T	C	T	0.013512	0.019281	0.382674	0.329528	FALSE	FALSE	FALSE	I9_PULMEM	0.230466	0.016079	pulmonary	TRUE	reported	textfile	22	18295575	0.00199	1.16E-11	507333	white blc	TRUE	reported	igd	9.09E-05	46.10324	2	TRUE
rs4074672	T	C	T	C	0.018004	0.030939	0.369536	0.294356	FALSE	FALSE	FALSE	I9_PULMEM	0.059907	0.016444	pulmonary	TRUE	reported	textfile	3	1.84E+08	0.001907	3.87E-21	560510	white blc	TRUE	reported	igd	0.000159	89.13229	2	TRUE
rs4127253	G	A	G	A	-0.03444	-0.00506	0.045737	0.03776	FALSE	FALSE	FALSE	I9_PULMEM	0.898508	0.039637	pulmonary	TRUE	reported	textfile	1	1.83E+08	0.004735	3.65E-13	508419	white blc	TRUE	reported	igd	0.000104	52.91274	2	TRUE
rs4129505	T	C	T	C	-0.01375	0.04118	0.186956	0.149938	FALSE	FALSE	FALSE	I9_PULMEM	0.050789	0.021083	pulmonary	TRUE	reported	textfile	10	6111622	0.002349	4.95E-09	562241	white blc	TRUE	reported	igd	6.09E-05	34.259	2	TRUE
rs4131338	A	C	A	C	0.048456	0.029334	0.0304	0.017483	FALSE	FALSE	FALSE	I9_PULMEM	0.610536	0.057596	pulmonary	TRUE	reported	textfile	1	79411968	0.005508	1.47E-18	505608	white blc	TRUE	reported	igd	0.000153	77.39369	2	TRUE
rs4131701	T	C	T	C	-0.02008	0.024003	0.247562	0.265175	FALSE	FALSE	FALSE	I9_PULMEM	0.162818	0.017198	pulmonary	TRUE	reported	textfile	9	1.4E+08	0.002239	3.25E-19	508416	white blc	TRUE	reported	igd	0.000158	80.38985	2	TRUE
rs4239149	G	A	G	A	0.016302	-0.00722	0.656343	0.614482	FALSE	FALSE	FALSE	I9_PULMEM	0.640304	0.01545	pulmonary	TRUE	reported	textfile	17	41328095	0.001945	5.58E-17	560511	white blc	TRUE	reported	igd	0.000125	70.24914	2	TRUE
rs4276676	C	T	C	T	-0.01926	0.003631	0.469209	0.371537	FALSE	FALSE	FALSE	I9_PULMEM	0.815079	0.015526	pulmonary	TRUE	reported	textfile	8	68795618	0.001849	2.22E-25	562240	white blc	TRUE	reported	igd	0.000193	108.5469	2	TRUE
rs430989	G	T	G	T	-0.01419	-0.01852	0.269456	0.370278	FALSE	FALSE	FALSE	I9_PULMEM	0.236208	0.015632	pulmonary	TRUE	reported	textfile	19	49120774	0.002164	5.67E-11	555166	white blc	TRUE	reported	igd	7.74E-05	42.99199	2	TRUE
rs4391200	G	A	G	A	0.022974	-0.01632	0.618964	0.656731	FALSE	FALSE	FALSE	I9_PULMEM	0.301354	0.015793	pulmonary	TRUE	reported	textfile	5	1.42E+08	0.001894	7.86E-34	561145	white blc	TRUE	reported	igd	0.000262	147.1336	2	TRUE
rs4413892	A	G	A	G	0.031056	0.000505	0.276136	0.252654	FALSE	FALSE	FALSE	I9_PULMEM	0.976676	0.017261	pulmonary	TRUE	reported	textfile	9	1.39E+08	0.002174	2.87E-46	517656	white blc	TRUE	reported	igd	0.000394	204.0658	2	TRUE
rs4468717	T	C	T	C	-0.02001	0.024196	0.077837	0.138231	FALSE	FALSE	FALSE	I9_PULMEM	0.274114	0.022124	pulmonary	TRUE	reported	textfile	18	3457606	0.003471	8.41E-09	560512	white blc	TRUE	reported	igd	5.93E-05	33.22742	2	TRUE
rs447124	T	C	T	C	0.016995	0.01033	0.490566	0.560154	FALSE	FALSE	FALSE	I9_PULMEM	0.495716	0.015163	pulmonary	TRUE	reported	textfile	9	4746539	0.001853	4.86E-20	561301	white blc	TRUE	reported	igd	0.00015	84.1182	2	TRUE
rs4535497	A	C	A	C	-0.01332	0.006775	0.570032	0.61981	FALSE	FALSE	FALSE	I9_PULMEM	0.661791	0.015488	pulmonary	TRUE	reported	textfile	5	1107428	0.001992	2.34E-11	488908	white blc	TRUE	reported	igd	9.15E-05	44.73255	2	TRUE
rs464609	A	G	A	G	0.012934	0.019263	0.544492	0.54392	FALSE	FALSE	FALSE	I9_PULMEM	0.200341	0.015042	pulmonary	TRUE	reported	textfile	5	34654477	0.001852	3.00E-12	557351	white blc	TRUE	reported	igd	8.75E-05	48.77331	2	TRUE
rs4672564	C	A	C	A	-0.01019	0.006999	0.500893	0.48183	FALSE	FALSE	FALSE	I9_PULMEM	0.641563	0.015036	pulmonary	TRUE	reported	textfile	2	2.11E+08	0.001848	3.67E-08	558143	white blc	TRUE	reported	igd	5.44E-05	30.37502	2	TRUE
rs4703541	A	G	A	G	-0.03619	-0.01526	0.878793	0.875916	FALSE	FALSE	FALSE	I9_PULMEM	0.501785	0.022714	pulmonary	TRUE	reported	textfile	5	71743755	0.002813	7.45E-38	562242	white blc	TRUE	reported	igd	0.000294	165.5328	2	TRUE
rs4704826	A	C	A	C	-0.0156	0.028918	0.633681	0.665757	FALSE	FALSE	FALSE	I9_PULMEM	0.070601	0.015994	pulmonary	TRUE	reported	textfile	5	1.56E+08	0.001876	9.71E-17	562239	white blc	TRUE	reported	igd	0.000123	69.15724	2	TRUE
rs4707609	C	T	C	T	-0.02109	0.02536	0.360123	0.26497	FALSE	FALSE	FALSE	I9_PULMEM	0.135698	0.016997	pulmonary	TRUE	reported	textfile	6	90946479	0.001914	3.21E-28	560507	white blc	TRUE	reported	igd	0.000217	121.4367	2	TRUE
rs4721650	G	A	G	A	0.015196	0.009048	0.209572	0.160193	FALSE	FALSE	FALSE	I9_PULMEM	0.658867	0.020496	pulmonary	TRUE	reported	textfile	7	2410151	0.002296	3.76E-11	562239	white blc	TRUE	reported	igd	7.79E-05	43.80395	2	TRUE
rs4729046	C	T	C	T	-0.03661	-0.00382	0.924065	0.913171	FALSE	FALSE	FALSE	I9_PULMEM	0.886431	0.026715	pulmonary	TRUE	reported	textfile	7	92223957	0.003489	1.01E-25	562243	white blc	TRUE	reported	igd	0.000196	110.0721	2	TRUE
rs47341	T	C	T	C	0.015865	0.000456	0.396441	0.370223	FALSE	FALSE	FALSE	I9_PULMEM	0.976585	0.01553	pulmonary	TRUE	reported	textfile	22	43560763	0.001975	1.01E-15	506394	white blc	TRUE	reported	igd	0.000127	64.52741	2	TRUE
rs4734879	G	A	G	A	-0.02197	-0.03689	0.276159	0.221988	FALSE	FALSE	FALSE	I9_PULMEM	0.042416	0.01818	pulmonary	TRUE	reported	textfile	8	1.07E+08	0.002057	1.34E-26	560514	white blc	TRUE	reported	igd	0.000204	114.1164	2	TRUE
rs4760	G	A	G	A	-0.07023	0.019451	0.152719	0.167591	FALSE	FALSE	FALSE	I9_PULMEM	0.337875	0.020296	pulmonary	TRUE	reported	textfile	19	44153100	0.002594	########	560511	white blc	TRUE	reported	igd	0.001306	733.0833	2	TRUE
rs4761234	C	T	C	T	0.015191	0.015702	0.4824	0.412369	FALSE	FALSE	FALSE	I9_PULMEM	0.303279	0.015253	pulmonary	TRUE	reported	textfile	12	69732105	0.001847	2.03E-16	562240	white blc	TRUE	reported	igd	0.00012	67.64526	2	TRUE
rs4789229	T	C	T	C	0.011726	-0.0411	0.644533	0.726522	FALSE	FALSE	FALSE	I9_PULMEM	0.014619	0.016833	pulmonary	TRUE	reported	textfile	17	73796002	0.001926	1.18E-09	562240	white blc	TRUE	reported	igd	6.59E-05	37.06685	2	TRUE
rs4790752	T	C	T	C	-0.01754	0.001313	0.450783	0.343649	FALSE	FALSE	FALSE	I9_PULMEM	0.934018	0.015862	pulmonary	TRUE	reported	textfile	17	5115877	0.001845	2.11E-21	562232	white blc	TRUE	reported	igd	0.000161	90.36815	2	TRUE
rs4794820	G	A	G	A	0.07212	-0.00395	0.552137	0.52571	FALSE	FALSE	FALSE	I9_PULMEM	0.792914	0.015044	pulmonary	TRUE	reported	textfile	17	38089344	0.001861	########	561295	white blc	TRUE	reported	igd	0.002668	1501.817	2	TRUE
rs4805881	C	A	C	A	-0.01642	0.016882	0.665284	0.6578	FALSE	FALSE	FALSE	I9_PULMEM	0.285299	0.0158	pulmonary	TRUE	reported	textfile	19	33896432	0.001984	1.32E-16	562242	white blc	TRUE	reported	igd	0.000122	68.4954	2	TRUE
rs4844390	G	A	G	A	-0.02655	-0.02124	0.220218	0.219918	FALSE	FALSE	FALSE	I9_PULMEM	0.240574	0.018095	pulmonary	TRUE	reported	textfile	1	2.08E+08	0.002216	4.97E-33	561301	white blc	TRUE	reported	igd	0.000256	143.5342	2	TRUE
rs4844565	T	A	T	A	-0.0114	-0.00614	0.410019	0.406736	FALSE	TRUE	FALSE	I9_PULMEM	0.688208	0.015308	pulmonary	TRUE	reported	textfile	1	2.07E+08	0.001866	1.01E-09	562236	white blc	TRUE	reported	igd	6.64E-05	37.34336	2	TRUE
rs4877107	C	T	C	T	0.010359	-0.01793	0.457254	0.465577	FALSE	FALSE	FALSE	I9_PULMEM	0.233959	0.015065	pulmonary	TRUE	reported	textfile	9	91383601	0.001856	2.45E-08	560512	white blc	TRUE	reported	igd	5.56E-05	31.15144	2	TRUE

rs4909932	G	A	G	A	0.013829	0.006243	0.59811	0.570502	FALSE	FALSE	FALSE	I9_PULMEM	0.680359	0.015153	pulmonary	TRUE	reported	textfile	11	10475967	0.001881	2.01E-13	559426	white blc	TRUE	reported	igd	9.66E-05	54.05084	2	TRUE
rs4909945	C	T	C	T	0.011456	0.012879	0.688902	0.669595	FALSE	FALSE	FALSE	I9_PULMEM	0.418244	0.015911	pulmonary	TRUE	reported	textfile	11	10673739	0.001986	8.23E-09	560509	white blc	TRUE	reported	igd	5.94E-05	33.27407	2	TRUE
rs4924450	A	G	A	G	0.01369	-0.0121	0.706498	0.78409	FALSE	FALSE	FALSE	I9_PULMEM	0.510632	0.018387	pulmonary	TRUE	reported	textfile	15	40597229	0.002122	1.14E-10	508419	white blc	TRUE	reported	igd	8.19E-05	41.62118	2	TRUE
rs4925756	T	C	T	C	-0.01456	0.011622	0.691881	0.721822	FALSE	FALSE	FALSE	I9_PULMEM	0.489822	0.016828	pulmonary	TRUE	reported	textfile	1	2.48E+08	0.002004	3.83E-13	561154	white blc	TRUE	reported	igd	9.41E-05	52.80861	2	TRUE
rs4970996	C	G	C	G	0.0154	-0.02974	0.750743	0.794506	FALSE	TRUE	FALSE	I9_PULMEM	0.113957	0.018813	pulmonary	TRUE	reported	textfile	1	1.51E+08	0.002154	8.97E-13	562241	white blc	TRUE	reported	igd	9.09E-05	51.11502	2	TRUE
rs507778	T	C	T	C	-0.05281	-0.01421	0.333105	0.258343	FALSE	FALSE	FALSE	I9_PULMEM	0.40729	0.017151	pulmonary	TRUE	reported	textfile	6	32209861	0.001961	########	556052	white blc	TRUE	reported	igd	0.001302	725.1198	2	TRUE
rs520910	C	T	C	T	-0.02476	-0.01785	0.067393	0.053213	FALSE	FALSE	FALSE	I9_PULMEM	0.594918	0.033562	pulmonary	TRUE	reported	textfile	1	1.56E+08	0.003685	1.87E-11	562239	white blc	TRUE	reported	igd	8.03E-05	45.16116	2	TRUE
rs527393	A	G	A	G	-0.02209	0.002409	0.467803	0.457963	FALSE	FALSE	FALSE	I9_PULMEM	0.873007	0.015071	pulmonary	TRUE	reported	textfile	2	1.7E+08	0.001842	4.34E-33	558138	white blc	TRUE	reported	igd	0.000258	143.8171	2	TRUE
rs533483	A	G	A	G	-0.0174	-0.01186	0.243389	0.192282	FALSE	FALSE	FALSE	I9_PULMEM	0.534172	0.019078	pulmonary	TRUE	reported	textfile	1	2.35E+08	0.002188	1.91E-15	557350	white blc	TRUE	reported	igd	0.000113	63.23425	2	TRUE
rs549302	A	G	A	G	0.020151	-0.00257	0.545007	0.531254	FALSE	FALSE	FALSE	I9_PULMEM	0.863994	0.015009	pulmonary	TRUE	reported	textfile	6	1.23E+08	0.001843	8.48E-28	562242	white blc	TRUE	reported	igd	0.000213	119.5476	2	TRUE
rs5567171	T	C	T	C	0.018588	-0.0398	0.095812	0.199645	FALSE	FALSE	FALSE	I9_PULMEM	0.033925	0.018765	pulmonary	TRUE	reported	textfile	1	1.7E+08	0.003119	2.59E-09	562240	white blc	TRUE	reported	igd	6.32E-05	35.51673	2	TRUE
rs5576780	C	T	C	T	-0.01838	-0.01359	0.632257	0.598946	FALSE	FALSE	FALSE	I9_PULMEM	0.374752	0.01531	pulmonary	TRUE	reported	textfile	17	17674967	0.002003	4.71E-20	508419	white blc	TRUE	reported	igd	0.000166	84.18465	2	TRUE
rs5596481	C	T	C	T	-0.03625	0.014137	0.567337	0.579086	FALSE	FALSE	FALSE	I9_PULMEM	0.352617	0.015209	pulmonary	TRUE	reported	textfile	8	1.31E+08	0.00186	1.54E-84	562237	white blc	TRUE	reported	igd	0.000675	379.9132	2	TRUE
rs5597899	A	C	A	C	0.013869	0.012361	0.16913	0.097659	FALSE	FALSE	FALSE	I9_PULMEM	0.624976	0.025288	pulmonary	TRUE	reported	textfile	13	73626897	0.002466	1.90E-08	562214	white blc	TRUE	reported	igd	5.63E-05	31.63025	2	TRUE
rs560194	C	T	C	T	-0.01359	0.010827	0.530014	0.511278	FALSE	FALSE	FALSE	I9_PULMEM	0.471649	0.015042	pulmonary	TRUE	reported	textfile	6	10528794	0.001843	1.76E-13	559430	white blc	TRUE	reported	igd	9.71E-05	54.34142	2	TRUE
rs5618886	C	T	C	T	-0.02701	0.035779	0.374222	0.358899	FALSE	FALSE	FALSE	I9_PULMEM	0.022638	0.015696	pulmonary	TRUE	reported	textfile	1	2.48E+08	0.001899	7.00E-46	561158	white blc	TRUE	reported	igd	0.00036	202.3608	2	TRUE
rs5637871	G	A	G	A	0.107631	0.082825	0.01278	0.032123	FALSE	FALSE	FALSE	I9_PULMEM	0.05262	0.042737	pulmonary	TRUE	reported	textfile	17	56356502	0.008291	1.70E-38	561450	white blc	TRUE	reported	igd	0.0003	168.5231	2	TRUE
rs5638817	T	G	T	G	0.059326	-0.01178	0.294509	0.283397	FALSE	FALSE	FALSE	I9_PULMEM	0.4813	0.016731	pulmonary	TRUE	reported	textfile	7	28724374	0.002043	########	561153	white blc	TRUE	reported	igd	0.0015	843.2413	2	TRUE
rs571497	A	G	A	G	-0.03636	0.035427	0.154843	0.124651	FALSE	FALSE	FALSE	I9_PULMEM	0.119376	0.022748	pulmonary	TRUE	reported	textfile	19	7827830	0.002584	6.32E-45	559429	white blc	TRUE	reported	igd	0.000354	197.9655	2	TRUE
rs5746451	C	T	C	T	0.019536	-0.00676	0.504194	0.497725	FALSE	FALSE	FALSE	I9_PULMEM	0.651989	0.014984	pulmonary	TRUE	reported	textfile	22	18126020	0.001849	4.61E-26	561295	white blc	TRUE	reported	igd	0.000199	111.6339	2	TRUE
rs5746990	A	G	A	G	-0.01512	-0.04107	0.622693	0.588397	FALSE	FALSE	FALSE	I9_PULMEM	0.006925	0.015209	pulmonary	TRUE	reported	textfile	22	17563536	0.001903	2.05E-15	562236	white blc	TRUE	reported	igd	0.000112	63.1033	2	TRUE
rs5787117	C	T	C	T	-0.01331	-0.04617	0.17625	0.176385	FALSE	FALSE	FALSE	I9_PULMEM	0.019164	0.019709	pulmonary	TRUE	reported	textfile	2	85425048	0.002414	3.55E-08	562237	white blc	TRUE	reported	igd	5.41E-05	30.41873	2	TRUE
rs579721	A	G	A	G	-0.01229	0.005043	0.283654	0.222772	FALSE	FALSE	FALSE	I9_PULMEM	0.77892	0.017966	pulmonary	TRUE	reported	textfile	11	59834318	0.002033	1.55E-09	562243	white blc	TRUE	reported	igd	6.50E-05	36.52712	2	TRUE
rs5908506	G	A	G	A	-0.05029	0.008831	0.040472	0.073291	FALSE	FALSE	FALSE	I9_PULMEM	0.760873	0.029017	pulmonary	TRUE	reported	textfile	10	89681458	0.004692	9.00E-27	559446	white blc	TRUE	reported	igd	0.000205	114.8895	2	TRUE
rs5924131	A	G	A	G	-0.01718	0.028769	0.099621	0.079385	FALSE	FALSE	FALSE	I9_PULMEM	0.298555	0.027674	pulmonary	TRUE	reported	textfile	12	2506038	0.00307	2.24E-08	561302	white blc	TRUE	reported	igd	5.58E-05	31.31613	2	TRUE
rs6019048	C	T	C	T	-0.0314	-0.06503	0.057277	0.049577	FALSE	FALSE	FALSE	I9_PULMEM	0.061396	0.034763	pulmonary	TRUE	reported	textfile	2	70385245	0.003962	2.43E-15	562202	white blc	TRUE	reported	igd	0.000112	62.79	2	TRUE
rs6029234	C	G	C	G	0.024169	0.019199	0.624198	0.595929	FALSE	TRUE	FALSE	I9_PULMEM	0.209043	0.015284	pulmonary	TRUE	reported	textfile	20	39259278	0.001905	7.57E-37	561154	white blc	TRUE	reported	igd	0.000287	160.9629	2	TRUE
rs6045615	C	A	C	A	-0.01932	0.010358	0.330205	0.306886	FALSE	FALSE	FALSE	I9_PULMEM	0.523279	0.016227	pulmonary	TRUE	reported	textfile	20	1931582	0.001982	1.99E-22	560509	white blc	TRUE	reported	igd	0.000169	95.00806	2	TRUE
rs6046684	A	C	A	C	-0.02972	0.009406	0.087587	0.055587	FALSE	FALSE	FALSE	I9_PULMEM	0.773548	0.03269	pulmonary	TRUE	reported	textfile	7	8024840	0.003269	1.04E-19	562237	white blc	TRUE	reported	igd	0.000147	82.6322	2	TRUE
rs6051431	T	C	T	C	0.011858	0.004372	0.351093	0.366833	FALSE	FALSE	FALSE	I9_PULMEM	0.778386	0.015535	pulmonary	TRUE	reported	textfile	20	2831990	0.001931	8.44E-10	562230	white blc	TRUE	reported	igd	6.71E-05	37.71002	2	TRUE
rs6055955	T	C	T	C	-0.0222	-0.00551	0.508614	0.485191	FALSE	FALSE	FALSE	I9_PULMEM	0.713587	0.015001	pulmonary	TRUE	reported	textfile	20	8604181	0.001845	2.63E-33	560513	white blc	TRUE	reported	igd	0.000258	144.8071	2	TRUE
rs6062468	T	C	T	C	0.010809	-0.02588	0.508105	0.441005	FALSE	FALSE	FALSE	I9_PULMEM	0.088227	0.01518	pulmonary	TRUE	reported	textfile	20	62268955	0.001937	2.46E-08	506687	white blc	TRUE	reported	igd	6.15E-05	31.13939	2	TRUE
rs6069534	T	C	T	C	-0.0299	0.009234	0.197348	0.215851	FALSE	FALSE	FALSE	I9_PULMEM	0.612439	0.018228	pulmonary	TRUE	reported	textfile	15	51010271	0.002312	3.20E-38	562209	white blc	TRUE	reported	igd	0.000297	167.2608	2	TRUE
rs6173928	T	C	T	C	-0.0337	-0.02133	0.033259	0.05242	FALSE	FALSE	FALSE	I9_PULMEM	0.528354	0.033836	pulmonary	TRUE	reported	textfile	16	27480797	0.005207	9.98E-11	560511	white blc	TRUE	reported	igd	7.47E-05	41.88488	2	TRUE
rs6186376	T	C	T	C	0.043116	-0.02275	0.596247	0.582357	FALSE	FALSE	FALSE	I9_PULMEM	0.135648	0.015244	pulmonary	TRUE	reported	textfile	10	99084426	0.001874	########	562236	white blc	TRUE	reported	igd	0.000941	529.3418	2	TRUE
rs6200717	C	T	C	T	-0.01545	0.01272	0.291138	0.330951	FALSE	FALSE	FALSE	I9_PULMEM	0.425209	0.015951	pulmonary	TRUE	reported	textfile	15	77306285	0.002058	6.22E-14	562230	white blc	TRUE	reported	igd	0.0001	56.36648	2	TRUE
rs6201935	C	T	C	T	0.011729	-0.01359	0.27272	0.206695	FALSE	FALSE	FALSE	I9_PULMEM	0.463416	0.018528	pulmonary	TRUE	reported	textfile	15	90933683	0.002072	1.54E-08	562243	white blc	TRUE	reported	igd	5.70E-05	32.04357	2	TRUE
rs6208969	A	G	A	G	0.017652	-0.01247	0.172097	0.171945	FALSE	FALSE	FALSE	I9_PULMEM	0.530658	0.019892	pulmonary	TRUE	reported	textfile	17	2726325	0.002518	2.48E-12	562238	white blc	TRUE	reported	igd	8.74E-05	49.14449	2	TRUE
rs6219450	A	G	A	G	-0.01088	-0.00714	0.382331	0.43377	FALSE	FALSE	FALSE	I9_PULMEM	0.63758	0.015153	pulmonary	TRUE	reported	textfile	2	2.39E+08	0.001937	2.00E-08	557048	white blc	TRUE	reported	igd	5.66E-05	31.53243	2	TRUE
rs6227093	T	C	T	C	0.016035	0.006264	0.177104	0.107742	FALSE	FALSE	FALSE	I9_PULMEM	0.795851	0.02421	pulmonary	TRUE	reported	textfile	3	1.28E+08	0.002454	6.59E-11	561154	white blc	TRUE	reported	igd	7.61E-05	42.69601	2	TRUE
rs6231139	G	A	G	A	-0.02525	-0.07141	0.061901	0.059718	FALSE	FALSE	FALSE	I9_PULMEM	0.026213	0.032123	pulmonary	TRUE	reported	textfile	4	83406211	0.003841	5.09E-11	561301	white blc	TRUE	reported	igd	7.70E-05	43.20454	2	TRUE
rs6234514	C	G	C	G	-0.03408	0.003771	0.024821	0.007778	FALSE	TRUE	FALSE	I9_PULMEM	0.965156	0.086315	pulmonary	TRUE	reported	textfile	4	1.46E+08	0.006181	3.58E-08	561295	white blc	TRUE	reported	igd	5.42E-05	30.4076	2	TRUE
rs6246631	T	C	T	C	-0.02121	0.023742	0.202209	0.192502	FALSE	FALSE	FALSE	I9_PULMEM	0.212142	0.019028	pulmonary	TRUE	reported	textfile	7	73042085	0.002399	9.80E-19	508416	white blc	TRUE	reported	igd	0.000154	78.19571	2	TRUE
rs6251026	G	A	G	A	-0.03609	-0.0097	0.14573	0.150267	FALSE	FALSE	FALSE	I9_PULMEM	0.643674	0.020967	pulmonary	TRUE	reported	textfile	8	79012343	0.002609	1.77E-43	562239	white blc	TRUE	reported	igd	0.00034	191.3693	2	TRUE

rs626416	C	G	C	G	-0.01661	0.007511	0.762943	0.809558	FALSE	TRUE	FALSE	I9_PULMEM	0.6941	0.019097	pulmonary	TRUE	reported	textfile	9	79326680	0.002181	2.81E-14	547355	white blc	TRUE	reported	igd	0.000106	57.96487	2	TRUE
rs630505	C	T	C	T	0.012464	-0.01618	0.265394	0.251651	FALSE	FALSE	FALSE	I9_PULMEM	0.351222	0.017357	pulmonary	TRUE	reported	textfile	1	1.12E+08	0.002102	3.13E-09	560509	white blc	TRUE	reported	igd	6.27E-05	35.15992	2	TRUE
rs632887	G	A	G	A	0.01204	0.016519	0.411004	0.385539	FALSE	FALSE	FALSE	I9_PULMEM	0.285524	0.015468	pulmonary	TRUE	reported	textfile	12	3392351	0.001921	3.75E-10	560507	white blc	TRUE	reported	igd	7.01E-05	39.28228	2	TRUE
rs6421984	C	T	C	T	0.038666	-0.03182	0.514549	0.433185	FALSE	FALSE	FALSE	I9_PULMEM	0.036418	0.015211	pulmonary	TRUE	reported	textfile	11	305619	0.001952	2.79E-87	500261	white blc	TRUE	reported	igd	0.000784	392.3712	2	TRUE
rs6426584	T	A	T	A	-0.01152	-0.02246	0.334952	0.242662	FALSE	TRUE	FALSE	I9_PULMEM	0.19963	0.017511	pulmonary	TRUE	reported	textfile	1	2.27E+08	0.001951	3.66E-09	562239	white blc	TRUE	reported	igd	6.20E-05	34.86494	2	TRUE
rs6429582	C	T	C	T	-0.01882	0.000116	0.435077	0.421787	FALSE	FALSE	FALSE	I9_PULMEM	0.993922	0.015203	pulmonary	TRUE	reported	textfile	1	46321843	0.001853	3.25E-24	557354	white blc	TRUE	reported	igd	0.000185	103.1871	2	TRUE
rs6440053	A	T	A	T	-0.01413	0.026215	0.842254	0.823941	FALSE	TRUE	FALSE	I9_PULMEM	0.18344	0.019707	pulmonary	TRUE	reported	textfile	3	1.42E+08	0.002541	2.76E-08	561158	white blc	TRUE	reported	igd	5.51E-05	30.91366	2	TRUE
rs6440732	A	C	A	C	0.023275	-0.02833	0.827718	0.836785	FALSE	FALSE	FALSE	I9_PULMEM	0.162265	0.020273	pulmonary	TRUE	reported	textfile	3	1.51E+08	0.002437	1.35E-21	560513	white blc	TRUE	reported	igd	0.000163	91.21511	2	TRUE
rs6445826	C	T	C	T	0.01247	0.011263	0.498475	0.556726	FALSE	FALSE	FALSE	I9_PULMEM	0.459453	0.015226	pulmonary	TRUE	reported	textfile	3	56848999	0.001887	4.03E-11	561148	white blc	TRUE	reported	igd	7.78E-05	43.67044	2	TRUE
rs6458354	T	C	T	C	0.014462	-0.00015	0.707553	0.688954	FALSE	FALSE	FALSE	I9_PULMEM	0.992734	0.016212	pulmonary	TRUE	reported	textfile	6	43814190	0.00202	8.36E-13	562236	white blc	TRUE	reported	igd	9.12E-05	51.25691	2	TRUE
rs6474514	C	T	C	T	0.011055	0.000722	0.41173	0.472527	FALSE	FALSE	FALSE	I9_PULMEM	0.961801	0.015072	pulmonary	TRUE	reported	textfile	8	38835269	0.001872	3.59E-09	557353	white blc	TRUE	reported	igd	6.26E-05	34.8742	2	TRUE
rs6476883	A	G	A	G	-0.01149	-0.00471	0.437247	0.344588	FALSE	FALSE	FALSE	I9_PULMEM	0.765098	0.015778	pulmonary	TRUE	reported	textfile	9	4654774	0.001857	6.27E-10	562241	white blc	TRUE	reported	igd	6.81E-05	38.2971	2	TRUE
rs6479830	C	T	C	T	-0.01241	-0.00982	0.715242	0.726781	FALSE	FALSE	FALSE	I9_PULMEM	0.560973	0.016892	pulmonary	TRUE	reported	textfile	10	64418739	0.002033	1.05E-09	561158	white blc	TRUE	reported	igd	6.64E-05	37.28612	2	TRUE
rs6500550	T	C	T	C	-0.02072	0.009223	0.304495	0.289731	FALSE	FALSE	FALSE	I9_PULMEM	0.576874	0.016529	pulmonary	TRUE	reported	textfile	16	3746241	0.002036	2.65E-24	557349	white blc	TRUE	reported	igd	0.000186	103.5672	2	TRUE
rs6502608	A	G	A	G	0.014711	-0.01045	0.707085	0.675273	FALSE	FALSE	FALSE	I9_PULMEM	0.514809	0.016038	pulmonary	TRUE	reported	textfile	17	1316427	0.002078	1.50E-12	560833	white blc	TRUE	reported	igd	8.94E-05	50.11777	2	TRUE
rs6583435	A	C	A	C	0.016915	0.009105	0.679683	0.725126	FALSE	FALSE	FALSE	I9_PULMEM	0.588293	0.01682	pulmonary	TRUE	reported	textfile	7	50293405	0.001968	8.70E-18	562242	white blc	TRUE	reported	igd	0.000131	73.87411	2	TRUE
rs6590806	A	G	A	G	0.01608	-0.00293	0.250457	0.220951	FALSE	FALSE	FALSE	I9_PULMEM	0.871304	0.018095	pulmonary	TRUE	reported	textfile	11	1.01E+08	0.002128	4.28E-14	559070	white blc	TRUE	reported	igd	0.000102	57.09884	2	TRUE
rs6667709	T	C	T	C	-0.01384	0.000692	0.370088	0.410556	FALSE	FALSE	FALSE	I9_PULMEM	0.963958	0.015316	pulmonary	TRUE	reported	textfile	1	42201943	0.001923	6.30E-13	557349	white blc	TRUE	reported	igd	9.30E-05	51.82783	2	TRUE
rs6679677	A	C	A	C	-0.05184	0.00222	0.099992	0.146443	FALSE	FALSE	FALSE	I9_PULMEM	0.917043	0.02131	pulmonary	TRUE	reported	textfile	1	1.14E+08	0.003052	1.14E-64	558294	white blc	TRUE	reported	igd	0.000516	288.4979	2	TRUE
rs6684709	C	G	C	G	-0.02059	0.007516	0.620492	0.712507	FALSE	TRUE	FALSE	I9_PULMEM	0.649943	0.016561	pulmonary	TRUE	reported	textfile	1	23850023	0.001895	1.84E-27	561156	white blc	TRUE	reported	igd	0.00021	118.0458	2	TRUE
rs672058	T	C	T	C	0.018163	-0.01865	0.880458	0.813715	FALSE	FALSE	FALSE	I9_PULMEM	0.334555	0.019325	pulmonary	TRUE	reported	textfile	11	1.17E+08	0.002846	1.81E-10	560510	white blc	TRUE	reported	igd	7.27E-05	40.72901	2	TRUE
rs6731993	T	A	T	A	0.022535	-0.0036	0.407782	0.337415	FALSE	TRUE	FALSE	I9_PULMEM	0.821137	0.015911	pulmonary	TRUE	reported	textfile	2	65642097	0.001877	3.70E-33	562235	white blc	TRUE	reported	igd	0.000256	144.1402	2	TRUE
rs6734238	G	A	G	A	0.036475	-0.02805	0.402192	0.3032	FALSE	FALSE	FALSE	I9_PULMEM	0.086286	0.016353	pulmonary	TRUE	reported	textfile	2	1.14E+08	0.001959	2.59E-77	503530	white blc	TRUE	reported	igd	0.000688	346.673	2	TRUE
rs6749187	C	A	C	A	0.011131	-0.00441	0.60436	0.638043	FALSE	FALSE	FALSE	I9_PULMEM	0.777702	0.015639	pulmonary	TRUE	reported	textfile	18	78003207	0.001914	6.15E-09	562238	white blc	TRUE	reported	igd	6.02E-05	33.82073	2	TRUE
rs6755895	C	T	C	T	-0.02333	0.044877	0.231769	0.215181	FALSE	FALSE	FALSE	I9_PULMEM	0.0141	0.018282	pulmonary	TRUE	reported	textfile	2	2.33E+08	0.002239	2.14E-25	558136	white blc	TRUE	reported	igd	0.000194	108.5446	2	TRUE
rs6768491	G	C	G	C	-0.0225	0.005718	0.19513	0.210477	FALSE	TRUE	FALSE	I9_PULMEM	0.758316	0.018584	pulmonary	TRUE	reported	textfile	12	4322852	0.002347	9.67E-22	561158	white blc	TRUE	reported	igd	0.000164	91.90461	2	TRUE
rs6779340	G	C	G	C	-0.01751	0.021879	0.336941	0.29803	FALSE	TRUE	FALSE	I9_PULMEM	0.183163	0.016437	pulmonary	TRUE	reported	textfile	3	58033701	0.001961	4.51E-19	561300	white blc	TRUE	reported	igd	0.000142	79.73797	2	TRUE
rs6831590	T	G	T	G	-0.0184	-0.03694	0.367282	0.392018	FALSE	FALSE	FALSE	I9_PULMEM	0.016185	0.015363	pulmonary	TRUE	reported	textfile	4	6943776	0.001904	4.54E-22	562238	white blc	TRUE	reported	igd	0.000166	93.38997	2	TRUE
rs6854855	G	A	G	A	0.016404	0.023192	0.486909	0.443016	FALSE	FALSE	FALSE	I9_PULMEM	0.125473	0.015137	pulmonary	TRUE	reported	textfile	4	1.52E+08	0.001855	9.69E-19	562240	white blc	TRUE	reported	igd	0.000139	78.20061	2	TRUE
rs6878780	C	T	C	T	0.013254	-0.02481	0.426771	0.528423	FALSE	FALSE	FALSE	I9_PULMEM	0.099156	0.015044	pulmonary	TRUE	reported	textfile	5	1.22E+08	0.001858	1.02E-12	562219	white blc	TRUE	reported	igd	9.05E-05	50.88631	2	TRUE
rs6924387	G	A	G	A	0.016547	-0.02456	0.412477	0.432156	FALSE	FALSE	FALSE	I9_PULMEM	0.106464	0.015212	pulmonary	TRUE	reported	textfile	6	1.37E+08	0.001881	1.48E-18	562239	white blc	TRUE	reported	igd	0.000138	77.38547	2	TRUE
rs6948695	C	T	C	T	-0.01099	0.002063	0.413883	0.391728	FALSE	FALSE	FALSE	I9_PULMEM	0.893465	0.015401	pulmonary	TRUE	reported	textfile	7	1.3E+08	0.001875	4.70E-09	561298	white blc	TRUE	reported	igd	6.12E-05	34.37386	2	TRUE
rs6952262	G	C	G	C	-0.01615	-0.05359	0.205159	0.180914	FALSE	TRUE	FALSE	I9_PULMEM	0.006425	0.019666	pulmonary	TRUE	reported	textfile	7	1.4E+08	0.002298	2.19E-12	560217	white blc	TRUE	reported	igd	8.82E-05	49.40274	2	TRUE
rs696	T	C	T	C	0.015003	-0.03477	0.36576	0.388934	FALSE	FALSE	FALSE	I9_PULMEM	0.02388	0.01539	pulmonary	TRUE	reported	textfile	14	35871093	0.001912	4.53E-15	561302	white blc	TRUE	reported	igd	0.00011	61.57138	2	TRUE
rs6985508	A	G	A	G	-0.02656	-0.00872	0.357998	0.369326	FALSE	FALSE	FALSE	I9_PULMEM	0.574115	0.015524	pulmonary	TRUE	reported	textfile	8	1.42E+08	0.002018	1.57E-39	503530	white blc	TRUE	reported	igd	0.000344	173.1995	2	TRUE
rs7005996	T	C	T	C	0.024146	-0.02985	0.906282	0.900881	FALSE	FALSE	FALSE	I9_PULMEM	0.237152	0.025253	pulmonary	TRUE	reported	textfile	8	1.42E+08	0.003326	4.06E-13	508418	white blc	TRUE	reported	igd	0.000104	52.70407	2	TRUE
rs703005	T	C	T	C	-0.01548	0.005669	0.598155	0.689299	FALSE	FALSE	FALSE	I9_PULMEM	0.72806	0.016305	pulmonary	TRUE	reported	textfile	10	26758576	0.001905	4.55E-16	561155	white blc	TRUE	reported	igd	0.000118	66.04845	2	TRUE
rs7036656	T	C	T	C	0.028141	-0.00536	0.7215	0.771704	FALSE	FALSE	FALSE	I9_PULMEM	0.763947	0.017842	pulmonary	TRUE	reported	textfile	9	21990457	0.002055	1.25E-42	560512	white blc	TRUE	reported	igd	0.000334	187.5227	2	TRUE
rs706809	C	T	C	T	0.022341	0.008932	0.777872	0.796839	FALSE	FALSE	FALSE	I9_PULMEM	0.633524	0.018735	pulmonary	TRUE	reported	textfile	12	52294257	0.002258	4.71E-23	561301	white blc	TRUE	reported	igd	0.000174	97.89394	2	TRUE
rs7082470	A	G	A	G	-0.02902	0.007834	0.474125	0.472409	FALSE	FALSE	FALSE	I9_PULMEM	0.60222	0.01503	pulmonary	TRUE	reported	textfile	10	65277026	0.00184	5.75E-56	562237	white blc	TRUE	reported	igd	0.000442	248.6954	2	TRUE
rs7135223	T	C	T	C	-0.01291	0.020879	0.323814	0.304681	FALSE	FALSE	FALSE	I9_PULMEM	0.20734	0.016559	pulmonary	TRUE	reported	textfile	19	45429543	0.002182	3.36E-09	507479	white blc	TRUE	reported	igd	6.90E-05	35.0059	2	TRUE
rs7159281	G	A	G	A	-0.01285	0.036537	0.671781	0.658716	FALSE	FALSE	FALSE	I9_PULMEM	0.021484	0.01589	pulmonary	TRUE	reported	textfile	14	23756604	0.002	1.35E-10	561156	white blc	TRUE	reported	igd	7.36E-05	41.28048	2	TRUE
rs7177	A	C	A	C	-0.01338	-0.00985	0.529439	0.571534	FALSE	FALSE	FALSE	I9_PULMEM	0.517223	0.015204	pulmonary	TRUE	reported	textfile	11	69466115	0.001888	1.43E-12	561295	white blc	TRUE	reported	igd	8.94E-05	50.19344	2	TRUE
rs7183988	G	T	G	T	-0.01578	-0.01491	0.526631	0.592467	FALSE	FALSE	FALSE	I9_PULMEM	0.328417	0.01526	pulmonary	TRUE	reported	textfile	15	91428589	0.001895	8.74E-17	561157	white blc	TRUE	reported	igd	0.000124	69.31526	2	TRUE

rs718515	A	G	A	G	-0.01639	-0.01343	0.551929	0.561605	FALSE	FALSE	FALSE	I9_PULMEN	0.375338	0.015144	pulmonary	TRUE	reported	textfile	18	43856297	0.001858	1.20E-18	562240	white blc	TRUE	reported	igd	0.000138	77.82479	2	TRUE
rs7198940	T	C	T	C	0.015619	0.024393	0.52238	0.56602	FALSE	FALSE	FALSE	I9_PULMEN	0.107298	0.015147	pulmonary	TRUE	reported	textfile	16	81605559	0.001851	3.33E-17	562241	white blc	TRUE	reported	igd	0.000127	71.20198	2	TRUE
rs7225843	C	T	C	T	-0.03151	0.020132	0.203083	0.242343	FALSE	FALSE	FALSE	I9_PULMEN	0.250579	0.017523	pulmonary	TRUE	reported	textfile	17	2001825	0.002297	8.58E-43	559082	white blc	TRUE	reported	igd	0.000336	188.1799	2	TRUE
rs7235882	T	C	T	C	0.017758	-0.03344	0.093753	0.069846	FALSE	FALSE	FALSE	I9_PULMEN	0.257111	0.029507	pulmonary	TRUE	reported	textfile	18	19684119	0.003188	2.60E-08	561300	white blc	TRUE	reported	igd	5.53E-05	31.02772	2	TRUE
rs7246841	C	T	C	T	0.022477	0.005201	0.731395	0.685579	FALSE	FALSE	FALSE	I9_PULMEN	0.749489	0.016288	pulmonary	TRUE	reported	textfile	19	1030431	0.002216	3.78E-24	502283	white blc	TRUE	reported	igd	0.000205	102.8811	2	TRUE
rs7266484	T	C	T	C	0.020614	-0.00855	0.179041	0.12859	FALSE	FALSE	FALSE	I9_PULMEN	0.702839	0.022418	pulmonary	TRUE	reported	textfile	14	35596323	0.002397	8.44E-18	562234	white blc	TRUE	reported	igd	0.000132	73.9583	2	TRUE
rs7272602	C	T	C	T	-0.04262	0.044881	0.111895	0.075539	FALSE	FALSE	FALSE	I9_PULMEN	0.114731	0.028455	pulmonary	TRUE	reported	textfile	15	42248826	0.002923	4.17E-48	561303	white blc	TRUE	reported	igd	0.000379	212.6022	2	TRUE
rs7274707	A	G	A	G	0.017932	-0.01143	0.31238	0.336992	FALSE	FALSE	FALSE	I9_PULMEN	0.471597	0.015882	pulmonary	TRUE	reported	textfile	9	91545621	0.002003	3.63E-19	562232	white blc	TRUE	reported	igd	0.000143	80.14824	2	TRUE
rs7278012	C	T	C	T	0.034599	-0.00502	0.131112	0.094528	FALSE	FALSE	FALSE	I9_PULMEN	0.843992	0.025509	pulmonary	TRUE	reported	textfile	2	24000145	0.002765	6.75E-36	541793	white blc	TRUE	reported	igd	0.000289	156.5796	2	TRUE
rs7279086	C	T	C	T	-0.02583	0.02938	0.306947	0.296648	FALSE	FALSE	FALSE	I9_PULMEN	0.073276	0.016403	pulmonary	TRUE	reported	textfile	10	44880260	0.002003	5.05E-38	562239	white blc	TRUE	reported	igd	0.000296	166.336	2	TRUE
rs7283662	T	C	T	C	-0.01417	-0.02295	0.249186	0.290466	FALSE	FALSE	FALSE	I9_PULMEN	0.165778	0.016562	pulmonary	TRUE	reported	textfile	10	1.14E+08	0.002131	3.02E-11	561151	white blc	TRUE	reported	igd	7.88E-05	44.21517	2	TRUE
rs7283930	T	C	T	C	-0.01171	0.006042	0.73874	0.809842	FALSE	FALSE	FALSE	I9_PULMEN	0.751583	0.019086	pulmonary	TRUE	reported	textfile	21	30461373	0.002102	2.57E-08	562234	white blc	TRUE	reported	igd	5.52E-05	31.05585	2	TRUE
rs7284359	A	C	A	C	0.024724	-0.01693	0.06259	0.048773	FALSE	FALSE	FALSE	I9_PULMEN	0.627438	0.034882	pulmonary	TRUE	reported	textfile	6	25866380	0.00384	1.25E-10	562241	white blc	TRUE	reported	igd	7.37E-05	41.45467	2	TRUE
rs7295172	A	C	A	C	-0.01783	0.033604	0.096253	0.08997	FALSE	FALSE	FALSE	I9_PULMEN	0.197324	0.026065	pulmonary	TRUE	reported	textfile	2	2.2E+08	0.003116	1.08E-08	557052	white blc	TRUE	reported	igd	5.88E-05	32.74208	2	TRUE
rs7297371	T	A	T	A	-0.03175	0.051523	0.067073	0.058737	FALSE	TRUE	FALSE	I9_PULMEN	0.107947	0.032052	pulmonary	TRUE	reported	textfile	18	74072245	0.003713	1.28E-17	561154	white blc	TRUE	reported	igd	0.00013	73.12458	2	TRUE
rs7297875	C	T	C	T	-0.02844	0.013826	0.065813	0.099397	FALSE	FALSE	FALSE	I9_PULMEN	0.582449	0.025147	pulmonary	TRUE	reported	textfile	6	1.38E+08	0.003727	2.45E-14	562240	white blc	TRUE	reported	igd	0.000104	58.23303	2	TRUE
rs7298298	A	G	A	G	-0.01277	0.037339	0.232903	0.167222	FALSE	FALSE	FALSE	I9_PULMEN	0.062476	0.020043	pulmonary	TRUE	reported	textfile	18	57802714	0.002204	7.10E-09	562237	white blc	TRUE	reported	igd	5.97E-05	33.57044	2	TRUE
rs7300096	A	T	A	T	0.028768	-0.01657	0.314788	0.375965	FALSE	TRUE	FALSE	I9_PULMEN	0.287892	0.015591	pulmonary	TRUE	reported	textfile	11	1.14E+08	0.001987	1.86E-47	562241	white blc	TRUE	reported	igd	0.000373	209.6149	2	TRUE
rs7302887	T	C	T	C	-0.01682	0.013516	0.133879	0.091961	FALSE	FALSE	FALSE	I9_PULMEN	0.606354	0.026231	pulmonary	TRUE	reported	textfile	3	14277058	0.002745	9.30E-10	544951	white blc	TRUE	reported	igd	6.89E-05	37.52832	2	TRUE
rs7303651	G	A	G	A	-0.05273	0.02717	0.258764	0.295022	FALSE	FALSE	FALSE	I9_PULMEN	0.098823	0.016461	pulmonary	TRUE	reported	textfile	19	45744842	0.00211	########	562226	white blc	TRUE	reported	igd	0.001109	624.4529	2	TRUE
rs7308123	C	T	C	T	0.018607	0.047833	0.18119	0.154396	FALSE	FALSE	FALSE	I9_PULMEN	0.021326	0.020777	pulmonary	TRUE	reported	textfile	12	1.22E+08	0.002436	2.29E-14	562241	white blc	TRUE	reported	igd	0.000104	58.34405	2	TRUE
rs7319067	A	G	A	G	0.028802	-0.03063	0.050278	0.032422	FALSE	FALSE	FALSE	I9_PULMEN	0.472152	0.0426	pulmonary	TRUE	reported	textfile	12	1.1E+08	0.004241	1.15E-11	562243	white blc	TRUE	reported	igd	8.20E-05	46.1219	2	TRUE
rs7319118	A	G	A	G	-0.01648	-0.02816	0.353128	0.445935	FALSE	FALSE	FALSE	I9_PULMEN	0.062968	0.015144	pulmonary	TRUE	reported	textfile	4	3105200	0.001926	1.23E-17	562226	white blc	TRUE	reported	igd	0.00013	73.18839	2	TRUE
rs7326825	A	G	A	G	0.020659	-0.00025	0.703613	0.640877	FALSE	FALSE	FALSE	I9_PULMEN	0.987159	0.015653	pulmonary	TRUE	reported	textfile	13	50113450	0.002021	1.73E-24	561300	white blc	TRUE	reported	igd	0.000186	104.4923	2	TRUE
rs7373852	G	A	G	A	0.022809	0.008142	0.080389	0.075426	FALSE	FALSE	FALSE	I9_PULMEN	0.774694	0.028446	pulmonary	TRUE	reported	textfile	6	45588963	0.003374	1.43E-11	562240	white blc	TRUE	reported	igd	8.13E-05	45.70048	2	TRUE
rs739842	C	T	C	T	-0.01555	0.021207	0.639495	0.662751	FALSE	FALSE	FALSE	I9_PULMEN	0.182775	0.015918	pulmonary	TRUE	reported	textfile	12	48202046	0.001973	3.44E-15	561300	white blc	TRUE	reported	igd	0.000111	62.08427	2	TRUE
rs7407632	C	T	C	T	-0.02866	-0.048	0.04472	0.019347	FALSE	FALSE	FALSE	I9_PULMEN	0.378813	0.054538	pulmonary	TRUE	reported	textfile	1	56979431	0.004475	1.55E-10	562240	white blc	TRUE	reported	igd	7.30E-05	41.02567	2	TRUE
rs7460784	A	G	A	G	-0.01541	-0.00291	0.625943	0.691255	FALSE	FALSE	FALSE	I9_PULMEN	0.858076	0.016263	pulmonary	TRUE	reported	textfile	7	1.49E+08	0.001914	8.82E-16	562236	white blc	TRUE	reported	igd	0.000115	64.77956	2	TRUE
rs7467983	G	C	G	C	0.012643	0.032185	0.277091	0.342502	FALSE	TRUE	FALSE	I9_PULMEN	0.043029	0.015906	pulmonary	TRUE	reported	textfile	6	86819356	0.002139	3.49E-09	562241	white blc	TRUE	reported	igd	6.21E-05	34.93632	2	TRUE
rs7535422	T	C	T	C	0.02445	-0.01316	0.128659	0.126987	FALSE	FALSE	FALSE	I9_PULMEN	0.559106	0.02253	pulmonary	TRUE	reported	textfile	18	42078782	0.002749	6.22E-19	562234	white blc	TRUE	reported	igd	0.000141	79.1055	2	TRUE
rs7547562	G	C	G	C	0.032813	0.003751	0.076568	0.088127	FALSE	TRUE	FALSE	I9_PULMEN	0.888162	0.026676	pulmonary	TRUE	reported	textfile	2	54787592	0.003538	1.92E-20	562242	white blc	TRUE	reported	igd	0.000153	86.0151	2	TRUE
rs7549164	T	C	T	C	0.025162	-0.01091	0.189126	0.286434	FALSE	FALSE	FALSE	I9_PULMEN	0.514156	0.016718	pulmonary	TRUE	reported	textfile	1	31224193	0.002456	1.33E-24	561299	white blc	TRUE	reported	igd	0.000187	104.962	2	TRUE
rs755951	C	A	C	A	-0.01347	0.014534	0.402765	0.363809	FALSE	FALSE	FALSE	I9_PULMEN	0.353781	0.015674	pulmonary	TRUE	reported	textfile	8	27226790	0.001888	1.02E-12	560510	white blc	TRUE	reported	igd	9.08E-05	50.87872	2	TRUE
rs7572278	A	T	A	T	0.015881	0.009696	0.207324	0.219939	FALSE	TRUE	FALSE	I9_PULMEN	0.595552	0.018266	pulmonary	TRUE	reported	textfile	2	8563029	0.002332	1.01E-11	561156	white blc	TRUE	reported	igd	8.26E-05	46.3764	2	TRUE
rs7573465	T	G	T	G	0.035796	-0.02998	0.554185	0.589725	FALSE	FALSE	FALSE	I9_PULMEN	0.049552	0.015265	pulmonary	TRUE	reported	textfile	2	1.82E+08	0.001853	4.66E-83	558139	white blc	TRUE	reported	igd	0.000668	373.1785	2	TRUE
rs759488	C	T	C	T	0.013512	0.037494	0.70489	0.619537	FALSE	FALSE	FALSE	I9_PULMEN	0.01513	0.015435	pulmonary	TRUE	reported	textfile	12	68587155	0.002025	2.58E-11	560512	white blc	TRUE	reported	igd	7.94E-05	44.52333	2	TRUE
rs7637816	A	G	A	G	0.023127	0.011229	0.061252	0.096247	FALSE	FALSE	FALSE	I9_PULMEN	0.661328	0.025633	pulmonary	TRUE	reported	textfile	19	18433714	0.004041	1.07E-08	508413	white blc	TRUE	reported	igd	6.44E-05	32.75361	2	TRUE
rs7639292	T	C	T	C	-0.02398	-0.00153	0.168472	0.118962	FALSE	FALSE	FALSE	I9_PULMEN	0.947378	0.023201	pulmonary	TRUE	reported	textfile	3	1.07E+08	0.002466	2.48E-22	562238	white blc	TRUE	reported	igd	0.000168	94.59225	2	TRUE
rs7642810	C	T	C	T	0.158445	-0.10983	0.013471	0.012756	FALSE	FALSE	FALSE	I9_PULMEN	0.108723	0.068471	pulmonary	TRUE	reported	textfile	13	28604007	0.008517	3.45E-77	562241	white blc	TRUE	reported	igd	0.000615	346.0846	2	TRUE
rs7654587	A	G	A	G	-0.01301	-0.01588	0.194057	0.187877	FALSE	FALSE	FALSE	I9_PULMEN	0.411118	0.019315	pulmonary	TRUE	reported	textfile	12	20432303	0.002333	2.50E-08	561301	white blc	TRUE	reported	igd	5.54E-05	31.11171	2	TRUE
rs7705526	A	C	A	C	0.029089	0.004212	0.327218	0.323917	FALSE	FALSE	FALSE	I9_PULMEN	0.793127	0.01606	pulmonary	TRUE	reported	textfile	5	1285974	0.00212	8.30E-43	490023	white blc	TRUE	reported	igd	0.000384	188.2713	2	TRUE
rs7721566	G	A	G	A	-0.04012	-0.05926	0.018274	0.003202	FALSE	FALSE	FALSE	I9_PULMEN	0.649534	0.130405	pulmonary	TRUE	reported	textfile	9	1.26E+08	0.006861	5.13E-09	562242	white blc	TRUE	reported	igd	6.08E-05	34.19365	2	TRUE
rs7751717	A	G	A	G	-0.0138	-0.00575	0.199529	0.274654	FALSE	FALSE	FALSE	I9_PULMEN	0.732762	0.016843	pulmonary	TRUE	reported	textfile	6	1.53E+08	0.002306	2.23E-09	562241	white blc	TRUE	reported	igd	6.37E-05	35.82316	2	TRUE
rs7776054	G	A	G	A	-0.04663	0.030199	0.261097	0.339957	FALSE	FALSE	FALSE	I9_PULMEN	0.057187	0.015878	pulmonary	TRUE	reported	textfile	6	1.35E+08	0.002089	########	561449	white blc	TRUE	reported	igd	0.000887	498.2558	2	TRUE

rs7776857	T	G	T	G	-0.01855	0.016337	0.65805	0.593381	FALSE	FALSE	FALSE	I9_PULMEM	0.285956	0.015311	pulmonary	TRUE	reported	textfile	7	22754768	0.001949	1.89E-21	562238	white blc	TRUE	reported	igd	0.000161	90.53749	2	TRUE
rs778125	G	A	G	A	0.01436	-0.01221	0.355448	0.29071	FALSE	FALSE	FALSE	I9_PULMEM	0.459169	0.016495	pulmonary	TRUE	reported	textfile	1	56605746	0.00192	7.75E-14	559079	white blc	TRUE	reported	igd	0.0001	55.93773	2	TRUE
rs7781268	A	G	A	G	-0.01333	-0.01969	0.245805	0.355821	FALSE	FALSE	FALSE	I9_PULMEM	0.210742	0.015732	pulmonary	TRUE	reported	textfile	7	75748183	0.002258	3.66E-09	507300	white blc	TRUE	reported	igd	6.87E-05	34.85064	2	TRUE
rs780142	G	T	G	T	0.014549	-0.0142	0.274035	0.253496	FALSE	FALSE	FALSE	I9_PULMEM	0.411821	0.017307	pulmonary	TRUE	reported	textfile	15	62797964	0.002067	2.02E-12	561156	white blc	TRUE	reported	igd	8.83E-05	49.54317	2	TRUE
rs7803075	G	A	G	A	-0.02444	-0.00957	0.7342	0.640978	FALSE	FALSE	FALSE	I9_PULMEM	0.542716	0.01573	pulmonary	TRUE	reported	textfile	7	1.31E+08	0.00211	5.47E-31	559426	white blc	TRUE	reported	igd	0.00024	134.164	2	TRUE
rs7828590	T	A	T	A	0.019812	-0.00136	0.089011	0.088396	FALSE	TRUE	FALSE	I9_PULMEM	0.959248	0.02669	pulmonary	TRUE	reported	textfile	18	21622003	0.003341	3.10E-09	562243	white blc	TRUE	reported	igd	6.25E-05	35.16431	2	TRUE
rs7846314	T	A	T	A	0.046831	-0.00159	0.187345	0.194016	FALSE	TRUE	FALSE	I9_PULMEM	0.933401	0.01898	pulmonary	TRUE	reported	textfile	8	61650831	0.002355	5.71E-88	562243	white blc	TRUE	reported	igd	0.000703	395.4426	2	TRUE
rs7850247	C	T	C	T	0.01034	-0.0252	0.514717	0.460201	FALSE	FALSE	FALSE	I9_PULMEM	0.093955	0.015046	pulmonary	TRUE	reported	textfile	9	95747389	0.001861	2.84E-08	560500	white blc	TRUE	reported	igd	5.51E-05	30.87072	2	TRUE
rs7852409	G	C	G	C	0.015628	-0.02939	0.764716	0.729015	FALSE	TRUE	FALSE	I9_PULMEM	0.086596	0.01715	pulmonary	TRUE	reported	textfile	9	2621482	0.002288	8.71E-12	560215	white blc	TRUE	reported	igd	8.33E-05	46.65447	2	TRUE
rs7855091	A	G	A	G	0.014823	-0.01007	0.307022	0.350037	FALSE	FALSE	FALSE	I9_PULMEM	0.522866	0.015754	pulmonary	TRUE	reported	textfile	9	1.15E+08	0.001994	1.11E-13	559081	white blc	TRUE	reported	igd	9.88E-05	55.26121	2	TRUE
rs7864482	G	A	G	A	0.012766	0.004047	0.377314	0.391721	FALSE	FALSE	FALSE	I9_PULMEM	0.792204	0.015361	pulmonary	TRUE	reported	textfile	9	1.02E+08	0.001902	2.00E-11	562241	white blc	TRUE	reported	igd	8.01E-05	45.0492	2	TRUE
rs7917772	A	G	A	G	0.016558	-0.00095	0.630809	0.584067	FALSE	FALSE	FALSE	I9_PULMEM	0.950511	0.015264	pulmonary	TRUE	reported	textfile	10	1.04E+08	0.001904	3.60E-18	561300	white blc	TRUE	reported	igd	0.000135	75.6276	2	TRUE
rs7923752	T	C	T	C	0.040077	0.118862	0.022751	0.020131	FALSE	FALSE	FALSE	I9_PULMEM	0.026917	0.053718	pulmonary	TRUE	reported	textfile	5	1.35E+08	0.006305	2.13E-10	559444	white blc	TRUE	reported	igd	7.22E-05	40.40347	2	TRUE
rs7927292	A	C	A	C	-0.02232	0.001941	0.221625	0.251419	FALSE	FALSE	FALSE	I9_PULMEM	0.910626	0.017288	pulmonary	TRUE	reported	textfile	5	1.19E+08	0.002222	1.04E-23	560821	white blc	TRUE	reported	igd	0.00018	100.8656	2	TRUE
rs7934719	T	C	T	C	0.017862	-0.01839	0.414299	0.396588	FALSE	FALSE	FALSE	I9_PULMEM	0.2297	0.015307	pulmonary	TRUE	reported	textfile	11	1.08E+08	0.001873	1.55E-21	562226	white blc	TRUE	reported	igd	0.000162	90.94589	2	TRUE
rs796003	T	G	T	G	-0.02535	0.018484	0.258407	0.25813	FALSE	FALSE	FALSE	I9_PULMEM	0.280912	0.017142	pulmonary	TRUE	reported	textfile	9	86595801	0.002125	8.84E-33	562238	white blc	TRUE	reported	igd	0.000253	142.3328	2	TRUE
rs796056	C	A	C	A	0.020577	-0.00791	0.623451	0.689541	FALSE	FALSE	FALSE	I9_PULMEM	0.625561	0.016203	pulmonary	TRUE	reported	textfile	2	1.02E+08	0.001898	2.31E-27	558143	white blc	TRUE	reported	igd	0.000211	117.5358	2	TRUE
rs7969238	C	G	C	G	0.02617	0.029602	0.061217	0.066336	FALSE	TRUE	FALSE	I9_PULMEM	0.328617	0.030302	pulmonary	TRUE	reported	textfile	5	1.11E+08	0.003837	9.39E-12	561302	white blc	TRUE	reported	igd	8.29E-05	46.51814	2	TRUE
rs7971658	A	G	A	G	-0.03586	0.036537	0.124095	0.084122	FALSE	FALSE	FALSE	I9_PULMEM	0.177452	0.027091	pulmonary	TRUE	reported	textfile	2	1.44E+08	0.00283	9.22E-37	558142	white blc	TRUE	reported	igd	0.000288	160.5901	2	TRUE
rs798555	C	T	C	T	0.018842	-0.03937	0.294964	0.361414	FALSE	FALSE	FALSE	I9_PULMEM	0.011897	0.015652	pulmonary	TRUE	reported	textfile	7	2759473	0.002054	4.81E-20	555158	white blc	TRUE	reported	igd	0.000152	84.1495	2	TRUE
rs806321	T	C	T	C	0.01598	-0.02322	0.530051	0.579208	FALSE	FALSE	FALSE	I9_PULMEM	0.126879	0.01521	pulmonary	TRUE	reported	textfile	13	50841323	0.001848	5.66E-18	560512	white blc	TRUE	reported	igd	0.000133	74.77356	2	TRUE
rs8084255	T	C	T	C	0.019947	0.008345	0.375635	0.2893	FALSE	FALSE	FALSE	I9_PULMEM	0.613766	0.016534	pulmonary	TRUE	reported	textfile	18	48144754	0.001905	1.26E-25	562237	white blc	TRUE	reported	igd	0.000195	109.6386	2	TRUE
rs8176528	A	G	A	G	-0.01687	-0.00667	0.297384	0.310948	FALSE	FALSE	FALSE	I9_PULMEM	0.680129	0.016181	pulmonary	TRUE	reported	textfile	2	1.88E+08	0.002017	6.31E-17	557056	white blc	TRUE	reported	igd	0.000126	69.97127	2	TRUE
rs830623	C	A	C	A	0.014483	0.007342	0.169687	0.165868	FALSE	FALSE	FALSE	I9_PULMEM	0.715595	0.020151	pulmonary	TRUE	reported	textfile	3	71677746	0.002457	3.88E-09	562242	white blc	TRUE	reported	igd	6.18E-05	34.74603	2	TRUE
rs832190	T	C	T	C	-0.01326	0.008943	0.633275	0.639854	FALSE	FALSE	FALSE	I9_PULMEM	0.566041	0.015583	pulmonary	TRUE	reported	textfile	3	63842629	0.001909	3.88E-12	561154	white blc	TRUE	reported	igd	8.60E-05	48.27648	2	TRUE
rs8705	A	G	A	G	-0.0264	0.025523	0.316569	0.435593	FALSE	FALSE	FALSE	I9_PULMEM	0.091207	0.01511	pulmonary	TRUE	reported	textfile	11	1.28E+08	0.002004	1.35E-39	559259	white blc	TRUE	reported	igd	0.00031	173.5051	2	TRUE
rs873218	A	G	A	G	-0.01192	-0.00961	0.553941	0.614024	FALSE	FALSE	FALSE	I9_PULMEM	0.533775	0.015446	pulmonary	TRUE	reported	textfile	12	12765603	0.001855	1.34E-10	560514	white blc	TRUE	reported	igd	7.37E-05	41.31945	2	TRUE
rs903123	T	C	T	C	0.013484	0.007303	0.818174	0.756843	FALSE	FALSE	FALSE	I9_PULMEM	0.677038	0.017535	pulmonary	TRUE	reported	textfile	1	2.12E+08	0.002387	1.67E-08	562242	white blc	TRUE	reported	igd	5.68E-05	31.91032	2	TRUE
rs921313	A	G	A	G	0.02711	0.013185	0.044853	0.040586	FALSE	FALSE	FALSE	I9_PULMEM	0.730545	0.038285	pulmonary	TRUE	reported	textfile	8	1.01E+08	0.004466	1.32E-09	560513	white blc	TRUE	reported	igd	6.57E-05	36.84852	2	TRUE
rs9313822	A	G	A	G	0.025101	-0.0446	0.052082	0.041394	FALSE	FALSE	FALSE	I9_PULMEM	0.234576	0.03752	pulmonary	TRUE	reported	textfile	5	1.59E+08	0.004098	9.35E-10	559082	white blc	TRUE	reported	igd	6.71E-05	37.51773	2	TRUE
rs9323285	A	C	A	C	0.012057	0.005546	0.337997	0.448806	FALSE	FALSE	FALSE	I9_PULMEM	0.713885	0.015125	pulmonary	TRUE	reported	textfile	14	55863829	0.001962	8.19E-10	557353	white blc	TRUE	reported	igd	6.78E-05	37.76408	2	TRUE
rs9330650	G	A	G	A	0.014403	-0.01194	0.21919	0.144095	FALSE	FALSE	FALSE	I9_PULMEM	0.580938	0.021637	pulmonary	TRUE	reported	textfile	12	1.24E+08	0.002256	1.77E-10	562238	white blc	TRUE	reported	igd	7.25E-05	40.75925	2	TRUE
rs9348970	C	G	C	G	-0.03276	-0.02745	0.178025	0.139748	FALSE	TRUE	FALSE	I9_PULMEM	0.207017	0.021753	pulmonary	TRUE	reported	textfile	6	35153565	0.002404	3.09E-42	562239	white blc	TRUE	reported	igd	0.00033	185.691	2	TRUE
rs935655	G	T	G	T	0.014783	-0.00623	0.761993	0.686397	FALSE	FALSE	FALSE	I9_PULMEM	0.700303	0.016182	pulmonary	TRUE	reported	textfile	2	46067445	0.002161	8.11E-12	562243	white blc	TRUE	reported	igd	8.32E-05	46.79658	2	TRUE
rs9375447	G	A	G	A	-0.01377	0.018439	0.494601	0.474836	FALSE	FALSE	FALSE	I9_PULMEM	0.218799	0.014994	pulmonary	TRUE	reported	textfile	6	1.27E+08	0.001834	6.34E-14	562238	white blc	TRUE	reported	igd	0.0001	56.34799	2	TRUE
rs9382100	A	T	A	T	-0.01196	-0.02504	0.324967	0.372176	FALSE	TRUE	FALSE	I9_PULMEM	0.107744	0.015566	pulmonary	TRUE	reported	textfile	6	52223211	0.001973	1.38E-09	562241	white blc	TRUE	reported	igd	6.54E-05	36.76415	2	TRUE
rs9390461	G	A	G	A	0.015366	0.035402	0.538265	0.485128	FALSE	FALSE	FALSE	I9_PULMEM	0.018782	0.015066	pulmonary	TRUE	reported	textfile	6	1.48E+08	0.001845	8.51E-17	562240	white blc	TRUE	reported	igd	0.000123	69.36292	2	TRUE
rs9429767	A	G	A	G	0.017446	0.001621	0.197243	0.231328	FALSE	FALSE	FALSE	I9_PULMEM	0.927712	0.01787	pulmonary	TRUE	reported	textfile	1	1.1E+08	0.002344	1.03E-13	561289	white blc	TRUE	reported	igd	9.87E-05	55.39556	2	TRUE
rs9430574	A	G	A	G	-0.01404	-0.0025	0.33041	0.417591	FALSE	FALSE	FALSE	I9_PULMEM	0.869364	0.015229	pulmonary	TRUE	reported	textfile	1	9709072	0.001984	1.55E-12	562233	white blc	TRUE	reported	igd	8.91E-05	50.07114	2	TRUE
rs9487043	T	C	T	C	0.023076	0.001503	0.486515	0.585462	FALSE	FALSE	FALSE	I9_PULMEM	0.921595	0.015274	pulmonary	TRUE	reported	textfile	6	1.1E+08	0.001839	4.35E-36	562232	white blc	TRUE	reported	igd	0.00028	157.4549	2	TRUE
rs9508005	G	T	G	T	-0.02253	0.020874	0.094159	0.061801	FALSE	FALSE	FALSE	I9_PULMEM	0.505701	0.031364	pulmonary	TRUE	reported	textfile	13	28789794	0.003425	4.92E-11	555496	white blc	TRUE	reported	igd	7.79E-05	43.27511	2	TRUE
rs9526795	C	T	C	T	-0.01411	-0.01224	0.258932	0.272917	FALSE	FALSE	FALSE	I9_PULMEM	0.46681	0.016817	pulmonary	TRUE	reported	textfile	13	52341181	0.002116	2.68E-11	557352	white blc	TRUE	reported	igd	7.98E-05	44.46529	2	TRUE
rs9625746	C	G	C	G	-0.01838	-0.04658	0.41186	0.305821	FALSE	TRUE	FALSE	I9_PULMEM	0.004447	0.016374	pulmonary	TRUE	reported	textfile	22	29637658	0.001915	8.77E-22	562221	white blc	TRUE	reported	igd	0.000164	92.0895	2	TRUE
rs9656395	G	A	G	A	-0.02752	-0.01172	0.095026	0.074239	FALSE	FALSE	FALSE	I9_PULMEM	0.682438	0.028656	pulmonary	TRUE	reported	textfile	7	1.31E+08	0.003156	2.97E-18	562239	white blc	TRUE	reported	igd	0.000135	76.00873	2	TRUE

rs9819371	T	C	T	C	-0.04445	-0.01507	0.064757	0.051937	FALSE	FALSE	FALSE	I9_PULMEM	0.657712	0.034002	pulmonary	TRUE	reported	textfile	3	1.41E+08	0.003776	5.94E-32	562240	white blc	TRUE	reported	igd	0.000246	138.5603	2	TRUE
rs9829114	A	G	A	G	-0.03358	0.01314	0.414149	0.432732	FALSE	FALSE	FALSE	I9_PULMEM	0.386497	0.015173	pulmonary	TRUE	reported	textfile	3	1.97E+08	0.001872	7.45E-72	562216	white blc	TRUE	reported	igd	0.000572	321.7336	2	TRUE
rs9835104	A	G	A	G	-0.01352	-0.02112	0.18798	0.195829	FALSE	FALSE	FALSE	I9_PULMEM	0.266236	0.019	pulmonary	TRUE	reported	textfile	3	1.19E+08	0.002467	4.35E-08	506686	white blc	TRUE	reported	igd	5.93E-05	30.02513	2	TRUE
rs9863	C	T	C	T	-0.01875	0.02812	0.329292	0.308029	FALSE	FALSE	FALSE	I9_PULMEM	0.08445	0.016297	pulmonary	TRUE	reported	textfile	12	1.24E+08	0.001965	1.53E-21	560508	white blc	TRUE	reported	igd	0.000162	91.03944	2	TRUE
rs9867398	T	C	T	C	0.022319	-0.01116	0.094175	0.106557	FALSE	FALSE	FALSE	I9_PULMEM	0.648896	0.024513	pulmonary	TRUE	reported	textfile	3	1.86E+08	0.003192	2.80E-12	562235	white blc	TRUE	reported	igd	8.69E-05	48.89024	2	TRUE
rs987107	A	G	A	G	0.020353	-0.02821	0.261079	0.294046	FALSE	FALSE	FALSE	I9_PULMEM	0.086119	0.016436	pulmonary	TRUE	reported	textfile	5	35875227	0.002093	2.52E-22	559079	white blc	TRUE	reported	igd	0.000169	94.56204	2	TRUE
rs9885207	C	T	C	T	0.016744	-0.02548	0.41613	0.418843	FALSE	FALSE	FALSE	I9_PULMEM	0.096206	0.015319	pulmonary	TRUE	reported	textfile	5	1.73E+08	0.001872	3.94E-19	562240	white blc	TRUE	reported	igd	0.000142	80.0028	2	TRUE
rs9900613	T	C	T	C	-0.01134	0.043133	0.43077	0.383018	FALSE	FALSE	FALSE	I9_PULMEM	0.004912	0.015335	pulmonary	TRUE	reported	textfile	17	74674857	0.001867	1.30E-09	561293	white blc	TRUE	reported	igd	6.57E-05	36.8793	2	TRUE
rs990558	T	C	T	C	-0.01106	0.004986	0.36139	0.407286	FALSE	FALSE	FALSE	I9_PULMEM	0.74425	0.015284	pulmonary	TRUE	reported	textfile	21	36914669	0.001929	1.00E-08	562243	white blc	TRUE	reported	igd	5.85E-05	32.88526	2	TRUE
rs9925985	C	A	C	A	-0.01413	0.001689	0.269481	0.254875	FALSE	FALSE	FALSE	I9_PULMEM	0.92182	0.017206	pulmonary	TRUE	reported	textfile	16	31305593	0.002078	1.09E-11	561297	white blc	TRUE	reported	igd	8.24E-05	46.24376	2	TRUE
rs9926183	T	C	T	C	0.018579	-0.00841	0.26273	0.229224	FALSE	FALSE	FALSE	I9_PULMEM	0.637535	0.017859	pulmonary	TRUE	reported	textfile	16	1363878	0.002197	2.86E-17	507332	white blc	TRUE	reported	igd	0.000141	71.51265	2	TRUE
rs9938104	T	C	T	C	-0.01662	-0.05251	0.12614	0.09793	FALSE	FALSE	FALSE	I9_PULMEM	0.037251	0.025211	pulmonary	TRUE	reported	textfile	16	81844974	0.002807	3.25E-09	562238	white blc	TRUE	reported	igd	6.24E-05	35.07395	2	TRUE
rs9977672	A	G	A	G	-0.02187	-0.02718	0.259116	0.237007	FALSE	FALSE	FALSE	I9_PULMEM	0.12502	0.017718	pulmonary	TRUE	reported	textfile	21	40463283	0.002122	7.08E-25	562242	white blc	TRUE	reported	igd	0.000189	106.2392	2	TRUE

Supplementary Table 8: Sensitivity analyses for the SVMR analysis between circulating white blood cell count and PE

	MR Egger results			Q-test for heterogeneity			MR-PRESSO results			
exposure	egger_intercept	se	pval	Q	Q_df	Q_pval	Causal Estimate	Sd	T-stat	P-value
basophil cell count	0.002132187	0.002919024	0.466047025	265.2118336	185	0.00010015	-0.079796309	0.063438895	-1.257845192	0.21003311
white blood cell count	-0.000331016	0.001996325	0.868379191	583.2851194	449	1.89E-05	-0.132265795	0.041160666	-3.213402674	0.001406183
monocyte cell count	0.002775534	0.001541053	0.072343476	578.7345148	464	0.000219329	-0.015313363	0.031522315	-0.485794355	0.627342297
lymphocyte cell count	0.003289555	0.001789065	0.066598144	504.574046	465	0.099516955	-0.099904019	0.036157129	-2.763051756	0.005953466
eosinophil cell count	-0.001395708	0.002135568	0.513755432	601.835328	423	2.25E-08	-0.021726398	0.039926372	-0.544161581	0.586616998
neutrophil cell count	-0.001313872	0.002071873	0.526358557	483.6187268	389	0.000751492	-0.12760534	0.043831546	-2.911267163	0.003806868

Supplementary Table 9: Leave-one-out analysis results for SVMR analysis of circulating white blood cell count and PE

Blood Cell	id.exposure	outcome	SNP	b	se	p
basophil	ieu-b-29	pulmonary embolism	rs10006833	-0.083315219	0.063412503	0.188892959
basophil	ieu-b-29	pulmonary embolism	rs10734121	-0.080345244	0.063751551	0.207565891
basophil	ieu-b-29	pulmonary embolism	rs10746147	-0.071952812	0.062647029	0.250744435
basophil	ieu-b-29	pulmonary embolism	rs10806232	-0.076842232	0.063548851	0.226592291
basophil	ieu-b-29	pulmonary embolism	rs10835333	-0.079539454	0.063689944	0.211718402
basophil	ieu-b-29	pulmonary embolism	rs10844657	-0.080809169	0.063641214	0.204169565
basophil	ieu-b-29	pulmonary embolism	rs1086893	-0.083605045	0.063770651	0.189848643
basophil	ieu-b-29	pulmonary embolism	rs10883359	-0.079719032	0.063676189	0.210590216
basophil	ieu-b-29	pulmonary embolism	rs10906375	-0.079497178	0.063670428	0.211821289
basophil	ieu-b-29	pulmonary embolism	rs10927074	-0.09129035	0.064176767	0.154886131
basophil	ieu-b-29	pulmonary embolism	rs11064881	-0.079532058	0.063739592	0.212116984
basophil	ieu-b-29	pulmonary embolism	rs11097787	-0.085575392	0.06313025	0.175246516
basophil	ieu-b-29	pulmonary embolism	rs11121246	-0.081059689	0.063642156	0.20277714
basophil	ieu-b-29	pulmonary embolism	rs11158159	-0.086582125	0.063521568	0.172871457
basophil	ieu-b-29	pulmonary embolism	rs11187969	-0.080197076	0.063637198	0.207588839
basophil	ieu-b-29	pulmonary embolism	rs112352373	-0.078789821	0.06364927	0.215762524
basophil	ieu-b-29	pulmonary embolism	rs1128099	-0.079883539	0.063674729	0.209639841
basophil	ieu-b-29	pulmonary embolism	rs11633707	-0.081329211	0.063617303	0.201103711
basophil	ieu-b-29	pulmonary embolism	rs11710737	-0.080532756	0.063648856	0.205775862
basophil	ieu-b-29	pulmonary embolism	rs11772895	-0.078613158	0.064019713	0.219464856
basophil	ieu-b-29	pulmonary embolism	rs118013485	-0.074808229	0.063258211	0.236973551
basophil	ieu-b-29	pulmonary embolism	rs1186222	-0.082804545	0.063715227	0.193736833
basophil	ieu-b-29	pulmonary embolism	rs12075	-0.078665568	0.063909901	0.218366875
basophil	ieu-b-29	pulmonary embolism	rs12123922	-0.086578548	0.063700596	0.174099645
basophil	ieu-b-29	pulmonary embolism	rs12143614	-0.075514572	0.063365281	0.233365484
basophil	ieu-b-29	pulmonary embolism	rs12376511	-0.086482935	0.063122862	0.170664084
basophil	ieu-b-29	pulmonary embolism	rs12443468	-0.080403977	0.063650523	0.206513726
basophil	ieu-b-29	pulmonary embolism	rs12453682	-0.083429802	0.063553209	0.189265472
basophil	ieu-b-29	pulmonary embolism	rs12459419	-0.08253715	0.063612243	0.194457777
basophil	ieu-b-29	pulmonary embolism	rs12497690	-0.082999422	0.063510359	0.191258797
basophil	ieu-b-29	pulmonary embolism	rs12921873	-0.083007621	0.0634035	0.1904679
basophil	ieu-b-29	pulmonary embolism	rs12941811	-0.070422789	0.064329803	0.273641591
basophil	ieu-b-29	pulmonary embolism	rs1295927	-0.077443935	0.063827984	0.225006509
basophil	ieu-b-29	pulmonary embolism	rs13089722	-0.097842391	0.06426004	0.127858599
basophil	ieu-b-29	pulmonary embolism	rs13188960	-0.07654988	0.06362876	0.228949065
basophil	ieu-b-29	pulmonary embolism	rs13267723	-0.079816984	0.063660477	0.209917691
basophil	ieu-b-29	pulmonary embolism	rs13386606	-0.083326251	0.06335974	0.188466448
basophil	ieu-b-29	pulmonary embolism	rs13419763	-0.084728667	0.063345817	0.181040755
basophil	ieu-b-29	pulmonary embolism	rs138595256	-0.081974784	0.063963866	0.199990172
basophil	ieu-b-29	pulmonary embolism	rs142405270	-0.080413746	0.063600805	0.206104046
basophil	ieu-b-29	pulmonary embolism	rs1427499	-0.083864688	0.063609756	0.187361442

basophil	ieu-b-29	pulmonary embolism	rs146970669	-0.081304907	0.063656617	0.201516808
basophil	ieu-b-29	pulmonary embolism	rs149007767	-0.076603297	0.063717037	0.229269785
basophil	ieu-b-29	pulmonary embolism	rs1537061	-0.077621899	0.063652143	0.222665734
basophil	ieu-b-29	pulmonary embolism	rs1539174	-0.07792241	0.064165435	0.224595573
basophil	ieu-b-29	pulmonary embolism	rs1598207	-0.090110772	0.062762867	0.151078092
basophil	ieu-b-29	pulmonary embolism	rs1633768	-0.078242599	0.063642244	0.218917016
basophil	ieu-b-29	pulmonary embolism	rs1669340	-0.081007833	0.063885649	0.204793311
basophil	ieu-b-29	pulmonary embolism	rs16923637	-0.075417734	0.063320741	0.233636651
basophil	ieu-b-29	pulmonary embolism	rs16928084	-0.077619717	0.0637267	0.223220416
basophil	ieu-b-29	pulmonary embolism	rs16989483	-0.070498548	0.062857682	0.262050338
basophil	ieu-b-29	pulmonary embolism	rs17613339	-0.076409206	0.063394237	0.228086709
basophil	ieu-b-29	pulmonary embolism	rs17625587	-0.080496832	0.063674954	0.206164144
basophil	ieu-b-29	pulmonary embolism	rs17758695	-0.066133703	0.064425294	0.304647637
basophil	ieu-b-29	pulmonary embolism	rs17860282	-0.067630144	0.064324913	0.293082548
basophil	ieu-b-29	pulmonary embolism	rs1804999	-0.079274902	0.063652984	0.212976236
basophil	ieu-b-29	pulmonary embolism	rs182090955	-0.080150075	0.063641763	0.207887939
basophil	ieu-b-29	pulmonary embolism	rs2028900	-0.083318231	0.063457219	0.189188777
basophil	ieu-b-29	pulmonary embolism	rs2070596	-0.084925587	0.063278332	0.179564921
basophil	ieu-b-29	pulmonary embolism	rs2074585	-0.078062296	0.063979315	0.222420251
basophil	ieu-b-29	pulmonary embolism	rs2089979	-0.077148143	0.063560567	0.224834161
basophil	ieu-b-29	pulmonary embolism	rs2118140	-0.084339084	0.063523523	0.184282945
basophil	ieu-b-29	pulmonary embolism	rs2229092	-0.082164448	0.063521337	0.195840369
basophil	ieu-b-29	pulmonary embolism	rs2239635	-0.076460657	0.064021546	0.232362326
basophil	ieu-b-29	pulmonary embolism	rs2271352	-0.076986031	0.064088022	0.229652453
basophil	ieu-b-29	pulmonary embolism	rs2273770	-0.081228783	0.06359883	0.201529664
basophil	ieu-b-29	pulmonary embolism	rs2276066	-0.075834141	0.063315421	0.231026075
basophil	ieu-b-29	pulmonary embolism	rs2282986	-0.085869683	0.063774424	0.178154352
basophil	ieu-b-29	pulmonary embolism	rs2286599	-0.074692329	0.063216374	0.237390441
basophil	ieu-b-29	pulmonary embolism	rs2289511	-0.079582778	0.063715198	0.211650743
basophil	ieu-b-29	pulmonary embolism	rs2294199	-0.078723431	0.063701278	0.216524795
basophil	ieu-b-29	pulmonary embolism	rs2298989	-0.079967361	0.063660634	0.209061271
basophil	ieu-b-29	pulmonary embolism	rs2524079	-0.065861342	0.06326301	0.297842178
basophil	ieu-b-29	pulmonary embolism	rs2594836	-0.075426696	0.063448997	0.234527508
basophil	ieu-b-29	pulmonary embolism	rs2606724	-0.081544155	0.063666167	0.200261007
basophil	ieu-b-29	pulmonary embolism	rs2607278	-0.082109006	0.063762379	0.197838451
basophil	ieu-b-29	pulmonary embolism	rs2738104	-0.07978427	0.063741354	0.210683675
basophil	ieu-b-29	pulmonary embolism	rs2805921	-0.084920912	0.063076589	0.178201184
basophil	ieu-b-29	pulmonary embolism	rs2811708	-0.080443947	0.063685808	0.206539691
basophil	ieu-b-29	pulmonary embolism	rs2834670	-0.074331067	0.063563356	0.242242012
basophil	ieu-b-29	pulmonary embolism	rs2836241	-0.075640292	0.063376593	0.232671582
basophil	ieu-b-29	pulmonary embolism	rs28364390	-0.084684024	0.063246314	0.180585036
basophil	ieu-b-29	pulmonary embolism	rs2860773	-0.079143353	0.063736564	0.214337654
basophil	ieu-b-29	pulmonary embolism	rs28647824	-0.078774543	0.063702657	0.216236569
basophil	ieu-b-29	pulmonary embolism	rs2959356	-0.07685126	0.063507497	0.226235457

basophil	ieu-b-29	pulmonary embolism	rs2967595	-0.075905074	0.063598882	0.232674794
basophil	ieu-b-29	pulmonary embolism	rs2977799	-0.085912845	0.063739795	0.177700768
basophil	ieu-b-29	pulmonary embolism	rs2998286	-0.072603993	0.063084565	0.249773591
basophil	ieu-b-29	pulmonary embolism	rs3071	-0.074807473	0.063381394	0.237890867
basophil	ieu-b-29	pulmonary embolism	rs310631	-0.079417773	0.063659841	0.21220201
basophil	ieu-b-29	pulmonary embolism	rs3181077	-0.073225757	0.06388484	0.251706236
basophil	ieu-b-29	pulmonary embolism	rs3184504	-0.090401461	0.06341924	0.154024862
basophil	ieu-b-29	pulmonary embolism	rs34097845	-0.077909368	0.063827768	0.222230418
basophil	ieu-b-29	pulmonary embolism	rs34158728	-0.080074672	0.064468547	0.214209455
basophil	ieu-b-29	pulmonary embolism	rs34288539	-0.081300127	0.063650158	0.201497574
basophil	ieu-b-29	pulmonary embolism	rs34377578	-0.078769419	0.063623362	0.215694518
basophil	ieu-b-29	pulmonary embolism	rs34500	-0.087417401	0.062791885	0.163868886
basophil	ieu-b-29	pulmonary embolism	rs34638654	-0.081918848	0.063555853	0.197423499
basophil	ieu-b-29	pulmonary embolism	rs34780507	-0.079046971	0.063675537	0.214457097
basophil	ieu-b-29	pulmonary embolism	rs34850939	-0.077811579	0.063594953	0.221122187
basophil	ieu-b-29	pulmonary embolism	rs35045014	-0.080622112	0.063642649	0.205229036
basophil	ieu-b-29	pulmonary embolism	rs357613	-0.078557957	0.063594905	0.216723725
basophil	ieu-b-29	pulmonary embolism	rs3731332	-0.076770727	0.063874913	0.229405494
basophil	ieu-b-29	pulmonary embolism	rs377763	-0.087400526	0.063318145	0.16748219
basophil	ieu-b-29	pulmonary embolism	rs3781452	-0.076287795	0.063657419	0.230756789
basophil	ieu-b-29	pulmonary embolism	rs3892360	-0.089847365	0.064102417	0.161028121
basophil	ieu-b-29	pulmonary embolism	rs4060971	-0.083657711	0.063653874	0.188758875
basophil	ieu-b-29	pulmonary embolism	rs42030	-0.079980939	0.063723999	0.209437059
basophil	ieu-b-29	pulmonary embolism	rs4324460	-0.077832017	0.06366689	0.221523091
basophil	ieu-b-29	pulmonary embolism	rs4475963	-0.07482062	0.063189959	0.236389832
basophil	ieu-b-29	pulmonary embolism	rs45577137	-0.084066749	0.063706439	0.18697001
basophil	ieu-b-29	pulmonary embolism	rs4602187	-0.084237953	0.063261771	0.182999053
basophil	ieu-b-29	pulmonary embolism	rs4705908	-0.083034496	0.063337021	0.189859567
basophil	ieu-b-29	pulmonary embolism	rs4763817	-0.079475205	0.063662894	0.21189353
basophil	ieu-b-29	pulmonary embolism	rs4876400	-0.076383352	0.063569044	0.229525925
basophil	ieu-b-29	pulmonary embolism	rs4911102	-0.081919399	0.063732184	0.198663227
basophil	ieu-b-29	pulmonary embolism	rs4912807	-0.077570432	0.063557208	0.222282186
basophil	ieu-b-29	pulmonary embolism	rs4941839	-0.080140634	0.063659464	0.208067962
basophil	ieu-b-29	pulmonary embolism	rs55690609	-0.085555981	0.063924464	0.18076874
basophil	ieu-b-29	pulmonary embolism	rs56043070	-0.07795644	0.063582863	0.220175891
basophil	ieu-b-29	pulmonary embolism	rs561102	-0.084724796	0.06320655	0.18010127
basophil	ieu-b-29	pulmonary embolism	rs56179563	-0.07761507	0.063604057	0.222356891
basophil	ieu-b-29	pulmonary embolism	rs56388170	-0.076845945	0.063890447	0.229062718
basophil	ieu-b-29	pulmonary embolism	rs56406125	-0.083071627	0.063930931	0.193807894
basophil	ieu-b-29	pulmonary embolism	rs5750494	-0.080990098	0.063647445	0.203202644
basophil	ieu-b-29	pulmonary embolism	rs59107033	-0.087012397	0.062451963	0.163538397
basophil	ieu-b-29	pulmonary embolism	rs602229	-0.080052686	0.063662371	0.208588248
basophil	ieu-b-29	pulmonary embolism	rs6029234	-0.08284816	0.063460868	0.191722663
basophil	ieu-b-29	pulmonary embolism	rs6045612	-0.079747778	0.063696667	0.210572433

basophil	ieu-b-29	pulmonary embolism	rs6091176	-0.081201175	0.063614251	0.201792245
basophil	ieu-b-29	pulmonary embolism	rs6141781	-0.073315155	0.063611444	0.249096527
basophil	ieu-b-29	pulmonary embolism	rs62021606	-0.079061203	0.063700114	0.214551466
basophil	ieu-b-29	pulmonary embolism	rs62105478	-0.078157903	0.06347098	0.218174837
basophil	ieu-b-29	pulmonary embolism	rs62160676	-0.081797401	0.063644493	0.198714175
basophil	ieu-b-29	pulmonary embolism	rs6421984	-0.073003179	0.063259119	0.2484862
basophil	ieu-b-29	pulmonary embolism	rs6543144	-0.081091635	0.063644859	0.202618401
basophil	ieu-b-29	pulmonary embolism	rs6557615	-0.076998947	0.063662499	0.22647591
basophil	ieu-b-29	pulmonary embolism	rs6671847	-0.075748169	0.063375025	0.231994485
basophil	ieu-b-29	pulmonary embolism	rs67175901	-0.081788755	0.063697803	0.199137693
basophil	ieu-b-29	pulmonary embolism	rs6734238	-0.07636052	0.063329032	0.227904517
basophil	ieu-b-29	pulmonary embolism	rs6780544	-0.082369289	0.063610408	0.195353797
basophil	ieu-b-29	pulmonary embolism	rs6927569	-0.079764247	0.063786915	0.211124343
basophil	ieu-b-29	pulmonary embolism	rs695113	-0.073381816	0.063163602	0.245327374
basophil	ieu-b-29	pulmonary embolism	rs6993770	-0.08599458	0.063123312	0.173094772
basophil	ieu-b-29	pulmonary embolism	rs7044519	-0.080758399	0.06363507	0.20441027
basophil	ieu-b-29	pulmonary embolism	rs7078507	-0.081701333	0.06365761	0.19933461
basophil	ieu-b-29	pulmonary embolism	rs71391089	-0.079886908	0.06366914	0.209580619
basophil	ieu-b-29	pulmonary embolism	rs71562671	-0.077627127	0.063551057	0.221899679
basophil	ieu-b-29	pulmonary embolism	rs7196129	-0.0733485	0.063187844	0.245722923
basophil	ieu-b-29	pulmonary embolism	rs7250849	-0.07874648	0.063695823	0.216350954
basophil	ieu-b-29	pulmonary embolism	rs7253094	-0.082309299	0.063590922	0.195542441
basophil	ieu-b-29	pulmonary embolism	rs72697295	-0.076612356	0.063434292	0.227145928
basophil	ieu-b-29	pulmonary embolism	rs72721631	-0.079990675	0.063720573	0.209357118
basophil	ieu-b-29	pulmonary embolism	rs72786903	-0.083451112	0.063174392	0.186513295
basophil	ieu-b-29	pulmonary embolism	rs7285377	-0.081122736	0.063653275	0.202504986
basophil	ieu-b-29	pulmonary embolism	rs73022294	-0.080890818	0.063560276	0.203137612
basophil	ieu-b-29	pulmonary embolism	rs73049252	-0.079062738	0.063655618	0.214222303
basophil	ieu-b-29	pulmonary embolism	rs73078376	-0.078270984	0.063621557	0.218600184
basophil	ieu-b-29	pulmonary embolism	rs73110111	-0.077634184	0.063565269	0.221961015
basophil	ieu-b-29	pulmonary embolism	rs73987603	-0.079234529	0.06371414	0.213648988
basophil	ieu-b-29	pulmonary embolism	rs7420	-0.079834109	0.063679306	0.209954706
basophil	ieu-b-29	pulmonary embolism	rs742631	-0.078020107	0.063644489	0.220246196
basophil	ieu-b-29	pulmonary embolism	rs74472890	-0.087716495	0.064013433	0.170598693
basophil	ieu-b-29	pulmonary embolism	rs74524365	-0.081273442	0.063612323	0.201377538
basophil	ieu-b-29	pulmonary embolism	rs74535412	-0.078856693	0.063586007	0.214916973
basophil	ieu-b-29	pulmonary embolism	rs748113	-0.080219412	0.063657362	0.207606264
basophil	ieu-b-29	pulmonary embolism	rs7503461	-0.077563271	0.063571706	0.222430351
basophil	ieu-b-29	pulmonary embolism	rs75084335	-0.080492401	0.063684407	0.206256458
basophil	ieu-b-29	pulmonary embolism	rs7515985	-0.075682779	0.063368134	0.232346957
basophil	ieu-b-29	pulmonary embolism	rs7573465	-0.074414464	0.063274866	0.239574535
basophil	ieu-b-29	pulmonary embolism	rs7613595	-0.085315776	0.063939144	0.182096458
basophil	ieu-b-29	pulmonary embolism	rs76427287	-0.076796951	0.063814384	0.228805072
basophil	ieu-b-29	pulmonary embolism	rs76428106	-0.074710658	0.063464017	0.239110553

basophil	ieu-b-29	pulmonary embolism	rs7684939	-0.078080911	0.063593981	0.219520747
basophil	ieu-b-29	pulmonary embolism	rs7832357	-0.078638127	0.063662149	0.216740648
basophil	ieu-b-29	pulmonary embolism	rs78744187	-0.045705769	0.066754909	0.493545698
basophil	ieu-b-29	pulmonary embolism	rs79140637	-0.083180034	0.063311527	0.188906344
basophil	ieu-b-29	pulmonary embolism	rs80194822	-0.075805194	0.063401045	0.231834806
basophil	ieu-b-29	pulmonary embolism	rs8113682	-0.081044633	0.06369132	0.203209861
basophil	ieu-b-29	pulmonary embolism	rs8178414	-0.089632138	0.063416241	0.157539894
basophil	ieu-b-29	pulmonary embolism	rs875740	-0.085430849	0.063388503	0.177743914
basophil	ieu-b-29	pulmonary embolism	rs905670	-0.076391509	0.063413669	0.228337027
basophil	ieu-b-29	pulmonary embolism	rs915125	-0.082474239	0.063864353	0.196566027
basophil	ieu-b-29	pulmonary embolism	rs9376098	-0.085319654	0.063599896	0.179756159
basophil	ieu-b-29	pulmonary embolism	rs9819371	-0.081075761	0.063649374	0.202738827
basophil	ieu-b-29	pulmonary embolism	rs9928015	-0.081091338	0.063665607	0.202767225
basophil	ieu-b-29	pulmonary embolism	All	-0.079796309	0.063438895	0.208447748
White blood celieu-b-30		pulmonary embolism	rs10006495	-0.131007806	0.041198933	0.001473343
White blood celieu-b-30		pulmonary embolism	rs1007938	-0.131720251	0.041207757	0.001391099
White blood celieu-b-30		pulmonary embolism	rs10087240	-0.132297693	0.041254155	0.001341765
White blood celieu-b-30		pulmonary embolism	rs10102877	-0.13323859	0.041196125	0.001219624
White blood celieu-b-30		pulmonary embolism	rs10138752	-0.131023299	0.041275357	0.001501616
White blood celieu-b-30		pulmonary embolism	rs10146962	-0.131646181	0.041226747	0.001406944
White blood celieu-b-30		pulmonary embolism	rs10164769	-0.136039696	0.041068554	0.000924652
White blood celieu-b-30		pulmonary embolism	rs10203838	-0.132622859	0.041219993	0.0012934
White blood celieu-b-30		pulmonary embolism	rs10205474	-0.132236375	0.041215904	0.001334839
White blood celieu-b-30		pulmonary embolism	rs1024091	-0.133146787	0.041210069	0.001233888
White blood celieu-b-30		pulmonary embolism	rs10260281	-0.131082001	0.041162225	0.001449934
White blood celieu-b-30		pulmonary embolism	rs1033415	-0.133917952	0.041125656	0.001128698
White blood celieu-b-30		pulmonary embolism	rs1042725	-0.131974286	0.041217319	0.00136518
White blood celieu-b-30		pulmonary embolism	rs1047891	-0.128486329	0.041079168	0.001761359
White blood celieu-b-30		pulmonary embolism	rs10494783	-0.129565761	0.041193996	0.001659402
White blood celieu-b-30		pulmonary embolism	rs10498635	-0.129959911	0.041205984	0.001610995
White blood celieu-b-30		pulmonary embolism	rs10808139	-0.130689015	0.041157148	0.001496475
White blood celieu-b-30		pulmonary embolism	rs10808536	-0.132315856	0.041222246	0.001328196
White blood celieu-b-30		pulmonary embolism	rs10814191	-0.132605417	0.041216295	0.001294007
White blood celieu-b-30		pulmonary embolism	rs10828725	-0.129566669	0.04147375	0.001783714
White blood celieu-b-30		pulmonary embolism	rs10844682	-0.134197617	0.041249299	0.001140606
White blood celieu-b-30		pulmonary embolism	rs10864368	-0.133590637	0.041244543	0.001199541
White blood celieu-b-30		pulmonary embolism	rs10872223	-0.13205849	0.041224841	0.001358285
White blood celieu-b-30		pulmonary embolism	rs10889574	-0.133514102	0.041429031	0.001269803
White blood celieu-b-30		pulmonary embolism	rs10931934	-0.131634982	0.041224708	0.001407499
White blood celieu-b-30		pulmonary embolism	rs10936588	-0.133256732	0.041199603	0.00121891
White blood celieu-b-30		pulmonary embolism	rs10940474	-0.131136105	0.041186102	0.001452604
White blood celieu-b-30		pulmonary embolism	rs10980797	-0.132445949	0.04129361	0.001339313
White blood celieu-b-30		pulmonary embolism	rs10986338	-0.132664644	0.041232092	0.001293088
White blood celieu-b-30		pulmonary embolism	rs11002309	-0.13188247	0.041216487	0.001375467

White blood celieu-b-30	pulmonary embolism	rs11022177	-0.13506425	0.041109435	0.001018086
White blood celieu-b-30	pulmonary embolism	rs11064881	-0.132242249	0.041245407	0.001344866
White blood celieu-b-30	pulmonary embolism	rs1109278	-0.132463405	0.041230212	0.001314591
White blood celieu-b-30	pulmonary embolism	rs11104881	-0.138130253	0.041001763	0.000754727
White blood celieu-b-30	pulmonary embolism	rs11114149	-0.131921105	0.041219082	0.001371959
White blood celieu-b-30	pulmonary embolism	rs11159261	-0.133616486	0.041189875	0.001178986
White blood celieu-b-30	pulmonary embolism	rs11169302	-0.134074267	0.041193209	0.001134857
White blood celieu-b-30	pulmonary embolism	rs11198788	-0.13184837	0.041217592	0.00137983
White blood celieu-b-30	pulmonary embolism	rs11221394	-0.131756148	0.041213487	0.001389043
White blood celieu-b-30	pulmonary embolism	rs112282032	-0.130709412	0.041199075	0.00151065
White blood celieu-b-30	pulmonary embolism	rs11242109	-0.138829108	0.041120184	0.000735
White blood celieu-b-30	pulmonary embolism	rs11250076	-0.129977232	0.041206552	0.001608916
White blood celieu-b-30	pulmonary embolism	rs11252331	-0.133355717	0.0411798	0.001202119
White blood celieu-b-30	pulmonary embolism	rs112750178	-0.131642967	0.041211955	0.001401748
White blood celieu-b-30	pulmonary embolism	rs112880875	-0.133634209	0.041187113	0.001176307
White blood celieu-b-30	pulmonary embolism	rs113473633	-0.132175117	0.041223342	0.001344456
White blood celieu-b-30	pulmonary embolism	rs113519804	-0.131835645	0.041240787	0.001389952
White blood celieu-b-30	pulmonary embolism	rs114050631	-0.133988282	0.041352126	0.001194548
White blood celieu-b-30	pulmonary embolism	rs114427331	-0.131125043	0.041239095	0.001474635
White blood celieu-b-30	pulmonary embolism	rs115433278	-0.132837148	0.041198294	0.001262651
White blood celieu-b-30	pulmonary embolism	rs11644125	-0.132983595	0.041235106	0.001259686
White blood celieu-b-30	pulmonary embolism	rs11669443	-0.129742329	0.041019838	0.001561971
White blood celieu-b-30	pulmonary embolism	rs11688303	-0.131486434	0.041207611	0.001418653
White blood celieu-b-30	pulmonary embolism	rs11723621	-0.136171045	0.041139816	0.000933101
White blood celieu-b-30	pulmonary embolism	rs11735662	-0.133462337	0.0412072	0.00120029
White blood celieu-b-30	pulmonary embolism	rs11744663	-0.133378057	0.041203939	0.001207841
White blood celieu-b-30	pulmonary embolism	rs117556162	-0.130999179	0.041165716	0.001461408
White blood celieu-b-30	pulmonary embolism	rs11854390	-0.133568218	0.041183504	0.001181763
White blood celieu-b-30	pulmonary embolism	rs11874453	-0.130873846	0.041152587	0.001471687
White blood celieu-b-30	pulmonary embolism	rs11927257	-0.131692333	0.041208289	0.001394568
White blood celieu-b-30	pulmonary embolism	rs11965885	-0.133929561	0.041176174	0.00114355
White blood celieu-b-30	pulmonary embolism	rs12138789	-0.132265383	0.041225425	0.001335013
White blood celieu-b-30	pulmonary embolism	rs12142474	-0.132373872	0.041225111	0.001322735
White blood celieu-b-30	pulmonary embolism	rs12157427	-0.132778103	0.041202391	0.001270406
White blood celieu-b-30	pulmonary embolism	rs12203592	-0.132653477	0.041201262	0.00128349
White blood celieu-b-30	pulmonary embolism	rs12214269	-0.131133832	0.04122405	0.00146766
White blood celieu-b-30	pulmonary embolism	rs12216862	-0.132859296	0.041204933	0.001262572
White blood celieu-b-30	pulmonary embolism	rs1228024	-0.128074183	0.041131152	0.001846946
White blood celieu-b-30	pulmonary embolism	rs12376511	-0.137754425	0.041093282	0.000801613
White blood celieu-b-30	pulmonary embolism	rs12429714	-0.132803587	0.04120977	0.001270222
White blood celieu-b-30	pulmonary embolism	rs1245035	-0.131909462	0.041228406	0.001376757
White blood celieu-b-30	pulmonary embolism	rs12502008	-0.131337911	0.041184637	0.001427646
White blood celieu-b-30	pulmonary embolism	rs1250215	-0.130640906	0.041116035	0.001486167
White blood celieu-b-30	pulmonary embolism	rs12540307	-0.132173557	0.041296479	0.001371412

White blood celieu-b-30	pulmonary embolism	rs12550612	-0.134920478	0.041253922	0.001073619
White blood celieu-b-30	pulmonary embolism	rs12566440	-0.13022661	0.041137768	0.001547519
White blood celieu-b-30	pulmonary embolism	rs1260326	-0.13176838	0.0413225	0.001428743
White blood celieu-b-30	pulmonary embolism	rs12630592	-0.132547862	0.041221334	0.001302102
White blood celieu-b-30	pulmonary embolism	rs12683699	-0.13082205	0.041206575	0.001499471
White blood celieu-b-30	pulmonary embolism	rs12716647	-0.133253406	0.041190154	0.00121609
White blood celieu-b-30	pulmonary embolism	rs12784071	-0.129527452	0.041067828	0.001610555
White blood celieu-b-30	pulmonary embolism	rs12792460	-0.132432279	0.041229561	0.001317818
White blood celieu-b-30	pulmonary embolism	rs1285254	-0.131000929	0.041133852	0.001448804
White blood celieu-b-30	pulmonary embolism	rs1285886	-0.130436281	0.041223791	0.001555623
White blood celieu-b-30	pulmonary embolism	rs12905862	-0.131701757	0.041203139	0.001391535
White blood celieu-b-30	pulmonary embolism	rs12929950	-0.133535032	0.041241988	0.001204376
White blood celieu-b-30	pulmonary embolism	rs12936529	-0.130498503	0.041259334	0.001562144
White blood celieu-b-30	pulmonary embolism	rs12956324	-0.133279151	0.041197884	0.001216014
White blood celieu-b-30	pulmonary embolism	rs12997100	-0.132929611	0.041198371	0.001252816
White blood celieu-b-30	pulmonary embolism	rs12999905	-0.133093946	0.041181005	0.001229591
White blood celieu-b-30	pulmonary embolism	rs13132853	-0.127618944	0.040986546	0.001847705
White blood celieu-b-30	pulmonary embolism	rs13149209	-0.132096387	0.041228476	0.001355285
White blood celieu-b-30	pulmonary embolism	rs13180726	-0.132907352	0.041228322	0.001265501
White blood celieu-b-30	pulmonary embolism	rs1322599	-0.13393249	0.041231538	0.001160959
White blood celieu-b-30	pulmonary embolism	rs13237635	-0.131036786	0.041153863	0.001452198
White blood celieu-b-30	pulmonary embolism	rs13248936	-0.132510892	0.041225191	0.001307546
White blood celieu-b-30	pulmonary embolism	rs13291664	-0.13223778	0.041242092	0.001344168
White blood celieu-b-30	pulmonary embolism	rs1366045	-0.131107752	0.0412608	0.001485313
White blood celieu-b-30	pulmonary embolism	rs1371045	-0.130838121	0.04116413	0.001480648
White blood celieu-b-30	pulmonary embolism	rs1371794	-0.13292165	0.04163444	0.001410035
White blood celieu-b-30	pulmonary embolism	rs138028125	-0.1316983	0.04121704	0.00139715
White blood celieu-b-30	pulmonary embolism	rs1383265	-0.13301619	0.041228519	0.001253953
White blood celieu-b-30	pulmonary embolism	rs139369470	-0.132594952	0.041198077	0.001288745
White blood celieu-b-30	pulmonary embolism	rs140311179	-0.130266404	0.041080982	0.001519328
White blood celieu-b-30	pulmonary embolism	rs144317085	-0.133309149	0.041259932	0.001233756
White blood celieu-b-30	pulmonary embolism	rs145209947	-0.132677037	0.041225042	0.001289258
White blood celieu-b-30	pulmonary embolism	rs146207734	-0.13215421	0.04121563	0.001344023
White blood celieu-b-30	pulmonary embolism	rs1468102	-0.132240034	0.041225612	0.001337938
White blood celieu-b-30	pulmonary embolism	rs1474419	-0.132985045	0.041235988	0.001259835
White blood celieu-b-30	pulmonary embolism	rs1476081	-0.132862966	0.041207755	0.001263153
White blood celieu-b-30	pulmonary embolism	rs148713124	-0.133200885	0.041206437	0.001227009
White blood celieu-b-30	pulmonary embolism	rs1506609	-0.13174531	0.041245573	0.001402342
White blood celieu-b-30	pulmonary embolism	rs150861794	-0.132275253	0.041221778	0.001332586
White blood celieu-b-30	pulmonary embolism	rs1611236	-0.134277187	0.04129268	0.001146606
White blood celieu-b-30	pulmonary embolism	rs1621453	-0.130819427	0.0411221	0.001466442
White blood celieu-b-30	pulmonary embolism	rs16850073	-0.133879158	0.041405658	0.001223458
White blood celieu-b-30	pulmonary embolism	rs16964983	-0.131897391	0.041236913	0.001381315
White blood celieu-b-30	pulmonary embolism	rs17011726	-0.135028613	0.041114561	0.001022708

White blood celieu-b-30	pulmonary embolism	rs17026212	-0.132665644	0.041228139	0.001291589
White blood celieu-b-30	pulmonary embolism	rs17041439	-0.132679091	0.041222467	0.001288131
White blood celieu-b-30	pulmonary embolism	rs17138597	-0.132899855	0.041202238	0.001257311
White blood celieu-b-30	pulmonary embolism	rs17209630	-0.132849854	0.041217279	0.001267851
White blood celieu-b-30	pulmonary embolism	rs17232826	-0.132091403	0.041222943	0.00135383
White blood celieu-b-30	pulmonary embolism	rs17270882	-0.136714299	0.041159794	0.000895191
White blood celieu-b-30	pulmonary embolism	rs1738074	-0.132680735	0.041253022	0.001298699
White blood celieu-b-30	pulmonary embolism	rs174548	-0.139337795	0.040879977	0.000653305
White blood celieu-b-30	pulmonary embolism	rs175714	-0.132500391	0.041217226	0.001305878
White blood celieu-b-30	pulmonary embolism	rs17758695	-0.12936437	0.041193893	0.001687303
White blood celieu-b-30	pulmonary embolism	rs1779809	-0.133985537	0.041145421	0.001128386
White blood celieu-b-30	pulmonary embolism	rs180941	-0.132804055	0.041225464	0.001275622
White blood celieu-b-30	pulmonary embolism	rs182090955	-0.132423254	0.041212826	0.001312848
White blood celieu-b-30	pulmonary embolism	rs1822534	-0.134459271	0.041242	0.001113136
White blood celieu-b-30	pulmonary embolism	rs1885474	-0.132594886	0.041235706	0.001302013
White blood celieu-b-30	pulmonary embolism	rs1886654	-0.129966413	0.041306683	0.001653065
White blood celieu-b-30	pulmonary embolism	rs1887428	-0.131667209	0.041223785	0.001403345
White blood celieu-b-30	pulmonary embolism	rs1891033	-0.132610597	0.041221595	0.001295306
White blood celieu-b-30	pulmonary embolism	rs1895994	-0.132633355	0.041215505	0.001290675
White blood celieu-b-30	pulmonary embolism	rs1933295	-0.130481193	0.041147217	0.001518723
White blood celieu-b-30	pulmonary embolism	rs1947897	-0.132715004	0.041224891	0.001285071
White blood celieu-b-30	pulmonary embolism	rs2012011	-0.133989027	0.04121265	0.001149341
White blood celieu-b-30	pulmonary embolism	rs2038700	-0.135459076	0.041299326	0.001038313
White blood celieu-b-30	pulmonary embolism	rs2062250	-0.132171354	0.041275948	0.001364108
White blood celieu-b-30	pulmonary embolism	rs2068330	-0.130294199	0.041190889	0.001560558
White blood celieu-b-30	pulmonary embolism	rs2082382	-0.134614646	0.041153564	0.001071556
White blood celieu-b-30	pulmonary embolism	rs2084312	-0.130897256	0.041267664	0.001514407
White blood celieu-b-30	pulmonary embolism	rs2091084	-0.129999888	0.041135549	0.001576171
White blood celieu-b-30	pulmonary embolism	rs2104415	-0.130621163	0.041138245	0.001497457
White blood celieu-b-30	pulmonary embolism	rs2148537	-0.133327285	0.041199773	0.001211677
White blood celieu-b-30	pulmonary embolism	rs2180369	-0.132642791	0.041224039	0.001292644
White blood celieu-b-30	pulmonary embolism	rs218264	-0.128831308	0.041153108	0.001744873
White blood celieu-b-30	pulmonary embolism	rs2194067	-0.132090946	0.041224232	0.001354354
White blood celieu-b-30	pulmonary embolism	rs2259855	-0.1308393	0.041256647	0.001517318
White blood celieu-b-30	pulmonary embolism	rs2260850	-0.131318515	0.041254702	0.001456991
White blood celieu-b-30	pulmonary embolism	rs2282677	-0.134530738	0.041027133	0.001041507
White blood celieu-b-30	pulmonary embolism	rs2282986	-0.136914911	0.04133232	0.000924533
White blood celieu-b-30	pulmonary embolism	rs2284837	-0.13211178	0.04121963	0.001350299
White blood celieu-b-30	pulmonary embolism	rs2286599	-0.128571091	0.041148957	0.001780903
White blood celieu-b-30	pulmonary embolism	rs2290669	-0.134881701	0.04140801	0.00112445
White blood celieu-b-30	pulmonary embolism	rs2298695	-0.133207052	0.041216101	0.001229624
White blood celieu-b-30	pulmonary embolism	rs2305980	-0.132798879	0.041207224	0.001269846
White blood celieu-b-30	pulmonary embolism	rs2312675	-0.132589188	0.041225538	0.001299045
White blood celieu-b-30	pulmonary embolism	rs2314339	-0.134094463	0.041221432	0.001141824

White blood celieu-b-30	pulmonary embolism	rs2327028	-0.131382981	0.041208287	0.001431288
White blood celieu-b-30	pulmonary embolism	rs2328667	-0.129498214	0.040953558	0.001566493
White blood celieu-b-30	pulmonary embolism	rs2358581	-0.134645526	0.041246794	0.00109701
White blood celieu-b-30	pulmonary embolism	rs2371108	-0.134535913	0.041177981	0.001086243
White blood celieu-b-30	pulmonary embolism	rs2394990	-0.129946384	0.041192685	0.001607191
White blood celieu-b-30	pulmonary embolism	rs2412771	-0.129134957	0.04110304	0.001679486
White blood celieu-b-30	pulmonary embolism	rs2427599	-0.131744795	0.041201622	0.00138594
White blood celieu-b-30	pulmonary embolism	rs2428608	-0.133316796	0.041179266	0.00120593
White blood celieu-b-30	pulmonary embolism	rs2432142	-0.129059596	0.040911492	0.001607156
White blood celieu-b-30	pulmonary embolism	rs2439963	-0.132180905	0.041218121	0.001341905
White blood celieu-b-30	pulmonary embolism	rs2469147	-0.13327547	0.041183726	0.001211665
White blood celieu-b-30	pulmonary embolism	rs247826	-0.13139979	0.041312234	0.001469446
White blood celieu-b-30	pulmonary embolism	rs2506699	-0.130782103	0.041177499	0.001492914
White blood celieu-b-30	pulmonary embolism	rs2561758	-0.128981568	0.04128421	0.001782681
White blood celieu-b-30	pulmonary embolism	rs257063	-0.132710306	0.041218731	0.001283426
White blood celieu-b-30	pulmonary embolism	rs2615061	-0.132524587	0.041257818	0.001317646
White blood celieu-b-30	pulmonary embolism	rs2665405	-0.13240669	0.041356013	0.00136652
White blood celieu-b-30	pulmonary embolism	rs2710804	-0.135539804	0.041123992	0.000981134
White blood celieu-b-30	pulmonary embolism	rs2713573	-0.137306751	0.041024061	0.000816968
White blood celieu-b-30	pulmonary embolism	rs2729707	-0.133459974	0.041270918	0.001221749
White blood celieu-b-30	pulmonary embolism	rs2790122	-0.131798348	0.041213107	0.001383979
White blood celieu-b-30	pulmonary embolism	rs284317	-0.132289825	0.041241012	0.001337893
White blood celieu-b-30	pulmonary embolism	rs28530750	-0.133337836	0.041226035	0.001219364
White blood celieu-b-30	pulmonary embolism	rs2853951	-0.117742753	0.041732406	0.004781832
White blood celieu-b-30	pulmonary embolism	rs28571765	-0.130656448	0.041213319	0.001523088
White blood celieu-b-30	pulmonary embolism	rs28574812	-0.132014419	0.041222976	0.001362651
White blood celieu-b-30	pulmonary embolism	rs28576226	-0.13349765	0.041247779	0.001210133
White blood celieu-b-30	pulmonary embolism	rs2857633	-0.132370224	0.041225247	0.001323191
White blood celieu-b-30	pulmonary embolism	rs28588746	-0.13226897	0.041218859	0.001332239
White blood celieu-b-30	pulmonary embolism	rs2875974	-0.13180086	0.041256806	0.001400028
White blood celieu-b-30	pulmonary embolism	rs2904270	-0.133320357	0.041212217	0.001216548
White blood celieu-b-30	pulmonary embolism	rs2935104	-0.131275464	0.041167907	0.001428738
White blood celieu-b-30	pulmonary embolism	rs2943650	-0.133110371	0.041191344	0.001231367
White blood celieu-b-30	pulmonary embolism	rs2993986	-0.127372937	0.041075194	0.001928879
White blood celieu-b-30	pulmonary embolism	rs301817	-0.132230917	0.041233695	0.001341897
White blood celieu-b-30	pulmonary embolism	rs303753	-0.131338816	0.041236699	0.001447549
White blood celieu-b-30	pulmonary embolism	rs309190	-0.131887912	0.04125948	0.001390826
White blood celieu-b-30	pulmonary embolism	rs3184504	-0.146748629	0.041515758	0.000408131
White blood celieu-b-30	pulmonary embolism	rs342294	-0.130911691	0.041214598	0.001491446
White blood celieu-b-30	pulmonary embolism	rs34298354	-0.132460283	0.041230875	0.001315174
White blood celieu-b-30	pulmonary embolism	rs34332006	-0.131558683	0.041196302	0.001405795
White blood celieu-b-30	pulmonary embolism	rs34505104	-0.135937746	0.041055194	0.000929309
White blood celieu-b-30	pulmonary embolism	rs34562738	-0.133228835	0.041208128	0.001224668
White blood celieu-b-30	pulmonary embolism	rs34599082	-0.128285975	0.04114582	0.001821833

White blood celieu-b-30	pulmonary embolism	rs34952318	-0.131687377	0.041229384	0.001403076
White blood celieu-b-30	pulmonary embolism	rs35111447	-0.132982308	0.041188394	0.001243824
White blood celieu-b-30	pulmonary embolism	rs35112940	-0.133988436	0.04124215	0.001158837
White blood celieu-b-30	pulmonary embolism	rs35571080	-0.13559426	0.041142223	0.000981612
White blood celieu-b-30	pulmonary embolism	rs35592432	-0.129607124	0.040994409	0.001569154
White blood celieu-b-30	pulmonary embolism	rs35592645	-0.134595715	0.041099395	0.001057057
White blood celieu-b-30	pulmonary embolism	rs35713889	-0.132649919	0.04120605	0.001285553
White blood celieu-b-30	pulmonary embolism	rs35734242	-0.133228859	0.041252735	0.001239737
White blood celieu-b-30	pulmonary embolism	rs35755865	-0.132840373	0.041218812	0.0012694
White blood celieu-b-30	pulmonary embolism	rs3731332	-0.131018755	0.041323856	0.001521582
White blood celieu-b-30	pulmonary embolism	rs3732378	-0.132927568	0.041223395	0.001261636
White blood celieu-b-30	pulmonary embolism	rs3735485	-0.136347698	0.041188068	0.000931731
White blood celieu-b-30	pulmonary embolism	rs3747869	-0.130471314	0.041298574	0.001581916
White blood celieu-b-30	pulmonary embolism	rs3752562	-0.132327215	0.041221911	0.001326803
White blood celieu-b-30	pulmonary embolism	rs3754224	-0.1313502	0.041207327	0.001434862
White blood celieu-b-30	pulmonary embolism	rs3773312	-0.132400511	0.041224982	0.001319717
White blood celieu-b-30	pulmonary embolism	rs3781454	-0.131265795	0.0412721	0.001470226
White blood celieu-b-30	pulmonary embolism	rs3793537	-0.130762005	0.041203249	0.001505689
White blood celieu-b-30	pulmonary embolism	rs3795503	-0.131934623	0.041230038	0.001374449
White blood celieu-b-30	pulmonary embolism	rs3812849	-0.133539801	0.041212835	0.001194258
White blood celieu-b-30	pulmonary embolism	rs3847147	-0.133074966	0.041195263	0.001236404
White blood celieu-b-30	pulmonary embolism	rs3857488	-0.133345416	0.041215692	0.00121512
White blood celieu-b-30	pulmonary embolism	rs38859	-0.131176679	0.041175837	0.001443701
White blood celieu-b-30	pulmonary embolism	rs399757	-0.133703169	0.041164793	0.001162201
White blood celieu-b-30	pulmonary embolism	rs4074672	-0.135163652	0.0410932	0.001004735
White blood celieu-b-30	pulmonary embolism	rs41272536	-0.132540856	0.041224803	0.001304102
White blood celieu-b-30	pulmonary embolism	rs41295055	-0.130677021	0.041094599	0.001473221
White blood celieu-b-30	pulmonary embolism	rs41313381	-0.132947334	0.041211986	0.001255603
White blood celieu-b-30	pulmonary embolism	rs41317014	-0.130372857	0.041188633	0.001549428
White blood celieu-b-30	pulmonary embolism	rs4239149	-0.131814185	0.041232723	0.001389447
White blood celieu-b-30	pulmonary embolism	rs4276676	-0.132152676	0.041247836	0.00135594
White blood celieu-b-30	pulmonary embolism	rs430989	-0.133811791	0.041168478	0.001152648
White blood celieu-b-30	pulmonary embolism	rs4391200	-0.130665632	0.041238455	0.001532075
White blood celieu-b-30	pulmonary embolism	rs4413892	-0.132895418	0.041291295	0.001288696
White blood celieu-b-30	pulmonary embolism	rs4468717	-0.131115805	0.041194993	0.001458537
White blood celieu-b-30	pulmonary embolism	rs447124	-0.133480296	0.041216005	0.001201369
White blood celieu-b-30	pulmonary embolism	rs4535497	-0.131902331	0.041222772	0.001375495
White blood celieu-b-30	pulmonary embolism	rs464609	-0.133830841	0.041157631	0.00114731
White blood celieu-b-30	pulmonary embolism	rs4672564	-0.131933503	0.041213917	0.001368621
White blood celieu-b-30	pulmonary embolism	rs4703541	-0.134105582	0.041247315	0.001148963
White blood celieu-b-30	pulmonary embolism	rs4704826	-0.130127222	0.041132283	0.001558161
White blood celieu-b-30	pulmonary embolism	rs4707609	-0.130112508	0.0411855	0.001582124
White blood celieu-b-30	pulmonary embolism	rs4721650	-0.13278787	0.041211062	0.001272363
White blood celieu-b-30	pulmonary embolism	rs4729046	-0.132846274	0.041253395	0.001280785

White blood celieu-b-30	pulmonary embolism	rs47341	-0.132485226	0.041233693	0.001313411
White blood celieu-b-30	pulmonary embolism	rs4734879	-0.135723448	0.041075754	0.000952401
White blood celieu-b-30	pulmonary embolism	rs4760	-0.129970425	0.04152311	0.00174762
White blood celieu-b-30	pulmonary embolism	rs4761234	-0.133775916	0.041185511	0.00116166
White blood celieu-b-30	pulmonary embolism	rs4789229	-0.130129906	0.041023981	0.001513696
White blood celieu-b-30	pulmonary embolism	rs4790752	-0.132357447	0.041239327	0.00132968
White blood celieu-b-30	pulmonary embolism	rs4794820	-0.134660619	0.041833258	0.001286453
White blood celieu-b-30	pulmonary embolism	rs4805881	-0.131002156	0.041204934	0.001476398
White blood celieu-b-30	pulmonary embolism	rs4844390	-0.13488999	0.041198143	0.001059704
White blood celieu-b-30	pulmonary embolism	rs4844565	-0.132751708	0.041212663	0.001276823
White blood celieu-b-30	pulmonary embolism	rs4877107	-0.131279381	0.041176545	0.001431576
White blood celieu-b-30	pulmonary embolism	rs4909932	-0.132900498	0.041218937	0.001262994
White blood celieu-b-30	pulmonary embolism	rs4909945	-0.133115906	0.041191566	0.001230862
White blood celieu-b-30	pulmonary embolism	rs4924450	-0.131722242	0.041210416	0.00139186
White blood celieu-b-30	pulmonary embolism	rs4925756	-0.131614937	0.041214973	0.001406194
White blood celieu-b-30	pulmonary embolism	rs4970996	-0.13069259	0.041147846	0.00149233
White blood celieu-b-30	pulmonary embolism	rs507778	-0.137290697	0.041408688	0.000914778
White blood celieu-b-30	pulmonary embolism	rs520910	-0.132871816	0.04120721	0.001262017
White blood celieu-b-30	pulmonary embolism	rs527393	-0.132331011	0.041264384	0.001341707
White blood celieu-b-30	pulmonary embolism	rs533483	-0.133149626	0.041209448	0.001233381
White blood celieu-b-30	pulmonary embolism	rs549302	-0.132276832	0.041255099	0.001344468
White blood celieu-b-30	pulmonary embolism	rs55671712	-0.129691824	0.041092586	0.001599005
White blood celieu-b-30	pulmonary embolism	rs55767800	-0.133906945	0.041206548	0.001155463
White blood celieu-b-30	pulmonary embolism	rs55964818	-0.130342016	0.041346639	0.001619226
White blood celieu-b-30	pulmonary embolism	rs55978995	-0.132667467	0.041203525	0.001282762
White blood celieu-b-30	pulmonary embolism	rs560194	-0.131557881	0.041215765	0.001413251
White blood celieu-b-30	pulmonary embolism	rs56188865	-0.127642456	0.041136552	0.001916368
White blood celieu-b-30	pulmonary embolism	rs56378716	-0.139787518	0.041193176	0.000690149
White blood celieu-b-30	pulmonary embolism	rs56388170	-0.131159935	0.041546638	0.001594382
White blood celieu-b-30	pulmonary embolism	rs571497	-0.129450823	0.041211022	0.001682744
White blood celieu-b-30	pulmonary embolism	rs5746451	-0.131791102	0.041249581	0.001398462
White blood celieu-b-30	pulmonary embolism	rs5746990	-0.135941341	0.040948344	0.000900738
White blood celieu-b-30	pulmonary embolism	rs57871178	-0.134409326	0.041009261	0.001047174
White blood celieu-b-30	pulmonary embolism	rs579721	-0.132095984	0.041217873	0.001351456
White blood celieu-b-30	pulmonary embolism	rs59085061	-0.132095384	0.04128733	0.001377059
White blood celieu-b-30	pulmonary embolism	rs59241311	-0.131490263	0.041184522	0.001409441
White blood celieu-b-30	pulmonary embolism	rs60190483	-0.134612226	0.041088156	0.001052212
White blood celieu-b-30	pulmonary embolism	rs6029234	-0.135297783	0.041197647	0.001023034
White blood celieu-b-30	pulmonary embolism	rs6045615	-0.131517862	0.041236525	0.001425913
White blood celieu-b-30	pulmonary embolism	rs60466842	-0.132067	0.041227808	0.001358399
White blood celieu-b-30	pulmonary embolism	rs6051431	-0.132646728	0.041217072	0.001289766
White blood celieu-b-30	pulmonary embolism	rs6055955	-0.133355224	0.041254326	0.001227057
White blood celieu-b-30	pulmonary embolism	rs6062468	-0.13076912	0.041128381	0.001475116
White blood celieu-b-30	pulmonary embolism	rs60695341	-0.131644001	0.041276098	0.001425922

White blood celieu-b-30	pulmonary embolism	rs61739285	-0.133257164	0.04121269	0.001223256
White blood celieu-b-30	pulmonary embolism	rs61863767	-0.128097965	0.041378356	0.001963011
White blood celieu-b-30	pulmonary embolism	rs62007171	-0.131419251	0.041215959	0.00142987
White blood celieu-b-30	pulmonary embolism	rs62019353	-0.131729281	0.041202436	0.001388054
White blood celieu-b-30	pulmonary embolism	rs62089697	-0.131675396	0.041218565	0.001400416
White blood celieu-b-30	pulmonary embolism	rs62194505	-0.132796014	0.0412091	0.001270805
White blood celieu-b-30	pulmonary embolism	rs62270938	-0.132565109	0.041214131	0.001297661
White blood celieu-b-30	pulmonary embolism	rs62311395	-0.13465288	0.041031238	0.001031774
White blood celieu-b-30	pulmonary embolism	rs62345144	-0.132270197	0.041210767	0.001329185
White blood celieu-b-30	pulmonary embolism	rs62466318	-0.130663507	0.041197131	0.001515676
White blood celieu-b-30	pulmonary embolism	rs62510269	-0.133821419	0.041269502	0.00118435
White blood celieu-b-30	pulmonary embolism	rs626416	-0.131949911	0.041224169	0.001370513
White blood celieu-b-30	pulmonary embolism	rs630505	-0.131481172	0.041195649	0.001414736
White blood celieu-b-30	pulmonary embolism	rs632887	-0.133455444	0.041174352	0.001190161
White blood celieu-b-30	pulmonary embolism	rs6421984	-0.126394805	0.041270853	0.002194464
White blood celieu-b-30	pulmonary embolism	rs6426584	-0.133441588	0.041151843	0.001184179
White blood celieu-b-30	pulmonary embolism	rs6429582	-0.132518418	0.041246966	0.00131446
White blood celieu-b-30	pulmonary embolism	rs6440053	-0.131109968	0.041166395	0.001448148
White blood celieu-b-30	pulmonary embolism	rs6440732	-0.13039755	0.041187054	0.001545594
White blood celieu-b-30	pulmonary embolism	rs6445826	-0.133172427	0.041199168	0.001227526
White blood celieu-b-30	pulmonary embolism	rs6458354	-0.132392603	0.041227559	0.001321522
White blood celieu-b-30	pulmonary embolism	rs6474514	-0.132404511	0.0412203	0.001317598
White blood celieu-b-30	pulmonary embolism	rs6476883	-0.132641395	0.041215321	0.001289733
White blood celieu-b-30	pulmonary embolism	rs6479830	-0.132916653	0.041204813	0.001256406
White blood celieu-b-30	pulmonary embolism	rs6500550	-0.131623377	0.041243417	0.001415954
White blood celieu-b-30	pulmonary embolism	rs6502608	-0.131631035	0.041219263	0.00140591
White blood celieu-b-30	pulmonary embolism	rs6583435	-0.133151386	0.041217685	0.001235985
White blood celieu-b-30	pulmonary embolism	rs6590806	-0.132214211	0.041227745	0.001341628
White blood celieu-b-30	pulmonary embolism	rs6667709	-0.13235356	0.041228355	0.00132617
White blood celieu-b-30	pulmonary embolism	rs6679677	-0.132961494	0.041364822	0.001307349
White blood celieu-b-30	pulmonary embolism	rs6684709	-0.131795619	0.041245204	0.00139629
White blood celieu-b-30	pulmonary embolism	rs672058	-0.131234172	0.04120532	0.00144812
White blood celieu-b-30	pulmonary embolism	rs6731993	-0.132194031	0.041260533	0.001355863
White blood celieu-b-30	pulmonary embolism	rs6734238	-0.128107303	0.041268895	0.001907881
White blood celieu-b-30	pulmonary embolism	rs67491878	-0.132091008	0.041218947	0.001352415
White blood celieu-b-30	pulmonary embolism	rs6755895	-0.128453802	0.041064809	0.001759553
White blood celieu-b-30	pulmonary embolism	rs67684915	-0.132032362	0.04124525	0.001368788
White blood celieu-b-30	pulmonary embolism	rs6779340	-0.130609734	0.041186933	0.001518394
White blood celieu-b-30	pulmonary embolism	rs6831590	-0.136276957	0.041011808	0.00089096
White blood celieu-b-30	pulmonary embolism	rs6854855	-0.134637577	0.041138659	0.001064972
White blood celieu-b-30	pulmonary embolism	rs6878780	-0.130503236	0.041144293	0.001514749
White blood celieu-b-30	pulmonary embolism	rs6924387	-0.130176675	0.041161786	0.001563878
White blood celieu-b-30	pulmonary embolism	rs6948695	-0.132228988	0.04122022	0.001337233
White blood celieu-b-30	pulmonary embolism	rs6952262	-0.135303889	0.040939684	0.00094991

White blood celieu-b-30	pulmonary embolism	rs696	-0.129554195	0.041071254	0.001608415
White blood celieu-b-30	pulmonary embolism	rs6985508	-0.134031287	0.041263366	0.001161413
White blood celieu-b-30	pulmonary embolism	rs7005996	-0.1309478	0.041191713	0.001477922
White blood celieu-b-30	pulmonary embolism	rs703005	-0.131990416	0.04122908	0.001367657
White blood celieu-b-30	pulmonary embolism	rs7036656	-0.13207658	0.041273286	0.001374037
White blood celieu-b-30	pulmonary embolism	rs706809	-0.13325441	0.04123058	0.001229565
White blood celieu-b-30	pulmonary embolism	rs7082470	-0.131593139	0.041304588	0.001442942
White blood celieu-b-30	pulmonary embolism	rs71352239	-0.131087622	0.041175489	0.001454396
White blood celieu-b-30	pulmonary embolism	rs7159281	-0.129951591	0.041053808	0.001548716
White blood celieu-b-30	pulmonary embolism	rs7177	-0.133143258	0.041206738	0.00123313
White blood celieu-b-30	pulmonary embolism	rs7183988	-0.133770007	0.041191375	0.001164136
White blood celieu-b-30	pulmonary embolism	rs718515	-0.133721421	0.041200538	0.001171939
White blood celieu-b-30	pulmonary embolism	rs7198940	-0.134618259	0.041127027	0.001063256
White blood celieu-b-30	pulmonary embolism	rs7225843	-0.130120041	0.041264227	0.00161415
White blood celieu-b-30	pulmonary embolism	rs7235882	-0.131438421	0.041177054	0.001412763
White blood celieu-b-30	pulmonary embolism	rs7246841	-0.133171241	0.041248917	0.001244506
White blood celieu-b-30	pulmonary embolism	rs72664840	-0.131953809	0.041226929	0.001371083
White blood celieu-b-30	pulmonary embolism	rs72726027	-0.129563824	0.041199454	0.001662034
White blood celieu-b-30	pulmonary embolism	rs72747074	-0.131424257	0.041229349	0.001434398
White blood celieu-b-30	pulmonary embolism	rs72780125	-0.132234959	0.04125609	0.00134958
White blood celieu-b-30	pulmonary embolism	rs72790862	-0.129004342	0.041184364	0.001734059
White blood celieu-b-30	pulmonary embolism	rs72836628	-0.13394002	0.041146694	0.001133193
White blood celieu-b-30	pulmonary embolism	rs7283930	-0.132077306	0.041214746	0.001352442
White blood celieu-b-30	pulmonary embolism	rs72843590	-0.131903588	0.041214662	0.001372349
White blood celieu-b-30	pulmonary embolism	rs72951729	-0.131195728	0.041168322	0.0014385
White blood celieu-b-30	pulmonary embolism	rs72973711	-0.130355876	0.04115575	0.001538195
White blood celieu-b-30	pulmonary embolism	rs72978754	-0.131674494	0.04123532	0.001406834
White blood celieu-b-30	pulmonary embolism	rs72982988	-0.130787132	0.041105504	0.001463919
White blood celieu-b-30	pulmonary embolism	rs73000965	-0.130286864	0.04127453	0.001596243
White blood celieu-b-30	pulmonary embolism	rs73028871	-0.131905683	0.041211074	0.001370781
White blood celieu-b-30	pulmonary embolism	rs73036517	-0.127070926	0.041430777	0.002161711
White blood celieu-b-30	pulmonary embolism	rs7308123	-0.135095908	0.041020326	0.000989873
White blood celieu-b-30	pulmonary embolism	rs73190675	-0.131710354	0.041204852	0.00139117
White blood celieu-b-30	pulmonary embolism	rs73191188	-0.135112756	0.041096087	0.001009994
White blood celieu-b-30	pulmonary embolism	rs7326825	-0.132539203	0.041252577	0.001314153
White blood celieu-b-30	pulmonary embolism	rs73738524	-0.132676371	0.041218422	0.001287009
White blood celieu-b-30	pulmonary embolism	rs739842	-0.130731579	0.041180976	0.001500626
White blood celieu-b-30	pulmonary embolism	rs74076327	-0.132916904	0.041182122	0.0012486
White blood celieu-b-30	pulmonary embolism	rs74607840	-0.132641908	0.041227436	0.001293936
White blood celieu-b-30	pulmonary embolism	rs74679834	-0.134474083	0.041063017	0.001057285
White blood celieu-b-30	pulmonary embolism	rs75354229	-0.131641212	0.041231385	0.001409284
White blood celieu-b-30	pulmonary embolism	rs75475627	-0.132753351	0.041244035	0.001287603
White blood celieu-b-30	pulmonary embolism	rs7549164	-0.131373371	0.04126029	0.001452476
White blood celieu-b-30	pulmonary embolism	rs755951	-0.131353206	0.041203005	0.001432843

White blood celieu-b-30	pulmonary embolism	rs7572278	-0.132998778	0.041212149	0.001250194
White blood celieu-b-30	pulmonary embolism	rs7573465	-0.12717204	0.041257377	0.002053337
White blood celieu-b-30	pulmonary embolism	rs759488	-0.135174384	0.040997411	0.000976733
White blood celieu-b-30	pulmonary embolism	rs76378167	-0.13292239	0.041217476	0.001260151
White blood celieu-b-30	pulmonary embolism	rs7639292	-0.132539494	0.041233869	0.001307468
White blood celieu-b-30	pulmonary embolism	rs76428106	-0.128321346	0.041291046	0.001885314
White blood celieu-b-30	pulmonary embolism	rs76545872	-0.133066739	0.041189427	0.001235288
White blood celieu-b-30	pulmonary embolism	rs7705526	-0.133456326	0.041286066	0.001227213
White blood celieu-b-30	pulmonary embolism	rs77215665	-0.132464472	0.041200461	0.001303895
White blood celieu-b-30	pulmonary embolism	rs7751717	-0.132746968	0.041217477	0.001279013
White blood celieu-b-30	pulmonary embolism	rs7776054	-0.126403459	0.041357908	0.002240637
White blood celieu-b-30	pulmonary embolism	rs7776857	-0.130830594	0.041216914	0.001502518
White blood celieu-b-30	pulmonary embolism	rs778125	-0.131555422	0.041213134	0.001412546
White blood celieu-b-30	pulmonary embolism	rs7781268	-0.133774043	0.04116006	0.001153671
White blood celieu-b-30	pulmonary embolism	rs780142	-0.131487234	0.04120777	0.001418618
White blood celieu-b-30	pulmonary embolism	rs7803075	-0.133920855	0.041248064	0.001167438
White blood celieu-b-30	pulmonary embolism	rs78285907	-0.132311408	0.041221314	0.001328359
White blood celieu-b-30	pulmonary embolism	rs7846314	-0.133053263	0.041369031	0.001298764
White blood celieu-b-30	pulmonary embolism	rs7850247	-0.130845251	0.041130471	0.001466541
White blood celieu-b-30	pulmonary embolism	rs7852409	-0.130370506	0.041139017	0.001529521
White blood celieu-b-30	pulmonary embolism	rs7855091	-0.131633754	0.041221019	0.001406252
White blood celieu-b-30	pulmonary embolism	rs7864482	-0.132670826	0.041220218	0.001288242
White blood celieu-b-30	pulmonary embolism	rs7917772	-0.13238116	0.041238002	0.001326547
White blood celieu-b-30	pulmonary embolism	rs79237520	-0.134516408	0.041032188	0.00104429
White blood celieu-b-30	pulmonary embolism	rs79272926	-0.132364469	0.041251304	0.001333207
White blood celieu-b-30	pulmonary embolism	rs7934719	-0.130669915	0.041204389	0.001517781
White blood celieu-b-30	pulmonary embolism	rs796003	-0.130558482	0.04123783	0.001545571
White blood celieu-b-30	pulmonary embolism	rs796056	-0.131734671	0.041246353	0.00140389
White blood celieu-b-30	pulmonary embolism	rs79692389	-0.133495976	0.041184498	0.001189387
White blood celieu-b-30	pulmonary embolism	rs79716587	-0.13023511	0.041204903	0.001574066
White blood celieu-b-30	pulmonary embolism	rs798555	-0.128559974	0.041048489	0.00173676
White blood celieu-b-30	pulmonary embolism	rs806321	-0.130361816	0.041168061	0.001542447
White blood celieu-b-30	pulmonary embolism	rs8084255	-0.133312962	0.041230111	0.001223311
White blood celieu-b-30	pulmonary embolism	rs8176528	-0.133015078	0.041225109	0.001252902
White blood celieu-b-30	pulmonary embolism	rs830623	-0.132696715	0.041213003	0.0012829
White blood celieu-b-30	pulmonary embolism	rs832190	-0.131753215	0.041218533	0.001391272
White blood celieu-b-30	pulmonary embolism	rs8705	-0.128930759	0.041213218	0.001757706
White blood celieu-b-30	pulmonary embolism	rs873218	-0.132995646	0.041204042	0.001247754
White blood celieu-b-30	pulmonary embolism	rs903123	-0.132785918	0.04121298	0.00127324
White blood celieu-b-30	pulmonary embolism	rs921313	-0.132670607	0.041213274	0.001285832
White blood celieu-b-30	pulmonary embolism	rs9313822	-0.131305365	0.041175797	0.001428169
White blood celieu-b-30	pulmonary embolism	rs9323285	-0.132756981	0.041215782	0.001277339
White blood celieu-b-30	pulmonary embolism	rs9330650	-0.13186278	0.041210879	0.001375669
White blood celieu-b-30	pulmonary embolism	rs9348970	-0.135143684	0.041191849	0.001035001

White blood celieu-b-30		pulmonary embolism	rs935655	-0.131950828	0.041226554	0.001371288
White blood celieu-b-30		pulmonary embolism	rs9375447	-0.130937247	0.041185768	0.001476889
White blood celieu-b-30		pulmonary embolism	rs9382100	-0.133981115	0.041118913	0.001120496
White blood celieu-b-30		pulmonary embolism	rs9390461	-0.135575358	0.04101545	0.000948171
White blood celieu-b-30		pulmonary embolism	rs9429767	-0.132546064	0.041230503	0.001305548
White blood celieu-b-30		pulmonary embolism	rs9430574	-0.132610508	0.041226531	0.001297056
White blood celieu-b-30		pulmonary embolism	rs9487043	-0.132855231	0.041264894	0.001283828
White blood celieu-b-30		pulmonary embolism	rs9508005	-0.131730922	0.041208942	0.001390293
White blood celieu-b-30		pulmonary embolism	rs9526795	-0.133184317	0.041200622	0.001226777
White blood celieu-b-30		pulmonary embolism	rs9625746	-0.136653862	0.040922029	0.000839682
White blood celieu-b-30		pulmonary embolism	rs9656395	-0.132937983	0.041221202	0.001259769
White blood celieu-b-30		pulmonary embolism	rs9819371	-0.133318251	0.041239122	0.00122579
White blood celieu-b-30		pulmonary embolism	rs9829114	-0.130600639	0.041327076	0.001576764
White blood celieu-b-30		pulmonary embolism	rs9835104	-0.133385409	0.04116874	0.00119543
White blood celieu-b-30		pulmonary embolism	rs9863	-0.129901266	0.041154439	0.001597111
White blood celieu-b-30		pulmonary embolism	rs9867398	-0.131867729	0.041224905	0.001380298
White blood celieu-b-30		pulmonary embolism	rs987107	-0.12975363	0.041162368	0.001620235
White blood celieu-b-30		pulmonary embolism	rs9885207	-0.13009721	0.041156946	0.001572271
White blood celieu-b-30		pulmonary embolism	rs9900613	-0.129646068	0.040959806	0.001549752
White blood celieu-b-30		pulmonary embolism	rs990558	-0.132048079	0.041218782	0.001357256
White blood celieu-b-30		pulmonary embolism	rs9925985	-0.13227704	0.041224712	0.001333443
White blood celieu-b-30		pulmonary embolism	rs9926183	-0.131812622	0.04123175	0.001389266
White blood celieu-b-30		pulmonary embolism	rs9938104	-0.134133133	0.041051423	0.001085242
White blood celieu-b-30		pulmonary embolism	rs9977672	-0.135003819	0.041145395	0.001033883
White blood celieu-b-30		pulmonary embolism	All	-0.132265795	0.041160666	0.001311722
monocyte	ieu-b-31	pulmonary embolism	rs1002774	-0.012965215	0.031535913	0.680980578
monocyte	ieu-b-31	pulmonary embolism	rs10094039	-0.010589358	0.03197703	0.740527302
monocyte	ieu-b-31	pulmonary embolism	rs10197805	-0.013906129	0.031578435	0.659670664
monocyte	ieu-b-31	pulmonary embolism	rs10238435	-0.016339836	0.031557037	0.604606622
monocyte	ieu-b-31	pulmonary embolism	rs1031091	-0.015351207	0.031561569	0.626691244
monocyte	ieu-b-31	pulmonary embolism	rs10403909	-0.015449868	0.031570845	0.624578796
monocyte	ieu-b-31	pulmonary embolism	rs10409752	-0.015369309	0.031561829	0.626287552
monocyte	ieu-b-31	pulmonary embolism	rs10420703	-0.016888333	0.031429466	0.591032259
monocyte	ieu-b-31	pulmonary embolism	rs10437954	-0.015368072	0.031562813	0.626326089
monocyte	ieu-b-31	pulmonary embolism	rs10460159	-0.015821131	0.031560097	0.61615878
monocyte	ieu-b-31	pulmonary embolism	rs10478058	-0.015072429	0.031560702	0.632956624
monocyte	ieu-b-31	pulmonary embolism	rs10518329	-0.014797869	0.031549386	0.639042293
monocyte	ieu-b-31	pulmonary embolism	rs1052484	-0.015341803	0.031562381	0.626911331
monocyte	ieu-b-31	pulmonary embolism	rs10780209	-0.014237346	0.031665354	0.652985161
monocyte	ieu-b-31	pulmonary embolism	rs10796828	-0.014950335	0.031564936	0.635758468
monocyte	ieu-b-31	pulmonary embolism	rs10814193	-0.015515627	0.031565935	0.623051122
monocyte	ieu-b-31	pulmonary embolism	rs10828725	-0.012823946	0.031646529	0.685312579
monocyte	ieu-b-31	pulmonary embolism	rs10831507	-0.014590961	0.031517239	0.643398956
monocyte	ieu-b-31	pulmonary embolism	rs10849020	-0.015221245	0.031577749	0.629788627

monocyte	ieu-b-31	pulmonary embolism	rs10849448	-0.012814808	0.031625599	0.68532783
monocyte	ieu-b-31	pulmonary embolism	rs10892342	-0.014074836	0.03151798	0.655188944
monocyte	ieu-b-31	pulmonary embolism	rs1091815	-0.016131647	0.031520285	0.608800654
monocyte	ieu-b-31	pulmonary embolism	rs10935473	-0.017295747	0.031529653	0.583310904
monocyte	ieu-b-31	pulmonary embolism	rs10948314	-0.016185824	0.031570831	0.608173184
monocyte	ieu-b-31	pulmonary embolism	rs11057841	-0.016036029	0.031541723	0.611168023
monocyte	ieu-b-31	pulmonary embolism	rs11082397	-0.014954823	0.031557002	0.635572132
monocyte	ieu-b-31	pulmonary embolism	rs11086102	-0.017037293	0.031572176	0.589452222
monocyte	ieu-b-31	pulmonary embolism	rs11104881	-0.017402141	0.031387045	0.579279755
monocyte	ieu-b-31	pulmonary embolism	rs11130612	-0.014547928	0.03151905	0.644396987
monocyte	ieu-b-31	pulmonary embolism	rs111456533	-0.016364299	0.031430927	0.602615338
monocyte	ieu-b-31	pulmonary embolism	rs111556916	-0.015124577	0.03154341	0.63159397
monocyte	ieu-b-31	pulmonary embolism	rs11155787	-0.015303758	0.031565388	0.627798931
monocyte	ieu-b-31	pulmonary embolism	rs11170652	-0.016767827	0.031482604	0.594306279
monocyte	ieu-b-31	pulmonary embolism	rs11189154	-0.010873612	0.031581479	0.730618098
monocyte	ieu-b-31	pulmonary embolism	rs11190141	-0.015093034	0.031662571	0.633587321
monocyte	ieu-b-31	pulmonary embolism	rs112248289	-0.015569037	0.031559054	0.621779251
monocyte	ieu-b-31	pulmonary embolism	rs11235689	-0.016012339	0.031551549	0.611805637
monocyte	ieu-b-31	pulmonary embolism	rs11242109	-0.017736666	0.031456613	0.572859371
monocyte	ieu-b-31	pulmonary embolism	rs11246065	-0.015854657	0.031569125	0.615512524
monocyte	ieu-b-31	pulmonary embolism	rs11247908	-0.01532672	0.031587941	0.627528842
monocyte	ieu-b-31	pulmonary embolism	rs11252148	-0.015629938	0.031556608	0.620389482
monocyte	ieu-b-31	pulmonary embolism	rs11255548	-0.01614891	0.031559181	0.608859271
monocyte	ieu-b-31	pulmonary embolism	rs1127101	-0.014346507	0.031521404	0.649011833
monocyte	ieu-b-31	pulmonary embolism	rs113015223	-0.014986516	0.031561569	0.634905069
monocyte	ieu-b-31	pulmonary embolism	rs113143864	-0.013643662	0.031464316	0.664561982
monocyte	ieu-b-31	pulmonary embolism	rs113155021	-0.016811696	0.031507738	0.593636608
monocyte	ieu-b-31	pulmonary embolism	rs113292043	-0.016242423	0.03149907	0.606100496
monocyte	ieu-b-31	pulmonary embolism	rs1146933	-0.014907089	0.031547701	0.636551845
monocyte	ieu-b-31	pulmonary embolism	rs11508026	-0.014021111	0.031447111	0.65569603
monocyte	ieu-b-31	pulmonary embolism	rs115202835	-0.015323249	0.031567936	0.627388759
monocyte	ieu-b-31	pulmonary embolism	rs115340020	-0.014742382	0.031564738	0.64046248
monocyte	ieu-b-31	pulmonary embolism	rs11557154	-0.01697364	0.031525659	0.590295998
monocyte	ieu-b-31	pulmonary embolism	rs11590380	-0.015572142	0.03158258	0.621969404
monocyte	ieu-b-31	pulmonary embolism	rs11602323	-0.011165679	0.031613596	0.723944267
monocyte	ieu-b-31	pulmonary embolism	rs11629297	-0.011013514	0.031707351	0.72832921
monocyte	ieu-b-31	pulmonary embolism	rs11631419	-0.015971558	0.031533003	0.612503639
monocyte	ieu-b-31	pulmonary embolism	rs11644125	-0.015656281	0.031573599	0.619988808
monocyte	ieu-b-31	pulmonary embolism	rs11655888	-0.0158853	0.03154394	0.614547573
monocyte	ieu-b-31	pulmonary embolism	rs116619972	-0.014580449	0.031528921	0.643760935
monocyte	ieu-b-31	pulmonary embolism	rs11713343	-0.01770423	0.0314583	0.573581926
monocyte	ieu-b-31	pulmonary embolism	rs11723621	-0.016285128	0.031480871	0.604945119
monocyte	ieu-b-31	pulmonary embolism	rs11792030	-0.014000337	0.031464195	0.656348012
monocyte	ieu-b-31	pulmonary embolism	rs1180658	-0.015805156	0.031546234	0.616360135

monocyte	ieu-b-31	pulmonary embolism	rs118083884	-0.015072186	0.031568992	0.633051397
monocyte	ieu-b-31	pulmonary embolism	rs11844354	-0.01531567	0.031562903	0.627504146
monocyte	ieu-b-31	pulmonary embolism	rs11854390	-0.017631898	0.031623107	0.577142316
monocyte	ieu-b-31	pulmonary embolism	rs11857609	-0.014391346	0.031562714	0.648418489
monocyte	ieu-b-31	pulmonary embolism	rs11965885	-0.015959692	0.031526418	0.612693574
monocyte	ieu-b-31	pulmonary embolism	rs12055642	-0.016072683	0.031518809	0.610093913
monocyte	ieu-b-31	pulmonary embolism	rs12118443	-0.015873744	0.031565216	0.615043567
monocyte	ieu-b-31	pulmonary embolism	rs12144117	-0.015188447	0.031563124	0.630368233
monocyte	ieu-b-31	pulmonary embolism	rs12198236	-0.016388079	0.031457694	0.602397308
monocyte	ieu-b-31	pulmonary embolism	rs12207691	-0.015274397	0.031561614	0.628417905
monocyte	ieu-b-31	pulmonary embolism	rs12306790	-0.015726456	0.031568258	0.618362064
monocyte	ieu-b-31	pulmonary embolism	rs12327253	-0.014697872	0.031558241	0.641403036
monocyte	ieu-b-31	pulmonary embolism	rs12332674	-0.015800324	0.031547771	0.616485112
monocyte	ieu-b-31	pulmonary embolism	rs12376511	-0.01968407	0.031507867	0.532144939
monocyte	ieu-b-31	pulmonary embolism	rs12461422	-0.015951415	0.031552273	0.613168961
monocyte	ieu-b-31	pulmonary embolism	rs12480462	-0.017467729	0.03156227	0.579964181
monocyte	ieu-b-31	pulmonary embolism	rs12480732	-0.014194479	0.031672901	0.654038831
monocyte	ieu-b-31	pulmonary embolism	rs12522498	-0.015454177	0.03156034	0.624366869
monocyte	ieu-b-31	pulmonary embolism	rs12530071	-0.016369032	0.031512456	0.60344949
monocyte	ieu-b-31	pulmonary embolism	rs12542907	-0.015425631	0.031585126	0.625278761
monocyte	ieu-b-31	pulmonary embolism	rs12694733	-0.015517542	0.031559758	0.622940188
monocyte	ieu-b-31	pulmonary embolism	rs12742428	-0.013941782	0.031504746	0.658106034
monocyte	ieu-b-31	pulmonary embolism	rs12747432	-0.013963604	0.03147919	0.657344774
monocyte	ieu-b-31	pulmonary embolism	rs1283945	-0.015634845	0.031533899	0.62002795
monocyte	ieu-b-31	pulmonary embolism	rs12874404	-0.014775925	0.031582354	0.639889132
monocyte	ieu-b-31	pulmonary embolism	rs12934481	-0.015566501	0.031559872	0.621845049
monocyte	ieu-b-31	pulmonary embolism	rs12941356	-0.016583857	0.031520658	0.598800245
monocyte	ieu-b-31	pulmonary embolism	rs12993696	-0.015185562	0.031564245	0.630445336
monocyte	ieu-b-31	pulmonary embolism	rs13027410	-0.014827395	0.03154714	0.638349599
monocyte	ieu-b-31	pulmonary embolism	rs13032491	-0.015397022	0.031561657	0.625663552
monocyte	ieu-b-31	pulmonary embolism	rs13032786	-0.018208116	0.031358245	0.561477424
monocyte	ieu-b-31	pulmonary embolism	rs13073885	-0.015070244	0.031559797	0.632996163
monocyte	ieu-b-31	pulmonary embolism	rs13077681	-0.015711585	0.031554396	0.618539979
monocyte	ieu-b-31	pulmonary embolism	rs13094390	-0.016327462	0.031527669	0.604543925
monocyte	ieu-b-31	pulmonary embolism	rs13167280	-0.016453925	0.031486553	0.601273579
monocyte	ieu-b-31	pulmonary embolism	rs13225324	-0.014348079	0.031500197	0.64875552
monocyte	ieu-b-31	pulmonary embolism	rs13229868	-0.015065668	0.031557155	0.633070944
monocyte	ieu-b-31	pulmonary embolism	rs13238198	-0.016737301	0.031483851	0.594992386
monocyte	ieu-b-31	pulmonary embolism	rs13267464	-0.01507693	0.031578166	0.633043188
monocyte	ieu-b-31	pulmonary embolism	rs13271228	-0.01400139	0.031579821	0.657501683
monocyte	ieu-b-31	pulmonary embolism	rs1335929	-0.014649606	0.031555216	0.642466443
monocyte	ieu-b-31	pulmonary embolism	rs1367153	-0.015559304	0.031557921	0.62198463
monocyte	ieu-b-31	pulmonary embolism	rs138028125	-0.014305456	0.031604531	0.650808424
monocyte	ieu-b-31	pulmonary embolism	rs139271	-0.014659005	0.031551454	0.642213397

monocyte	ieu-b-31	pulmonary embolism	rs1400003	-0.01552802	0.03155526	0.622655899
monocyte	ieu-b-31	pulmonary embolism	rs141936164	-0.016800028	0.031506426	0.593877532
monocyte	ieu-b-31	pulmonary embolism	rs143273199	-0.010110345	0.031694967	0.749734869
monocyte	ieu-b-31	pulmonary embolism	rs144317085	-0.015842152	0.031598512	0.616119517
monocyte	ieu-b-31	pulmonary embolism	rs145447411	-0.01527874	0.031563507	0.628340841
monocyte	ieu-b-31	pulmonary embolism	rs145718079	-0.01674116	0.03149289	0.595013193
monocyte	ieu-b-31	pulmonary embolism	rs146039611	-0.015665582	0.031598227	0.620053802
monocyte	ieu-b-31	pulmonary embolism	rs146474125	-0.01481098	0.031552704	0.638780548
monocyte	ieu-b-31	pulmonary embolism	rs146681239	-0.01515008	0.031562051	0.631220725
monocyte	ieu-b-31	pulmonary embolism	rs1468488	-0.015394798	0.031602736	0.626162694
monocyte	ieu-b-31	pulmonary embolism	rs147341073	-0.014708343	0.031560202	0.641186268
monocyte	ieu-b-31	pulmonary embolism	rs149007767	-0.018331366	0.031720645	0.56333152
monocyte	ieu-b-31	pulmonary embolism	rs149110519	-0.015454022	0.031587155	0.624664559
monocyte	ieu-b-31	pulmonary embolism	rs149902937	-0.015712672	0.031552118	0.618490359
monocyte	ieu-b-31	pulmonary embolism	rs150449635	-0.016540428	0.031572039	0.600351693
monocyte	ieu-b-31	pulmonary embolism	rs1535701	-0.015568546	0.031576916	0.621987377
monocyte	ieu-b-31	pulmonary embolism	rs1587222	-0.015401788	0.03156506	0.625593856
monocyte	ieu-b-31	pulmonary embolism	rs1594541	-0.015924852	0.031538166	0.613601633
monocyte	ieu-b-31	pulmonary embolism	rs1611236	-0.016114722	0.031592834	0.609998764
monocyte	ieu-b-31	pulmonary embolism	rs16831132	-0.016977456	0.03142034	0.58896721
monocyte	ieu-b-31	pulmonary embolism	rs16939607	-0.015774231	0.031568215	0.617295386
monocyte	ieu-b-31	pulmonary embolism	rs16940328	-0.014312182	0.03157417	0.650341598
monocyte	ieu-b-31	pulmonary embolism	rs16978176	-0.015473919	0.031591829	0.624270022
monocyte	ieu-b-31	pulmonary embolism	rs17005891	-0.014085804	0.031653034	0.656315098
monocyte	ieu-b-31	pulmonary embolism	rs17011726	-0.016825057	0.031487111	0.593101182
monocyte	ieu-b-31	pulmonary embolism	rs17086239	-0.015725483	0.031575306	0.618462145
monocyte	ieu-b-31	pulmonary embolism	rs17156536	-0.015003713	0.031574586	0.634656397
monocyte	ieu-b-31	pulmonary embolism	rs17196752	-0.014166894	0.031764159	0.655595448
monocyte	ieu-b-31	pulmonary embolism	rs17387886	-0.014197295	0.031566806	0.652887873
monocyte	ieu-b-31	pulmonary embolism	rs1749824	-0.014373671	0.031503157	0.648202109
monocyte	ieu-b-31	pulmonary embolism	rs17498743	-0.015781419	0.031559441	0.617037179
monocyte	ieu-b-31	pulmonary embolism	rs17544073	-0.014271813	0.031477168	0.650259249
monocyte	ieu-b-31	pulmonary embolism	rs17575497	-0.036046026	0.03217115	0.262523881
monocyte	ieu-b-31	pulmonary embolism	rs17656204	-0.01479545	0.031661594	0.640285547
monocyte	ieu-b-31	pulmonary embolism	rs17700436	-0.015627673	0.031559138	0.620468158
monocyte	ieu-b-31	pulmonary embolism	rs17731	-0.015870686	0.031546465	0.614901547
monocyte	ieu-b-31	pulmonary embolism	rs17758695	-0.011465038	0.03159667	0.716712521
monocyte	ieu-b-31	pulmonary embolism	rs17776203	-0.015248067	0.031562171	0.629016134
monocyte	ieu-b-31	pulmonary embolism	rs1781799	-0.015059336	0.031557875	0.633221556
monocyte	ieu-b-31	pulmonary embolism	rs17860428	-0.015167615	0.031563669	0.630843246
monocyte	ieu-b-31	pulmonary embolism	rs1800973	-0.012231338	0.031659459	0.699244344
monocyte	ieu-b-31	pulmonary embolism	rs1822534	-0.016626339	0.031586045	0.598622525
monocyte	ieu-b-31	pulmonary embolism	rs183363123	-0.015265266	0.031575309	0.628772141
monocyte	ieu-b-31	pulmonary embolism	rs1863651	-0.01624803	0.031532916	0.606362931

monocyte	ieu-b-31	pulmonary embolism	rs1869365	-0.015857188	0.031565518	0.615415769
monocyte	ieu-b-31	pulmonary embolism	rs188175496	-0.017590958	0.031540523	0.577031744
monocyte	ieu-b-31	pulmonary embolism	rs188393352	-0.013325281	0.031477266	0.672054283
monocyte	ieu-b-31	pulmonary embolism	rs1885525	-0.015314985	0.031565656	0.627549578
monocyte	ieu-b-31	pulmonary embolism	rs1889033	-0.016619939	0.031502394	0.59779294
monocyte	ieu-b-31	pulmonary embolism	rs1892548	-0.015543004	0.031743088	0.624381565
monocyte	ieu-b-31	pulmonary embolism	rs1902796	-0.015926217	0.031533809	0.613522245
monocyte	ieu-b-31	pulmonary embolism	rs190982	-0.014077515	0.031448558	0.654415732
monocyte	ieu-b-31	pulmonary embolism	rs1930303	-0.0122306	0.031729287	0.69989129
monocyte	ieu-b-31	pulmonary embolism	rs1933295	-0.013492514	0.031510407	0.668511104
monocyte	ieu-b-31	pulmonary embolism	rs1954735	-0.015310521	0.031573047	0.627730431
monocyte	ieu-b-31	pulmonary embolism	rs1967309	-0.016865742	0.031531929	0.592734062
monocyte	ieu-b-31	pulmonary embolism	rs1970364	-0.015285435	0.031576302	0.628329572
monocyte	ieu-b-31	pulmonary embolism	rs1976633	-0.014526394	0.031524963	0.644949126
monocyte	ieu-b-31	pulmonary embolism	rs1992057	-0.015783269	0.031547604	0.616863781
monocyte	ieu-b-31	pulmonary embolism	rs1997577	-0.014915478	0.031581994	0.636728441
monocyte	ieu-b-31	pulmonary embolism	rs2013605	-0.014352272	0.031497645	0.648633242
monocyte	ieu-b-31	pulmonary embolism	rs2022003	-0.015763214	0.031563081	0.61748398
monocyte	ieu-b-31	pulmonary embolism	rs2038700	-0.017043617	0.031622391	0.589905437
monocyte	ieu-b-31	pulmonary embolism	rs205422	-0.014748549	0.031541907	0.64008081
monocyte	ieu-b-31	pulmonary embolism	rs2063996	-0.015096796	0.031559802	0.632397397
monocyte	ieu-b-31	pulmonary embolism	rs2084312	-0.013896756	0.031597878	0.660081756
monocyte	ieu-b-31	pulmonary embolism	rs2106135	-0.01620881	0.031544923	0.607369022
monocyte	ieu-b-31	pulmonary embolism	rs2136451	-0.01448533	0.031533325	0.645971521
monocyte	ieu-b-31	pulmonary embolism	rs2176777	-0.014138018	0.031640961	0.655000378
monocyte	ieu-b-31	pulmonary embolism	rs2184697	-0.015658628	0.031559677	0.619782008
monocyte	ieu-b-31	pulmonary embolism	rs2213290	-0.020574097	0.031382073	0.512081331
monocyte	ieu-b-31	pulmonary embolism	rs2228467	-0.012600557	0.0319405	0.693211237
monocyte	ieu-b-31	pulmonary embolism	rs2239630	-0.013297765	0.031673748	0.674605612
monocyte	ieu-b-31	pulmonary embolism	rs224111	-0.015401668	0.031579633	0.625756051
monocyte	ieu-b-31	pulmonary embolism	rs2253843	-0.015876276	0.031531463	0.61460867
monocyte	ieu-b-31	pulmonary embolism	rs2273215	-0.014359485	0.031545373	0.648964648
monocyte	ieu-b-31	pulmonary embolism	rs2274664	-0.01512563	0.03156709	0.631826102
monocyte	ieu-b-31	pulmonary embolism	rs2295481	-0.016195651	0.031516578	0.607337838
monocyte	ieu-b-31	pulmonary embolism	rs2302774	-0.013730128	0.031539216	0.663319216
monocyte	ieu-b-31	pulmonary embolism	rs231988	-0.017107932	0.031431537	0.586240613
monocyte	ieu-b-31	pulmonary embolism	rs2327028	-0.014801882	0.031543176	0.638885358
monocyte	ieu-b-31	pulmonary embolism	rs2338021	-0.016086203	0.03156768	0.610346966
monocyte	ieu-b-31	pulmonary embolism	rs2371108	-0.015994921	0.031519563	0.611832022
monocyte	ieu-b-31	pulmonary embolism	rs2385094	-0.015258559	0.031571424	0.628880723
monocyte	ieu-b-31	pulmonary embolism	rs2504209	-0.015115827	0.031568301	0.632060078
monocyte	ieu-b-31	pulmonary embolism	rs2523562	-0.017589165	0.031730515	0.579353253
monocyte	ieu-b-31	pulmonary embolism	rs2535393	-0.014582627	0.03154656	0.643896828
monocyte	ieu-b-31	pulmonary embolism	rs2548257	-0.015367362	0.031564686	0.626362508

monocyte	ieu-b-31	pulmonary embolism	rs2665405	-0.014850312	0.0316372	0.638788029
monocyte	ieu-b-31	pulmonary embolism	rs2711981	-0.01433913	0.031561621	0.649597288
monocyte	ieu-b-31	pulmonary embolism	rs2729450	-0.013880508	0.031588104	0.660355795
monocyte	ieu-b-31	pulmonary embolism	rs2734047	-0.022342835	0.031753061	0.481654827
monocyte	ieu-b-31	pulmonary embolism	rs2734442	-0.013442079	0.031555586	0.670121723
monocyte	ieu-b-31	pulmonary embolism	rs2784250	-0.015442998	0.031561336	0.624628497
monocyte	ieu-b-31	pulmonary embolism	rs2797681	-0.015710324	0.031549503	0.618513716
monocyte	ieu-b-31	pulmonary embolism	rs2810883	-0.014787374	0.031546067	0.639244836
monocyte	ieu-b-31	pulmonary embolism	rs2817441	-0.016374789	0.031548264	0.603733328
monocyte	ieu-b-31	pulmonary embolism	rs2834670	-0.013742098	0.031520822	0.662859283
monocyte	ieu-b-31	pulmonary embolism	rs2836220	-0.016210607	0.031502985	0.606850972
monocyte	ieu-b-31	pulmonary embolism	rs28367597	-0.014295213	0.031551426	0.650493575
monocyte	ieu-b-31	pulmonary embolism	rs284324	-0.01504569	0.031578625	0.6337526
monocyte	ieu-b-31	pulmonary embolism	rs2846573	-0.01462604	0.031522051	0.642652107
monocyte	ieu-b-31	pulmonary embolism	rs28498283	-0.016703307	0.031520861	0.596172414
monocyte	ieu-b-31	pulmonary embolism	rs28612496	-0.015517544	0.031559484	0.62293713
monocyte	ieu-b-31	pulmonary embolism	rs28722705	-0.014052156	0.031542604	0.655960143
monocyte	ieu-b-31	pulmonary embolism	rs28723530	-0.015215779	0.031562901	0.629750551
monocyte	ieu-b-31	pulmonary embolism	rs290243	-0.016012544	0.031542001	0.61169333
monocyte	ieu-b-31	pulmonary embolism	rs2927	-0.016439685	0.031526937	0.602054134
monocyte	ieu-b-31	pulmonary embolism	rs2978889	-0.014150282	0.031540691	0.653694428
monocyte	ieu-b-31	pulmonary embolism	rs30102	-0.016149512	0.031504839	0.608228222
monocyte	ieu-b-31	pulmonary embolism	rs3012415	-0.014608919	0.031541222	0.643243159
monocyte	ieu-b-31	pulmonary embolism	rs3014807	-0.015850507	0.031554464	0.615440915
monocyte	ieu-b-31	pulmonary embolism	rs3027012	-0.01538221	0.031579869	0.626195188
monocyte	ieu-b-31	pulmonary embolism	rs3111414	-0.015424084	0.031566717	0.625111776
monocyte	ieu-b-31	pulmonary embolism	rs31243	-0.015539657	0.031565792	0.622511371
monocyte	ieu-b-31	pulmonary embolism	rs3128959	-0.013052295	0.031477616	0.678395781
monocyte	ieu-b-31	pulmonary embolism	rs3177609	-0.01551726	0.031571595	0.623076867
monocyte	ieu-b-31	pulmonary embolism	rs3184504	-0.020074867	0.031574697	0.524913506
monocyte	ieu-b-31	pulmonary embolism	rs329125	-0.014668857	0.031541461	0.641884284
monocyte	ieu-b-31	pulmonary embolism	rs333947	-0.015866121	0.031594154	0.615537152
monocyte	ieu-b-31	pulmonary embolism	rs34097845	-0.017132438	0.031727083	0.589201096
monocyte	ieu-b-31	pulmonary embolism	rs34236350	-0.015932809	0.031596817	0.614083184
monocyte	ieu-b-31	pulmonary embolism	rs34281413	-0.015660179	0.031556471	0.619711774
monocyte	ieu-b-31	pulmonary embolism	rs34417629	-0.015319424	0.031563176	0.627422779
monocyte	ieu-b-31	pulmonary embolism	rs34478611	-0.015027848	0.031554252	0.633893223
monocyte	ieu-b-31	pulmonary embolism	rs34505104	-0.018754087	0.031473631	0.551264317
monocyte	ieu-b-31	pulmonary embolism	rs34600126	-0.014362706	0.031546206	0.648899844
monocyte	ieu-b-31	pulmonary embolism	rs34743120	-0.015754752	0.031535864	0.617369387
monocyte	ieu-b-31	pulmonary embolism	rs34746889	-0.014985002	0.031541166	0.634720348
monocyte	ieu-b-31	pulmonary embolism	rs35068491	-0.014234614	0.031464074	0.650974736
monocyte	ieu-b-31	pulmonary embolism	rs35108724	-0.015331586	0.031562617	0.627143424
monocyte	ieu-b-31	pulmonary embolism	rs35979828	-0.014483082	0.031529351	0.645981161

monocyte	ieu-b-31	pulmonary embolism	rs360017	-0.014036529	0.031541683	0.656308749
monocyte	ieu-b-31	pulmonary embolism	rs36026517	-0.015411637	0.031565555	0.625378267
monocyte	ieu-b-31	pulmonary embolism	rs3731211	-0.014947378	0.031585888	0.636049369
monocyte	ieu-b-31	pulmonary embolism	rs3731332	-0.014247734	0.031589076	0.651965345
monocyte	ieu-b-31	pulmonary embolism	rs3761986	-0.016277214	0.031500942	0.605350578
monocyte	ieu-b-31	pulmonary embolism	rs3762281	-0.015067075	0.03156199	0.63309128
monocyte	ieu-b-31	pulmonary embolism	rs3789062	-0.015210185	0.031573134	0.629987436
monocyte	ieu-b-31	pulmonary embolism	rs3795503	-0.014958816	0.031570756	0.635629179
monocyte	ieu-b-31	pulmonary embolism	rs3808609	-0.014294233	0.031532827	0.650323552
monocyte	ieu-b-31	pulmonary embolism	rs3856364	-0.015555362	0.031560805	0.622104719
monocyte	ieu-b-31	pulmonary embolism	rs3859570	-0.013726695	0.031566983	0.663676096
monocyte	ieu-b-31	pulmonary embolism	rs391023	-0.01308889	0.031848093	0.681088101
monocyte	ieu-b-31	pulmonary embolism	rs41268099	-0.015214032	0.031570153	0.629868543
monocyte	ieu-b-31	pulmonary embolism	rs41313381	-0.015623365	0.031555954	0.620529267
monocyte	ieu-b-31	pulmonary embolism	rs413141	-0.012377599	0.0315682	0.694991157
monocyte	ieu-b-31	pulmonary embolism	rs41381344	-0.017233842	0.031325014	0.582208052
monocyte	ieu-b-31	pulmonary embolism	rs41409548	-0.014824358	0.031605686	0.639040433
monocyte	ieu-b-31	pulmonary embolism	rs4142441	-0.016324117	0.031564384	0.605038281
monocyte	ieu-b-31	pulmonary embolism	rs41430449	-0.015355905	0.03157525	0.626735153
monocyte	ieu-b-31	pulmonary embolism	rs4269828	-0.014438747	0.031512264	0.646812682
monocyte	ieu-b-31	pulmonary embolism	rs4335411	-0.014503554	0.031539126	0.645617372
monocyte	ieu-b-31	pulmonary embolism	rs4365101	-0.015361538	0.031568387	0.626533711
monocyte	ieu-b-31	pulmonary embolism	rs4385425	-0.019198876	0.031681974	0.544523151
monocyte	ieu-b-31	pulmonary embolism	rs4411554	-0.014887442	0.031551821	0.63704029
monocyte	ieu-b-31	pulmonary embolism	rs445611	-0.016307736	0.031481625	0.604452632
monocyte	ieu-b-31	pulmonary embolism	rs4462758	-0.016942572	0.031566529	0.591456989
monocyte	ieu-b-31	pulmonary embolism	rs45577137	-0.016879809	0.031595097	0.593165767
monocyte	ieu-b-31	pulmonary embolism	rs4566648	-0.014789137	0.031578485	0.63954897
monocyte	ieu-b-31	pulmonary embolism	rs4577206	-0.015137611	0.031565978	0.631544108
monocyte	ieu-b-31	pulmonary embolism	rs458699	-0.015550147	0.031559399	0.622205972
monocyte	ieu-b-31	pulmonary embolism	rs4626924	-0.015425018	0.031573063	0.625160365
monocyte	ieu-b-31	pulmonary embolism	rs4658231	-0.015739588	0.031559391	0.617970221
monocyte	ieu-b-31	pulmonary embolism	rs4669869	-0.015206706	0.031579191	0.630131358
monocyte	ieu-b-31	pulmonary embolism	rs4712652	-0.013673835	0.031434123	0.663562616
monocyte	ieu-b-31	pulmonary embolism	rs4721764	-0.014617824	0.031540125	0.643029276
monocyte	ieu-b-31	pulmonary embolism	rs47341	-0.015409334	0.031606711	0.625880214
monocyte	ieu-b-31	pulmonary embolism	rs4737009	-0.014921164	0.031557665	0.636340054
monocyte	ieu-b-31	pulmonary embolism	rs475616	-0.013835774	0.031460114	0.660090813
monocyte	ieu-b-31	pulmonary embolism	rs4763944	-0.015229297	0.031563348	0.629451212
monocyte	ieu-b-31	pulmonary embolism	rs4810832	-0.013888249	0.031403923	0.658311442
monocyte	ieu-b-31	pulmonary embolism	rs4813619	-0.015445638	0.031570678	0.624671803
monocyte	ieu-b-31	pulmonary embolism	rs4865956	-0.014003333	0.031514518	0.656792845
monocyte	ieu-b-31	pulmonary embolism	rs4871844	-0.014648854	0.031549526	0.642423533
monocyte	ieu-b-31	pulmonary embolism	rs4886615	-0.015527348	0.03157201	0.622855524

monocyte	ieu-b-31	pulmonary embolism	rs4905043	-0.01636099	0.031542217	0.603969021
monocyte	ieu-b-31	pulmonary embolism	rs4907230	-0.013900387	0.03148978	0.658905091
monocyte	ieu-b-31	pulmonary embolism	rs4952782	-0.015122667	0.031573137	0.631958135
monocyte	ieu-b-31	pulmonary embolism	rs4970966	-0.006653664	0.03154976	0.832969765
monocyte	ieu-b-31	pulmonary embolism	rs4983387	-0.014371399	0.031503616	0.648258731
monocyte	ieu-b-31	pulmonary embolism	rs4987353	-0.014646851	0.031564987	0.642631923
monocyte	ieu-b-31	pulmonary embolism	rs55684236	-0.015682742	0.031552318	0.619161334
monocyte	ieu-b-31	pulmonary embolism	rs55860328	-0.015314351	0.031566069	0.627568303
monocyte	ieu-b-31	pulmonary embolism	rs56007794	-0.017640382	0.031577035	0.576403404
monocyte	ieu-b-31	pulmonary embolism	rs56058420	-0.015532892	0.031557019	0.622566169
monocyte	ieu-b-31	pulmonary embolism	rs56344893	-0.018755908	0.031590697	0.552701962
monocyte	ieu-b-31	pulmonary embolism	rs56795609	-0.014411633	0.031485534	0.647151912
monocyte	ieu-b-31	pulmonary embolism	rs56925909	-0.015639913	0.031554888	0.620147345
monocyte	ieu-b-31	pulmonary embolism	rs571497	-0.013793538	0.031510927	0.661576039
monocyte	ieu-b-31	pulmonary embolism	rs57221391	-0.015965748	0.03153092	0.61260948
monocyte	ieu-b-31	pulmonary embolism	rs573790	-0.013790704	0.031482913	0.661358934
monocyte	ieu-b-31	pulmonary embolism	rs58011825	-0.015372426	0.03156752	0.626279806
monocyte	ieu-b-31	pulmonary embolism	rs58814158	-0.015616855	0.031565223	0.620777486
monocyte	ieu-b-31	pulmonary embolism	rs59295685	-0.016358125	0.031527877	0.603867859
monocyte	ieu-b-31	pulmonary embolism	rs6044081	-0.016321881	0.031461026	0.603902418
monocyte	ieu-b-31	pulmonary embolism	rs6055955	-0.015925747	0.031599066	0.614265433
monocyte	ieu-b-31	pulmonary embolism	rs60695341	-0.014923032	0.031562235	0.636346657
monocyte	ieu-b-31	pulmonary embolism	rs60699901	-0.016450143	0.031506894	0.601592013
monocyte	ieu-b-31	pulmonary embolism	rs61165644	-0.01328888	0.031496092	0.673082279
monocyte	ieu-b-31	pulmonary embolism	rs6120950	-0.014688656	0.03155985	0.641629105
monocyte	ieu-b-31	pulmonary embolism	rs61985545	-0.016029701	0.031525919	0.61113007
monocyte	ieu-b-31	pulmonary embolism	rs62011334	-0.015068181	0.031557261	0.633015392
monocyte	ieu-b-31	pulmonary embolism	rs62018159	-0.015213814	0.03156329	0.629799005
monocyte	ieu-b-31	pulmonary embolism	rs62054807	-0.016406021	0.031523555	0.602759089
monocyte	ieu-b-31	pulmonary embolism	rs62057793	-0.016458152	0.031482251	0.601130432
monocyte	ieu-b-31	pulmonary embolism	rs62143205	-0.014148175	0.031612218	0.654475106
monocyte	ieu-b-31	pulmonary embolism	rs62176784	-0.015001543	0.031581589	0.63478046
monocyte	ieu-b-31	pulmonary embolism	rs62261974	-0.01528206	0.031564019	0.628271773
monocyte	ieu-b-31	pulmonary embolism	rs62454420	-0.015081509	0.031579069	0.632949688
monocyte	ieu-b-31	pulmonary embolism	rs62470670	-0.015701982	0.03156499	0.618872245
monocyte	ieu-b-31	pulmonary embolism	rs62491927	-0.014874061	0.031545393	0.637274483
monocyte	ieu-b-31	pulmonary embolism	rs62501136	-0.017626581	0.031393803	0.574479285
monocyte	ieu-b-31	pulmonary embolism	rs62502392	-0.0145357	0.03152802	0.644769417
monocyte	ieu-b-31	pulmonary embolism	rs62513343	-0.014764451	0.0315265	0.639556652
monocyte	ieu-b-31	pulmonary embolism	rs628615	-0.017166837	0.031300031	0.583375612
monocyte	ieu-b-31	pulmonary embolism	rs628965	-0.015701452	0.031554955	0.618772552
monocyte	ieu-b-31	pulmonary embolism	rs6429432	-0.012830465	0.031637873	0.685079651
monocyte	ieu-b-31	pulmonary embolism	rs6429438	-0.013247126	0.031373614	0.672851451
monocyte	ieu-b-31	pulmonary embolism	rs6434817	-0.016108314	0.031503954	0.609133395

monocyte	ieu-b-31	pulmonary embolism	rs644492	-0.014898786	0.031558377	0.636853681
monocyte	ieu-b-31	pulmonary embolism	rs6470759	-0.015445515	0.0315727	0.624696744
monocyte	ieu-b-31	pulmonary embolism	rs6493575	-0.015901539	0.031539566	0.614136661
monocyte	ieu-b-31	pulmonary embolism	rs6512627	-0.014975492	0.03162303	0.635811686
monocyte	ieu-b-31	pulmonary embolism	rs6545873	-0.013847763	0.0314757	0.65997265
monocyte	ieu-b-31	pulmonary embolism	rs6579771	-0.016270206	0.031590198	0.606525274
monocyte	ieu-b-31	pulmonary embolism	rs6581124	-0.016227912	0.031502361	0.606459941
monocyte	ieu-b-31	pulmonary embolism	rs6591578	-0.014281344	0.031558156	0.650879634
monocyte	ieu-b-31	pulmonary embolism	rs662333	-0.015442104	0.031562284	0.62465896
monocyte	ieu-b-31	pulmonary embolism	rs663202	-0.014941415	0.031557848	0.63588421
monocyte	ieu-b-31	pulmonary embolism	rs6664626	-0.013815886	0.031437185	0.660316709
monocyte	ieu-b-31	pulmonary embolism	rs6687430	-0.015289599	0.031564128	0.628103475
monocyte	ieu-b-31	pulmonary embolism	rs6696074	-0.016701164	0.03151634	0.596166846
monocyte	ieu-b-31	pulmonary embolism	rs67224956	-0.017296327	0.031477178	0.582670691
monocyte	ieu-b-31	pulmonary embolism	rs6736362	-0.016467846	0.031550946	0.601708893
monocyte	ieu-b-31	pulmonary embolism	rs6740847	-0.007267666	0.032424396	0.822646878
monocyte	ieu-b-31	pulmonary embolism	rs6745920	-0.014790137	0.031544092	0.639161223
monocyte	ieu-b-31	pulmonary embolism	rs6753534	-0.015845203	0.031548012	0.615486897
monocyte	ieu-b-31	pulmonary embolism	rs6772164	-0.015309245	0.031562619	0.627645454
monocyte	ieu-b-31	pulmonary embolism	rs6796	-0.017126554	0.03163845	0.588286425
monocyte	ieu-b-31	pulmonary embolism	rs6799804	-0.016290891	0.031547848	0.605584309
monocyte	ieu-b-31	pulmonary embolism	rs6800122	-0.015853673	0.03157463	0.615596046
monocyte	ieu-b-31	pulmonary embolism	rs6856799	-0.014960527	0.031549849	0.635366599
monocyte	ieu-b-31	pulmonary embolism	rs6869021	-0.015269707	0.031569366	0.628607686
monocyte	ieu-b-31	pulmonary embolism	rs6883116	-0.014337586	0.031545086	0.649461134
monocyte	ieu-b-31	pulmonary embolism	rs695113	-0.012985497	0.031446515	0.679651188
monocyte	ieu-b-31	pulmonary embolism	rs701905	-0.016030103	0.031558321	0.611487222
monocyte	ieu-b-31	pulmonary embolism	rs7030655	-0.015425489	0.031573922	0.6251592
monocyte	ieu-b-31	pulmonary embolism	rs706819	-0.015184347	0.031569951	0.63053449
monocyte	ieu-b-31	pulmonary embolism	rs707793	-0.014616077	0.031602248	0.643721953
monocyte	ieu-b-31	pulmonary embolism	rs7095778	-0.015599734	0.031554065	0.621037043
monocyte	ieu-b-31	pulmonary embolism	rs7097656	-0.015593658	0.03156924	0.621340772
monocyte	ieu-b-31	pulmonary embolism	rs7111769	-0.014949561	0.031543258	0.63554384
monocyte	ieu-b-31	pulmonary embolism	rs71331613	-0.016768574	0.031542717	0.594992848
monocyte	ieu-b-31	pulmonary embolism	rs71368117	-0.014900324	0.031552761	0.636758927
monocyte	ieu-b-31	pulmonary embolism	rs7180079	-0.01584861	0.031639969	0.616438182
monocyte	ieu-b-31	pulmonary embolism	rs7180804	-0.013589013	0.031783341	0.668977737
monocyte	ieu-b-31	pulmonary embolism	rs7185007	-0.013456821	0.031417575	0.668417112
monocyte	ieu-b-31	pulmonary embolism	rs718515	-0.016265781	0.031555687	0.606229698
monocyte	ieu-b-31	pulmonary embolism	rs7196129	-0.012511275	0.0314624	0.6908823
monocyte	ieu-b-31	pulmonary embolism	rs723585	-0.014294807	0.031534387	0.650326578
monocyte	ieu-b-31	pulmonary embolism	rs7249692	-0.016066316	0.031541458	0.610491922
monocyte	ieu-b-31	pulmonary embolism	rs72673751	-0.015974482	0.031531593	0.612422673
monocyte	ieu-b-31	pulmonary embolism	rs72675573	-0.015157255	0.031567421	0.631117196

monocyte	ieu-b-31	pulmonary embolism	rs72720206	-0.016500088	0.031515719	0.600590822
monocyte	ieu-b-31	pulmonary embolism	rs72743796	-0.015511883	0.031557618	0.623043413
monocyte	ieu-b-31	pulmonary embolism	rs72759267	-0.015406169	0.031574738	0.625601523
monocyte	ieu-b-31	pulmonary embolism	rs72790862	-0.013564961	0.031489691	0.666632212
monocyte	ieu-b-31	pulmonary embolism	rs72805138	-0.01417623	0.031515414	0.652841
monocyte	ieu-b-31	pulmonary embolism	rs72836307	-0.016181705	0.031586793	0.60844571
monocyte	ieu-b-31	pulmonary embolism	rs72970406	-0.015096122	0.031555707	0.632368439
monocyte	ieu-b-31	pulmonary embolism	rs72987040	-0.01452547	0.031555173	0.645286718
monocyte	ieu-b-31	pulmonary embolism	rs73058593	-0.017004205	0.031579579	0.590262463
monocyte	ieu-b-31	pulmonary embolism	rs73067080	-0.015858798	0.031553681	0.615247359
monocyte	ieu-b-31	pulmonary embolism	rs7308348	-0.0165596	0.031443652	0.59844106
monocyte	ieu-b-31	pulmonary embolism	rs73142138	-0.014966197	0.031557781	0.635323473
monocyte	ieu-b-31	pulmonary embolism	rs731707	-0.012962733	0.031652018	0.682144207
monocyte	ieu-b-31	pulmonary embolism	rs73201961	-0.015871372	0.031557467	0.615009577
monocyte	ieu-b-31	pulmonary embolism	rs73203055	-0.01430587	0.031531095	0.650039921
monocyte	ieu-b-31	pulmonary embolism	rs73217470	-0.014480795	0.031555617	0.646307754
monocyte	ieu-b-31	pulmonary embolism	rs73467599	-0.015113258	0.031572113	0.632159114
monocyte	ieu-b-31	pulmonary embolism	rs73784287	-0.015985428	0.031522064	0.61207154
monocyte	ieu-b-31	pulmonary embolism	rs73809166	-0.015768713	0.031538004	0.617081522
monocyte	ieu-b-31	pulmonary embolism	rs739241	-0.017031723	0.031480243	0.588487623
monocyte	ieu-b-31	pulmonary embolism	rs74233809	-0.013923245	0.031487373	0.658355321
monocyte	ieu-b-31	pulmonary embolism	rs74259566	-0.014860293	0.031553183	0.637669164
monocyte	ieu-b-31	pulmonary embolism	rs745570	-0.016925645	0.031440009	0.590337284
monocyte	ieu-b-31	pulmonary embolism	rs745822	-0.01569799	0.031562373	0.618932357
monocyte	ieu-b-31	pulmonary embolism	rs74735005	-0.016000263	0.031539033	0.611932963
monocyte	ieu-b-31	pulmonary embolism	rs74765249	-0.015001749	0.031556807	0.634509963
monocyte	ieu-b-31	pulmonary embolism	rs7516138	-0.014527086	0.031573168	0.645438286
monocyte	ieu-b-31	pulmonary embolism	rs7522307	-0.01609025	0.031542008	0.609966483
monocyte	ieu-b-31	pulmonary embolism	rs7524046	-0.016195571	0.031499595	0.607145926
monocyte	ieu-b-31	pulmonary embolism	rs754388	-0.01348442	0.031508682	0.668681073
monocyte	ieu-b-31	pulmonary embolism	rs75475627	-0.01542476	0.031565977	0.62508849
monocyte	ieu-b-31	pulmonary embolism	rs7569084	-0.015472639	0.031594818	0.6243315
monocyte	ieu-b-31	pulmonary embolism	rs7572278	-0.015833218	0.03156584	0.615953536
monocyte	ieu-b-31	pulmonary embolism	rs7574456	-0.016161721	0.031589995	0.608924798
monocyte	ieu-b-31	pulmonary embolism	rs7593080	-0.016065797	0.031513291	0.610184437
monocyte	ieu-b-31	pulmonary embolism	rs761841	-0.015971524	0.031535361	0.612530968
monocyte	ieu-b-31	pulmonary embolism	rs7620353	-0.014823786	0.031540027	0.638355622
monocyte	ieu-b-31	pulmonary embolism	rs7633965	-0.014798049	0.031533369	0.63886793
monocyte	ieu-b-31	pulmonary embolism	rs76427287	-0.01622206	0.031565574	0.607310575
monocyte	ieu-b-31	pulmonary embolism	rs76428106	-0.005591858	0.032289739	0.862511863
monocyte	ieu-b-31	pulmonary embolism	rs77733744	-0.015532261	0.031558886	0.622600872
monocyte	ieu-b-31	pulmonary embolism	rs7776054	-0.011749714	0.031532561	0.709430065
monocyte	ieu-b-31	pulmonary embolism	rs7785014	-0.017385212	0.031510993	0.581140936
monocyte	ieu-b-31	pulmonary embolism	rs7786376	-0.015967072	0.031522063	0.612480147

monocyte	ieu-b-31	pulmonary embolism	rs7787179	-0.016082346	0.031550831	0.610241938
monocyte	ieu-b-31	pulmonary embolism	rs7803075	-0.015766922	0.031556253	0.61732511
monocyte	ieu-b-31	pulmonary embolism	rs78218855	-0.014958347	0.03155401	0.635460436
monocyte	ieu-b-31	pulmonary embolism	rs7824937	-0.014958711	0.031569046	0.635613233
monocyte	ieu-b-31	pulmonary embolism	rs7826487	-0.015594473	0.031579746	0.621438616
monocyte	ieu-b-31	pulmonary embolism	rs7836456	-0.014603067	0.031545942	0.643425806
monocyte	ieu-b-31	pulmonary embolism	rs7836786	-0.015986212	0.03152291	0.612063637
monocyte	ieu-b-31	pulmonary embolism	rs7861055	-0.016147617	0.031532795	0.608588292
monocyte	ieu-b-31	pulmonary embolism	rs7919533	-0.016202422	0.031559737	0.607679222
monocyte	ieu-b-31	pulmonary embolism	rs79335847	-0.015750267	0.031548904	0.617614957
monocyte	ieu-b-31	pulmonary embolism	rs79407714	-0.015319327	0.03157277	0.627529559
monocyte	ieu-b-31	pulmonary embolism	rs79446921	-0.01504851	0.031557943	0.633466576
monocyte	ieu-b-31	pulmonary embolism	rs7971382	-0.015135355	0.031560147	0.631531933
monocyte	ieu-b-31	pulmonary embolism	rs7975680	-0.015494022	0.031562865	0.623501405
monocyte	ieu-b-31	pulmonary embolism	rs798563	-0.013465495	0.031444721	0.668485485
monocyte	ieu-b-31	pulmonary embolism	rs79894332	-0.012606135	0.03152639	0.689259801
monocyte	ieu-b-31	pulmonary embolism	rs8049116	-0.01622987	0.031534588	0.606784458
monocyte	ieu-b-31	pulmonary embolism	rs8077619	-0.014429505	0.031511626	0.647016734
monocyte	ieu-b-31	pulmonary embolism	rs8207	-0.01594233	0.031533865	0.613164027
monocyte	ieu-b-31	pulmonary embolism	rs869785	-0.015757892	0.031563987	0.61761283
monocyte	ieu-b-31	pulmonary embolism	rs871134	-0.020832395	0.031433751	0.507497274
monocyte	ieu-b-31	pulmonary embolism	rs875890	-0.015793972	0.031559968	0.616763033
monocyte	ieu-b-31	pulmonary embolism	rs907612	-0.015076716	0.031613299	0.63342582
monocyte	ieu-b-31	pulmonary embolism	rs915125	-0.014727483	0.031606767	0.641244274
monocyte	ieu-b-31	pulmonary embolism	rs919217	-0.015678796	0.031551425	0.619239599
monocyte	ieu-b-31	pulmonary embolism	rs9295484	-0.016161158	0.031423239	0.607038115
monocyte	ieu-b-31	pulmonary embolism	rs932641	-0.014761618	0.031534826	0.639709315
monocyte	ieu-b-31	pulmonary embolism	rs932905	-0.015231578	0.031578617	0.629565607
monocyte	ieu-b-31	pulmonary embolism	rs9375150	-0.015294402	0.03157521	0.628116174
monocyte	ieu-b-31	pulmonary embolism	rs9379077	-0.013753259	0.031546712	0.662862205
monocyte	ieu-b-31	pulmonary embolism	rs9390460	-0.016776909	0.031413923	0.593300261
monocyte	ieu-b-31	pulmonary embolism	rs9410425	-0.014834999	0.031596873	0.638706242
monocyte	ieu-b-31	pulmonary embolism	rs9480737	-0.013914139	0.031536689	0.659064729
monocyte	ieu-b-31	pulmonary embolism	rs9494573	-0.015196977	0.031569252	0.630242617
monocyte	ieu-b-31	pulmonary embolism	rs9526475	-0.015272215	0.031563151	0.628483678
monocyte	ieu-b-31	pulmonary embolism	rs9532580	-0.014545745	0.031581976	0.645106362
monocyte	ieu-b-31	pulmonary embolism	rs954954	-0.014665117	0.031564601	0.642213321
monocyte	ieu-b-31	pulmonary embolism	rs9555596	-0.017569762	0.031485873	0.57683039
monocyte	ieu-b-31	pulmonary embolism	rs9564870	-0.014870967	0.031549668	0.637390114
monocyte	ieu-b-31	pulmonary embolism	rs9625746	-0.018280725	0.031352606	0.559847008
monocyte	ieu-b-31	pulmonary embolism	rs9734613	-0.015569357	0.031556723	0.621746318
monocyte	ieu-b-31	pulmonary embolism	rs9783374	-0.014586627	0.031561783	0.64396584
monocyte	ieu-b-31	pulmonary embolism	rs9787298	-0.014335743	0.031515118	0.649192236
monocyte	ieu-b-31	pulmonary embolism	rs9809116	-0.015407939	0.031571076	0.625521703

monocyte	ieu-b-31	pulmonary embolism	rs9815073	-0.015101987	0.031578332	0.63248017
monocyte	ieu-b-31	pulmonary embolism	rs9898876	-0.015051661	0.031558624	0.633402811
monocyte	ieu-b-31	pulmonary embolism	rs9915112	-0.013975177	0.031551715	0.657816931
monocyte	ieu-b-31	pulmonary embolism	rs9943753	-0.015529935	0.031563519	0.62270404
monocyte	ieu-b-31	pulmonary embolism	rs9963693	-0.015626979	0.031558503	0.620476667
monocyte	ieu-b-31	pulmonary embolism	rs9992013	-0.01570869	0.031563798	0.618709181
monocyte	ieu-b-31	pulmonary embolism	All	-0.015313363	0.031522315	0.627112977
lymphocyte	ieu-b-32	pulmonary embolism	rs1000329	-0.100479192	0.036801325	0.006327393
lymphocyte	ieu-b-32	pulmonary embolism	rs1002607	-0.099146591	0.036192071	0.006154093
lymphocyte	ieu-b-32	pulmonary embolism	rs10027415	-0.101073591	0.0361898	0.005224186
lymphocyte	ieu-b-32	pulmonary embolism	rs10076701	-0.102808309	0.036149736	0.004455701
lymphocyte	ieu-b-32	pulmonary embolism	rs10109370	-0.098770513	0.036200876	0.006364216
lymphocyte	ieu-b-32	pulmonary embolism	rs10138752	-0.098903919	0.036215927	0.006315187
lymphocyte	ieu-b-32	pulmonary embolism	rs10145277	-0.099966147	0.036215755	0.005774929
lymphocyte	ieu-b-32	pulmonary embolism	rs10174056	-0.098958039	0.036187196	0.006245333
lymphocyte	ieu-b-32	pulmonary embolism	rs1017875	-0.098566533	0.0362696	0.006575581
lymphocyte	ieu-b-32	pulmonary embolism	rs10179705	-0.10072484	0.036224246	0.005425991
lymphocyte	ieu-b-32	pulmonary embolism	rs10191329	-0.100799464	0.036168392	0.005320685
lymphocyte	ieu-b-32	pulmonary embolism	rs10206961	-0.100580328	0.03618236	0.005438959
lymphocyte	ieu-b-32	pulmonary embolism	rs10212190	-0.099457913	0.036200764	0.006007016
lymphocyte	ieu-b-32	pulmonary embolism	rs10230506	-0.102498476	0.036077793	0.004496658
lymphocyte	ieu-b-32	pulmonary embolism	rs10237524	-0.099336447	0.036223615	0.00610084
lymphocyte	ieu-b-32	pulmonary embolism	rs10245472	-0.10024795	0.036208773	0.005629607
lymphocyte	ieu-b-32	pulmonary embolism	rs1043996	-0.100389414	0.03619876	0.00554938
lymphocyte	ieu-b-32	pulmonary embolism	rs10445308	-0.101269395	0.036271535	0.005238661
lymphocyte	ieu-b-32	pulmonary embolism	rs10466905	-0.091318771	0.036259107	0.011785394
lymphocyte	ieu-b-32	pulmonary embolism	rs10480003	-0.099238828	0.036198805	0.006116036
lymphocyte	ieu-b-32	pulmonary embolism	rs10494783	-0.096792169	0.036194705	0.007490599
lymphocyte	ieu-b-32	pulmonary embolism	rs10503147	-0.099970232	0.036206866	0.005760969
lymphocyte	ieu-b-32	pulmonary embolism	rs1050979	-0.101741222	0.036223174	0.004973688
lymphocyte	ieu-b-32	pulmonary embolism	rs10748526	-0.100383256	0.036207178	0.005563292
lymphocyte	ieu-b-32	pulmonary embolism	rs10761129	-0.10038543	0.03619354	0.005544437
lymphocyte	ieu-b-32	pulmonary embolism	rs10762264	-0.100834199	0.03621143	0.005359447
lymphocyte	ieu-b-32	pulmonary embolism	rs10774624	-0.117564655	0.036697897	0.001357302
lymphocyte	ieu-b-32	pulmonary embolism	rs10783340	-0.100892421	0.036162168	0.005270817
lymphocyte	ieu-b-32	pulmonary embolism	rs10794175	-0.101815989	0.03619492	0.0049083
lymphocyte	ieu-b-32	pulmonary embolism	rs10814138	-0.10113631	0.036155474	0.005153783
lymphocyte	ieu-b-32	pulmonary embolism	rs10823631	-0.101038552	0.036198122	0.005250246
lymphocyte	ieu-b-32	pulmonary embolism	rs10828725	-0.09776001	0.03626422	0.007022606
lymphocyte	ieu-b-32	pulmonary embolism	rs10838634	-0.098942712	0.036189055	0.00625604
lymphocyte	ieu-b-32	pulmonary embolism	rs10846577	-0.098106277	0.0361228	0.006609387
lymphocyte	ieu-b-32	pulmonary embolism	rs10865331	-0.099387946	0.036201788	0.006043943
lymphocyte	ieu-b-32	pulmonary embolism	rs10875371	-0.100832892	0.03622142	0.005372751
lymphocyte	ieu-b-32	pulmonary embolism	rs10932018	-0.101267629	0.036136789	0.005073311

lymphocyte	ieu-b-32	pulmonary embolism	rs10956401	-0.097226111	0.036182868	0.007208058
lymphocyte	ieu-b-32	pulmonary embolism	rs10965124	-0.099716803	0.036214208	0.00589569
lymphocyte	ieu-b-32	pulmonary embolism	rs10985915	-0.100108805	0.036209932	0.0056979
lymphocyte	ieu-b-32	pulmonary embolism	rs10986338	-0.100105342	0.036205478	0.005693631
lymphocyte	ieu-b-32	pulmonary embolism	rs11033388	-0.09864536	0.036206036	0.006438807
lymphocyte	ieu-b-32	pulmonary embolism	rs11052877	-0.107091605	0.036585413	0.003420654
lymphocyte	ieu-b-32	pulmonary embolism	rs11073903	-0.098266933	0.036118003	0.006514013
lymphocyte	ieu-b-32	pulmonary embolism	rs11084096	-0.099469555	0.036217525	0.00602444
lymphocyte	ieu-b-32	pulmonary embolism	rs11134475	-0.097908767	0.036088007	0.006666593
lymphocyte	ieu-b-32	pulmonary embolism	rs11160706	-0.097286209	0.036212907	0.007220355
lymphocyte	ieu-b-32	pulmonary embolism	rs111626441	-0.099957647	0.036230857	0.005799463
lymphocyte	ieu-b-32	pulmonary embolism	rs11168249	-0.100554705	0.036226391	0.005507781
lymphocyte	ieu-b-32	pulmonary embolism	rs1118866	-0.103120804	0.035935728	0.004110044
lymphocyte	ieu-b-32	pulmonary embolism	rs11218725	-0.099367729	0.036293757	0.006183779
lymphocyte	ieu-b-32	pulmonary embolism	rs11224302	-0.104772009	0.035921833	0.003537938
lymphocyte	ieu-b-32	pulmonary embolism	rs11231804	-0.100779168	0.03621637	0.00539092
lymphocyte	ieu-b-32	pulmonary embolism	rs11246147	-0.100303258	0.036201688	0.005593954
lymphocyte	ieu-b-32	pulmonary embolism	rs11247712	-0.097395241	0.036138481	0.007037674
lymphocyte	ieu-b-32	pulmonary embolism	rs112992671	-0.100355049	0.036190512	0.005554797
lymphocyte	ieu-b-32	pulmonary embolism	rs113116201	-0.100380397	0.036185654	0.005536513
lymphocyte	ieu-b-32	pulmonary embolism	rs113142693	-0.101311929	0.036159674	0.005081944
lymphocyte	ieu-b-32	pulmonary embolism	rs1132352	-0.098562098	0.036234436	0.006525777
lymphocyte	ieu-b-32	pulmonary embolism	rs113513990	-0.096732575	0.036152756	0.00745817
lymphocyte	ieu-b-32	pulmonary embolism	rs113542380	-0.098321335	0.036231108	0.006653097
lymphocyte	ieu-b-32	pulmonary embolism	rs11505019	-0.100711666	0.036192569	0.005391504
lymphocyte	ieu-b-32	pulmonary embolism	rs11567701	-0.094604396	0.036374763	0.009299986
lymphocyte	ieu-b-32	pulmonary embolism	rs11652760	-0.098854563	0.036158445	0.006258385
lymphocyte	ieu-b-32	pulmonary embolism	rs11670621	-0.098843174	0.036151659	0.006254618
lymphocyte	ieu-b-32	pulmonary embolism	rs11676298	-0.101571646	0.036208072	0.005028209
lymphocyte	ieu-b-32	pulmonary embolism	rs11684463	-0.099676104	0.036360704	0.006119402
lymphocyte	ieu-b-32	pulmonary embolism	rs116876036	-0.099050325	0.036157316	0.006154639
lymphocyte	ieu-b-32	pulmonary embolism	rs11688289	-0.099673995	0.036204782	0.005904079
lymphocyte	ieu-b-32	pulmonary embolism	rs11688303	-0.099306853	0.036184833	0.006061551
lymphocyte	ieu-b-32	pulmonary embolism	rs11706384	-0.101600157	0.036212912	0.005021778
lymphocyte	ieu-b-32	pulmonary embolism	rs11712335	-0.101304817	0.036143994	0.005065921
lymphocyte	ieu-b-32	pulmonary embolism	rs11742873	-0.099694066	0.036208495	0.005899175
lymphocyte	ieu-b-32	pulmonary embolism	rs117556162	-0.098027123	0.036174788	0.006731996
lymphocyte	ieu-b-32	pulmonary embolism	rs11761199	-0.101111995	0.036136949	0.005141653
lymphocyte	ieu-b-32	pulmonary embolism	rs1177268	-0.100938472	0.036181639	0.00527454
lymphocyte	ieu-b-32	pulmonary embolism	rs117934175	-0.10177915	0.036179575	0.004905632
lymphocyte	ieu-b-32	pulmonary embolism	rs11800059	-0.100057321	0.036259638	0.00578955
lymphocyte	ieu-b-32	pulmonary embolism	rs12021809	-0.100977949	0.03620656	0.005288075
lymphocyte	ieu-b-32	pulmonary embolism	rs12038019	-0.099702176	0.03621459	0.005903492
lymphocyte	ieu-b-32	pulmonary embolism	rs12045893	-0.099536428	0.036252592	0.00603937

lymphocyte	ieu-b-32	pulmonary embolism	rs12059564	-0.100993183	0.036160198	0.005223174
lymphocyte	ieu-b-32	pulmonary embolism	rs12088882	-0.099969245	0.036307071	0.005897306
lymphocyte	ieu-b-32	pulmonary embolism	rs12127284	-0.099193295	0.03620965	0.006154844
lymphocyte	ieu-b-32	pulmonary embolism	rs12133917	-0.099181338	0.036205874	0.006155678
lymphocyte	ieu-b-32	pulmonary embolism	rs12142033	-0.098879308	0.036172399	0.006265432
lymphocyte	ieu-b-32	pulmonary embolism	rs12190136	-0.101207861	0.036141777	0.005105495
lymphocyte	ieu-b-32	pulmonary embolism	rs12330493	-0.10101728	0.036208899	0.0052733
lymphocyte	ieu-b-32	pulmonary embolism	rs12463256	-0.099276442	0.036192876	0.006088384
lymphocyte	ieu-b-32	pulmonary embolism	rs12526696	-0.097674715	0.036213107	0.006992106
lymphocyte	ieu-b-32	pulmonary embolism	rs12542907	-0.100021447	0.036206047	0.005734968
lymphocyte	ieu-b-32	pulmonary embolism	rs12567173	-0.098462076	0.036128971	0.00642442
lymphocyte	ieu-b-32	pulmonary embolism	rs12586002	-0.100277728	0.036208996	0.005615709
lymphocyte	ieu-b-32	pulmonary embolism	rs12598978	-0.092305475	0.036375186	0.01116172
lymphocyte	ieu-b-32	pulmonary embolism	rs1260326	-0.099342675	0.036243148	0.006125145
lymphocyte	ieu-b-32	pulmonary embolism	rs12627489	-0.100586981	0.036195746	0.005453115
lymphocyte	ieu-b-32	pulmonary embolism	rs12636926	-0.099487412	0.036203118	0.005995373
lymphocyte	ieu-b-32	pulmonary embolism	rs12640189	-0.100058692	0.036210974	0.005723513
lymphocyte	ieu-b-32	pulmonary embolism	rs1264593	-0.10198099	0.036103925	0.004733213
lymphocyte	ieu-b-32	pulmonary embolism	rs12692735	-0.098278669	0.03610438	0.006487429
lymphocyte	ieu-b-32	pulmonary embolism	rs12745411	-0.101492627	0.036170756	0.005017151
lymphocyte	ieu-b-32	pulmonary embolism	rs12811832	-0.101815732	0.036129172	0.004830829
lymphocyte	ieu-b-32	pulmonary embolism	rs1291414	-0.099179567	0.036179919	0.00611989
lymphocyte	ieu-b-32	pulmonary embolism	rs1292069	-0.099022759	0.03623637	0.006281947
lymphocyte	ieu-b-32	pulmonary embolism	rs1297265	-0.100679189	0.036183894	0.005395333
lymphocyte	ieu-b-32	pulmonary embolism	rs13040989	-0.101676254	0.036168695	0.00493621
lymphocyte	ieu-b-32	pulmonary embolism	rs13105987	-0.093223727	0.03624605	0.010112186
lymphocyte	ieu-b-32	pulmonary embolism	rs13133642	-0.099238702	0.03661933	0.006728145
lymphocyte	ieu-b-32	pulmonary embolism	rs1317575	-0.099482246	0.036204045	0.005999269
lymphocyte	ieu-b-32	pulmonary embolism	rs13199519	-0.099920881	0.036212542	0.005792722
lymphocyte	ieu-b-32	pulmonary embolism	rs1320562	-0.099770679	0.036207851	0.005860277
lymphocyte	ieu-b-32	pulmonary embolism	rs13262073	-0.100195985	0.036203567	0.005647554
lymphocyte	ieu-b-32	pulmonary embolism	rs1344603	-0.099007987	0.036263533	0.00632891
lymphocyte	ieu-b-32	pulmonary embolism	rs1371076	-0.100167619	0.036210692	0.005670604
lymphocyte	ieu-b-32	pulmonary embolism	rs1384804	-0.103223091	0.036358014	0.004524467
lymphocyte	ieu-b-32	pulmonary embolism	rs1399180	-0.098846825	0.036215328	0.006344593
lymphocyte	ieu-b-32	pulmonary embolism	rs139974673	-0.098752739	0.036078032	0.006196488
lymphocyte	ieu-b-32	pulmonary embolism	rs140763487	-0.1000824	0.03619348	0.005688699
lymphocyte	ieu-b-32	pulmonary embolism	rs141801008	-0.098763123	0.036173817	0.006328866
lymphocyte	ieu-b-32	pulmonary embolism	rs1431545	-0.100814086	0.03622039	0.005380044
lymphocyte	ieu-b-32	pulmonary embolism	rs143875179	-0.10026235	0.036203613	0.005615934
lymphocyte	ieu-b-32	pulmonary embolism	rs144520705	-0.099424421	0.036145955	0.005947941
lymphocyte	ieu-b-32	pulmonary embolism	rs1448318	-0.100899417	0.036208527	0.005326084
lymphocyte	ieu-b-32	pulmonary embolism	rs1456059	-0.099530233	0.036203219	0.005973919
lymphocyte	ieu-b-32	pulmonary embolism	rs145719494	-0.099198893	0.036190808	0.006125309

lymphocyte	ieu-b-32	pulmonary embolism	rs145947882	-0.101403422	0.036125622	0.00500104
lymphocyte	ieu-b-32	pulmonary embolism	rs147700878	-0.099124902	0.036144454	0.006097993
lymphocyte	ieu-b-32	pulmonary embolism	rs148601577	-0.098415677	0.036129552	0.006450312
lymphocyte	ieu-b-32	pulmonary embolism	rs1491664	-0.100065823	0.036209186	0.005717668
lymphocyte	ieu-b-32	pulmonary embolism	rs1491765	-0.100886374	0.036202551	0.005324445
lymphocyte	ieu-b-32	pulmonary embolism	rs1493694	-0.100798335	0.036190997	0.005349849
lymphocyte	ieu-b-32	pulmonary embolism	rs149453951	-0.09946208	0.036208046	0.006015034
lymphocyte	ieu-b-32	pulmonary embolism	rs149984502	-0.100671897	0.036186036	0.005401425
lymphocyte	ieu-b-32	pulmonary embolism	rs1523178	-0.098134774	0.036187384	0.006690877
lymphocyte	ieu-b-32	pulmonary embolism	rs1533065	-0.09957284	0.036215975	0.005970127
lymphocyte	ieu-b-32	pulmonary embolism	rs1533298	-0.100238376	0.036255669	0.005696395
lymphocyte	ieu-b-32	pulmonary embolism	rs1611236	-0.102020872	0.036297653	0.004943784
lymphocyte	ieu-b-32	pulmonary embolism	rs1637366	-0.100928082	0.036171936	0.005267034
lymphocyte	ieu-b-32	pulmonary embolism	rs1677490	-0.102754086	0.035893039	0.004199343
lymphocyte	ieu-b-32	pulmonary embolism	rs16986308	-0.097039369	0.036181599	0.007318152
lymphocyte	ieu-b-32	pulmonary embolism	rs17005891	-0.099176369	0.036230246	0.006192854
lymphocyte	ieu-b-32	pulmonary embolism	rs17041439	-0.100292554	0.036211356	0.005611764
lymphocyte	ieu-b-32	pulmonary embolism	rs17055818	-0.096044969	0.03616172	0.007907724
lymphocyte	ieu-b-32	pulmonary embolism	rs17093026	-0.099191173	0.036188659	0.006126251
lymphocyte	ieu-b-32	pulmonary embolism	rs17138740	-0.099818198	0.036206793	0.005835371
lymphocyte	ieu-b-32	pulmonary embolism	rs17180155	-0.100268216	0.036200362	0.005608857
lymphocyte	ieu-b-32	pulmonary embolism	rs17229044	-0.100879028	0.036190705	0.005312807
lymphocyte	ieu-b-32	pulmonary embolism	rs17366643	-0.099590797	0.036213776	0.005958064
lymphocyte	ieu-b-32	pulmonary embolism	rs17507693	-0.099690004	0.036210794	0.005904349
lymphocyte	ieu-b-32	pulmonary embolism	rs175714	-0.100619902	0.03625809	0.005518437
lymphocyte	ieu-b-32	pulmonary embolism	rs17703261	-0.099535947	0.036182495	0.005942416
lymphocyte	ieu-b-32	pulmonary embolism	rs17710008	-0.102024809	0.036081281	0.004689276
lymphocyte	ieu-b-32	pulmonary embolism	rs1807669	-0.099852471	0.036250549	0.005878023
lymphocyte	ieu-b-32	pulmonary embolism	rs1822534	-0.102613998	0.036262824	0.004658731
lymphocyte	ieu-b-32	pulmonary embolism	rs187856913	-0.10122396	0.03623367	0.005211788
lymphocyte	ieu-b-32	pulmonary embolism	rs1883801	-0.101371874	0.036123454	0.005011994
lymphocyte	ieu-b-32	pulmonary embolism	rs1893033	-0.098946493	0.036145249	0.006191398
lymphocyte	ieu-b-32	pulmonary embolism	rs1893592	-0.100913042	0.036183356	0.005288162
lymphocyte	ieu-b-32	pulmonary embolism	rs1923403	-0.099895113	0.036203641	0.005793314
lymphocyte	ieu-b-32	pulmonary embolism	rs1966865	-0.102328585	0.036200421	0.004702689
lymphocyte	ieu-b-32	pulmonary embolism	rs1971429	-0.101064437	0.036156798	0.005187225
lymphocyte	ieu-b-32	pulmonary embolism	rs1976055	-0.100262941	0.036204531	0.005616863
lymphocyte	ieu-b-32	pulmonary embolism	rs1991431	-0.099352489	0.036247481	0.006126205
lymphocyte	ieu-b-32	pulmonary embolism	rs2055101	-0.09904568	0.036190669	0.006204491
lymphocyte	ieu-b-32	pulmonary embolism	rs2057340	-0.101192094	0.036143454	0.005114457
lymphocyte	ieu-b-32	pulmonary embolism	rs2065500	-0.103751127	0.036250783	0.004209261
lymphocyte	ieu-b-32	pulmonary embolism	rs2068485	-0.100453738	0.036193116	0.00551181
lymphocyte	ieu-b-32	pulmonary embolism	rs2089979	-0.097499153	0.036283031	0.007205742
lymphocyte	ieu-b-32	pulmonary embolism	rs2100139	-0.101708609	0.036131572	0.004878465

lymphocyte	ieu-b-32	pulmonary embolism	rs2145270	-0.099318466	0.036186861	0.006058466
lymphocyte	ieu-b-32	pulmonary embolism	rs2161118	-0.100111645	0.036206261	0.005691636
lymphocyte	ieu-b-32	pulmonary embolism	rs2180369	-0.100171659	0.036206337	0.005662883
lymphocyte	ieu-b-32	pulmonary embolism	rs2230604	-0.101884189	0.03618061	0.004862738
lymphocyte	ieu-b-32	pulmonary embolism	rs2270968	-0.099904471	0.036208404	0.005795166
lymphocyte	ieu-b-32	pulmonary embolism	rs2273215	-0.098806093	0.036184844	0.006322043
lymphocyte	ieu-b-32	pulmonary embolism	rs2275902	-0.099840407	0.036216054	0.005837009
lymphocyte	ieu-b-32	pulmonary embolism	rs2276566	-0.09906874	0.036185359	0.006184927
lymphocyte	ieu-b-32	pulmonary embolism	rs2282680	-0.101443659	0.036097419	0.004949926
lymphocyte	ieu-b-32	pulmonary embolism	rs2282718	-0.098732478	0.036198902	0.00638164
lymphocyte	ieu-b-32	pulmonary embolism	rs2291900	-0.097556372	0.036102719	0.006888459
lymphocyte	ieu-b-32	pulmonary embolism	rs2294421	-0.100635909	0.036178081	0.005407804
lymphocyte	ieu-b-32	pulmonary embolism	rs2294630	-0.099482805	0.036205992	0.006001692
lymphocyte	ieu-b-32	pulmonary embolism	rs2294752	-0.099224404	0.036180465	0.006097612
lymphocyte	ieu-b-32	pulmonary embolism	rs2294861	-0.099769191	0.036221162	0.005879181
lymphocyte	ieu-b-32	pulmonary embolism	rs2295797	-0.098928438	0.036207052	0.006289433
lymphocyte	ieu-b-32	pulmonary embolism	rs2297508	-0.102600908	0.036157757	0.004545546
lymphocyte	ieu-b-32	pulmonary embolism	rs2298552	-0.097857148	0.036136717	0.006769612
lymphocyte	ieu-b-32	pulmonary embolism	rs2298979	-0.101155917	0.036206184	0.005207898
lymphocyte	ieu-b-32	pulmonary embolism	rs2313532	-0.100872324	0.036187655	0.005311992
lymphocyte	ieu-b-32	pulmonary embolism	rs2322830	-0.100863012	0.036161876	0.005283703
lymphocyte	ieu-b-32	pulmonary embolism	rs2327531	-0.100857526	0.036159095	0.005282679
lymphocyte	ieu-b-32	pulmonary embolism	rs2369391	-0.098965035	0.036206546	0.006269434
lymphocyte	ieu-b-32	pulmonary embolism	rs2403246	-0.099838284	0.03620918	0.005828719
lymphocyte	ieu-b-32	pulmonary embolism	rs2412544	-0.099628591	0.036219489	0.005946988
lymphocyte	ieu-b-32	pulmonary embolism	rs2453582	-0.098041615	0.036143058	0.006675788
lymphocyte	ieu-b-32	pulmonary embolism	rs2453628	-0.101102028	0.036136502	0.005145495
lymphocyte	ieu-b-32	pulmonary embolism	rs2462017	-0.098151909	0.036102156	0.006553366
lymphocyte	ieu-b-32	pulmonary embolism	rs246524	-0.100006227	0.036209139	0.005746508
lymphocyte	ieu-b-32	pulmonary embolism	rs247557	-0.101391979	0.036105867	0.004982149
lymphocyte	ieu-b-32	pulmonary embolism	rs247826	-0.098886904	0.036307433	0.006457477
lymphocyte	ieu-b-32	pulmonary embolism	rs2489279	-0.101814821	0.036140727	0.004844783
lymphocyte	ieu-b-32	pulmonary embolism	rs251391	-0.098958524	0.036194383	0.006255387
lymphocyte	ieu-b-32	pulmonary embolism	rs251398	-0.100482138	0.036217542	0.005530274
lymphocyte	ieu-b-32	pulmonary embolism	rs2542573	-0.101632458	0.036132895	0.004912127
lymphocyte	ieu-b-32	pulmonary embolism	rs2548256	-0.101048961	0.036175585	0.005217463
lymphocyte	ieu-b-32	pulmonary embolism	rs2569693	-0.104334824	0.036290405	0.004040307
lymphocyte	ieu-b-32	pulmonary embolism	rs259981	-0.100354369	0.036259707	0.005646114
lymphocyte	ieu-b-32	pulmonary embolism	rs2665960	-0.098824321	0.036149999	0.006262145
lymphocyte	ieu-b-32	pulmonary embolism	rs2710804	-0.101836124	0.036093695	0.004780887
lymphocyte	ieu-b-32	pulmonary embolism	rs2727487	-0.099450181	0.036202578	0.006013452
lymphocyte	ieu-b-32	pulmonary embolism	rs2738783	-0.098776859	0.036153598	0.006292351
lymphocyte	ieu-b-32	pulmonary embolism	rs2755253	-0.097422393	0.036244494	0.007189806
lymphocyte	ieu-b-32	pulmonary embolism	rs2807303	-0.100108515	0.036234752	0.005731202

lymphocyte	ieu-b-32	pulmonary embolism	rs2847468	-0.099167155	0.036182941	0.006130552
lymphocyte	ieu-b-32	pulmonary embolism	rs28533432	-0.099285896	0.036245112	0.006157191
lymphocyte	ieu-b-32	pulmonary embolism	rs28539372	-0.101507316	0.036309541	0.005180186
lymphocyte	ieu-b-32	pulmonary embolism	rs2853951	-0.085702412	0.036540562	0.019006428
lymphocyte	ieu-b-32	pulmonary embolism	rs28588745	-0.101396264	0.036176019	0.005065188
lymphocyte	ieu-b-32	pulmonary embolism	rs28710104	-0.099492181	0.036207414	0.005998927
lymphocyte	ieu-b-32	pulmonary embolism	rs2908425	-0.099796126	0.036214039	0.005856125
lymphocyte	ieu-b-32	pulmonary embolism	rs2980821	-0.101610192	0.036117142	0.004902813
lymphocyte	ieu-b-32	pulmonary embolism	rs2980888	-0.100071724	0.036212777	0.005719615
lymphocyte	ieu-b-32	pulmonary embolism	rs2992263	-0.099415164	0.036224193	0.006061403
lymphocyte	ieu-b-32	pulmonary embolism	rs3012415	-0.099192978	0.036174817	0.006105797
lymphocyte	ieu-b-32	pulmonary embolism	rs304154	-0.098931198	0.036166492	0.006229732
lymphocyte	ieu-b-32	pulmonary embolism	rs309375	-0.102531727	0.036124087	0.004535165
lymphocyte	ieu-b-32	pulmonary embolism	rs3118469	-0.098604164	0.036260857	0.006542016
lymphocyte	ieu-b-32	pulmonary embolism	rs3134754	-0.096123877	0.036403746	0.008278522
lymphocyte	ieu-b-32	pulmonary embolism	rs3136685	-0.099996943	0.036216306	0.005760654
lymphocyte	ieu-b-32	pulmonary embolism	rs332507	-0.100721516	0.03618023	0.005371246
lymphocyte	ieu-b-32	pulmonary embolism	rs34025077	-0.099912183	0.036220609	0.005807891
lymphocyte	ieu-b-32	pulmonary embolism	rs34030812	-0.100291467	0.036223528	0.005628336
lymphocyte	ieu-b-32	pulmonary embolism	rs34140544	-0.098782407	0.036197772	0.00635337
lymphocyte	ieu-b-32	pulmonary embolism	rs34229224	-0.100743876	0.036147829	0.005319903
lymphocyte	ieu-b-32	pulmonary embolism	rs34323943	-0.098242813	0.03615727	0.006585728
lymphocyte	ieu-b-32	pulmonary embolism	rs34423734	-0.099090432	0.036150228	0.006123881
lymphocyte	ieu-b-32	pulmonary embolism	rs34499378	-0.099313078	0.036188731	0.006063834
lymphocyte	ieu-b-32	pulmonary embolism	rs34662054	-0.101398242	0.036138075	0.005018307
lymphocyte	ieu-b-32	pulmonary embolism	rs34664889	-0.100243533	0.03621763	0.005643428
lymphocyte	ieu-b-32	pulmonary embolism	rs34850939	-0.09774592	0.036378701	0.007211861
lymphocyte	ieu-b-32	pulmonary embolism	rs35010780	-0.099127359	0.036201806	0.00617787
lymphocyte	ieu-b-32	pulmonary embolism	rs35114152	-0.098822537	0.036235143	0.006386334
lymphocyte	ieu-b-32	pulmonary embolism	rs35121828	-0.09946597	0.036196518	0.005997042
lymphocyte	ieu-b-32	pulmonary embolism	rs35186877	-0.105216119	0.035978063	0.003450633
lymphocyte	ieu-b-32	pulmonary embolism	rs35345753	-0.100314613	0.036232922	0.005629701
lymphocyte	ieu-b-32	pulmonary embolism	rs35592432	-0.093485111	0.036085449	0.009579222
lymphocyte	ieu-b-32	pulmonary embolism	rs35993840	-0.099607137	0.03624554	0.005993785
lymphocyte	ieu-b-32	pulmonary embolism	rs360017	-0.098375071	0.036183992	0.006552998
lymphocyte	ieu-b-32	pulmonary embolism	rs36084354	-0.098353428	0.036295736	0.006732803
lymphocyte	ieu-b-32	pulmonary embolism	rs36108061	-0.100043264	0.036199214	0.005715249
lymphocyte	ieu-b-32	pulmonary embolism	rs3731211	-0.099700383	0.036326668	0.006059379
lymphocyte	ieu-b-32	pulmonary embolism	rs3735485	-0.103878543	0.03617674	0.004086348
lymphocyte	ieu-b-32	pulmonary embolism	rs3739756	-0.099703573	0.03619938	0.005881971
lymphocyte	ieu-b-32	pulmonary embolism	rs3744399	-0.098526791	0.036153896	0.006426151
lymphocyte	ieu-b-32	pulmonary embolism	rs3760456	-0.098669781	0.036178	0.006384682
lymphocyte	ieu-b-32	pulmonary embolism	rs3761846	-0.098057936	0.036191596	0.006740264
lymphocyte	ieu-b-32	pulmonary embolism	rs3797727	-0.101115964	0.036149744	0.005155688

lymphocyte	ieu-b-32	pulmonary embolism	rs3810818	-0.101449854	0.036165622	0.005029377
lymphocyte	ieu-b-32	pulmonary embolism	rs3812316	-0.099288617	0.036191285	0.006079916
lymphocyte	ieu-b-32	pulmonary embolism	rs3812849	-0.10282766	0.036295459	0.004610296
lymphocyte	ieu-b-32	pulmonary embolism	rs3822030	-0.099117415	0.036177026	0.006147866
lymphocyte	ieu-b-32	pulmonary embolism	rs3848132	-0.099212628	0.036184503	0.006109347
lymphocyte	ieu-b-32	pulmonary embolism	rs3851820	-0.101294971	0.036176157	0.005109503
lymphocyte	ieu-b-32	pulmonary embolism	rs3992621	-0.101628474	0.036044902	0.004809905
lymphocyte	ieu-b-32	pulmonary embolism	rs4129560	-0.099879703	0.036207408	0.005805962
lymphocyte	ieu-b-32	pulmonary embolism	rs41317270	-0.099579269	0.036203822	0.00595011
lymphocyte	ieu-b-32	pulmonary embolism	rs419470	-0.099274178	0.036201075	0.006101075
lymphocyte	ieu-b-32	pulmonary embolism	rs4319365	-0.099438794	0.03622876	0.00605575
lymphocyte	ieu-b-32	pulmonary embolism	rs4368798	-0.1008481	0.036174624	0.00530651
lymphocyte	ieu-b-32	pulmonary embolism	rs4406174	-0.100632234	0.036191718	0.00542699
lymphocyte	ieu-b-32	pulmonary embolism	rs4411786	-0.100123905	0.036348224	0.005876845
lymphocyte	ieu-b-32	pulmonary embolism	rs444613	-0.100183146	0.036215263	0.00566922
lymphocyte	ieu-b-32	pulmonary embolism	rs4497886	-0.098328465	0.036247515	0.006673843
lymphocyte	ieu-b-32	pulmonary embolism	rs4530278	-0.098459436	0.036181871	0.006503816
lymphocyte	ieu-b-32	pulmonary embolism	rs45528934	-0.098460515	0.036154046	0.006462141
lymphocyte	ieu-b-32	pulmonary embolism	rs4578474	-0.100214426	0.036203172	0.005638212
lymphocyte	ieu-b-32	pulmonary embolism	rs4619033	-0.100341261	0.036220972	0.005601275
lymphocyte	ieu-b-32	pulmonary embolism	rs464609	-0.101189451	0.036143124	0.00511521
lymphocyte	ieu-b-32	pulmonary embolism	rs4660128	-0.098650595	0.036169215	0.006382128
lymphocyte	ieu-b-32	pulmonary embolism	rs4685105	-0.099395486	0.036193885	0.006029081
lymphocyte	ieu-b-32	pulmonary embolism	rs4696314	-0.101480336	0.036146994	0.004993776
lymphocyte	ieu-b-32	pulmonary embolism	rs4714431	-0.099043655	0.036164456	0.006168231
lymphocyte	ieu-b-32	pulmonary embolism	rs4718976	-0.100554661	0.036217449	0.005496204
lymphocyte	ieu-b-32	pulmonary embolism	rs4737010	-0.099199085	0.036242609	0.006198636
lymphocyte	ieu-b-32	pulmonary embolism	rs4760278	-0.099131363	0.036184749	0.006151595
lymphocyte	ieu-b-32	pulmonary embolism	rs4789294	-0.099275683	0.036224087	0.006132737
lymphocyte	ieu-b-32	pulmonary embolism	rs4805881	-0.098787274	0.036181751	0.006327544
lymphocyte	ieu-b-32	pulmonary embolism	rs4812804	-0.098898572	0.036164806	0.006244409
lymphocyte	ieu-b-32	pulmonary embolism	rs4833031	-0.099685743	0.036205594	0.005899342
lymphocyte	ieu-b-32	pulmonary embolism	rs4849169	-0.098729717	0.036219268	0.006412849
lymphocyte	ieu-b-32	pulmonary embolism	rs4869745	-0.09994703	0.036208663	0.005774704
lymphocyte	ieu-b-32	pulmonary embolism	rs4880192	-0.097349868	0.036212732	0.007182172
lymphocyte	ieu-b-32	pulmonary embolism	rs4907236	-0.101286046	0.036076582	0.004992321
lymphocyte	ieu-b-32	pulmonary embolism	rs4925547	-0.09930051	0.036194445	0.006078268
lymphocyte	ieu-b-32	pulmonary embolism	rs4940572	-0.099081762	0.036210351	0.006213759
lymphocyte	ieu-b-32	pulmonary embolism	rs494091	-0.101418879	0.036249773	0.005145519
lymphocyte	ieu-b-32	pulmonary embolism	rs4948492	-0.10002599	0.036230558	0.005765674
lymphocyte	ieu-b-32	pulmonary embolism	rs4948822	-0.1009793	0.036157496	0.005226004
lymphocyte	ieu-b-32	pulmonary embolism	rs4972950	-0.098378875	0.036165717	0.006523758
lymphocyte	ieu-b-32	pulmonary embolism	rs4976642	-0.099607012	0.036236387	0.00598117
lymphocyte	ieu-b-32	pulmonary embolism	rs4987855	-0.099578383	0.036201109	0.005946812

lymphocyte	ieu-b-32	pulmonary embolism	rs501461	-0.100763375	0.036212175	0.005392811
lymphocyte	ieu-b-32	pulmonary embolism	rs5026470	-0.098719968	0.036322375	0.006570106
lymphocyte	ieu-b-32	pulmonary embolism	rs529002	-0.100293131	0.03620181	0.005598922
lymphocyte	ieu-b-32	pulmonary embolism	rs533483	-0.100534794	0.036190943	0.005471158
lymphocyte	ieu-b-32	pulmonary embolism	rs55938093	-0.10085664	0.036200991	0.005335978
lymphocyte	ieu-b-32	pulmonary embolism	rs55977949	-0.100753712	0.036209229	0.005393483
lymphocyte	ieu-b-32	pulmonary embolism	rs55982282	-0.100427771	0.036324776	0.005697198
lymphocyte	ieu-b-32	pulmonary embolism	rs56094005	-0.098231902	0.036169427	0.006609944
lymphocyte	ieu-b-32	pulmonary embolism	rs56179616	-0.0968599	0.036201114	0.007459438
lymphocyte	ieu-b-32	pulmonary embolism	rs56252104	-0.100603758	0.036209697	0.00546332
lymphocyte	ieu-b-32	pulmonary embolism	rs56308324	-0.100400318	0.036203025	0.005549816
lymphocyte	ieu-b-32	pulmonary embolism	rs56850780	-0.099127776	0.03617749	0.006143167
lymphocyte	ieu-b-32	pulmonary embolism	rs58265751	-0.099903599	0.036210574	0.005798527
lymphocyte	ieu-b-32	pulmonary embolism	rs58408429	-0.096428048	0.036066591	0.007503936
lymphocyte	ieu-b-32	pulmonary embolism	rs58667319	-0.099474658	0.03620617	0.006006059
lymphocyte	ieu-b-32	pulmonary embolism	rs6001858	-0.098529256	0.036144218	0.006410637
lymphocyte	ieu-b-32	pulmonary embolism	rs6021231	-0.10100114	0.036149773	0.005206638
lymphocyte	ieu-b-32	pulmonary embolism	rs603424	-0.099810811	0.036212148	0.005846296
lymphocyte	ieu-b-32	pulmonary embolism	rs60633358	-0.099859805	0.036214816	0.005825775
lymphocyte	ieu-b-32	pulmonary embolism	rs6072080	-0.101976264	0.036170856	0.004812947
lymphocyte	ieu-b-32	pulmonary embolism	rs607465	-0.098686677	0.036202564	0.006411511
lymphocyte	ieu-b-32	pulmonary embolism	rs6103270	-0.10120497	0.036125643	0.005087009
lymphocyte	ieu-b-32	pulmonary embolism	rs611847	-0.099611227	0.036209318	0.005941668
lymphocyte	ieu-b-32	pulmonary embolism	rs61387190	-0.09959831	0.036254015	0.00601
lymphocyte	ieu-b-32	pulmonary embolism	rs61750929	-0.103386157	0.036154092	0.004241788
lymphocyte	ieu-b-32	pulmonary embolism	rs61778219	-0.102425765	0.036066115	0.004512139
lymphocyte	ieu-b-32	pulmonary embolism	rs61850684	-0.103436133	0.03611802	0.004185429
lymphocyte	ieu-b-32	pulmonary embolism	rs61867141	-0.099717603	0.036214116	0.005895166
lymphocyte	ieu-b-32	pulmonary embolism	rs62091998	-0.098387146	0.036207546	0.006581483
lymphocyte	ieu-b-32	pulmonary embolism	rs62160910	-0.100568425	0.036168538	0.005426692
lymphocyte	ieu-b-32	pulmonary embolism	rs62241216	-0.09941043	0.036201876	0.006032637
lymphocyte	ieu-b-32	pulmonary embolism	rs62334097	-0.100794471	0.036201664	0.005365171
lymphocyte	ieu-b-32	pulmonary embolism	rs62389639	-0.099119031	0.036164378	0.006129133
lymphocyte	ieu-b-32	pulmonary embolism	rs62485905	-0.100051072	0.036224886	0.00574585
lymphocyte	ieu-b-32	pulmonary embolism	rs62621812	-0.100480764	0.036240353	0.005560684
lymphocyte	ieu-b-32	pulmonary embolism	rs631864	-0.101934281	0.036093056	0.004739768
lymphocyte	ieu-b-32	pulmonary embolism	rs6445826	-0.10127612	0.036206063	0.005154532
lymphocyte	ieu-b-32	pulmonary embolism	rs6474359	-0.098473841	0.036149085	0.006447635
lymphocyte	ieu-b-32	pulmonary embolism	rs6475476	-0.098269951	0.036075347	0.006449268
lymphocyte	ieu-b-32	pulmonary embolism	rs6487543	-0.098271289	0.036162558	0.006577969
lymphocyte	ieu-b-32	pulmonary embolism	rs6488548	-0.100582405	0.036215272	0.00548045
lymphocyte	ieu-b-32	pulmonary embolism	rs6502555	-0.097138865	0.036191883	0.007274801
lymphocyte	ieu-b-32	pulmonary embolism	rs6546881	-0.0992264	0.036187062	0.006105875
lymphocyte	ieu-b-32	pulmonary embolism	rs6554158	-0.100120918	0.036209548	0.005691548

lymphocyte	ieu-b-32	pulmonary embolism	rs6586777	-0.101031572	0.036167813	0.005215536
lymphocyte	ieu-b-32	pulmonary embolism	rs6679677	-0.100358814	0.036335273	0.005744465
lymphocyte	ieu-b-32	pulmonary embolism	rs6700896	-0.099344761	0.036219278	0.006090485
lymphocyte	ieu-b-32	pulmonary embolism	rs6721663	-0.099799019	0.036235857	0.005884463
lymphocyte	ieu-b-32	pulmonary embolism	rs6742799	-0.101060995	0.036284651	0.005349024
lymphocyte	ieu-b-32	pulmonary embolism	rs6743068	-0.100617234	0.036276805	0.005544032
lymphocyte	ieu-b-32	pulmonary embolism	rs67483792	-0.100622861	0.036176991	0.005412419
lymphocyte	ieu-b-32	pulmonary embolism	rs67516712	-0.101266324	0.036160366	0.005102692
lymphocyte	ieu-b-32	pulmonary embolism	rs6755786	-0.104149598	0.036008535	0.003823612
lymphocyte	ieu-b-32	pulmonary embolism	rs6755895	-0.096305407	0.03603664	0.007530402
lymphocyte	ieu-b-32	pulmonary embolism	rs678393	-0.09906591	0.03618741	0.006189317
lymphocyte	ieu-b-32	pulmonary embolism	rs6808949	-0.100474946	0.036197137	0.005507106
lymphocyte	ieu-b-32	pulmonary embolism	rs6926219	-0.100331123	0.036264248	0.005663245
lymphocyte	ieu-b-32	pulmonary embolism	rs6929523	-0.101318685	0.036163538	0.005083716
lymphocyte	ieu-b-32	pulmonary embolism	rs6955702	-0.100324877	0.036241645	0.005636325
lymphocyte	ieu-b-32	pulmonary embolism	rs696	-0.095732312	0.03609696	0.00799957
lymphocyte	ieu-b-32	pulmonary embolism	rs6966893	-0.099271082	0.036201678	0.006103512
lymphocyte	ieu-b-32	pulmonary embolism	rs7007986	-0.098131092	0.036200969	0.006713505
lymphocyte	ieu-b-32	pulmonary embolism	rs7071131	-0.102483574	0.036073791	0.004498039
lymphocyte	ieu-b-32	pulmonary embolism	rs707833	-0.099486709	0.036213281	0.006009846
lymphocyte	ieu-b-32	pulmonary embolism	rs7093481	-0.097842118	0.036115578	0.006745809
lymphocyte	ieu-b-32	pulmonary embolism	rs7127911	-0.099348562	0.036238655	0.006115787
lymphocyte	ieu-b-32	pulmonary embolism	rs71318973	-0.101198462	0.036155893	0.00512694
lymphocyte	ieu-b-32	pulmonary embolism	rs714027	-0.097291627	0.036283504	0.007330854
lymphocyte	ieu-b-32	pulmonary embolism	rs71420836	-0.100651738	0.036206779	0.005437327
lymphocyte	ieu-b-32	pulmonary embolism	rs7161799	-0.101221806	0.036164075	0.005126746
lymphocyte	ieu-b-32	pulmonary embolism	rs716723	-0.098648463	0.036129691	0.006325776
lymphocyte	ieu-b-32	pulmonary embolism	rs7192652	-0.102054935	0.036188605	0.004801096
lymphocyte	ieu-b-32	pulmonary embolism	rs721131	-0.099078218	0.036211834	0.006217725
lymphocyte	ieu-b-32	pulmonary embolism	rs721377	-0.101200186	0.036173197	0.005147479
lymphocyte	ieu-b-32	pulmonary embolism	rs7249236	-0.099031988	0.036229988	0.006267922
lymphocyte	ieu-b-32	pulmonary embolism	rs7252565	-0.099234076	0.036248531	0.00618888
lymphocyte	ieu-b-32	pulmonary embolism	rs7254517	-0.100895342	0.036269977	0.005406055
lymphocyte	ieu-b-32	pulmonary embolism	rs72634819	-0.100507318	0.036195163	0.00548942
lymphocyte	ieu-b-32	pulmonary embolism	rs72740550	-0.09927464	0.036205073	0.006106465
lymphocyte	ieu-b-32	pulmonary embolism	rs72835315	-0.101326739	0.036215495	0.005143865
lymphocyte	ieu-b-32	pulmonary embolism	rs72928038	-0.100009198	0.036239676	0.005786147
lymphocyte	ieu-b-32	pulmonary embolism	rs73000965	-0.098924376	0.036176566	0.006247743
lymphocyte	ieu-b-32	pulmonary embolism	rs7308380	-0.099767343	0.036214052	0.005870388
lymphocyte	ieu-b-32	pulmonary embolism	rs73105786	-0.09979851	0.036210793	0.005850526
lymphocyte	ieu-b-32	pulmonary embolism	rs7312770	-0.101266739	0.036138151	0.00507536
lymphocyte	ieu-b-32	pulmonary embolism	rs73142294	-0.099797443	0.0362135	0.005854741
lymphocyte	ieu-b-32	pulmonary embolism	rs73165514	-0.09991713	0.036221346	0.005806462
lymphocyte	ieu-b-32	pulmonary embolism	rs73207610	-0.09936003	0.036165131	0.006007008

lymphocyte	ieu-b-32	pulmonary embolism	rs73239280	-0.099024646	0.036267727	0.006326152
lymphocyte	ieu-b-32	pulmonary embolism	rs73961715	-0.099470534	0.036264172	0.006089137
lymphocyte	ieu-b-32	pulmonary embolism	rs74227709	-0.099050191	0.036189049	0.006199832
lymphocyte	ieu-b-32	pulmonary embolism	rs74280151	-0.10017643	0.036236456	0.00570065
lymphocyte	ieu-b-32	pulmonary embolism	rs74344617	-0.099878423	0.036206645	0.005805557
lymphocyte	ieu-b-32	pulmonary embolism	rs74408817	-0.101406676	0.036088124	0.004954556
lymphocyte	ieu-b-32	pulmonary embolism	rs744222	-0.100649336	0.036183703	0.005408821
lymphocyte	ieu-b-32	pulmonary embolism	rs74526869	-0.10043932	0.036233287	0.005570997
lymphocyte	ieu-b-32	pulmonary embolism	rs74679312	-0.099906651	0.036211712	0.00579857
lymphocyte	ieu-b-32	pulmonary embolism	rs748113	-0.100870442	0.036298819	0.005454491
lymphocyte	ieu-b-32	pulmonary embolism	rs74911261	-0.099670109	0.036197413	0.005895912
lymphocyte	ieu-b-32	pulmonary embolism	rs75018496	-0.098508505	0.036130198	0.006401253
lymphocyte	ieu-b-32	pulmonary embolism	rs75049211	-0.099436135	0.036192853	0.006007037
lymphocyte	ieu-b-32	pulmonary embolism	rs7529794	-0.101590071	0.036129588	0.004926133
lymphocyte	ieu-b-32	pulmonary embolism	rs7548606	-0.100516518	0.036211008	0.005505671
lymphocyte	ieu-b-32	pulmonary embolism	rs75628831	-0.103054908	0.036023124	0.004225752
lymphocyte	ieu-b-32	pulmonary embolism	rs75653581	-0.098443124	0.035800513	0.005963738
lymphocyte	ieu-b-32	pulmonary embolism	rs7572278	-0.100771533	0.03620855	0.005384442
lymphocyte	ieu-b-32	pulmonary embolism	rs7596846	-0.101318942	0.036108359	0.005016549
lymphocyte	ieu-b-32	pulmonary embolism	rs760908	-0.09876745	0.036144796	0.00628462
lymphocyte	ieu-b-32	pulmonary embolism	rs76428106	-0.098230813	0.036132183	0.006554863
lymphocyte	ieu-b-32	pulmonary embolism	rs7661349	-0.098651668	0.036233561	0.006475866
lymphocyte	ieu-b-32	pulmonary embolism	rs766228	-0.100864422	0.036160312	0.0052811
lymphocyte	ieu-b-32	pulmonary embolism	rs7672879	-0.099980815	0.03621279	0.005763783
lymphocyte	ieu-b-32	pulmonary embolism	rs76830965	-0.099209936	0.036188461	0.006116312
lymphocyte	ieu-b-32	pulmonary embolism	rs7696969	-0.099950939	0.036208103	0.005772042
lymphocyte	ieu-b-32	pulmonary embolism	rs77057307	-0.099525451	0.036209547	0.005985093
lymphocyte	ieu-b-32	pulmonary embolism	rs77101566	-0.100545031	0.036196562	0.005473657
lymphocyte	ieu-b-32	pulmonary embolism	rs77265382	-0.100515235	0.036207833	0.00550215
lymphocyte	ieu-b-32	pulmonary embolism	rs7757316	-0.102826466	0.036168237	0.004469042
lymphocyte	ieu-b-32	pulmonary embolism	rs7776054	-0.094841698	0.036237382	0.008864562
lymphocyte	ieu-b-32	pulmonary embolism	rs7780328	-0.102549001	0.036130124	0.004535111
lymphocyte	ieu-b-32	pulmonary embolism	rs77876222	-0.09957505	0.036186989	0.005929025
lymphocyte	ieu-b-32	pulmonary embolism	rs7790229	-0.100719186	0.03618486	0.005378215
lymphocyte	ieu-b-32	pulmonary embolism	rs77954449	-0.099790644	0.03620993	0.005853239
lymphocyte	ieu-b-32	pulmonary embolism	rs78058190	-0.101872119	0.036229319	0.004925448
lymphocyte	ieu-b-32	pulmonary embolism	rs780669	-0.100851616	0.036202836	0.005340602
lymphocyte	ieu-b-32	pulmonary embolism	rs78270096	-0.099179873	0.036208757	0.006160522
lymphocyte	ieu-b-32	pulmonary embolism	rs78394891	-0.101826578	0.036065969	0.00475258
lymphocyte	ieu-b-32	pulmonary embolism	rs7839516	-0.099540942	0.036215192	0.005985106
lymphocyte	ieu-b-32	pulmonary embolism	rs78487935	-0.099650657	0.036211806	0.005925367
lymphocyte	ieu-b-32	pulmonary embolism	rs7855586	-0.099144857	0.036197915	0.006163276
lymphocyte	ieu-b-32	pulmonary embolism	rs7893800	-0.101054129	0.036142983	0.005174671
lymphocyte	ieu-b-32	pulmonary embolism	rs78976959	-0.100098992	0.036209617	0.005702214

lymphocyte	ieu-b-32	pulmonary embolism	rs78983078	-0.100105713	0.036200454	0.005686759
lymphocyte	ieu-b-32	pulmonary embolism	rs79237520	-0.104459202	0.036048063	0.003758192
lymphocyte	ieu-b-32	pulmonary embolism	rs7924213	-0.099680786	0.036206118	0.005902529
lymphocyte	ieu-b-32	pulmonary embolism	rs79503	-0.09762763	0.036234226	0.007052639
lymphocyte	ieu-b-32	pulmonary embolism	rs79805393	-0.098965678	0.0361795	0.006230308
lymphocyte	ieu-b-32	pulmonary embolism	rs7983682	-0.099980397	0.036212462	0.005763545
lymphocyte	ieu-b-32	pulmonary embolism	rs80006387	-0.099532083	0.036192781	0.005958555
lymphocyte	ieu-b-32	pulmonary embolism	rs8004780	-0.096222172	0.035982936	0.007492885
lymphocyte	ieu-b-32	pulmonary embolism	rs80177247	-0.100360284	0.036196294	0.005559892
lymphocyte	ieu-b-32	pulmonary embolism	rs8046391	-0.097330515	0.036178551	0.00713916
lymphocyte	ieu-b-32	pulmonary embolism	rs8052370	-0.099842355	0.036237089	0.005864673
lymphocyte	ieu-b-32	pulmonary embolism	rs8075090	-0.100308218	0.036234366	0.005634659
lymphocyte	ieu-b-32	pulmonary embolism	rs8096327	-0.100301144	0.036204021	0.005598025
lymphocyte	ieu-b-32	pulmonary embolism	rs8181326	-0.098062642	0.036073719	0.006559934
lymphocyte	ieu-b-32	pulmonary embolism	rs9328393	-0.099130371	0.036238031	0.006227913
lymphocyte	ieu-b-32	pulmonary embolism	rs937283	-0.099994916	0.03620621	0.005748074
lymphocyte	ieu-b-32	pulmonary embolism	rs9375486	-0.10000425	0.036213167	0.005752878
lymphocyte	ieu-b-32	pulmonary embolism	rs949349	-0.100384385	0.036200771	0.005554379
lymphocyte	ieu-b-32	pulmonary embolism	rs9525619	-0.103316065	0.036067742	0.004176675
lymphocyte	ieu-b-32	pulmonary embolism	rs9532679	-0.098340613	0.036231446	0.006642929
lymphocyte	ieu-b-32	pulmonary embolism	rs9534338	-0.100291416	0.036213739	0.005615446
lymphocyte	ieu-b-32	pulmonary embolism	rs9592965	-0.099575514	0.036205925	0.005954897
lymphocyte	ieu-b-32	pulmonary embolism	rs9605047	-0.100938022	0.036215965	0.005317977
lymphocyte	ieu-b-32	pulmonary embolism	rs9662343	-0.098764193	0.036165249	0.006315891
lymphocyte	ieu-b-32	pulmonary embolism	rs968478	-0.098975184	0.036190171	0.006240612
lymphocyte	ieu-b-32	pulmonary embolism	rs9835571	-0.101563889	0.036199254	0.005020896
lymphocyte	ieu-b-32	pulmonary embolism	rs9864087	-0.099742007	0.036214201	0.005883156
lymphocyte	ieu-b-32	pulmonary embolism	rs9864216	-0.098078075	0.036186899	0.006721833
lymphocyte	ieu-b-32	pulmonary embolism	rs9898958	-0.101903129	0.036083189	0.004741115
lymphocyte	ieu-b-32	pulmonary embolism	rs9906320	-0.09931952	0.03623777	0.006129473
lymphocyte	ieu-b-32	pulmonary embolism	rs9920	-0.09921067	0.036208221	0.006143844
lymphocyte	ieu-b-32	pulmonary embolism	rs9937837	-0.099899483	0.036212849	0.005803621
lymphocyte	ieu-b-32	pulmonary embolism	rs9939124	-0.098995871	0.036200661	0.006244803
lymphocyte	ieu-b-32	pulmonary embolism	All	-0.099904019	0.036157129	0.005726369
eosinophil	ieu-b-33	pulmonary embolism	rs1004870	-0.022754919	0.040029185	0.569723867
eosinophil	ieu-b-33	pulmonary embolism	rs10059018	-0.020769228	0.039983764	0.603452867
eosinophil	ieu-b-33	pulmonary embolism	rs10062687	-0.022296255	0.040007115	0.577317531
eosinophil	ieu-b-33	pulmonary embolism	rs10100356	-0.021230014	0.039975909	0.595370125
eosinophil	ieu-b-33	pulmonary embolism	rs10165678	-0.020346472	0.039930159	0.610365718
eosinophil	ieu-b-33	pulmonary embolism	rs10174238	-0.020726811	0.039938885	0.603786414
eosinophil	ieu-b-33	pulmonary embolism	rs10195713	-0.023082562	0.039953676	0.563444319
eosinophil	ieu-b-33	pulmonary embolism	rs10208293	-0.016795124	0.040433651	0.677867475
eosinophil	ieu-b-33	pulmonary embolism	rs10243429	-0.022129096	0.039992066	0.58003256
eosinophil	ieu-b-33	pulmonary embolism	rs10275896	-0.022291028	0.039968727	0.577041249

eosinophil	ieu-b-33	pulmonary embolism	rs1036332	-0.022929564	0.040048794	0.566955722
eosinophil	ieu-b-33	pulmonary embolism	rs1037674	-0.020701965	0.039941876	0.604247387
eosinophil	ieu-b-33	pulmonary embolism	rs10472984	-0.020129837	0.040077175	0.615472799
eosinophil	ieu-b-33	pulmonary embolism	rs1047891	-0.024565322	0.039805376	0.537145174
eosinophil	ieu-b-33	pulmonary embolism	rs1057258	-0.019693775	0.039973257	0.622243116
eosinophil	ieu-b-33	pulmonary embolism	rs1059091	-0.017080894	0.039945009	0.668934928
eosinophil	ieu-b-33	pulmonary embolism	rs10777378	-0.022316605	0.040019234	0.577085479
eosinophil	ieu-b-33	pulmonary embolism	rs10782957	-0.022068092	0.040018681	0.581328818
eosinophil	ieu-b-33	pulmonary embolism	rs10876550	-0.020771107	0.039956831	0.603176015
eosinophil	ieu-b-33	pulmonary embolism	rs10900595	-0.021597087	0.039988468	0.589139881
eosinophil	ieu-b-33	pulmonary embolism	rs10930337	-0.021343287	0.039987685	0.593516941
eosinophil	ieu-b-33	pulmonary embolism	rs1099448	-0.020708722	0.039984289	0.604513057
eosinophil	ieu-b-33	pulmonary embolism	rs10995240	-0.018242938	0.040098588	0.649143683
eosinophil	ieu-b-33	pulmonary embolism	rs11024646	-0.020246714	0.039934853	0.612159315
eosinophil	ieu-b-33	pulmonary embolism	rs11071559	-0.022031043	0.039997161	0.581760382
eosinophil	ieu-b-33	pulmonary embolism	rs11079340	-0.020264558	0.039900313	0.611537732
eosinophil	ieu-b-33	pulmonary embolism	rs11088236	-0.019553393	0.039923194	0.62429293
eosinophil	ieu-b-33	pulmonary embolism	rs11120043	-0.022205129	0.039972575	0.578546497
eosinophil	ieu-b-33	pulmonary embolism	rs111759324	-0.022959853	0.040005914	0.566027699
eosinophil	ieu-b-33	pulmonary embolism	rs1120275	-0.019072018	0.039951153	0.633089126
eosinophil	ieu-b-33	pulmonary embolism	rs11204682	-0.014029532	0.039766545	0.724240368
eosinophil	ieu-b-33	pulmonary embolism	rs112255429	-0.021023753	0.039996607	0.599139471
eosinophil	ieu-b-33	pulmonary embolism	rs11228990	-0.021168209	0.039974882	0.596432459
eosinophil	ieu-b-33	pulmonary embolism	rs11236813	-0.022819446	0.039992154	0.568272123
eosinophil	ieu-b-33	pulmonary embolism	rs11255507	-0.019193226	0.039863018	0.630175745
eosinophil	ieu-b-33	pulmonary embolism	rs113473633	-0.021536484	0.039994636	0.590242732
eosinophil	ieu-b-33	pulmonary embolism	rs113496608	-0.020438598	0.039916623	0.608628449
eosinophil	ieu-b-33	pulmonary embolism	rs113542380	-0.02103409	0.039953017	0.59856125
eosinophil	ieu-b-33	pulmonary embolism	rs113859409	-0.020410312	0.039927459	0.609221624
eosinophil	ieu-b-33	pulmonary embolism	rs114152720	-0.022818277	0.039957154	0.567953128
eosinophil	ieu-b-33	pulmonary embolism	rs114741563	-0.022118932	0.039991949	0.580205466
eosinophil	ieu-b-33	pulmonary embolism	rs11495865	-0.021876908	0.039989842	0.584336397
eosinophil	ieu-b-33	pulmonary embolism	rs11555542	-0.022372536	0.039990532	0.57585749
eosinophil	ieu-b-33	pulmonary embolism	rs115647629	-0.022363735	0.040002366	0.576120558
eosinophil	ieu-b-33	pulmonary embolism	rs115697464	-0.0228785	0.039962029	0.566979242
eosinophil	ieu-b-33	pulmonary embolism	rs11628569	-0.022322078	0.039990331	0.576716792
eosinophil	ieu-b-33	pulmonary embolism	rs11648292	-0.022557413	0.039959859	0.572412674
eosinophil	ieu-b-33	pulmonary embolism	rs11684770	-0.021161466	0.039980349	0.59659966
eosinophil	ieu-b-33	pulmonary embolism	rs116888884	-0.022567661	0.039900895	0.571670592
eosinophil	ieu-b-33	pulmonary embolism	rs11695281	-0.021186923	0.040022717	0.596547126
eosinophil	ieu-b-33	pulmonary embolism	rs11701475	-0.019841496	0.039861486	0.61865246
eosinophil	ieu-b-33	pulmonary embolism	rs11702918	-0.021355763	0.039985678	0.593282511
eosinophil	ieu-b-33	pulmonary embolism	rs1170439	-0.020852368	0.039987503	0.602037917
eosinophil	ieu-b-33	pulmonary embolism	rs117068593	-0.019759592	0.039936316	0.620757165

eosinophil	ieu-b-33	pulmonary embolism	rs117481629	-0.021246227	0.039972854	0.595060987
eosinophil	ieu-b-33	pulmonary embolism	rs117577334	-0.021860605	0.039978507	0.584510003
eosinophil	ieu-b-33	pulmonary embolism	rs1178016	-0.021560033	0.039985091	0.589747612
eosinophil	ieu-b-33	pulmonary embolism	rs11786536	-0.020562638	0.039996439	0.607173882
eosinophil	ieu-b-33	pulmonary embolism	rs117955557	-0.018099902	0.039948171	0.650487813
eosinophil	ieu-b-33	pulmonary embolism	rs117961539	-0.02307786	0.039887994	0.562881668
eosinophil	ieu-b-33	pulmonary embolism	rs118013485	-0.024698031	0.039880549	0.535718629
eosinophil	ieu-b-33	pulmonary embolism	rs11869228	-0.019913588	0.039907275	0.617781893
eosinophil	ieu-b-33	pulmonary embolism	rs11886928	-0.021303551	0.039980661	0.594139913
eosinophil	ieu-b-33	pulmonary embolism	rs11888323	-0.022223843	0.039988503	0.578377795
eosinophil	ieu-b-33	pulmonary embolism	rs11890306	-0.023185933	0.039908697	0.561257685
eosinophil	ieu-b-33	pulmonary embolism	rs11931711	-0.020333696	0.039971676	0.610960686
eosinophil	ieu-b-33	pulmonary embolism	rs12100034	-0.021446692	0.039997515	0.591819902
eosinophil	ieu-b-33	pulmonary embolism	rs12154498	-0.021922418	0.040036793	0.583996262
eosinophil	ieu-b-33	pulmonary embolism	rs121564	-0.022347804	0.039981834	0.576196569
eosinophil	ieu-b-33	pulmonary embolism	rs12208103	-0.023046414	0.040051275	0.565005438
eosinophil	ieu-b-33	pulmonary embolism	rs12408934	-0.024893478	0.039957105	0.533281207
eosinophil	ieu-b-33	pulmonary embolism	rs12487980	-0.021720847	0.039986142	0.586985662
eosinophil	ieu-b-33	pulmonary embolism	rs12515180	-0.034611764	0.040210865	0.389372153
eosinophil	ieu-b-33	pulmonary embolism	rs12530946	-0.018196662	0.040087039	0.649880057
eosinophil	ieu-b-33	pulmonary embolism	rs12540285	-0.021012051	0.039975118	0.599146527
eosinophil	ieu-b-33	pulmonary embolism	rs12545733	-0.020535187	0.040042177	0.608063996
eosinophil	ieu-b-33	pulmonary embolism	rs1257192	-0.021440333	0.039980825	0.591775144
eosinophil	ieu-b-33	pulmonary embolism	rs12581511	-0.020337435	0.039936059	0.610577054
eosinophil	ieu-b-33	pulmonary embolism	rs12705849	-0.021409055	0.040010854	0.592593729
eosinophil	ieu-b-33	pulmonary embolism	rs12820863	-0.025042238	0.039875514	0.529997117
eosinophil	ieu-b-33	pulmonary embolism	rs12827988	-0.020117159	0.039936983	0.614456352
eosinophil	ieu-b-33	pulmonary embolism	rs12861824	-0.02307189	0.039975805	0.563840587
eosinophil	ieu-b-33	pulmonary embolism	rs12878610	-0.022679734	0.039959687	0.570330145
eosinophil	ieu-b-33	pulmonary embolism	rs12882281	-0.021012668	0.0399606	0.599003093
eosinophil	ieu-b-33	pulmonary embolism	rs12928503	-0.021400785	0.039961915	0.59228376
eosinophil	ieu-b-33	pulmonary embolism	rs12941068	-0.023501593	0.039841589	0.555273853
eosinophil	ieu-b-33	pulmonary embolism	rs13033333	-0.022077304	0.039982677	0.580830706
eosinophil	ieu-b-33	pulmonary embolism	rs13073683	-0.022443573	0.039985179	0.574594876
eosinophil	ieu-b-33	pulmonary embolism	rs13105682	-0.02192866	0.039966432	0.583227175
eosinophil	ieu-b-33	pulmonary embolism	rs13120371	-0.019573584	0.039868771	0.623461622
eosinophil	ieu-b-33	pulmonary embolism	rs13138355	-0.019767295	0.040179255	0.622735159
eosinophil	ieu-b-33	pulmonary embolism	rs13139941	-0.021152137	0.039970936	0.59667509
eosinophil	ieu-b-33	pulmonary embolism	rs13207791	-0.022681449	0.039901233	0.56973637
eosinophil	ieu-b-33	pulmonary embolism	rs13226583	-0.02305952	0.040144552	0.5656889
eosinophil	ieu-b-33	pulmonary embolism	rs1323650	-0.022039839	0.039985042	0.581495132
eosinophil	ieu-b-33	pulmonary embolism	rs13251643	-0.02161799	0.039990602	0.588799339
eosinophil	ieu-b-33	pulmonary embolism	rs13511	-0.022757644	0.039976377	0.569167957
eosinophil	ieu-b-33	pulmonary embolism	rs1353286	-0.022666673	0.040001106	0.570951216

eosinophil	ieu-b-33	pulmonary embolism	rs1365623	-0.021922222	0.039984233	0.583505391
eosinophil	ieu-b-33	pulmonary embolism	rs139640694	-0.022950552	0.039952906	0.565670016
eosinophil	ieu-b-33	pulmonary embolism	rs140411271	-0.02148832	0.03998989	0.591030146
eosinophil	ieu-b-33	pulmonary embolism	rs140509806	-0.020854761	0.039966692	0.601807126
eosinophil	ieu-b-33	pulmonary embolism	rs1406449	-0.023553093	0.039854874	0.55453954
eosinophil	ieu-b-33	pulmonary embolism	rs1414517	-0.022790558	0.039988397	0.568725672
eosinophil	ieu-b-33	pulmonary embolism	rs1427499	-0.023061962	0.039967183	0.563924283
eosinophil	ieu-b-33	pulmonary embolism	rs143491704	-0.02181192	0.039978991	0.585351555
eosinophil	ieu-b-33	pulmonary embolism	rs145823386	-0.019745736	0.039837409	0.620135637
eosinophil	ieu-b-33	pulmonary embolism	rs145947882	-0.024010752	0.039942327	0.547749724
eosinophil	ieu-b-33	pulmonary embolism	rs146078144	-0.023110845	0.039821174	0.561668009
eosinophil	ieu-b-33	pulmonary embolism	rs14713	-0.022209256	0.039975754	0.578506114
eosinophil	ieu-b-33	pulmonary embolism	rs1471816	-0.021392236	0.03997993	0.59259843
eosinophil	ieu-b-33	pulmonary embolism	rs1479918	-0.020971374	0.039970683	0.599813384
eosinophil	ieu-b-33	pulmonary embolism	rs149110519	-0.021821625	0.039987825	0.585267547
eosinophil	ieu-b-33	pulmonary embolism	rs150640087	-0.023276767	0.04000848	0.560704216
eosinophil	ieu-b-33	pulmonary embolism	rs1516527	-0.021886291	0.039983878	0.584119139
eosinophil	ieu-b-33	pulmonary embolism	rs152197	-0.025577434	0.040098634	0.52356288
eosinophil	ieu-b-33	pulmonary embolism	rs1539174	-0.020765715	0.040061685	0.60421861
eosinophil	ieu-b-33	pulmonary embolism	rs159963	-0.021817196	0.040007055	0.585524016
eosinophil	ieu-b-33	pulmonary embolism	rs1611236	-0.023228482	0.040091504	0.56232832
eosinophil	ieu-b-33	pulmonary embolism	rs1672753	-0.022868079	0.039957085	0.567107901
eosinophil	ieu-b-33	pulmonary embolism	rs1684578	-0.022752942	0.039972681	0.569212059
eosinophil	ieu-b-33	pulmonary embolism	rs1689510	-0.018769143	0.039949657	0.638483713
eosinophil	ieu-b-33	pulmonary embolism	rs16903574	-0.021366128	0.040006218	0.593292976
eosinophil	ieu-b-33	pulmonary embolism	rs16956811	-0.021060419	0.03997394	0.598295171
eosinophil	ieu-b-33	pulmonary embolism	rs17061503	-0.024432963	0.040052868	0.541850018
eosinophil	ieu-b-33	pulmonary embolism	rs17175830	-0.026969492	0.039824463	0.498273245
eosinophil	ieu-b-33	pulmonary embolism	rs17293632	-0.026160092	0.039867005	0.511705725
eosinophil	ieu-b-33	pulmonary embolism	rs174548	-0.027036177	0.039690746	0.495763421
eosinophil	ieu-b-33	pulmonary embolism	rs17482472	-0.020996736	0.039988719	0.599536981
eosinophil	ieu-b-33	pulmonary embolism	rs175705	-0.021461828	0.040112498	0.592621554
eosinophil	ieu-b-33	pulmonary embolism	rs17668272	-0.022668378	0.040009389	0.571001969
eosinophil	ieu-b-33	pulmonary embolism	rs17682575	-0.021136205	0.039978473	0.597020756
eosinophil	ieu-b-33	pulmonary embolism	rs17689024	-0.021949667	0.039989131	0.583080385
eosinophil	ieu-b-33	pulmonary embolism	rs17758695	-0.015745652	0.040138731	0.694850776
eosinophil	ieu-b-33	pulmonary embolism	rs17849501	-0.021920186	0.039981825	0.583517671
eosinophil	ieu-b-33	pulmonary embolism	rs1800692	-0.022647202	0.039987341	0.571149562
eosinophil	ieu-b-33	pulmonary embolism	rs180506	-0.024637815	0.039842547	0.536325195
eosinophil	ieu-b-33	pulmonary embolism	rs1828803	-0.022056423	0.039984739	0.581208027
eosinophil	ieu-b-33	pulmonary embolism	rs1861489	-0.02272718	0.039940016	0.569333873
eosinophil	ieu-b-33	pulmonary embolism	rs201798	-0.022645403	0.039994805	0.571251968
eosinophil	ieu-b-33	pulmonary embolism	rs2025489	-0.021232882	0.039992527	0.595473353
eosinophil	ieu-b-33	pulmonary embolism	rs2043293	-0.022342852	0.039957606	0.576049846

eosinophil	ieu-b-33	pulmonary embolism	rs2053927	-0.02371429	0.039778497	0.551069337
eosinophil	ieu-b-33	pulmonary embolism	rs2089979	-0.020535688	0.039957025	0.607290892
eosinophil	ieu-b-33	pulmonary embolism	rs2095044	-0.021830932	0.040627041	0.591026009
eosinophil	ieu-b-33	pulmonary embolism	rs2182885	-0.021790711	0.04004182	0.586304672
eosinophil	ieu-b-33	pulmonary embolism	rs2223043	-0.021707274	0.040008137	0.587424888
eosinophil	ieu-b-33	pulmonary embolism	rs2228467	-0.020150595	0.040068953	0.615036005
eosinophil	ieu-b-33	pulmonary embolism	rs2239633	-0.020853725	0.040089833	0.602941674
eosinophil	ieu-b-33	pulmonary embolism	rs2242193	-0.018797073	0.039643366	0.635389876
eosinophil	ieu-b-33	pulmonary embolism	rs2253427	-0.020995641	0.039959207	0.599286487
eosinophil	ieu-b-33	pulmonary embolism	rs2255531	-0.024957097	0.039753522	0.530137678
eosinophil	ieu-b-33	pulmonary embolism	rs2282718	-0.020936262	0.039956358	0.600293682
eosinophil	ieu-b-33	pulmonary embolism	rs2399441	-0.021088223	0.039996371	0.598017619
eosinophil	ieu-b-33	pulmonary embolism	rs2419313	-0.024650266	0.039731884	0.534984457
eosinophil	ieu-b-33	pulmonary embolism	rs2431097	-0.022711903	0.0399701	0.569884008
eosinophil	ieu-b-33	pulmonary embolism	rs244689	-0.022669703	0.039991804	0.570810177
eosinophil	ieu-b-33	pulmonary embolism	rs2497318	-0.019430243	0.040026035	0.627364173
eosinophil	ieu-b-33	pulmonary embolism	rs2502995	-0.018441213	0.039882295	0.643800979
eosinophil	ieu-b-33	pulmonary embolism	rs2505521	-0.020902912	0.039963929	0.600943312
eosinophil	ieu-b-33	pulmonary embolism	rs2566133	-0.020600152	0.039957354	0.606166312
eosinophil	ieu-b-33	pulmonary embolism	rs2568928	-0.021163502	0.039969528	0.59646495
eosinophil	ieu-b-33	pulmonary embolism	rs2579506	-0.01743556	0.040148863	0.664090342
eosinophil	ieu-b-33	pulmonary embolism	rs2646438	-0.022787798	0.039998176	0.568867001
eosinophil	ieu-b-33	pulmonary embolism	rs2683913	-0.021616362	0.039984723	0.58877262
eosinophil	ieu-b-33	pulmonary embolism	rs2713548	-0.022900208	0.039933026	0.566329629
eosinophil	ieu-b-33	pulmonary embolism	rs2788211	-0.023607697	0.039847242	0.553545966
eosinophil	ieu-b-33	pulmonary embolism	rs2807740	-0.017100075	0.039894395	0.668189954
eosinophil	ieu-b-33	pulmonary embolism	rs28362902	-0.022848154	0.039924461	0.56712918
eosinophil	ieu-b-33	pulmonary embolism	rs2838317	-0.020522823	0.039929808	0.607271229
eosinophil	ieu-b-33	pulmonary embolism	rs28383314	-0.023540806	0.040296921	0.559096702
eosinophil	ieu-b-33	pulmonary embolism	rs28421324	-0.020664343	0.0400171	0.605583569
eosinophil	ieu-b-33	pulmonary embolism	rs2847266	-0.02099182	0.039972377	0.599473256
eosinophil	ieu-b-33	pulmonary embolism	rs28532037	-0.025464211	0.039777884	0.522068581
eosinophil	ieu-b-33	pulmonary embolism	rs2894401	-0.0226824	0.039984028	0.570519539
eosinophil	ieu-b-33	pulmonary embolism	rs290430	-0.022200879	0.039977535	0.578666322
eosinophil	ieu-b-33	pulmonary embolism	rs2920505	-0.024995397	0.039986725	0.531910427
eosinophil	ieu-b-33	pulmonary embolism	rs2942062	-0.021059954	0.039959856	0.598174304
eosinophil	ieu-b-33	pulmonary embolism	rs295	-0.021201866	0.039982187	0.595915874
eosinophil	ieu-b-33	pulmonary embolism	rs295273	-0.023267587	0.039961591	0.560399135
eosinophil	ieu-b-33	pulmonary embolism	rs2979489	-0.020761421	0.039953474	0.603314556
eosinophil	ieu-b-33	pulmonary embolism	rs2992333	-0.021792895	0.040074234	0.586570077
eosinophil	ieu-b-33	pulmonary embolism	rs301162	-0.022391683	0.039993311	0.575557399
eosinophil	ieu-b-33	pulmonary embolism	rs3024971	-0.021827577	0.040000809	0.585287031
eosinophil	ieu-b-33	pulmonary embolism	rs3093023	-0.02142476	0.039985928	0.592091605
eosinophil	ieu-b-33	pulmonary embolism	rs3096309	-0.021704435	0.039983233	0.587241058

eosinophil	ieu-b-33	pulmonary embolism	rs3110791	-0.022003544	0.039991781	0.582181079
eosinophil	ieu-b-33	pulmonary embolism	rs3218148	-0.020413688	0.039965332	0.609501681
eosinophil	ieu-b-33	pulmonary embolism	rs33982662	-0.021686307	0.040005099	0.587757495
eosinophil	ieu-b-33	pulmonary embolism	rs34173062	-0.025264759	0.039905532	0.526659068
eosinophil	ieu-b-33	pulmonary embolism	rs34210653	-0.02155943	0.040057571	0.590431317
eosinophil	ieu-b-33	pulmonary embolism	rs34212866	-0.020093422	0.039913786	0.614668592
eosinophil	ieu-b-33	pulmonary embolism	rs34290285	-0.022467461	0.040113963	0.575417507
eosinophil	ieu-b-33	pulmonary embolism	rs34363176	-0.022457348	0.039954274	0.574064076
eosinophil	ieu-b-33	pulmonary embolism	rs34439695	-0.021324703	0.039982636	0.59379195
eosinophil	ieu-b-33	pulmonary embolism	rs34448954	-0.021432961	0.039995616	0.592039591
eosinophil	ieu-b-33	pulmonary embolism	rs34466956	-0.022455778	0.039973923	0.574279094
eosinophil	ieu-b-33	pulmonary embolism	rs34495	-0.024068751	0.039846666	0.545820873
eosinophil	ieu-b-33	pulmonary embolism	rs34505104	-0.025547195	0.039867094	0.521646755
eosinophil	ieu-b-33	pulmonary embolism	rs346835	-0.024395627	0.0399725	0.541656303
eosinophil	ieu-b-33	pulmonary embolism	rs350836	-0.022288479	0.039988434	0.577272502
eosinophil	ieu-b-33	pulmonary embolism	rs35249183	-0.021928745	0.040003273	0.5835726
eosinophil	ieu-b-33	pulmonary embolism	rs35409523	-0.024500162	0.04000677	0.540272882
eosinophil	ieu-b-33	pulmonary embolism	rs35850753	-0.02279827	0.039958214	0.568302826
eosinophil	ieu-b-33	pulmonary embolism	rs36084354	-0.020295029	0.040018261	0.612053642
eosinophil	ieu-b-33	pulmonary embolism	rs3731211	-0.021405208	0.039992477	0.592490217
eosinophil	ieu-b-33	pulmonary embolism	rs3742704	-0.022200245	0.039973438	0.578638242
eosinophil	ieu-b-33	pulmonary embolism	rs3746420	-0.021877314	0.039992061	0.584350276
eosinophil	ieu-b-33	pulmonary embolism	rs3747869	-0.020663511	0.039968652	0.605161092
eosinophil	ieu-b-33	pulmonary embolism	rs3756374	-0.022609841	0.039938332	0.571313013
eosinophil	ieu-b-33	pulmonary embolism	rs3759332	-0.020987729	0.039962492	0.59945411
eosinophil	ieu-b-33	pulmonary embolism	rs3785356	-0.019831382	0.040015139	0.62017879
eosinophil	ieu-b-33	pulmonary embolism	rs3786586	-0.024514998	0.039964058	0.539594605
eosinophil	ieu-b-33	pulmonary embolism	rs3790163	-0.023932077	0.03976872	0.547319645
eosinophil	ieu-b-33	pulmonary embolism	rs3812206	-0.021204833	0.03997495	0.595797873
eosinophil	ieu-b-33	pulmonary embolism	rs3823536	-0.021999915	0.040003295	0.582351905
eosinophil	ieu-b-33	pulmonary embolism	rs3824867	-0.017474731	0.039602131	0.659026712
eosinophil	ieu-b-33	pulmonary embolism	rs3846855	-0.018748149	0.039962632	0.638968133
eosinophil	ieu-b-33	pulmonary embolism	rs3850107	-0.020148208	0.039889831	0.613491161
eosinophil	ieu-b-33	pulmonary embolism	rs3950296	-0.020437032	0.039995318	0.609361124
eosinophil	ieu-b-33	pulmonary embolism	rs397187	-0.022018419	0.039983923	0.581851811
eosinophil	ieu-b-33	pulmonary embolism	rs4074672	-0.023607915	0.039870805	0.553776713
eosinophil	ieu-b-33	pulmonary embolism	rs410867	-0.032225244	0.039943221	0.419795379
eosinophil	ieu-b-33	pulmonary embolism	rs412884	-0.018601133	0.040239101	0.643891199
eosinophil	ieu-b-33	pulmonary embolism	rs41313381	-0.022241961	0.039981347	0.57800002
eosinophil	ieu-b-33	pulmonary embolism	rs4148757	-0.022063806	0.039982191	0.581057393
eosinophil	ieu-b-33	pulmonary embolism	rs4149909	-0.020863124	0.039954325	0.601548926
eosinophil	ieu-b-33	pulmonary embolism	rs4236746	-0.021175714	0.039996228	0.59649833
eosinophil	ieu-b-33	pulmonary embolism	rs4240624	-0.024772263	0.039728438	0.532929839
eosinophil	ieu-b-33	pulmonary embolism	rs4243951	-0.023391069	0.039843972	0.557158909

eosinophil	ieu-b-33	pulmonary embolism	rs4280242	-0.020795952	0.040047968	0.603568233
eosinophil	ieu-b-33	pulmonary embolism	rs4310436	-0.020399371	0.039927555	0.609414354
eosinophil	ieu-b-33	pulmonary embolism	rs4366169	-0.02284284	0.039928424	0.567257812
eosinophil	ieu-b-33	pulmonary embolism	rs4409785	-0.020731488	0.039949316	0.60379926
eosinophil	ieu-b-33	pulmonary embolism	rs45577137	-0.023789402	0.040038793	0.552405885
eosinophil	ieu-b-33	pulmonary embolism	rs4652560	-0.019002973	0.039770624	0.632782386
eosinophil	ieu-b-33	pulmonary embolism	rs466547	-0.022228867	0.039973181	0.578146237
eosinophil	ieu-b-33	pulmonary embolism	rs4675190	-0.022306327	0.03997185	0.576809624
eosinophil	ieu-b-33	pulmonary embolism	rs4680250	-0.022107559	0.039989651	0.580378441
eosinophil	ieu-b-33	pulmonary embolism	rs4703589	-0.024015085	0.039838582	0.546635279
eosinophil	ieu-b-33	pulmonary embolism	rs4703730	-0.020741763	0.039974906	0.603851687
eosinophil	ieu-b-33	pulmonary embolism	rs4722171	-0.017733134	0.039919198	0.656879417
eosinophil	ieu-b-33	pulmonary embolism	rs473739	-0.020753396	0.039950478	0.603427418
eosinophil	ieu-b-33	pulmonary embolism	rs4739140	-0.021842782	0.039993064	0.584953034
eosinophil	ieu-b-33	pulmonary embolism	rs4746153	-0.020777023	0.039951648	0.603025795
eosinophil	ieu-b-33	pulmonary embolism	rs4849748	-0.020419652	0.039913232	0.608930249
eosinophil	ieu-b-33	pulmonary embolism	rs4857909	-0.031884337	0.040567944	0.431897342
eosinophil	ieu-b-33	pulmonary embolism	rs4870977	-0.020712076	0.039948545	0.604131184
eosinophil	ieu-b-33	pulmonary embolism	rs4908835	-0.023197375	0.039932617	0.56129911
eosinophil	ieu-b-33	pulmonary embolism	rs4931002	-0.022293137	0.039996274	0.577267598
eosinophil	ieu-b-33	pulmonary embolism	rs495149	-0.023380632	0.039952824	0.558409218
eosinophil	ieu-b-33	pulmonary embolism	rs496475	-0.016857801	0.039957937	0.673106279
eosinophil	ieu-b-33	pulmonary embolism	rs520461	-0.020778961	0.039949505	0.60297254
eosinophil	ieu-b-33	pulmonary embolism	rs526369	-0.022040944	0.039994122	0.581561983
eosinophil	ieu-b-33	pulmonary embolism	rs556063	-0.021607149	0.03998814	0.588963309
eosinophil	ieu-b-33	pulmonary embolism	rs55868524	-0.022290307	0.039978068	0.577142557
eosinophil	ieu-b-33	pulmonary embolism	rs55879743	-0.022536488	0.040050726	0.573640128
eosinophil	ieu-b-33	pulmonary embolism	rs55955211	-0.022475397	0.040001857	0.574212206
eosinophil	ieu-b-33	pulmonary embolism	rs56117721	-0.016786011	0.040344108	0.677358092
eosinophil	ieu-b-33	pulmonary embolism	rs56179563	-0.020600993	0.03997641	0.606323316
eosinophil	ieu-b-33	pulmonary embolism	rs56195338	-0.021406829	0.039976505	0.592314342
eosinophil	ieu-b-33	pulmonary embolism	rs56330463	-0.017926256	0.040060883	0.654531906
eosinophil	ieu-b-33	pulmonary embolism	rs574183	-0.021420789	0.039988699	0.592185915
eosinophil	ieu-b-33	pulmonary embolism	rs5747308	-0.021175127	0.039990434	0.596455313
eosinophil	ieu-b-33	pulmonary embolism	rs57633475	-0.021513831	0.040005474	0.590734343
eosinophil	ieu-b-33	pulmonary embolism	rs57834782	-0.016825297	0.040234039	0.675811393
eosinophil	ieu-b-33	pulmonary embolism	rs58745116	-0.021202407	0.039985017	0.595932514
eosinophil	ieu-b-33	pulmonary embolism	rs58833930	-0.021098612	0.039993288	0.597809053
eosinophil	ieu-b-33	pulmonary embolism	rs60175411	-0.021414016	0.040018295	0.592576811
eosinophil	ieu-b-33	pulmonary embolism	rs60600003	-0.021315408	0.04003943	0.594476393
eosinophil	ieu-b-33	pulmonary embolism	rs60646393	-0.021482941	0.039991268	0.591135837
eosinophil	ieu-b-33	pulmonary embolism	rs6103572	-0.023299269	0.039989233	0.56013665
eosinophil	ieu-b-33	pulmonary embolism	rs6139104	-0.021395907	0.039980183	0.592537281
eosinophil	ieu-b-33	pulmonary embolism	rs6141755	-0.022285967	0.039990421	0.577334329

eosinophil	ieu-b-33	pulmonary embolism	rs61426394	-0.021538965	0.03998272	0.590089142
eosinophil	ieu-b-33	pulmonary embolism	rs61798836	-0.02116692	0.039974019	0.596446896
eosinophil	ieu-b-33	pulmonary embolism	rs62005030	-0.020512236	0.039869779	0.606915876
eosinophil	ieu-b-33	pulmonary embolism	rs62006172	-0.019114144	0.03978497	0.630917184
eosinophil	ieu-b-33	pulmonary embolism	rs62011287	-0.02431027	0.039728255	0.54059475
eosinophil	ieu-b-33	pulmonary embolism	rs62061733	-0.020088964	0.039957771	0.615136617
eosinophil	ieu-b-33	pulmonary embolism	rs62086903	-0.0249167	0.039965989	0.532990429
eosinophil	ieu-b-33	pulmonary embolism	rs62105489	-0.021078218	0.039936225	0.597640279
eosinophil	ieu-b-33	pulmonary embolism	rs62117160	-0.020593702	0.039970253	0.606395314
eosinophil	ieu-b-33	pulmonary embolism	rs62183994	-0.023810251	0.039762875	0.549302216
eosinophil	ieu-b-33	pulmonary embolism	rs62308111	-0.019847194	0.039850068	0.618451145
eosinophil	ieu-b-33	pulmonary embolism	rs62385501	-0.022704865	0.039949316	0.569802923
eosinophil	ieu-b-33	pulmonary embolism	rs62395833	-0.023218992	0.039940343	0.561010097
eosinophil	ieu-b-33	pulmonary embolism	rs62408224	-0.017679444	0.040034725	0.6587767
eosinophil	ieu-b-33	pulmonary embolism	rs62473720	-0.020560644	0.039946132	0.606756202
eosinophil	ieu-b-33	pulmonary embolism	rs62539154	-0.024038932	0.039903826	0.546893451
eosinophil	ieu-b-33	pulmonary embolism	rs634534	-0.024961973	0.040017556	0.53277501
eosinophil	ieu-b-33	pulmonary embolism	rs637064	-0.018334749	0.03990479	0.645902174
eosinophil	ieu-b-33	pulmonary embolism	rs6490291	-0.02327715	0.040028379	0.560892618
eosinophil	ieu-b-33	pulmonary embolism	rs6496717	-0.01869917	0.039806511	0.63853253
eosinophil	ieu-b-33	pulmonary embolism	rs6556313	-0.020013412	0.039958439	0.616472678
eosinophil	ieu-b-33	pulmonary embolism	rs6573020	-0.02146348	0.040014399	0.591686342
eosinophil	ieu-b-33	pulmonary embolism	rs6670664	-0.02112801	0.03998561	0.597228451
eosinophil	ieu-b-33	pulmonary embolism	rs66735324	-0.022964084	0.039932925	0.565246388
eosinophil	ieu-b-33	pulmonary embolism	rs668248	-0.020095467	0.039930466	0.614780407
eosinophil	ieu-b-33	pulmonary embolism	rs6684992	-0.022617373	0.040030464	0.57207104
eosinophil	ieu-b-33	pulmonary embolism	rs6731125	-0.020111099	0.039946976	0.614651559
eosinophil	ieu-b-33	pulmonary embolism	rs6741307	-0.023348087	0.039901373	0.558450248
eosinophil	ieu-b-33	pulmonary embolism	rs6750754	-0.023251327	0.040292481	0.563896437
eosinophil	ieu-b-33	pulmonary embolism	rs67856193	-0.018070639	0.03986238	0.650314275
eosinophil	ieu-b-33	pulmonary embolism	rs6787336	-0.017289366	0.039958471	0.665244819
eosinophil	ieu-b-33	pulmonary embolism	rs6904506	-0.019094194	0.039986919	0.63299828
eosinophil	ieu-b-33	pulmonary embolism	rs6924350	-0.030922658	0.039858801	0.437864111
eosinophil	ieu-b-33	pulmonary embolism	rs6924387	-0.019772283	0.039916264	0.620357298
eosinophil	ieu-b-33	pulmonary embolism	rs6930635	-0.023650845	0.039958835	0.553930783
eosinophil	ieu-b-33	pulmonary embolism	rs6956283	-0.020820731	0.039962708	0.602363976
eosinophil	ieu-b-33	pulmonary embolism	rs6971710	-0.024687866	0.039932462	0.536416802
eosinophil	ieu-b-33	pulmonary embolism	rs6979947	-0.020819563	0.039950509	0.602273514
eosinophil	ieu-b-33	pulmonary embolism	rs6986109	-0.02180358	0.039993577	0.58563176
eosinophil	ieu-b-33	pulmonary embolism	rs6989099	-0.020941565	0.039984211	0.600455308
eosinophil	ieu-b-33	pulmonary embolism	rs699664	-0.023402218	0.039915421	0.557677189
eosinophil	ieu-b-33	pulmonary embolism	rs6999452	-0.021255443	0.039975748	0.594927935
eosinophil	ieu-b-33	pulmonary embolism	rs7009579	-0.021970821	0.039986125	0.582689043
eosinophil	ieu-b-33	pulmonary embolism	rs7026022	-0.021752599	0.039986633	0.586443698

eosinophil	ieu-b-33	pulmonary embolism	rs7080536	-0.021983298	0.039992205	0.582532307
eosinophil	ieu-b-33	pulmonary embolism	rs708776	-0.02135076	0.039978312	0.593301017
eosinophil	ieu-b-33	pulmonary embolism	rs7123726	-0.020513325	0.039939149	0.607521567
eosinophil	ieu-b-33	pulmonary embolism	rs7141943	-0.022740855	0.039971959	0.569410291
eosinophil	ieu-b-33	pulmonary embolism	rs71429414	-0.020936985	0.039971444	0.60041865
eosinophil	ieu-b-33	pulmonary embolism	rs71508968	-0.021017607	0.039950181	0.598821909
eosinophil	ieu-b-33	pulmonary embolism	rs7158239	-0.020976092	0.039982555	0.599839661
eosinophil	ieu-b-33	pulmonary embolism	rs71628184	-0.023306771	0.039868174	0.558819469
eosinophil	ieu-b-33	pulmonary embolism	rs7173571	-0.020758393	0.039964322	0.603465718
eosinophil	ieu-b-33	pulmonary embolism	rs7215391	-0.022384002	0.039959481	0.575365204
eosinophil	ieu-b-33	pulmonary embolism	rs7220649	-0.021247115	0.039959813	0.594925429
eosinophil	ieu-b-33	pulmonary embolism	rs725613	-0.02198081	0.040171622	0.584260169
eosinophil	ieu-b-33	pulmonary embolism	rs7257	-0.023570603	0.040067048	0.55634504
eosinophil	ieu-b-33	pulmonary embolism	rs7267752	-0.022775678	0.039916535	0.568282654
eosinophil	ieu-b-33	pulmonary embolism	rs72695582	-0.021257029	0.040007386	0.59519177
eosinophil	ieu-b-33	pulmonary embolism	rs72766638	-0.025804352	0.039745662	0.516185232
eosinophil	ieu-b-33	pulmonary embolism	rs72800841	-0.022313301	0.039979876	0.576767009
eosinophil	ieu-b-33	pulmonary embolism	rs72834751	-0.021476347	0.040008791	0.591412201
eosinophil	ieu-b-33	pulmonary embolism	rs72987040	-0.020763969	0.039974949	0.60346474
eosinophil	ieu-b-33	pulmonary embolism	rs73046033	-0.021179602	0.039980206	0.59628376
eosinophil	ieu-b-33	pulmonary embolism	rs73072498	-0.020664279	0.039913221	0.604646536
eosinophil	ieu-b-33	pulmonary embolism	rs73086541	-0.021956896	0.039985204	0.5829193
eosinophil	ieu-b-33	pulmonary embolism	rs73118830	-0.022196016	0.040028097	0.579229243
eosinophil	ieu-b-33	pulmonary embolism	rs73191842	-0.02251882	0.039830772	0.571827315
eosinophil	ieu-b-33	pulmonary embolism	rs73205140	-0.023083194	0.039999528	0.563880916
eosinophil	ieu-b-33	pulmonary embolism	rs73232881	-0.022845963	0.040234692	0.570158886
eosinophil	ieu-b-33	pulmonary embolism	rs73238201	-0.02217327	0.039981981	0.57918089
eosinophil	ieu-b-33	pulmonary embolism	rs73272842	-0.019911076	0.039934223	0.618063446
eosinophil	ieu-b-33	pulmonary embolism	rs7327960	-0.020905658	0.039960817	0.600867155
eosinophil	ieu-b-33	pulmonary embolism	rs73322872	-0.019630165	0.039792001	0.621786949
eosinophil	ieu-b-33	pulmonary embolism	rs73428834	-0.023707607	0.039939847	0.552791174
eosinophil	ieu-b-33	pulmonary embolism	rs7382061	-0.028619862	0.040180422	0.476289085
eosinophil	ieu-b-33	pulmonary embolism	rs73963711	-0.021681072	0.040016446	0.587953511
eosinophil	ieu-b-33	pulmonary embolism	rs7423615	-0.021104807	0.039988535	0.597657967
eosinophil	ieu-b-33	pulmonary embolism	rs743002	-0.022417466	0.040020936	0.575381576
eosinophil	ieu-b-33	pulmonary embolism	rs74331768	-0.022119947	0.039973481	0.580013128
eosinophil	ieu-b-33	pulmonary embolism	rs7441808	-0.02223504	0.039979474	0.578100532
eosinophil	ieu-b-33	pulmonary embolism	rs74480102	-0.022067475	0.040028477	0.581431889
eosinophil	ieu-b-33	pulmonary embolism	rs74612091	-0.015957731	0.039854102	0.688859183
eosinophil	ieu-b-33	pulmonary embolism	rs746550	-0.022116636	0.039995331	0.580276795
eosinophil	ieu-b-33	pulmonary embolism	rs7556106	-0.021899498	0.039995319	0.583999878
eosinophil	ieu-b-33	pulmonary embolism	rs7569084	-0.021876992	0.040005084	0.584478157
eosinophil	ieu-b-33	pulmonary embolism	rs75737219	-0.023449266	0.040018158	0.557898603
eosinophil	ieu-b-33	pulmonary embolism	rs7591231	-0.021791452	0.039983541	0.58574626

eosinophil	ieu-b-33	pulmonary embolism	rs7593992	-0.019997396	0.040012624	0.617231989
eosinophil	ieu-b-33	pulmonary embolism	rs7636495	-0.023313362	0.039965788	0.559669171
eosinophil	ieu-b-33	pulmonary embolism	rs7646283	-0.020889046	0.040025327	0.601742955
eosinophil	ieu-b-33	pulmonary embolism	rs7646695	-0.021926998	0.03999052	0.583482568
eosinophil	ieu-b-33	pulmonary embolism	rs7649812	-0.020467162	0.039948193	0.608411245
eosinophil	ieu-b-33	pulmonary embolism	rs76793172	-0.022473838	0.040058443	0.574779644
eosinophil	ieu-b-33	pulmonary embolism	rs76830965	-0.020943645	0.039968409	0.600275077
eosinophil	ieu-b-33	pulmonary embolism	rs7687708	-0.021438504	0.03998392	0.591835439
eosinophil	ieu-b-33	pulmonary embolism	rs76908370	-0.022438587	0.039977774	0.574609029
eosinophil	ieu-b-33	pulmonary embolism	rs76981581	-0.022569555	0.039981706	0.572415913
eosinophil	ieu-b-33	pulmonary embolism	rs7700687	-0.018930112	0.040086358	0.636760298
eosinophil	ieu-b-33	pulmonary embolism	rs77331334	-0.022166525	0.039966988	0.57915403
eosinophil	ieu-b-33	pulmonary embolism	rs77625297	-0.022910292	0.03993733	0.566200559
eosinophil	ieu-b-33	pulmonary embolism	rs7764278	-0.021208893	0.0399846	0.595816231
eosinophil	ieu-b-33	pulmonary embolism	rs778756	-0.020583554	0.039968039	0.606552771
eosinophil	ieu-b-33	pulmonary embolism	rs7797428	-0.022244002	0.039977966	0.57793297
eosinophil	ieu-b-33	pulmonary embolism	rs783646	-0.020652076	0.039977733	0.605442775
eosinophil	ieu-b-33	pulmonary embolism	rs7839946	-0.021700446	0.039986487	0.587340168
eosinophil	ieu-b-33	pulmonary embolism	rs7840212	-0.023474204	0.040094428	0.558229725
eosinophil	ieu-b-33	pulmonary embolism	rs7846314	-0.021962167	0.040040413	0.583348624
eosinophil	ieu-b-33	pulmonary embolism	rs7868130	-0.018112625	0.039982215	0.650536466
eosinophil	ieu-b-33	pulmonary embolism	rs7897422	-0.023858214	0.039935824	0.55023111
eosinophil	ieu-b-33	pulmonary embolism	rs79716587	-0.020380998	0.039933127	0.609786511
eosinophil	ieu-b-33	pulmonary embolism	rs7975	-0.022879471	0.039931556	0.566666917
eosinophil	ieu-b-33	pulmonary embolism	rs7986796	-0.023459505	0.039996043	0.557508891
eosinophil	ieu-b-33	pulmonary embolism	rs79881201	-0.019786506	0.040097091	0.62168415
eosinophil	ieu-b-33	pulmonary embolism	rs8	-0.022075303	0.040005338	0.581079263
eosinophil	ieu-b-33	pulmonary embolism	rs80054178	-0.022671286	0.039973653	0.570608387
eosinophil	ieu-b-33	pulmonary embolism	rs80066203	-0.021918708	0.039980584	0.583531371
eosinophil	ieu-b-33	pulmonary embolism	rs8012643	-0.021376309	0.039987384	0.592942863
eosinophil	ieu-b-33	pulmonary embolism	rs8020739	-0.022984019	0.040009149	0.565650426
eosinophil	ieu-b-33	pulmonary embolism	rs8026803	-0.020815107	0.040033936	0.60310778
eosinophil	ieu-b-33	pulmonary embolism	rs8044920	-0.024075996	0.039746597	0.544689218
eosinophil	ieu-b-33	pulmonary embolism	rs8050508	-0.021866932	0.040012386	0.584719494
eosinophil	ieu-b-33	pulmonary embolism	rs8076052	-0.019113874	0.039823117	0.631249224
eosinophil	ieu-b-33	pulmonary embolism	rs8083368	-0.02090365	0.039972908	0.601012218
eosinophil	ieu-b-33	pulmonary embolism	rs8113367	-0.022992097	0.039963091	0.565065902
eosinophil	ieu-b-33	pulmonary embolism	rs884634	-0.021172321	0.039976274	0.596373927
eosinophil	ieu-b-33	pulmonary embolism	rs900382	-0.022596138	0.040019853	0.572330077
eosinophil	ieu-b-33	pulmonary embolism	rs911603	-0.02076349	0.040024451	0.603921047
eosinophil	ieu-b-33	pulmonary embolism	rs925966	-0.020508549	0.039971949	0.607899882
eosinophil	ieu-b-33	pulmonary embolism	rs9349322	-0.022105565	0.04000212	0.580530612
eosinophil	ieu-b-33	pulmonary embolism	rs9389268	-0.015794069	0.040044047	0.693272889
eosinophil	ieu-b-33	pulmonary embolism	rs9392525	-0.019734477	0.039851815	0.620461453

eosinophil	ieu-b-33	pulmonary embolism	rs941616	-0.021770918	0.039986839	0.586130408
eosinophil	ieu-b-33	pulmonary embolism	rs9428321	-0.022882469	0.039955469	0.566848315
eosinophil	ieu-b-33	pulmonary embolism	rs9430574	-0.022005871	0.040009904	0.58231211
eosinophil	ieu-b-33	pulmonary embolism	rs954954	-0.02064075	0.040012244	0.605951607
eosinophil	ieu-b-33	pulmonary embolism	rs962993	-0.014014492	0.039859918	0.725143064
eosinophil	ieu-b-33	pulmonary embolism	rs964184	-0.021304292	0.040012347	0.594419252
eosinophil	ieu-b-33	pulmonary embolism	rs9666598	-0.022304655	0.039987266	0.576985097
eosinophil	ieu-b-33	pulmonary embolism	rs9675999	-0.02100711	0.039985793	0.599329928
eosinophil	ieu-b-33	pulmonary embolism	rs9815874	-0.024457228	0.039946075	0.540368322
eosinophil	ieu-b-33	pulmonary embolism	rs9818987	-0.021297825	0.039985328	0.594282112
eosinophil	ieu-b-33	pulmonary embolism	rs9835307	-0.021963133	0.040034485	0.583276299
eosinophil	ieu-b-33	pulmonary embolism	rs9837045	-0.021796439	0.039986047	0.585683964
eosinophil	ieu-b-33	pulmonary embolism	rs9840310	-0.022972678	0.039911054	0.564887695
eosinophil	ieu-b-33	pulmonary embolism	rs9872485	-0.022871844	0.039963307	0.567104452
eosinophil	ieu-b-33	pulmonary embolism	rs9894839	-0.022802184	0.039938824	0.568048591
eosinophil	ieu-b-33	pulmonary embolism	rs9939774	-0.022462739	0.040049184	0.574880158
eosinophil	ieu-b-33	pulmonary embolism	rs9965489	-0.022291278	0.039992789	0.577266164
eosinophil	ieu-b-33	pulmonary embolism	rs9979383	-0.019847909	0.040017926	0.619911721
eosinophil	ieu-b-33	pulmonary embolism	All	-0.021726398	0.039926372	0.586330287
neutrophil	ieu-b-34	pulmonary embolism	rs10252457	-0.126990812	0.04390657	0.00382437
neutrophil	ieu-b-34	pulmonary embolism	rs1042725	-0.127216949	0.043908435	0.003763612
neutrophil	ieu-b-34	pulmonary embolism	rs10447304	-0.128327104	0.04392134	0.003480726
neutrophil	ieu-b-34	pulmonary embolism	rs1047891	-0.123470903	0.043709582	0.004730988
neutrophil	ieu-b-34	pulmonary embolism	rs10498635	-0.124931196	0.043880393	0.004412162
neutrophil	ieu-b-34	pulmonary embolism	rs10509912	-0.127070497	0.043917613	0.003811157
neutrophil	ieu-b-34	pulmonary embolism	rs10750623	-0.128669683	0.043864877	0.00335359
neutrophil	ieu-b-34	pulmonary embolism	rs10760690	-0.128164948	0.043895742	0.003503032
neutrophil	ieu-b-34	pulmonary embolism	rs10786325	-0.122765616	0.044095066	0.005367445
neutrophil	ieu-b-34	pulmonary embolism	rs10833024	-0.127817613	0.043916308	0.00360872
neutrophil	ieu-b-34	pulmonary embolism	rs10846411	-0.127889585	0.043924199	0.003595857
neutrophil	ieu-b-34	pulmonary embolism	rs10849020	-0.127725338	0.043946205	0.003656114
neutrophil	ieu-b-34	pulmonary embolism	rs10864368	-0.129362514	0.043949506	0.00324592
neutrophil	ieu-b-34	pulmonary embolism	rs10868825	-0.12826583	0.04389245	0.00347484
neutrophil	ieu-b-34	pulmonary embolism	rs10945542	-0.12736325	0.043907679	0.003723235
neutrophil	ieu-b-34	pulmonary embolism	rs10948036	-0.125796459	0.043959839	0.004214788
neutrophil	ieu-b-34	pulmonary embolism	rs10992394	-0.127962064	0.043903704	0.003561362
neutrophil	ieu-b-34	pulmonary embolism	rs10995477	-0.126987969	0.044025745	0.003921572
neutrophil	ieu-b-34	pulmonary embolism	rs11039195	-0.123899213	0.043730079	0.004607415
neutrophil	ieu-b-34	pulmonary embolism	rs11064881	-0.127582508	0.043942957	0.003691744
neutrophil	ieu-b-34	pulmonary embolism	rs11080133	-0.126511715	0.043861252	0.00392208
neutrophil	ieu-b-34	pulmonary embolism	rs11104881	-0.134907528	0.043630793	0.001987957
neutrophil	ieu-b-34	pulmonary embolism	rs112203773	-0.129233572	0.04361474	0.003045863
neutrophil	ieu-b-34	pulmonary embolism	rs1135172	-0.125748932	0.043771695	0.004068044
neutrophil	ieu-b-34	pulmonary embolism	rs114050631	-0.130206034	0.04414551	0.003183153

neutrophil	ieu-b-34	pulmonary embolism	rs114427170	-0.128306835	0.043909181	0.003476845
neutrophil	ieu-b-34	pulmonary embolism	rs11580229	-0.131226925	0.043853719	0.002768128
neutrophil	ieu-b-34	pulmonary embolism	rs1159649	-0.13042287	0.043704114	0.002842984
neutrophil	ieu-b-34	pulmonary embolism	rs11625865	-0.128900651	0.043888214	0.003313806
neutrophil	ieu-b-34	pulmonary embolism	rs116447416	-0.130054026	0.043737482	0.002944107
neutrophil	ieu-b-34	pulmonary embolism	rs11648664	-0.125071307	0.04377585	0.004275519
neutrophil	ieu-b-34	pulmonary embolism	rs116631966	-0.12755692	0.043894607	0.00366103
neutrophil	ieu-b-34	pulmonary embolism	rs11664534	-0.128550154	0.043911282	0.003417022
neutrophil	ieu-b-34	pulmonary embolism	rs11673093	-0.121676158	0.044113654	0.005811306
neutrophil	ieu-b-34	pulmonary embolism	rs11683933	-0.127144038	0.043894929	0.003772855
neutrophil	ieu-b-34	pulmonary embolism	rs11689257	-0.127027669	0.043907473	0.003814886
neutrophil	ieu-b-34	pulmonary embolism	rs11690365	-0.126835377	0.043885533	0.003850691
neutrophil	ieu-b-34	pulmonary embolism	rs11691193	-0.129143585	0.043841131	0.003222066
neutrophil	ieu-b-34	pulmonary embolism	rs11712552	-0.127311427	0.043940675	0.00376332
neutrophil	ieu-b-34	pulmonary embolism	rs11716015	-0.128632132	0.04387546	0.003370517
neutrophil	ieu-b-34	pulmonary embolism	rs11721064	-0.128553115	0.043894307	0.003403864
neutrophil	ieu-b-34	pulmonary embolism	rs11723621	-0.131842654	0.043792896	0.002607367
neutrophil	ieu-b-34	pulmonary embolism	rs11735662	-0.128871872	0.043883295	0.003317297
neutrophil	ieu-b-34	pulmonary embolism	rs117468663	-0.125029745	0.043724127	0.00424291
neutrophil	ieu-b-34	pulmonary embolism	rs11753041	-0.127514322	0.043906573	0.0036817
neutrophil	ieu-b-34	pulmonary embolism	rs11894199	-0.126207164	0.043824642	0.003978999
neutrophil	ieu-b-34	pulmonary embolism	rs11997631	-0.13039405	0.043785007	0.002900863
neutrophil	ieu-b-34	pulmonary embolism	rs12118277	-0.12673594	0.043897954	0.003888625
neutrophil	ieu-b-34	pulmonary embolism	rs12189880	-0.129018903	0.043876798	0.003277028
neutrophil	ieu-b-34	pulmonary embolism	rs12203592	-0.127870797	0.04387697	0.003564834
neutrophil	ieu-b-34	pulmonary embolism	rs12214269	-0.126182644	0.0439163	0.004062685
neutrophil	ieu-b-34	pulmonary embolism	rs12328347	-0.124699987	0.043779106	0.004394098
neutrophil	ieu-b-34	pulmonary embolism	rs12378064	-0.127909051	0.043910133	0.00358005
neutrophil	ieu-b-34	pulmonary embolism	rs1245035	-0.127147113	0.043924402	0.003795446
neutrophil	ieu-b-34	pulmonary embolism	rs12460279	-0.129762688	0.043747651	0.003015401
neutrophil	ieu-b-34	pulmonary embolism	rs12481262	-0.127493684	0.043919889	0.003697613
neutrophil	ieu-b-34	pulmonary embolism	rs12487658	-0.127823621	0.043912068	0.003603898
neutrophil	ieu-b-34	pulmonary embolism	rs1250568	-0.129480217	0.043837314	0.003140368
neutrophil	ieu-b-34	pulmonary embolism	rs12530608	-0.128818836	0.043833867	0.003294888
neutrophil	ieu-b-34	pulmonary embolism	rs12541521	-0.128432951	0.043889683	0.003430559
neutrophil	ieu-b-34	pulmonary embolism	rs12550612	-0.130508248	0.043935991	0.00297395
neutrophil	ieu-b-34	pulmonary embolism	rs1260326	-0.126981481	0.044031002	0.0039277
neutrophil	ieu-b-34	pulmonary embolism	rs12658947	-0.12977731	0.04377083	0.003027549
neutrophil	ieu-b-34	pulmonary embolism	rs12716647	-0.128971553	0.04387234	0.003285293
neutrophil	ieu-b-34	pulmonary embolism	rs12802157	-0.126997135	0.043894612	0.00381304
neutrophil	ieu-b-34	pulmonary embolism	rs12909505	-0.125602435	0.043916579	0.0042361
neutrophil	ieu-b-34	pulmonary embolism	rs12923918	-0.127223627	0.043898062	0.003753585
neutrophil	ieu-b-34	pulmonary embolism	rs12927351	-0.127621134	0.043932079	0.003672946
neutrophil	ieu-b-34	pulmonary embolism	rs12930850	-0.131227258	0.043780986	0.002723313

neutrophil	ieu-b-34	pulmonary embolism	rs13190036	-0.126792701	0.04392023	0.003890751
neutrophil	ieu-b-34	pulmonary embolism	rs13291664	-0.127592262	0.043944169	0.003690071
neutrophil	ieu-b-34	pulmonary embolism	rs13392977	-0.128417286	0.04387374	0.003422778
neutrophil	ieu-b-34	pulmonary embolism	rs1362623	-0.126946451	0.043909887	0.003839355
neutrophil	ieu-b-34	pulmonary embolism	rs1366045	-0.126324014	0.043936998	0.004038777
neutrophil	ieu-b-34	pulmonary embolism	rs1371794	-0.128911631	0.044603839	0.003850623
neutrophil	ieu-b-34	pulmonary embolism	rs140311179	-0.125296045	0.043723933	0.004161974
neutrophil	ieu-b-34	pulmonary embolism	rs141010613	-0.125837446	0.043722328	0.004000721
neutrophil	ieu-b-34	pulmonary embolism	rs1411424	-0.126995364	0.043895946	0.003814597
neutrophil	ieu-b-34	pulmonary embolism	rs1412445	-0.125633457	0.043914438	0.004224815
neutrophil	ieu-b-34	pulmonary embolism	rs14243	-0.127894827	0.043899743	0.003575863
neutrophil	ieu-b-34	pulmonary embolism	rs1474419	-0.128410365	0.043922261	0.003460282
neutrophil	ieu-b-34	pulmonary embolism	rs148713124	-0.128600787	0.043883777	0.003384324
neutrophil	ieu-b-34	pulmonary embolism	rs1490384	-0.126523886	0.043897205	0.00394813
neutrophil	ieu-b-34	pulmonary embolism	rs149157044	-0.129614286	0.043809483	0.003090503
neutrophil	ieu-b-34	pulmonary embolism	rs150861794	-0.127593697	0.043900471	0.003655773
neutrophil	ieu-b-34	pulmonary embolism	rs1555275	-0.127463923	0.043911394	0.003698985
neutrophil	ieu-b-34	pulmonary embolism	rs156334	-0.127542943	0.043908123	0.003675245
neutrophil	ieu-b-34	pulmonary embolism	rs1571956	-0.124940646	0.043712467	0.004260004
neutrophil	ieu-b-34	pulmonary embolism	rs1611236	-0.128649983	0.043907028	0.003389026
neutrophil	ieu-b-34	pulmonary embolism	rs16843353	-0.124904934	0.043733676	0.00428967
neutrophil	ieu-b-34	pulmonary embolism	rs16850073	-0.130305012	0.044266507	0.003243688
neutrophil	ieu-b-34	pulmonary embolism	rs16939607	-0.128941155	0.04393215	0.003335405
neutrophil	ieu-b-34	pulmonary embolism	rs16958642	-0.126633681	0.043882953	0.00390526
neutrophil	ieu-b-34	pulmonary embolism	rs16961474	-0.129878994	0.043704275	0.002960897
neutrophil	ieu-b-34	pulmonary embolism	rs17026212	-0.128170617	0.043924554	0.00352316
neutrophil	ieu-b-34	pulmonary embolism	rs17139597	-0.126194052	0.043888897	0.004036348
neutrophil	ieu-b-34	pulmonary embolism	rs17213043	-0.128271044	0.043891209	0.003472594
neutrophil	ieu-b-34	pulmonary embolism	rs172240	-0.126542277	0.043888673	0.003935875
neutrophil	ieu-b-34	pulmonary embolism	rs17381821	-0.124937603	0.043678071	0.00423081
neutrophil	ieu-b-34	pulmonary embolism	rs17440390	-0.128923653	0.043838906	0.003273128
neutrophil	ieu-b-34	pulmonary embolism	rs174548	-0.135614324	0.043463993	0.001807569
neutrophil	ieu-b-34	pulmonary embolism	rs17728	-0.126871002	0.043899941	0.00385237
neutrophil	ieu-b-34	pulmonary embolism	rs17742008	-0.127655755	0.043919481	0.003653963
neutrophil	ieu-b-34	pulmonary embolism	rs17831500	-0.129704334	0.043866006	0.003108191
neutrophil	ieu-b-34	pulmonary embolism	rs1800961	-0.126109335	0.043938181	0.004102722
neutrophil	ieu-b-34	pulmonary embolism	rs180941	-0.128059543	0.0439	0.003533321
neutrophil	ieu-b-34	pulmonary embolism	rs1885474	-0.128043633	0.043931905	0.003561524
neutrophil	ieu-b-34	pulmonary embolism	rs1886654	-0.124812688	0.044018826	0.004576278
neutrophil	ieu-b-34	pulmonary embolism	rs1887428	-0.126913569	0.043906293	0.003845618
neutrophil	ieu-b-34	pulmonary embolism	rs1939212	-0.130004269	0.043762584	0.002971492
neutrophil	ieu-b-34	pulmonary embolism	rs1966479	-0.12762995	0.043915987	0.003658127
neutrophil	ieu-b-34	pulmonary embolism	rs1977289	-0.129301371	0.043897397	0.003223954
neutrophil	ieu-b-34	pulmonary embolism	rs1985157	-0.131782532	0.043884515	0.002673858

neutrophil	ieu-b-34	pulmonary embolism	rs2001613	-0.124724811	0.043654891	0.004275798
neutrophil	ieu-b-34	pulmonary embolism	rs2038700	-0.131224046	0.04399655	0.002858126
neutrophil	ieu-b-34	pulmonary embolism	rs2046934	-0.126325637	0.043849181	0.003965261
neutrophil	ieu-b-34	pulmonary embolism	rs2052249	-0.127833008	0.043904505	0.003595657
neutrophil	ieu-b-34	pulmonary embolism	rs2062250	-0.12745026	0.04396334	0.003743403
neutrophil	ieu-b-34	pulmonary embolism	rs207253	-0.126181353	0.043844047	0.004002577
neutrophil	ieu-b-34	pulmonary embolism	rs2082382	-0.132478441	0.043881705	0.002536187
neutrophil	ieu-b-34	pulmonary embolism	rs212409	-0.127826409	0.043953659	0.003635058
neutrophil	ieu-b-34	pulmonary embolism	rs2157770	-0.124141191	0.043832284	0.004623092
neutrophil	ieu-b-34	pulmonary embolism	rs2179593	-0.128456005	0.043881609	0.003418845
neutrophil	ieu-b-34	pulmonary embolism	rs218264	-0.122676899	0.043862442	0.005160266
neutrophil	ieu-b-34	pulmonary embolism	rs2241621	-0.125069384	0.043797334	0.004295029
neutrophil	ieu-b-34	pulmonary embolism	rs2254458	-0.126323563	0.043872838	0.003985446
neutrophil	ieu-b-34	pulmonary embolism	rs2260850	-0.126453922	0.043951038	0.004012792
neutrophil	ieu-b-34	pulmonary embolism	rs2282611	-0.12629239	0.043856708	0.003981048
neutrophil	ieu-b-34	pulmonary embolism	rs2290846	-0.12821035	0.043918588	0.003508488
neutrophil	ieu-b-34	pulmonary embolism	rs2296028	-0.128204775	0.043897863	0.00349443
neutrophil	ieu-b-34	pulmonary embolism	rs2301557	-0.133423906	0.044046035	0.002452086
neutrophil	ieu-b-34	pulmonary embolism	rs2312675	-0.128055568	0.043919183	0.00354881
neutrophil	ieu-b-34	pulmonary embolism	rs2314339	-0.130324301	0.043933754	0.003013259
neutrophil	ieu-b-34	pulmonary embolism	rs2315008	-0.129539361	0.04384223	0.003130027
neutrophil	ieu-b-34	pulmonary embolism	rs2421200	-0.127320232	0.043949383	0.003767807
neutrophil	ieu-b-34	pulmonary embolism	rs2432142	-0.123156323	0.043520773	0.004657296
neutrophil	ieu-b-34	pulmonary embolism	rs2468832	-0.127176478	0.043910205	0.00377609
neutrophil	ieu-b-34	pulmonary embolism	rs2494747	-0.125054801	0.043737403	0.004246859
neutrophil	ieu-b-34	pulmonary embolism	rs249677	-0.124787615	0.043884793	0.004461716
neutrophil	ieu-b-34	pulmonary embolism	rs2522051	-0.131520162	0.043855373	0.002709099
neutrophil	ieu-b-34	pulmonary embolism	rs2522440	-0.12793323	0.043903509	0.003568714
neutrophil	ieu-b-34	pulmonary embolism	rs2561758	-0.123871287	0.043962645	0.004837672
neutrophil	ieu-b-34	pulmonary embolism	rs2632372	-0.128008802	0.043952209	0.003586006
neutrophil	ieu-b-34	pulmonary embolism	rs2665405	-0.127559194	0.044019177	0.003757786
neutrophil	ieu-b-34	pulmonary embolism	rs2710804	-0.131283972	0.043777975	0.002709928
neutrophil	ieu-b-34	pulmonary embolism	rs2729707	-0.129313714	0.043998936	0.003292528
neutrophil	ieu-b-34	pulmonary embolism	rs2734031	-0.123096594	0.044046052	0.005194391
neutrophil	ieu-b-34	pulmonary embolism	rs2807742	-0.122323524	0.043814274	0.005240527
neutrophil	ieu-b-34	pulmonary embolism	rs2808519	-0.126524775	0.043882936	0.003936149
neutrophil	ieu-b-34	pulmonary embolism	rs2824372	-0.126596569	0.043889225	0.003920897
neutrophil	ieu-b-34	pulmonary embolism	rs2839183	-0.127742368	0.043910631	0.003624194
neutrophil	ieu-b-34	pulmonary embolism	rs284317	-0.127577138	0.043914482	0.003671044
neutrophil	ieu-b-34	pulmonary embolism	rs2852800	-0.128994796	0.043862217	0.003272505
neutrophil	ieu-b-34	pulmonary embolism	rs28530750	-0.128878246	0.043913161	0.003337162
neutrophil	ieu-b-34	pulmonary embolism	rs28540102	-0.126103148	0.043883076	0.004058009
neutrophil	ieu-b-34	pulmonary embolism	rs28571765	-0.125664892	0.043896779	0.004200002
neutrophil	ieu-b-34	pulmonary embolism	rs28588142	-0.126878593	0.043891407	0.003843374

neutrophil	ieu-b-34	pulmonary embolism	rs2923416	-0.128464515	0.043877925	0.003414014
neutrophil	ieu-b-34	pulmonary embolism	rs2955958	-0.128202292	0.043892539	0.003491093
neutrophil	ieu-b-34	pulmonary embolism	rs3014874	-0.130045851	0.043674903	0.002905266
neutrophil	ieu-b-34	pulmonary embolism	rs301817	-0.127542158	0.043914785	0.003680633
neutrophil	ieu-b-34	pulmonary embolism	rs303753	-0.126372053	0.043945214	0.004031675
neutrophil	ieu-b-34	pulmonary embolism	rs305082	-0.129827521	0.044016095	0.003182406
neutrophil	ieu-b-34	pulmonary embolism	rs3184504	-0.133955145	0.043808099	0.002229897
neutrophil	ieu-b-34	pulmonary embolism	rs33951980	-0.1265922	0.043927384	0.00395346
neutrophil	ieu-b-34	pulmonary embolism	rs34215892	-0.128181178	0.043900907	0.003502738
neutrophil	ieu-b-34	pulmonary embolism	rs342242	-0.1260342	0.043882664	0.004077892
neutrophil	ieu-b-34	pulmonary embolism	rs34599082	-0.122270056	0.043838134	0.005285059
neutrophil	ieu-b-34	pulmonary embolism	rs34765661	-0.131222816	0.043897126	0.002795937
neutrophil	ieu-b-34	pulmonary embolism	rs34990593	-0.1267847	0.043893079	0.003870969
neutrophil	ieu-b-34	pulmonary embolism	rs35020138	-0.127696038	0.043928263	0.003650041
neutrophil	ieu-b-34	pulmonary embolism	rs35284073	-0.130186216	0.043714802	0.002900594
neutrophil	ieu-b-34	pulmonary embolism	rs354703	-0.127053474	0.043912737	0.003811962
neutrophil	ieu-b-34	pulmonary embolism	rs35734242	-0.128851949	0.043956798	0.003375124
neutrophil	ieu-b-34	pulmonary embolism	rs35759345	-0.12615561	0.043851475	0.004016231
neutrophil	ieu-b-34	pulmonary embolism	rs35789178	-0.126856021	0.043899106	0.00385588
neutrophil	ieu-b-34	pulmonary embolism	rs35864914	-0.127511439	0.04390584	0.003681903
neutrophil	ieu-b-34	pulmonary embolism	rs35915186	-0.132040103	0.043751561	0.002544893
neutrophil	ieu-b-34	pulmonary embolism	rs35929659	-0.126704142	0.043906466	0.003904523
neutrophil	ieu-b-34	pulmonary embolism	rs3731332	-0.126100971	0.044031269	0.004184655
neutrophil	ieu-b-34	pulmonary embolism	rs3735311	-0.128907223	0.043913395	0.00333024
neutrophil	ieu-b-34	pulmonary embolism	rs3735485	-0.130927602	0.043820218	0.002809608
neutrophil	ieu-b-34	pulmonary embolism	rs3739873	-0.127294218	0.043908706	0.003742766
neutrophil	ieu-b-34	pulmonary embolism	rs3747869	-0.125687852	0.043960295	0.004248152
neutrophil	ieu-b-34	pulmonary embolism	rs3749440	-0.131003463	0.04372773	0.002736453
neutrophil	ieu-b-34	pulmonary embolism	rs3754224	-0.126381047	0.04389808	0.003989823
neutrophil	ieu-b-34	pulmonary embolism	rs3762297	-0.125769155	0.043906154	0.004176688
neutrophil	ieu-b-34	pulmonary embolism	rs3777755	-0.124039751	0.04355081	0.004397257
neutrophil	ieu-b-34	pulmonary embolism	rs3781454	-0.126427058	0.043962829	0.004030411
neutrophil	ieu-b-34	pulmonary embolism	rs3793537	-0.126409011	0.043852035	0.003943755
neutrophil	ieu-b-34	pulmonary embolism	rs3843301	-0.12875742	0.043917184	0.003369785
neutrophil	ieu-b-34	pulmonary embolism	rs3856364	-0.12828579	0.0439062	0.003479983
neutrophil	ieu-b-34	pulmonary embolism	rs3861100	-0.129852049	0.043843621	0.003059367
neutrophil	ieu-b-34	pulmonary embolism	rs386243	-0.131707657	0.043870537	0.002680454
neutrophil	ieu-b-34	pulmonary embolism	rs4074882	-0.132206007	0.04371297	0.002491174
neutrophil	ieu-b-34	pulmonary embolism	rs409801	-0.129382107	0.043922235	0.003222158
neutrophil	ieu-b-34	pulmonary embolism	rs41272536	-0.127945767	0.043913121	0.003572744
neutrophil	ieu-b-34	pulmonary embolism	rs41313381	-0.12861326	0.043904628	0.003396409
neutrophil	ieu-b-34	pulmonary embolism	rs41433144	-0.12778699	0.043913582	0.003614691
neutrophil	ieu-b-34	pulmonary embolism	rs4145952	-0.12655514	0.043882773	0.003927377
neutrophil	ieu-b-34	pulmonary embolism	rs4276676	-0.127451087	0.043933334	0.003719608

neutrophil	ieu-b-34	pulmonary embolism	rs4347951	-0.129289196	0.043787437	0.003150539
neutrophil	ieu-b-34	pulmonary embolism	rs4413892	-0.128447944	0.044013927	0.003518917
neutrophil	ieu-b-34	pulmonary embolism	rs4468717	-0.125945524	0.043890218	0.00411046
neutrophil	ieu-b-34	pulmonary embolism	rs4535497	-0.127146956	0.043912657	0.003786139
neutrophil	ieu-b-34	pulmonary embolism	rs45577137	-0.126260793	0.043917562	0.004040916
neutrophil	ieu-b-34	pulmonary embolism	rs4626924	-0.127711057	0.043939581	0.003654792
neutrophil	ieu-b-34	pulmonary embolism	rs4632345	-0.12785396	0.04390671	0.00359185
neutrophil	ieu-b-34	pulmonary embolism	rs464609	-0.129111395	0.043819611	0.003214657
neutrophil	ieu-b-34	pulmonary embolism	rs4727392	-0.126578127	0.043899204	0.003934314
neutrophil	ieu-b-34	pulmonary embolism	rs4729046	-0.12846757	0.043969675	0.003480918
neutrophil	ieu-b-34	pulmonary embolism	rs47341	-0.127782241	0.043911015	0.003613975
neutrophil	ieu-b-34	pulmonary embolism	rs4734879	-0.131566744	0.043718088	0.002617341
neutrophil	ieu-b-34	pulmonary embolism	rs4760	-0.124764812	0.044350207	0.004905522
neutrophil	ieu-b-34	pulmonary embolism	rs4761234	-0.130261618	0.043889341	0.002997876
neutrophil	ieu-b-34	pulmonary embolism	rs4794820	-0.130721022	0.044775002	0.003505833
neutrophil	ieu-b-34	pulmonary embolism	rs4805881	-0.126259682	0.04387511	0.004005779
neutrophil	ieu-b-34	pulmonary embolism	rs4812447	-0.128181892	0.043926554	0.003521762
neutrophil	ieu-b-34	pulmonary embolism	rs4843073	-0.128736904	0.04388652	0.00335267
neutrophil	ieu-b-34	pulmonary embolism	rs4844622	-0.131092969	0.043808548	0.002767877
neutrophil	ieu-b-34	pulmonary embolism	rs486650	-0.128484514	0.043898856	0.003424354
neutrophil	ieu-b-34	pulmonary embolism	rs4903580	-0.129707234	0.0438596	0.003103176
neutrophil	ieu-b-34	pulmonary embolism	rs4909932	-0.128325041	0.043902104	0.00346697
neutrophil	ieu-b-34	pulmonary embolism	rs4924450	-0.126952427	0.043892785	0.003823959
neutrophil	ieu-b-34	pulmonary embolism	rs4925756	-0.126897862	0.043892323	0.003838746
neutrophil	ieu-b-34	pulmonary embolism	rs4984803	-0.126426409	0.043907448	0.003984527
neutrophil	ieu-b-34	pulmonary embolism	rs520910	-0.128242491	0.043885834	0.003475859
neutrophil	ieu-b-34	pulmonary embolism	rs55729107	-0.124031819	0.043652937	0.004492767
neutrophil	ieu-b-34	pulmonary embolism	rs55767800	-0.128742628	0.04386305	0.003334356
neutrophil	ieu-b-34	pulmonary embolism	rs55851397	-0.126390308	0.043866161	0.003960746
neutrophil	ieu-b-34	pulmonary embolism	rs55873273	-0.126230583	0.043898567	0.004033808
neutrophil	ieu-b-34	pulmonary embolism	rs55964818	-0.125274292	0.044066336	0.004471113
neutrophil	ieu-b-34	pulmonary embolism	rs56174170	-0.126857424	0.043880767	0.003840703
neutrophil	ieu-b-34	pulmonary embolism	rs56188865	-0.122025856	0.043798211	0.005334769
neutrophil	ieu-b-34	pulmonary embolism	rs56217149	-0.127070676	0.043906618	0.003802323
neutrophil	ieu-b-34	pulmonary embolism	rs56378716	-0.138620903	0.043950374	0.001610312
neutrophil	ieu-b-34	pulmonary embolism	rs56388170	-0.126474779	0.044493244	0.00447526
neutrophil	ieu-b-34	pulmonary embolism	rs56408111	-0.127269081	0.043917366	0.003756445
neutrophil	ieu-b-34	pulmonary embolism	rs567385	-0.129161181	0.043808032	0.003194806
neutrophil	ieu-b-34	pulmonary embolism	rs571497	-0.124125846	0.04389992	0.004691695
neutrophil	ieu-b-34	pulmonary embolism	rs5747308	-0.126903488	0.043957364	0.003889743
neutrophil	ieu-b-34	pulmonary embolism	rs5753576	-0.126160615	0.043907046	0.00406135
neutrophil	ieu-b-34	pulmonary embolism	rs58005484	-0.12221624	0.04376161	0.005225836
neutrophil	ieu-b-34	pulmonary embolism	rs58605236	-0.127990287	0.043912321	0.003560554
neutrophil	ieu-b-34	pulmonary embolism	rs58984522	-0.127494054	0.043905734	0.003686479

neutrophil	ieu-b-34	pulmonary embolism	rs59519784	-0.128670962	0.043872377	0.003358696
neutrophil	ieu-b-34	pulmonary embolism	rs60124939	-0.128538057	0.043891151	0.003405314
neutrophil	ieu-b-34	pulmonary embolism	rs6067411	-0.12671479	0.043881618	0.003881305
neutrophil	ieu-b-34	pulmonary embolism	rs609264	-0.127476418	0.043911634	0.003695813
neutrophil	ieu-b-34	pulmonary embolism	rs610578	-0.129174196	0.043864235	0.003230969
neutrophil	ieu-b-34	pulmonary embolism	rs61739285	-0.129041381	0.04390793	0.003293705
neutrophil	ieu-b-34	pulmonary embolism	rs61754230	-0.127045037	0.043855908	0.003769011
neutrophil	ieu-b-34	pulmonary embolism	rs619450	-0.127813153	0.043917315	0.003610664
neutrophil	ieu-b-34	pulmonary embolism	rs62011281	-0.124765494	0.04363699	0.004247535
neutrophil	ieu-b-34	pulmonary embolism	rs62189859	-0.130157264	0.043726844	0.002914653
neutrophil	ieu-b-34	pulmonary embolism	rs62360185	-0.128665551	0.043879068	0.003364872
neutrophil	ieu-b-34	pulmonary embolism	rs62375385	-0.128910043	0.043840654	0.003277651
neutrophil	ieu-b-34	pulmonary embolism	rs62429983	-0.127202343	0.043912375	0.003770728
neutrophil	ieu-b-34	pulmonary embolism	rs626416	-0.127187694	0.043920951	0.003781548
neutrophil	ieu-b-34	pulmonary embolism	rs632887	-0.129299731	0.04385296	0.003193371
neutrophil	ieu-b-34	pulmonary embolism	rs636317	-0.126272041	0.043868704	0.003996875
neutrophil	ieu-b-34	pulmonary embolism	rs6414435	-0.127736606	0.043929198	0.00364
neutrophil	ieu-b-34	pulmonary embolism	rs6432335	-0.127569699	0.04391027	0.003669763
neutrophil	ieu-b-34	pulmonary embolism	rs6463566	-0.127384839	0.043921055	0.003727893
neutrophil	ieu-b-34	pulmonary embolism	rs6468341	-0.125980407	0.04389454	0.004103808
neutrophil	ieu-b-34	pulmonary embolism	rs6476883	-0.128171461	0.043907032	0.003509809
neutrophil	ieu-b-34	pulmonary embolism	rs6500550	-0.126797467	0.04394343	0.003908292
neutrophil	ieu-b-34	pulmonary embolism	rs6503727	-0.12700382	0.043933593	0.003842461
neutrophil	ieu-b-34	pulmonary embolism	rs6672898	-0.127933019	0.043940856	0.003597184
neutrophil	ieu-b-34	pulmonary embolism	rs6678033	-0.127064613	0.044162211	0.004011895
neutrophil	ieu-b-34	pulmonary embolism	rs6679677	-0.12794409	0.043994879	0.003635638
neutrophil	ieu-b-34	pulmonary embolism	rs67175901	-0.129939723	0.043998922	0.003144494
neutrophil	ieu-b-34	pulmonary embolism	rs671835	-0.128760162	0.043896086	0.003353853
neutrophil	ieu-b-34	pulmonary embolism	rs6723009	-0.128098206	0.043897948	0.003521816
neutrophil	ieu-b-34	pulmonary embolism	rs6731993	-0.127509941	0.043957993	0.003723033
neutrophil	ieu-b-34	pulmonary embolism	rs6734238	-0.122320365	0.043994478	0.005429913
neutrophil	ieu-b-34	pulmonary embolism	rs6740847	-0.125196479	0.043902116	0.004348358
neutrophil	ieu-b-34	pulmonary embolism	rs6764912	-0.129187418	0.043928119	0.003272791
neutrophil	ieu-b-34	pulmonary embolism	rs6779340	-0.125864645	0.043850286	0.00410048
neutrophil	ieu-b-34	pulmonary embolism	rs68002561	-0.12569411	0.043724669	0.004044466
neutrophil	ieu-b-34	pulmonary embolism	rs68016381	-0.127024863	0.043907408	0.00381561
neutrophil	ieu-b-34	pulmonary embolism	rs68137036	-0.127683429	0.0439095	0.003638913
neutrophil	ieu-b-34	pulmonary embolism	rs6817881	-0.128662841	0.043895203	0.003377241
neutrophil	ieu-b-34	pulmonary embolism	rs6831590	-0.132897079	0.043644901	0.002327094
neutrophil	ieu-b-34	pulmonary embolism	rs6855981	-0.131004575	0.043741117	0.002744463
neutrophil	ieu-b-34	pulmonary embolism	rs6859727	-0.130091903	0.043958318	0.00308201
neutrophil	ieu-b-34	pulmonary embolism	rs6878780	-0.125497354	0.043807363	0.004173323
neutrophil	ieu-b-34	pulmonary embolism	rs6891328	-0.125296122	0.043743823	0.004179112
neutrophil	ieu-b-34	pulmonary embolism	rs6915310	-0.129528937	0.043931549	0.003193962

neutrophil	ieu-b-34	pulmonary embolism	rs6924387	-0.125022458	0.043833381	0.0043415
neutrophil	ieu-b-34	pulmonary embolism	rs6927569	-0.127847471	0.043982592	0.003651765
neutrophil	ieu-b-34	pulmonary embolism	rs6936191	-0.125367399	0.043865632	0.004263381
neutrophil	ieu-b-34	pulmonary embolism	rs694180	-0.12635144	0.043913331	0.004011053
neutrophil	ieu-b-34	pulmonary embolism	rs6985508	-0.129760466	0.043965513	0.003163218
neutrophil	ieu-b-34	pulmonary embolism	rs6998846	-0.124254803	0.043655804	0.004423913
neutrophil	ieu-b-34	pulmonary embolism	rs7005996	-0.126109179	0.043864167	0.004040363
neutrophil	ieu-b-34	pulmonary embolism	rs706809	-0.128652712	0.043911541	0.003391633
neutrophil	ieu-b-34	pulmonary embolism	rs7183988	-0.129540584	0.043873214	0.003150976
neutrophil	ieu-b-34	pulmonary embolism	rs7214290	-0.127188215	0.04391214	0.00377441
neutrophil	ieu-b-34	pulmonary embolism	rs7225843	-0.125676215	0.043897463	0.004197176
neutrophil	ieu-b-34	pulmonary embolism	rs723585	-0.125330637	0.043917142	0.004320003
neutrophil	ieu-b-34	pulmonary embolism	rs7235882	-0.126343243	0.043860731	0.003969759
neutrophil	ieu-b-34	pulmonary embolism	rs7237878	-0.127778691	0.043903309	0.003609007
neutrophil	ieu-b-34	pulmonary embolism	rs72664840	-0.127227037	0.043914237	0.00376545
neutrophil	ieu-b-34	pulmonary embolism	rs72726027	-0.124346585	0.04387903	0.004599093
neutrophil	ieu-b-34	pulmonary embolism	rs72731564	-0.128549796	0.043906893	0.003413897
neutrophil	ieu-b-34	pulmonary embolism	rs72789923	-0.128995857	0.043911873	0.003307553
neutrophil	ieu-b-34	pulmonary embolism	rs72790862	-0.124372551	0.043823032	0.004538814
neutrophil	ieu-b-34	pulmonary embolism	rs72803323	-0.128312319	0.043915678	0.003480279
neutrophil	ieu-b-34	pulmonary embolism	rs72825306	-0.126417428	0.043850903	0.003940422
neutrophil	ieu-b-34	pulmonary embolism	rs72832055	-0.130553815	0.043653226	0.002783365
neutrophil	ieu-b-34	pulmonary embolism	rs72836628	-0.12977815	0.043814446	0.003056532
neutrophil	ieu-b-34	pulmonary embolism	rs7283930	-0.127366691	0.043900641	0.003716784
neutrophil	ieu-b-34	pulmonary embolism	rs72901753	-0.127674466	0.04391357	0.003644427
neutrophil	ieu-b-34	pulmonary embolism	rs72973711	-0.125028138	0.043834577	0.004340794
neutrophil	ieu-b-34	pulmonary embolism	rs72978754	-0.126943686	0.043915161	0.00384437
neutrophil	ieu-b-34	pulmonary embolism	rs73000965	-0.125208107	0.043976386	0.004411024
neutrophil	ieu-b-34	pulmonary embolism	rs73049276	-0.127160358	0.043907271	0.00377818
neutrophil	ieu-b-34	pulmonary embolism	rs7322886	-0.126928657	0.043903201	0.003838928
neutrophil	ieu-b-34	pulmonary embolism	rs73271394	-0.128118365	0.043891735	0.003511975
neutrophil	ieu-b-34	pulmonary embolism	rs74076327	-0.128670626	0.043862261	0.003351469
neutrophil	ieu-b-34	pulmonary embolism	rs7409459	-0.12450326	0.043606067	0.004301217
neutrophil	ieu-b-34	pulmonary embolism	rs7488780	-0.129967011	0.043739394	0.002964503
neutrophil	ieu-b-34	pulmonary embolism	rs749780	-0.128656594	0.043921314	0.003397789
neutrophil	ieu-b-34	pulmonary embolism	rs75475627	-0.128178076	0.043936011	0.003529845
neutrophil	ieu-b-34	pulmonary embolism	rs7555995	-0.129007513	0.043862652	0.003269751
neutrophil	ieu-b-34	pulmonary embolism	rs7575465	-0.125663327	0.043806613	0.004123024
neutrophil	ieu-b-34	pulmonary embolism	rs7590263	-0.127643522	0.043908578	0.00364879
neutrophil	ieu-b-34	pulmonary embolism	rs75974417	-0.126985361	0.043904481	0.003824206
neutrophil	ieu-b-34	pulmonary embolism	rs763362	-0.128008009	0.043916339	0.003558993
neutrophil	ieu-b-34	pulmonary embolism	rs7639292	-0.127885283	0.04391765	0.00359199
neutrophil	ieu-b-34	pulmonary embolism	rs76428106	-0.124270436	0.04387646	0.00462173
neutrophil	ieu-b-34	pulmonary embolism	rs7658676	-0.128700999	0.043856264	0.00333968

neutrophil	ieu-b-34	pulmonary embolism	rs76603681	-0.130327762	0.043735754	0.002883513
neutrophil	ieu-b-34	pulmonary embolism	rs7679673	-0.127365745	0.04397288	0.003773972
neutrophil	ieu-b-34	pulmonary embolism	rs7684253	-0.124519232	0.043711713	0.004390559
neutrophil	ieu-b-34	pulmonary embolism	rs76873475	-0.128024335	0.043912149	0.003551589
neutrophil	ieu-b-34	pulmonary embolism	rs7687658	-0.127307499	0.04390105	0.003733131
neutrophil	ieu-b-34	pulmonary embolism	rs7705526	-0.129326352	0.044024161	0.003307398
neutrophil	ieu-b-34	pulmonary embolism	rs7738554	-0.126822697	0.043897198	0.003863657
neutrophil	ieu-b-34	pulmonary embolism	rs77552263	-0.130099169	0.043847839	0.003006623
neutrophil	ieu-b-34	pulmonary embolism	rs7776857	-0.126190919	0.043879363	0.00402928
neutrophil	ieu-b-34	pulmonary embolism	rs7777484	-0.126066468	0.04383366	0.004027246
neutrophil	ieu-b-34	pulmonary embolism	rs778732	-0.127537256	0.043913873	0.003681236
neutrophil	ieu-b-34	pulmonary embolism	rs780142	-0.126778558	0.04388234	0.003863997
neutrophil	ieu-b-34	pulmonary embolism	rs7803075	-0.129056816	0.043912307	0.003293083
neutrophil	ieu-b-34	pulmonary embolism	rs7815046	-0.126183064	0.043852448	0.004009081
neutrophil	ieu-b-34	pulmonary embolism	rs7816785	-0.123815943	0.04381557	0.004715631
neutrophil	ieu-b-34	pulmonary embolism	rs78285907	-0.127673724	0.043910788	0.003642479
neutrophil	ieu-b-34	pulmonary embolism	rs7846314	-0.129439078	0.044272147	0.003458828
neutrophil	ieu-b-34	pulmonary embolism	rs7852409	-0.125058145	0.043810967	0.004310543
neutrophil	ieu-b-34	pulmonary embolism	rs7866863	-0.127043639	0.043903525	0.003807315
neutrophil	ieu-b-34	pulmonary embolism	rs78738581	-0.127288358	0.04394336	0.003771751
neutrophil	ieu-b-34	pulmonary embolism	rs78813154	-0.130932393	0.043720193	0.002746421
neutrophil	ieu-b-34	pulmonary embolism	rs789858	-0.126010952	0.043856346	0.004062518
neutrophil	ieu-b-34	pulmonary embolism	rs79047930	-0.127955387	0.043896011	0.003557271
neutrophil	ieu-b-34	pulmonary embolism	rs7917772	-0.127724786	0.043925756	0.00364048
neutrophil	ieu-b-34	pulmonary embolism	rs7934719	-0.125683549	0.043885148	0.004184338
neutrophil	ieu-b-34	pulmonary embolism	rs7940176	-0.127453425	0.043908743	0.003699739
neutrophil	ieu-b-34	pulmonary embolism	rs7947419	-0.128462229	0.04390048	0.003431141
neutrophil	ieu-b-34	pulmonary embolism	rs796007	-0.126186067	0.043910338	0.00405667
neutrophil	ieu-b-34	pulmonary embolism	rs796056	-0.126938476	0.043948184	0.00387248
neutrophil	ieu-b-34	pulmonary embolism	rs7969023	-0.126939898	0.043889667	0.003824932
neutrophil	ieu-b-34	pulmonary embolism	rs7987258	-0.127368409	0.04390178	0.003717213
neutrophil	ieu-b-34	pulmonary embolism	rs8030089	-0.126727025	0.043910474	0.003901328
neutrophil	ieu-b-34	pulmonary embolism	rs8079215	-0.128743995	0.043852907	0.003326733
neutrophil	ieu-b-34	pulmonary embolism	rs8112528	-0.129617765	0.043791726	0.0030777
neutrophil	ieu-b-34	pulmonary embolism	rs820384	-0.127487822	0.04390376	0.003686613
neutrophil	ieu-b-34	pulmonary embolism	rs8705	-0.124722222	0.043830692	0.004433544
neutrophil	ieu-b-34	pulmonary embolism	rs915125	-0.12847926	0.04391497	0.003437518
neutrophil	ieu-b-34	pulmonary embolism	rs9265980	-0.128171623	0.044057304	0.003623519
neutrophil	ieu-b-34	pulmonary embolism	rs9267575	-0.127813279	0.043992914	0.003668835
neutrophil	ieu-b-34	pulmonary embolism	rs9277764	-0.124496567	0.043781758	0.004461097
neutrophil	ieu-b-34	pulmonary embolism	rs930232	-0.12916212	0.043927683	0.003278572
neutrophil	ieu-b-34	pulmonary embolism	rs9390461	-0.132162983	0.043649615	0.002463284
neutrophil	ieu-b-34	pulmonary embolism	rs9402685	-0.122148971	0.043918671	0.005414939
neutrophil	ieu-b-34	pulmonary embolism	rs9411293	-0.125937929	0.04384452	0.004073984

neutrophil	ieu-b-34	pulmonary embolism	rs9419387	-0.12888464	0.043855466	0.003294323
neutrophil	ieu-b-34	pulmonary embolism	rs9508005	-0.127030838	0.04388619	0.003797006
neutrophil	ieu-b-34	pulmonary embolism	rs9543219	-0.127566837	0.043910733	0.003670886
neutrophil	ieu-b-34	pulmonary embolism	rs9656395	-0.128235038	0.043897685	0.003486574
neutrophil	ieu-b-34	pulmonary embolism	rs9804265	-0.12458929	0.044092098	0.004718327
neutrophil	ieu-b-34	pulmonary embolism	rs981132	-0.128960463	0.04391963	0.003321712
neutrophil	ieu-b-34	pulmonary embolism	rs9819371	-0.128793815	0.04392597	0.003367159
neutrophil	ieu-b-34	pulmonary embolism	rs9842724	-0.126885385	0.04389802	0.003846809
neutrophil	ieu-b-34	pulmonary embolism	rs9885207	-0.125104877	0.043819462	0.004303586
neutrophil	ieu-b-34	pulmonary embolism	rs9905106	-0.12566018	0.043922777	0.004223945
neutrophil	ieu-b-34	pulmonary embolism	rs9965539	-0.12762633	0.043961817	0.003694677
neutrophil	ieu-b-34	pulmonary embolism	rs9977672	-0.13091324	0.043810912	0.002806788
neutrophil	ieu-b-34	pulmonary embolism	rs9988894	-0.127125572	0.043908981	0.003789091
neutrophil	ieu-b-34	pulmonary embolism	All	-0.12760534	0.043831546	0.003599661

Supplementary Table 10: Reverse MR results of peripheral blood WBC, neutrophils and lymphocytes with PE

outcome	exposure	method	nsnp	b	se	pval
white blood cell count	pulmonary embolism	MR Egger	11	-0.020386341	0.018112197	0.289471025
white blood cell count	pulmonary embolism	Weighted median	11	-0.009561929	0.006576751	0.145974421
white blood cell count	pulmonary embolism	Inverse variance weighted	11	-0.008260227	0.00563369	0.142588426
white blood cell count	pulmonary embolism	Simple mode	11	-0.021266217	0.010191449	0.063482771
white blood cell count	pulmonary embolism	Weighted mode	11	-0.008286381	0.007043831	0.266678419
lymphocyte cell count	pulmonary embolism	MR Egger	11	0.011397333	0.018565951	0.554489658
lymphocyte cell count	pulmonary embolism	Weighted median	11	0.001638837	0.00555361	0.767922064
lymphocyte cell count	pulmonary embolism	Inverse variance weighted	11	5.61E-05	0.005684356	0.992126617
lymphocyte cell count	pulmonary embolism	Simple mode	11	-0.000286119	0.008619331	0.974172295
lymphocyte cell count	pulmonary embolism	Weighted mode	11	0.001893651	0.006060372	0.761105962
neutrophil cell count	pulmonary embolism	MR Egger	11	-0.025880092	0.020231244	0.232816329
neutrophil cell count	pulmonary embolism	Weighted median	11	-0.008883353	0.00651436	0.172675493
neutrophil cell count	pulmonary embolism	Inverse variance weighted	11	-0.008768429	0.005904386	0.13752516
neutrophil cell count	pulmonary embolism	Simple mode	11	-0.014079359	0.008898099	0.14466562
neutrophil cell count	pulmonary embolism	Weighted mode	11	-0.008665871	0.006240779	0.195108307

Supplementary Table 11: MVMR results between circulating white blood cell count and PE

		IVW results									Mvmedian results					MR Egger results								est for heterogene		F-statistic
exposure	nsnp	b	se	pval	lo_ci	up_ci	OR	OR_lci95	OR_uci95	p.adjust	Estimate	Std_Error	CI_Lower	CI_Upper	P_Value	Estimate	Std_Error	CI_Lower	CI_Upper	P_Value	Residual_	Heteroger	Heteroger	Qstat	Qpval	F-statistic
basophil	77	-0.0652	0.092187	0.479433	-0.24588	0.11549	0.936884	0.782014	1.122424	0.479432661	0.008686	0.134364	-0.25466	0.272034	0.948455	-0.0818	0.102102	-0.28191	0.118318	0.423049	1.133442	701.4415	7.15E-06	/	/	12.05445384
monocyte	266	0.033767	0.040406	0.403321	-0.04543	0.112962	1.034344	0.955589	1.119589	0.479432661	-0.04793	0.056299	-0.15828	0.06241	0.394532	0.031815	0.040763	-0.04808	0.111709	0.435108	1.133442	701.4415	7.15E-06	/	/	56.75837667
lymphocyt	194	-0.17468	0.058417	0.002787	-0.28918	-0.06019	0.839722	0.748876	0.941588	0.01393481	-0.17476	0.07807	-0.32778	-0.02175	0.025184	-0.17898	0.05955	-0.2957	-0.06227	0.00265	1.133442	701.4415	7.15E-06	/	/	38.9050004
eosinophi	194	-0.07999	0.052703	0.129092	-0.18328	0.023311	0.923128	0.832531	1.023585	0.215152528	-0.19196	0.071501	-0.3321	-0.05182	0.007259	-0.08262	0.0532	-0.18689	0.021646	0.120402	1.133442	701.4415	7.15E-06	/	/	37.62041225
neutrophi	198	-0.10173	0.062321	0.102592	-0.22388	0.020415	0.903271	0.79941	1.020625	0.215152528	-0.12603	0.08165	-0.28606	0.033997	0.122688	-0.10564	0.063214	-0.22954	0.018255	0.094686	1.133442	701.4415	7.15E-06	/	/	28.55424132
(intercep	/	/	/	/	/	/	/	/	/	/	/	/	/	/	/	0.000466	0.001229	-0.00194	0.002875	0.704259	1.133442	701.4415	7.15E-06	701.0132	7.51E-06	/

Supplementary Table 12: MVMR results between circulating white blood cell count and PE adjusting for confounders BMI and CRP

					IVW results								mvmedian results						MR Egger results					Q-test for heterogeneity		F-statistic
	exposure	nsnp	b	se	pval	lo_ci	up_ci	OR	OR_lci95	OR_uci95	Estimate	Std_Error	CI_Lower	CI_Upper	P_Value	Estimate	Std_Error	CI_Lower	CI_Upper	P_Value	Residual_	Heteroger	Heteroger	Qstat	Qpval	F-statistic
model 1	lymphocyte cell coun	220	-0.10237	0.047927	0.032692	-0.1963	-0.00843	0.9027	0.821763	0.991607	-0.12863	0.068118	-0.26214	0.004878	0.058979	-0.0856	0.066114	-0.21518	0.043986	0.195438	1.069559	505.6285	0.019349	/	/	70.34970575
model 1	body mass index	235	0.307931	0.064803	2.02E-06	0.180917	0.434944	1.360607	1.198316	1.544877	0.319649	0.093792	0.13582	0.503478	0.000654	0.30879	0.064908	0.181573	0.436007	1.96E-06	1.069559	505.6285	0.019349	/	/	49.96278401
model 1	(intercept)	/	/	/	/	/	/	/	/	/	/	/	/	/	/	-0.00044	0.001201	-0.0028	0.001912	0.7124	1.069559	505.6285	0.019349	505.0908224	0.020141563	/
model 2	C-reactive protein |	73	0.098855	0.054384	0.069105	-0.00774	0.205447	1.103906	0.992293	1.228074	0.03232	0.077169	-0.11893	0.183569	0.675346	0.059034	0.059237	-0.05707	0.175137	0.318973	1.283298	736.1439	1.75E-16	/	/	52.66276972
model 2	lymphocyte cell coun	394	-0.10535	0.047694	0.027182	-0.19883	-0.01187	0.900009	0.819689	0.988199	-0.12768	0.057373	-0.24013	-0.01523	0.026055	-0.11353	0.047846	-0.2073	-0.01975	0.017656	1.283298	736.1439	1.75E-16	/	/	101.8138679
model 2	(intercept)	/	/	/	/	/	/	/	/	/	/	/	/	/	/	0.002026	0.001208	-0.00034	0.004394	0.09341	1.283298	736.1439	1.75E-16	740.4775706	7.34E-17	/
model 3	C-reactive protein |	25	-0.05411	0.103087	0.599632	-0.25616	0.147937	0.947324	0.774015	1.15944	-0.02549	0.148555	-0.31665	0.265674	0.863766	-0.17399	0.114054	-0.39753	0.049551	0.127132	1.024408	333.7128	0.261278	/	/	11.11075186
model 3	lymphocyte cell coun	116	-0.13954	0.059959	0.019952	-0.25706	-0.02202	0.869759	0.773323	0.978221	-0.12652	0.083608	-0.29039	0.037352	0.130226	-0.14998	0.059686	-0.26696	-0.033	0.011976	1.024408	333.7128	0.261278	/	/	56.58508981
model 3	body mass index || i	201	0.28925	0.076032	0.000142	0.140226	0.438273	1.335425	1.150534	1.550028	0.244276	0.112461	0.023857	0.464695	0.029848	0.185547	0.087146	0.014745	0.356349	0.033241	1.024408	333.7128	0.261278	/	/	32.6275083
model 3	(intercept)	/	/	/	/	/	/	/	/	/	/	/	/	/	/	0.003324	0.001396	0.000588	0.006059	0.017261	1.024408	333.7128	0.261278	339.1763326	0.198206553	/

Supplementary Table 13: SVMR analysis between lymphocyte subtype count and PE

Lymphocyte subgroup	id.exposure	id.outcome	outcome	method	nsnp	b	se	pval
Resting Treg count	ebi-a-GCST90001480	I9_PULMEMB	pulmonary embolism	MR Egger	13	0.026880719	0.019688638	0.199432013
Resting Treg count	ebi-a-GCST90001480	I9_PULMEMB	pulmonary embolism	Weighted median	13	0.017274664	0.018230554	0.343350152
Resting Treg count	ebi-a-GCST90001480	I9_PULMEMB	pulmonary embolism	Inverse variance we	13	0.011677691	0.014637526	0.42499158
Resting Treg count	ebi-a-GCST90001480	I9_PULMEMB	pulmonary embolism	Simple mode	13	-0.009516723	0.026346657	0.724220586
Resting Treg count	ebi-a-GCST90001480	I9_PULMEMB	pulmonary embolism	Weighted mode	13	0.017253577	0.018079009	0.35873996
Secreting Treg count	ebi-a-GCST90001492	I9_PULMEMB	pulmonary embolism	MR Egger	7	-0.07559375	0.040525072	0.121133913
Secreting Treg count	ebi-a-GCST90001492	I9_PULMEMB	pulmonary embolism	Weighted median	7	-0.017408799	0.018635002	0.350201292
Secreting Treg count	ebi-a-GCST90001492	I9_PULMEMB	pulmonary embolism	Inverse variance we	7	-0.010862083	0.015224303	0.475555005
Secreting Treg count	ebi-a-GCST90001492	I9_PULMEMB	pulmonary embolism	Simple mode	7	-0.020639582	0.023278504	0.409402518
Secreting Treg count	ebi-a-GCST90001492	I9_PULMEMB	pulmonary embolism	Weighted mode	7	-0.018092173	0.019557393	0.390613146
CD8+ T cell count	ebi-a-GCST90001592	I9_PULMEMB	pulmonary embolism	MR Egger	5	-1.245036265	0.879816261	0.251978543
CD8+ T cell count	ebi-a-GCST90001592	I9_PULMEMB	pulmonary embolism	Weighted median	5	-0.028149691	0.06384561	0.659283558
CD8+ T cell count	ebi-a-GCST90001592	I9_PULMEMB	pulmonary embolism	Inverse variance we	5	0.001750004	0.068432007	0.97959801
CD8+ T cell count	ebi-a-GCST90001592	I9_PULMEMB	pulmonary embolism	Simple mode	5	0.147974429	0.124567783	0.300585788
CD8+ T cell count	ebi-a-GCST90001592	I9_PULMEMB	pulmonary embolism	Weighted mode	5	-0.074234987	0.079195723	0.401642242
CD4+CD8dim T cell coun	ebi-a-GCST90001609	I9_PULMEMB	pulmonary embolism	MR Egger	8	0.053353807	0.107505536	0.637345043
CD4+CD8dim T cell coun	ebi-a-GCST90001609	I9_PULMEMB	pulmonary embolism	Weighted median	8	0.045007354	0.035081875	0.199518962
CD4+CD8dim T cell coun	ebi-a-GCST90001609	I9_PULMEMB	pulmonary embolism	Inverse variance we	8	0.033214087	0.02833948	0.241193991
CD4+CD8dim T cell coun	ebi-a-GCST90001609	I9_PULMEMB	pulmonary embolism	Simple mode	8	0.045822096	0.048547389	0.376668125
CD4+CD8dim T cell coun	ebi-a-GCST90001609	I9_PULMEMB	pulmonary embolism	Weighted mode	8	0.04763608	0.03823782	0.252921336
NKT count	ebi-a-GCST90001621	I9_PULMEMB	pulmonary embolism	MR Egger	7	-0.10162801	0.085976533	0.290335573
NKT count	ebi-a-GCST90001621	I9_PULMEMB	pulmonary embolism	Weighted median	7	-0.035912048	0.048715134	0.461010074
NKT count	ebi-a-GCST90001621	I9_PULMEMB	pulmonary embolism	Inverse variance we	7	-0.028188714	0.039720779	0.47790773
NKT count	ebi-a-GCST90001621	I9_PULMEMB	pulmonary embolism	Simple mode	7	-0.057412925	0.075663511	0.476716566
NKT count	ebi-a-GCST90001621	I9_PULMEMB	pulmonary embolism	Weighted mode	7	-0.05602023	0.065795873	0.427202196
B cell count	ebi-a-GCST90001642	I9_PULMEMB	pulmonary embolism	Wald ratio	1	0.056785556	0.120914769	0.638617299
HLA DR+ NK count	ebi-a-GCST90001648	I9_PULMEMB	pulmonary embolism	MR Egger	19	-0.027229188	0.041331798	0.518851525
HLA DR+ NK count	ebi-a-GCST90001648	I9_PULMEMB	pulmonary embolism	Weighted median	19	-0.030068209	0.023519839	0.201101738
HLA DR+ NK count	ebi-a-GCST90001648	I9_PULMEMB	pulmonary embolism	Inverse variance we	19	-0.036366959	0.017067399	0.033106855
HLA DR+ NK count	ebi-a-GCST90001648	I9_PULMEMB	pulmonary embolism	Simple mode	19	-0.045702575	0.03801704	0.244878575
HLA DR+ NK count	ebi-a-GCST90001648	I9_PULMEMB	pulmonary embolism	Weighted mode	19	-0.036672157	0.024992176	0.159537938

Supplementary Table 14: Description of SNPs for lymphocyte subtype cell counts incorporated after MR-presso deletion

Lymphocyte.subgroup	id.exposure	SNP	effect_al	other_all	effect_al	other_all	beta.expc	beta.outc	eaf.expos	eaf.outcc	remove	palindrom	ambiguous	id.outcom	pval.outc	se.outcom	outcome	mr_keep.c	pval_orig	data_sour	samplesiz	pos.expos	pval.expc	se.expos	chr.expos	exposure	mr_keep.e	pval_orig	data_sour	R2	F	Lymphocyt	action	mr_keep
Resting Treg AC	ebi-a-GCST90001480	rs1135041	C	T	C	T	-0.1819	0.031666	0.1605	0.06576	FALSE	FALSE	FALSE	I9_PULMEM	0.295508	0.03027	pulmonary	TRUE	reported	textfile	3405	38900569	2.64E-08	0.03263	2	|| id:eb	TRUE	reported	igd	0.009044	31.05819	Resting T	2	TRUE
Resting Treg AC	ebi-a-GCST90001480	rs1157971	A	G	A	G	1.149	0.020065	0.0044	0.075089	FALSE	FALSE	FALSE	I9_PULMEM	0.481637	0.028515	pulmonary	TRUE	reported	textfile	3405	2E+08	5.46E-11	0.1746	1	|| id:eb	TRUE	reported	igd	0.012559	43.28091	Resting T	2	TRUE
Resting Treg AC	ebi-a-GCST90001480	rs116758	T	G	T	G	0.2573	0.048505	0.09	0.010562	FALSE	FALSE	FALSE	I9_PULMEM	0.499931	0.071902	pulmonary	TRUE	reported	textfile	3405	39020877	6.23E-10	0.04149	2	|| id:eb	TRUE	reported	igd	0.011169	38.43594	Resting T	2	TRUE
Resting Treg AC	ebi-a-GCST90001480	rs1271261	T	G	T	G	-0.1407	0.002882	0.4887	0.567764	FALSE	FALSE	FALSE	I9_PULMEM	0.849738	0.01521	pulmonary	TRUE	reported	textfile	3405	38948860	3.40E-09	0.02375	2	|| id:eb	TRUE	reported	igd	0.010202	35.07566	Resting T	2	TRUE
Resting Treg AC	ebi-a-GCST90001480	rs1300484	T	C	T	C	0.274	-0.00495	0.1808	0.056972	FALSE	FALSE	FALSE	I9_PULMEM	0.878512	0.032362	pulmonary	TRUE	reported	textfile	3405	38903819	4.95E-19	0.03056	2	|| id:eb	TRUE	reported	igd	0.023064	80.34137	Resting T	2	TRUE
Resting Treg AC	ebi-a-GCST90001480	rs1403721	C	A	C	A	1.107	-0.00769	0.0044	0.009995	FALSE	FALSE	FALSE	I9_PULMEM	0.919879	0.076408	pulmonary	TRUE	reported	textfile	3405	1.99E+08	3.58E-10	0.176	1	|| id:eb	TRUE	reported	igd	0.011485	39.538	Resting T	2	TRUE
Resting Treg AC	ebi-a-GCST90001480	rs146620	T	G	T	G	1.803	0.083125	0.0032	0.005585	FALSE	FALSE	FALSE	I9_PULMEM	0.414285	0.101822	pulmonary	TRUE	reported	textfile	3405	1.99E+08	9.09E-12	0.2635	1	|| id:eb	TRUE	reported	igd	0.013564	46.79237	Resting T	2	TRUE
Resting Treg AC	ebi-a-GCST90001480	rs1758387	A	G	A	G	1.651	0.027128	0.0029	0.021825	FALSE	FALSE	FALSE	I9_PULMEM	0.60103	0.051878	pulmonary	TRUE	reported	textfile	3405	1.98E+08	6.94E-15	0.2111	1	|| id:eb	TRUE	reported	igd	0.017647	61.13114	Resting T	2	TRUE
Resting Treg AC	ebi-a-GCST90001480	rs182240	G	C	G	C	0.3864	-0.01432	0.0323	0.015252	FALSE	TRUE	FALSE	I9_PULMEM	0.817036	0.061876	pulmonary	TRUE	reported	textfile	3405	38955419	1.00E-08	0.06726	2	|| id:eb	TRUE	reported	igd	0.0096	32.98415	Resting T	2	TRUE
Resting Treg AC	ebi-a-GCST90001480	rs191753	T	C	T	C	1.493	0.028867	0.0041	0.022698	FALSE	FALSE	FALSE	I9_PULMEM	0.568662	0.050642	pulmonary	TRUE	reported	textfile	3405	1.96E+08	4.11E-13	0.2051	1	|| id:eb	TRUE	reported	igd	0.015324	52.95819	Resting T	2	TRUE
Resting Treg AC	ebi-a-GCST90001480	rs621425	A	G	A	G	0.3829	-0.00822	0.0332	0.037452	FALSE	FALSE	FALSE	I9_PULMEM	0.835101	0.039483	pulmonary	TRUE	reported	textfile	3405	38713988	8.84E-09	0.0664	2	|| id:eb	TRUE	reported	igd	0.009672	33.23375	Resting T	2	TRUE
Resting Treg AC	ebi-a-GCST90001480	rs6751481	C	T	C	T	0.2177	-0.01725	0.4699	0.526386	FALSE	FALSE	FALSE	I9_PULMEM	0.25069	0.015014	pulmonary	TRUE	reported	textfile	3405	38897810	3.10E-20	0.02348	2	|| id:eb	TRUE	reported	igd	0.024625	85.91431	Resting T	2	TRUE
Resting Treg AC	ebi-a-GCST90001480	rs7595807	C	G	C	G	-0.2006	-0.01524	0.1217	0.150782	FALSE	TRUE	FALSE	I9_PULMEM	0.474146	0.021296	pulmonary	TRUE	reported	textfile	3405	79203049	3.02E-08	0.03612	11	|| id:eb	TRUE	reported	igd	0.008977	30.82558	Resting T	2	TRUE
Secreting Treg AC	ebi-a-GCST90001492	rs113116	C	T	C	T	-1.446	0.04676	0.0047	0.010524	FALSE	FALSE	FALSE	I9_PULMEM	0.522041	0.073039	pulmonary	TRUE	reported	textfile	3405	1.99E+08	8.02E-16	0.1787	1	|| id:eb	TRUE	reported	igd	0.018867	65.43835	Secreting	2	TRUE
Secreting Treg AC	ebi-a-GCST90001492	rs113435	G	A	G	A	-2.032	0.07549	0.0032	0.004709	FALSE	FALSE	FALSE	I9_PULMEM	0.488494	0.108978	pulmonary	TRUE	reported	textfile	3405	1.98E+08	8.69E-19	0.2282	1	|| id:eb	TRUE	reported	igd	0.022756	79.24301	Secreting	2	TRUE
Secreting Treg AC	ebi-a-GCST90001492	rs1157971	A	G	A	G	-1.201	0.020065	0.0044	0.075089	FALSE	FALSE	FALSE	I9_PULMEM	0.481637	0.028515	pulmonary	TRUE	reported	textfile	3405	2E+08	5.94E-10	0.1934	1	|| id:eb	TRUE	reported	igd	0.011199	38.54055	Secreting	2	TRUE
Secreting Treg AC	ebi-a-GCST90001492	rs1389157	A	C	A	C	-1.455	-0.02072	0.0034	0.015896	FALSE	FALSE	FALSE	I9_PULMEM	0.729946	0.060031	pulmonary	TRUE	reported	textfile	3405	1.9E+08	9.40E-10	0.2371	1	|| id:eb	TRUE	reported	igd	0.010939	37.63637	Secreting	2	TRUE
Secreting Treg AC	ebi-a-GCST90001492	rs1403721	C	A	C	A	-1.292	-0.00769	0.0044	0.009995	FALSE	FALSE	FALSE	I9_PULMEM	0.919879	0.076408	pulmonary	TRUE	reported	textfile	3405	1.99E+08	3.77E-11	0.1947	1	|| id:eb	TRUE	reported	igd	0.012767	44.00864	Secreting	2	TRUE
Secreting Treg AC	ebi-a-GCST90001492	rs191753	T	C	T	C	-1.471	0.028867	0.0041	0.022698	FALSE	FALSE	FALSE	I9_PULMEM	0.568662	0.050642	pulmonary	TRUE	reported	textfile	3405	1.96E+08	1.16E-10	0.2275	1	|| id:eb	TRUE	reported	igd	0.01213	41.78375	Secreting	2	TRUE
Secreting Treg AC	ebi-a-GCST90001492	rs618396	T	C	T	C	0.3436	0.060946	0.0542	0.041028	FALSE	FALSE	FALSE	I9_PULMEM	0.104547	0.037547	pulmonary	TRUE	reported	textfile	3405	6094697	2.27E-09	0.05734	10	|| id:eb	TRUE	reported	igd	0.010436	35.88689	Secreting	2	TRUE
CD8+ T cell AC	ebi-a-GCST90001592	rs105178	A	G	A	G	-0.1775	-0.02553	0.3454	0.060176	FALSE	FALSE	FALSE	I9_PULMEM	0.415877	0.031383	pulmonary	TRUE	reported	textfile	3652	31378388	1.30E-08	0.03114	6	|| id:eb	TRUE	reported	igd	0.008818	32.47294	CD8+ T ce	2	TRUE
CD8+ T cell AC	ebi-a-GCST90001592	rs114968	C	T	C	T	-0.2013	-0.05628	0.299	0.057208	FALSE	FALSE	FALSE	I9_PULMEM	0.082459	0.03241	pulmonary	TRUE	reported	textfile	3652	32666960	1.30E-10	0.03123	6	|| id:eb	TRUE	reported	igd	0.011249	41.52462	CD8+ T ce	2	TRUE
CD8+ T cell AC	ebi-a-GCST90001592	rs251646	G	T	G	T	0.1719	0.023022	0.3758	0.443263	FALSE	FALSE	FALSE	I9_PULMEM	0.127629	0.015111	pulmonary	TRUE	reported	textfile	3652	31414327	6.74E-10	0.02778	6	|| id:eb	TRUE	reported	igd	0.010376	38.2692	CD8+ T ce	2	TRUE
CD8+ T cell AC	ebi-a-GCST90001592	rs310436	C	T	C	T	0.2047	-0.02914	0.7719	0.668102	FALSE	FALSE	FALSE	I9_PULMEM	0.067021	0.015912	pulmonary	TRUE	reported	textfile	3652	32602482	1.52E-10	0.03187	6	|| id:eb	TRUE	reported	igd	0.01117	41.23193	CD8+ T ce	2	TRUE
CD8+ T cell AC	ebi-a-GCST90001592	rs313068	T	C	T	C	0.1887	-0.00991	0.3428	0.450414	FALSE	FALSE	FALSE	I9_PULMEM	0.510507	0.015065	pulmonary	TRUE	reported	textfile	3652	31206206	3.52E-11	0.0284	6	|| id:eb	TRUE	reported	igd	0.011944	44.12342	CD8+ T ce	2	TRUE
CD4+CD8dim T cell A	ebi-a-GCST90001609	rs115623	C	A	C	A	-0.3539	0.041711	0.0427	0.013452	FALSE	FALSE	FALSE	I9_PULMEM	0.516559	0.064303	pulmonary	TRUE	reported	textfile	3652	87008667	3.08E-08	0.06378	2	|| id:eb	TRUE	reported	igd	0.00836	30.77189	CD4+CD8di	2	TRUE
CD4+CD8dim T cell A	ebi-a-GCST90001609	rs142158	C	T	C	T	-0.4078	0.011982	0.033	0.012475	FALSE	FALSE	FALSE	I9_PULMEM	0.859647	0.067763	pulmonary	TRUE	reported	textfile	3652	86997140	6.79E-09	0.07019	2	|| id:eb	TRUE	reported	igd	0.009158	33.73697	CD4+CD8di	2	TRUE
CD4+CD8dim T cell A	ebi-a-GCST90001609	rs184619	A	G	A	G	0.1841	0.010717	0.2824	0.283002	FALSE	FALSE	FALSE	I9_PULMEM	0.519978	0.016658	pulmonary	TRUE	reported	textfile	3652	32583813	1.04E-08	0.03209	6	|| id:eb	TRUE	reported	igd	0.008932	32.89503	CD4+CD8di	2	TRUE
CD4+CD8dim T cell A	ebi-a-GCST90001609	rs3529087	C	G	C	G	-0.364	-0.01574	0.1955	0.190417	FALSE	TRUE	FALSE	I9_PULMEM	0.410321	0.01912	pulmonary	TRUE	reported	textfile	3652	87023103	5.06E-31	0.03114	2	|| id:eb	TRUE	reported	igd	0.036065	136.5613	CD4+CD8di	2	TRUE
CD4+CD8dim T cell A	ebi-a-GCST90001609	rs483205	T	C	T	C	-0.2835	-0.01338	0.2329	0.285164	FALSE	FALSE	FALSE	I9_PULMEM	0.418772	0.016551	pulmonary	TRUE	reported	textfile	3652	87054693	4.98E-22	0.02919	2	|| id:eb	TRUE	reported	igd	0.025179	94.27576	CD4+CD8di	2	TRUE
CD4+CD8dim T cell A	ebi-a-GCST90001609	rs495902	G	A	G	A	0.2258	-0.01904	0.23	0.10944	FALSE	FALSE	FALSE	I9_PULMEM	0.431606	0.024204	pulmonary	TRUE	reported	textfile	3652	32383138	3.19E-11	0.03391	6	|| id:eb	TRUE	reported	igd	0.011996	44.31537	CD4+CD8di	2	TRUE
CD4+CD8dim T cell A	ebi-a-GCST90001609	rs675026	C	T	C	T	-0.2368	-0.02706	0.7508	0.774578	FALSE	FALSE	FALSE	I9_PULMEM	0.131173	0.017926	pulmonary	TRUE	reported	textfile	3652	86993528	3.55E-16	0.02891	2	|| id:eb	TRUE	reported	igd	0.01804	67.05471	CD4+CD8di	2	TRUE
CD4+CD8dim T cell A	ebi-a-GCST90001609	rs927140	G	A	G	A	0.1781	-0.00416	0.4458	0.539202	FALSE	FALSE	FALSE	I9_PULMEM	0.783036	0.0151	pulmonary	TRUE	reported	textfile	3652	32587588	3.58E-09	0.0301	6	|| id:eb	TRUE	reported	igd	0.009496	34.99105	CD4+CD8di	2	TRUE
NKT AC	ebi-a-GCST90001621	rs139249	G	A	G	A	0.4624	-0.01245	0.0316	0.01549	FALSE	FALSE	FALSE	I9_PULMEM	0.842155	0.062509	pulmonary	TRUE	reported	textfile	3653	1.02E+08	2.44E-10	0.07284	6	|| id:eb	TRUE	reported	igd	0.010911	40.27708	NKT AC	2	TRUE
NKT AC	ebi-a-GCST90001621	rs175154	T	C	T	C	0.1592	0.007609	0.2423	0.302884	FALSE	FALSE	FALSE	I9_PULMEM	0.641729	0.016355	pulmonary	TRUE	reported	textfile	3653	1.13E+08	4.03E-08	0.02893	11	|| id:eb	TRUE	reported	igd	0.008222	30.26575	NKT AC	2	TRUE
NKT AC	ebi-a-GCST90001621	rs7549421	A	G	A	G	0.4706	-0.02817	0.0328	0.062701	FALSE	FALSE	FALSE	I9_PULMEM	0.365088	0.031104	pulmonary	TRUE	reported	textfile	3653	1.02E+08	2.22E-11	0.07011	6	|| id:eb	TRUE	reported	igd	0.012183	45.03043	NKT AC	2	TRUE
NKT AC	ebi-a-GCST90001621	rs781909	G	A	G	A	0.1387	0.005497	0.5116	0.400466	FALSE	FALSE	FALSE	I9_PULMEM	0.719528	0.015308	pulmonary	TRUE	reported	textfile	3653	1.45E+08	2.84E-08	0.02493	8	|| id:eb	TRUE	reported	igd	0.008402	30.93645	NKT AC	2	TRUE
NKT AC	ebi-a-GCST90001621	rs7826811	T	C	T	C	-0.4086	0.036767	0.1096	0.014695	FALSE	FALSE	FALSE	I9_PULMEM	0.557083	0.062616	pulmonary	TRUE	reported	textfile	3653	1.13E+08	2.79E-25	0.03904	11	|| id:eb	TRUE	reported	igd	0.029114	109.4811	NKT AC	2	TRUE
NKT AC	ebi-a-GCST90001621	rs7885617	G	A	G	A	-0.2235	0.021561	0.1139	0.071259	FALSE	FALSE	FALSE	I9_PULMEM	0.461463	0.029278	pulmonary	TRUE	reported	textfile	3653	33805956	1.64E-08	0.03949	17	|| id:eb	TRUE	reported	igd	0.008692	32.01422	NKT AC	2	TRUE
NKT AC	ebi-a-GCST90001621	rs991662	C	T	C	T	0.1457	-0.00205	0.5123	0.260423	FALSE	FALSE	FALSE	I9_PULMEM	0.904688	0.017114	pulmonary	TRUE	reported	textfile	3653	33796260	4.13E-09	0.02472	17	|| id:eb	TRUE	reported	igd	0.00942	34.72037	NKT AC	2	TRUE
B cell AC	ebi-a-GCST90001642	rs128654	G	A	G	A	0.1537	0.008728	0.4777	0.207932	FALSE	FALSE	FALSE	I9_PULMEM	0.638618	0.018585	pulmonary	TRUE	reported	textfile	3653	1.09E+08	1.83E-10	0.02404	13	|| id:eb	TRUE	reported	igd	0.011066	40.8546	B cell AC	2	TRUE
HLA DR+ NK AC	ebi-a-GCST90001648	rs111504	A	G	A	G	0.3565	-0.01045	0.2913	0.395425	FALSE	FALSE	FALSE	I9_PULMEM	0.507932	0.015785	pulmonary	TRUE	reported	textfile	3580	1.62E+08	3.35E-38	0.02727	1	|| id:eb	TRUE	reported	igd	0.045563	170.8072	HLA DR+ N	2	TRUE

HLA DR+ NK AC	ebi-a-GCST90001648	rs1115166	T	C	T	C	0.242	-0.04322	0.0948	0.065705	FALSE	FALSE	FALSE	I9_PULMEM	0.154477	0.030356	pulmonary	TRUE	reported	textfile	3580	1.61E+08	1.04E-08	0.04218	1	||	id:eb	TRUE	reported	igd	0.009111	32.89841	HLA DR+ N	2	TRUE
HLA DR+ NK AC	ebi-a-GCST90001648	rs1203266	A	G	A	G	0.347	-0.01877	0.1566	0.355409	FALSE	FALSE	FALSE	I9_PULMEM	0.233646	0.015759	pulmonary	TRUE	reported	textfile	3580	1.62E+08	4.85E-25	0.03332	1	||	id:eb	TRUE	reported	igd	0.029404	108.3943	HLA DR+ N	2	TRUE
HLA DR+ NK AC	ebi-a-GCST90001648	rs1213572	T	G	T	G	0.212	-0.0175	0.3355	0.243749	FALSE	FALSE	FALSE	I9_PULMEM	0.319306	0.017577	pulmonary	TRUE	reported	textfile	3580	1.61E+08	1.58E-15	0.02648	1	||	id:eb	TRUE	reported	igd	0.017589	64.06091	HLA DR+ N	2	TRUE
HLA DR+ NK AC	ebi-a-GCST90001648	rs1389663	A	G	A	G	0.2889	0.027549	0.0656	0.027142	FALSE	FALSE	FALSE	I9_PULMEM	0.558856	0.04713	pulmonary	TRUE	reported	textfile	3580	1.62E+08	9.15E-09	0.05016	1	||	id:eb	TRUE	reported	igd	0.009181	33.15411	HLA DR+ N	2	TRUE
HLA DR+ NK AC	ebi-a-GCST90001648	rs1467188	A	G	A	G	0.3231	0.026724	0.1455	0.02672	FALSE	FALSE	FALSE	I9_PULMEM	0.570152	0.047064	pulmonary	TRUE	reported	textfile	3580	1.61E+08	2.44E-19	0.03573	1	||	id:eb	TRUE	reported	igd	0.022331	81.72693	HLA DR+ N	2	TRUE
HLA DR+ NK AC	ebi-a-GCST90001648	rs1488245	C	T	C	T	0.3538	-0.02766	0.0383	0.037714	FALSE	FALSE	FALSE	I9_PULMEM	0.4837	0.039496	pulmonary	TRUE	reported	textfile	3580	1.62E+08	4.63E-08	0.06459	1	||	id:eb	TRUE	reported	igd	0.008311	29.98765	HLA DR+ N	2	TRUE
HLA DR+ NK AC	ebi-a-GCST90001648	rs1503813	G	A	G	A	-0.1556	0.005182	0.2806	0.229808	FALSE	FALSE	FALSE	I9_PULMEM	0.771603	0.017851	pulmonary	TRUE	reported	textfile	3580	1.62E+08	2.46E-08	0.02784	1	||	id:eb	TRUE	reported	igd	0.00865	31.22037	HLA DR+ N	2	TRUE
HLA DR+ NK AC	ebi-a-GCST90001648	rs1572411	T	G	T	G	-0.1551	-0.01658	0.7013	0.764684	FALSE	FALSE	FALSE	I9_PULMEM	0.351062	0.017782	pulmonary	TRUE	reported	textfile	3580	1.57E+08	1.26E-08	0.02718	1	||	id:eb	TRUE	reported	igd	0.009014	32.54483	HLA DR+ N	2	TRUE
HLA DR+ NK AC	ebi-a-GCST90001648	rs3394112	T	C	T	C	0.169	-0.0053	0.2313	0.22072	FALSE	FALSE	FALSE	I9_PULMEM	0.770541	0.018186	pulmonary	TRUE	reported	textfile	3580	1.61E+08	1.32E-08	0.02967	1	||	id:eb	TRUE	reported	igd	0.008981	32.42617	HLA DR+ N	2	TRUE
HLA DR+ NK AC	ebi-a-GCST90001648	rs3580133	A	G	A	G	-0.2574	-0.00478	0.1018	0.018092	FALSE	FALSE	FALSE	I9_PULMEM	0.93185	0.055842	pulmonary	TRUE	reported	textfile	3580	1.62E+08	1.06E-09	0.04208	1	||	id:eb	TRUE	reported	igd	0.010343	37.39581	HLA DR+ N	2	TRUE
HLA DR+ NK AC	ebi-a-GCST90001648	rs4326638	C	T	C	T	-0.2321	0.024993	0.7291	0.544671	FALSE	FALSE	FALSE	I9_PULMEM	0.099715	0.015182	pulmonary	TRUE	reported	textfile	3580	1.61E+08	5.23E-17	0.02755	1	||	id:eb	TRUE	reported	igd	0.01944	70.93562	HLA DR+ N	2	TRUE
HLA DR+ NK AC	ebi-a-GCST90001648	rs6180182	A	T	A	T	-0.2249	0.020956	0.7669	0.651332	FALSE	TRUE	FALSE	I9_PULMEM	0.196215	0.016215	pulmonary	TRUE	reported	textfile	3580	1.62E+08	4.27E-14	0.02965	1	||	id:eb	TRUE	reported	igd	0.015817	57.50251	HLA DR+ N	2	TRUE
HLA DR+ NK AC	ebi-a-GCST90001648	rs6427594	C	T	C	T	-0.2702	-0.01302	0.9236	0.855664	FALSE	FALSE	FALSE	I9_PULMEM	0.544123	0.021471	pulmonary	TRUE	reported	textfile	3580	1.61E+08	4.61E-09	0.04599	1	||	id:eb	TRUE	reported	igd	0.00955	34.49858	HLA DR+ N	2	TRUE
HLA DR+ NK AC	ebi-a-GCST90001648	rs7163298	G	T	G	T	-0.6984	0.010451	0.2473	0.109281	FALSE	FALSE	FALSE	I9_PULMEM	0.666701	0.024267	pulmonary	TRUE	reported	textfile	3580	1.62E+08	########	0.02689	1	||	id:eb	TRUE	reported	igd	0.158552	674.1929	HLA DR+ N	2	TRUE
HLA DR+ NK AC	ebi-a-GCST90001648	rs7270407	T	C	T	C	-0.5729	-0.11278	0.0267	0.006259	FALSE	FALSE	FALSE	I9_PULMEM	0.240915	0.096172	pulmonary	TRUE	reported	textfile	3580	1.62E+08	1.60E-14	0.07429	1	||	id:eb	TRUE	reported	igd	0.01634	59.43664	HLA DR+ N	2	TRUE
HLA DR+ NK AC	ebi-a-GCST90001648	rs7527826	A	G	A	G	-0.1595	-0.00264	0.4496	0.44557	FALSE	FALSE	FALSE	I9_PULMEM	0.86174	0.015135	pulmonary	TRUE	reported	textfile	3580	1.62E+08	2.18E-10	0.02506	1	||	id:eb	TRUE	reported	igd	0.011189	40.48709	HLA DR+ N	2	TRUE
HLA DR+ NK AC	ebi-a-GCST90001648	rs7532602	A	G	A	G	-0.3597	0.046877	0.1946	0.108757	FALSE	FALSE	FALSE	I9_PULMEM	0.052459	0.024172	pulmonary	TRUE	reported	textfile	3580	1.61E+08	1.09E-29	0.0315	1	||	id:eb	TRUE	reported	igd	0.035143	130.3218	HLA DR+ N	2	TRUE
HLA DR+ NK AC	ebi-a-GCST90001648	rs7810566	G	A	G	A	-0.3481	-0.04573	0.0658	0.01111	FALSE	FALSE	FALSE	I9_PULMEM	0.523733	0.071716	pulmonary	TRUE	reported	textfile	3580	1.61E+08	9.66E-12	0.05093	1	||	id:eb	TRUE	reported	igd	0.012881	46.68937	HLA DR+ N	2	TRUE

Supplementary Table 15: Sensitivity analyses for the SVMR analysis between lymphocyte subtype count and PE

	MR Egger results			Q-test for heterogeneity			MR-PRESSO results			
exposure	egger_intercept	se	pval	Q	Q_df	Q_pval	Causal Estimate	Sd	T-stat	P-value
Resting Treg count	-0.012139748	0.010514431	0.27272915	4.670959475	12	0.96807648	0.011677691	0.009132298	1.278724242	0.225170949
Secreting Treg count	0.083080036	0.048202187	0.145390709	3.964801847	6	0.681439845	-0.010862083	0.012375779	-0.877688817	0.413872279
CD8+ T cell count	0.23557742	0.165837945	0.250550452	9.784769406	4	0.044213553	0.001750004	0.068432007	0.02557289	0.980822945
CD4+CD8dim T cell count	-0.005255009	0.027058976	0.85242322	3.796873972	7	0.802853802	0.033214087	0.02087161	1.591352422	0.155556796
NKT count	0.017536807	0.018208237	0.379718759	1.603233269	6	0.952344674	-0.028188714	0.020532437	-1.372886925	0.218888025
HLA DR+ NK count	-0.00287048	0.011825034	0.81110676	13.04373765	18	0.788968155	-0.036366959	0.014528879	-2.503080782	0.022165594

Supplementary Table 16: Leave-one-out analysis results for SVMR analysis of lymphocyte subtype count and PE

Lymphocyte.subgroup	id.exposure	outcome	SNP	b	se	p
Resting Treg count	ebi-a-GCST90001480	pulmonary embolism	rs113504153	0.013126136	0.0146945	0.3717127
Resting Treg count	ebi-a-GCST90001480	pulmonary embolism	rs11579717	0.008591363	0.0181262	0.6355173
Resting Treg count	ebi-a-GCST90001480	pulmonary embolism	rs116758203	0.011191174	0.0146576	0.4451628
Resting Treg count	ebi-a-GCST90001480	pulmonary embolism	rs12712613	0.0122783	0.0147736	0.4059181
Resting Treg count	ebi-a-GCST90001480	pulmonary embolism	rs13004842	0.012141457	0.0147512	0.4104624
Resting Treg count	ebi-a-GCST90001480	pulmonary embolism	rs140372175	0.012554547	0.0149782	0.4019254
Resting Treg count	ebi-a-GCST90001480	pulmonary embolism	rs146620586	0.009198378	0.0151555	0.5438939
Resting Treg count	ebi-a-GCST90001480	pulmonary embolism	rs17583875	0.010360266	0.0165419	0.5311163
Resting Treg count	ebi-a-GCST90001480	pulmonary embolism	rs182240059	0.012088245	0.0146991	0.4108595
Resting Treg count	ebi-a-GCST90001480	pulmonary embolism	rs191753228	0.009925413	0.0162261	0.5407402
Resting Treg count	ebi-a-GCST90001480	pulmonary embolism	rs62142582	0.012359279	0.0147873	0.4032645
Resting Treg count	ebi-a-GCST90001480	pulmonary embolism	rs6751481	0.0159656	0.0149788	0.286478
Resting Treg count	ebi-a-GCST90001480	pulmonary embolism	rs75958070	0.010431463	0.0147787	0.4802842
Resting Treg count	ebi-a-GCST90001480	pulmonary embolism	All	0.011677691	0.0146375	0.4249916
Secreting Treg count	ebi-a-GCST90001492	pulmonary embolism	rs113116201	-0.008716225	0.0159668	0.5851374
Secreting Treg count	ebi-a-GCST90001492	pulmonary embolism	rs113435341	-0.008558	0.0158775	0.5898849
Secreting Treg count	ebi-a-GCST90001492	pulmonary embolism	rs11579717	-0.006780709	0.0198401	0.7325251
Secreting Treg count	ebi-a-GCST90001492	pulmonary embolism	rs138915779	-0.014819111	0.0163803	0.3656283
Secreting Treg count	ebi-a-GCST90001492	pulmonary embolism	rs140372175	-0.012055214	0.0157553	0.4441808
Secreting Treg count	ebi-a-GCST90001492	pulmonary embolism	rs191753228	-0.008732029	0.0169742	0.6069521
Secreting Treg count	ebi-a-GCST90001492	pulmonary embolism	rs61839660	-0.014588177	0.0153742	0.3426862
Secreting Treg count	ebi-a-GCST90001492	pulmonary embolism	All	-0.010862083	0.0152243	0.475555
CD8+ T cell count	ebi-a-GCST90001592	pulmonary embolism	rs1051785	-0.007519658	0.0786353	0.9238168
CD8+ T cell count	ebi-a-GCST90001592	pulmonary embolism	rs114968045	-0.020405342	0.0672772	0.7616595
CD8+ T cell count	ebi-a-GCST90001592	pulmonary embolism	rs2516466	-0.041777524	0.0758336	0.5816949
CD8+ T cell count	ebi-a-GCST90001592	pulmonary embolism	rs3104369	0.068584814	0.0666311	0.3033283
CD8+ T cell count	ebi-a-GCST90001592	pulmonary embolism	rs3130685	0.025055474	0.0912239	0.7835781
CD8+ T cell count	ebi-a-GCST90001592	pulmonary embolism	All	0.001750004	0.068432	0.979598
CD4+CD8dim T cell count	ebi-a-GCST90001609	pulmonary embolism	rs115623211	0.036980867	0.0286906	0.1974141
CD4+CD8dim T cell count	ebi-a-GCST90001609	pulmonary embolism	rs142158385	0.035089363	0.0287608	0.22245
CD4+CD8dim T cell count	ebi-a-GCST90001609	pulmonary embolism	rs1846190	0.03049483	0.0298409	0.306822
CD4+CD8dim T cell count	ebi-a-GCST90001609	pulmonary embolism	rs35290870	0.029094371	0.0336581	0.3873637
CD4+CD8dim T cell count	ebi-a-GCST90001609	pulmonary embolism	rs4832052	0.02890145	0.032415	0.3726032
CD4+CD8dim T cell count	ebi-a-GCST90001609	pulmonary embolism	rs4959028	0.042045301	0.029385	0.152476
CD4+CD8dim T cell count	ebi-a-GCST90001609	pulmonary embolism	rs6750263	0.020002972	0.0305618	0.5127845
CD4+CD8dim T cell count	ebi-a-GCST90001609	pulmonary embolism	rs9271406	0.040328185	0.0300689	0.1798587
CD4+CD8dim T cell count	ebi-a-GCST90001609	pulmonary embolism	All	0.033214087	0.0283395	0.241194
NKT count	ebi-a-GCST90001621	pulmonary embolism	rs139249541	-0.028308558	0.0415551	0.4957261
NKT count	ebi-a-GCST90001621	pulmonary embolism	rs17515489	-0.041545654	0.0430706	0.3347478
NKT count	ebi-a-GCST90001621	pulmonary embolism	rs75494211	-0.010281929	0.0496962	0.8360912

NKT count	ebi-a-GCST90001621	pulmonary embolism	rs7819099	-0.038280317	0.0425736	0.3685689
NKT count	ebi-a-GCST90001621	pulmonary embolism	rs78268116	-0.023738207	0.0411263	0.5638025
NKT count	ebi-a-GCST90001621	pulmonary embolism	rs78856173	-0.021274986	0.0416832	0.6097733
NKT count	ebi-a-GCST90001621	pulmonary embolism	rs9916629	-0.030012261	0.0422072	0.4770412
NKT count	ebi-a-GCST90001621	pulmonary embolism	All	-0.028188714	0.0397208	0.4779077
B cell count	ebi-a-GCST90001642	pulmonary embolism	All	NA	NA	NA
HLA DR+ NK count	ebi-a-GCST90001648	pulmonary embolism	rs111504845	-0.037597916	0.0184968	0.0420863
HLA DR+ NK count	ebi-a-GCST90001648	pulmonary embolism	rs111516661	-0.033683929	0.0172276	0.050556
HLA DR+ NK count	ebi-a-GCST90001648	pulmonary embolism	rs12032664	-0.033452254	0.0184175	0.0693203
HLA DR+ NK count	ebi-a-GCST90001648	pulmonary embolism	rs12135728	-0.034322467	0.017441	0.0490769
HLA DR+ NK count	ebi-a-GCST90001648	pulmonary embolism	rs138966305	-0.037824743	0.0171616	0.0275218
HLA DR+ NK count	ebi-a-GCST90001648	pulmonary embolism	rs146718864	-0.038024528	0.0171858	0.0269281
HLA DR+ NK count	ebi-a-GCST90001648	pulmonary embolism	rs148824583	-0.0353661	0.0172704	0.0405814
HLA DR+ NK count	ebi-a-GCST90001648	pulmonary embolism	rs1503813	-0.036436341	0.0172595	0.0347643
HLA DR+ NK count	ebi-a-GCST90001648	pulmonary embolism	rs1572411	-0.039614333	0.0172597	0.0217223
HLA DR+ NK count	ebi-a-GCST90001648	pulmonary embolism	rs33941127	-0.036495508	0.0172862	0.0347509
HLA DR+ NK count	ebi-a-GCST90001648	pulmonary embolism	rs35801335	-0.036708985	0.0171205	0.0320202
HLA DR+ NK count	ebi-a-GCST90001648	pulmonary embolism	rs4326638	-0.031156911	0.0176799	0.0780218
HLA DR+ NK count	ebi-a-GCST90001648	pulmonary embolism	rs61801824	-0.032994184	0.0175667	0.0603508
HLA DR+ NK count	ebi-a-GCST90001648	pulmonary embolism	rs6427594	-0.040457008	0.0174752	0.0206073
HLA DR+ NK count	ebi-a-GCST90001648	pulmonary embolism	rs71632989	-0.043172809	0.0195941	0.0275692
HLA DR+ NK count	ebi-a-GCST90001648	pulmonary embolism	rs72704075	-0.038803024	0.0171563	0.0237137
HLA DR+ NK count	ebi-a-GCST90001648	pulmonary embolism	rs7527826	-0.038135408	0.0173504	0.027952
HLA DR+ NK count	ebi-a-GCST90001648	pulmonary embolism	rs7532602	-0.029888299	0.017646	0.0903092
HLA DR+ NK count	ebi-a-GCST90001648	pulmonary embolism	rs78180560	-0.037526009	0.0171263	0.0284418
HLA DR+ NK count	ebi-a-GCST90001648	pulmonary embolism	All	-0.036366959	0.0170674	0.0331069

Supplementary Table 17: MVMR results between lymphocyte subtype count and PE

					IVW results						mvmedian results					MR Egger results								est for heterogene		F-statistic
exposure	nsnp	b	se	pval	lo_ci	up_ci	OR	OR_lci95	OR_uci95	p.adjust	Estimate	Std_Error	CI_Lower	CI_Upper	P_Value	Estimate	Std_Error	CI_Lower	CI_Upper	P_Value	Residual_	Heteroger	Heteroger	Qstat	Qpval	F-statistic
Resting Treg A	13	0.026349	0.028805	0.360318	-0.03011	0.082806	1.026699	0.970341	1.086331	0.531105	0.033471	0.047324	-0.05928	0.126223	0.479395	0.064619	0.042221	-0.01813	0.147371	0.125899	0.792386	28.88228	0.977224	/	/	2.386411789
Secreting Treg	7	0.018342	0.029285	0.531105	-0.03906	0.07574	1.018511	0.961696	1.078682	0.531105	0.037256	0.050867	-0.06244	0.136953	0.463917	0.036031	0.037337	-0.03715	0.10921	0.334539	0.792386	28.88228	0.977224	/	/	2.144208507
CD8+ T cell AC	4	0.026726	0.041715	0.521737	-0.05504	0.108488	1.027086	0.946451	1.114591	0.531105	-0.07558	0.071341	-0.2154	0.06425	0.28943	0.048214	0.052574	-0.05483	0.151257	0.359102	0.792386	28.88228	0.977224	/	/	2.716690617
CD4+CD8dim T c	8	0.033563	0.023074	0.145785	-0.01166	0.078788	1.034132	0.988406	1.081974	0.510249	0.052069	0.038356	-0.02311	0.127245	0.17461	0.036165	0.028203	-0.01911	0.091441	0.199737	0.792386	28.88228	0.977224	/	/	7.522820364
NKT AC	7	-0.02594	0.031601	0.411799	-0.08787	0.036002	0.974398	0.915877	1.036657	0.531105	0.031455	0.057087	-0.08043	0.143344	0.581633	-0.00878	0.039964	-0.08711	0.069548	0.826115	0.792386	28.88228	0.977224	/	/	3.992810724
B cell AC	1	0.045118	0.064134	0.481746	-0.08059	0.170822	1.046152	0.922576	1.186279	0.531105	0.070785	0.092513	-0.11054	0.252108	0.444195	0.034411	0.078539	-0.11952	0.188345	0.66129	0.792386	28.88228	0.977224	/	/	1.41505128
HLA DR+ NK AC	19	-0.05029	0.01626	0.001984	-0.08216	-0.01842	0.950957	0.921128	0.981753	0.013891	-0.07037	0.027505	-0.12428	-0.01646	0.010512	-0.03984	0.020846	-0.08069	0.00102	0.055999	0.792386	28.88228	0.977224	/	/	8.25828518
(intercept)	/	/	/	/	/	/	/	/	/	/	/	/	/	/	/	-0.00741	0.004531	-0.0163	0.001467	0.101782	0.792386	28.88228	0.977224	30.96502	0.95628	/
